# Supplementary material for: Mass spectrometry-based absolute quantification of amyloid proteins in pathology tissue specimens: Merits and limitations
Source: PLoS One. 2020 Jul 1;15(7):e0235143. doi: 10.1371/journal.pone.0235143 (PMC7329117; doi:10.1371/journal.pone.0235143)
Supplement: S1 Appendix — (PDF) [file pone.0235143.s001.pdf]

Supplementary file
References for the determination of peptide sequeces

Amyloid A
[1] Steinkasserer A, Weiss EH, Schwaeble W, Linke RP: Heterogeneity of human serum amyloid A protein. Five different variants from one individual demonstrated by cDNA sequence analysis. Biochem J 1990, 268:187-93.
[2] Baba S, Takahashi T, Kasama T, Shirasawa H: Identification of two novel amyloid A protein subsets coexisting in an individual patient of AA-amyloidosis. Biochim Biophys Acta 1992, 1180:195-200.
[3] Lu J, Yu Y, Zhu I, Cheng Y, Sun PD: Structural mechanism of serum amyloid A-mediated inflammatory amyloidosis. Proc Natl Acad Sci U S A 2014, 111:5189-94.

Transthyretin
[1] Ueno S, Uemichi T, Takahashi N, Soga F, Yorifuji S, Tarui S: Two novel variants of transthyretin identified in Japanese cases with familial amyloidotic polyneuropathy: Transthyretin (Glu42 to Gly) and transthyretin (Ser50 to Arg). Biochemical and Biophysical Research Communications 1990, 169:1117-21.
[2] Almeida MR, Ferlini A, Forabosco A, Gawinowicz M, Costa PP, Salvi F, Plasmati R, Tassinari CA, Altland K, Saraiva MJ: Two transthyretin variants (TTR Ala-49 and TTR Gln-89) in two Sicilian kindreds with hereditary amyloidosis. Hum Mutat 1992, 1:211-5.
[3] Jacobson DR, Buxbaum JN: A double-variant transthyretin allele (Ser 6, Ile 33) in the Israeli patient "SKO" with familial amyloidotic polyneuropathy. Hum Mutat 1994, 3:254-60.
[4] Misrahi AM, Plante V, Lalu T, Serre I, Adams D, Lacroix DC, Saïd G: New transthyretin variants SER 91 and SER 116 associated with familial amyloidotic polyneuropathy. Human Mutation 1998, 12:71-.
[5] Théberge R, Connors L, Share J, Skinner M, Falk RH, Costello CE: A new amyloidogenic transthyretin variant (Vail 22Ala) found in a compound heterozygous patient. Amyloid 2009, 6:54-8.
[6] Lashuel HA, Wurth C, Woo L, Kelly JW: The most pathogenic transthyretin variant, L55P, forms amyloid fibrils under acidic conditions and protofilaments under physiological conditions. Biochemistry 1999, 38:13560-73.
[7] Lim A, Prokaeva T, McComb ME, O'Connor PB, Theberge R, Connors LH,

Skinner M, Costello CE: Characterization of transthyretin variants in familial transthyretin amyloidosis by mass spectrometric peptide mapping and DNA sequence analysis. Anal Chem 2002, 74:741-51.
[8] Bergen HR, 3rd, Zeldenrust SR, Butz ML, Snow DS, Dyck PJ, Dyck PJ, Klein CJ, O'Brien JF, Thibodeau SN, Muddiman DC: Identification of transthyretin variants by sequential proteomic and genomic analysis. Clin Chem 2004, 50:1544-52.
[9] Morais-de-Sa E, Neto-Silva RM, Pereira PJ, Saraiva MJ, Damas AM: The binding of 2,4-dinitrophenol to wild-type and amyloidogenic transthyretin. Acta Crystallogr D Biol Crystallogr 2006, 62:512-9.

Beta-2-microglobulin

[1] Wani MA, Haynes LD, Kim J, Bronson CL, Chaudhury C, Mohanty S, Waldmann TA, Robinson JM, Anderson CL: Familial hypercatabolic hypoproteinemia caused by deficiency of the neonatal Fc receptor, FcRn, due to a mutant beta2-microglobulin gene. Proc Natl Acad Sci U S A 2006, 103:5084-9.
[2] Ricagno S, Raimondi S, Giorgetti S, Bellotti V, Bolognesi M: Human beta-2 microglobulin W60V mutant structure: Implications for stability and amyloid aggregation. Biochem Biophys Res Commun 2009, 380:543-7.

Apolipoprotein

[1] von Eckardstein A, Funke H, Walter M, Altland K, Benninghoven A, Assmann G: Structural analysis of human apolipoprotein A-I variants. Amino acid substitutions are nonrandomly distributed throughout the apolipoprotein A-I primary structure. J Biol Chem 1990, 265:8610-7.
[2] Lohse P, Kindt MR, Rader DJ, Brewer HB, Jr.: Human plasma apolipoproteins A-IV-0 and A-IV-3. Molecular basis for two rare variants of apolipoprotein A-IV-1. J Biol Chem 1990, 265:12734-9.
[3] Lohse P, Kindt MR, Rader DJ, Brewer HB, Jr.: Three genetic variants of human plasma apolipoprotein A-IV. apoA-IV-1(Thr347----Ser), apoA-IV-0(Lys167----Glu,Gln360----His), and apoA-IV-3(Glu165----Lys). J Biol Chem 1991, 266:13513-8.
[4] Kamboh MI, Williams ER, Law JC, Aston CE, Bunker CH, Ferrell RE, Pollitzer WS: Molecular basis of a unique African variant (A-IV 5) of human apolipoprotein A-IV and its significance in lipid metabolism. Genet Epidemiol

1992, 9:379-88.
[5] Menzel HJ, Dieplinger H, Sandholzer C, Karadi I, Utermann G, Csaszar A: Apolipoprotein A-IV polymorphism in the Hungarian population: gene frequencies, effect on lipid levels, and sequence of two new variants. Hum Mutat 1995, 5:58-65.
[6] Deeb SS, Nevin DN, Iwasaki L, Brunzell JD: Two novel apolipoprotein A-IV variants in individuals with familial combined hyperlipidemia and diminished levels of lipoprotein lipase activity. Human Mutation 1996, 8:319-25.
[7] Lohse P, Mann WA, Stein EA, Brewer HB, Jr.: Apolipoprotein E-4Philadelphia (Glu13----Lys,Arg145----Cys). Homozygosity for two rare point mutations in the apolipoprotein E gene combined with severe type III hyperlipoproteinemia. J Biol Chem 1991, 266:10479-84.
[8] van den Maagdenberg AM, Weng W, de Bruijn IH, de Knijff P, Funke H, Smelt AH, Gevers Leuven JA, van't Hooft FM, Assmann G, Hofker MH, et al.: Characterization of five new mutants in the carboxyl-terminal domain of human apolipoprotein E: no cosegregation with severe hyperlipidemia. Am J Hum Genet 1993, 52:937-46.
[9] Kang AK, Jenkins DJA, Wolever TMS, Huff MW, Maguire GF, Connelly PW, Hegele RA: Apolipoprotein E R112; R251G: a carboxy-terminal variant found in patients with hyperlipidemia and coronary heart disease. Mutation Research/Mutation Research Genomics 1997, 382:57-65.
[10] von Eckardstein A, Funke H, Henke A, Altland K, Benninghoven A, Assmann G: Apolipoprotein A-I variants. Naturally occurring substitutions of proline residues affect plasma concentration of apolipoprotein A-I. J Clin Invest 1989, 84:1722-30.

Gelsolin
[1] Ghiso J, Haltia M, Prelli F, Novello J, Frangione B: Gelsolin variant (Asn-187) in familial amyloidosis, Finnish type. Biochem J 1990, 272:827-30.
[2] de la Chapelle A, Tolvanen R, Boysen G, Santavy J, Bleeker-Wagemakers L, Maury CP, Kere J: Gelsolin-derived familial amyloidosis caused by asparagine or tyrosine substitution for aspartic acid at residue 187. Nat Genet 1992, 2:157-60.

Fibrinogen alpha-chain
[1] Benson MD, Liepnieks J, Uemichi T, Wheeler G, Correa R: Hereditary renal

amyloidosis associated with a mutant fibrinogen alpha-chain. Nat Genet 1993, 3:252-5.
[2] Koopman J, Haverkate F, Grimbergen J, Lord ST, Mosesson MW, DiOrio JP, Siebenlist KS, Legrand C, Soria J, Soria C, et al.: Molecular basis for fibrinogen Dusart (A alpha 554 Arg-->Cys) and its association with abnormal fibrin polymerization and thrombophilia. J Clin Invest 1993, 91:1637-43.
