# Supplementary material for: Mass spectrometry-based absolute quantification of amyloid proteins in pathology tissue specimens: Merits and limitations
Source: PLoS One. 2020 Jul 1;15(7):e0235143. doi: 10.1371/journal.pone.0235143 (PMC7329117; doi:10.1371/journal.pone.0235143)
Supplement: S2 Appendix — (PDF) [file pone.0235143.s002.pdf]

S2 Appendix Signal linearity of each MS-QBIC peptide measured by the mass spectrometry

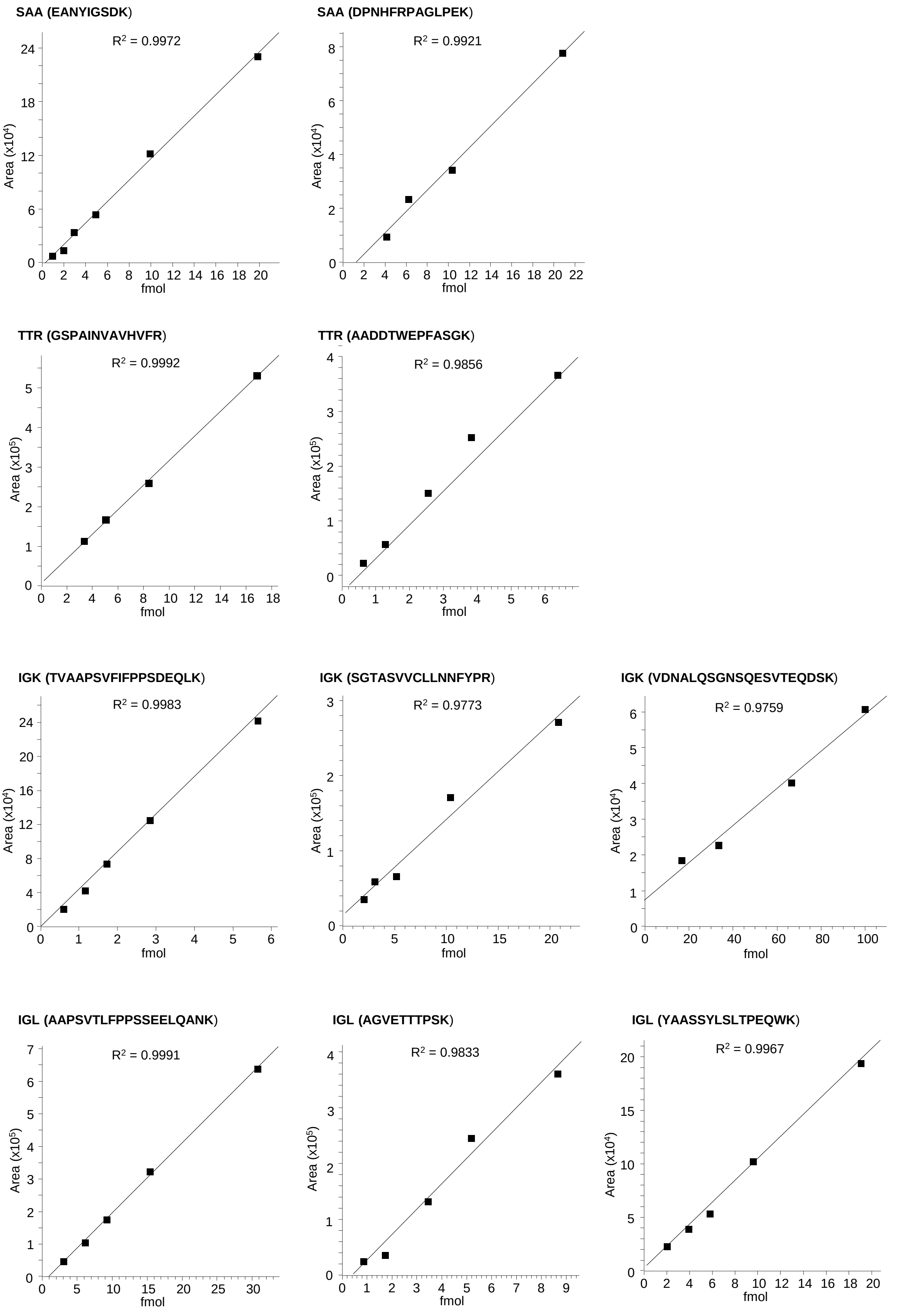

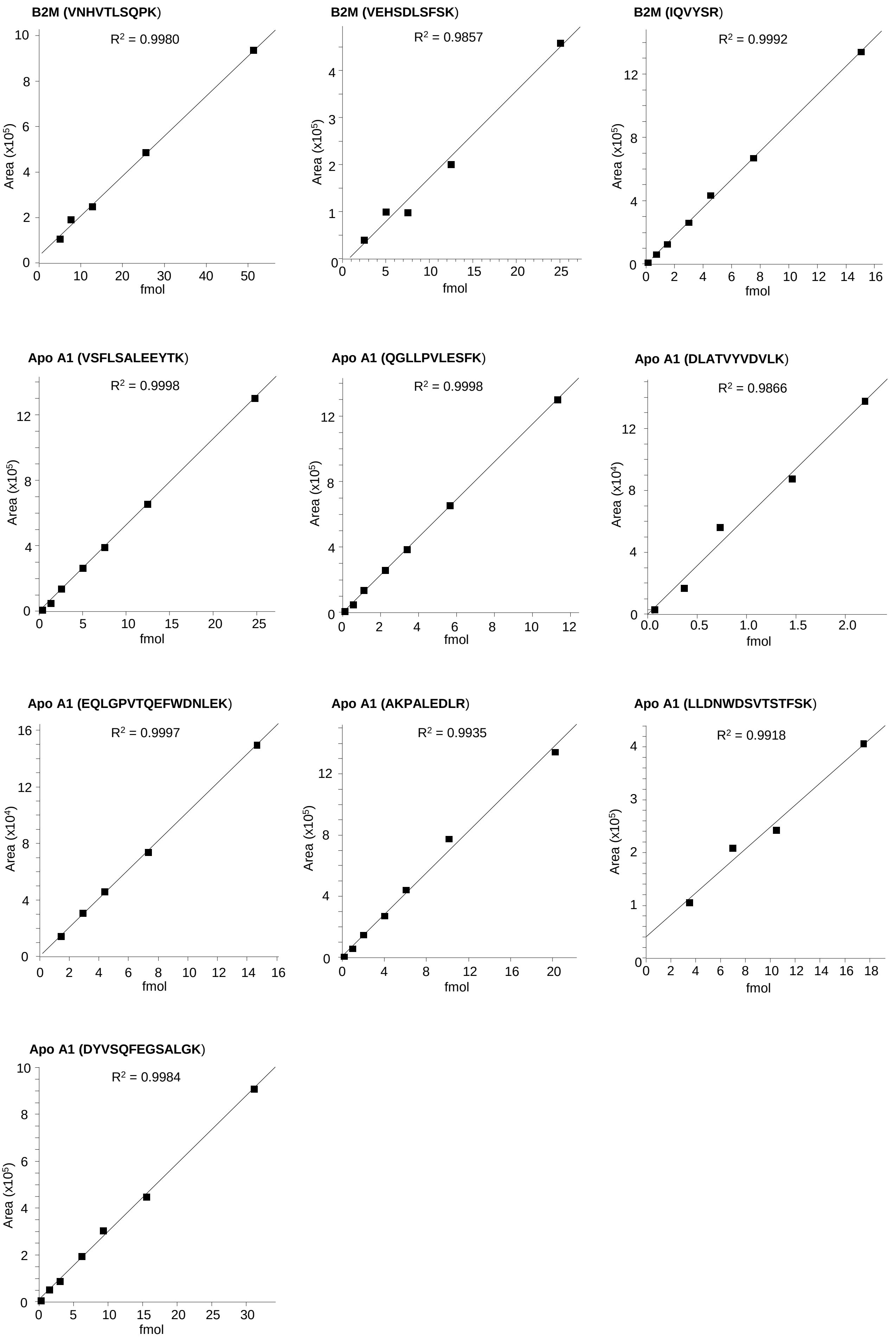
B2M (VNHVTLSQPK)
R² = 0.9980
Area (x10⁵)
fmol
B2M (VEHSDLSFSK)
R² = 0.9857
Area (x10⁵)
fmol
B2M (IQVYSR)
R² = 0.9992
Area (x10⁵)
fmol
Apo A1 (VSFLSALEEYTK)
R² = 0.9998
Area (x10⁵)
fmol
Apo A1 (QGLLPVLESFK)
R² = 0.9998
Area (x10⁵)
fmol
Apo A1 (DLATVYVDVLK)
R² = 0.9866
Area (x10⁴)
fmol
Apo A1 (EQLGPVTQEFWDNLEK)
R² = 0.9997
Area (x10⁴)
fmol
Apo A1 (AKPALEDLR)
R² = 0.9935
Area (x10⁵)
fmol
Apo A1 (LLDNWDSVTSTFSK)
R² = 0.9918
Area (x10⁵)
fmol
Apo A1 (DYVSQFEGSALGK)
R² = 0.9984
Area (x10⁵)
fmol

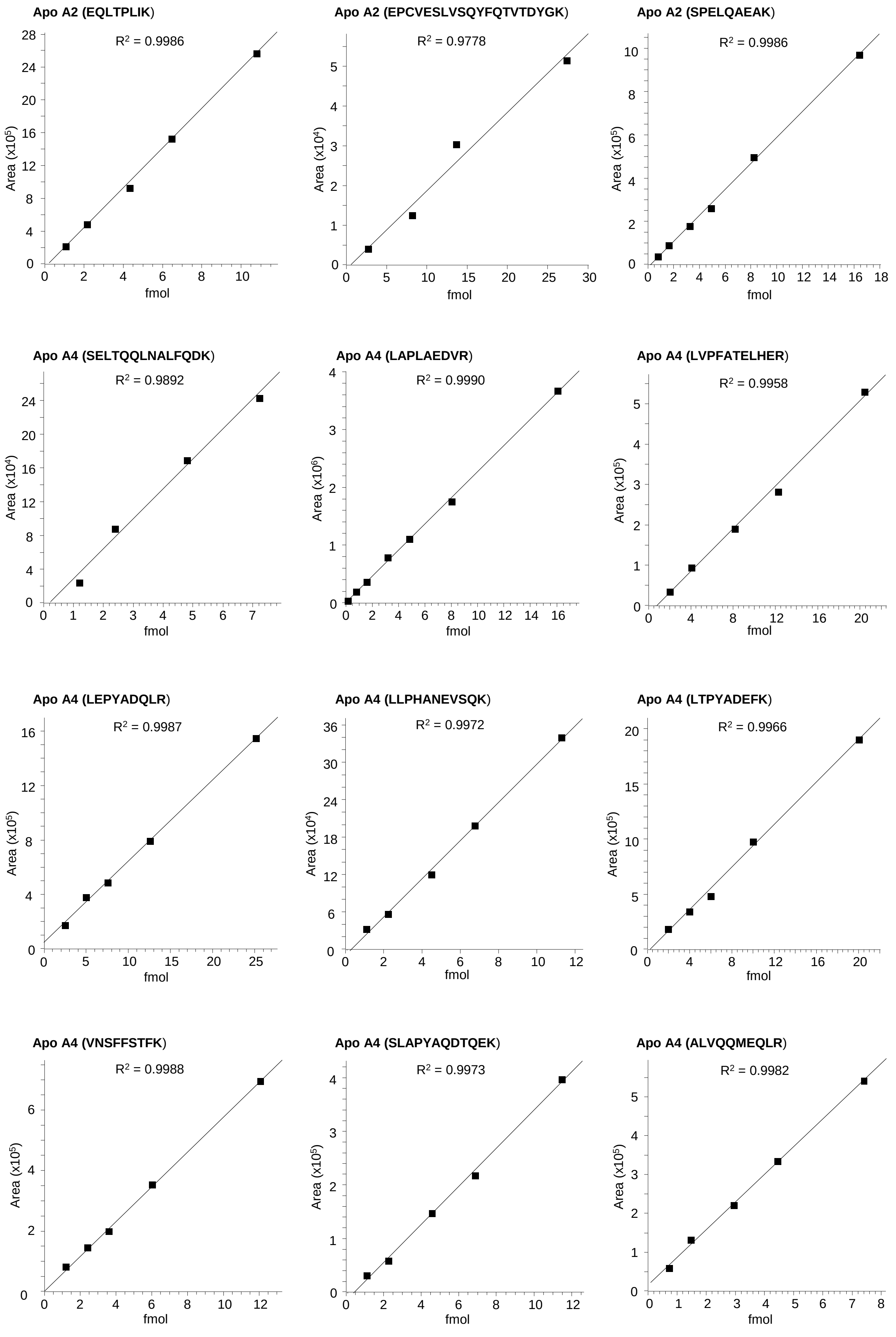

Apo A2 (EQLTPLIK)
R2 = 0.9986
Area (x105)
fmol
Apo A2 (EPCVESLVSQYFQTVTDYGK)
R2 = 0.9778
Area (x104)
fmol
Apo A2 (SPELQAEAK)
R2 = 0.9986
Area (x105)
fmol
Apo A4 (SELTQQLNALFQDK)
R2 = 0.9892
Area (x104)
fmol
Apo A4 (LAPLAEDVR)
R2 = 0.9990
Area (x106)
fmol
Apo A4 (LVPFATELHER)
R2 = 0.9958
Area (x105)
fmol
Apo A4 (LEPYADQLR)
R2 = 0.9987
Area (x105)
fmol
Apo A4 (LLPHANEVSQK)
R2 = 0.9972
Area (x104)
fmol
Apo A4 (LTPYADEFK)
R2 = 0.9966
Area (x105)
fmol
Apo A4 (VNSFFSTFK)
R2 = 0.9988
Area (x105)
fmol
Apo A4 (SLAPYAQDTQEK)
R2 = 0.9973
Area (x105)
fmol
Apo A4 (ALVQQMEQLR)
R2 = 0.9982
Area (x105)
fmol

Apo A4 (IDQNVEELK)

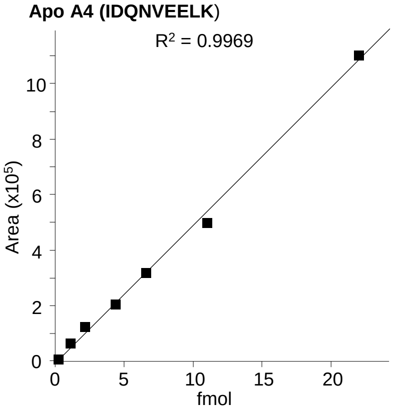

Apo A4 (IDQTVEELR)

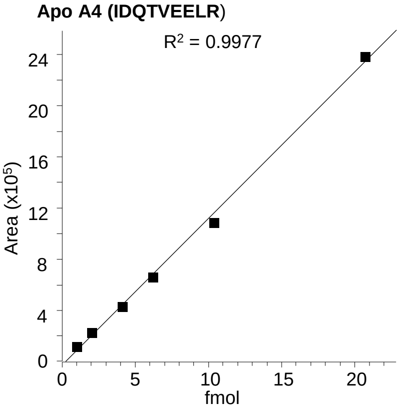

Apo E (AATVGSLAGQPLQER)

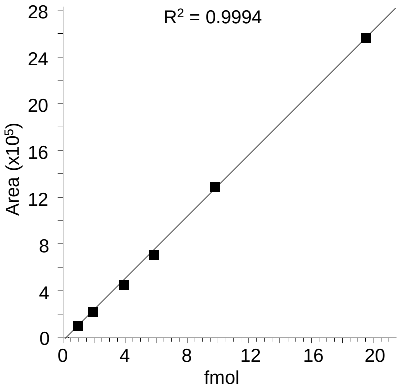

Apo E (LGPLVEQGR)

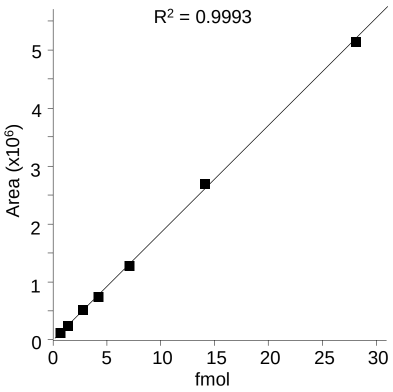

Apo E (FWDYLR)

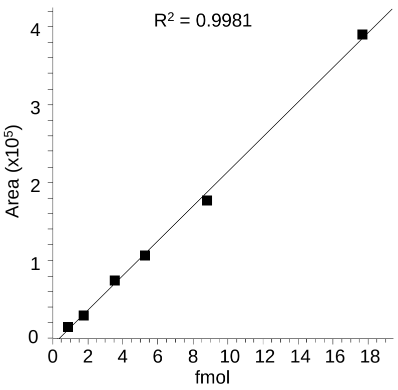

Apo E (ALMDETMK)

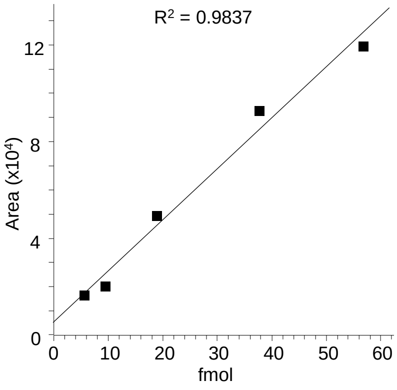

Apo E (AQAWGER)

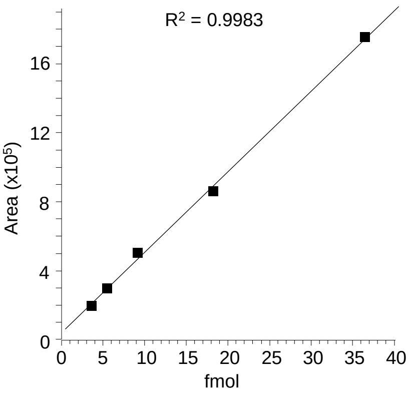

Apo E (QQTEWQSGQR)

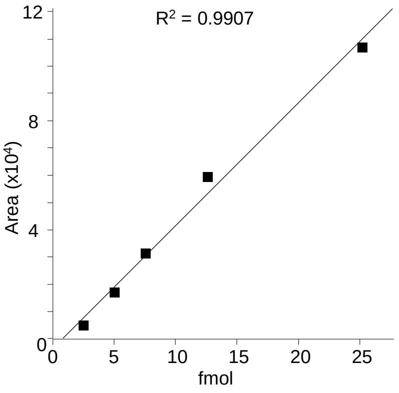

Gelsolin (HVVPNEVVVQR)

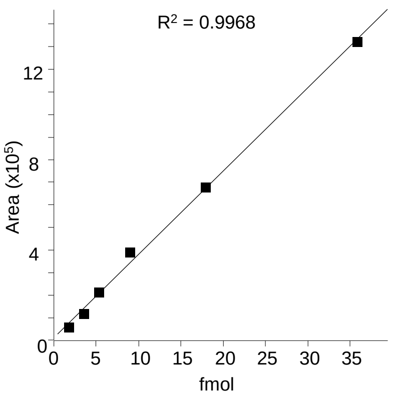

Gelsolin (EVQGFESATFLGYFK)

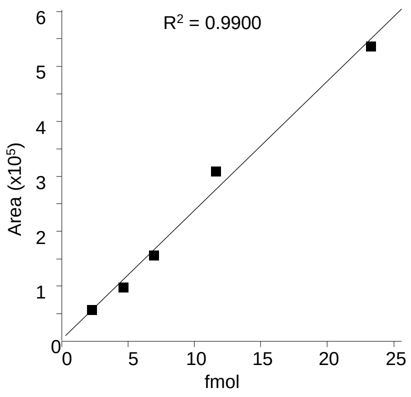

Gelsolin (TGAQELLR)

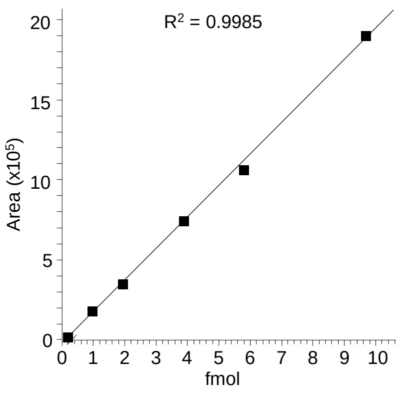

Gelsolin (QTQVSVLPEGGETPLFK)

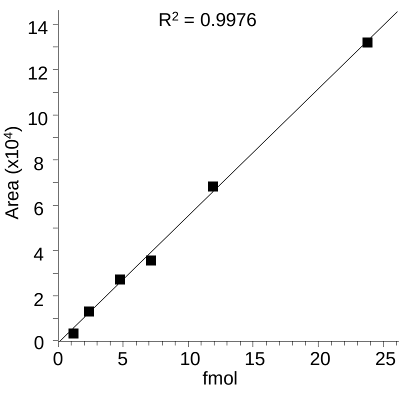

Gelsolin (AGALNSNDAFVLK)

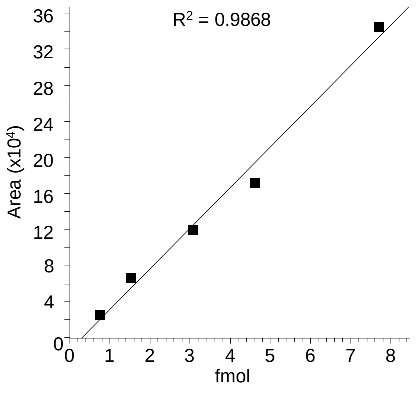

Gelsolin (AVEVLPK)

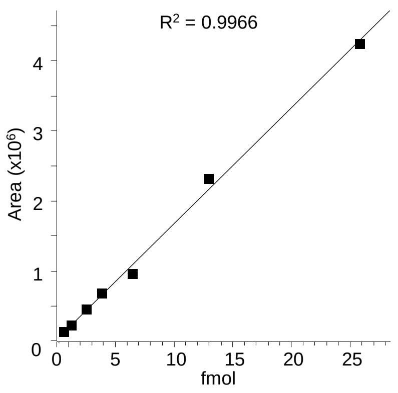

Gelsolin (TPITVVK)

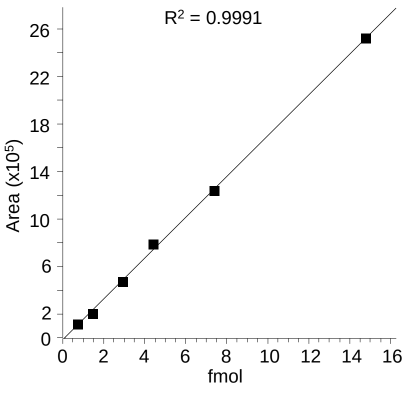

Gelsolin (EPGLQIWR)

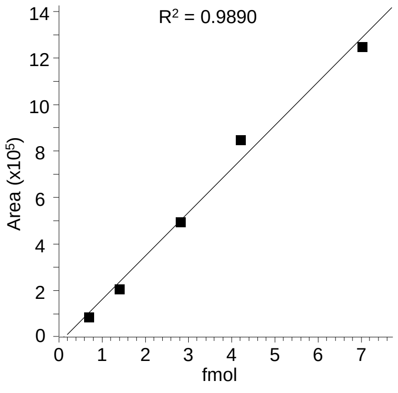

Gelsolin (AQPVQVAEGSEPDGFWEALGGK)

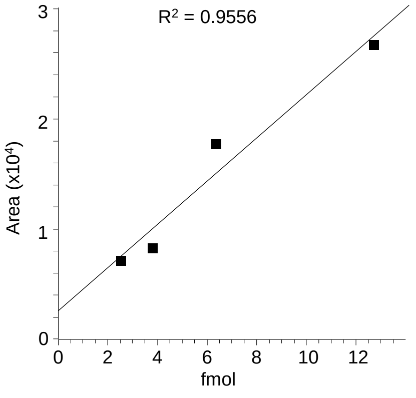

FGA (GLIDEVNQDFTNR)

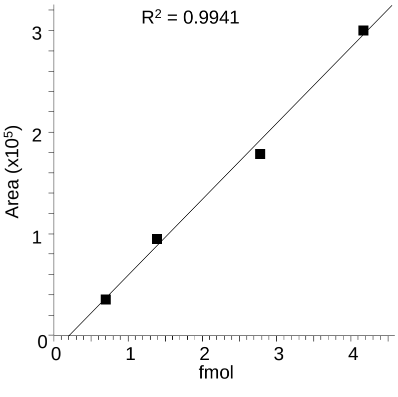

FGA (HPDEAAFFDTASTGK)

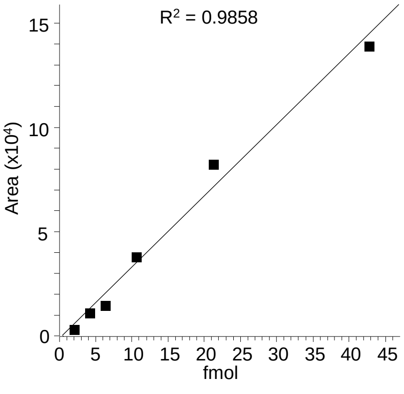

FGA (QLEQVIAK)

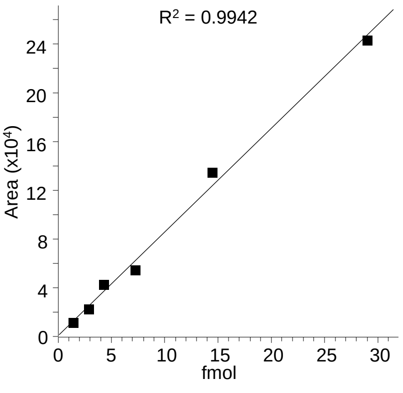

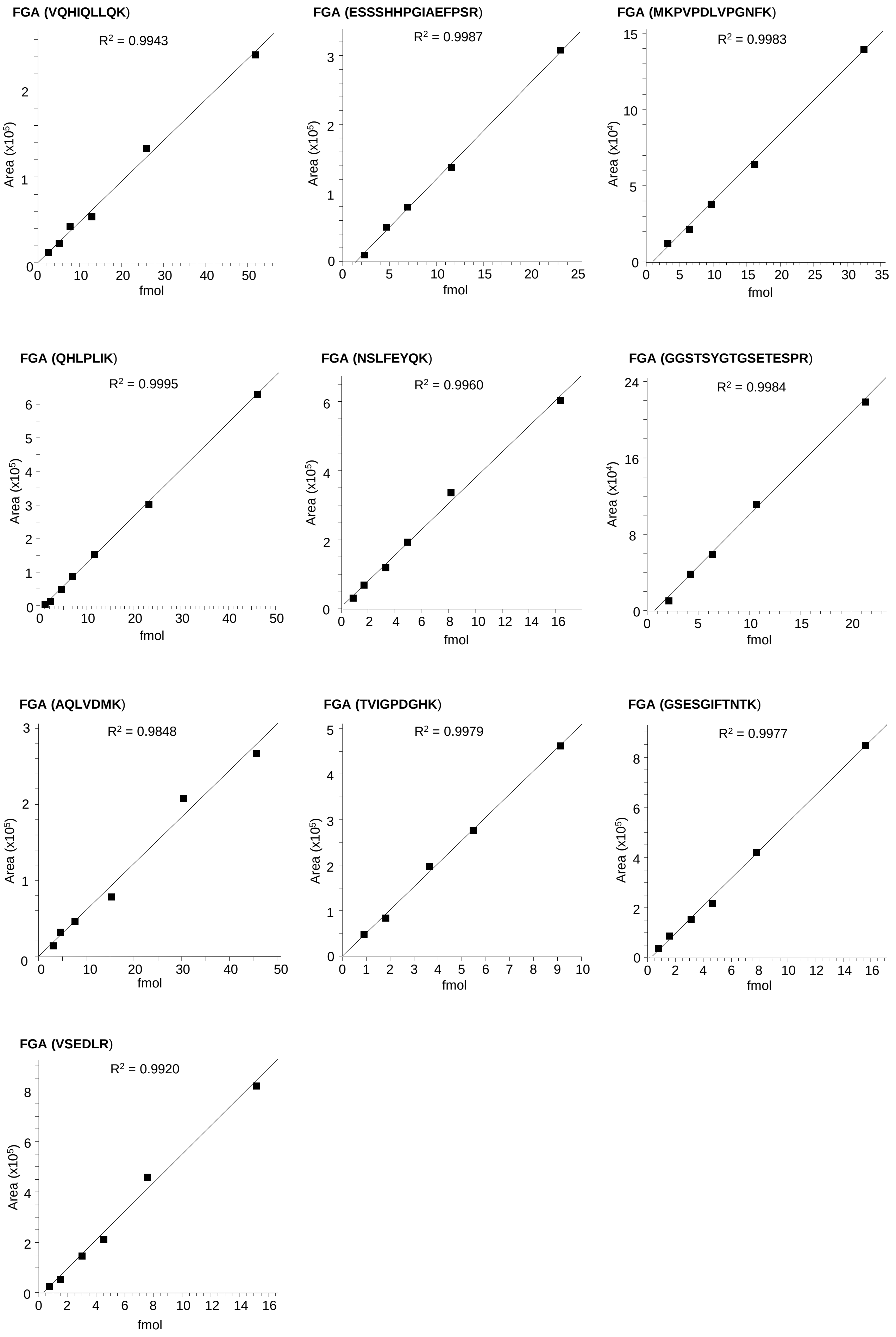
FGA (VQHIQLLQK)
R² = 0.9943
Area (x10⁵)
fmol
FGA (ESSSHHPGIAEFPSR)
R² = 0.9987
Area (x10⁵)
fmol
FGA (MKPVPDLVPGNFK)
R² = 0.9983
Area (x10⁴)
fmol
FGA (QHLPLIK)
R² = 0.9995
Area (x10⁵)
fmol
FGA (NSLFEYQK)
R² = 0.9960
Area (x10⁵)
fmol
FGA (GGSTSYGTGSETESPR)
R² = 0.9984
Area (x10⁴)
fmol
FGA (AQLVDMK)
R² = 0.9848
Area (x10⁵)
fmol
FGA (TVIGPDGHK)
R² = 0.9979
Area (x10⁵)
fmol
FGA (GSESGIFTNTK)
R² = 0.9977
Area (x10⁵)
fmol
FGA (VSEDLR)
R² = 0.9920
Area (x10⁵)
fmol

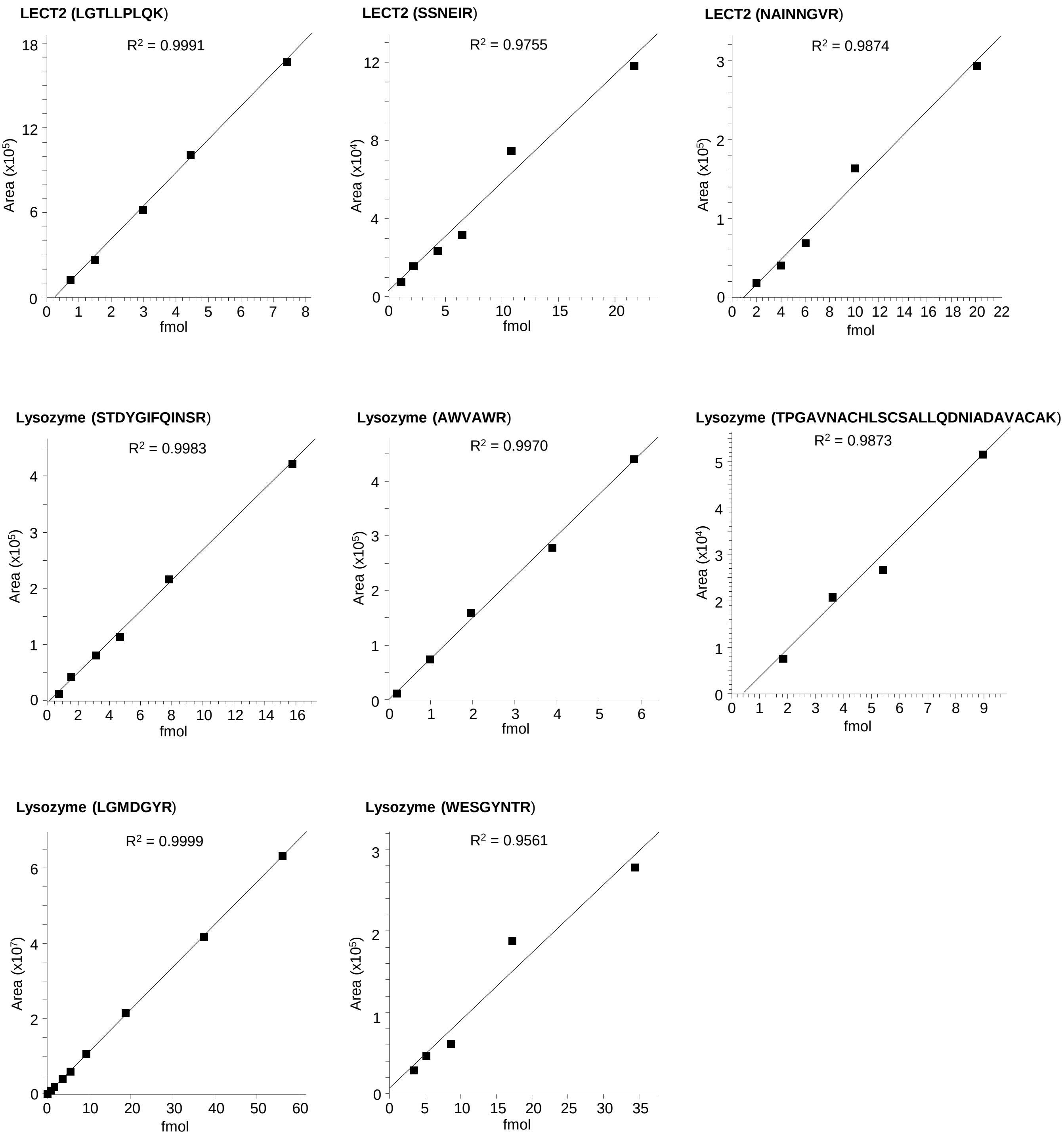
LECT2 (LGTLLPLQK)
R² = 0.9991
Area (x10⁵)
fmol
LECT2 (SSNEIR)
R² = 0.9755
Area (x10⁴)
fmol
LECT2 (NAINNGVR)
R² = 0.9874
Area (x10⁵)
fmol
Lysozyme (STDYGIFQINSR)
R² = 0.9983
Area (x10⁵)
fmol
Lysozyme (AWVAWR)
R² = 0.9970
Area (x10⁵)
fmol
Lysozyme (TPGAVNACHLSCSALLQDNIADAVACAK)
R² = 0.9873
Area (x10⁴)
fmol
Lysozyme (LGMDGYR)
R² = 0.9999
Area (x10⁷)
fmol
Lysozyme (WESGYNTR)
R² = 0.9561
Area (x10⁵)
fmol
