# Supplementary material for: Mass spectrometry-based absolute quantification of amyloid proteins in pathology tissue specimens: Merits and limitations
Source: PLoS One. 2020 Jul 1;15(7):e0235143. doi: 10.1371/journal.pone.0235143 (PMC7329117; doi:10.1371/journal.pone.0235143)

S3 Appendix.
Chromatogram of precursor ions and spectrum of fragmentation ions for a representative peptide of quantified amyloid proteins.

Case ID (e.g. P1) and raw file ID (e.g. A003) correspond to the ID numbers shown in S3 Table.

(A) and (M) serum amyloid A (SAA)
(B) and (N) transthyretin (TTR),
(C) and (O) immunoglobulin kappa light chain (IGK),
(D) and (P) immunoglobulin lambda light chain (IGL),
(E) and (Q) beta-2-microglobulin (B2M),
(F) and (R) apolipoprotein (Apo) A1,
(G) and (S) Apo A2,
(H) and (T) Apo A4,
(I) and (U) Apo E
(J) and (V) gelsolin,
(K) and (W) fibrinogen alpha chain (FGA),
(L) and (X) lysozyme

(A-L) specimens are derived from the heart, and (M-X) from other organs.

A SAA (EANYIGSDK) Heart

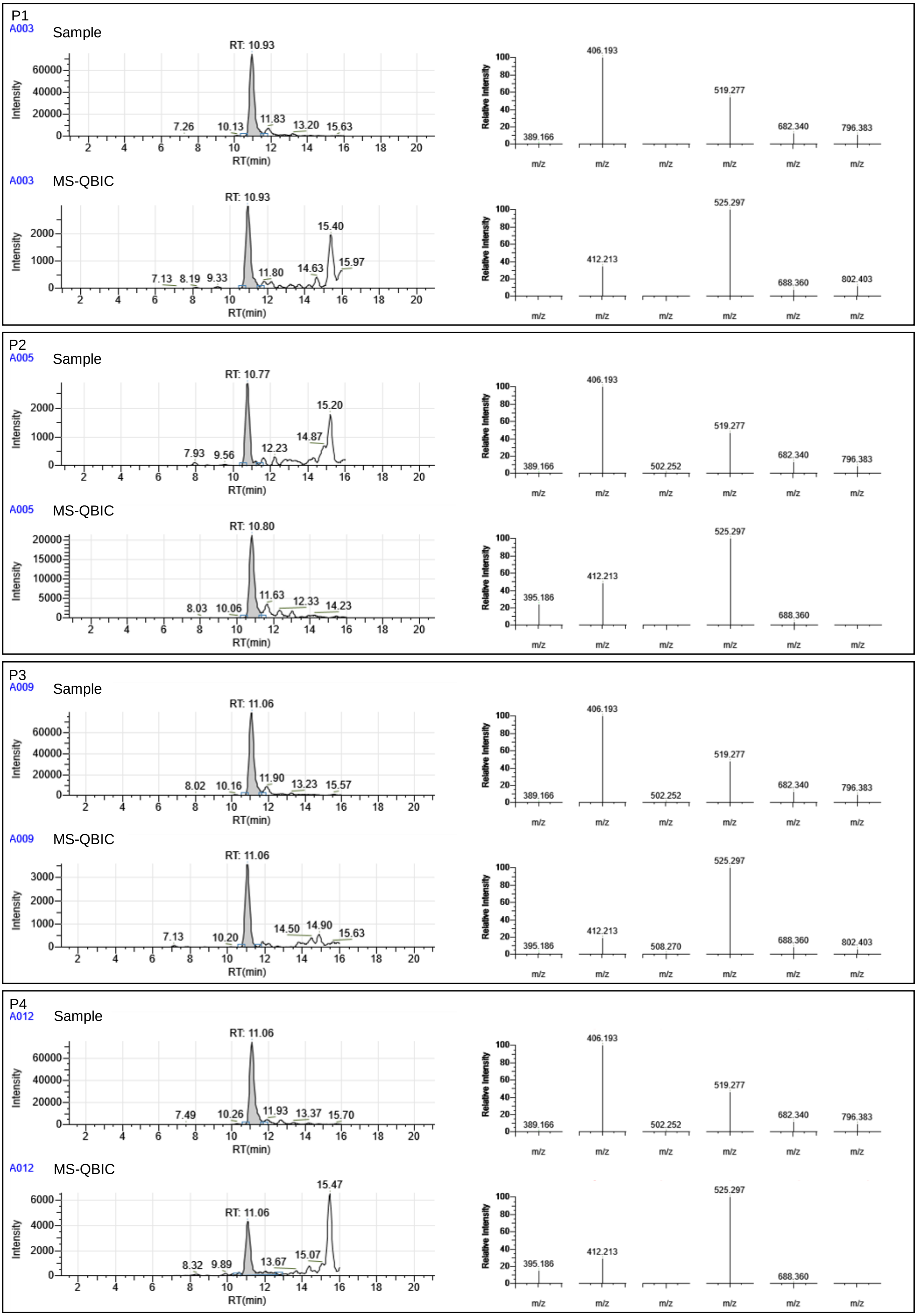

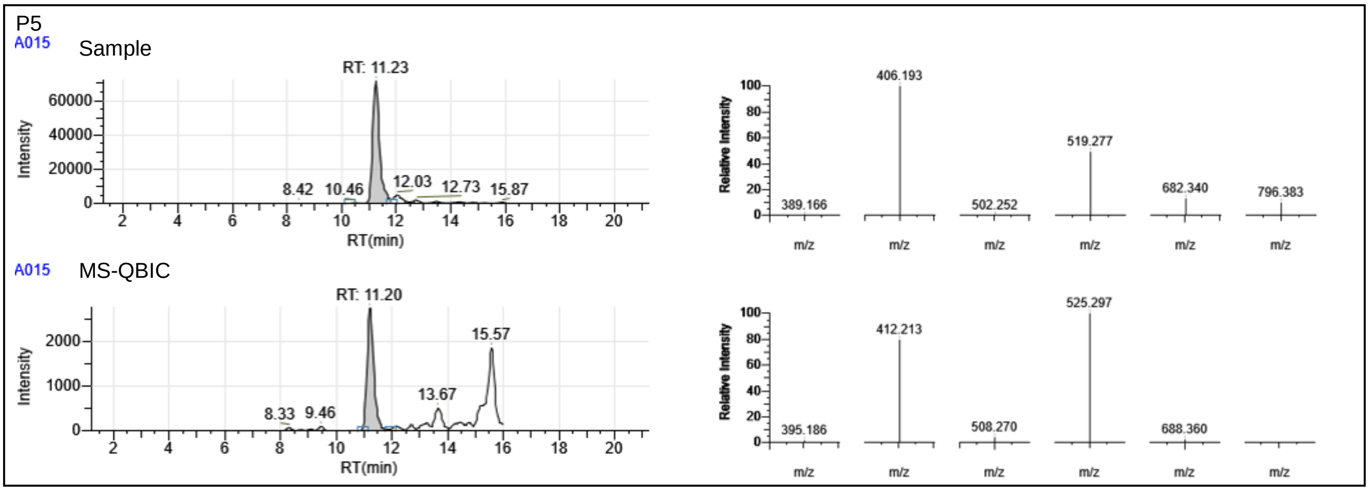
P5
A015 Sample
RT: 11.23
8.42 10.46 12.03 12.73 15.87
389.166 406.193 502.252 519.277 682.340 796.383
A015 MS-QBIC
RT: 11.20
8.33 9.46 13.67 15.57
395.186 412.213 508.270 525.297 688.360
Intensity
RT(min)
Relative Intensity
m/z

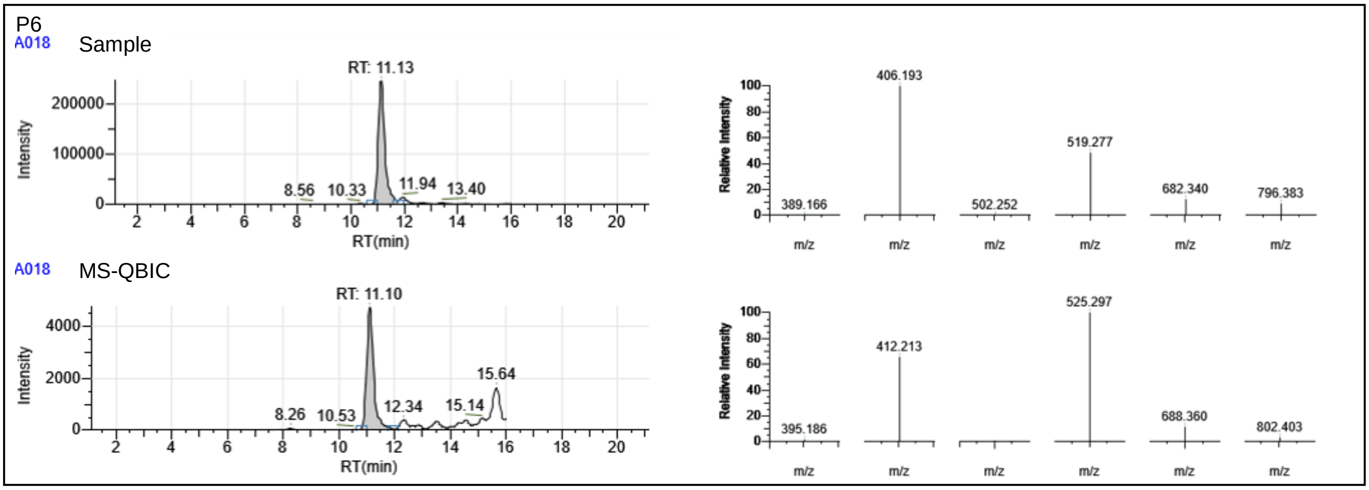
P6
A018 Sample
RT: 11.13
8.56 10.33 11.94 13.40
389.166 406.193 502.252 519.277 682.340 796.383
A018 MS-QBIC
RT: 11.10
8.26 10.53 12.34 15.14 15.64
395.186 412.213 525.297 688.360 802.403
Intensity
RT(min)
Relative Intensity
m/z

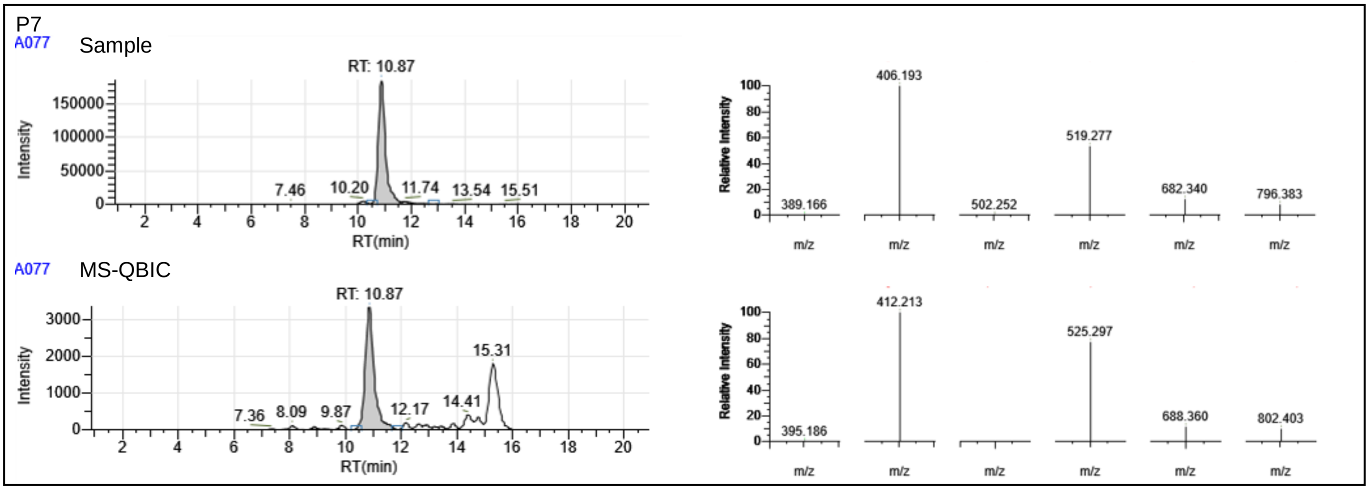
P7
A077 Sample
RT: 10.87
7.46 10.20 11.74 13.54 15.51
389.166 406.193 502.252 519.277 682.340 796.383
A077 MS-QBIC
RT: 10.87
7.36 8.09 9.87 12.17 14.41 15.31
395.186 412.213 525.297 688.360 802.403
Intensity
RT(min)
Relative Intensity
m/z

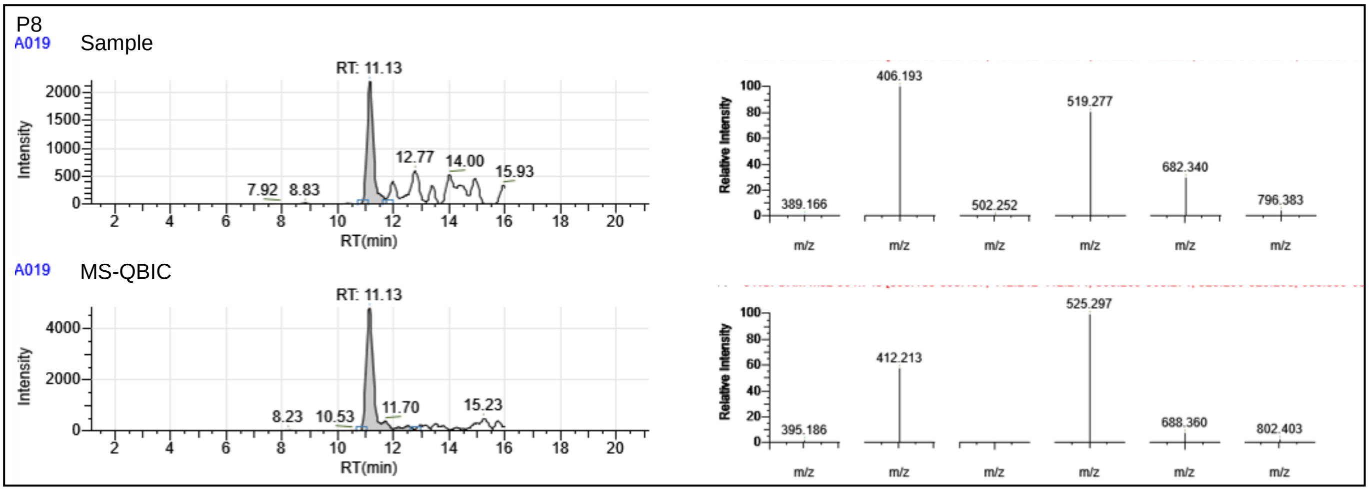
P8
A019 Sample
RT: 11.13
7.92 8.83 12.77 14.00 15.93
389.166 406.193 502.252 519.277 682.340 796.383
A019 MS-QBIC
RT: 11.13
8.23 10.53 11.70 15.23
395.186 412.213 525.297 688.360 802.403
Intensity
RT(min)
Relative Intensity
m/z

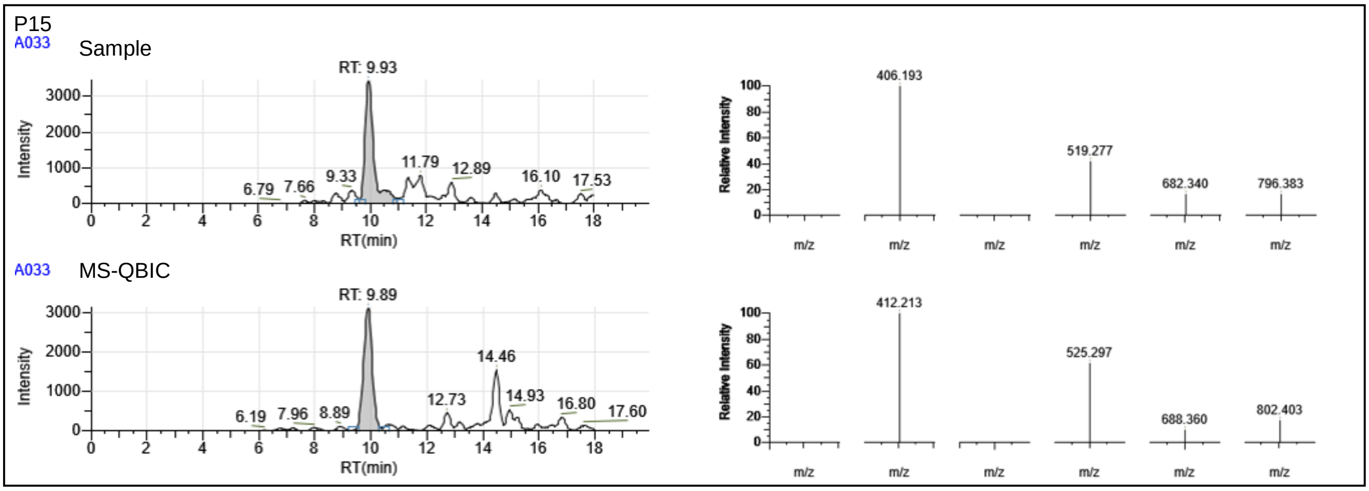

P15
A033 Sample
RT: 9.93
6.79 7.66 9.33 11.79 12.89 16.10 17.53
Intensity
RT(min)
406.193
519.277
682.340
796.383
Relative Intensity
m/z
A033 MS-QBIC
RT: 9.89
6.19 7.96 8.89 12.73 14.46 14.93 16.80 17.60
412.213
525.297
688.360
802.403

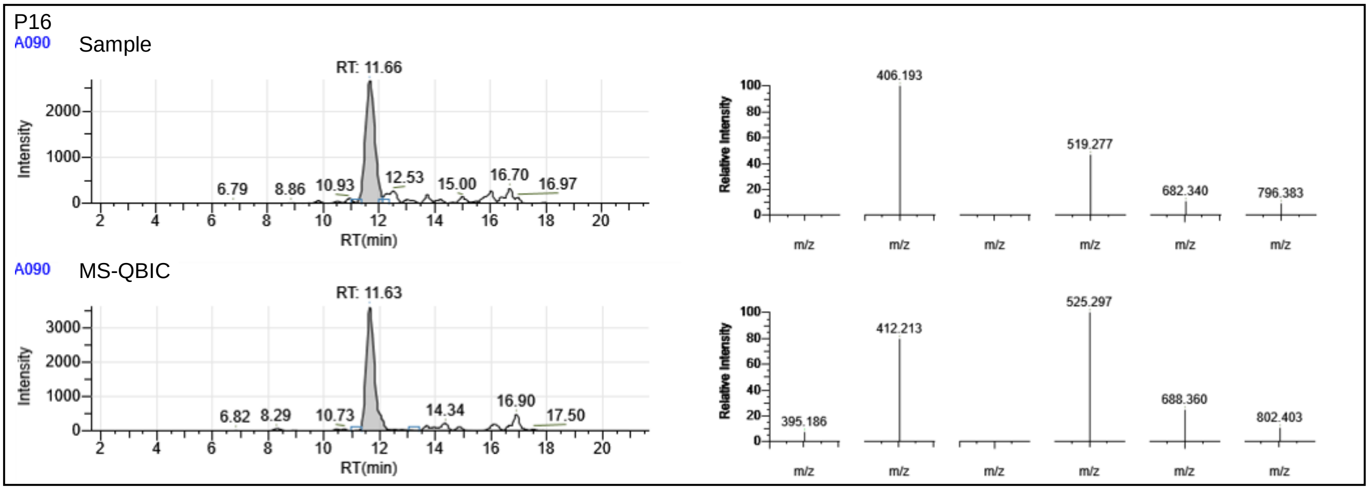

P16
A090 Sample
RT: 11.66
6.79 8.86 10.93 12.53 15.00 16.70 16.97
Intensity
RT(min)
406.193
519.277
682.340
796.383
Relative Intensity
m/z
A090 MS-QBIC
RT: 11.63
6.82 8.29 10.73 14.34 16.90 17.50
395.186
412.213
525.297
688.360
802.403

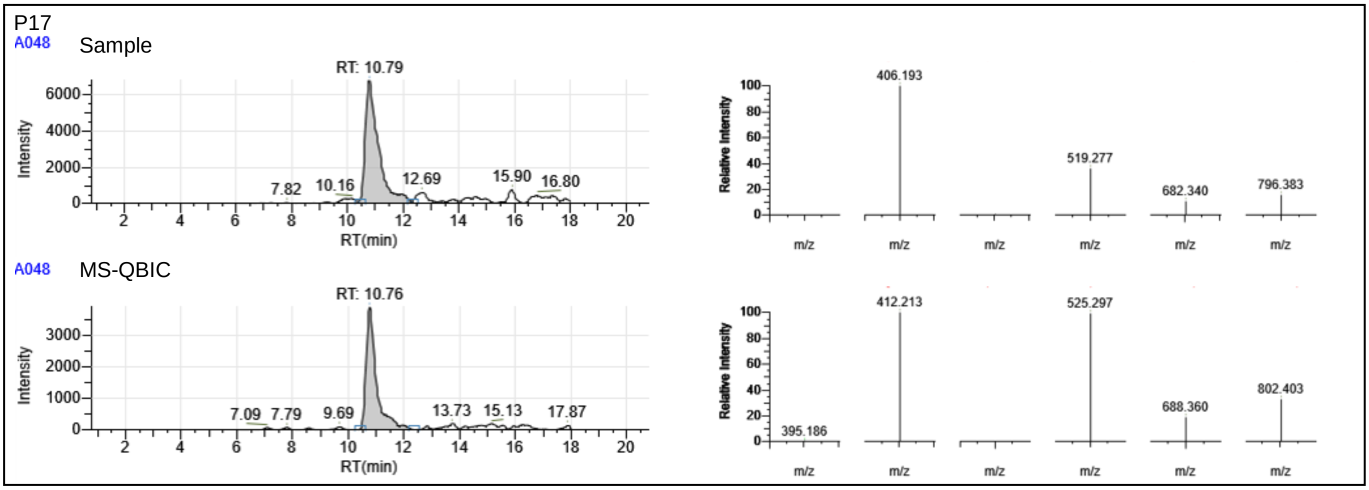

P17
A048 Sample
RT: 10.79
7.82 10.16 12.69 15.90 16.80
Intensity
RT(min)
406.193
519.277
682.340
796.383
Relative Intensity
m/z
A048 MS-QBIC
RT: 10.76
7.09 7.79 9.69 13.73 15.13 17.87
395.186
412.213
525.297
688.360
802.403

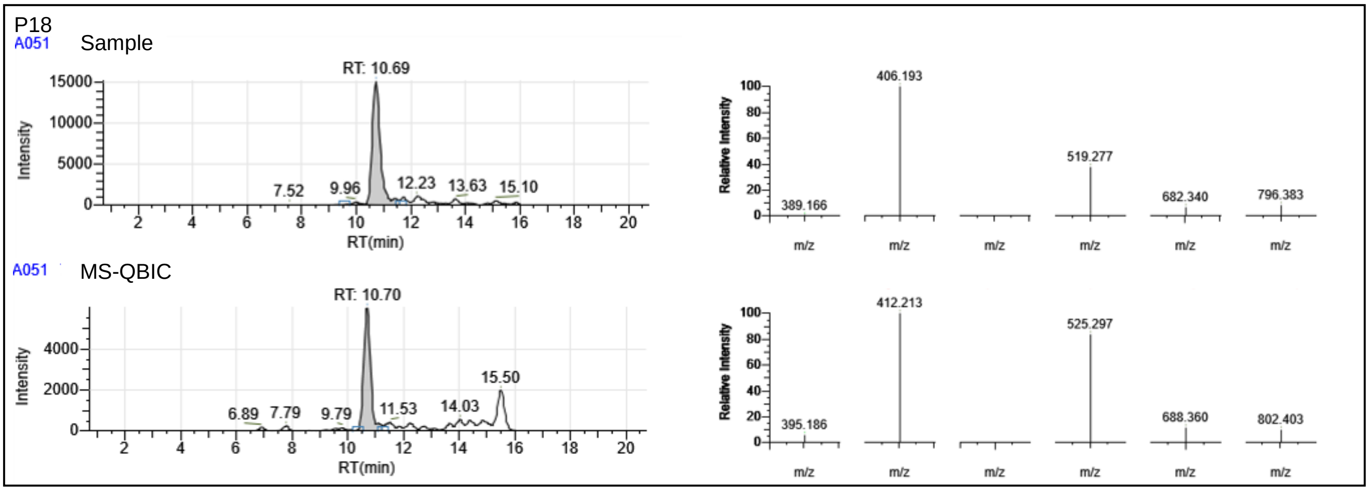

P18
A051 Sample
RT: 10.69
7.52 9.96 12.23 13.63 15.10
Intensity
RT(min)
389.166
406.193
519.277
682.340
796.383
Relative Intensity
m/z
A051 MS-QBIC
RT: 10.70
6.89 7.79 9.79 11.53 14.03 15.50
395.186
412.213
525.297
688.360
802.403

P19

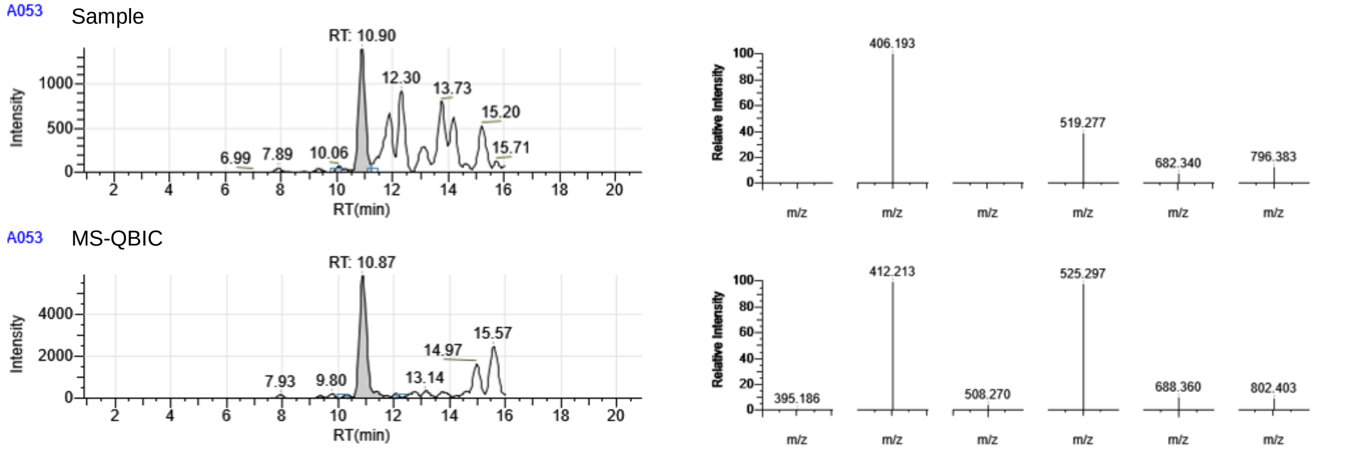

P21

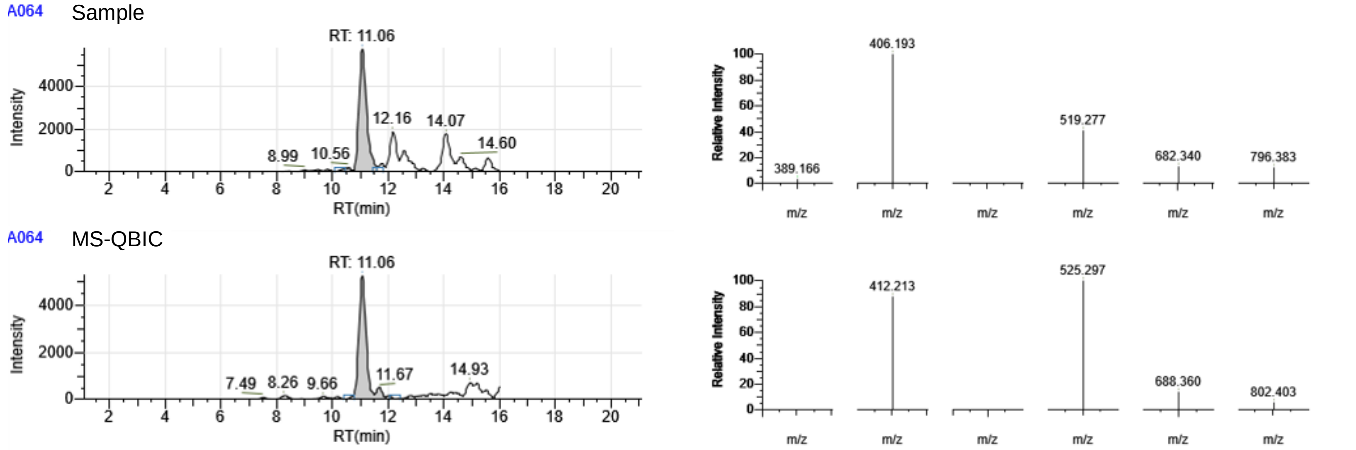

P23

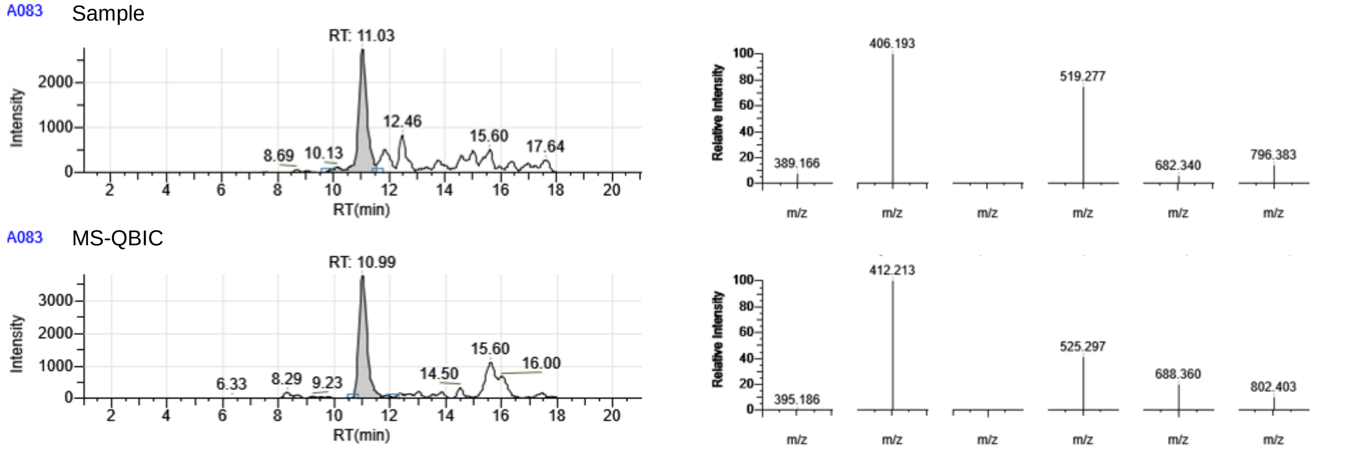

P30

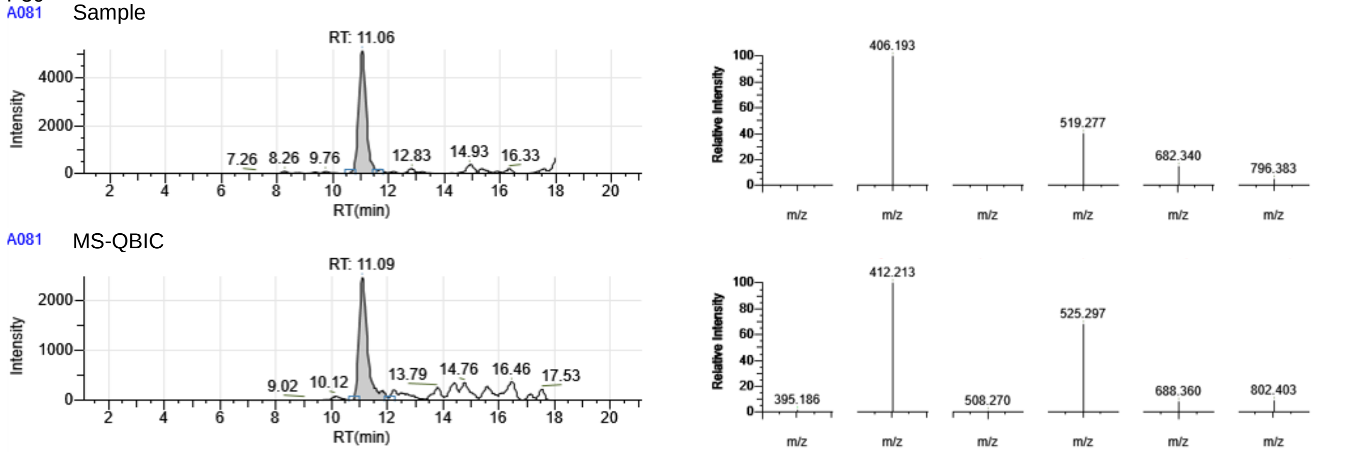

B TTR (AADDTWEPFASGK) Heart

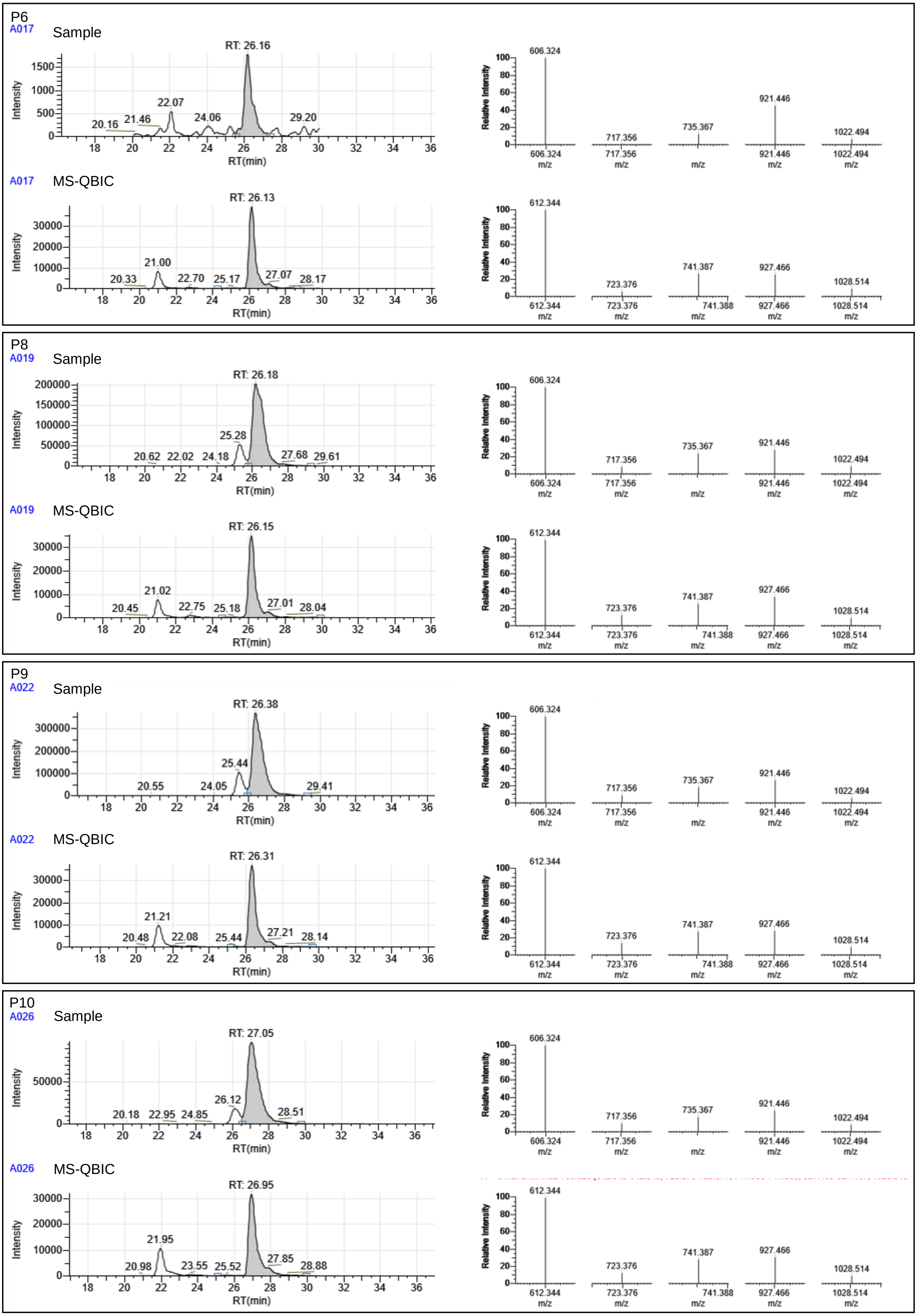

P11

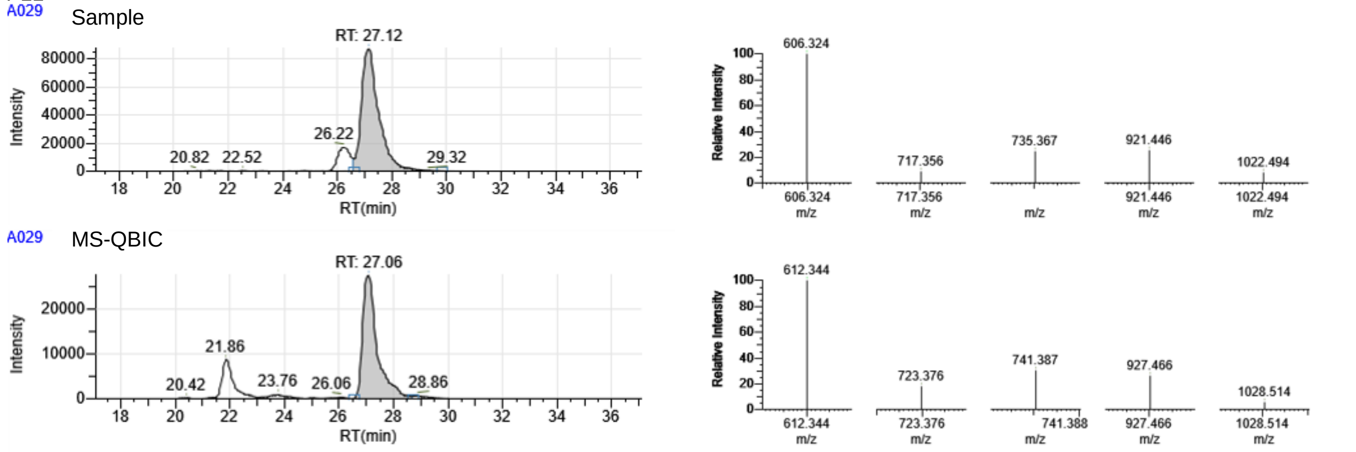

P12

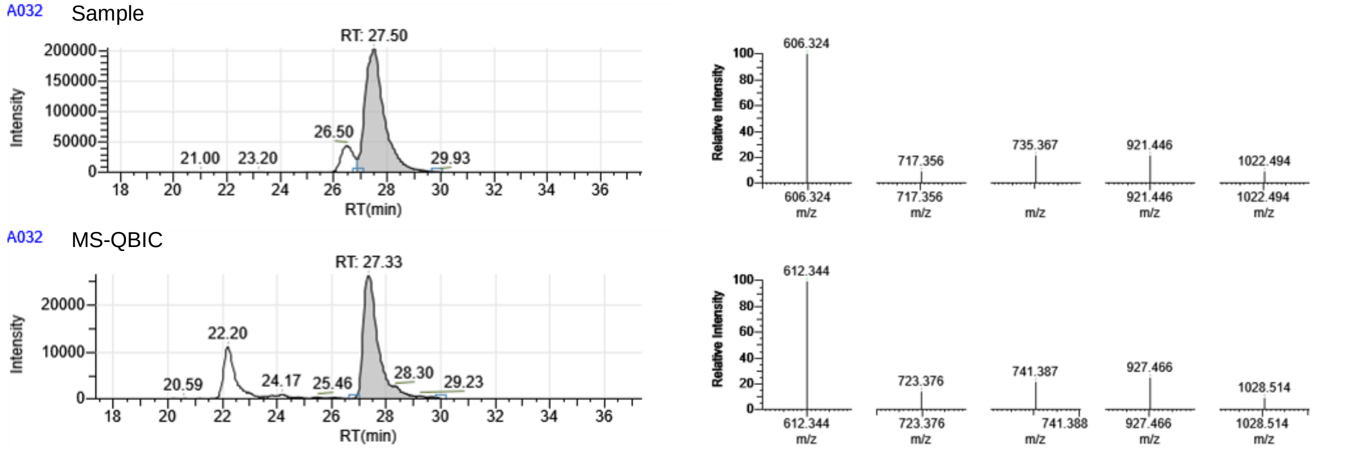

P13

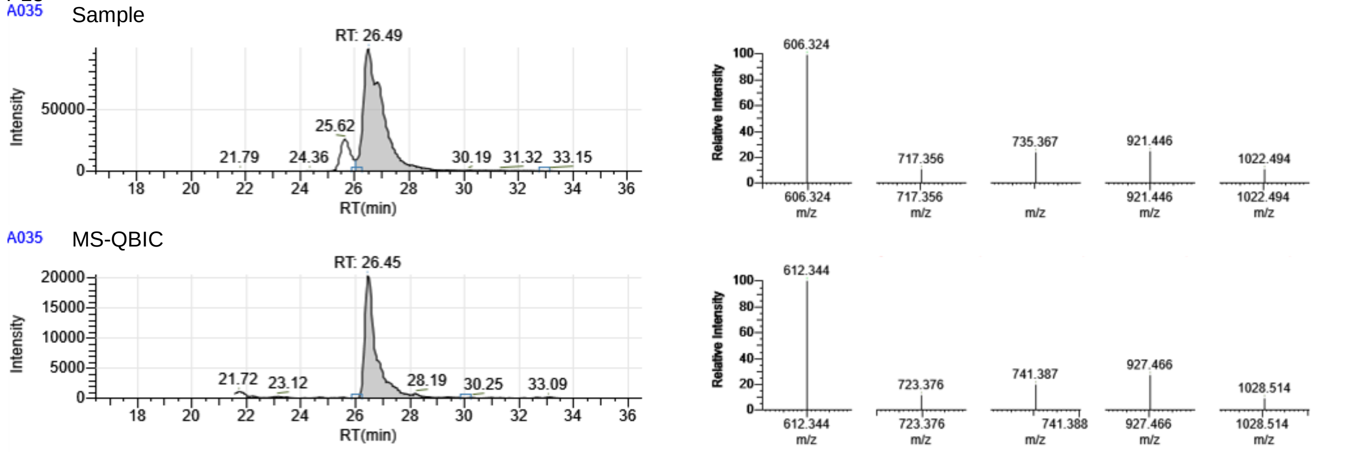

P14

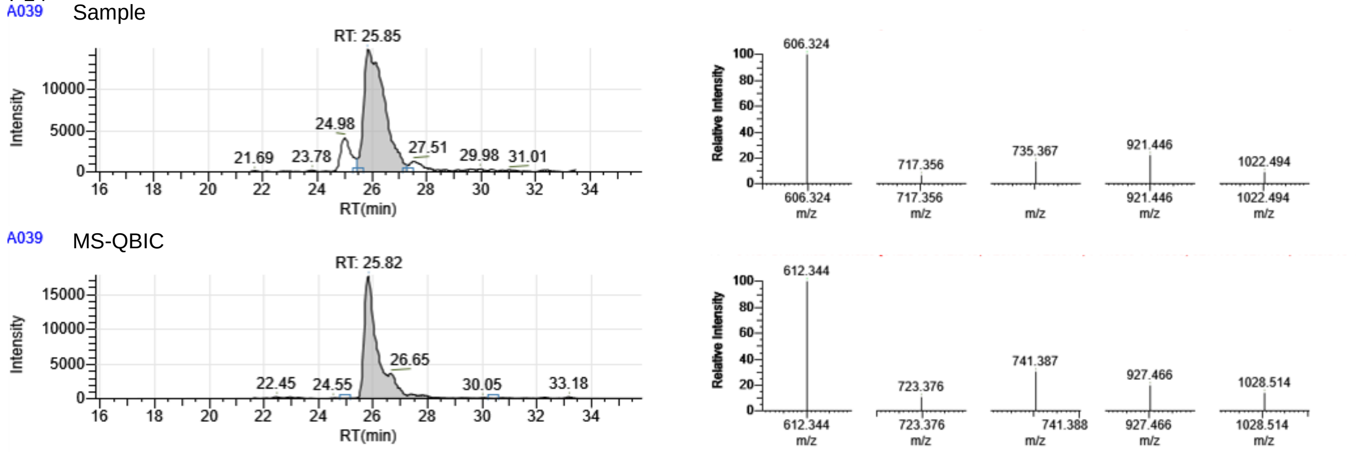

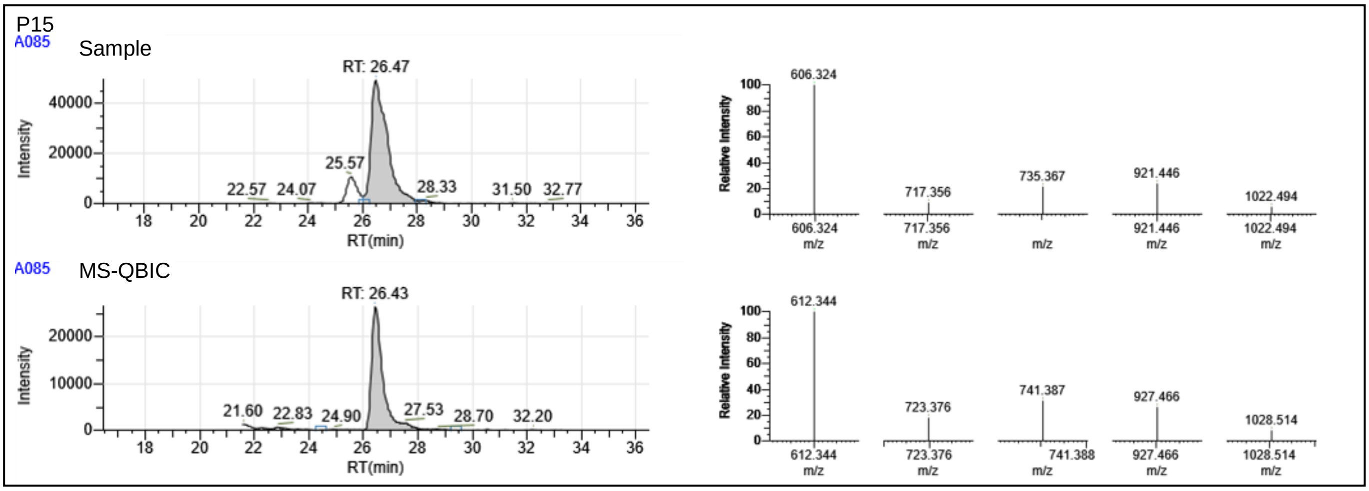
P15
A085 Sample
RT: 26.47
22.57 24.07 25.57 28.33 31.50 32.77
Intensity
RT(min)
606.324 717.356 735.367 921.446 1022.494
Relative Intensity
m/z
A085 MS-QBIC
RT: 26.43
21.60 22.83 24.90 27.53 28.70 32.20
Intensity
RT(min)
612.344 723.376 741.387 741.388 927.466 1028.514
Relative Intensity
m/z

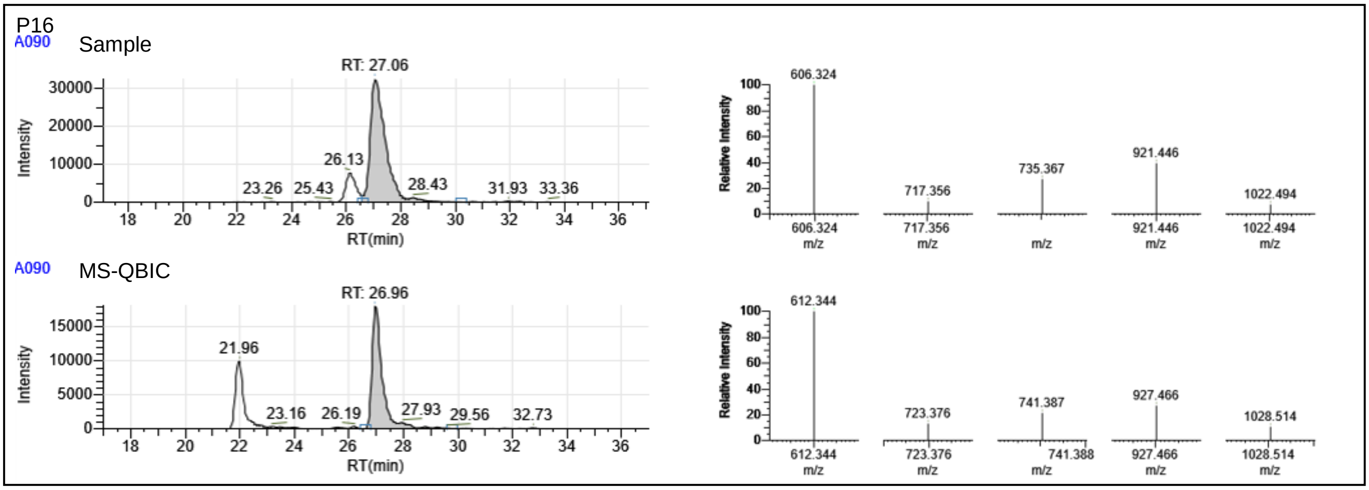
P16
A090 Sample
RT: 27.06
23.26 25.43 26.13 28.43 31.93 33.36
Intensity
RT(min)
606.324 717.356 735.367 921.446 1022.494
Relative Intensity
m/z
A090 MS-QBIC
RT: 26.96
21.96 23.16 26.19 27.93 29.56 32.73
Intensity
RT(min)
612.344 723.376 741.387 741.388 927.466 1028.514
Relative Intensity
m/z

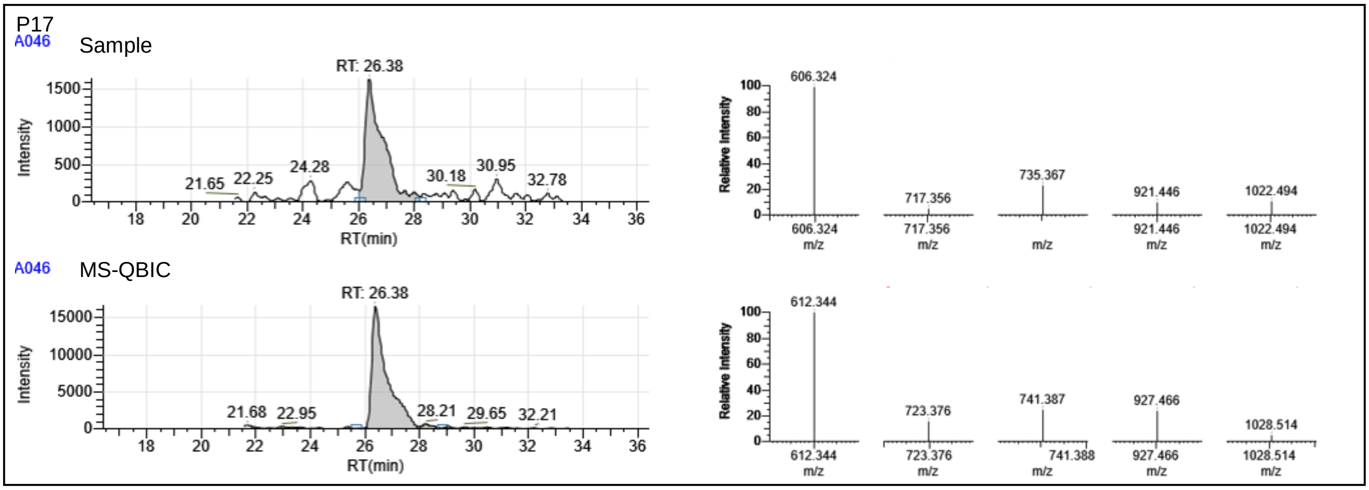
P17
A046 Sample
RT: 26.38
21.65 22.25 24.28 30.18 30.95 32.78
Intensity
RT(min)
606.324 717.356 735.367 921.446 1022.494
Relative Intensity
m/z
A046 MS-QBIC
RT: 26.38
21.68 22.95 28.21 29.65 32.21
Intensity
RT(min)
612.344 723.376 741.387 741.388 927.466 1028.514
Relative Intensity
m/z

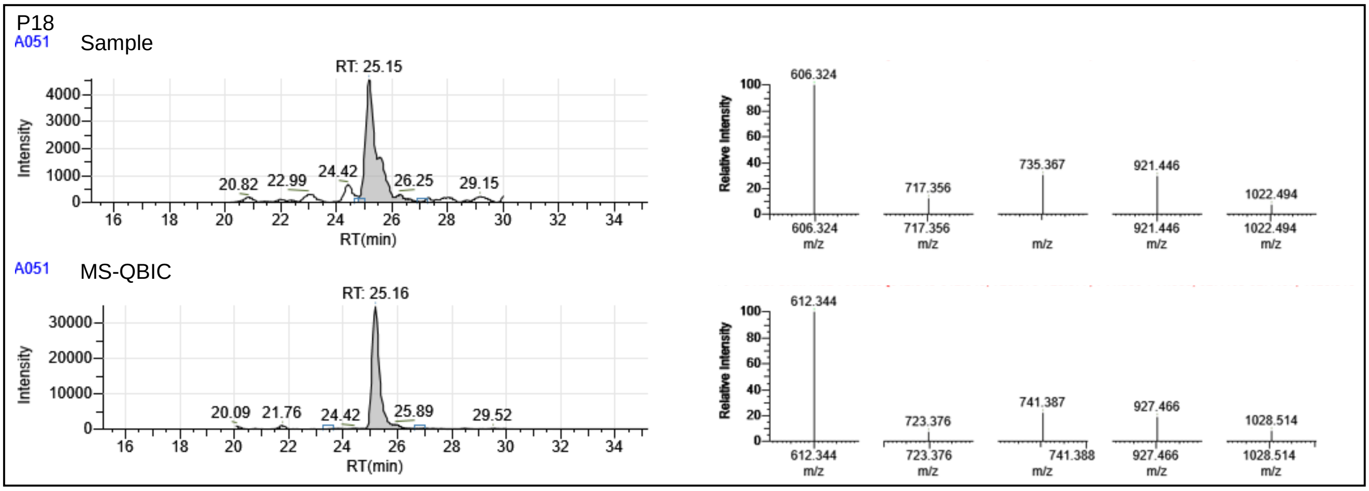
P18
A051 Sample
RT: 25.15
20.82 22.99 24.42 26.25 29.15
Intensity
RT(min)
606.324 717.356 735.367 921.446 1022.494
Relative Intensity
m/z
A051 MS-QBIC
RT: 25.16
20.09 21.76 24.42 25.89 29.52
Intensity
RT(min)
612.344 723.376 741.387 741.388 927.466 1028.514
Relative Intensity
m/z

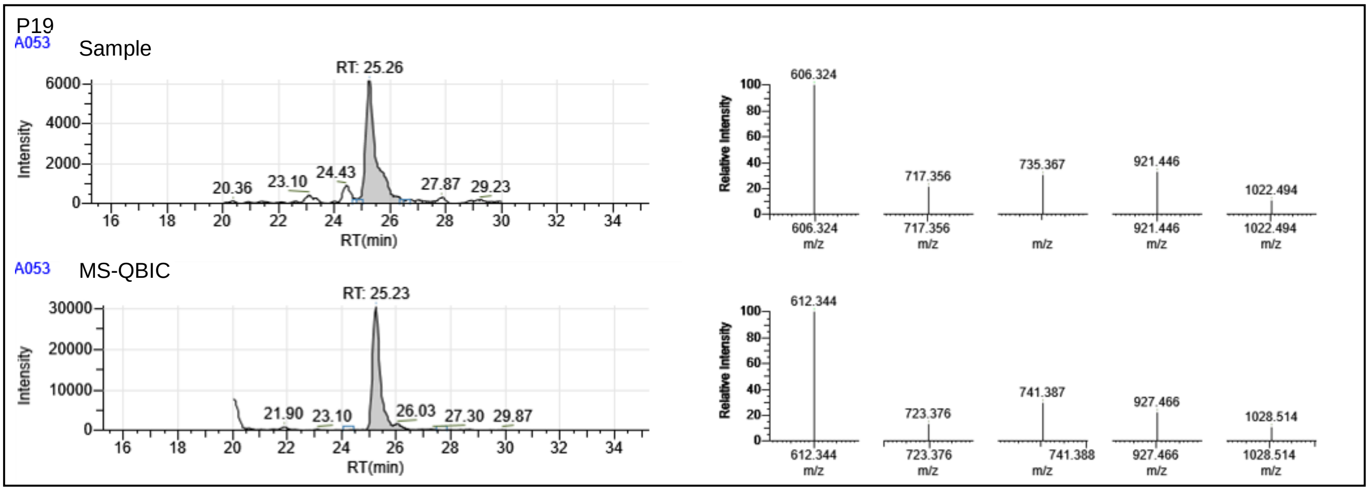
P19
A053 Sample
RT: 25.26
Intensity
RT(min)
20.36 23.10 24.43 27.87 29.23
Relative Intensity
606.324 717.356 735.367 921.446 1022.494
m/z
A053 MS-QBIC
RT: 25.23
21.90 23.10 26.03 27.30 29.87
612.344 723.376 741.387 741.388 927.466 1028.514

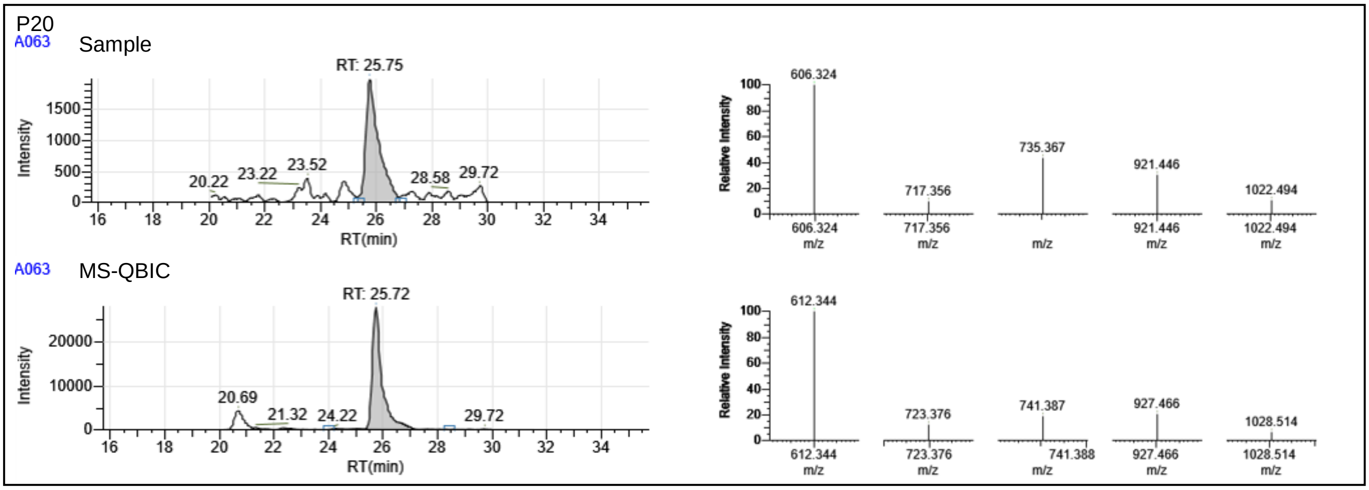
P20
A063 Sample
RT: 25.75
Intensity
RT(min)
20.22 23.22 23.52 28.58 29.72
Relative Intensity
606.324 717.356 735.367 921.446 1022.494
m/z
A063 MS-QBIC
RT: 25.72
20.69 21.32 24.22 29.72
612.344 723.376 741.387 741.388 927.466 1028.514

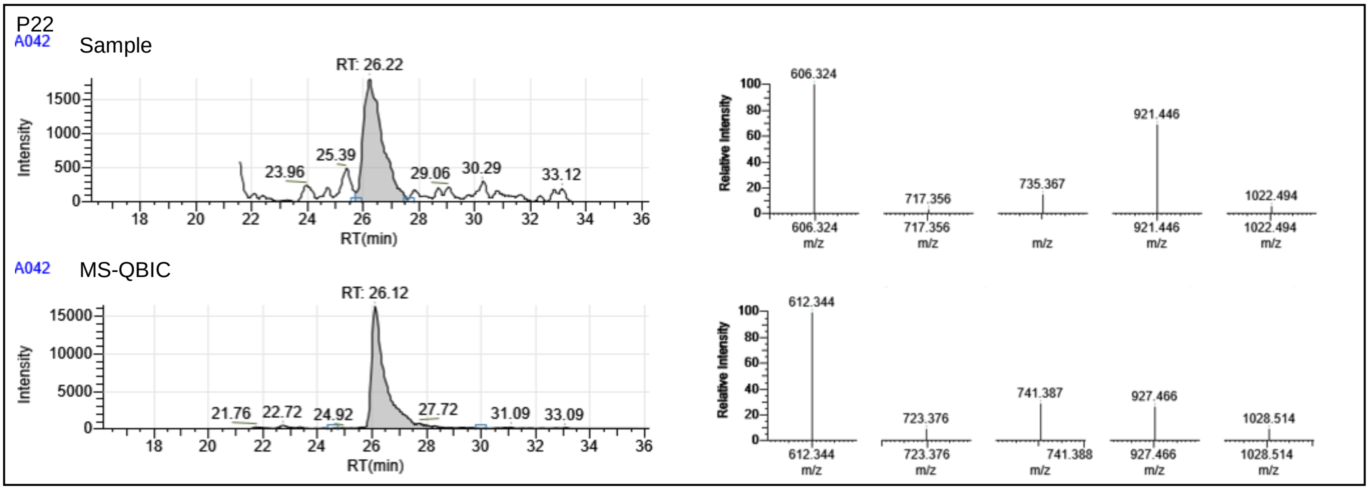
P22
A042 Sample
RT: 26.22
Intensity
RT(min)
23.96 25.39 29.06 30.29 33.12
Relative Intensity
606.324 717.356 735.367 921.446 1022.494
m/z
A042 MS-QBIC
RT: 26.12
21.76 22.72 24.92 27.72 31.09 33.09
612.344 723.376 741.387 741.388 927.466 1028.514

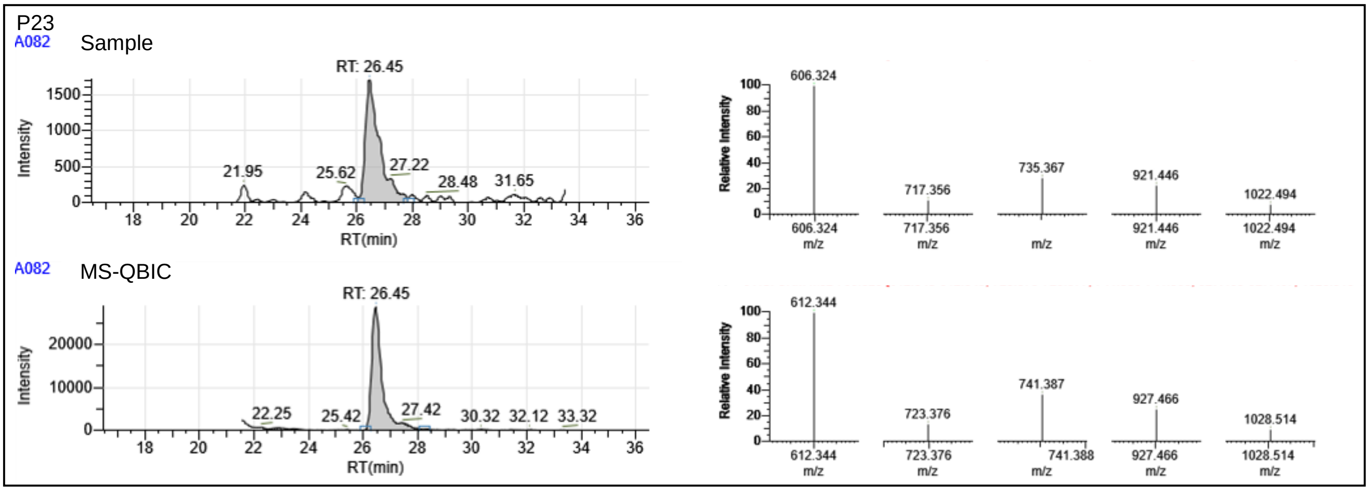
P23
A082 Sample
RT: 26.45
Intensity
RT(min)
21.95 25.62 27.22 28.48 31.65
Relative Intensity
606.324 717.356 735.367 921.446 1022.494
m/z
A082 MS-QBIC
RT: 26.45
22.25 25.42 27.42 30.32 32.12 33.32
612.344 723.376 741.387 741.388 927.466 1028.514

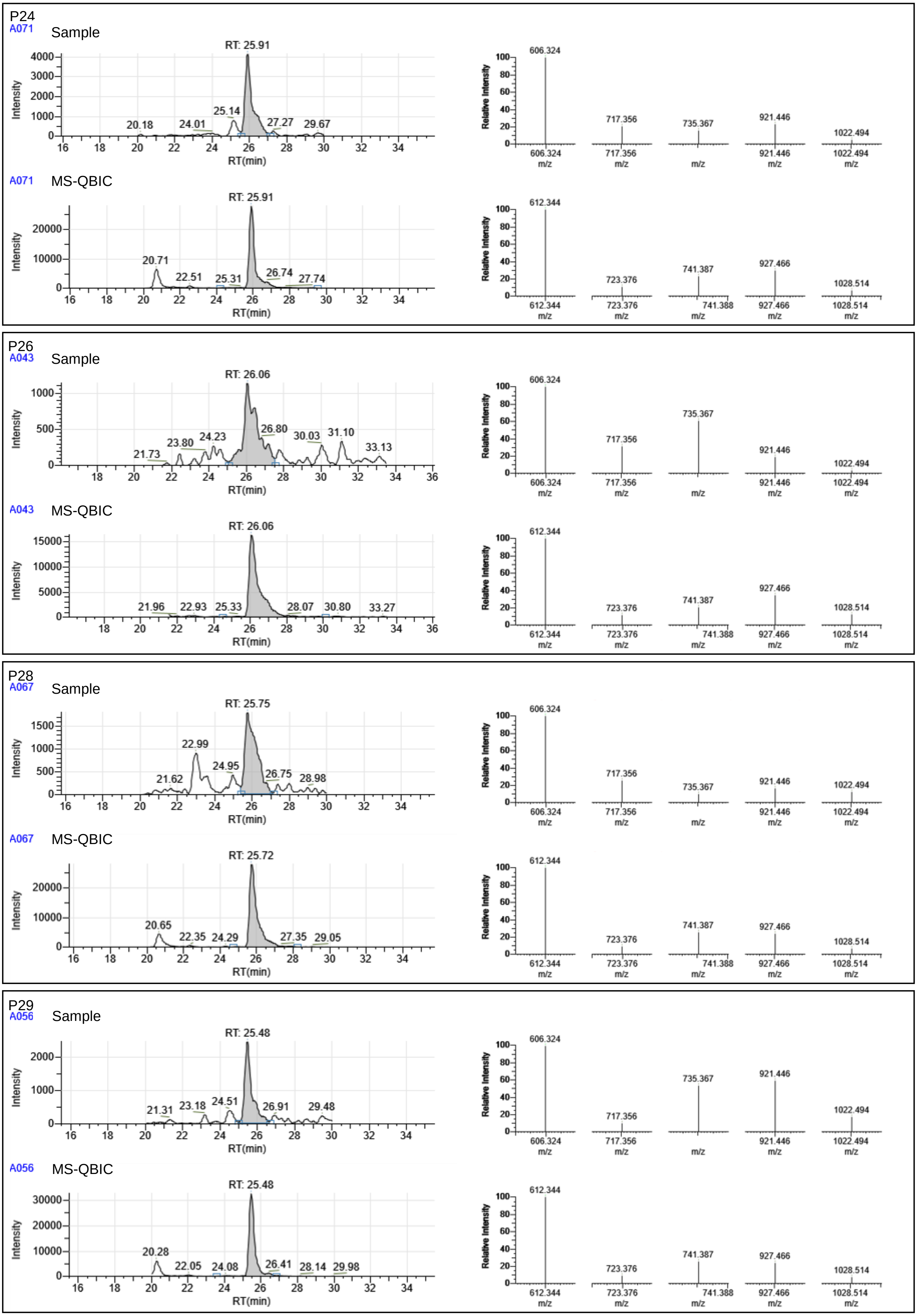
P24
A071 Sample
RT: 25.91
20.18
24.01
25.14
27.27
29.67
RT(min)
Intensity
606.324
717.356
735.367
921.446
1022.494
Relative Intensity
m/z
A071 MS-QBIC
RT: 25.91
20.71
22.51
25.31
26.74
27.74
612.344
723.376
741.387
741.388
927.466
1028.514
P26
A043 Sample
RT: 26.06
21.73
23.80
24.23
26.80
30.03
31.10
33.13
A043 MS-QBIC
RT: 26.06
21.96
22.93
25.33
28.07
30.80
33.27
P28
A067 Sample
RT: 25.75
21.62
22.99
24.95
26.75
28.98
A067 MS-QBIC
RT: 25.72
20.65
22.35
24.29
27.35
29.05
P29
A056 Sample
RT: 25.48
21.31
23.18
24.51
26.91
29.48
A056 MS-QBIC
RT: 25.48
20.28
22.05
24.08
26.41
28.14
29.98

C IGK (TVAAPSVFIFPPSDEQLK) Heart

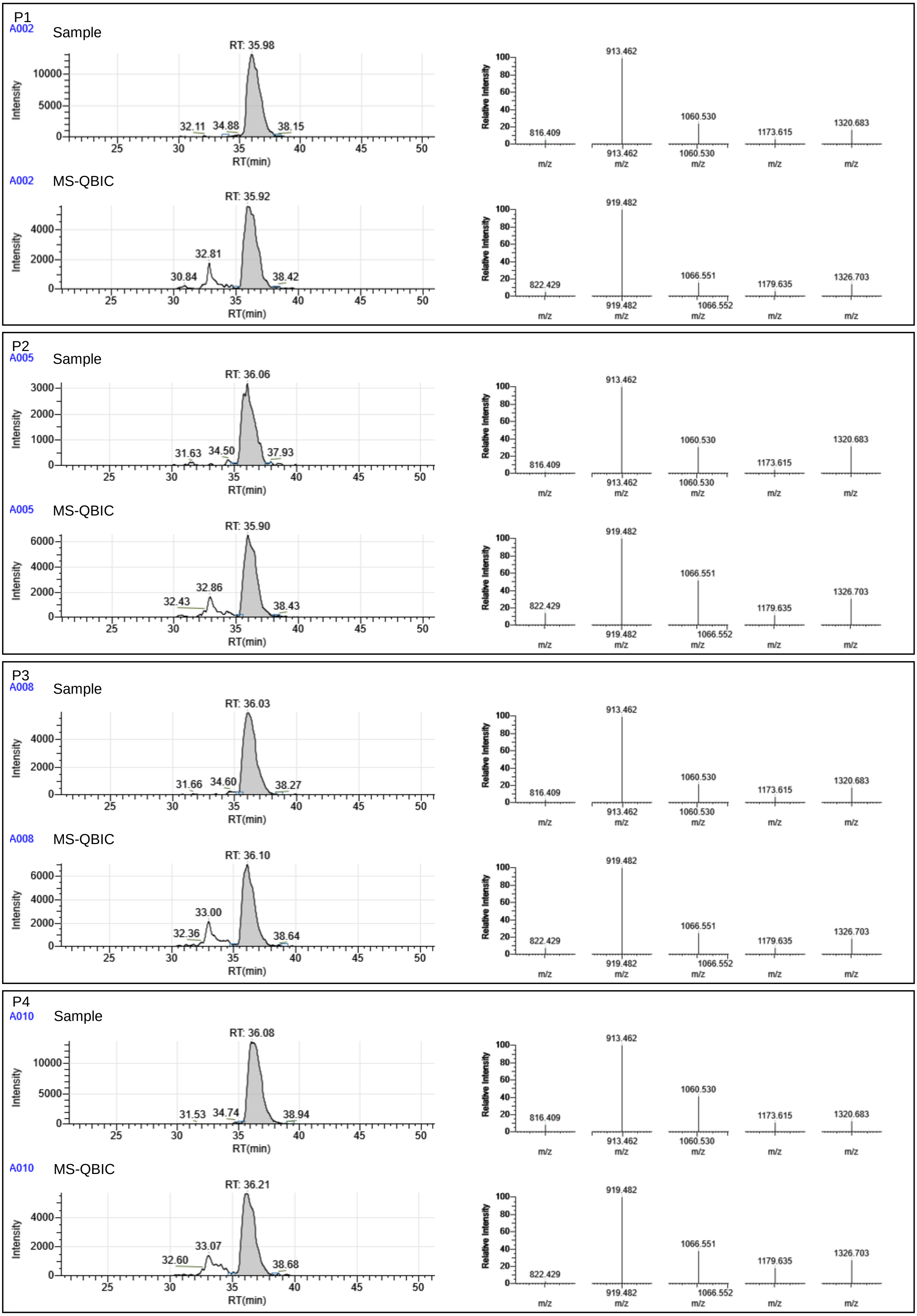

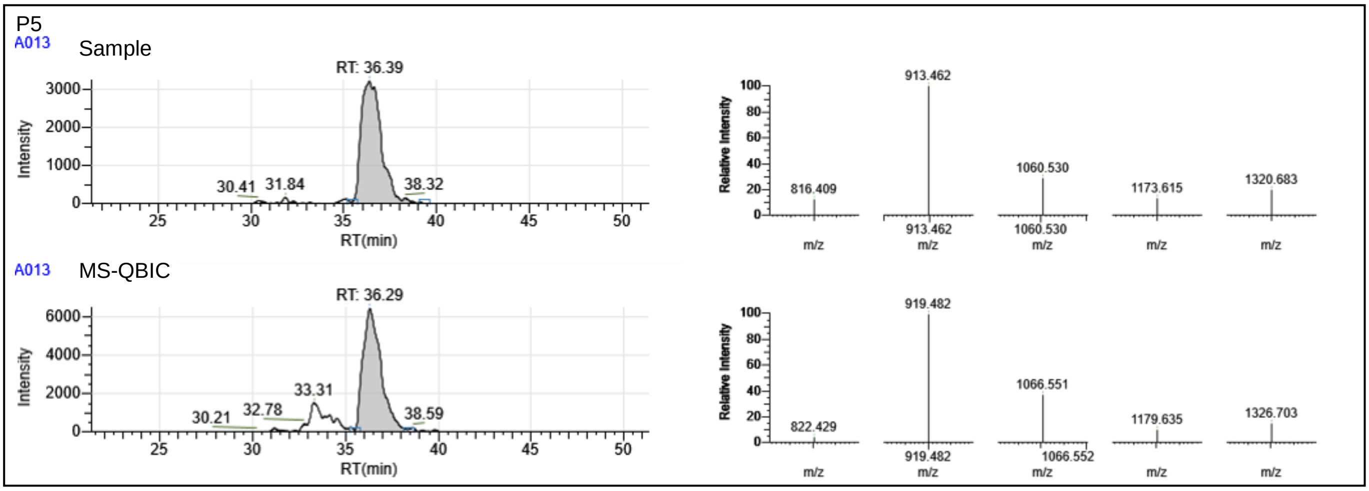
P5
A013 Sample
RT: 36.39
30.41 31.84 38.32
Intensity
RT(min)
816.409
913.462
1060.530
1173.615
1320.683
Relative Intensity
m/z
A013 MS-QBIC
RT: 36.29
30.21 32.78 33.31 38.59
822.429
919.482
1066.551
1066.552
1179.635
1326.703

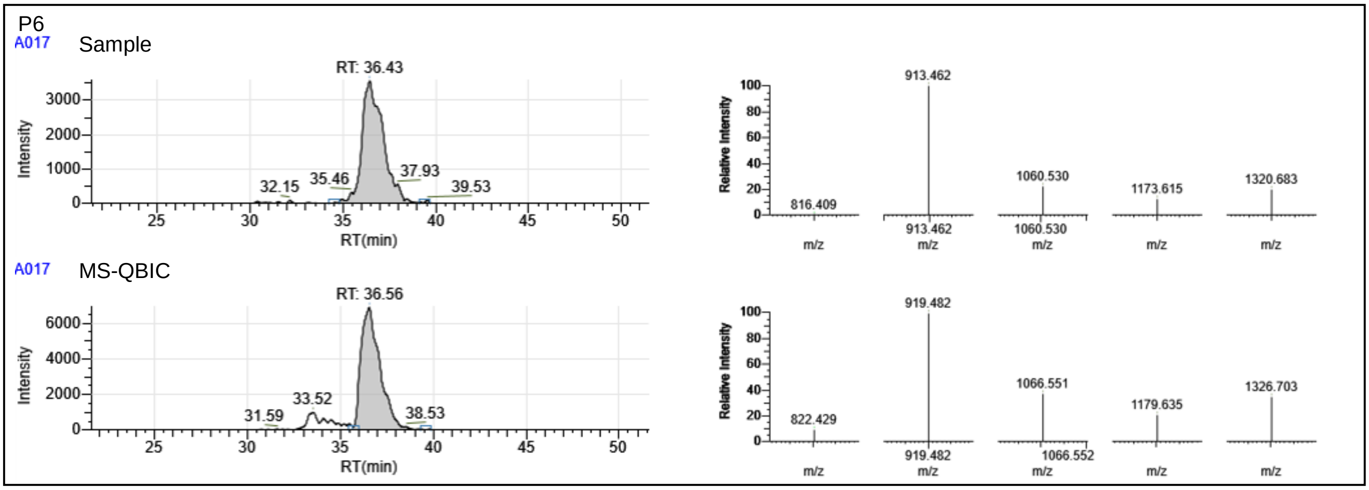
P6
A017 Sample
RT: 36.43
32.15 35.46 37.93 39.53
Intensity
RT(min)
816.409
913.462
1060.530
1173.615
1320.683
Relative Intensity
m/z
A017 MS-QBIC
RT: 36.56
31.59 33.52 38.53
822.429
919.482
1066.551
1066.552
1179.635
1326.703

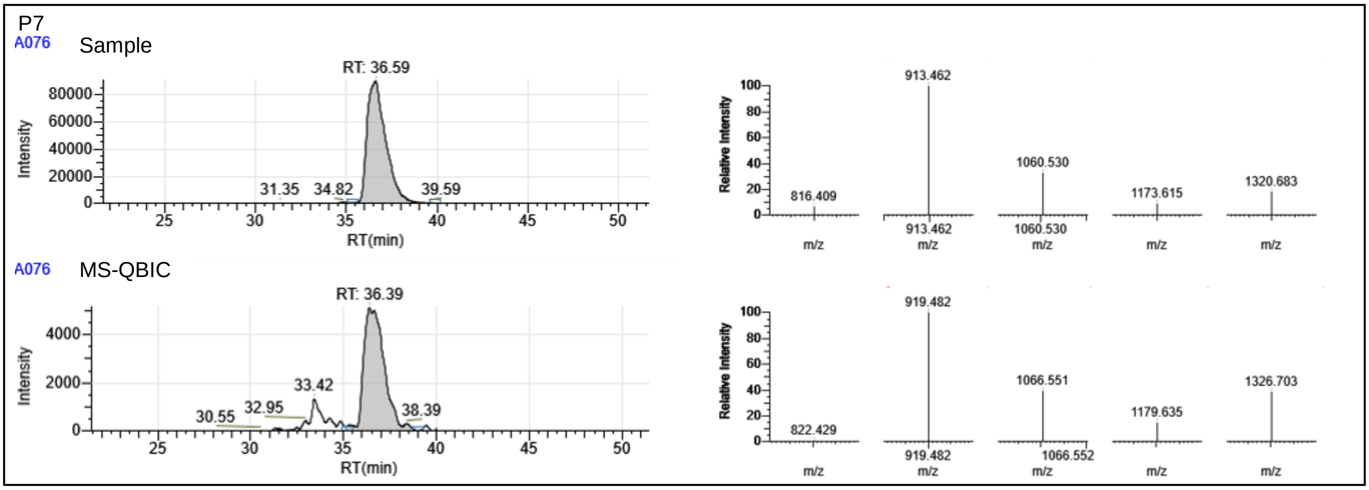
P7
A076 Sample
RT: 36.59
31.35 34.82 39.59
Intensity
RT(min)
816.409
913.462
1060.530
1173.615
1320.683
Relative Intensity
m/z
A076 MS-QBIC
RT: 36.39
30.55 32.95 33.42 38.39
822.429
919.482
1066.551
1066.552
1179.635
1326.703

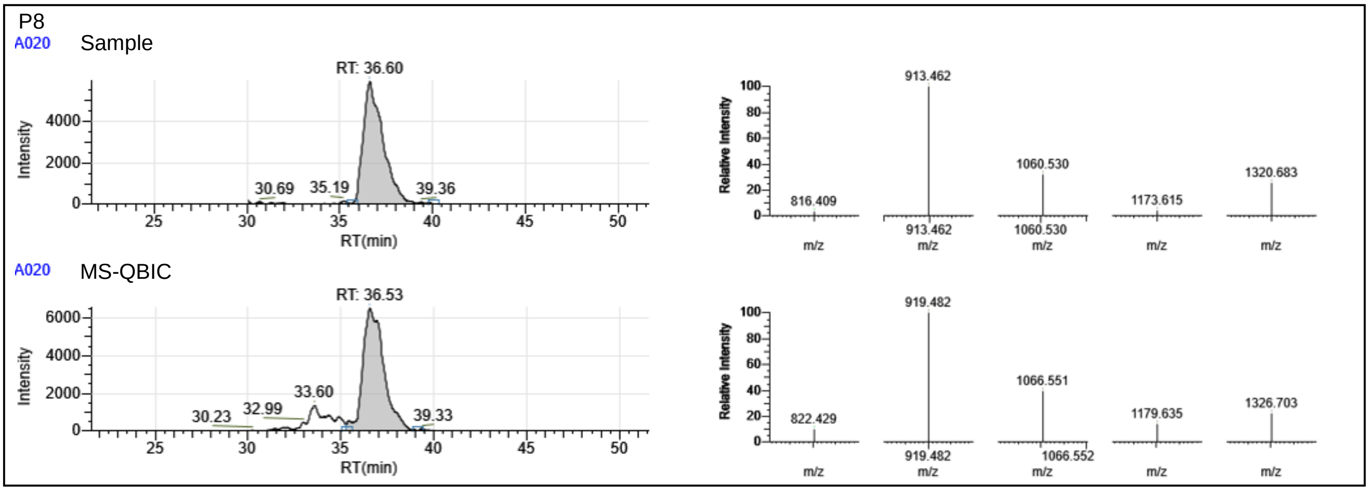
P8
A020 Sample
RT: 36.60
30.69 35.19 39.36
Intensity
RT(min)
816.409
913.462
1060.530
1173.615
1320.683
Relative Intensity
m/z
A020 MS-QBIC
RT: 36.53
30.23 32.99 33.60 39.33
822.429
919.482
1066.551
1066.552
1179.635
1326.703

P9

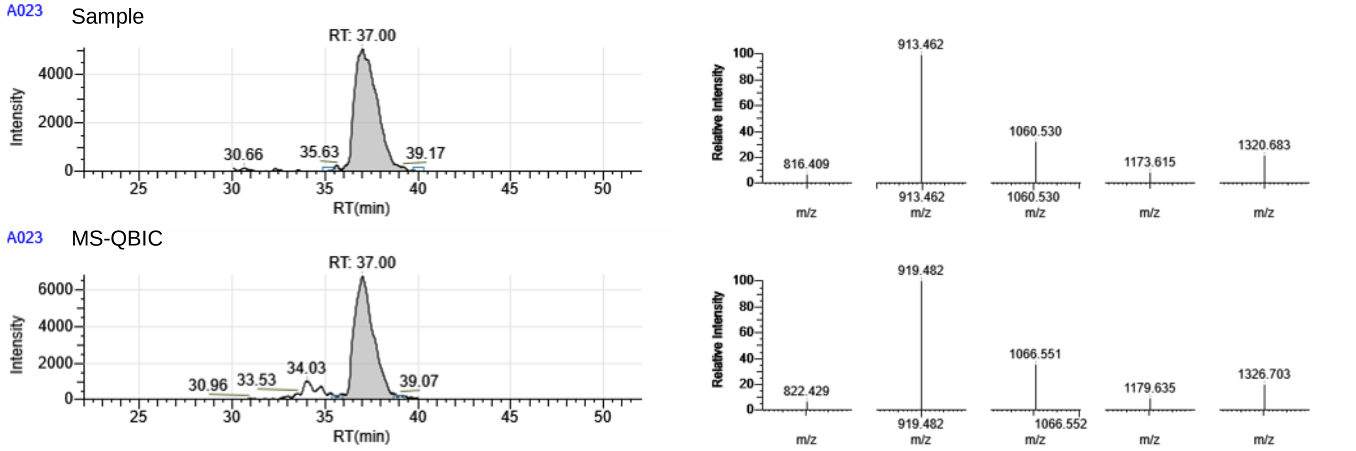

P10

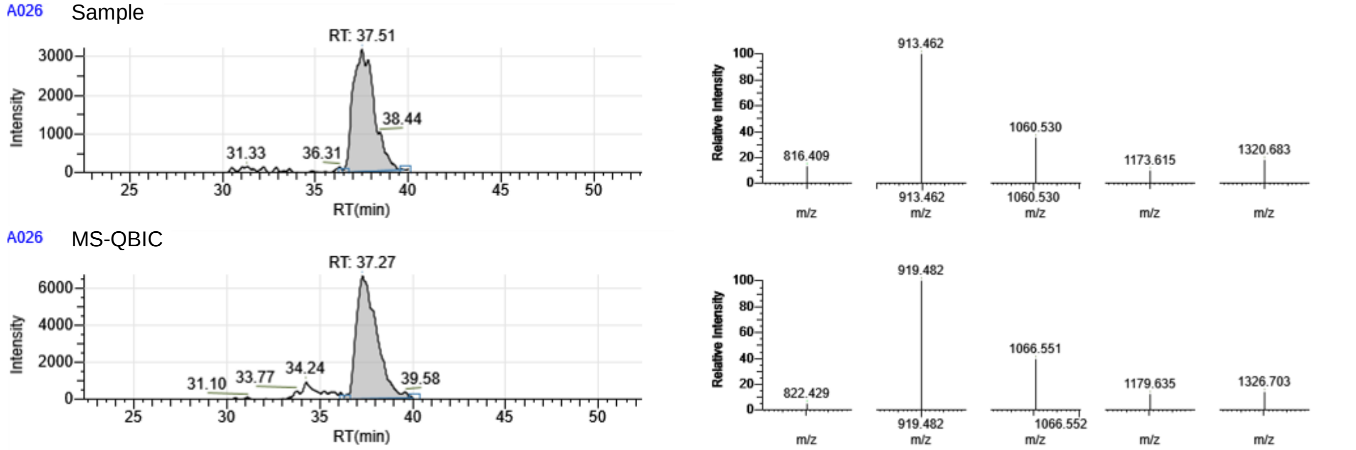

P11

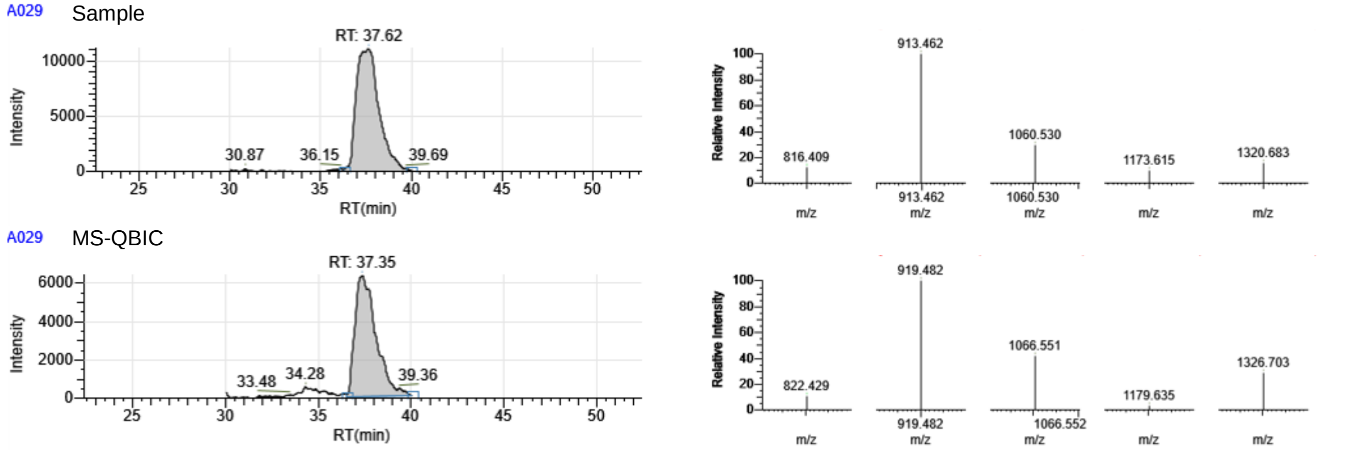

P12

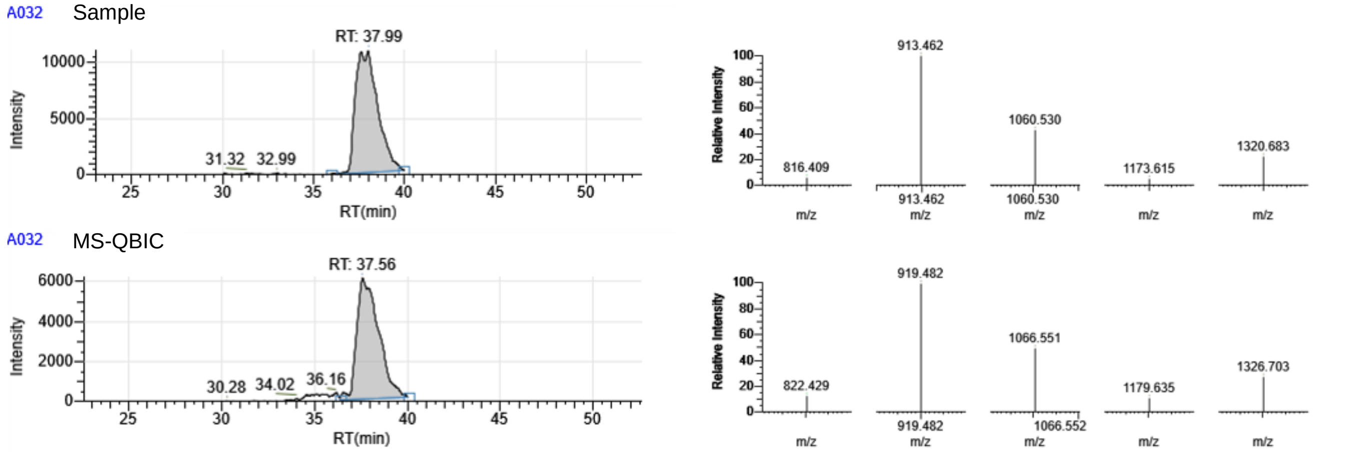

P13

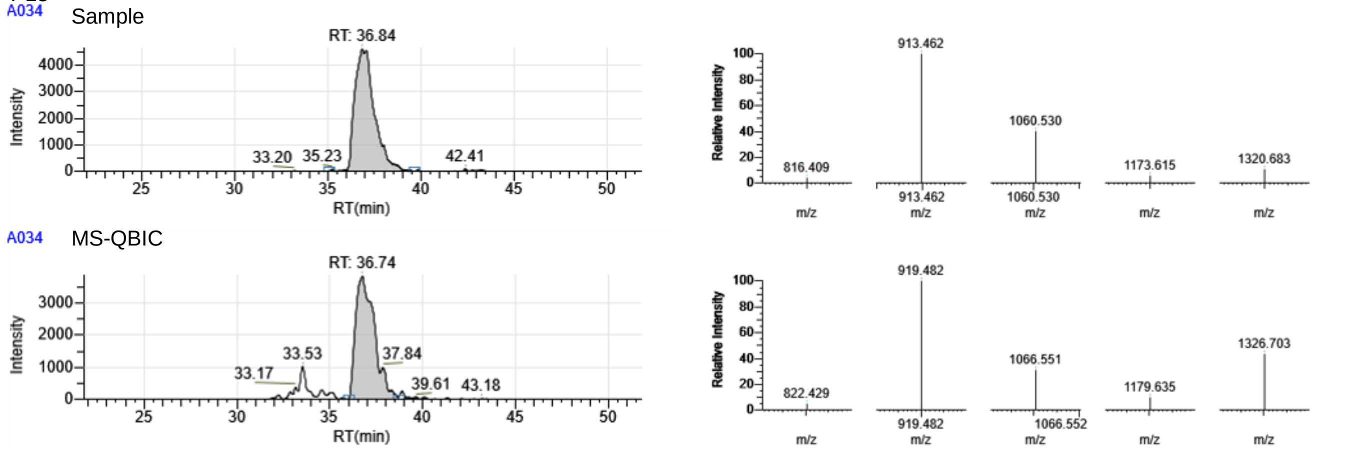

P14

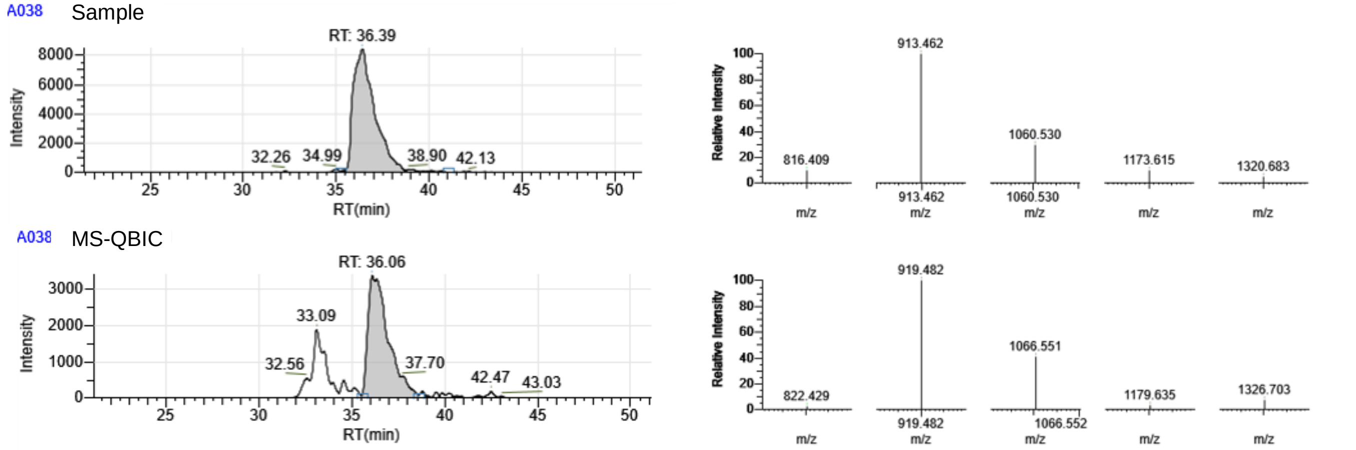

P15

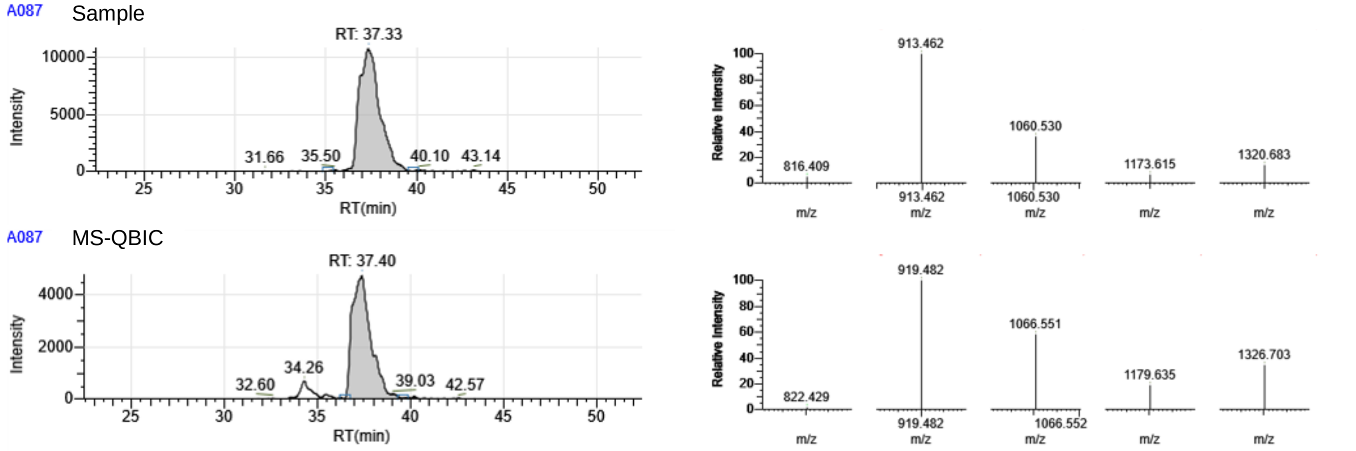

P16

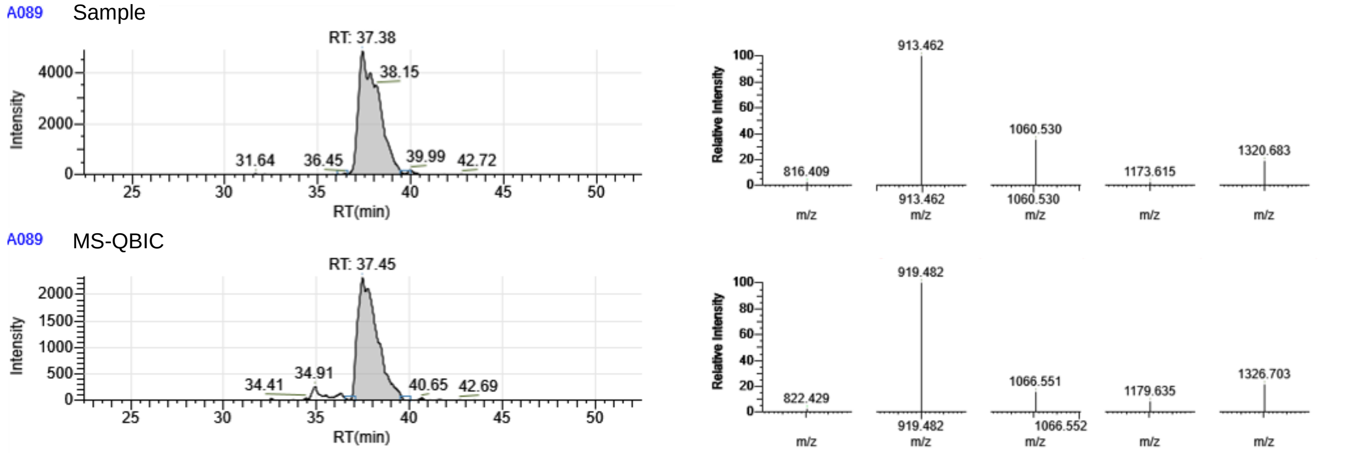

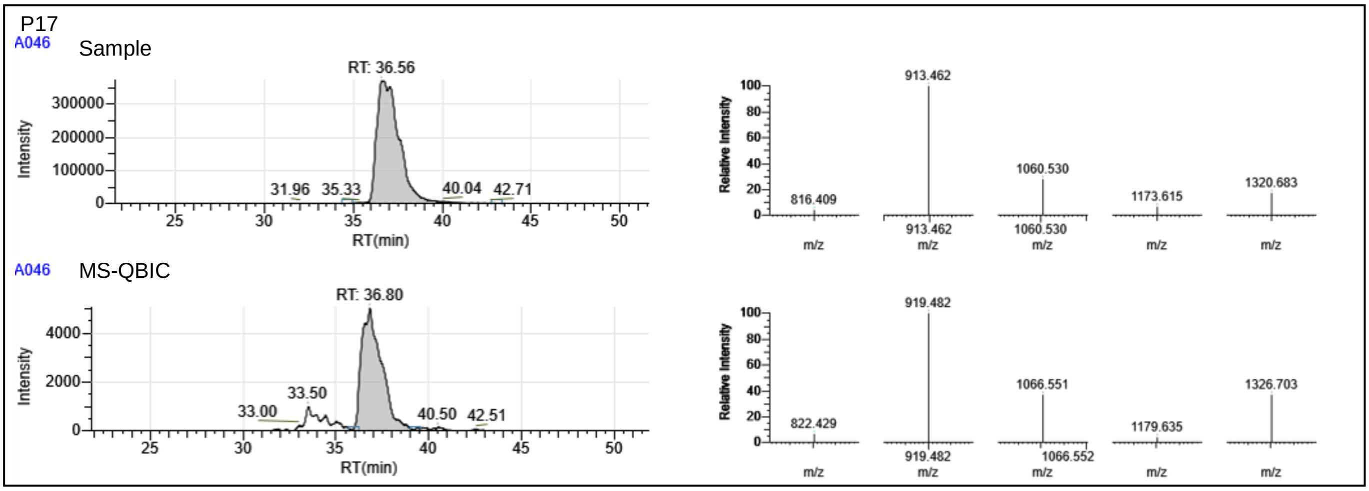
P17
A046 Sample
RT: 36.56
31.96 35.33 40.04 42.71
Intensity
RT(min)
913.462
1060.530
816.409
1173.615
1320.683
Relative Intensity
m/z
A046 MS-QBIC
RT: 36.80
33.50
33.00
40.50 42.51
919.482
1066.551
1066.552
822.429
1179.635
1326.703

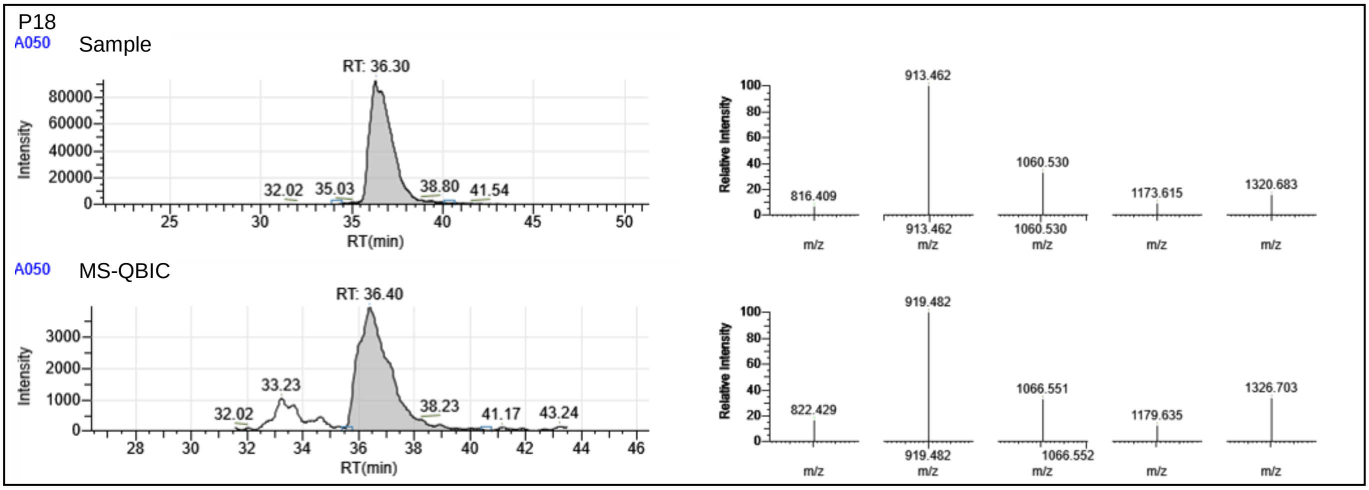
P18
A050 Sample
RT: 36.30
32.02 35.03 38.80 41.54
Intensity
RT(min)
913.462
1060.530
816.409
1173.615
1320.683
Relative Intensity
m/z
A050 MS-QBIC
RT: 36.40
33.23
32.02
38.23
41.17
43.24
919.482
1066.551
1066.552
822.429
1179.635
1326.703

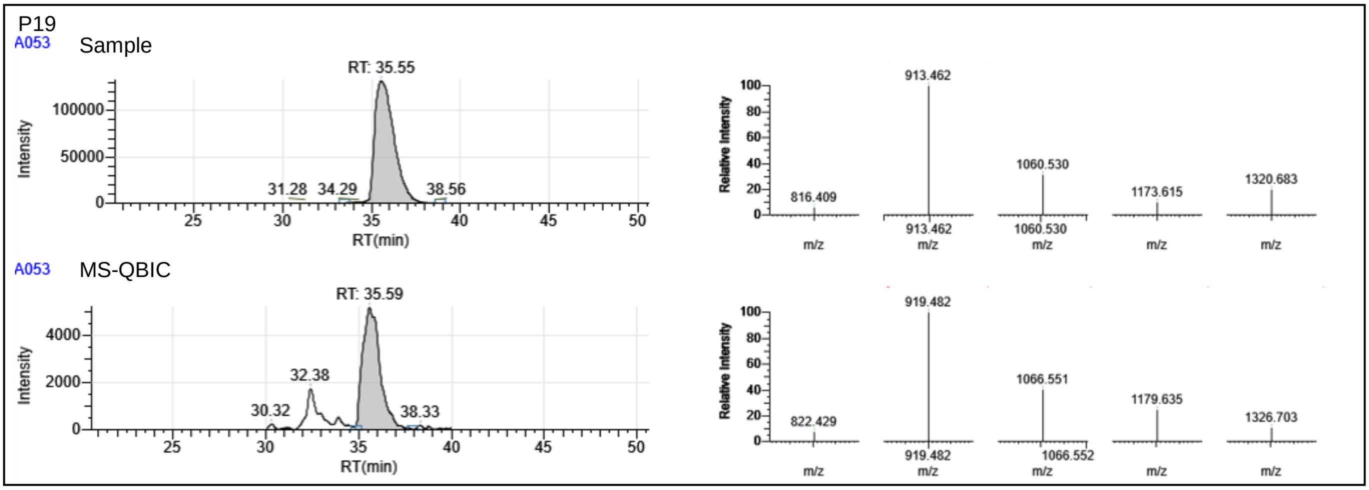
P19
A053 Sample
RT: 35.55
31.28 34.29 38.56
Intensity
RT(min)
913.462
1060.530
816.409
1173.615
1320.683
Relative Intensity
m/z
A053 MS-QBIC
RT: 35.59
32.38
30.32
38.33
919.482
1066.551
1066.552
822.429
1179.635
1326.703

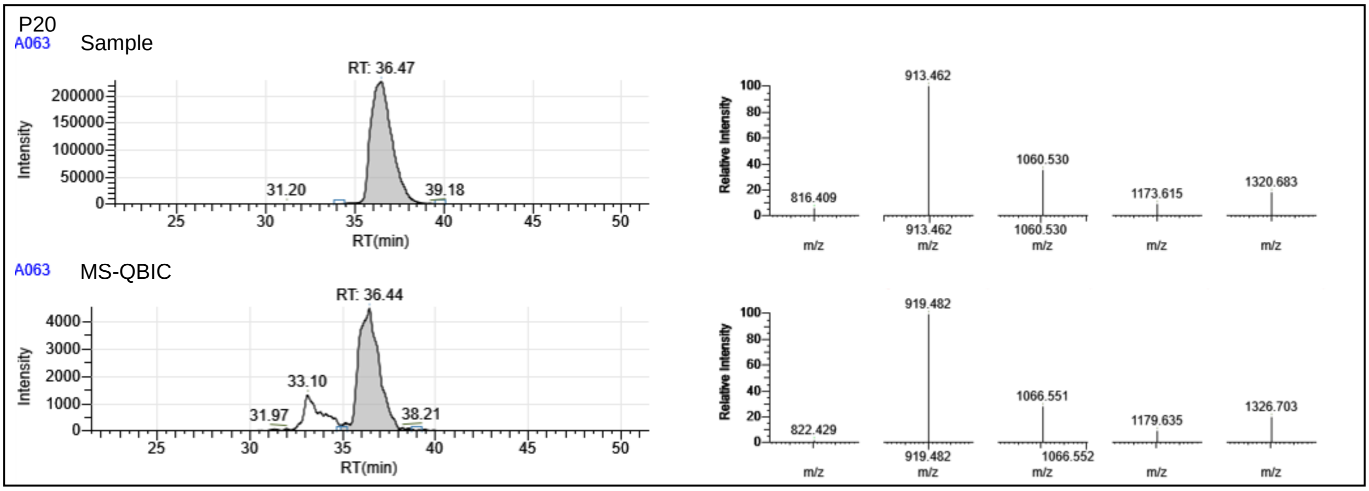
P20
A063 Sample
RT: 36.47
31.20 39.18
Intensity
RT(min)
913.462
1060.530
816.409
1173.615
1320.683
Relative Intensity
m/z
A063 MS-QBIC
RT: 36.44
33.10
31.97
38.21
919.482
1066.551
1066.552
822.429
1179.635
1326.703

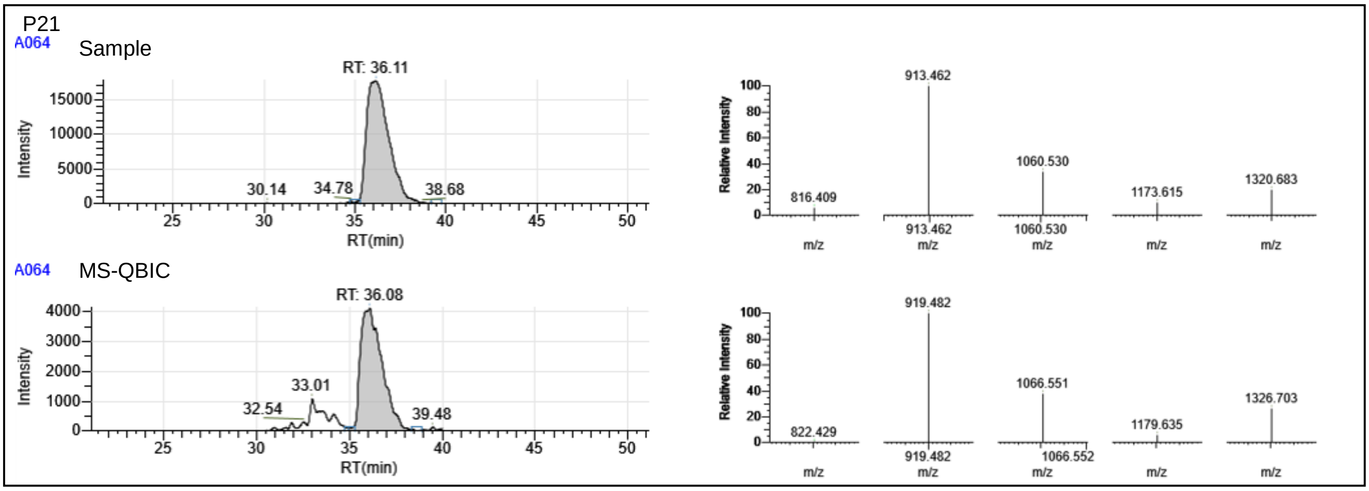
P21
A064 Sample
RT: 36.11
30.14
34.78
38.68
Intensity
RT(min)
913.462
816.409
1060.530
1173.615
1320.683
Relative Intensity
m/z
A064 MS-QBIC
RT: 36.08
33.01
32.54
39.48
919.482
822.429
1066.551
1066.552
1179.635
1326.703

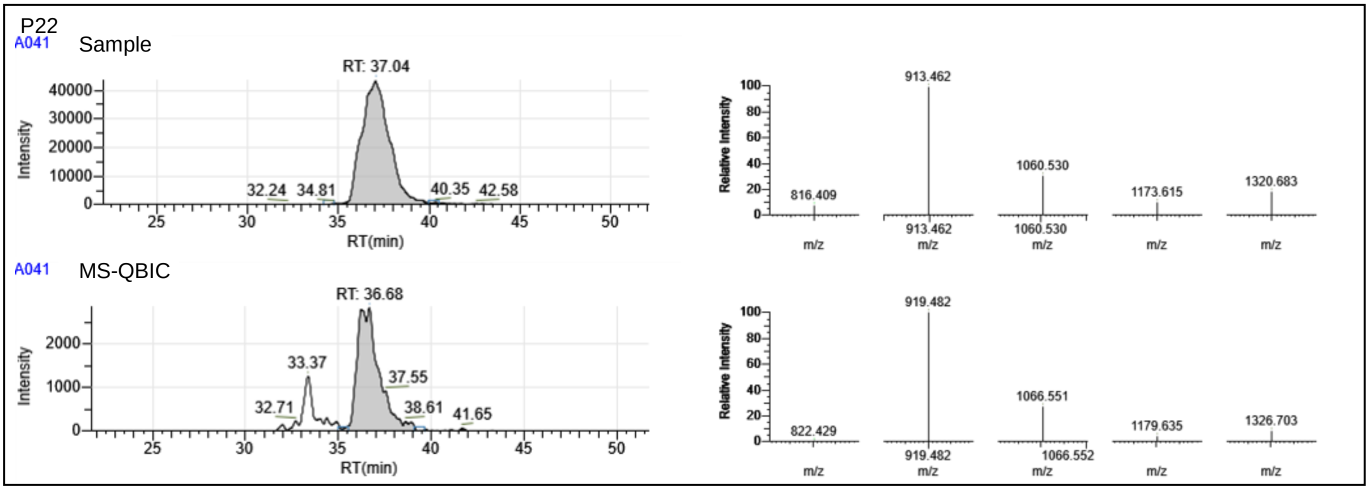
P22
A041 Sample
RT: 37.04
32.24
34.81
40.35
42.58
Intensity
RT(min)
913.462
816.409
1060.530
1173.615
1320.683
Relative Intensity
m/z
A041 MS-QBIC
RT: 36.68
33.37
37.55
32.71
38.61
41.65
919.482
822.429
1066.551
1066.552
1179.635
1326.703

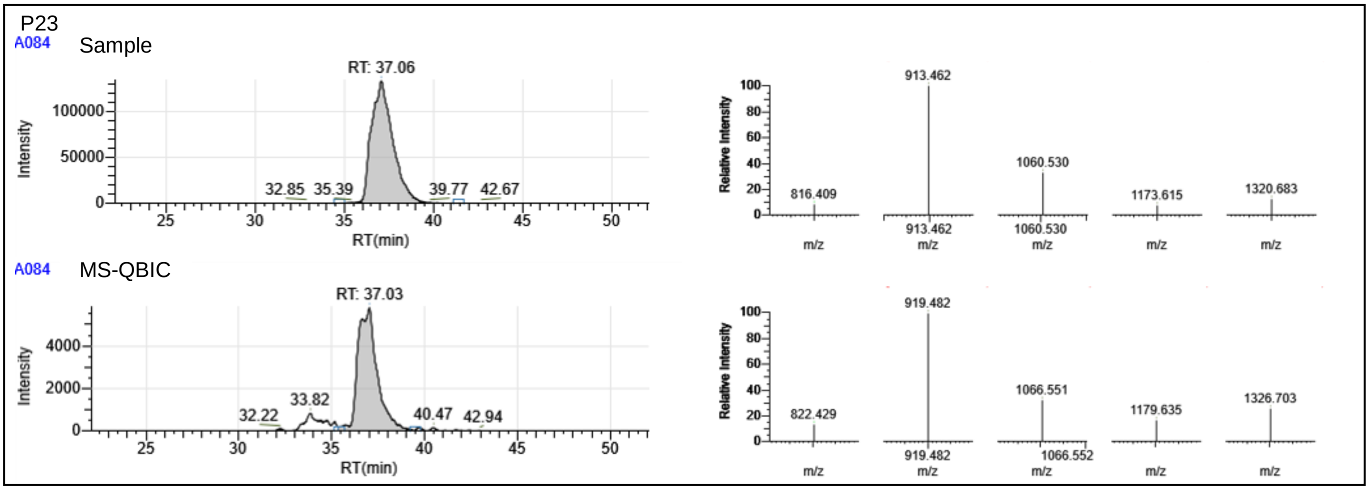
P23
A084 Sample
RT: 37.06
32.85
35.39
39.77
42.67
Intensity
RT(min)
913.462
816.409
1060.530
1173.615
1320.683
Relative Intensity
m/z
A084 MS-QBIC
RT: 37.03
33.82
32.22
40.47
42.94
919.482
822.429
1066.551
1066.552
1179.635
1326.703

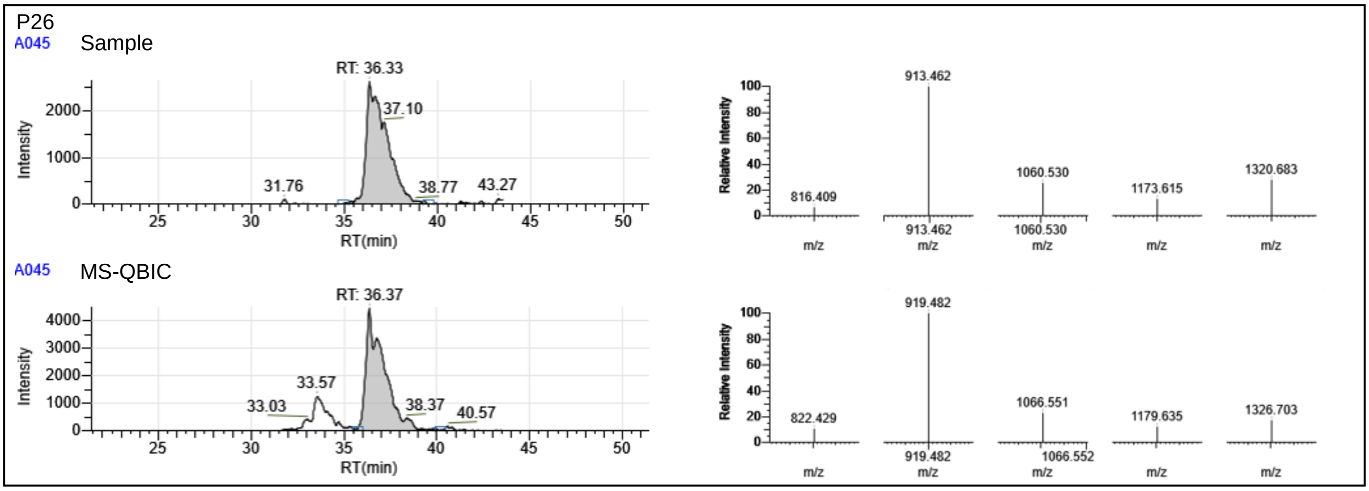
P26
A045 Sample
RT: 36.33
37.10
31.76
38.77
43.27
Intensity
RT(min)
913.462
816.409
1060.530
1173.615
1320.683
Relative Intensity
m/z
A045 MS-QBIC
RT: 36.37
33.57
33.03
38.37
40.57
919.482
822.429
1066.551
1066.552
1179.635
1326.703

P27

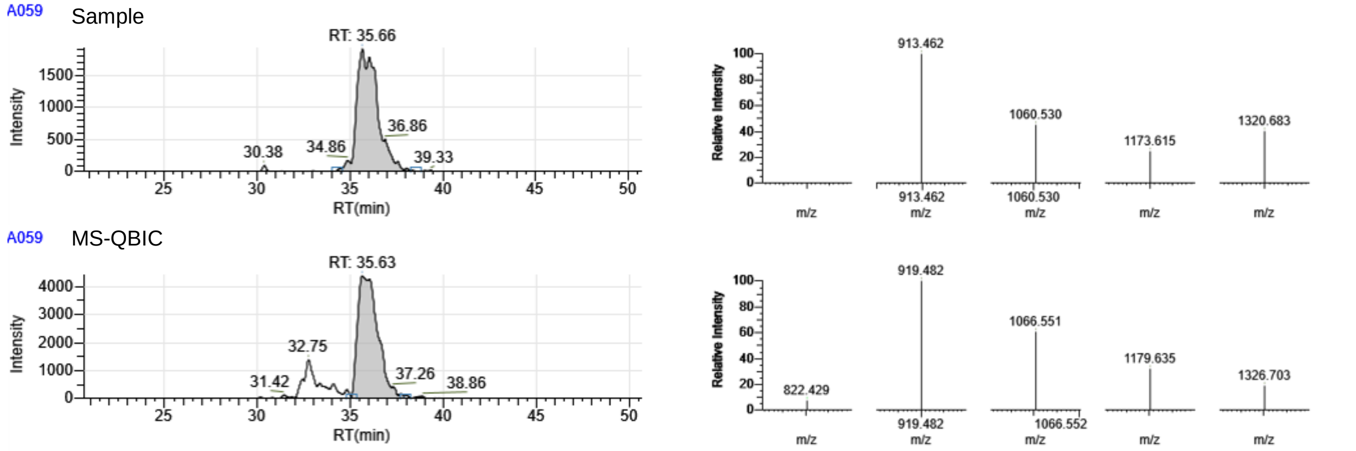

P28

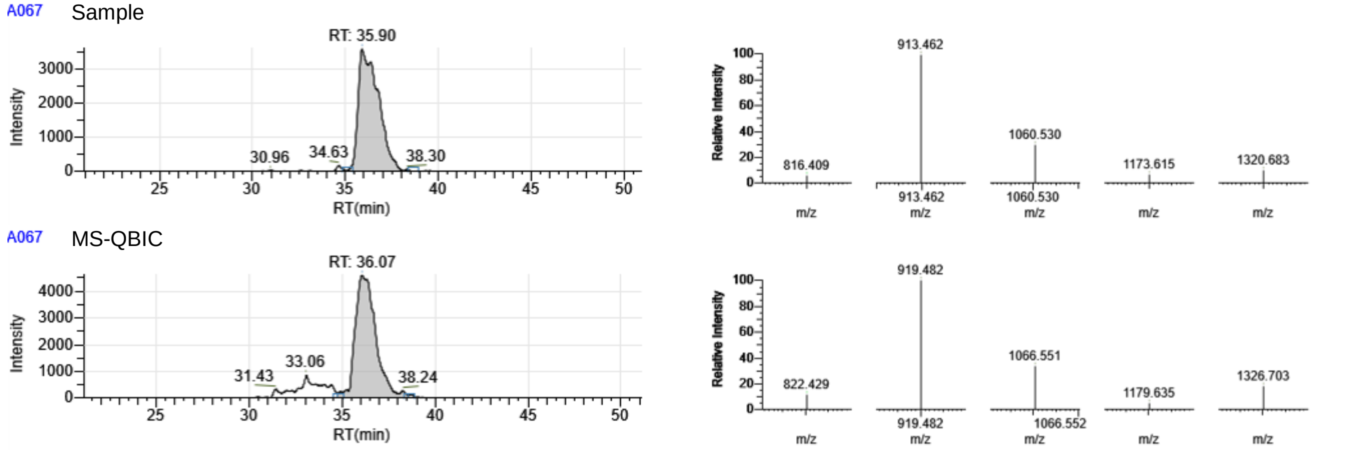

P29

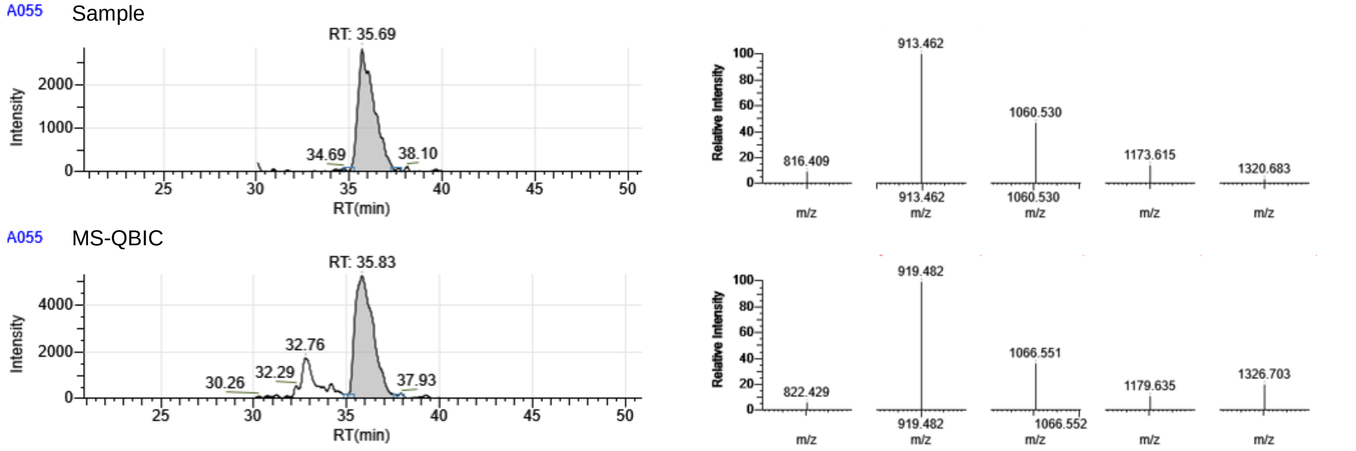

P30

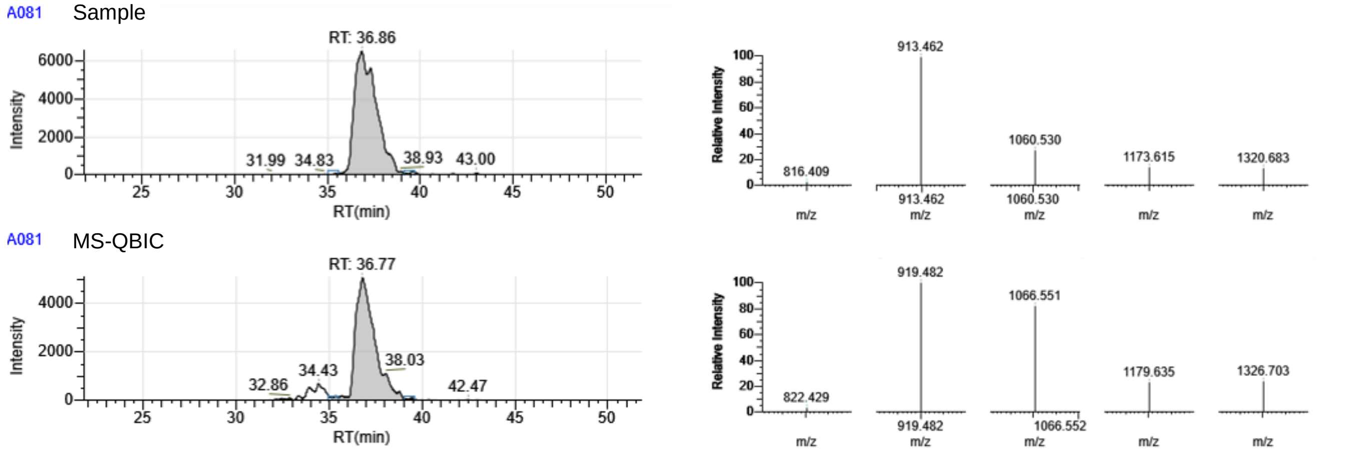

D IGL (YAASSYLSLTPEQWK) Heart

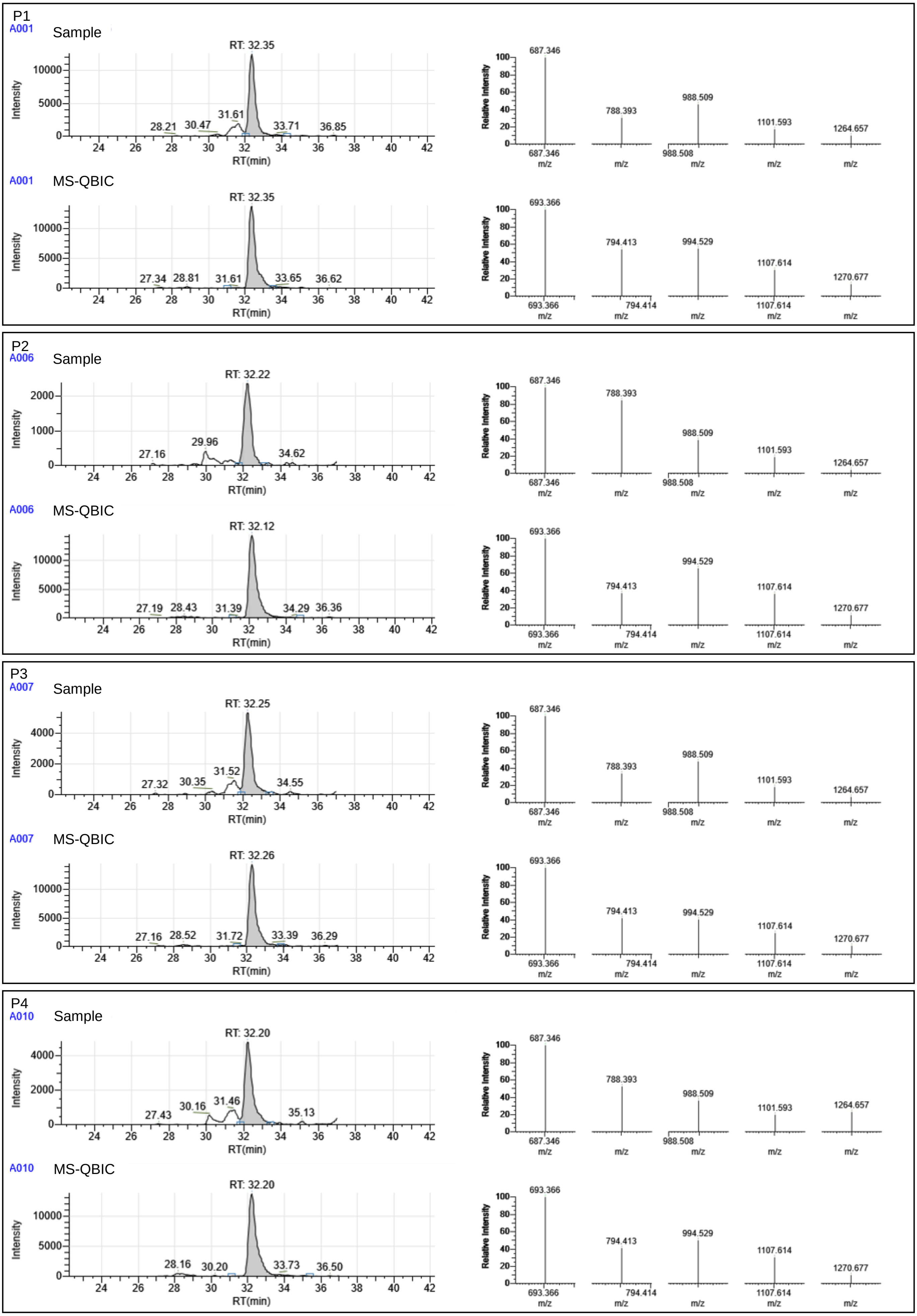

P5

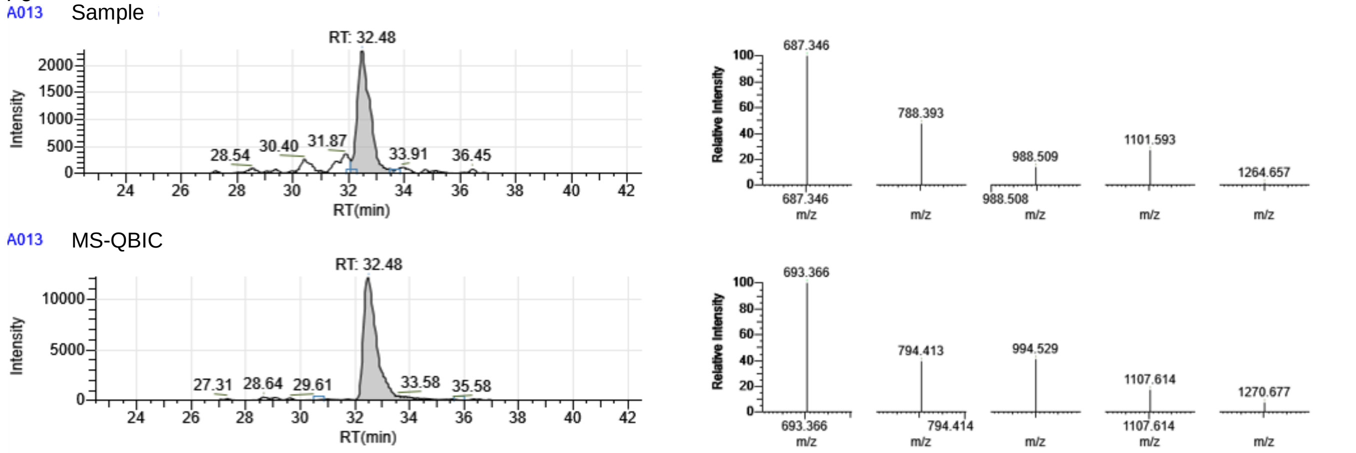

A013 Sample
RT: 32.48
28.54
30.40
31.87
33.91
36.45
Intensity
RT(min)
687.346
788.393
988.509
1101.593
1264.657
988.508
Relative Intensity
m/z
A013 MS-QBIC
RT: 32.48
27.31
28.64
29.61
33.58
35.58
693.366
794.413
994.529
1107.614
1270.677
794.414

P6

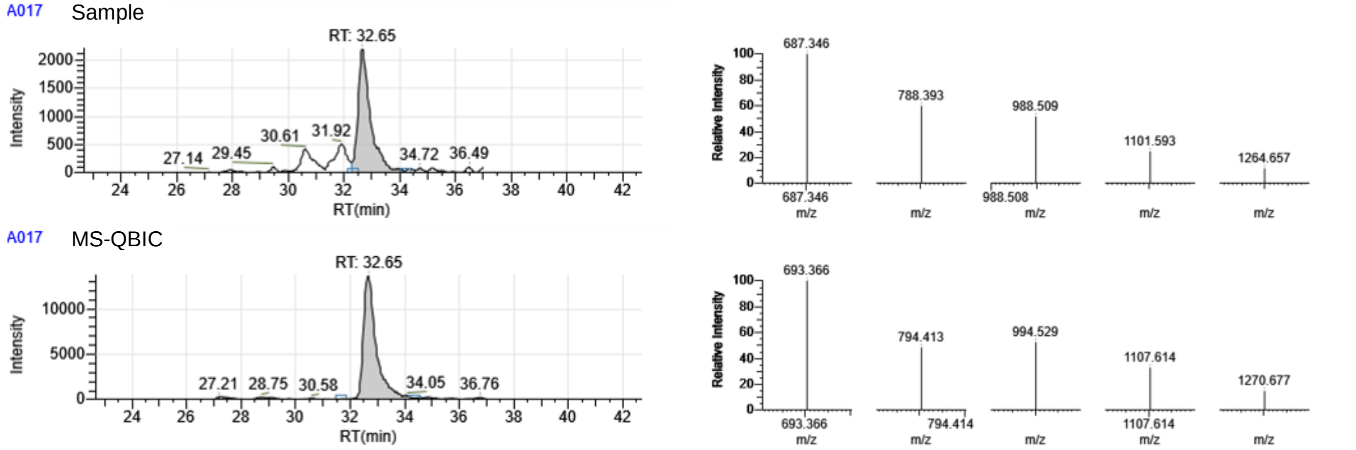

A017 Sample
RT: 32.65
27.14
29.45
30.61
31.92
34.72
36.49
Intensity
RT(min)
687.346
788.393
988.509
1101.593
1264.657
988.508
Relative Intensity
m/z
A017 MS-QBIC
RT: 32.65
27.21
28.75
30.58
34.05
36.76
693.366
794.413
994.529
1107.614
1270.677
794.414

P7

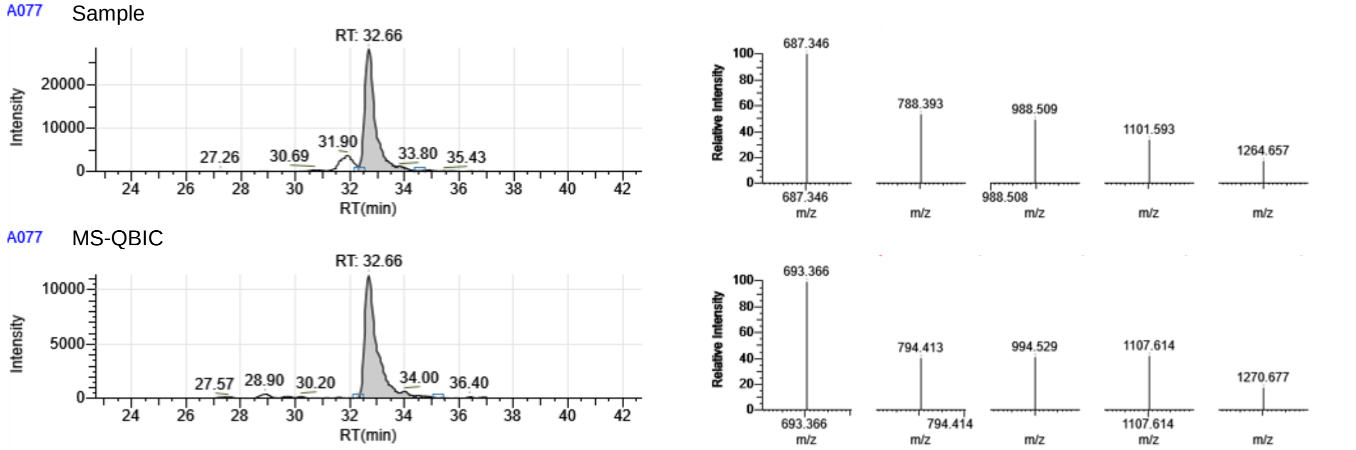

A077 Sample
RT: 32.66
27.26
30.69
31.90
33.80
35.43
Intensity
RT(min)
687.346
788.393
988.509
1101.593
1264.657
988.508
Relative Intensity
m/z
A077 MS-QBIC
RT: 32.66
27.57
28.90
30.20
34.00
36.40
693.366
794.413
994.529
1107.614
1270.677
794.414

P8

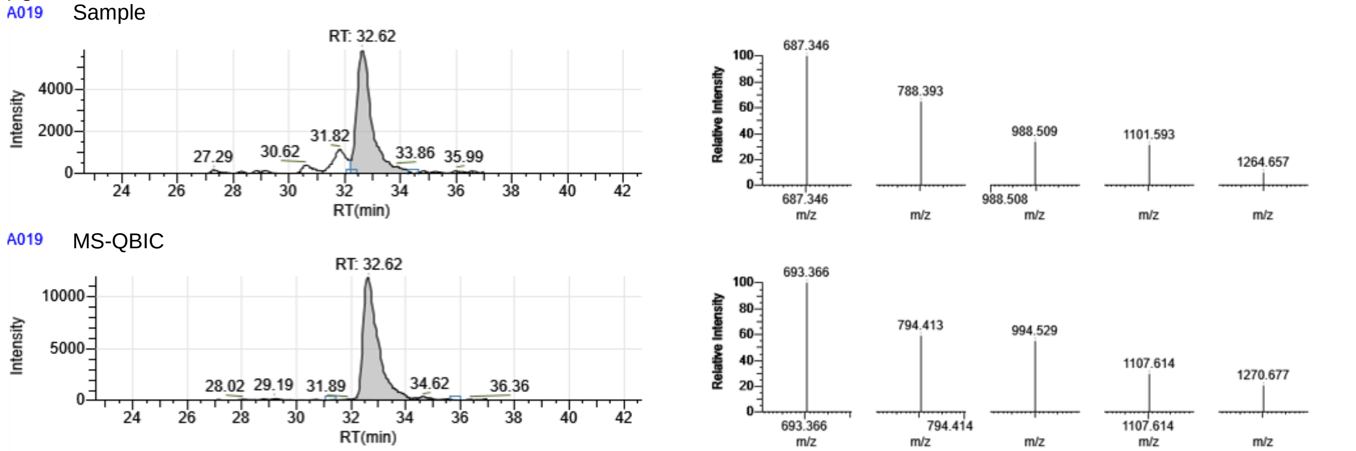

A019 Sample
RT: 32.62
27.29
30.62
31.82
33.86
35.99
Intensity
RT(min)
687.346
788.393
988.509
1101.593
1264.657
988.508
Relative Intensity
m/z
A019 MS-QBIC
RT: 32.62
28.02
29.19
31.89
34.62
36.36
693.366
794.413
994.529
1107.614
1270.677
794.414

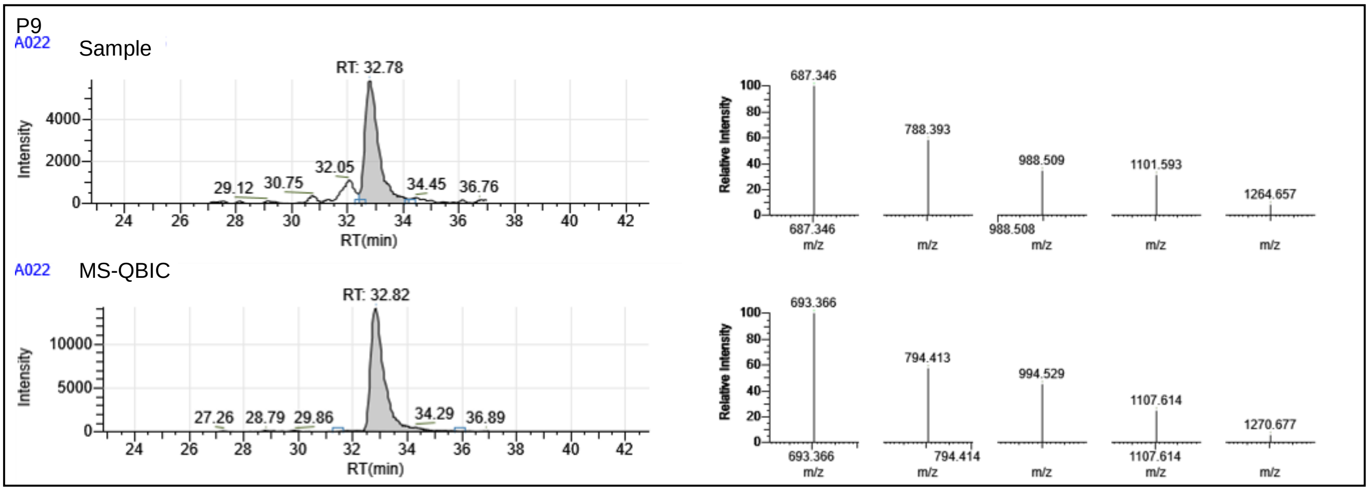
P9
A022 Sample
RT: 32.78
29.12
30.75
32.05
34.45
36.76
Intensity
RT(min)
687.346
788.393
988.509
1101.593
1264.657
988.508
Relative Intensity
m/z
A022 MS-QBIC
RT: 32.82
27.26
28.79
29.86
34.29
36.89
693.366
794.413
794.414
994.529
1107.614
1270.677

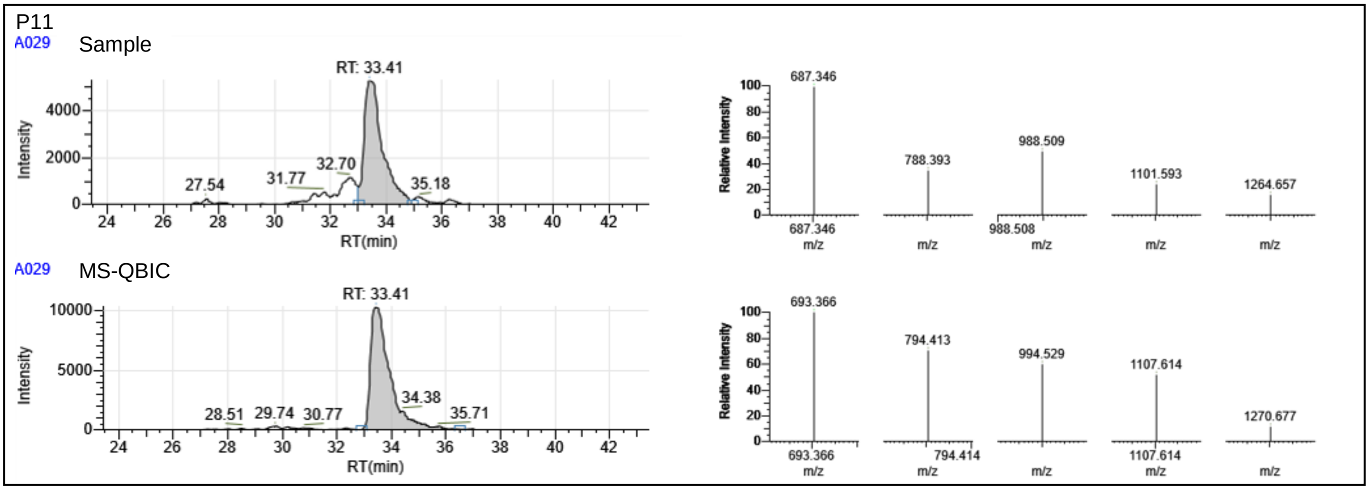
P11
A029 Sample
RT: 33.41
27.54
31.77
32.70
35.18
Intensity
RT(min)
687.346
788.393
988.509
1101.593
1264.657
988.508
Relative Intensity
m/z
A029 MS-QBIC
RT: 33.41
28.51
29.74
30.77
34.38
35.71
693.366
794.413
794.414
994.529
1107.614
1270.677

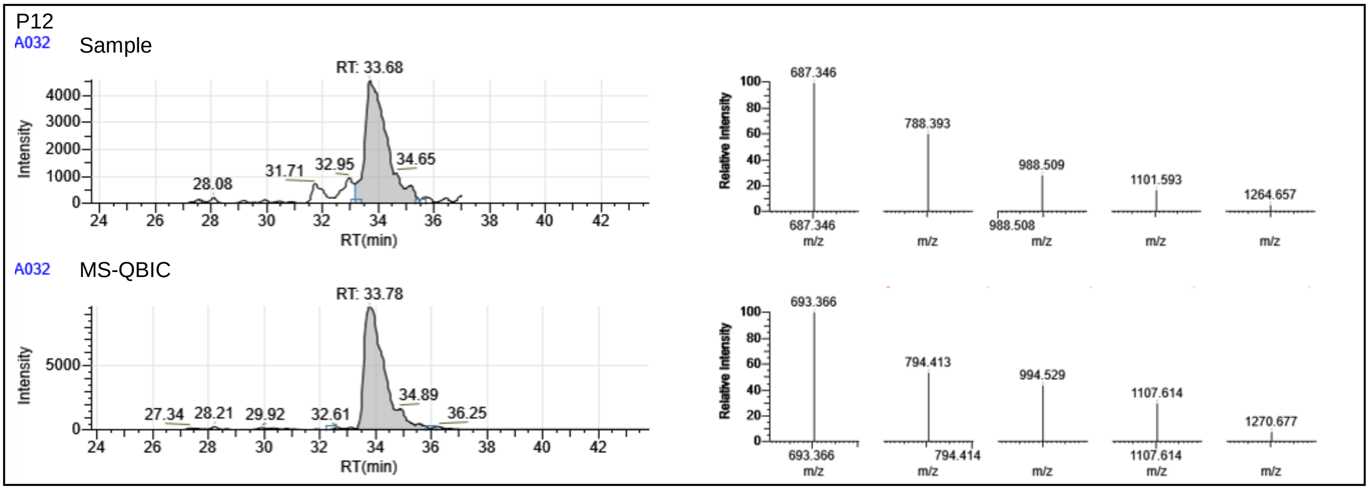
P12
A032 Sample
RT: 33.68
28.08
31.71
32.95
34.65
Intensity
RT(min)
687.346
788.393
988.509
1101.593
1264.657
988.508
Relative Intensity
m/z
A032 MS-QBIC
RT: 33.78
27.34
28.21
29.92
32.61
34.89
36.25
693.366
794.413
794.414
994.529
1107.614
1270.677

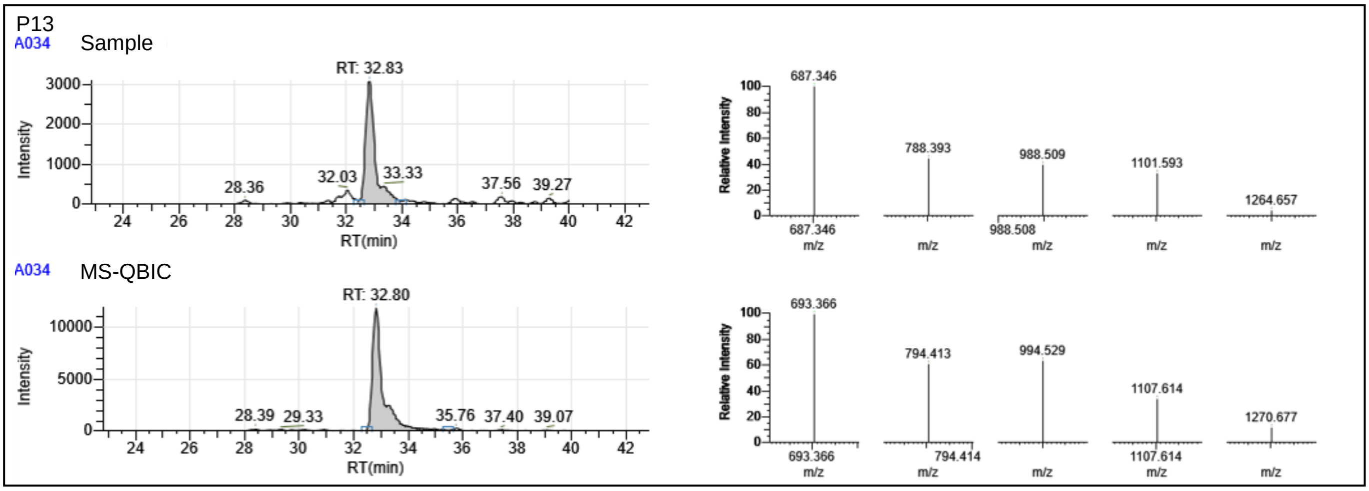
P13
A034 Sample
RT: 32.83
28.36
32.03
33.33
37.56
39.27
Intensity
RT(min)
687.346
788.393
988.509
1101.593
1264.657
988.508
Relative Intensity
m/z
A034 MS-QBIC
RT: 32.80
28.39
29.33
35.76
37.40
39.07
693.366
794.413
794.414
994.529
1107.614
1270.677

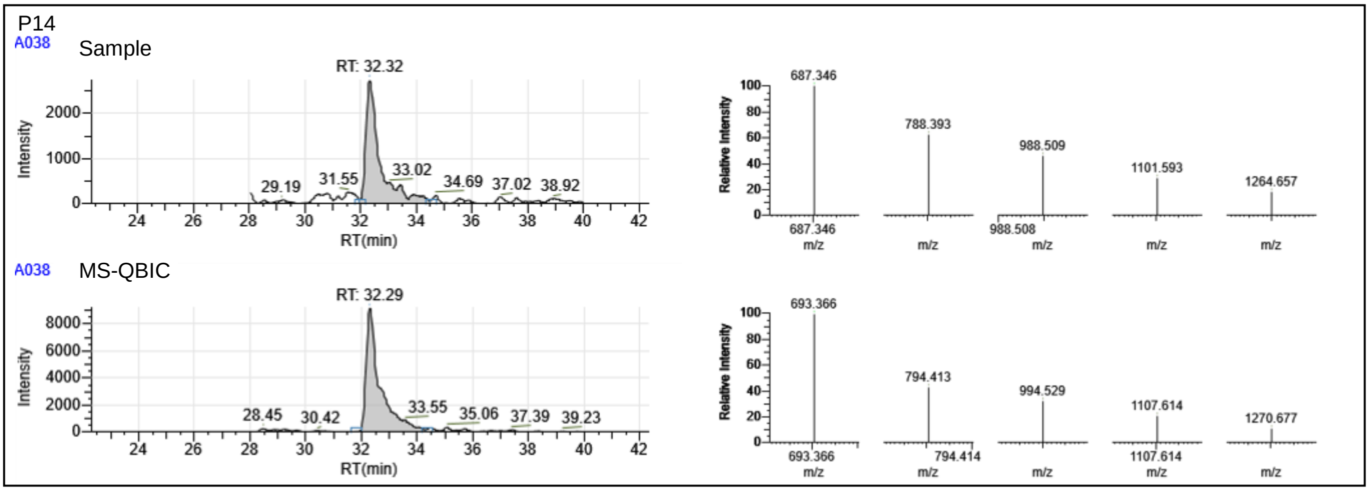
P14
A038 Sample
RT: 32.32
29.19
31.55
33.02
34.69
37.02
38.92
Intensity
RT(min)
687.346
788.393
988.509
1101.593
1264.657
988.508
Relative Intensity
m/z
A038 MS-QBIC
RT: 32.29
28.45
30.42
33.55
35.06
37.39
39.23
693.366
794.413
994.529
1107.614
1270.677
794.414

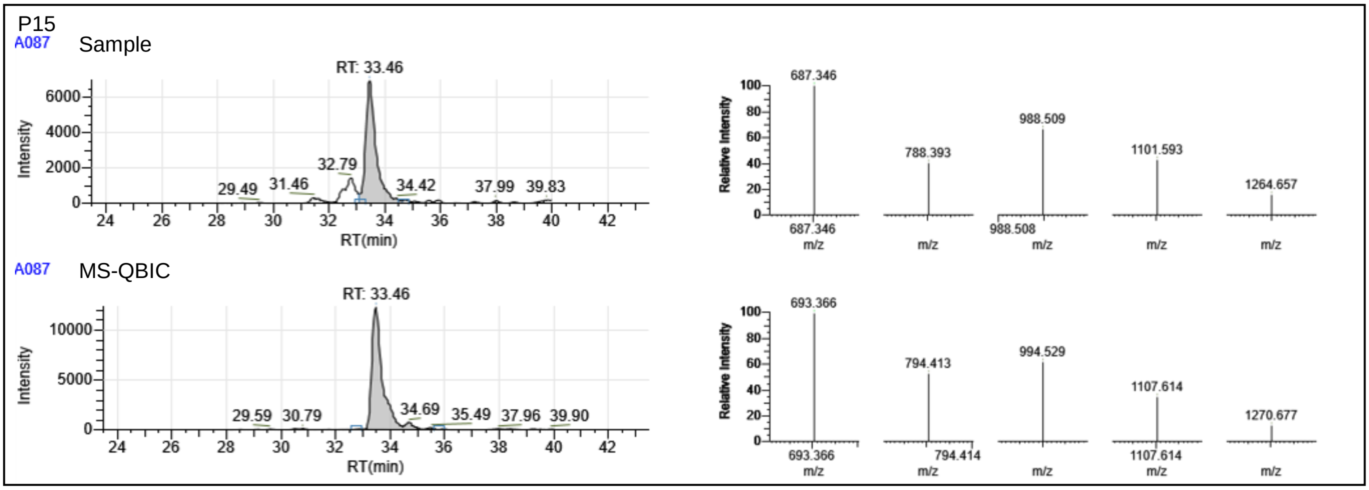
P15
A087 Sample
RT: 33.46
29.49
31.46
32.79
34.42
37.99
39.83
Intensity
RT(min)
687.346
788.393
988.509
1101.593
1264.657
988.508
Relative Intensity
m/z
A087 MS-QBIC
RT: 33.46
29.59
30.79
34.69
35.49
37.96
39.90
693.366
794.413
994.529
1107.614
1270.677
794.414

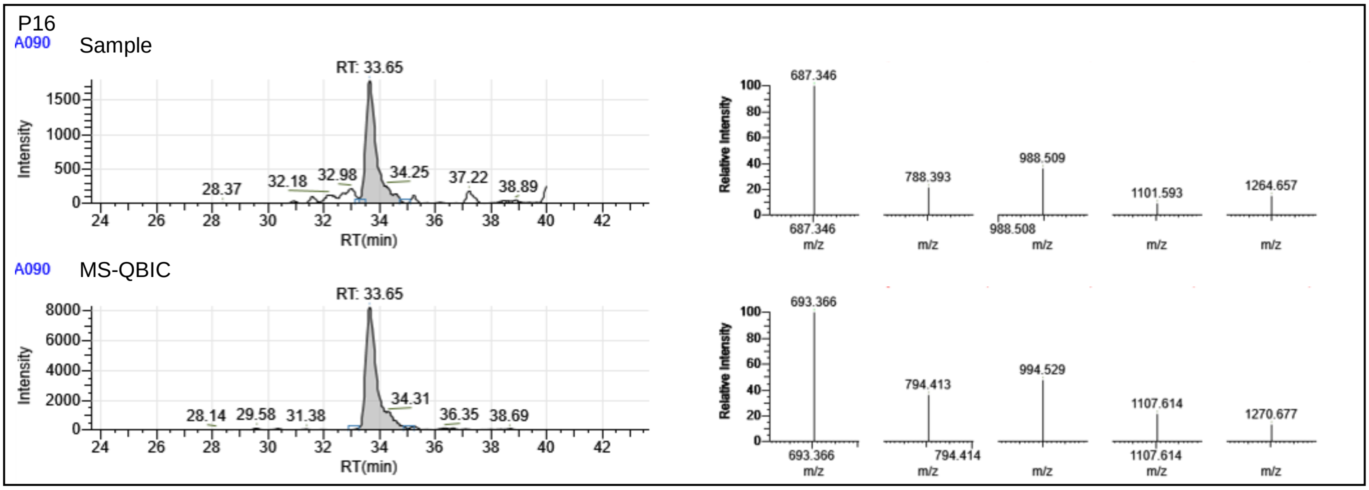
P16
A090 Sample
RT: 33.65
28.37
32.18
32.98
34.25
37.22
38.89
Intensity
RT(min)
687.346
788.393
988.509
1101.593
1264.657
988.508
Relative Intensity
m/z
A090 MS-QBIC
RT: 33.65
28.14
29.58
31.38
34.31
36.35
38.69
693.366
794.413
994.529
1107.614
1270.677
794.414

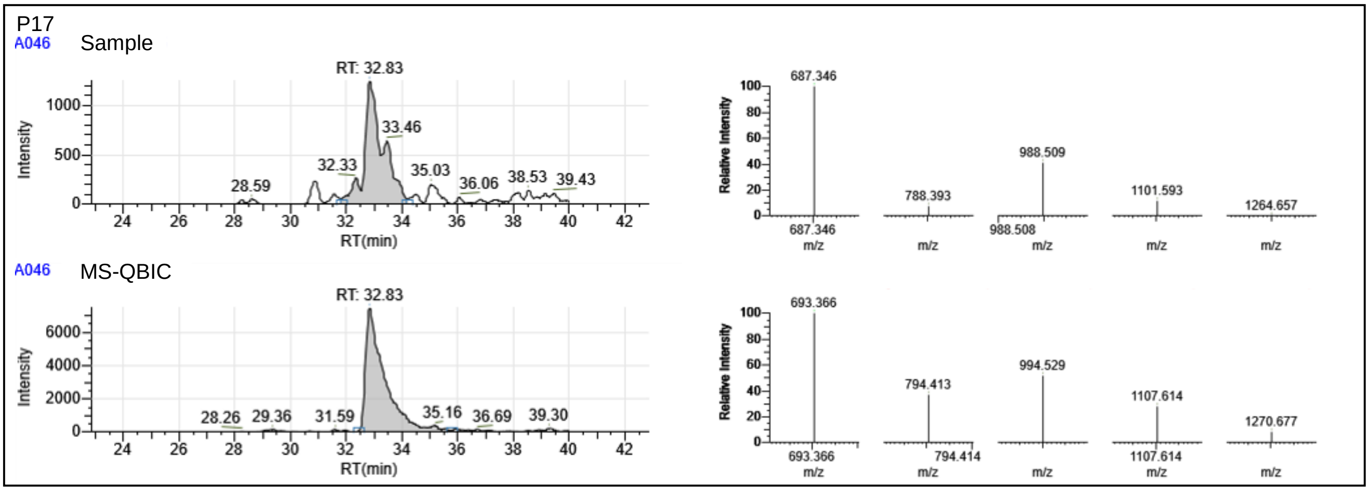
P17
A046 Sample
RT: 32.83
28.59
32.33
33.46
35.03
36.06
38.53
39.43
Intensity
RT(min)
687.346
788.393
988.509
1101.593
1264.657
988.508
Relative Intensity
m/z
A046 MS-QBIC
RT: 32.83
28.26
29.36
31.59
35.16
36.69
39.30
693.366
794.413
994.529
1107.614
1270.677
794.414

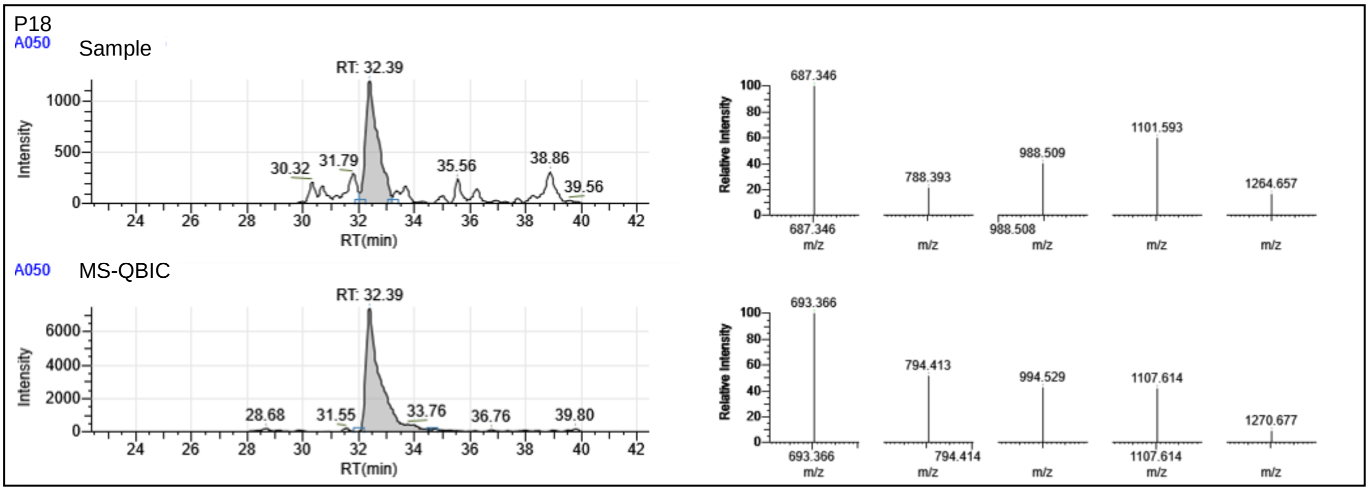
P18
A050 Sample
RT: 32.39
30.32
31.79
35.56
38.86
39.56
Intensity
RT(min)
687.346
788.393
988.509
988.508
1101.593
1264.657
Relative Intensity
m/z
A050 MS-QBIC
RT: 32.39
28.68
31.55
33.76
36.76
39.80
693.366
794.413
794.414
994.529
1107.614
1270.677

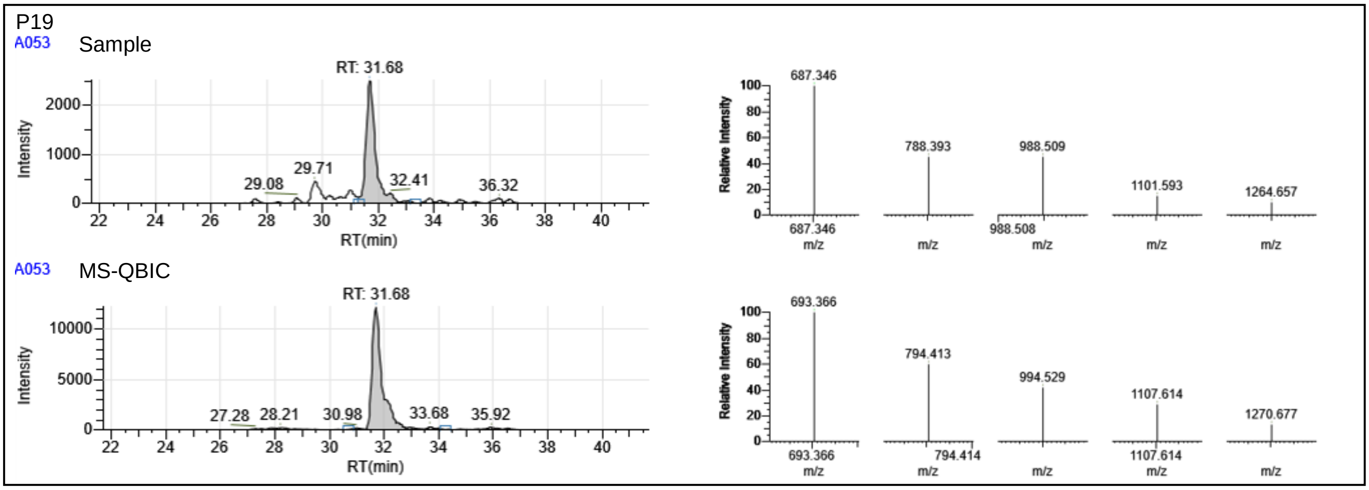
P19
A053 Sample
RT: 31.68
29.08
29.71
32.41
36.32
Intensity
RT(min)
687.346
788.393
988.509
988.508
1101.593
1264.657
Relative Intensity
m/z
A053 MS-QBIC
RT: 31.68
27.28
28.21
30.98
33.68
35.92
693.366
794.413
794.414
994.529
1107.614
1270.677

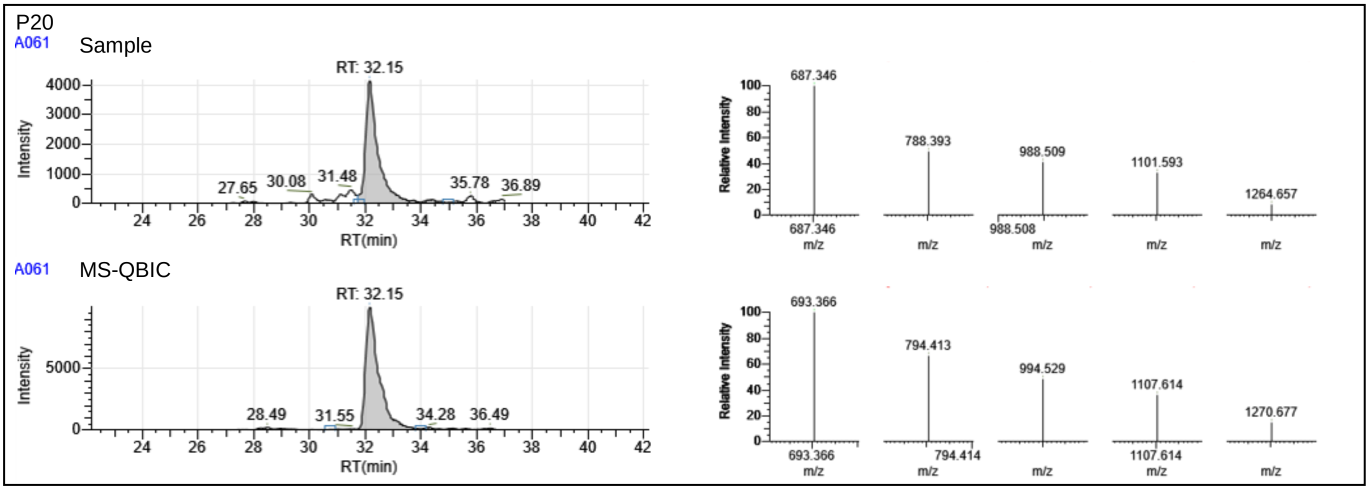
P20
A061 Sample
RT: 32.15
27.65
30.08
31.48
35.78
36.89
Intensity
RT(min)
687.346
788.393
988.509
988.508
1101.593
1264.657
Relative Intensity
m/z
A061 MS-QBIC
RT: 32.15
28.49
31.55
34.28
36.49
693.366
794.413
794.414
994.529
1107.614
1270.677

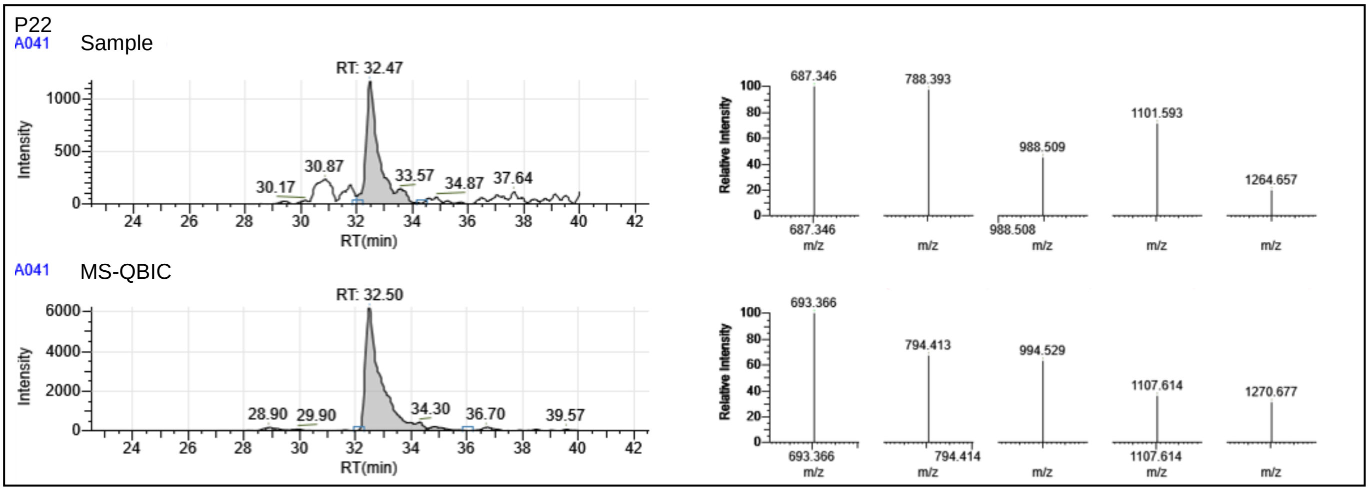
P22
A041 Sample
RT: 32.47
30.17
30.87
33.57
34.87
37.64
Intensity
RT(min)
687.346
788.393
988.509
988.508
1101.593
1264.657
Relative Intensity
m/z
A041 MS-QBIC
RT: 32.50
28.90
29.90
34.30
36.70
39.57
693.366
794.413
794.414
994.529
1107.614
1270.677

P23

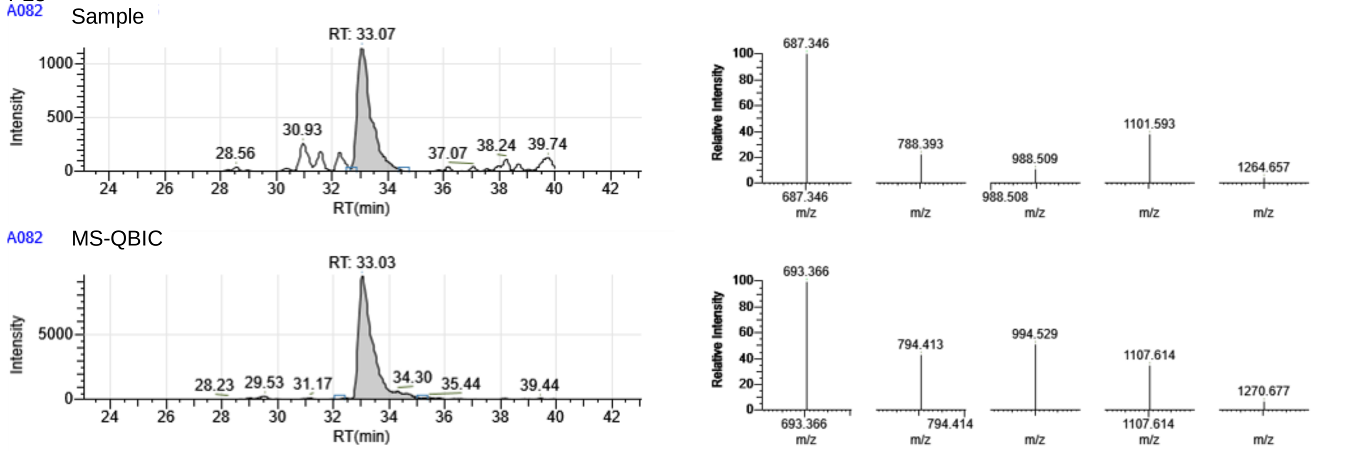

P24

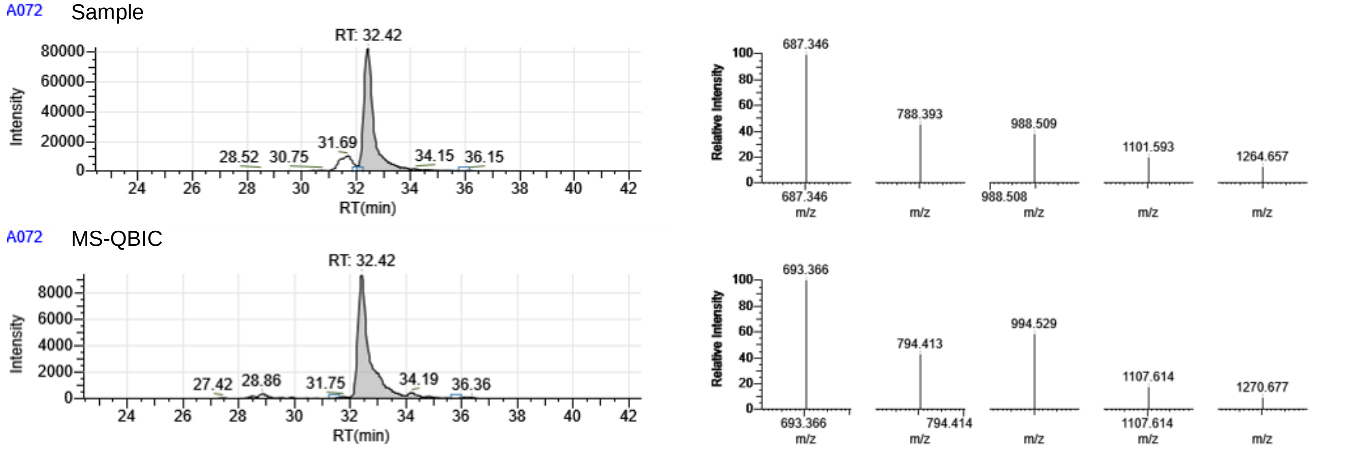

P25

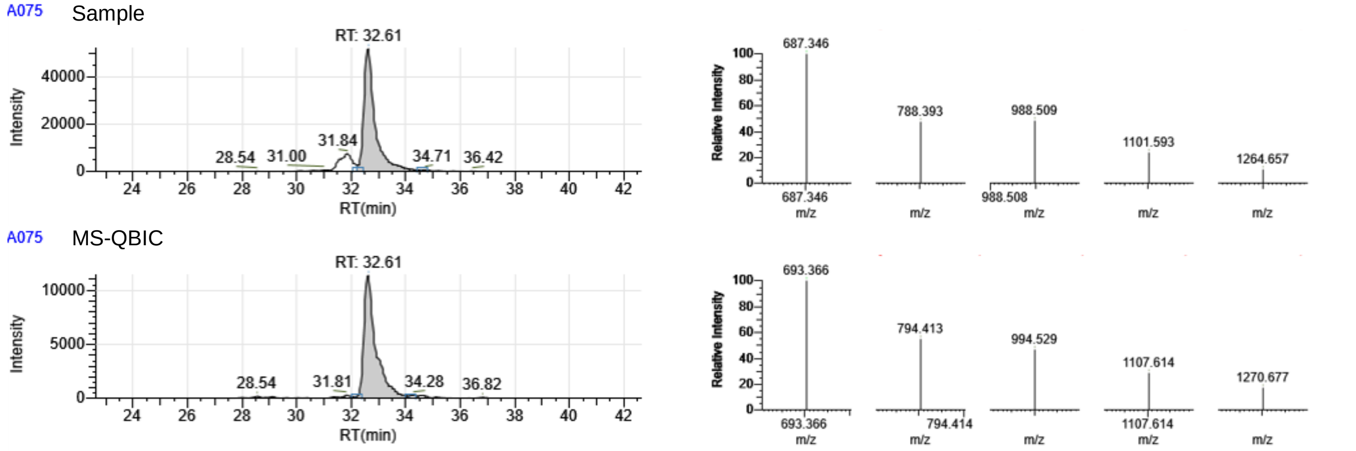

P26

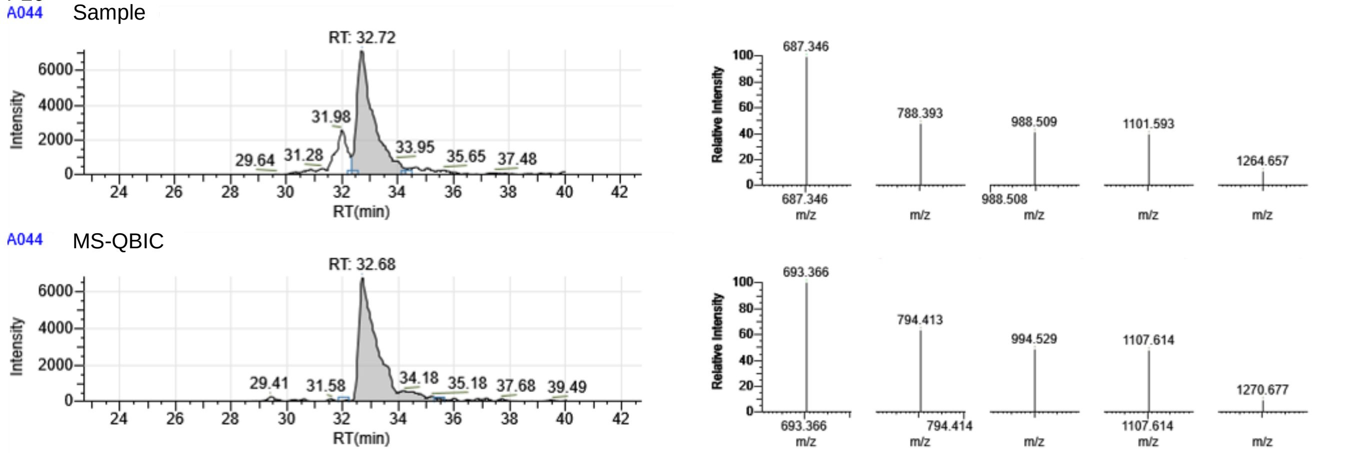

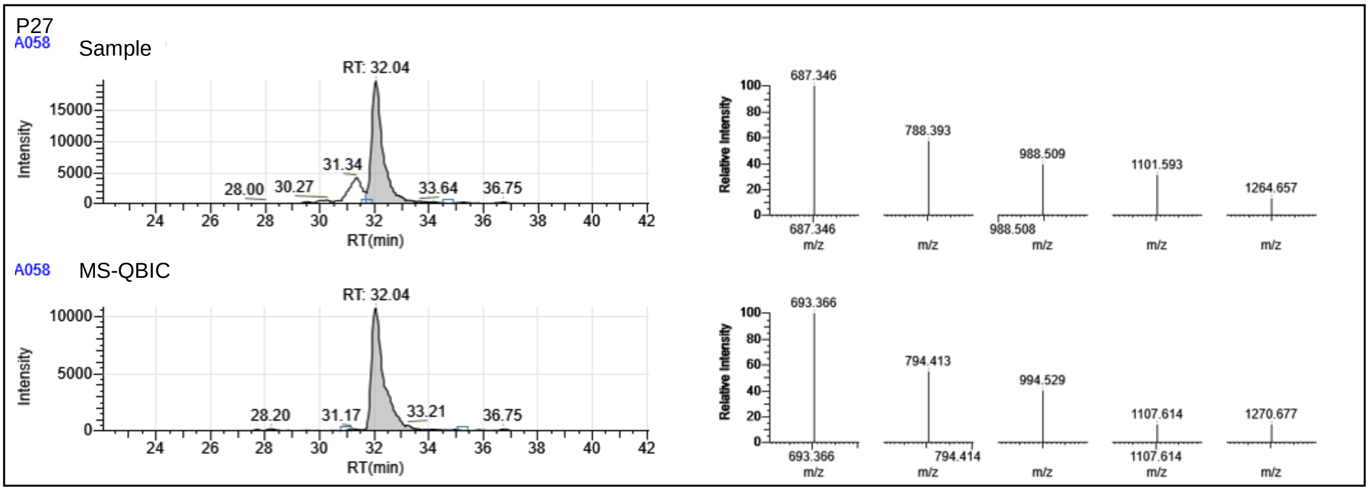
P27
A058 Sample
RT: 32.04
31.34
28.00
30.27
33.64
36.75
Intensity
RT(min)
687.346
788.393
988.509
1101.593
1264.657
988.508
Relative Intensity
m/z
A058 MS-QBIC
RT: 32.04
28.20
31.17
33.21
36.75
693.366
794.413
994.529
1107.614
1270.677
794.414

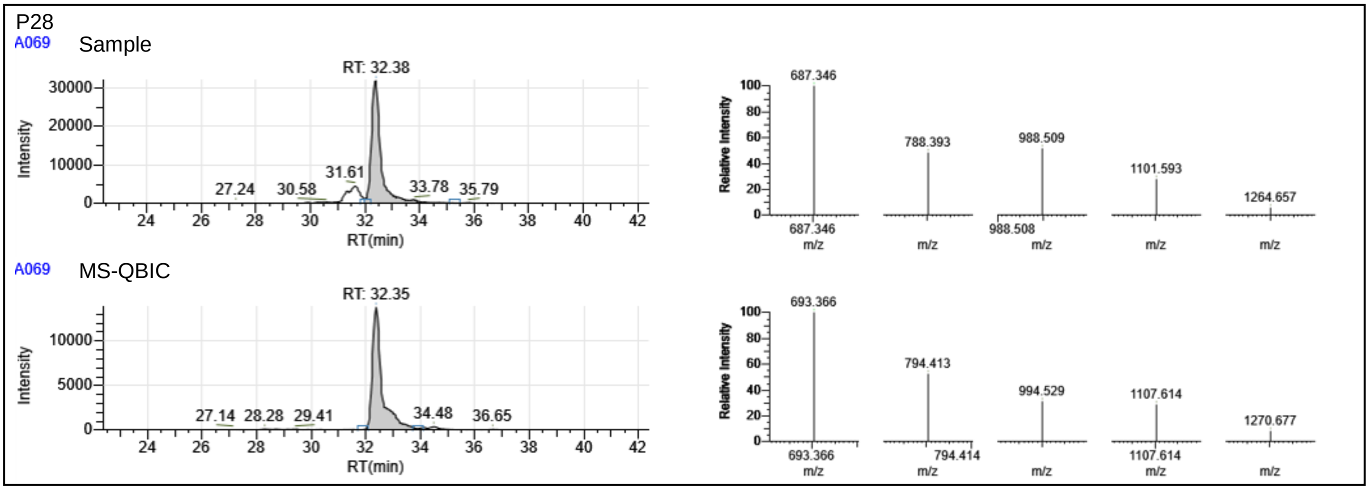
P28
A069 Sample
RT: 32.38
31.61
27.24
30.58
33.78
35.79
Intensity
RT(min)
687.346
788.393
988.509
1101.593
1264.657
988.508
Relative Intensity
m/z
A069 MS-QBIC
RT: 32.35
27.14
28.28
29.41
34.48
36.65
693.366
794.413
994.529
1107.614
1270.677
794.414

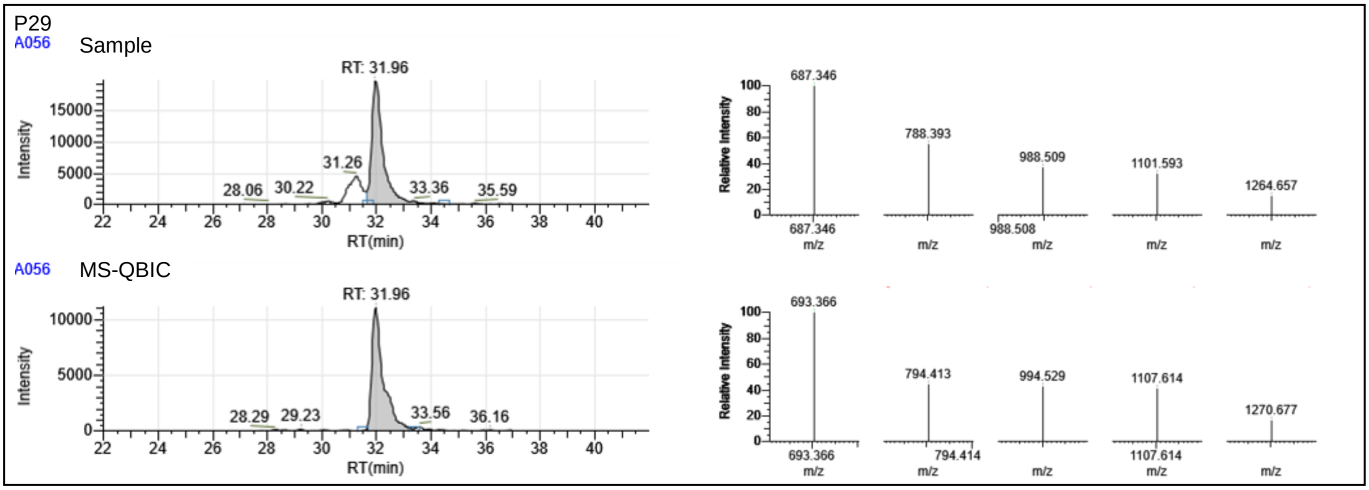
P29
A056 Sample
RT: 31.96
31.26
28.06
30.22
33.36
35.59
Intensity
RT(min)
687.346
788.393
988.509
1101.593
1264.657
988.508
Relative Intensity
m/z
A056 MS-QBIC
RT: 31.96
28.29
29.23
33.56
36.16
693.366
794.413
994.529
1107.614
1270.677
794.414

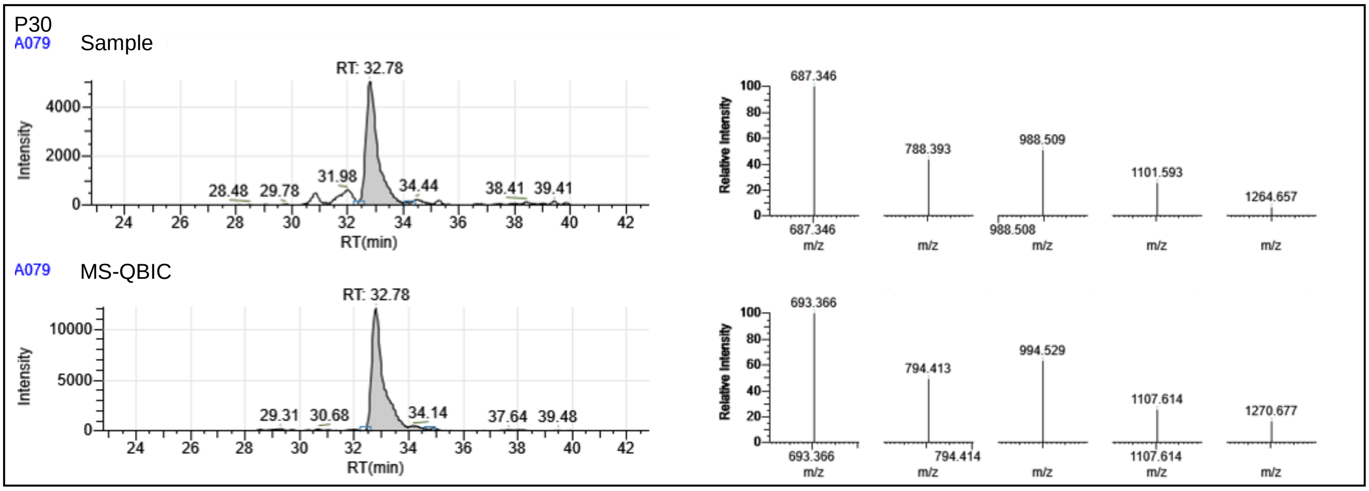
P30
A079 Sample
RT: 32.78
31.98
28.48
29.78
34.44
38.41
39.41
Intensity
RT(min)
687.346
788.393
988.509
1101.593
1264.657
988.508
Relative Intensity
m/z
A079 MS-QBIC
RT: 32.78
29.31
30.68
34.14
37.64
39.48
693.366
794.413
994.529
1107.614
1270.677
794.414

E B2M (IQVYSR) Heart

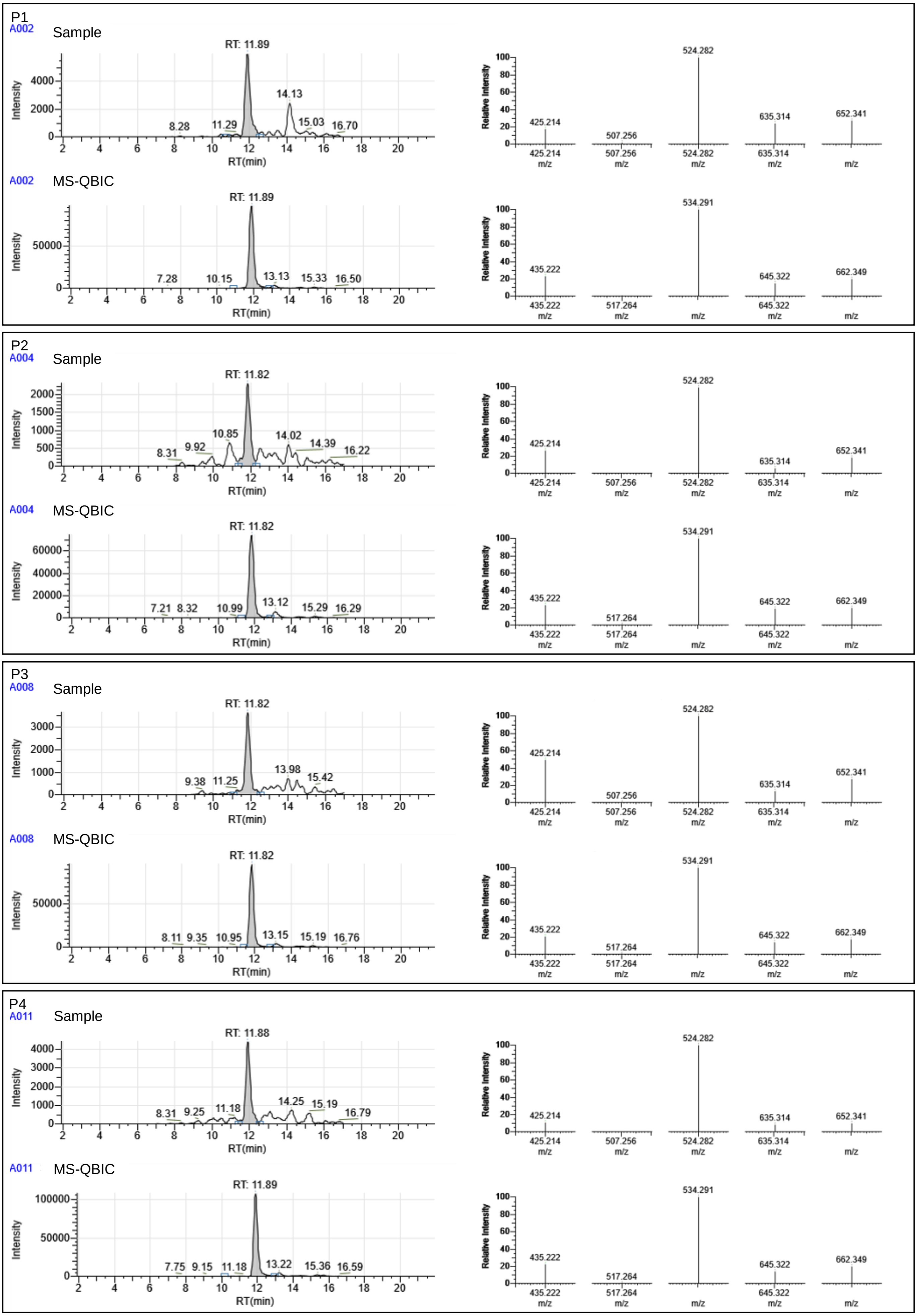

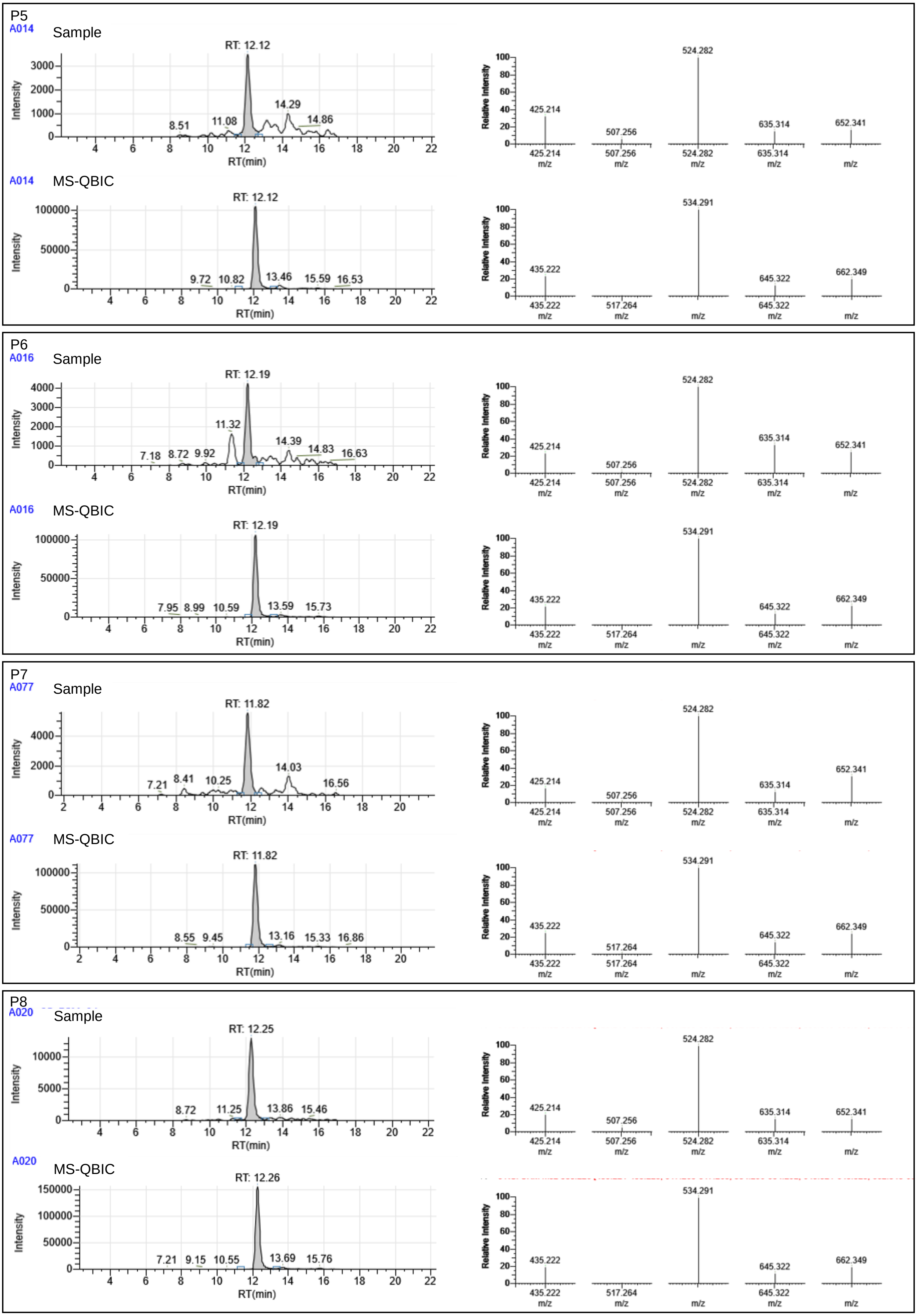
P5
A014 Sample
RT: 12.12
8.51
11.08
14.29
14.86
Intensity
RT(min)
Relative Intensity
425.214
507.256
524.282
635.314
652.341
m/z
A014 MS-QBIC
RT: 12.12
9.72
10.82
13.46
15.59
16.53
435.222
517.264
534.291
645.322
662.349
P6
A016 Sample
RT: 12.19
7.18
8.72
9.92
11.32
14.39
14.83
16.63
A016 MS-QBIC
RT: 12.19
7.95
8.99
10.59
13.59
15.73
P7
A077 Sample
RT: 11.82
7.21
8.41
10.25
14.03
16.56
A077 MS-QBIC
RT: 11.82
8.55
9.45
13.16
15.33
16.86
P8
A020 Sample
RT: 12.25
8.72
11.25
13.86
15.46
A020 MS-QBIC
RT: 12.26
7.21
9.15
10.55
13.69
15.76

P9

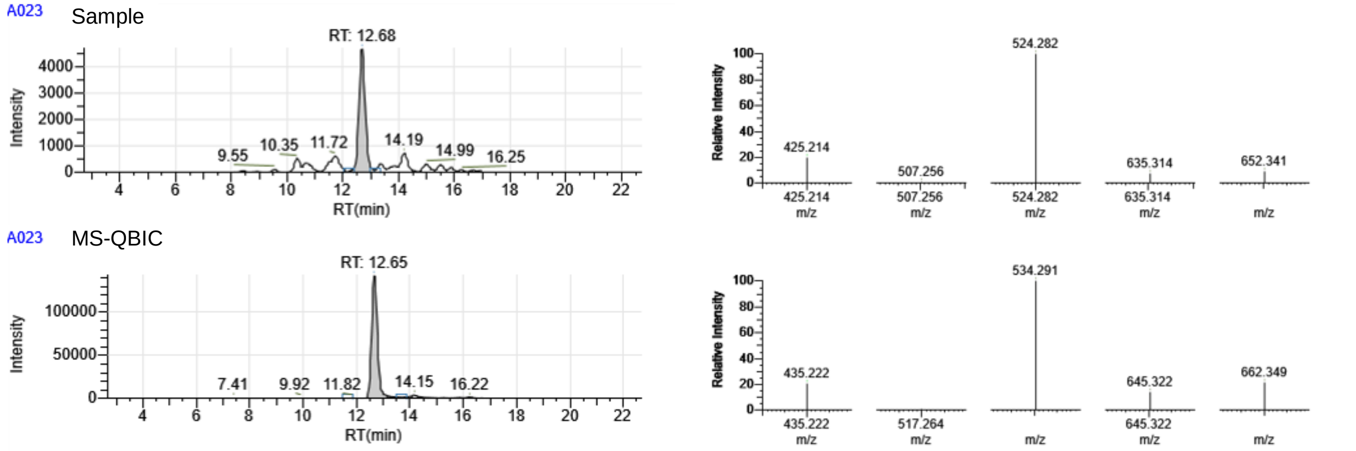

P10

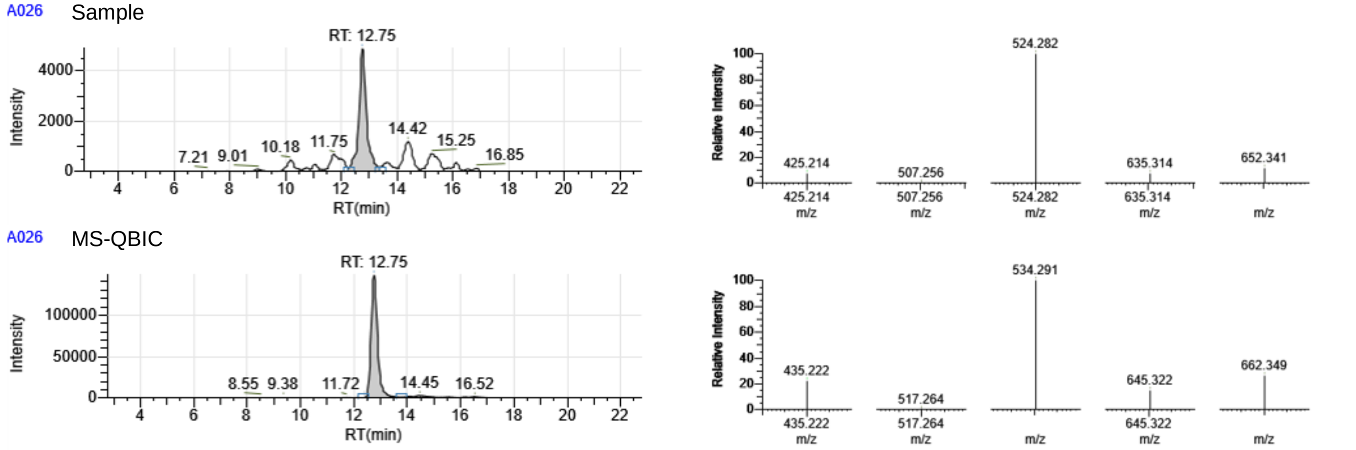

P11

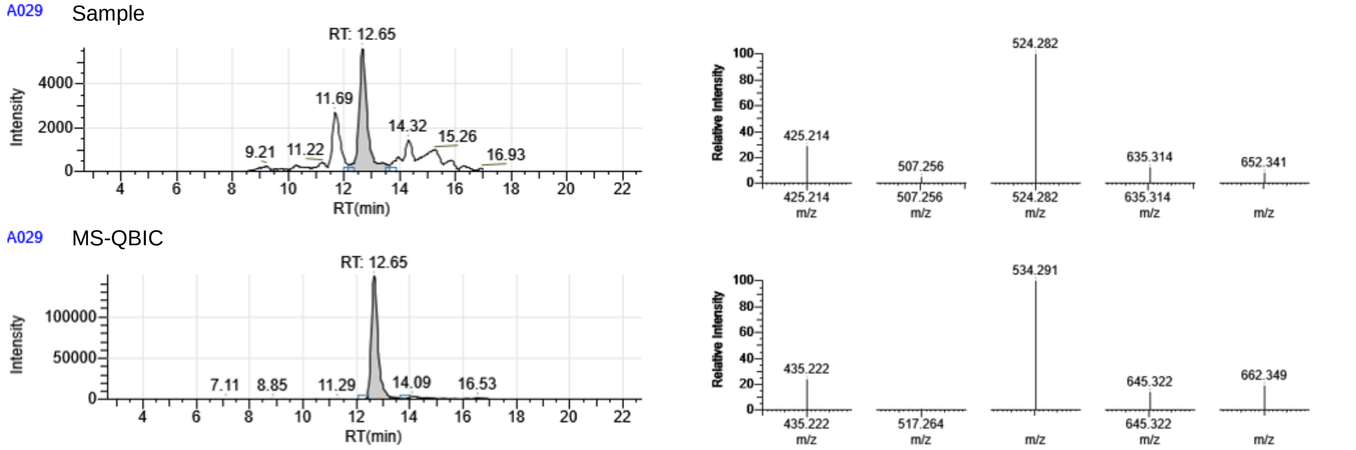

P12

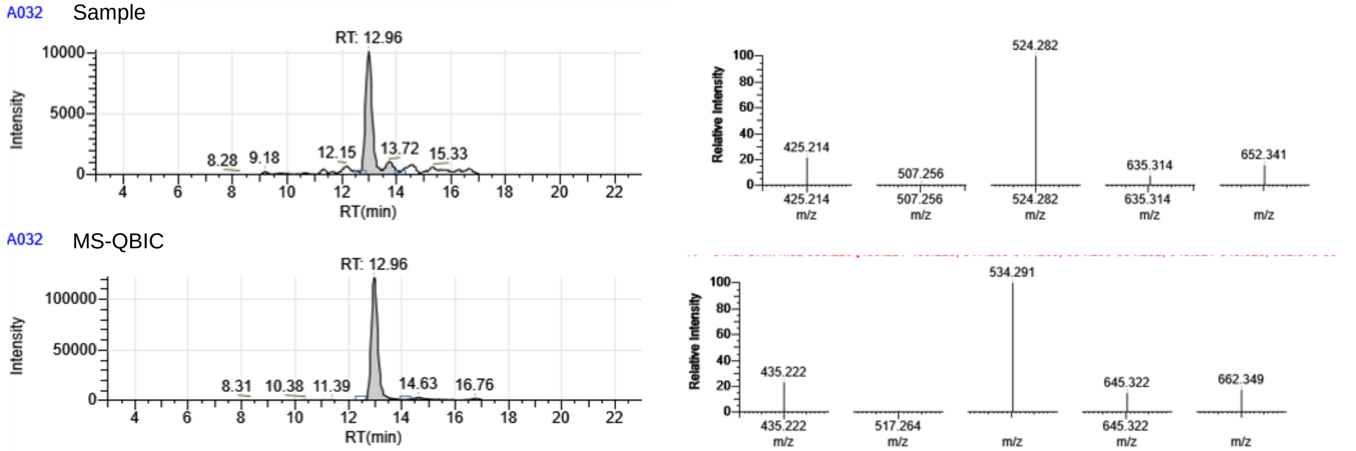

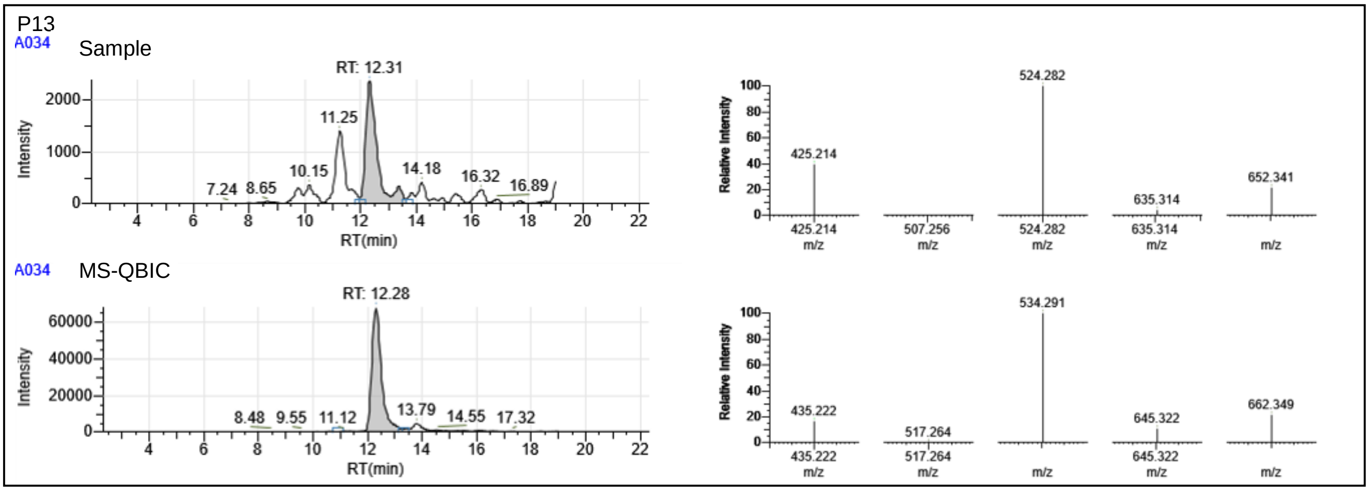
P13
A034 Sample
RT: 12.31
11.25
10.15
14.18
16.32
7.24 8.65
16.89
Intensity
RT(min)
Relative Intensity
425.214
524.282
635.314
652.341
507.256
m/z
A034 MS-QBIC
RT: 12.28
8.48 9.55 11.12
13.79
14.55 17.32
435.222
534.291
517.264
645.322
662.349

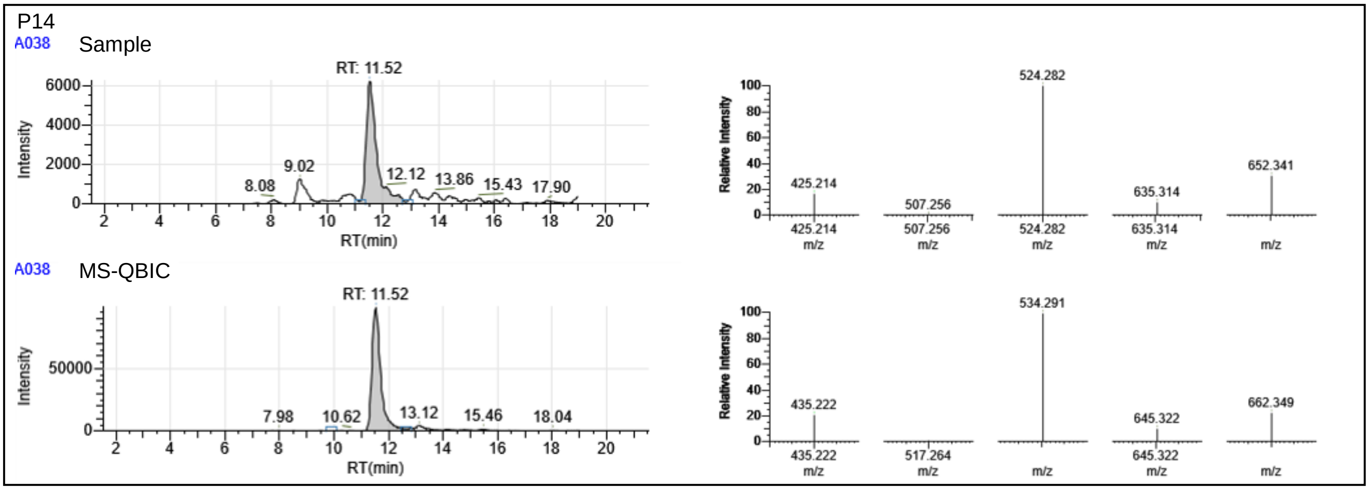
P14
A038 Sample
RT: 11.52
9.02
8.08
12.12
13.86
15.43
17.90
Intensity
RT(min)
Relative Intensity
425.214
507.256
524.282
635.314
652.341
m/z
A038 MS-QBIC
RT: 11.52
7.98
10.62
13.12
15.46
18.04
435.222
517.264
534.291
645.322
662.349

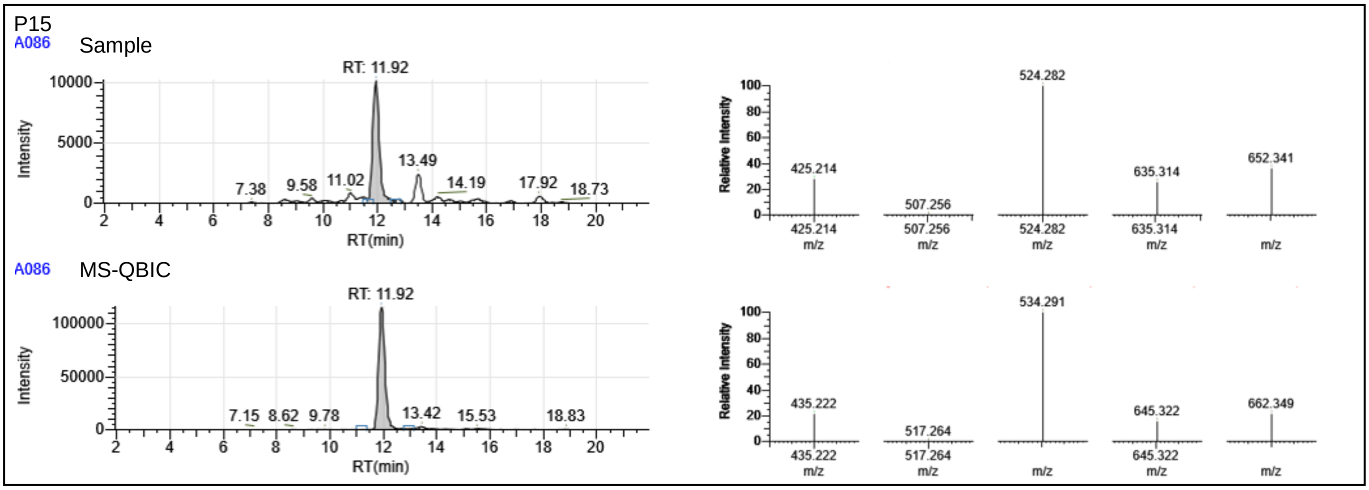
P15
A086 Sample
RT: 11.92
13.49
7.38
9.58
11.02
14.19
17.92
18.73
Intensity
RT(min)
Relative Intensity
425.214
507.256
524.282
635.314
652.341
m/z
A086 MS-QBIC
RT: 11.92
7.15 8.62 9.78
13.42
15.53
18.83
435.222
517.264
534.291
645.322
662.349

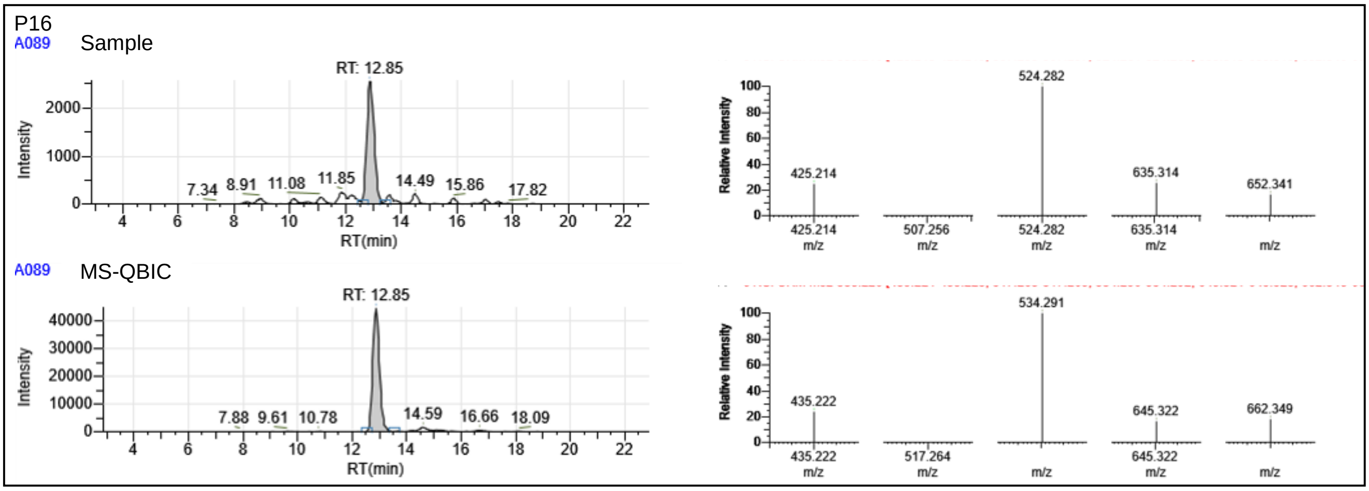
P16
A089 Sample
RT: 12.85
7.34
8.91
11.08
11.85
14.49
15.86
17.82
Intensity
RT(min)
Relative Intensity
425.214
507.256
524.282
635.314
652.341
m/z
A089 MS-QBIC
RT: 12.85
7.88 9.61 10.78
14.59
16.66 18.09
435.222
517.264
534.291
645.322
662.349

P17

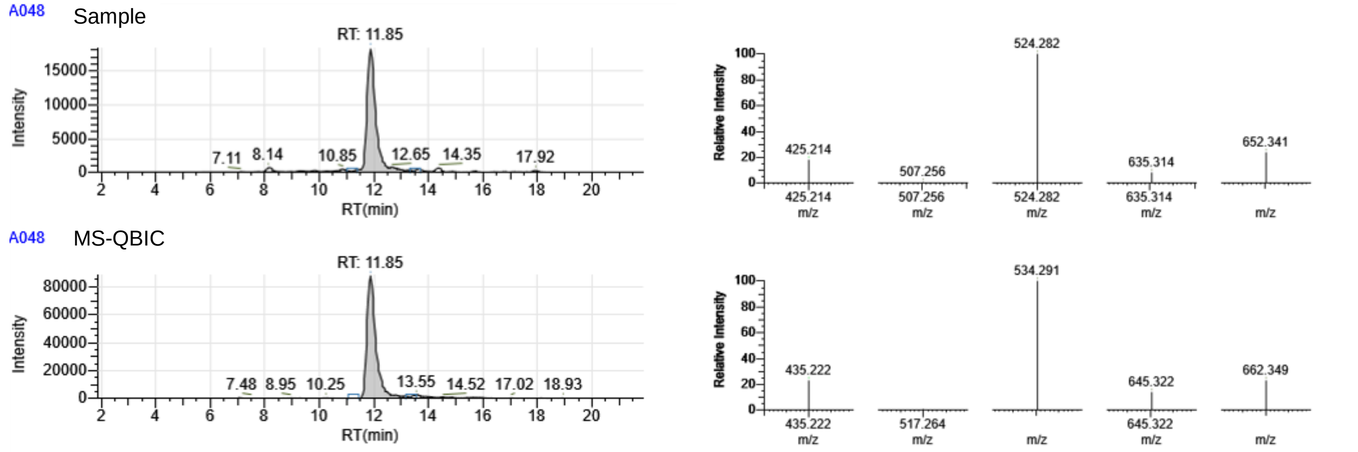

P18

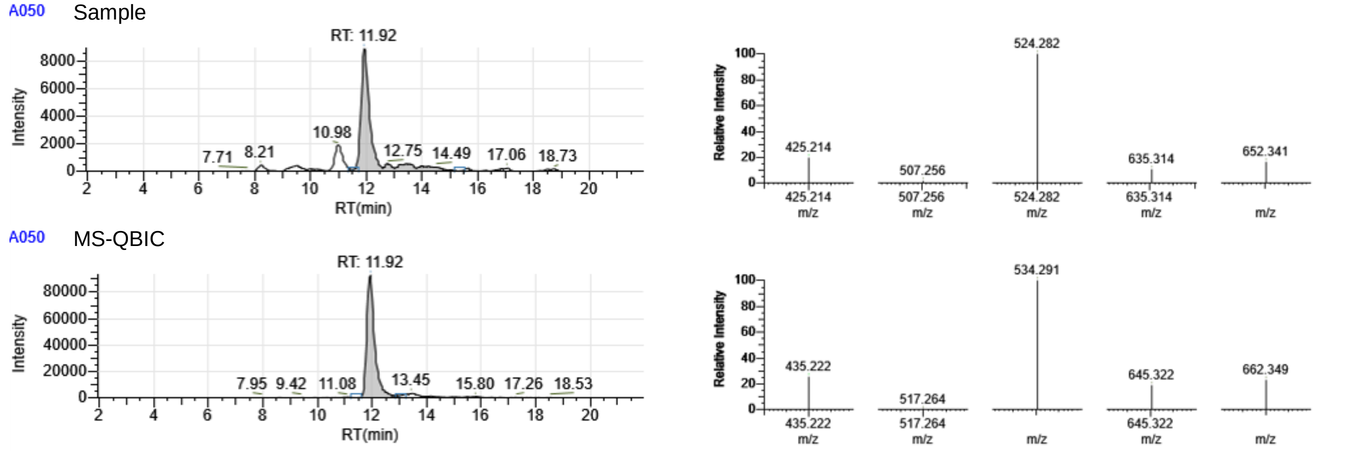

P19

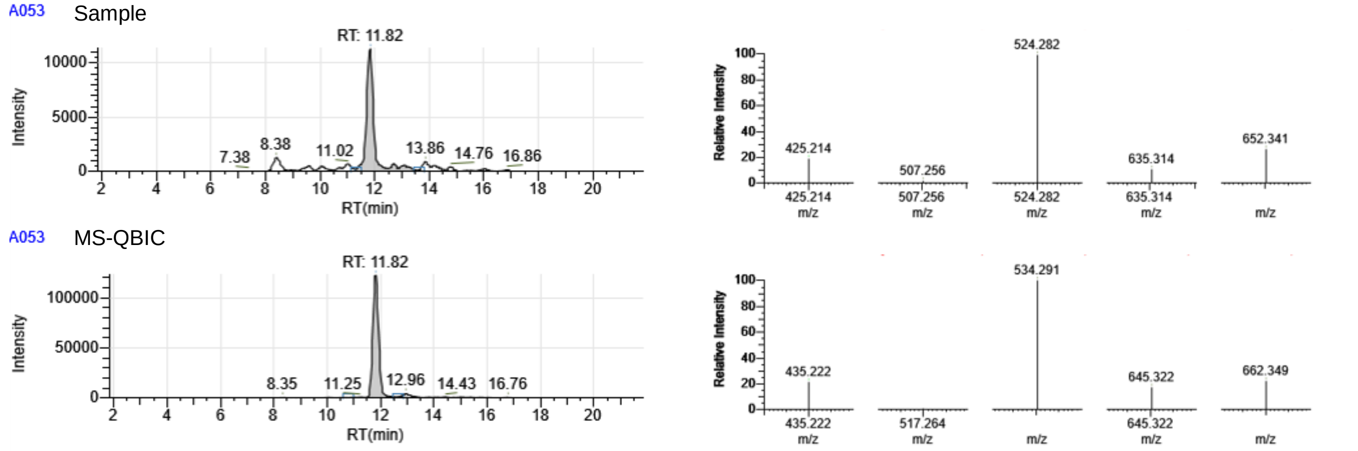

P20

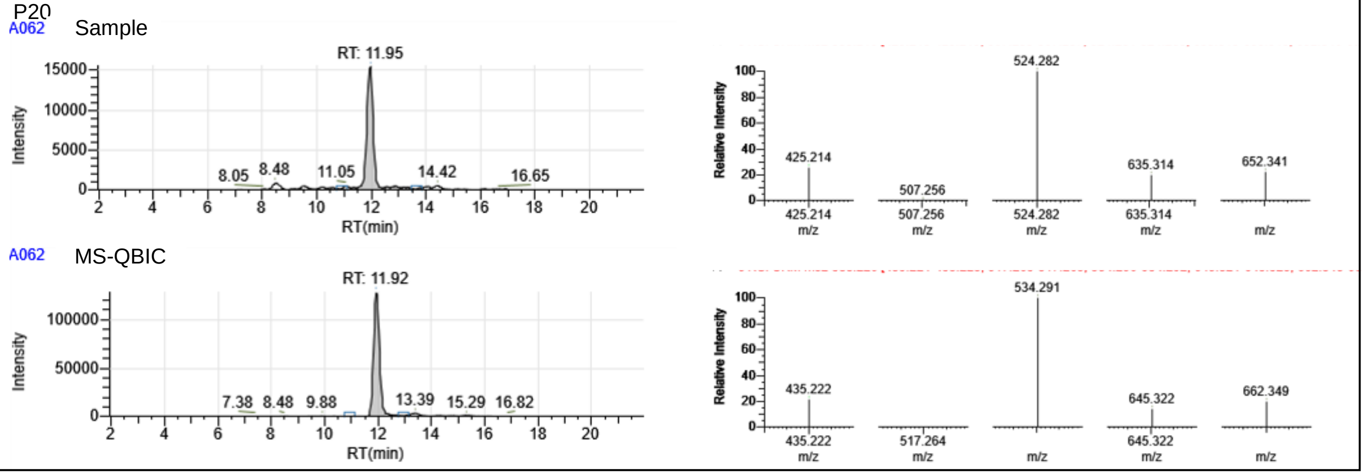

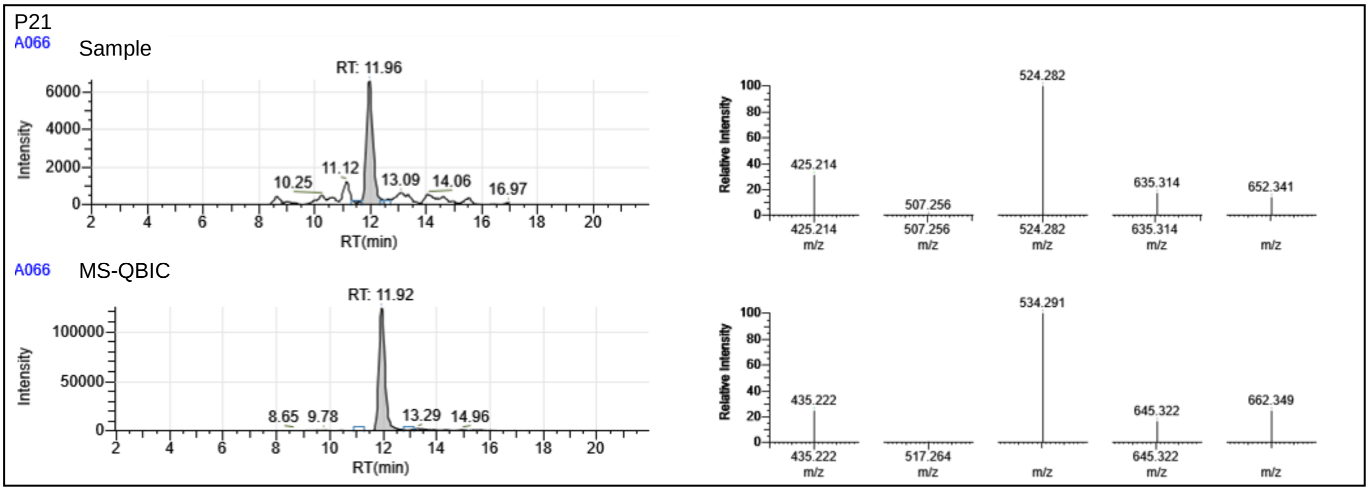
P21
A066 Sample
RT: 11.96
10.25
11.12
13.09
14.06
16.97
Intensity
RT(min)
Relative Intensity
425.214
507.256
524.282
635.314
652.341
m/z
A066 MS-QBIC
RT: 11.92
8.65 9.78
13.29 14.96
435.222
517.264
534.291
645.322
662.349

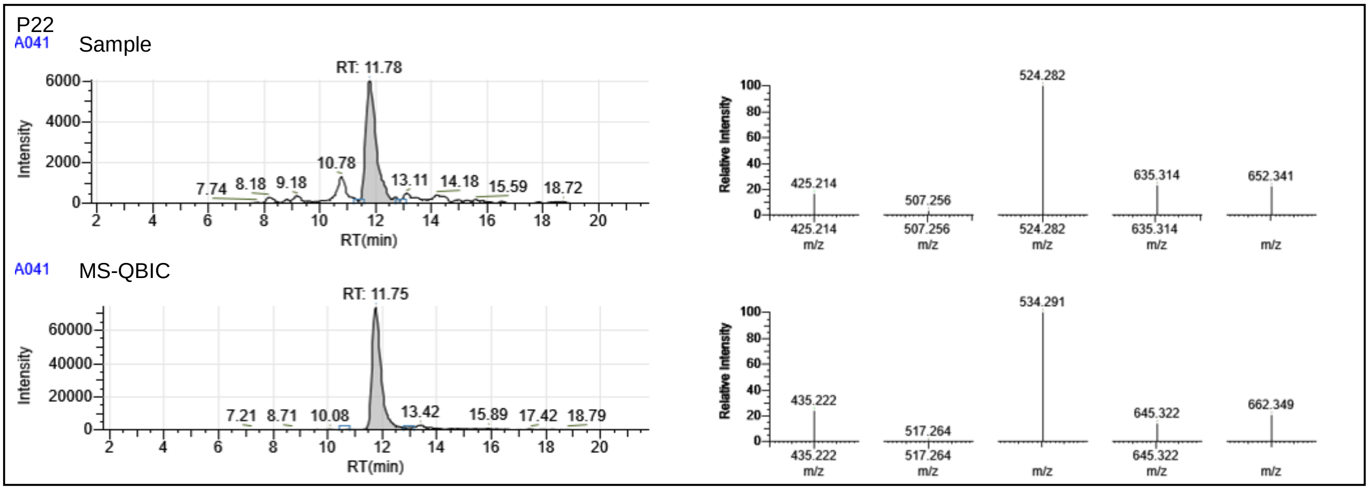
P22
A041 Sample
RT: 11.78
7.74 8.18 9.18
10.78
13.11 14.18 15.59 18.72
Intensity
RT(min)
Relative Intensity
425.214
507.256
524.282
635.314
652.341
m/z
A041 MS-QBIC
RT: 11.75
7.21 8.71 10.08
13.42
15.89 17.42 18.79
435.222
517.264
534.291
645.322
662.349

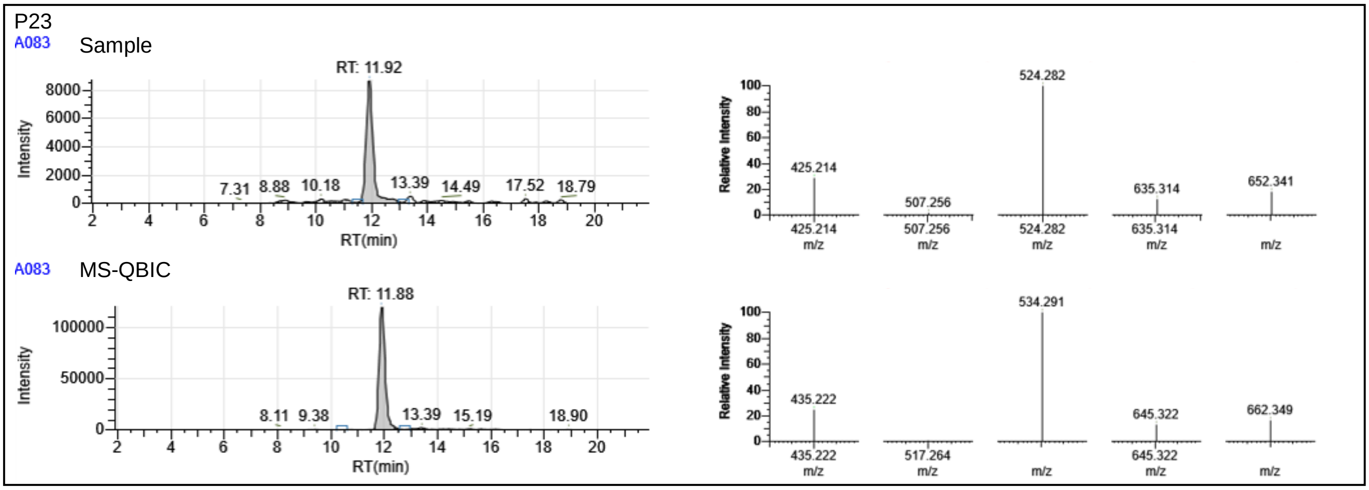
P23
A083 Sample
RT: 11.92
7.31 8.88 10.18
13.39 14.49
17.52 18.79
Intensity
RT(min)
Relative Intensity
425.214
507.256
524.282
635.314
652.341
m/z
A083 MS-QBIC
RT: 11.88
8.11 9.38
13.39 15.19
18.90
435.222
517.264
534.291
645.322
662.349

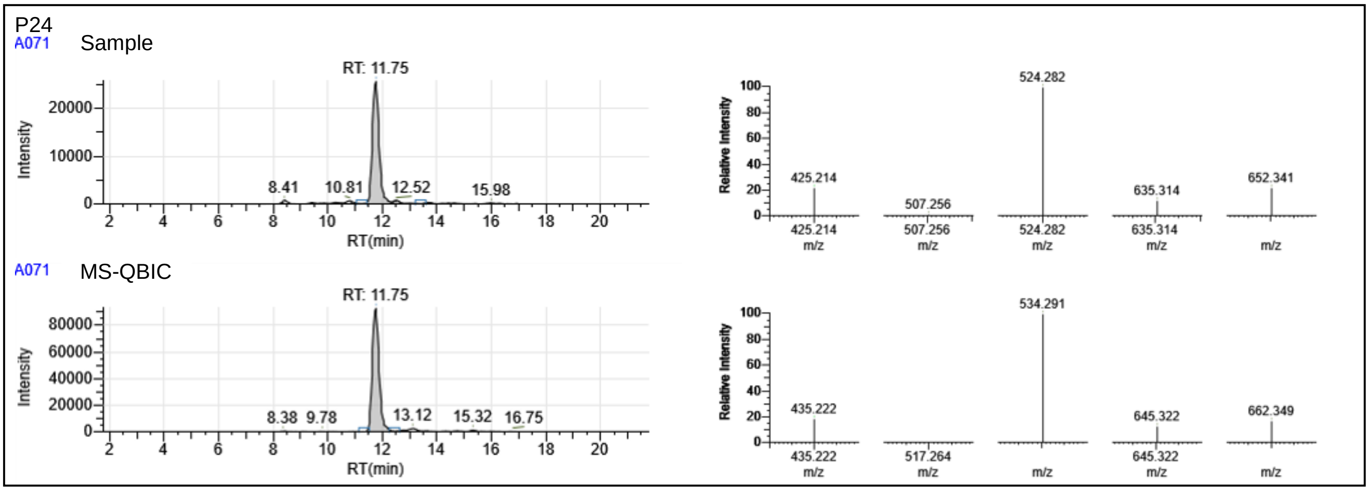
P24
A071 Sample
RT: 11.75
8.41
10.81
12.52
15.98
Intensity
RT(min)
Relative Intensity
425.214
507.256
524.282
635.314
652.341
m/z
A071 MS-QBIC
RT: 11.75
8.38 9.78
13.12
15.32 16.75
435.222
517.264
534.291
645.322
662.349

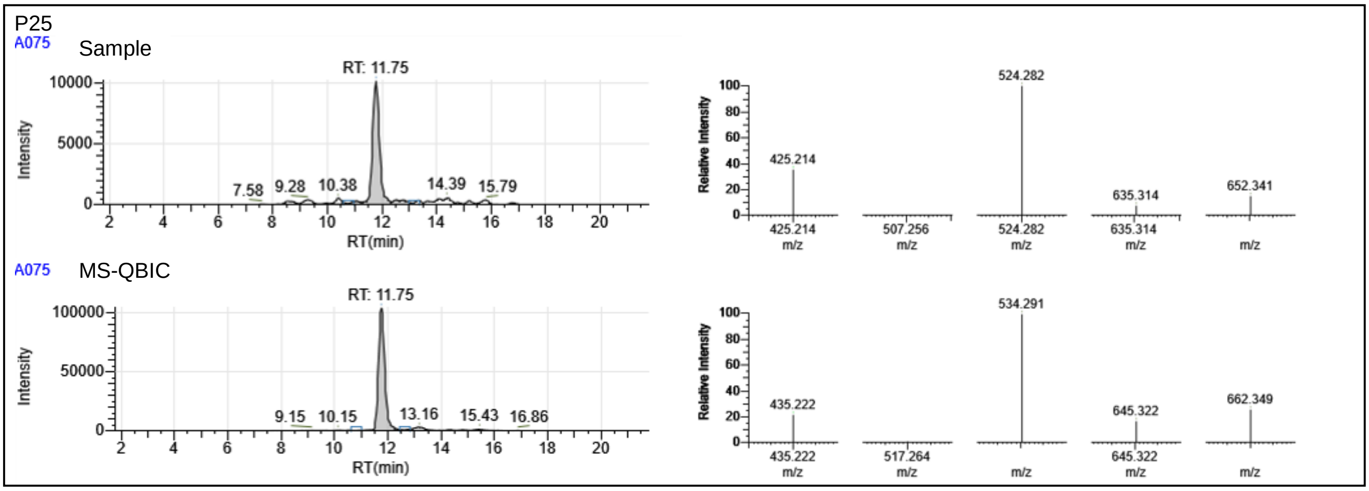
P25
A075 Sample
RT: 11.75
Intensity
RT(min)
7.58 9.28 10.38 14.39 15.79
Relative Intensity
524.282
425.214
635.314
652.341
425.214 507.256 524.282 635.314
m/z
A075 MS-QBIC
RT: 11.75
9.15 10.15 13.16 15.43 16.86
534.291
435.222
645.322
662.349
435.222 517.264 645.322

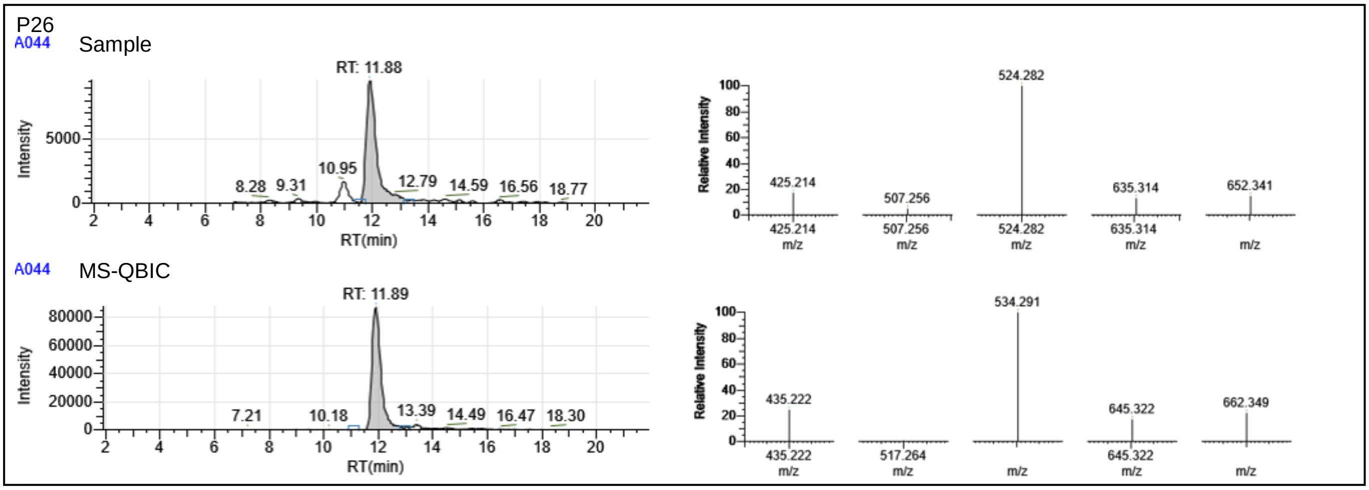
P26
A044 Sample
RT: 11.88
Intensity
RT(min)
8.28 9.31 10.95 12.79 14.59 16.56 18.77
Relative Intensity
524.282
425.214
507.256
635.314
652.341
m/z
A044 MS-QBIC
RT: 11.89
7.21 10.18 13.39 14.49 16.47 18.30
534.291
435.222
645.322
662.349
435.222 517.264 645.322

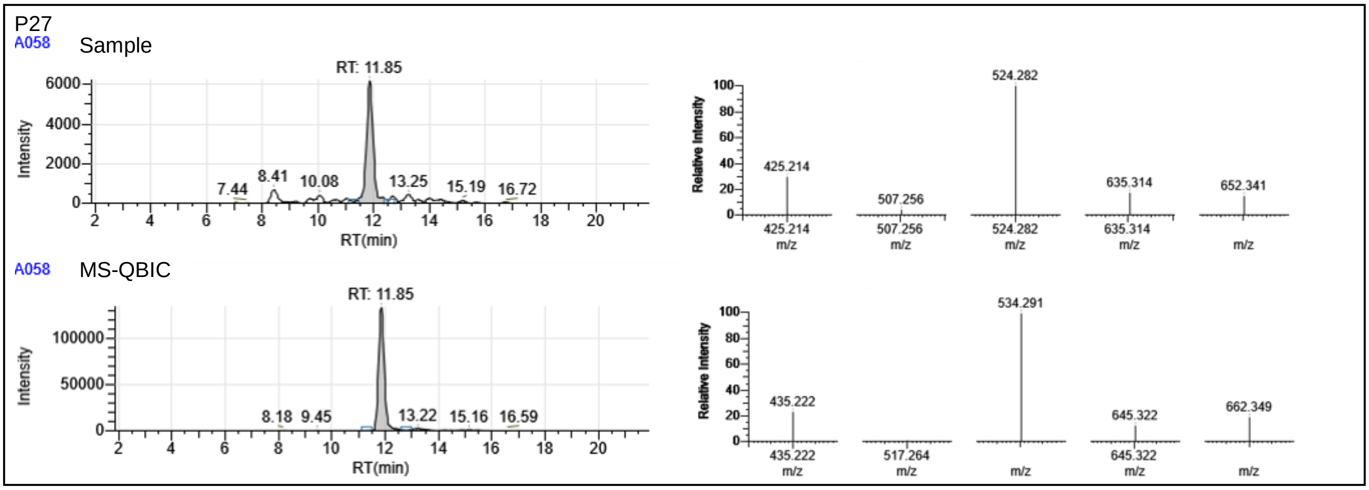
P27
A058 Sample
RT: 11.85
Intensity
RT(min)
7.44 8.41 10.08 13.25 15.19 16.72
Relative Intensity
524.282
425.214
507.256
635.314
652.341
m/z
A058 MS-QBIC
RT: 11.85
8.18 9.45 13.22 15.16 16.59
534.291
435.222
645.322
662.349
435.222 517.264 645.322

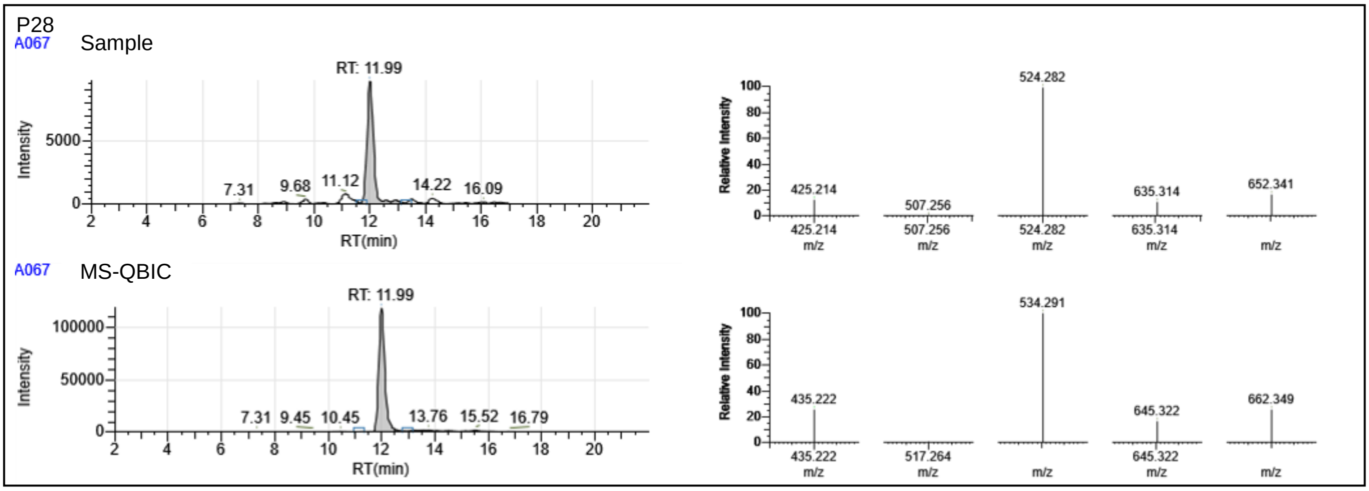
P28
A067 Sample
RT: 11.99
Intensity
RT(min)
7.31 9.68 11.12 14.22 16.09
Relative Intensity
524.282
425.214
507.256
635.314
652.341
m/z
A067 MS-QBIC
RT: 11.99
7.31 9.45 10.45 13.76 15.52 16.79
534.291
435.222
645.322
662.349
435.222 517.264 645.322

P29

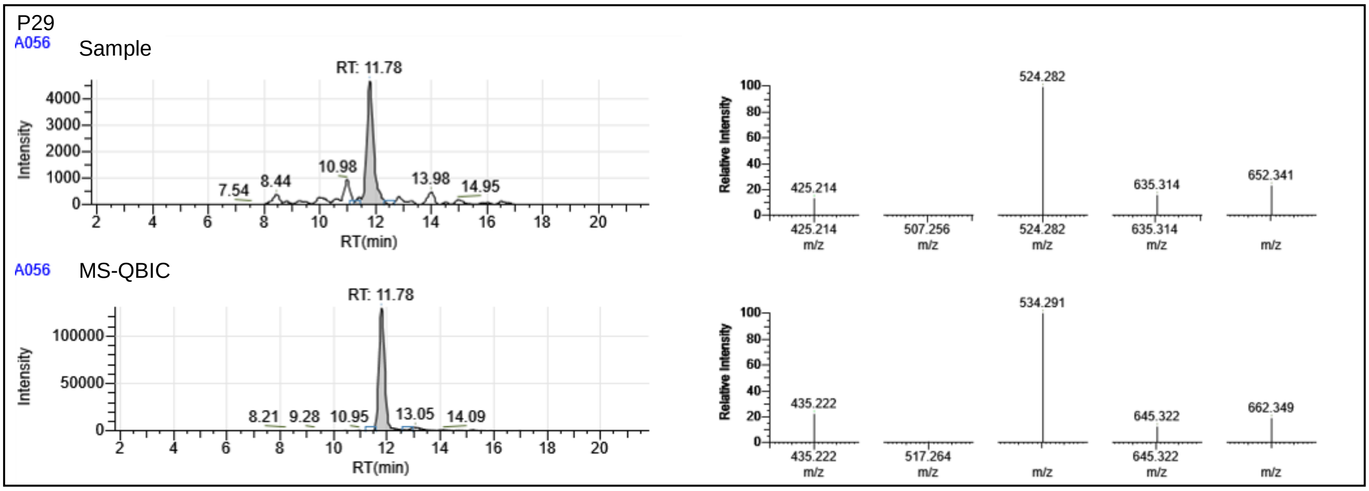
A056 Sample
RT: 11.78
Intensity
RT(min)
7.54
8.44
10.98
13.98
14.95
Relative Intensity
524.282
425.214
635.314
652.341
507.256
m/z
A056 MS-QBIC
RT: 11.78
8.21
9.28
10.95
13.05
14.09
534.291
435.222
645.322
662.349
517.264

P30

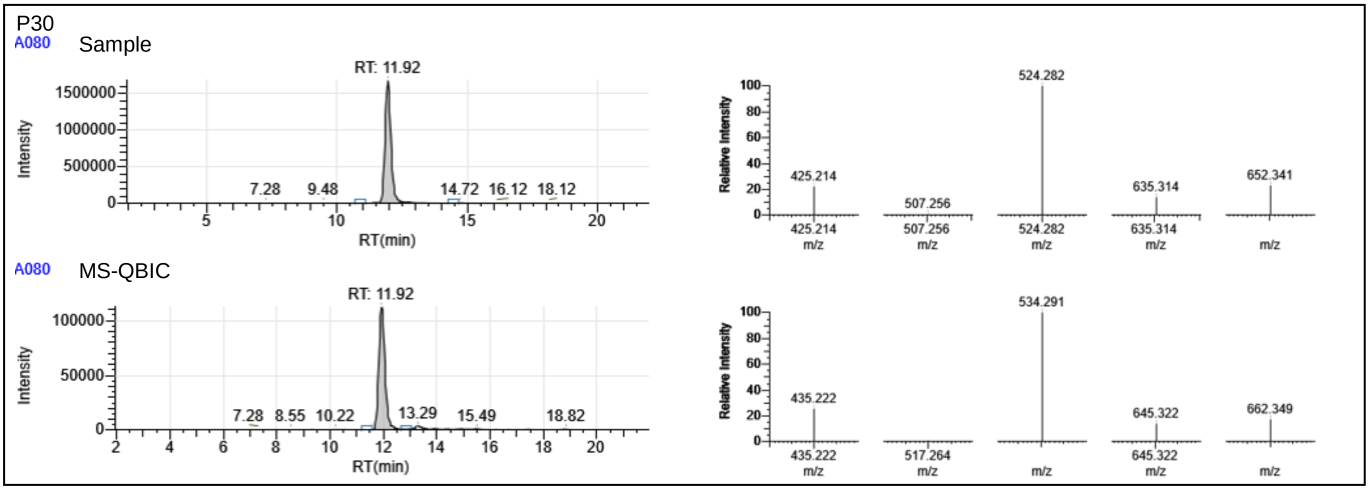
A080 Sample
RT: 11.92
Intensity
RT(min)
7.28
9.48
14.72
16.12
18.12
Relative Intensity
524.282
425.214
507.256
635.314
652.341
m/z
A080 MS-QBIC
RT: 11.92
7.28
8.55
10.22
13.29
15.49
18.82
534.291
435.222
645.322
662.349
517.264

F Apo A1 (DLATVYVDVLK) Heart

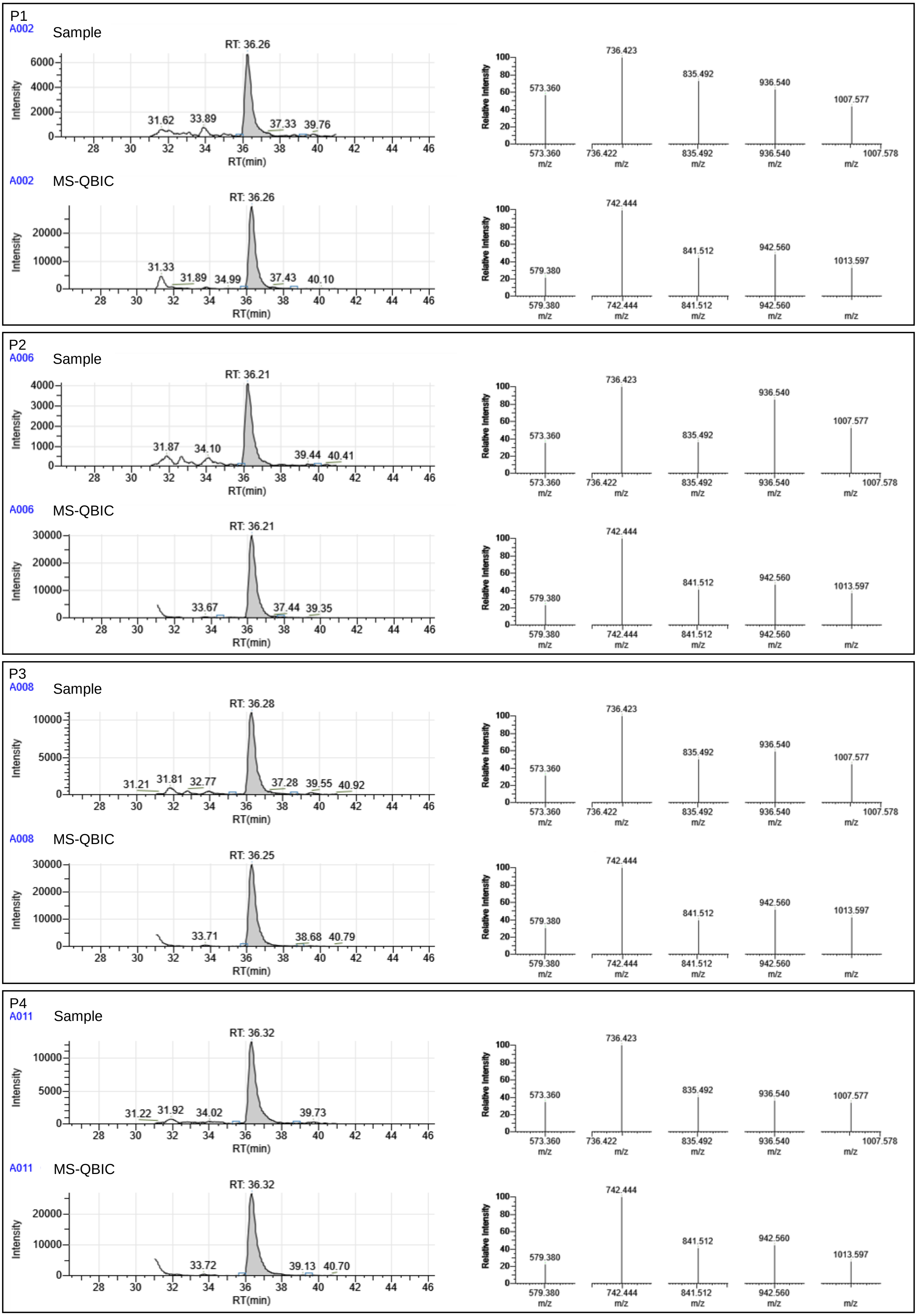

P6

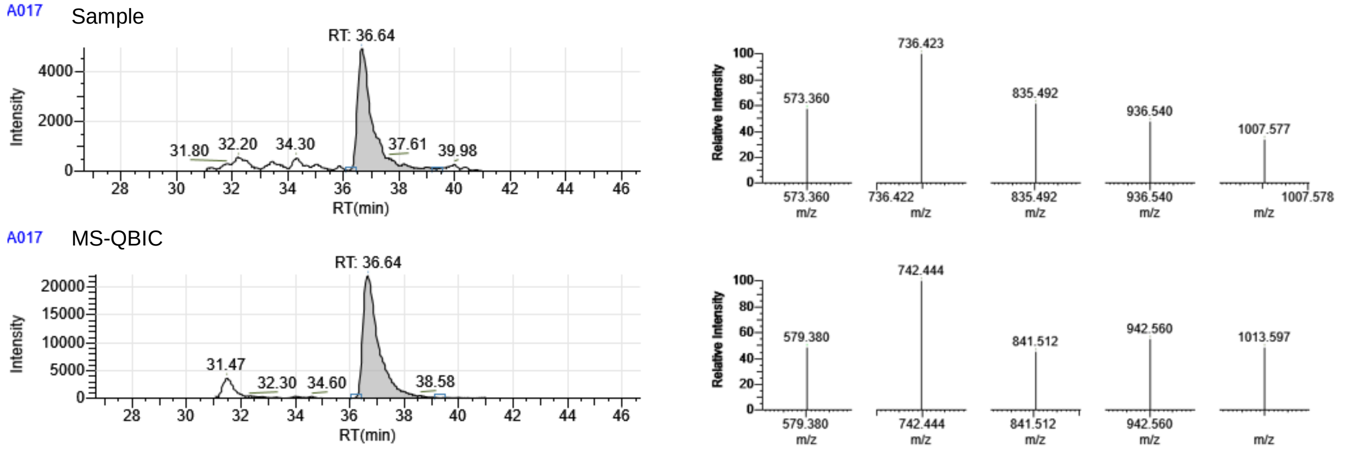
A017 Sample
RT: 36.64
31.80 32.20 34.30 37.61 39.98
Intensity
RT(min)
573.360
736.423
835.492
936.540
1007.577
Relative Intensity
573.360 736.422 835.492 936.540 1007.578
m/z
A017 MS-QBIC
RT: 36.64
31.47 32.30 34.60 38.58
579.380
742.444
841.512
942.560
1013.597

P7

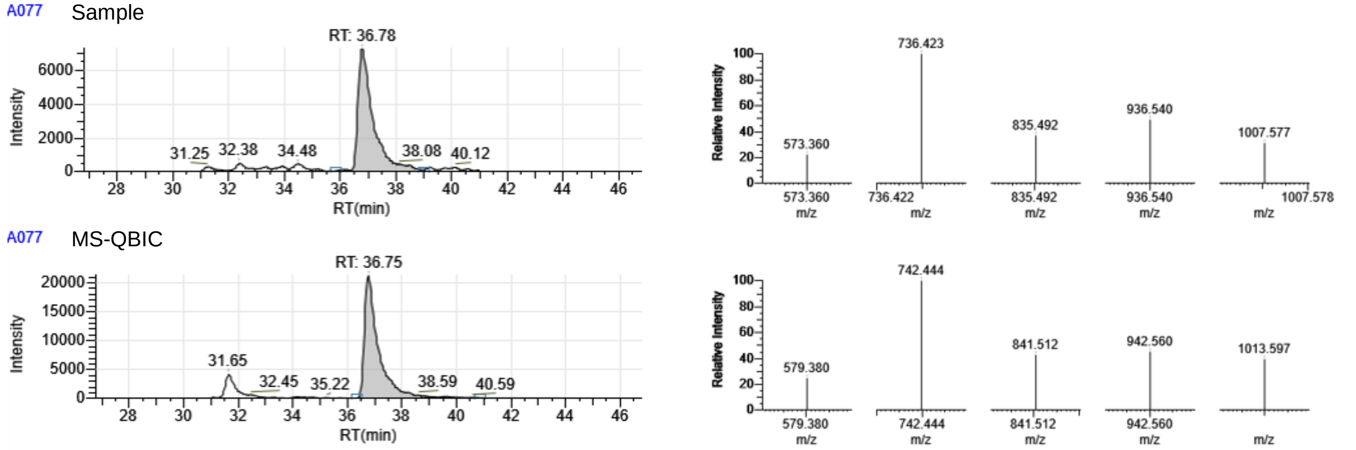
A077 Sample
RT: 36.78
31.25 32.38 34.48 38.08 40.12
Intensity
RT(min)
573.360
736.423
835.492
936.540
1007.577
Relative Intensity
573.360 736.422 835.492 936.540 1007.578
m/z
A077 MS-QBIC
RT: 36.75
31.65 32.45 35.22 38.59 40.59
579.380
742.444
841.512
942.560
1013.597

P8

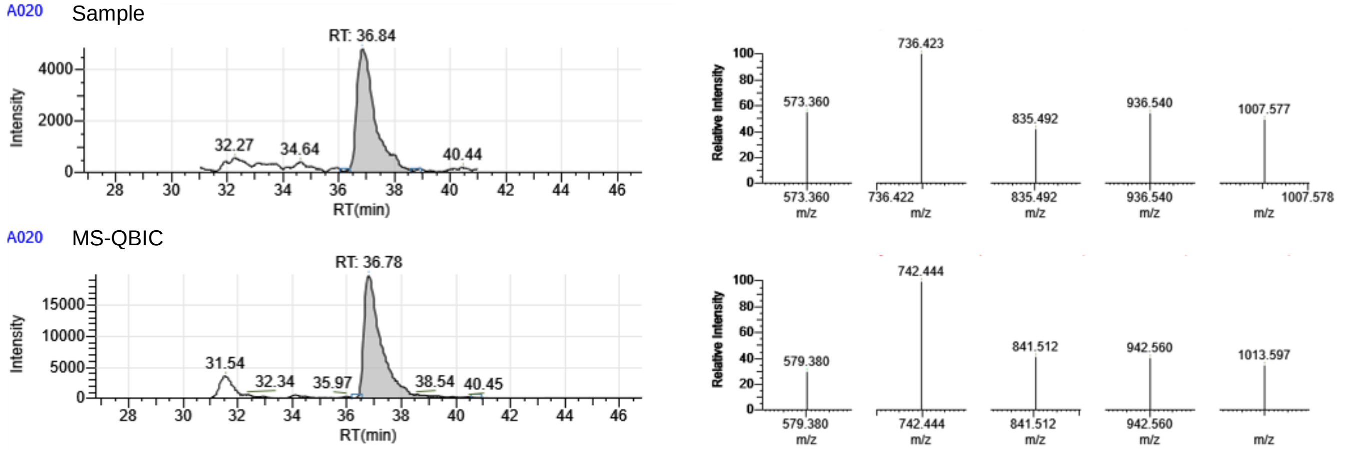
A020 Sample
RT: 36.84
32.27 34.64 40.44
Intensity
RT(min)
573.360
736.423
835.492
936.540
1007.577
Relative Intensity
573.360 736.422 835.492 936.540 1007.578
m/z
A020 MS-QBIC
RT: 36.78
31.54 32.34 35.97 38.54 40.45
579.380
742.444
841.512
942.560
1013.597

P9

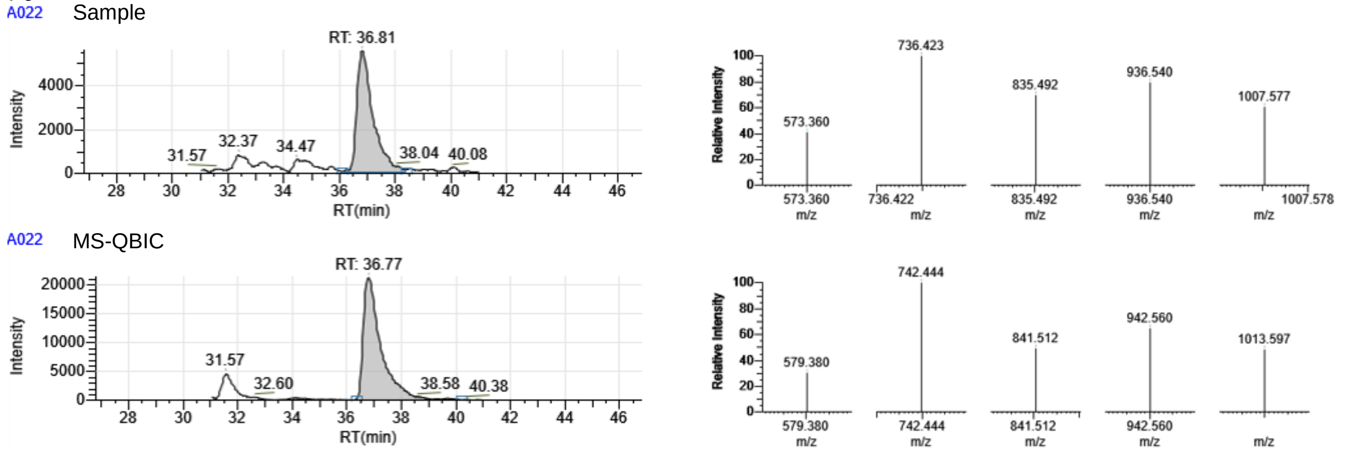
A022 Sample
RT: 36.81
31.57 32.37 34.47 38.04 40.08
Intensity
RT(min)
573.360
736.423
835.492
936.540
1007.577
Relative Intensity
573.360 736.422 835.492 936.540 1007.578
m/z
A022 MS-QBIC
RT: 36.77
31.57 32.60 38.58 40.38
579.380
742.444
841.512
942.560
1013.597

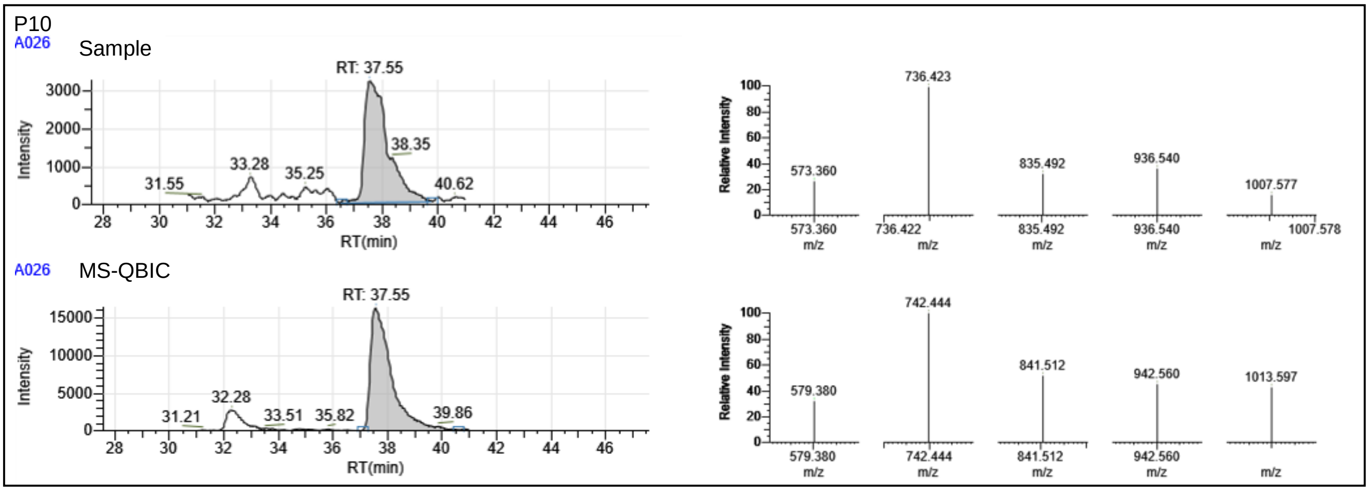
P10
A026 Sample
RT: 37.55
31.55
33.28
35.25
38.35
40.62
Intensity
RT(min)
573.360
736.423
835.492
936.540
1007.577
Relative Intensity
m/z
A026 MS-QBIC
RT: 37.55
31.21
32.28
33.51
35.82
39.86
579.380
742.444
841.512
942.560
1013.597

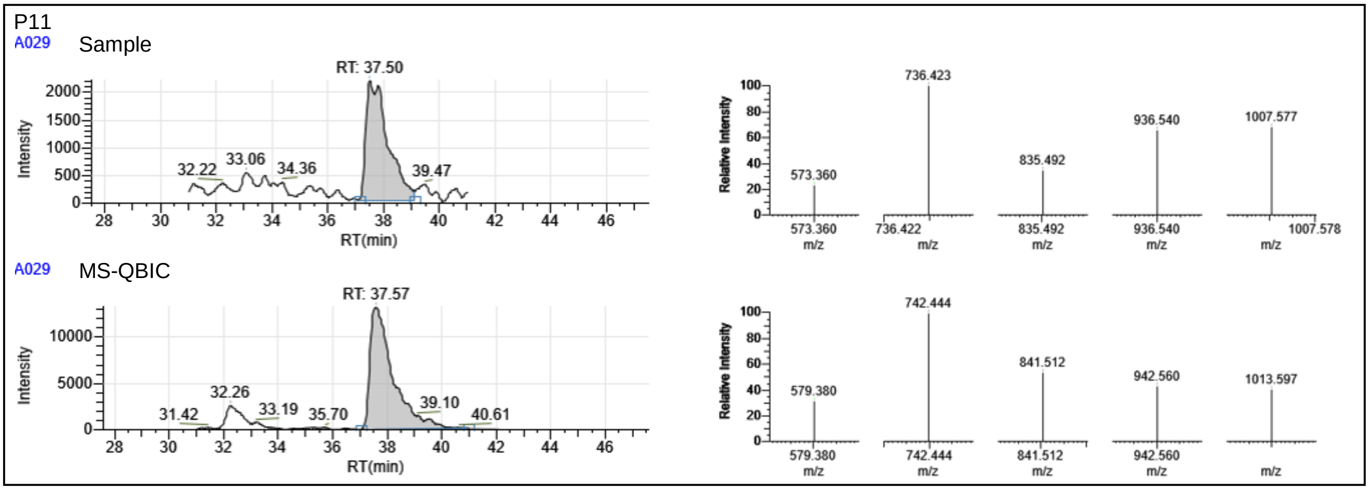
P11
A029 Sample
RT: 37.50
32.22
33.06
34.36
39.47
Intensity
RT(min)
573.360
736.423
835.492
936.540
1007.577
Relative Intensity
m/z
A029 MS-QBIC
RT: 37.57
31.42
32.26
33.19
35.70
39.10
40.61
579.380
742.444
841.512
942.560
1013.597

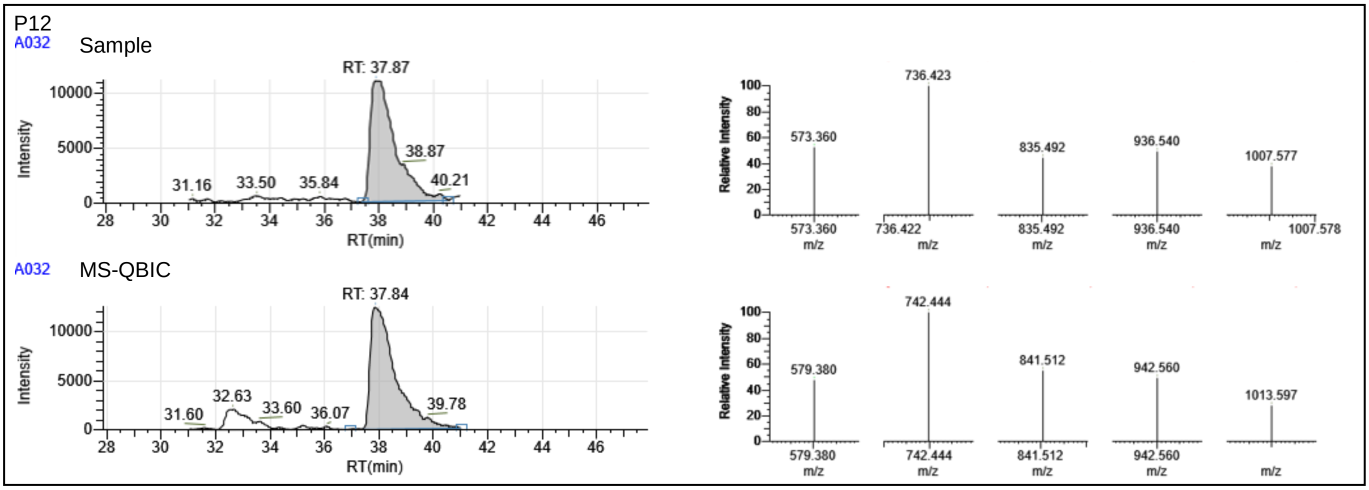
P12
A032 Sample
RT: 37.87
31.16
33.50
35.84
38.87
40.21
Intensity
RT(min)
573.360
736.423
835.492
936.540
1007.577
Relative Intensity
m/z
A032 MS-QBIC
RT: 37.84
31.60
32.63
33.60
36.07
39.78
579.380
742.444
841.512
942.560
1013.597

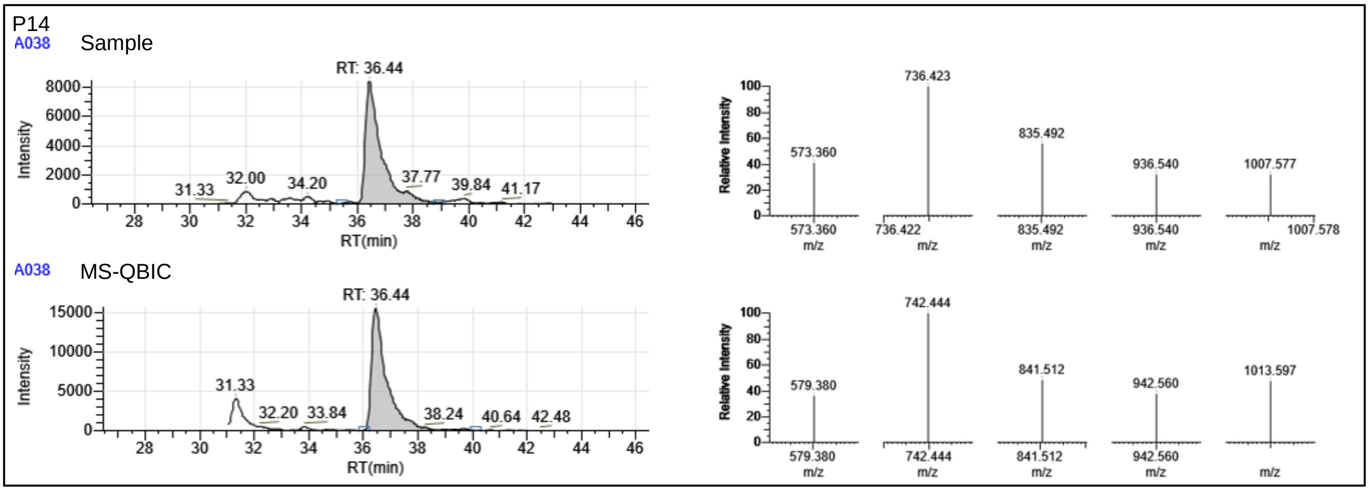
P14
A038 Sample
RT: 36.44
31.33
32.00
34.20
37.77
39.84
41.17
Intensity
RT(min)
573.360
736.423
835.492
936.540
1007.577
Relative Intensity
m/z
A038 MS-QBIC
RT: 36.44
31.33
32.20
33.84
38.24
40.64
42.48
579.380
742.444
841.512
942.560
1013.597

P15

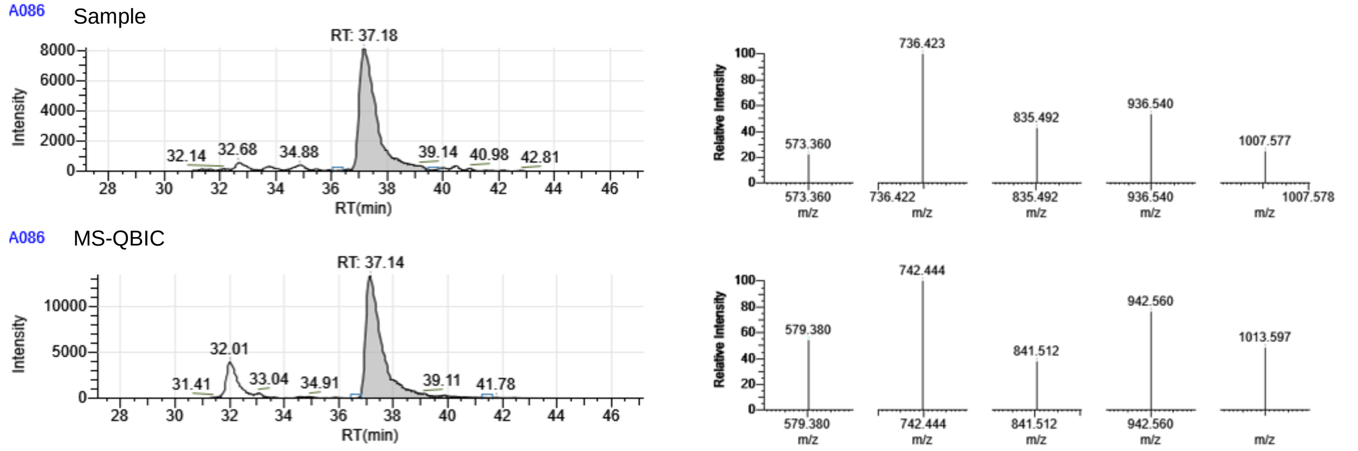

P17

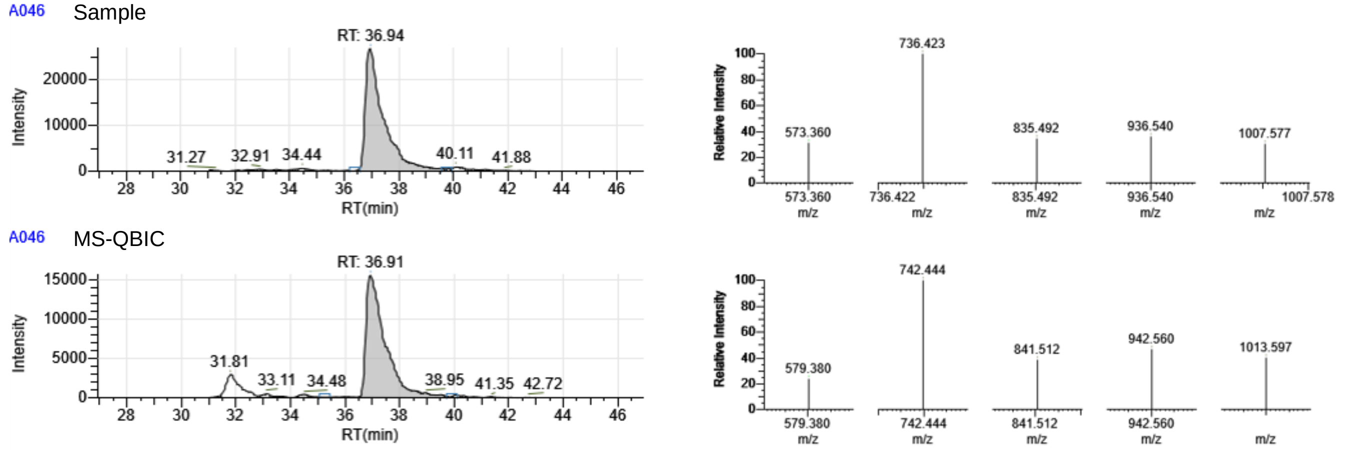

P18

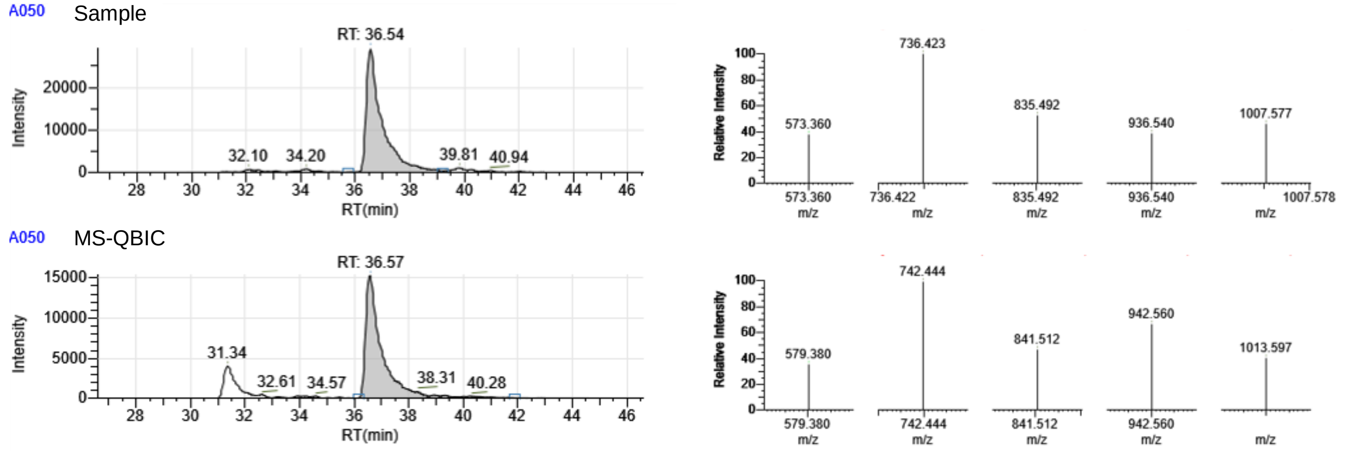

P19

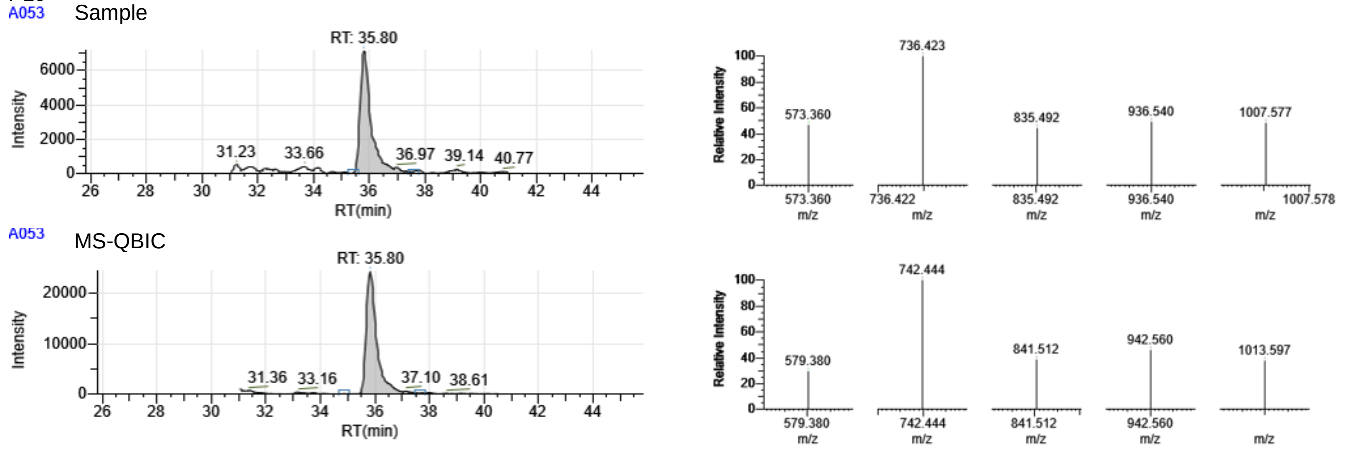

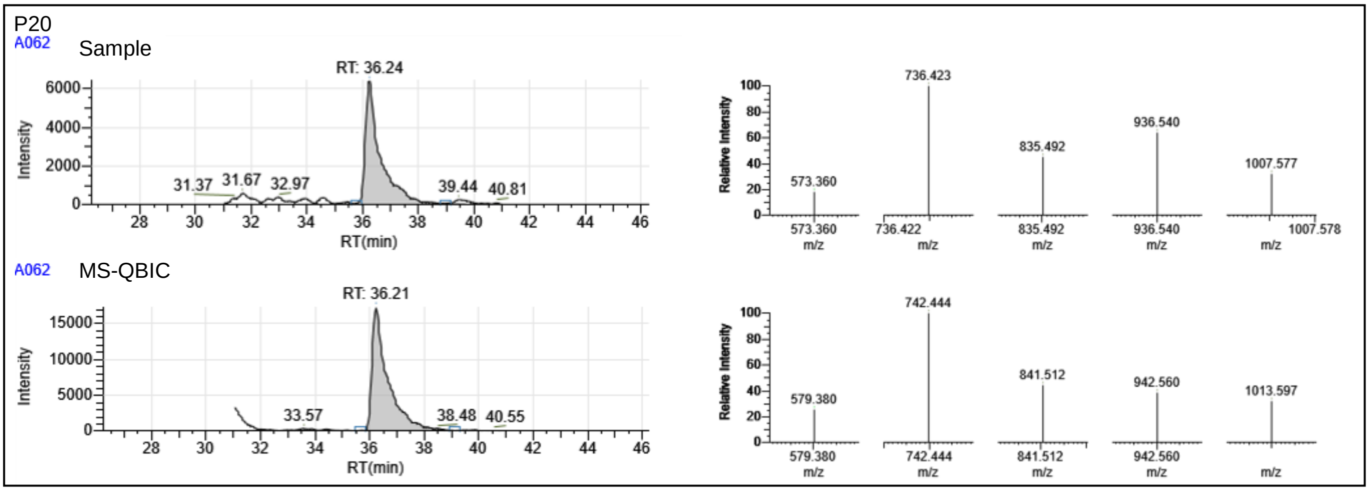
P20
A062 Sample
RT: 36.24
31.37 31.67 32.97 39.44 40.81
Intensity
RT(min)
Relative Intensity
736.423
573.360
835.492
936.540
1007.577
736.422
1007.578
m/z
A062 MS-QBIC
RT: 36.21
33.57 38.48 40.55
742.444
579.380
841.512
942.560
1013.597

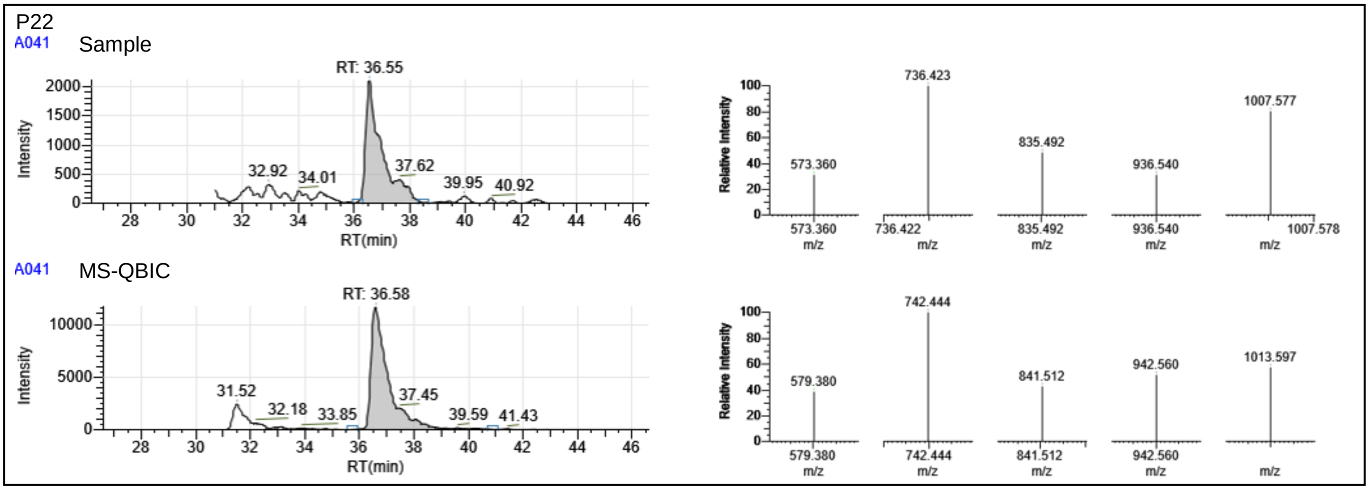
P22
A041 Sample
RT: 36.55
32.92 34.01 37.62 39.95 40.92
Intensity
RT(min)
Relative Intensity
736.423
1007.577
835.492
573.360
936.540
736.422
1007.578
m/z
A041 MS-QBIC
RT: 36.58
31.52 32.18 33.85 37.45 39.59 41.43
742.444
1013.597
942.560
579.380
841.512

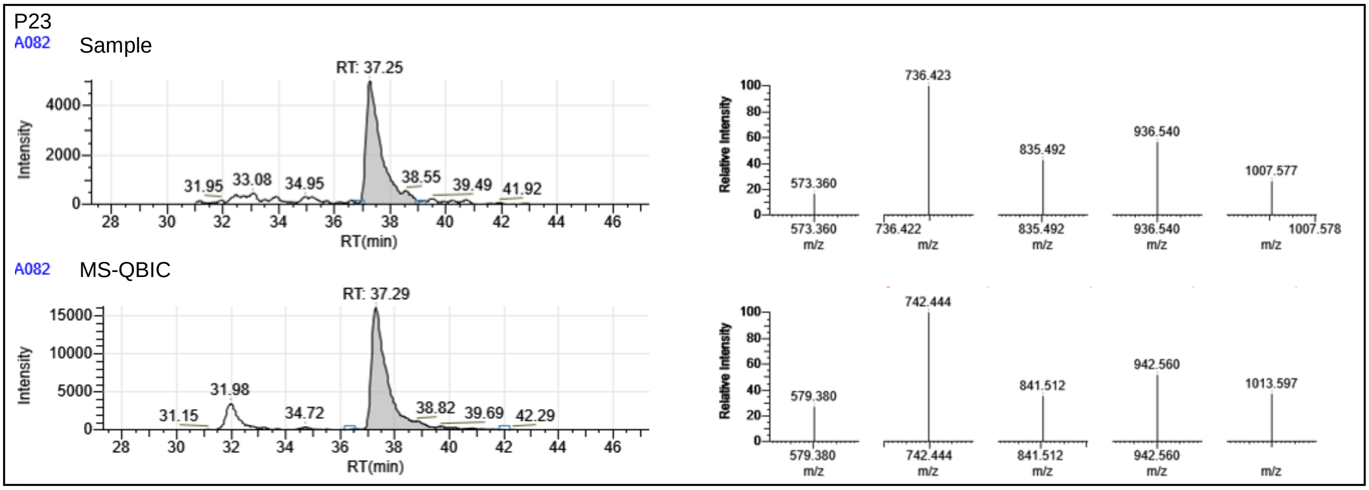
P23
A082 Sample
RT: 37.25
31.95 33.08 34.95 38.55 39.49 41.92
Intensity
RT(min)
Relative Intensity
736.423
936.540
835.492
1007.577
573.360
736.422
1007.578
m/z
A082 MS-QBIC
RT: 37.29
31.15 31.98 34.72 38.82 39.69 42.29
742.444
942.560
841.512
1013.597
579.380

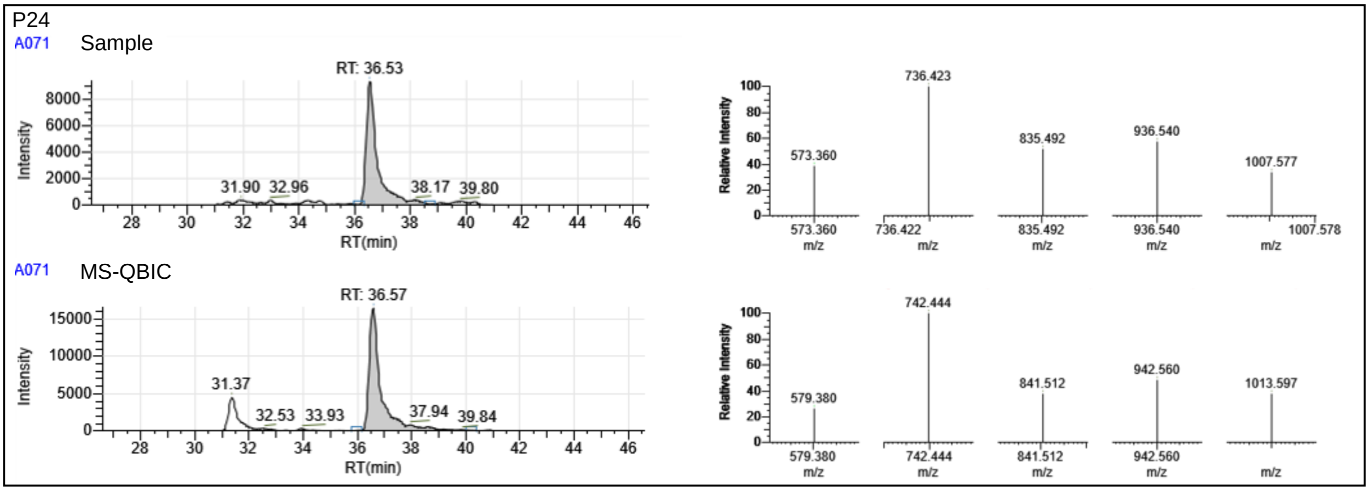
P24
A071 Sample
RT: 36.53
31.90 32.96 38.17 39.80
Intensity
RT(min)
Relative Intensity
736.423
936.540
835.492
573.360
1007.577
736.422
1007.578
m/z
A071 MS-QBIC
RT: 36.57
31.37 32.53 33.93 37.94 39.84
742.444
942.560
841.512
1013.597
579.380

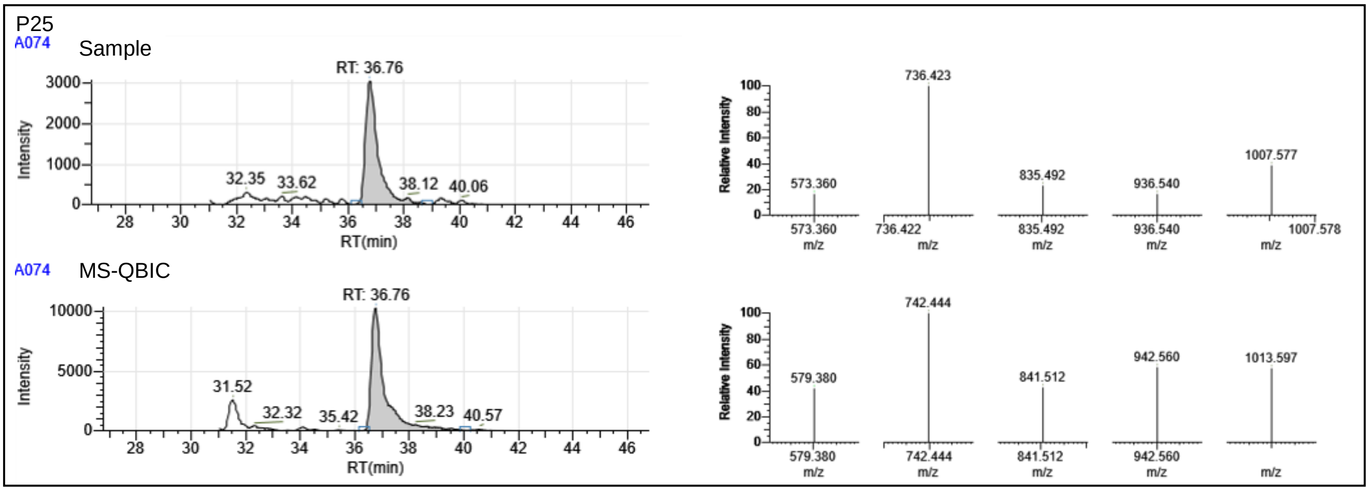
P25
A074 Sample
RT: 36.76
32.35
33.62
38.12
40.06
Intensity
RT(min)
736.423
573.360
835.492
936.540
1007.577
1007.578
736.422
Relative Intensity
m/z
A074 MS-QBIC
RT: 36.76
31.52
32.32
35.42
38.23
40.57
742.444
579.380
841.512
942.560
1013.597

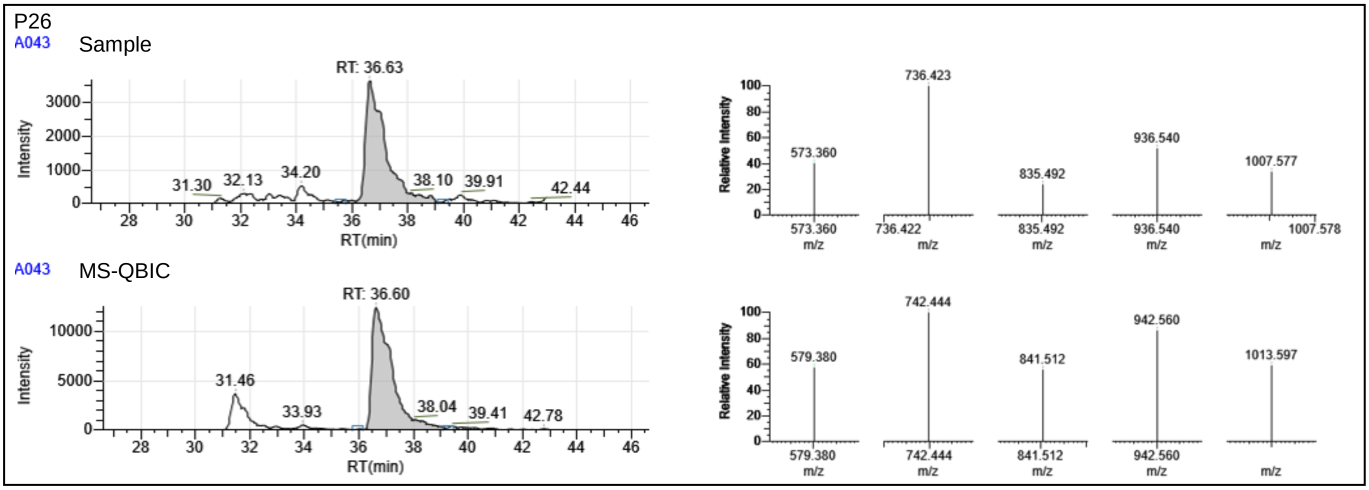
P26
A043 Sample
RT: 36.63
31.30
32.13
34.20
38.10
39.91
42.44
Intensity
RT(min)
736.423
573.360
835.492
936.540
1007.577
1007.578
736.422
Relative Intensity
m/z
A043 MS-QBIC
RT: 36.60
31.46
33.93
38.04
39.41
42.78
742.444
579.380
841.512
942.560
1013.597

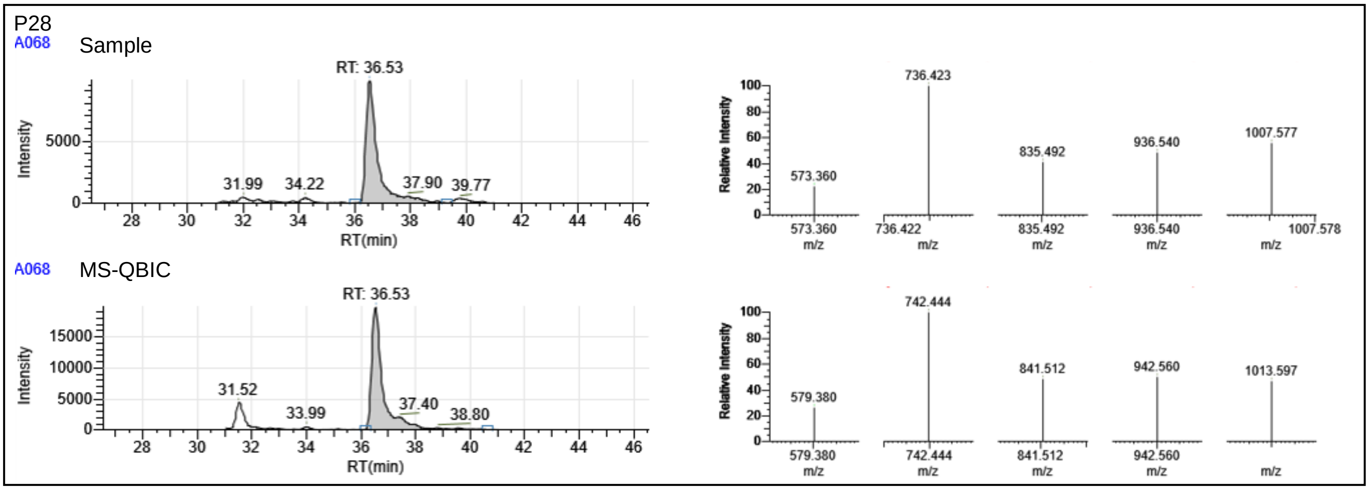
P28
A068 Sample
RT: 36.53
31.99
34.22
37.90
39.77
Intensity
RT(min)
736.423
573.360
835.492
936.540
1007.577
1007.578
736.422
Relative Intensity
m/z
A068 MS-QBIC
RT: 36.53
31.52
33.99
37.40
38.80
742.444
579.380
841.512
942.560
1013.597

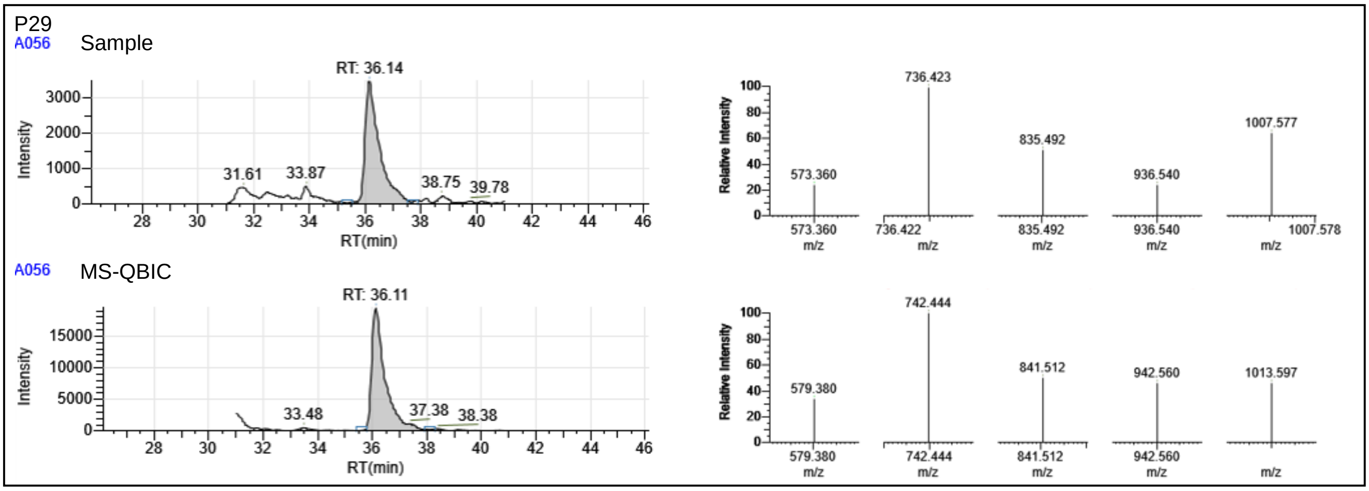
P29
A056 Sample
RT: 36.14
31.61
33.87
38.75
39.78
Intensity
RT(min)
736.423
573.360
835.492
936.540
1007.577
1007.578
736.422
Relative Intensity
m/z
A056 MS-QBIC
RT: 36.11
33.48
37.38
38.38
742.444
579.380
841.512
942.560
1013.597

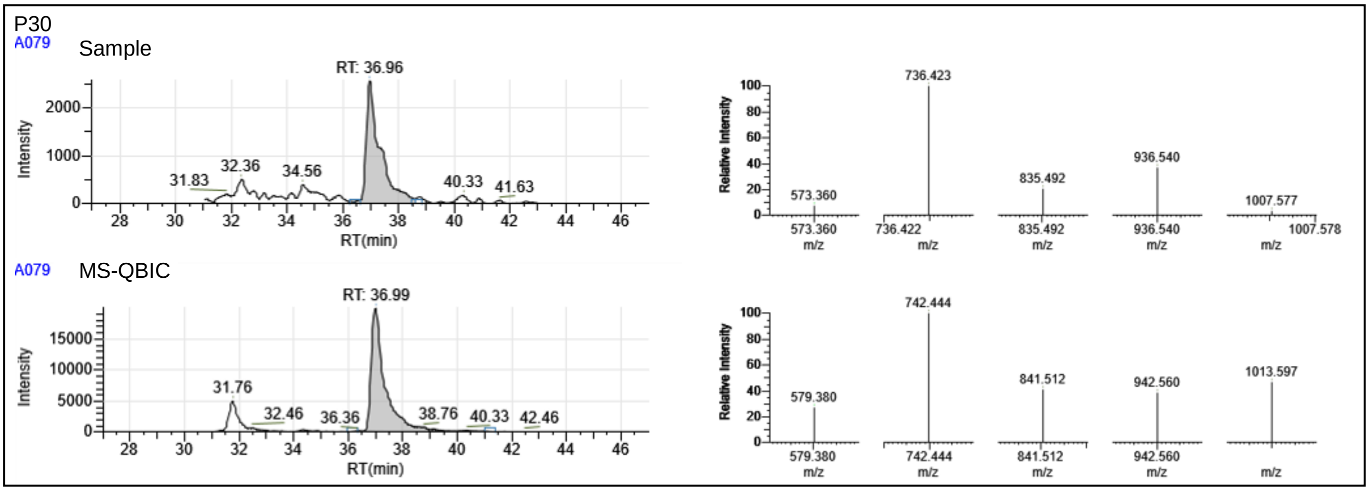
P30
A079
Sample
RT: 36.96
31.83
32.36
34.56
40.33
41.63
Intensity
RT(min)
Relative Intensity
736.423
573.360
835.492
936.540
1007.577
1007.578
736.422
m/z
A079
MS-QBIC
RT: 36.99
31.76
32.46
36.36
38.76
40.33
42.46
742.444
579.380
841.512
942.560
1013.597

G Apo A2 (EQLTPLIK) Heart

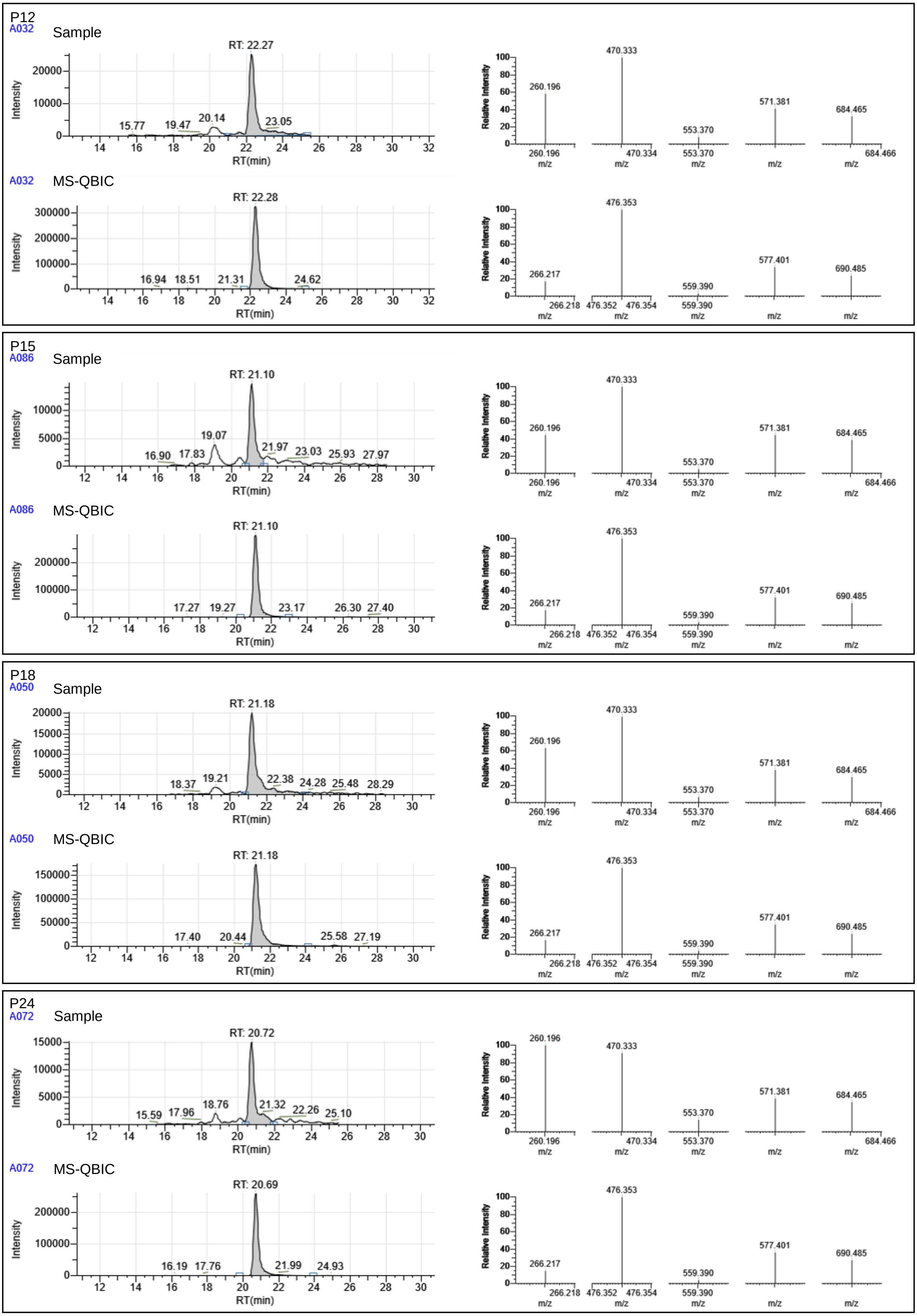

H Apo A4 (LAPLAEDVR) Heart

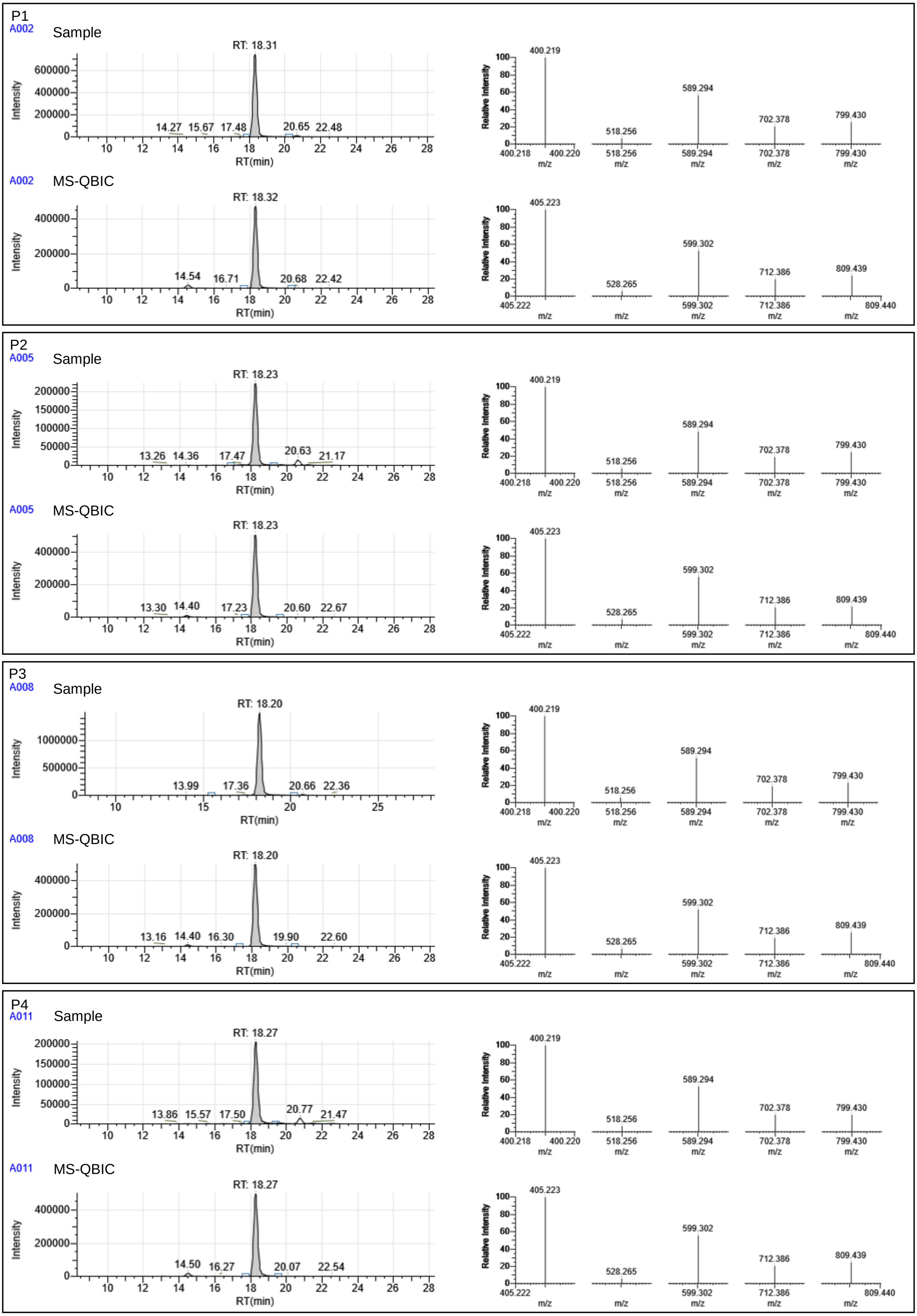

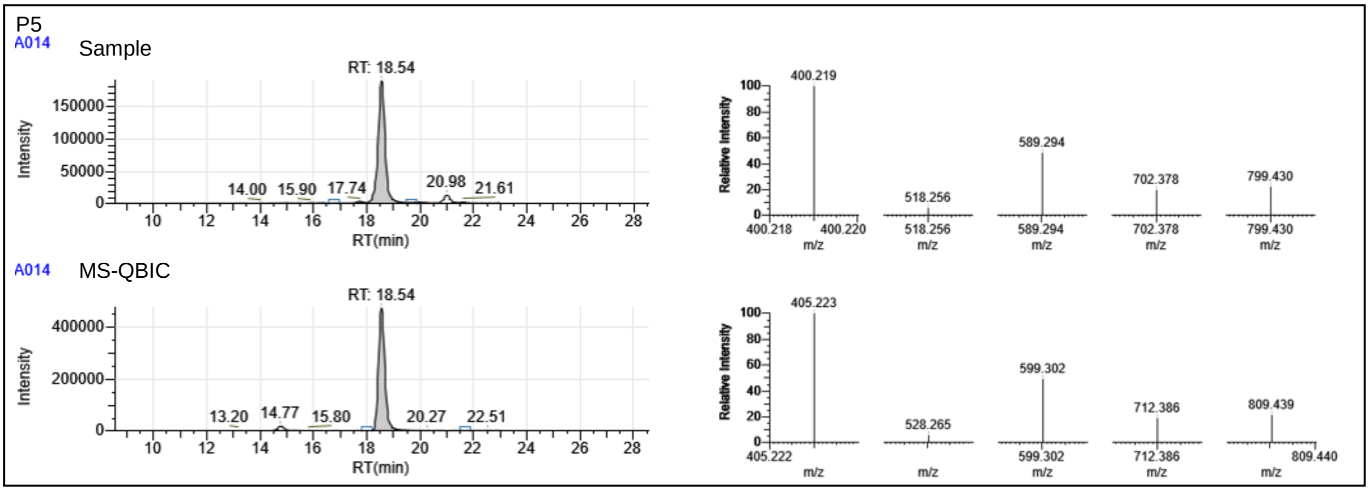
P5
A014 Sample
RT: 18.54
14.00 15.90 17.74 20.98 21.61
Intensity
RT(min)
400.219
589.294
518.256
702.378
799.430
Relative Intensity
m/z
A014 MS-QBIC
RT: 18.54
13.20 14.77 15.80 20.27 22.51
405.223
599.302
528.265
712.386
809.439

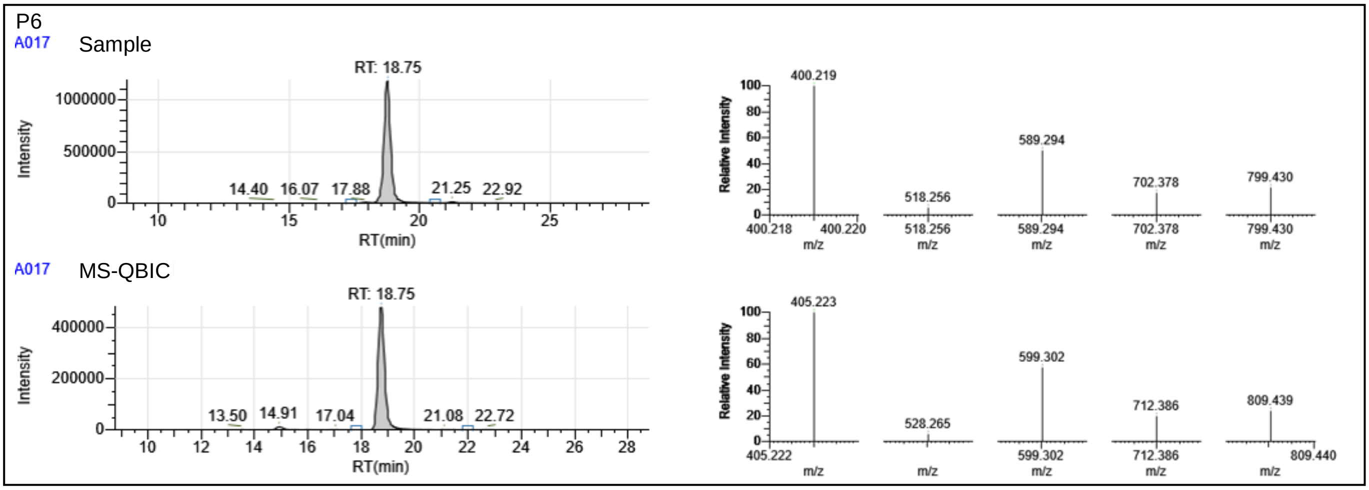
P6
A017 Sample
RT: 18.75
14.40 16.07 17.88 21.25 22.92
Intensity
RT(min)
400.219
589.294
518.256
702.378
799.430
Relative Intensity
m/z
A017 MS-QBIC
RT: 18.75
13.50 14.91 17.04 21.08 22.72
405.223
599.302
528.265
712.386
809.439

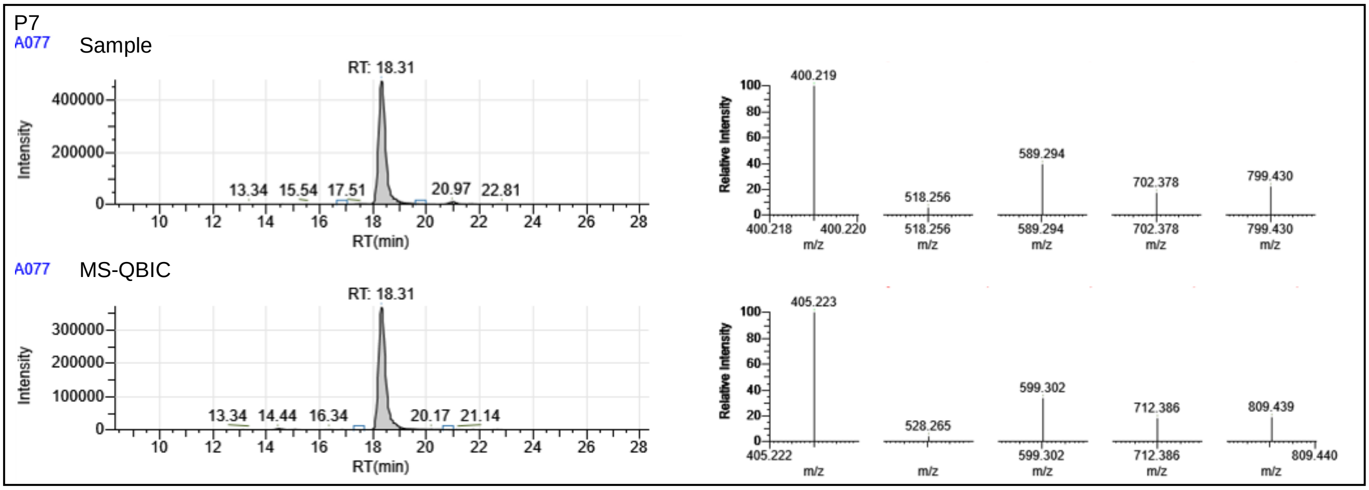
P7
A077 Sample
RT: 18.31
13.34 15.54 17.51 20.97 22.81
Intensity
RT(min)
400.219
589.294
518.256
702.378
799.430
Relative Intensity
m/z
A077 MS-QBIC
RT: 18.31
13.34 14.44 16.34 20.17 21.14
405.223
599.302
528.265
712.386
809.439

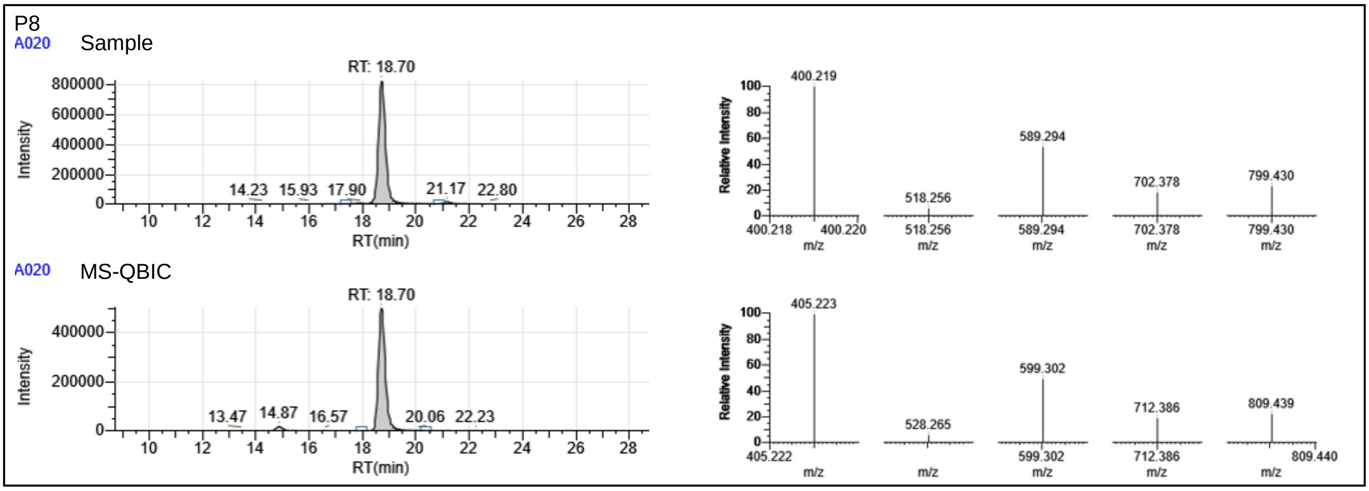
P8
A020 Sample
RT: 18.70
14.23 15.93 17.90 21.17 22.80
Intensity
RT(min)
400.219
589.294
518.256
702.378
799.430
Relative Intensity
m/z
A020 MS-QBIC
RT: 18.70
13.47 14.87 16.57 20.06 22.23
405.223
599.302
528.265
712.386
809.439

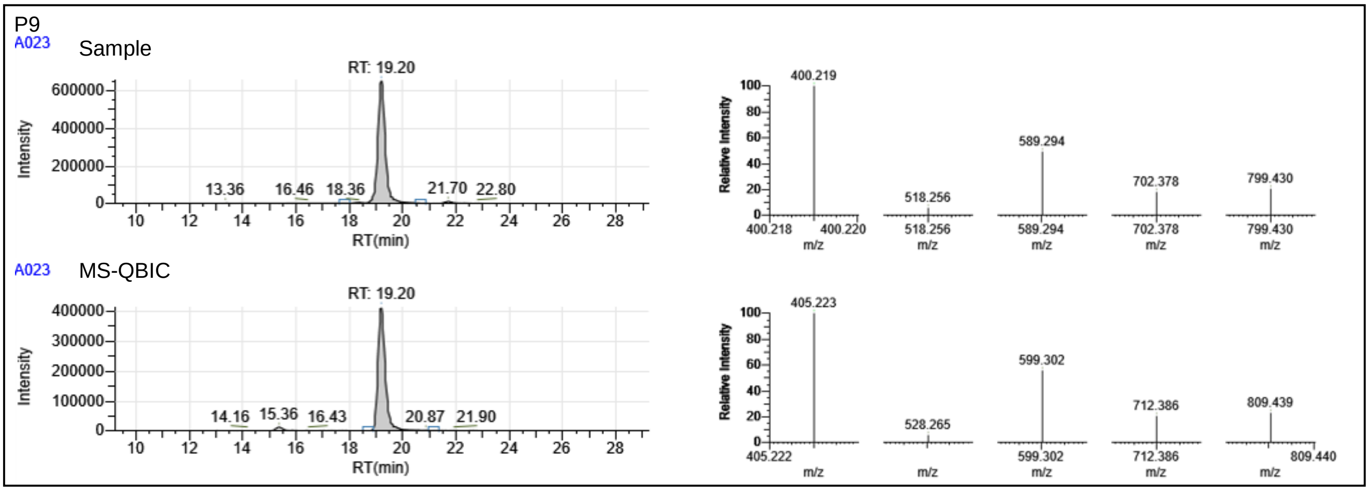
P9
A023 Sample
RT: 19.20
Intensity
RT(min)
13.36 16.46 18.36 21.70 22.80
400.219
589.294
518.256
702.378
799.430
Relative Intensity
m/z
A023 MS-QBIC
RT: 19.20
14.16 15.36 16.43 20.87 21.90
405.223
599.302
528.265
712.386
809.439

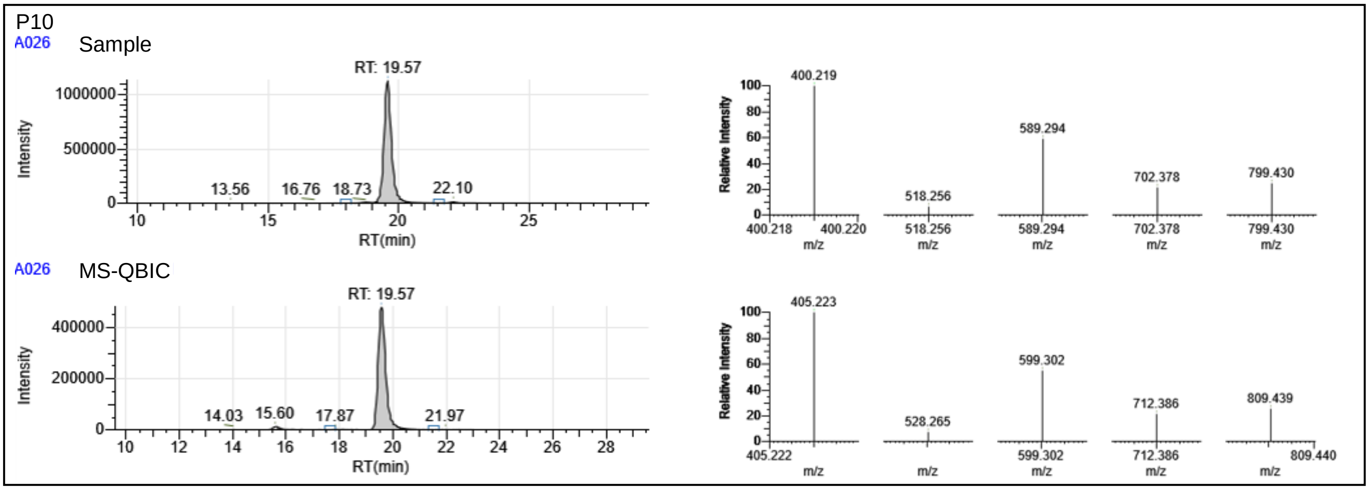
P10
A026 Sample
RT: 19.57
Intensity
RT(min)
13.56 16.76 18.73 22.10
400.219
589.294
518.256
702.378
799.430
Relative Intensity
m/z
A026 MS-QBIC
RT: 19.57
14.03 15.60 17.87 21.97
405.223
599.302
528.265
712.386
809.439

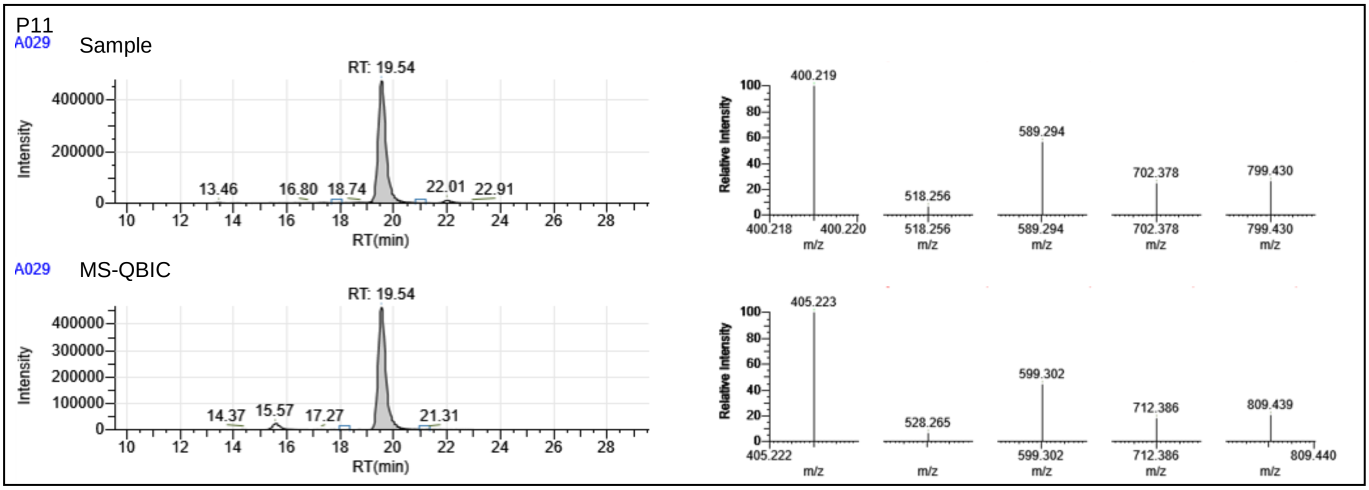
P11
A029 Sample
RT: 19.54
Intensity
RT(min)
13.46 16.80 18.74 22.01 22.91
400.219
589.294
518.256
702.378
799.430
Relative Intensity
m/z
A029 MS-QBIC
RT: 19.54
14.37 15.57 17.27 21.31
405.223
599.302
528.265
712.386
809.439

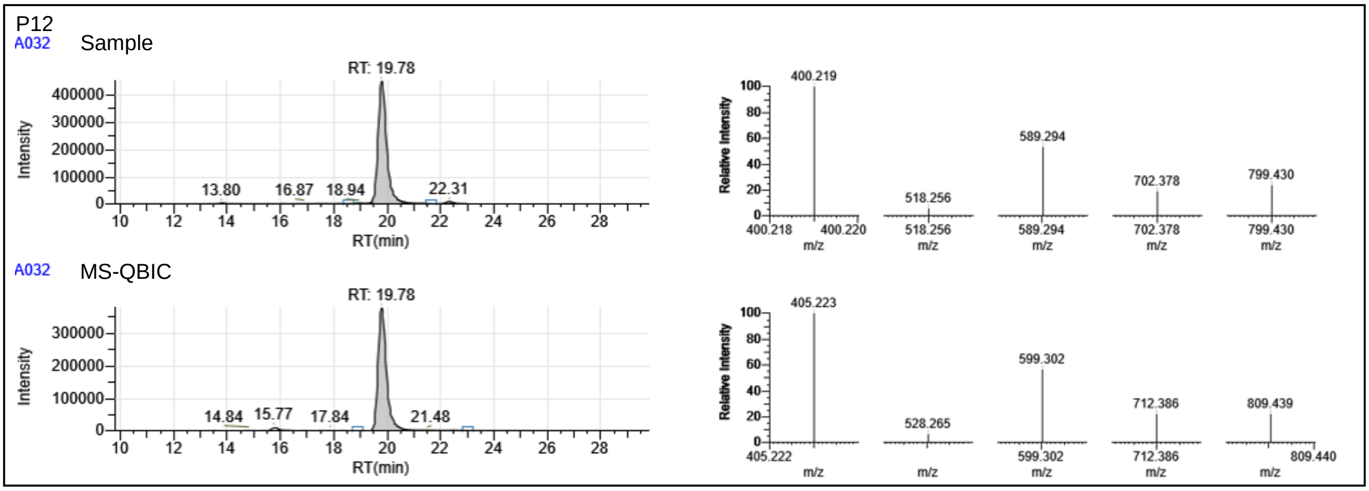
P12
A032 Sample
RT: 19.78
Intensity
RT(min)
13.80 16.87 18.94 22.31
400.219
589.294
518.256
702.378
799.430
Relative Intensity
m/z
A032 MS-QBIC
RT: 19.78
14.84 15.77 17.84 21.48
405.223
599.302
528.265
712.386
809.439

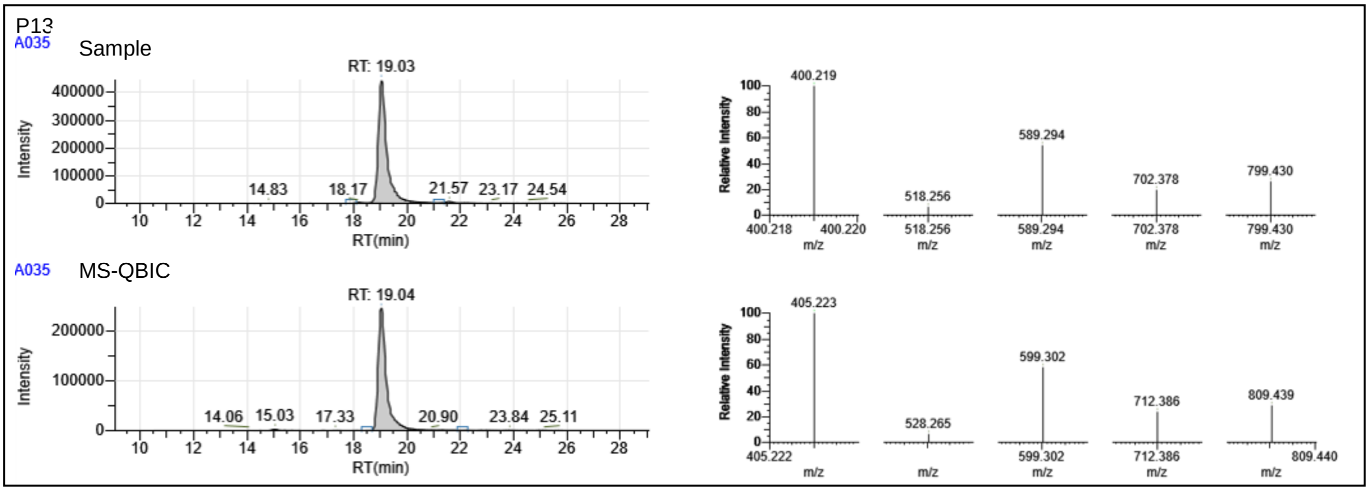
P13
A035 Sample
RT: 19.03
Intensity
RT(min)
Relative Intensity
400.219
518.256
589.294
702.378
799.430
m/z
A035 MS-QBIC
RT: 19.04
405.223
528.265
599.302
712.386
809.439

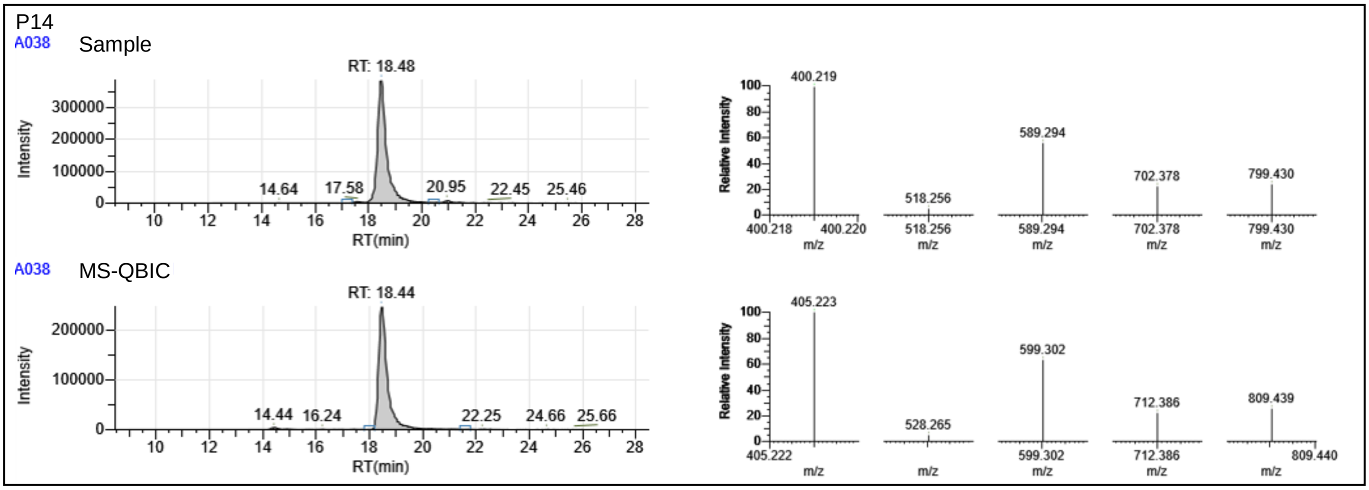
P14
A038 Sample
RT: 18.48
Intensity
RT(min)
Relative Intensity
400.219
518.256
589.294
702.378
799.430
m/z
A038 MS-QBIC
RT: 18.44
405.223
528.265
599.302
712.386
809.439

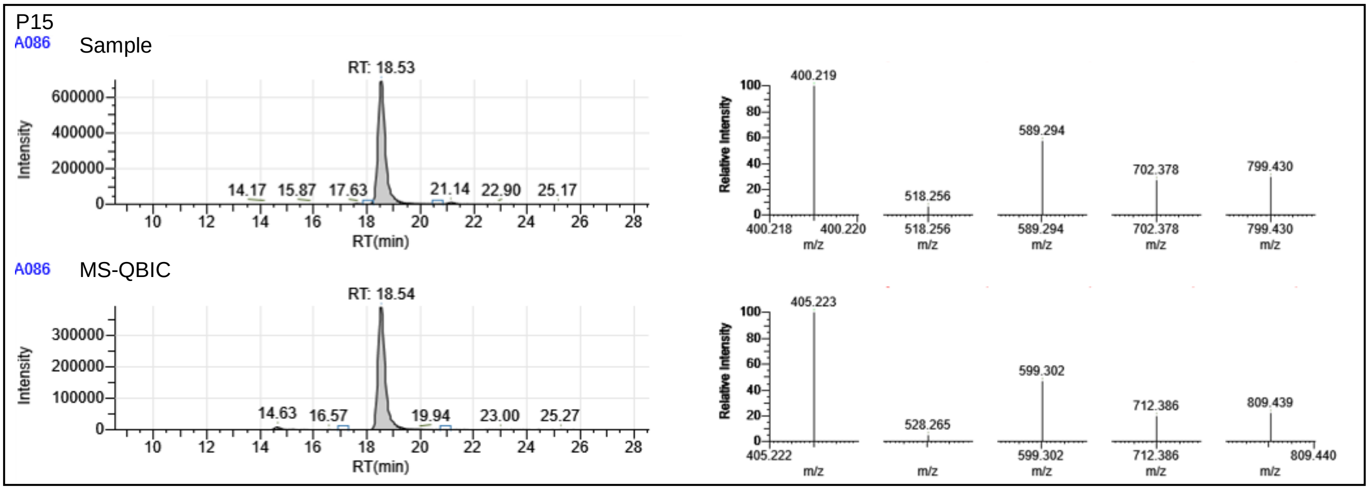
P15
A086 Sample
RT: 18.53
Intensity
RT(min)
Relative Intensity
400.219
518.256
589.294
702.378
799.430
m/z
A086 MS-QBIC
RT: 18.54
405.223
528.265
599.302
712.386
809.439

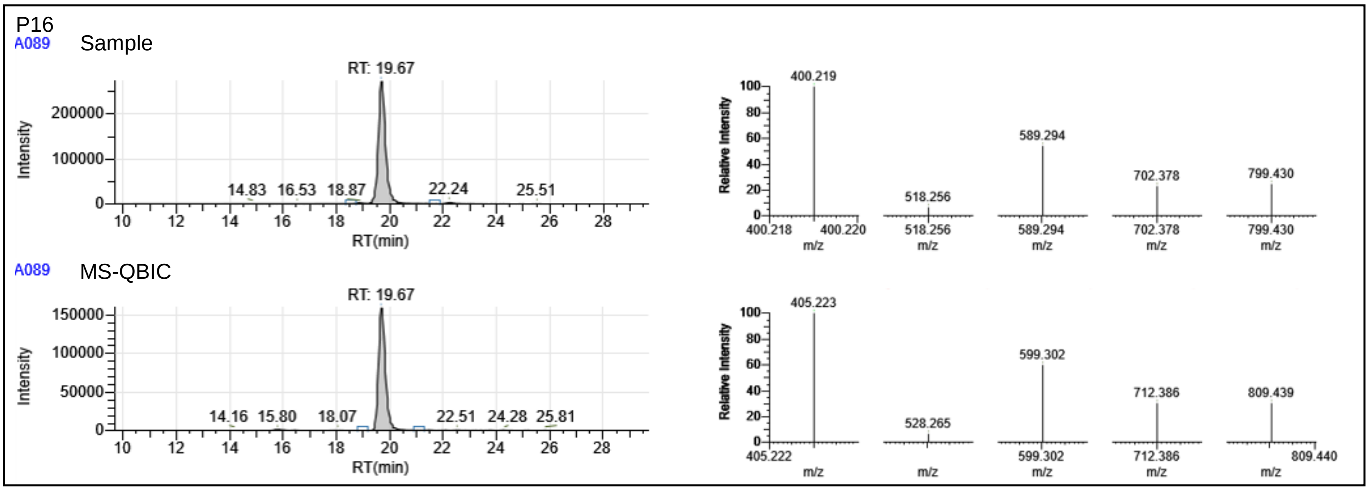
P16
A089 Sample
RT: 19.67
Intensity
RT(min)
Relative Intensity
400.219
518.256
589.294
702.378
799.430
m/z
A089 MS-QBIC
RT: 19.67
405.223
528.265
599.302
712.386
809.439

P17

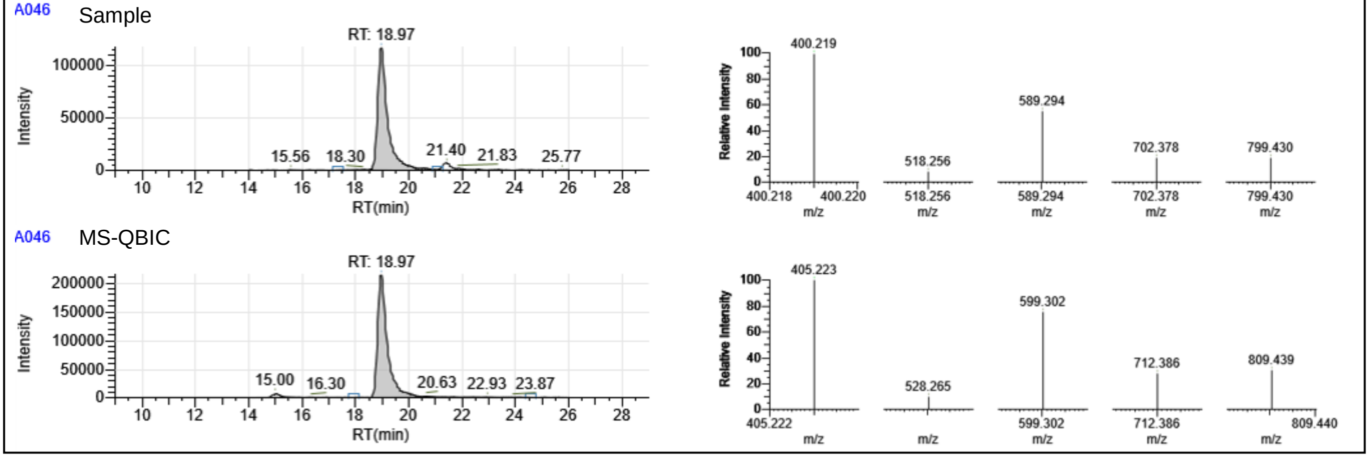

P18

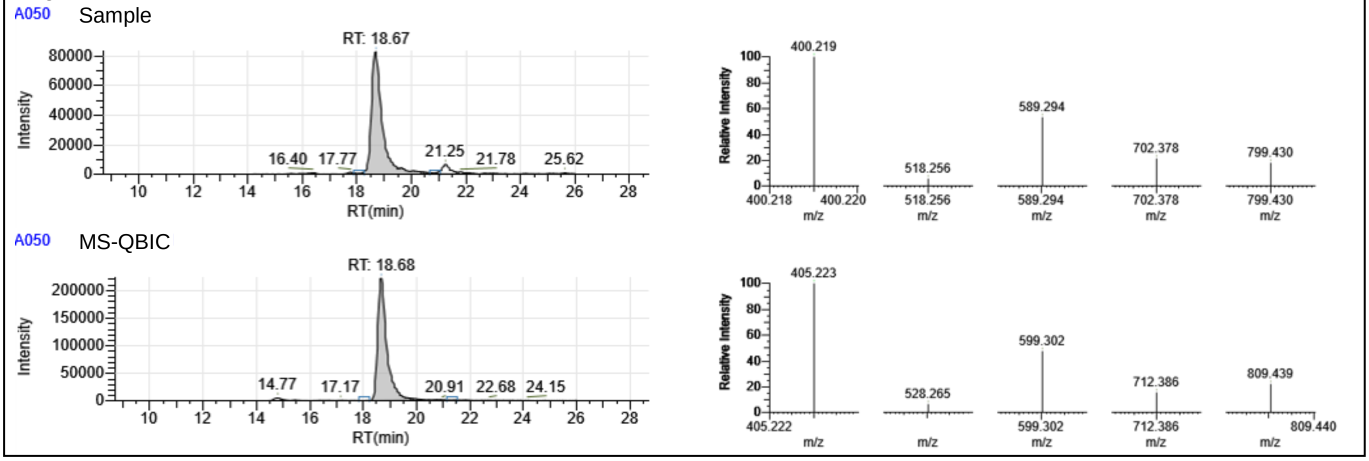

P19

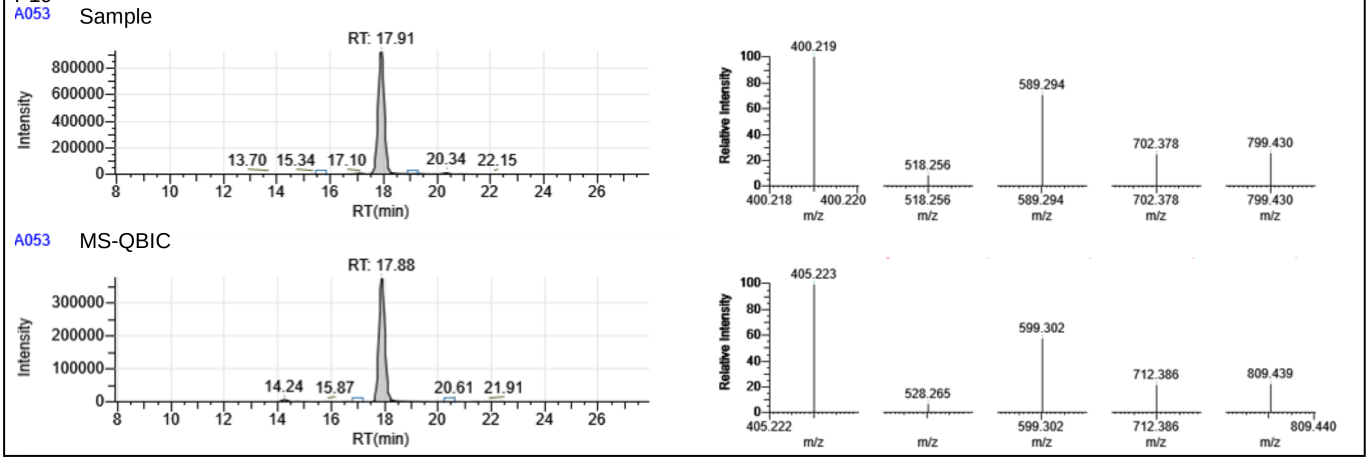

P20

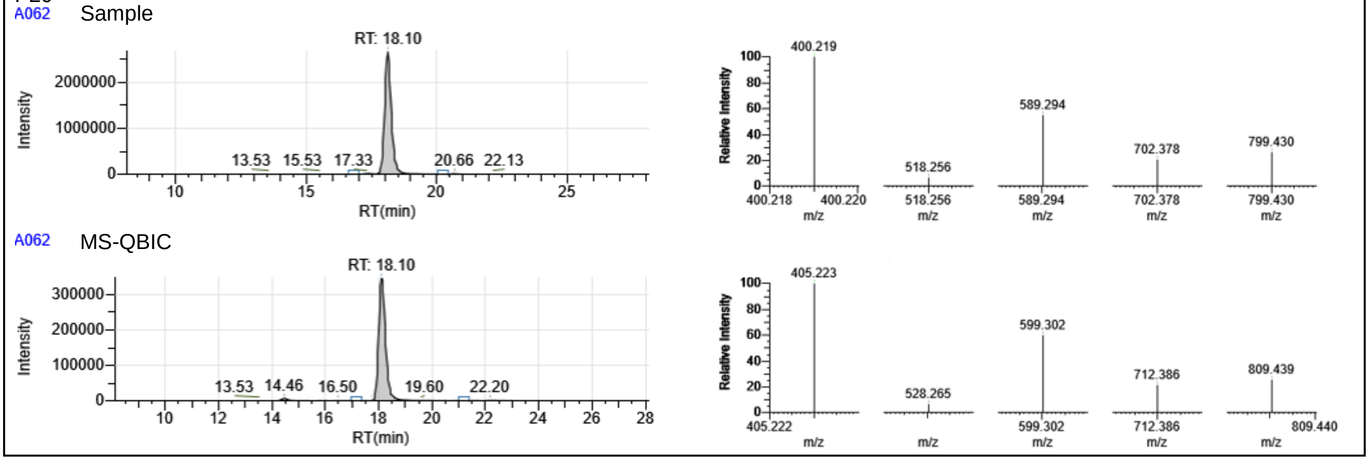

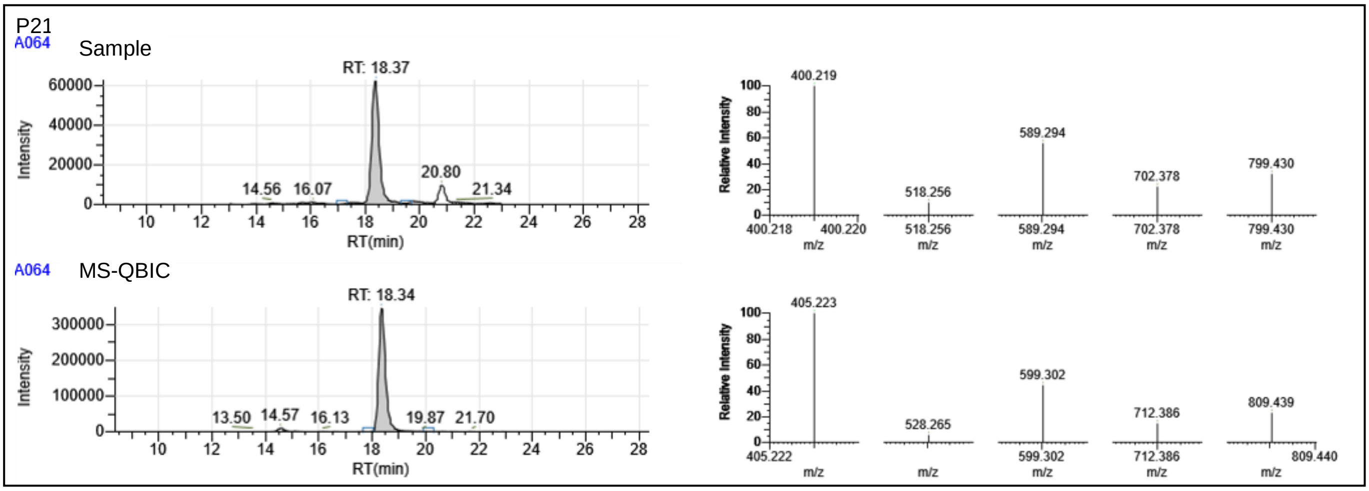
P21
A064 Sample
RT: 18.37
14.56 16.07 20.80 21.34
Intensity
RT(min)
400.219
589.294
702.378
799.430
518.256
Relative Intensity
m/z
A064 MS-QBIC
RT: 18.34
13.50 14.57 16.13 19.87 21.70
405.223
599.302
712.386
809.439
528.265
405.222
809.440

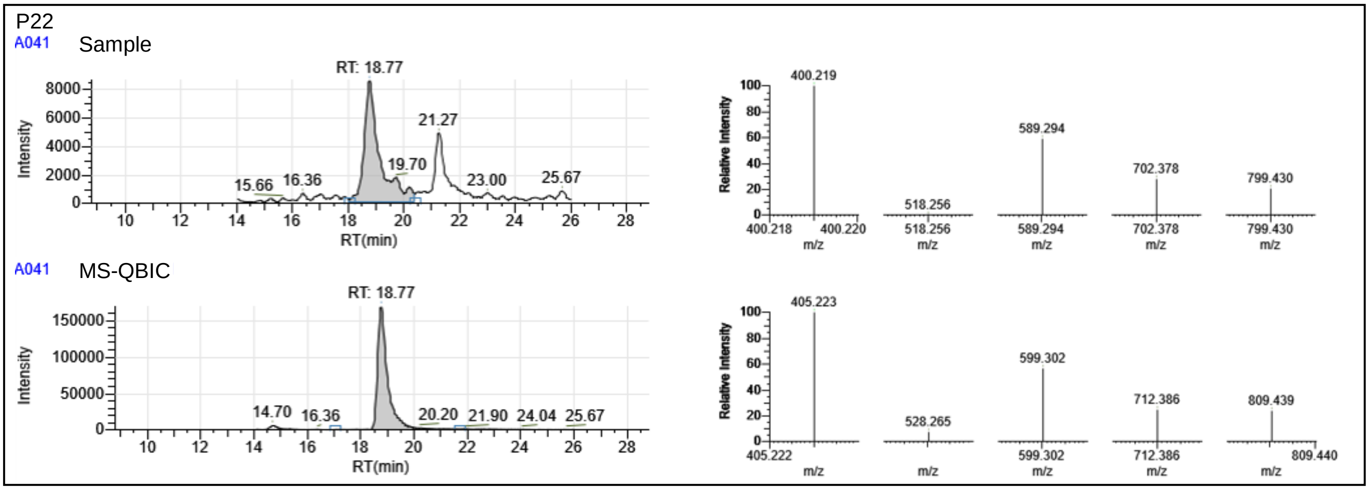
P22
A041 Sample
RT: 18.77
21.27
19.70
15.66 16.36 23.00 25.67
Intensity
RT(min)
400.219
589.294
702.378
799.430
518.256
Relative Intensity
m/z
A041 MS-QBIC
RT: 18.77
14.70 16.36 20.20 21.90 24.04 25.67
405.223
599.302
712.386
809.439
528.265
405.222
809.440

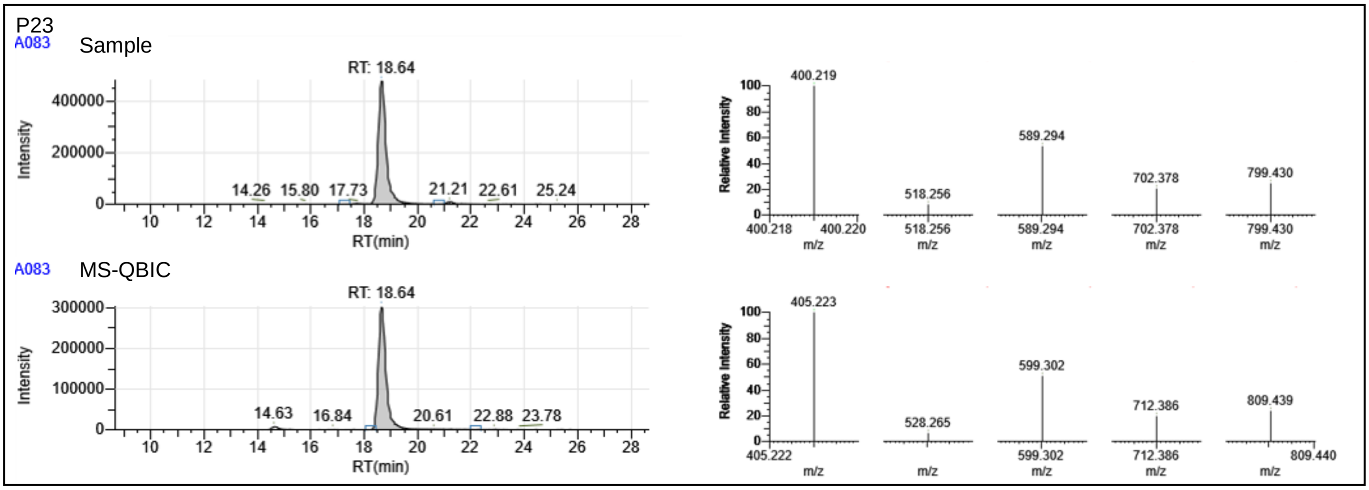
P23
A083 Sample
RT: 18.64
14.26 15.80 17.73 21.21 22.61 25.24
Intensity
RT(min)
400.219
589.294
702.378
799.430
518.256
Relative Intensity
m/z
A083 MS-QBIC
RT: 18.64
14.63 16.84 20.61 22.88 23.78
405.223
599.302
712.386
809.439
528.265
405.222
809.440

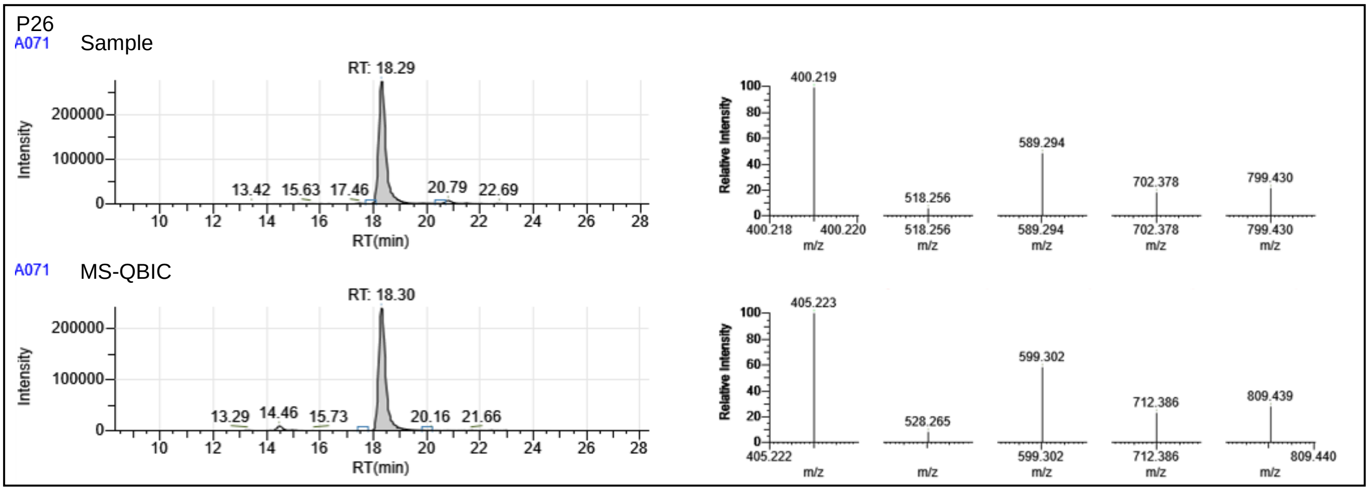
P26
A071 Sample
RT: 18.29
13.42 15.63 17.46 20.79 22.69
Intensity
RT(min)
400.219
589.294
702.378
799.430
518.256
Relative Intensity
m/z
A071 MS-QBIC
RT: 18.30
13.29 14.46 15.73 20.16 21.66
405.223
599.302
712.386
809.439
528.265
405.222
809.440

P25

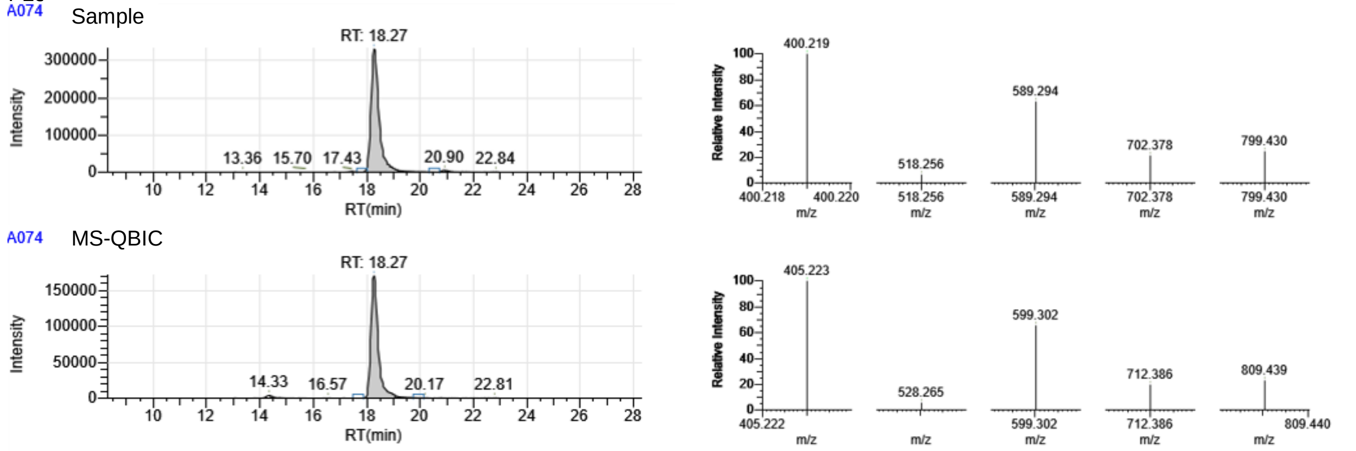

P26

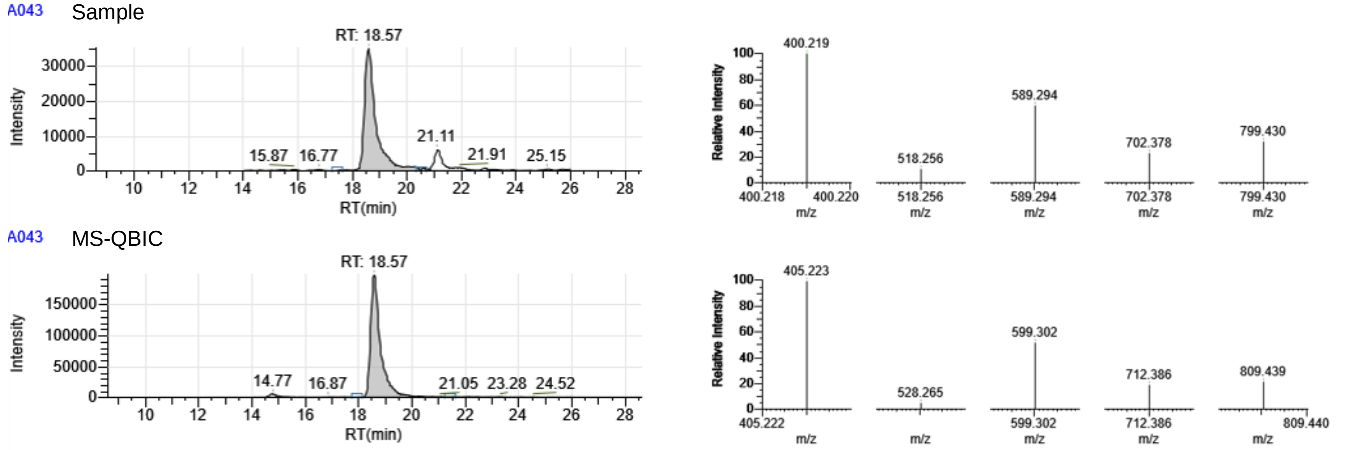

P27

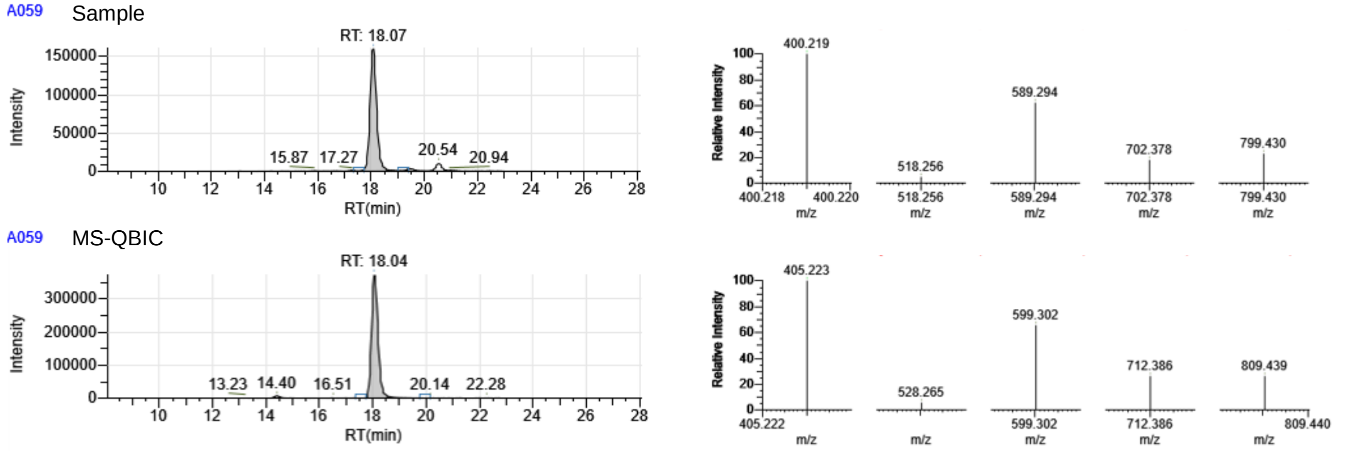

P28

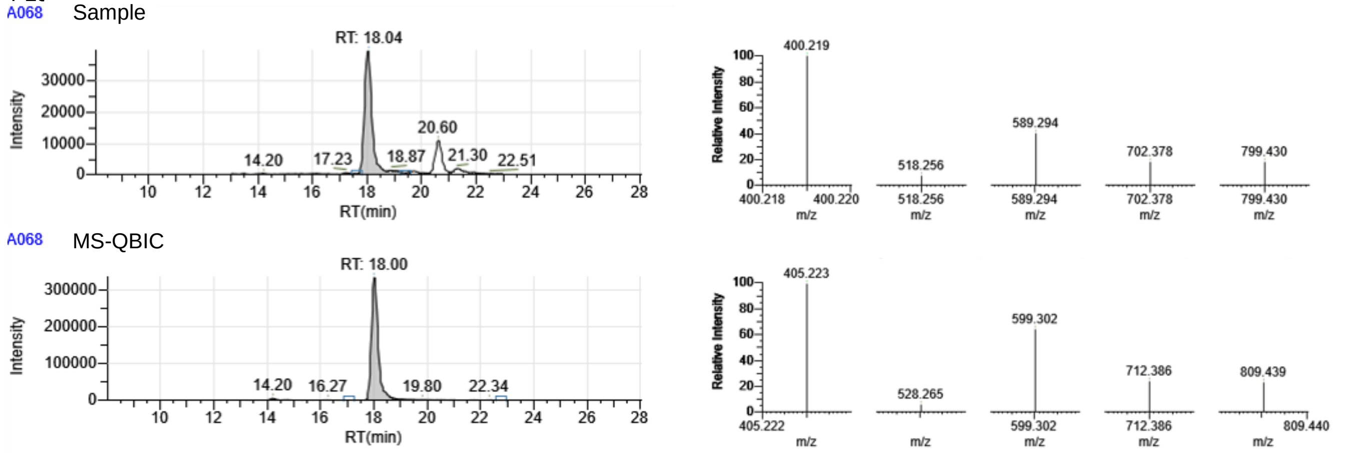

P29

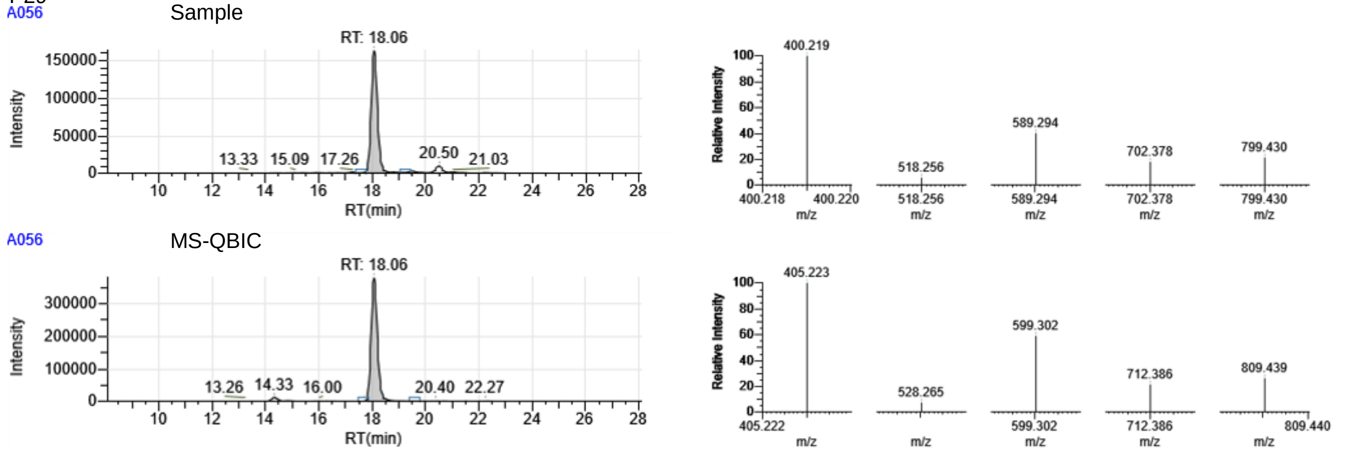
A056
Sample
RT: 18.06
13.33
15.09
17.26
20.50
21.03
Intensity
RT(min)
400.219
518.256
589.294
702.378
799.430
Relative Intensity
m/z
A056
MS-QBIC
RT: 18.06
13.26
14.33
16.00
20.40
22.27
Intensity
RT(min)
405.223
528.265
599.302
712.386
809.439
405.222
809.440
Relative Intensity
m/z

P30

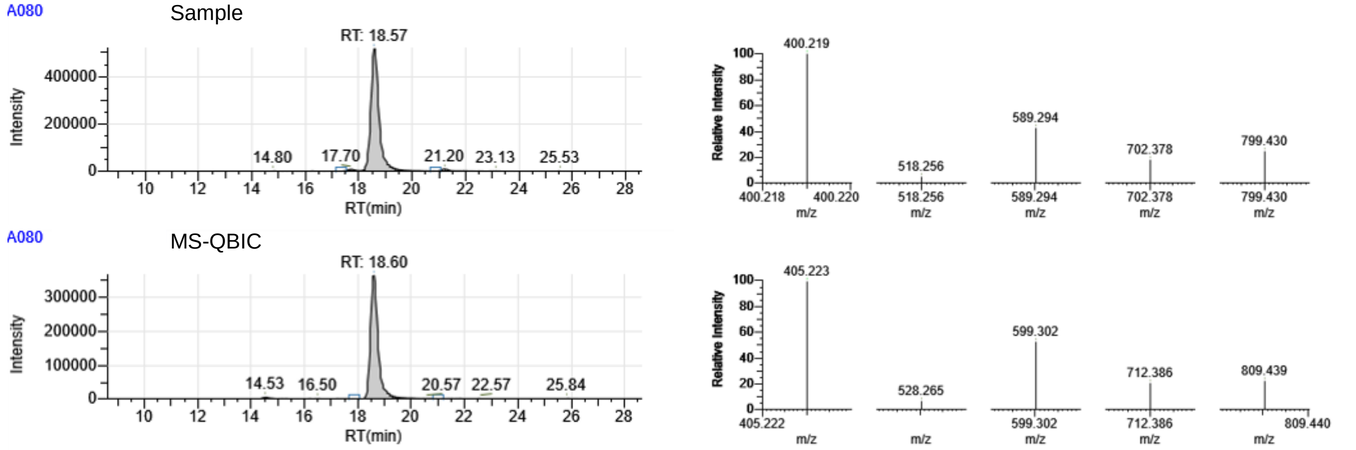
A080
Sample
RT: 18.57
14.80
17.70
21.20
23.13
25.53
Intensity
RT(min)
400.219
518.256
589.294
702.378
799.430
Relative Intensity
m/z
A080
MS-QBIC
RT: 18.60
14.53
16.50
20.57
22.57
25.84
Intensity
RT(min)
405.223
528.265
599.302
712.386
809.439
405.222
809.440
Relative Intensity
m/z

I Apo E (LGPLVEQGR) Heart

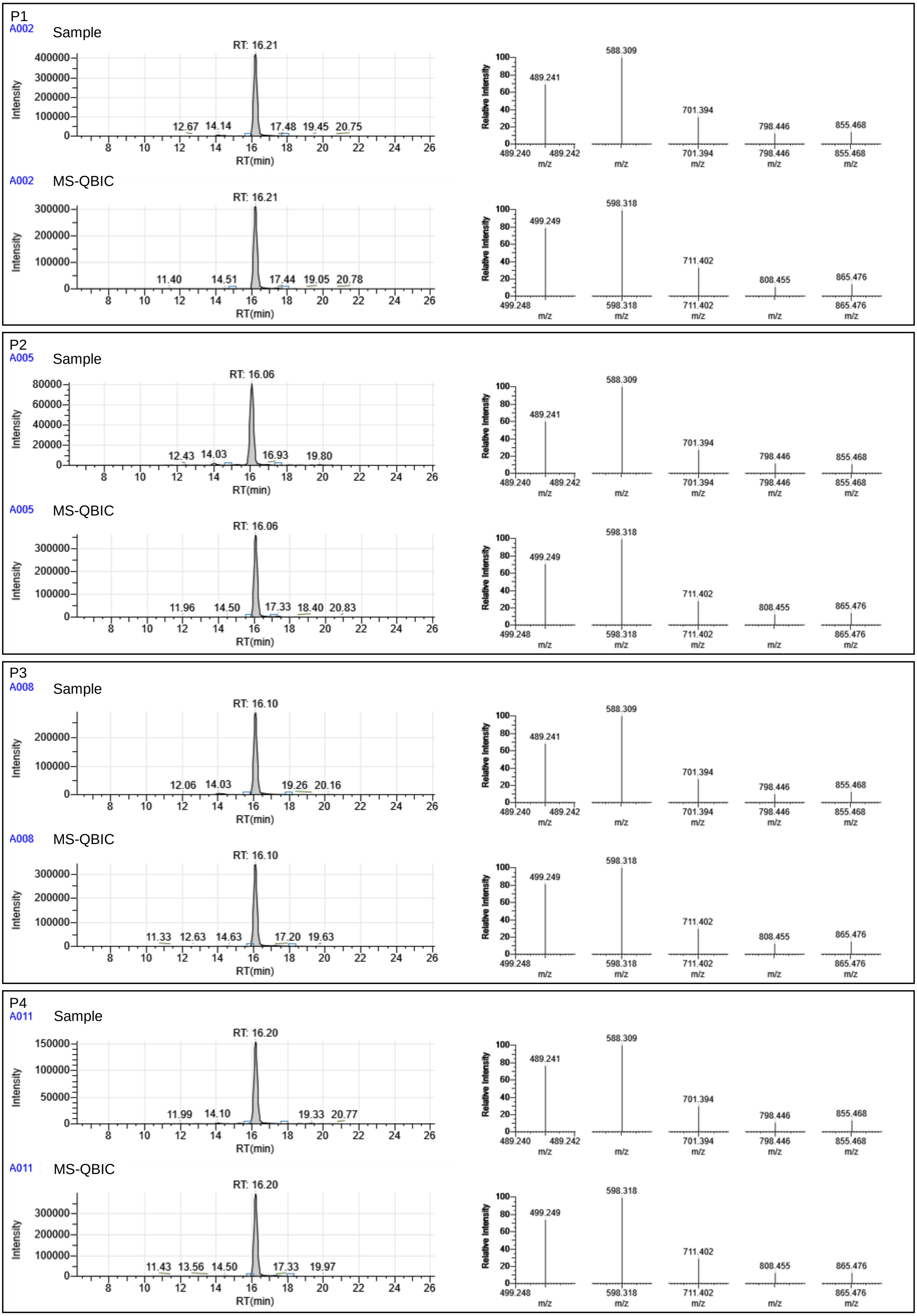

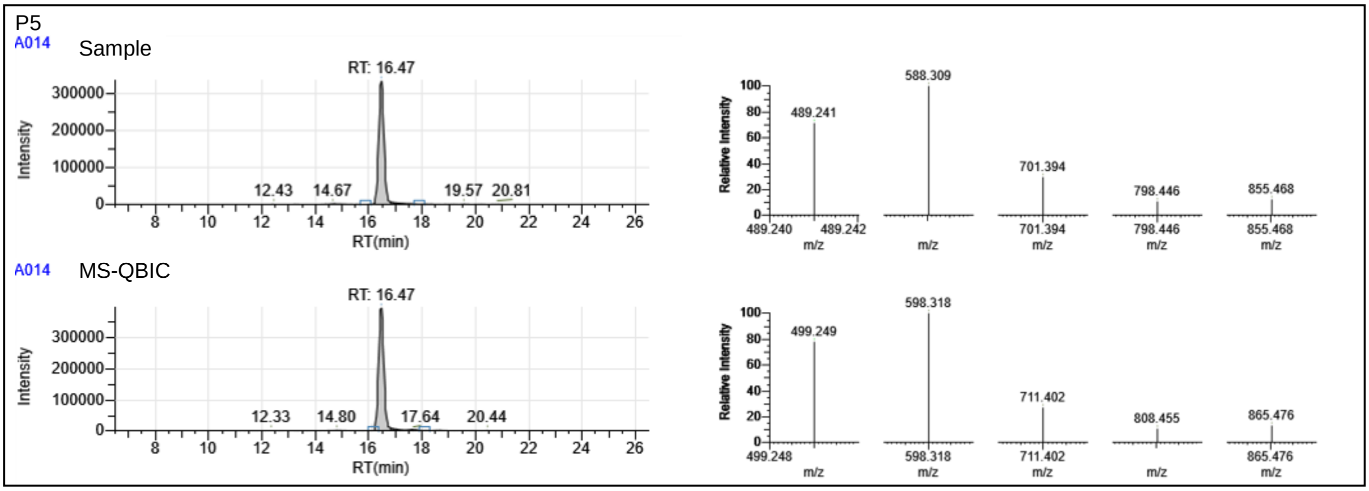
P5
A014 Sample
RT: 16.47
12.43 14.67 19.57 20.81
Intensity
RT(min)
489.241
588.309
701.394
798.446
855.468
Relative Intensity
m/z
A014 MS-QBIC
RT: 16.47
12.33 14.80 17.64 20.44
499.249
598.318
711.402
808.455
865.476

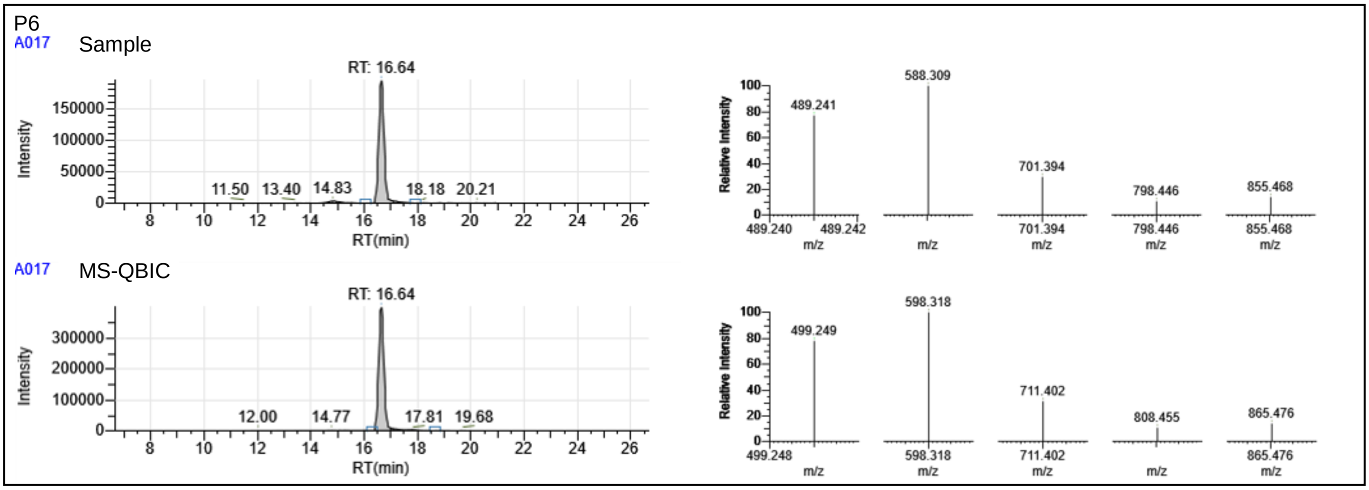
P6
A017 Sample
RT: 16.64
11.50 13.40 14.83 18.18 20.21
Intensity
RT(min)
489.241
588.309
701.394
798.446
855.468
Relative Intensity
m/z
A017 MS-QBIC
RT: 16.64
12.00 14.77 17.81 19.68
499.249
598.318
711.402
808.455
865.476

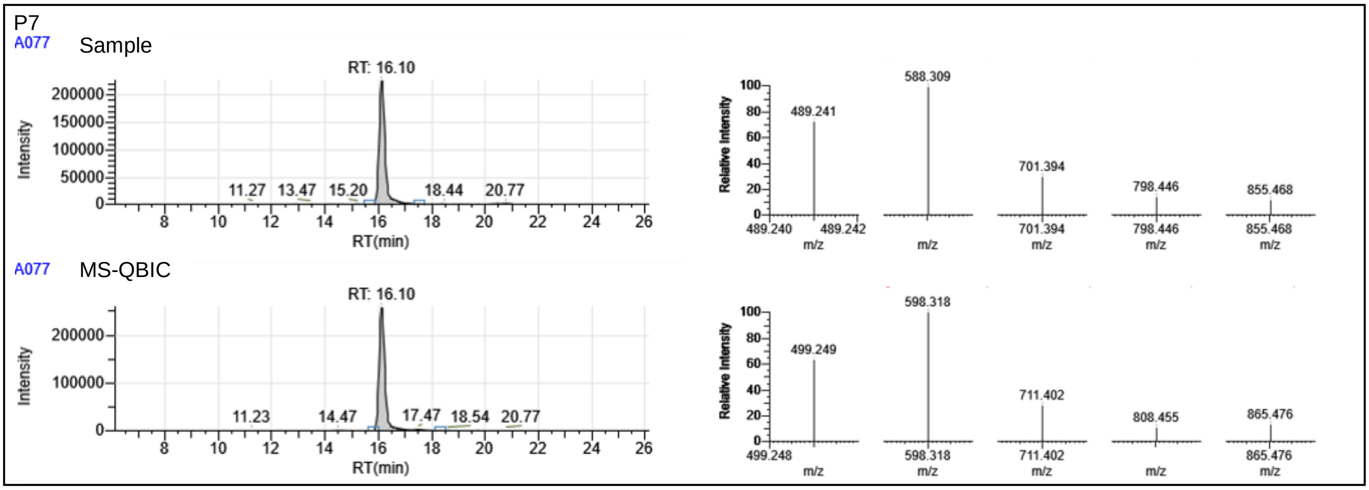
P7
A077 Sample
RT: 16.10
11.27 13.47 15.20 18.44 20.77
Intensity
RT(min)
489.241
588.309
701.394
798.446
855.468
Relative Intensity
m/z
A077 MS-QBIC
RT: 16.10
11.23 14.47 17.47 18.54 20.77
499.249
598.318
711.402
808.455
865.476

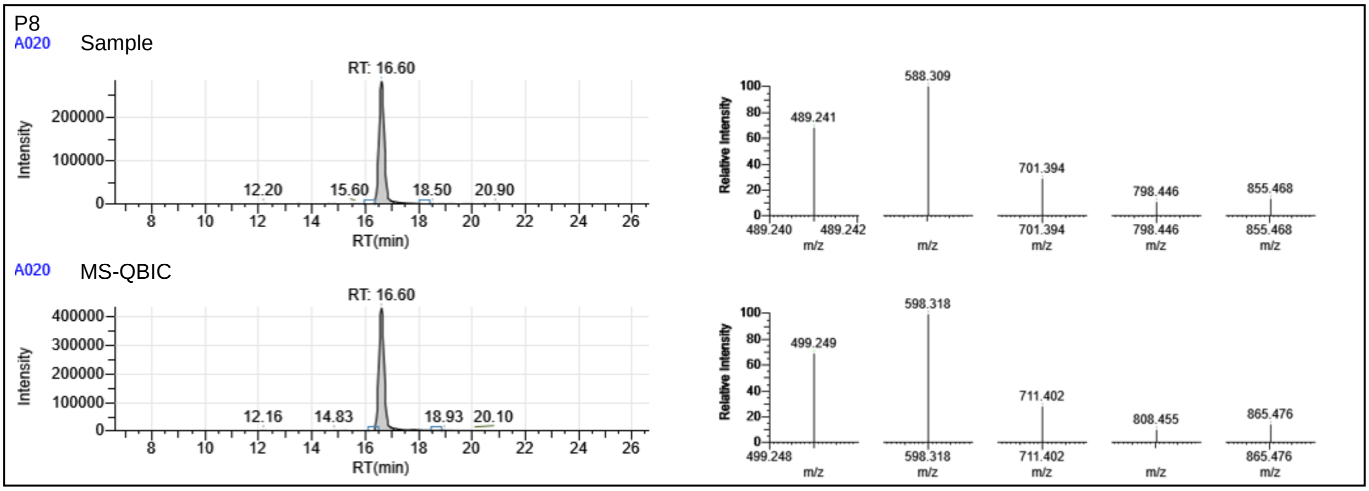
P8
A020 Sample
RT: 16.60
12.20 15.60 18.50 20.90
Intensity
RT(min)
489.241
588.309
701.394
798.446
855.468
Relative Intensity
m/z
A020 MS-QBIC
RT: 16.60
12.16 14.83 18.93 20.10
499.249
598.318
711.402
808.455
865.476

P9

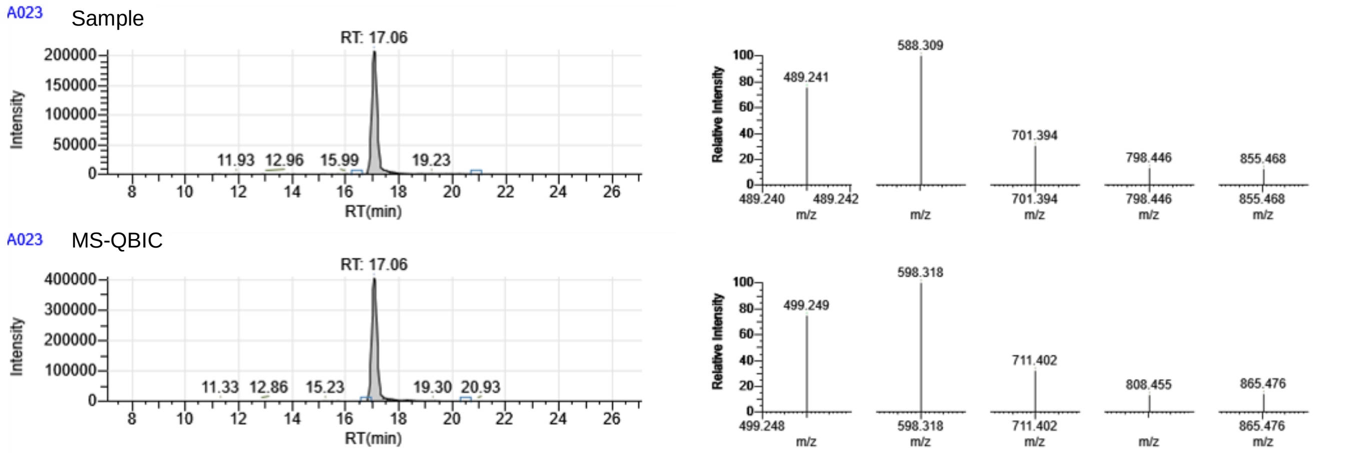

P10

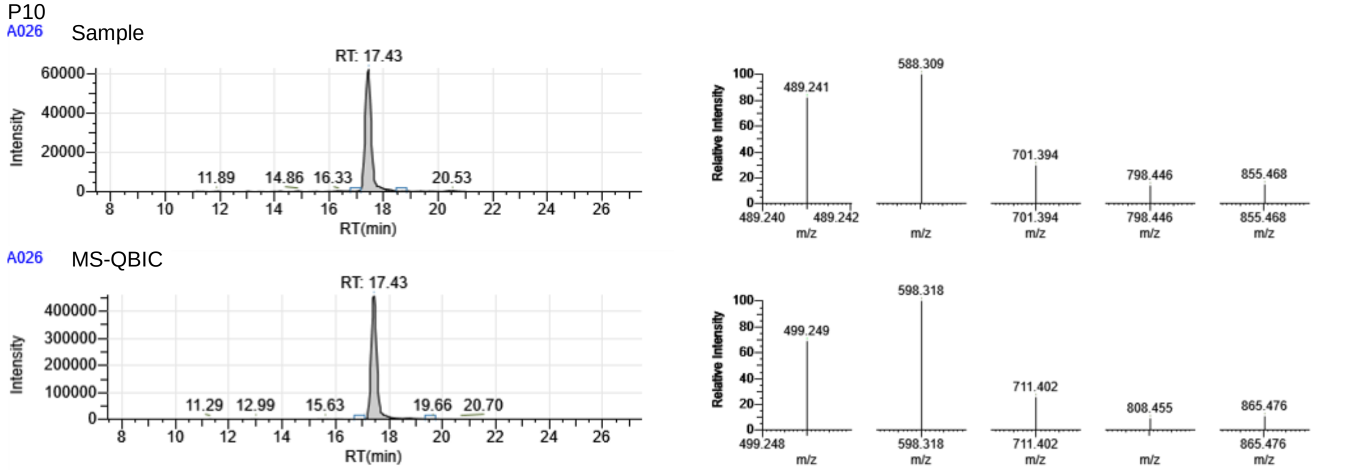

P11

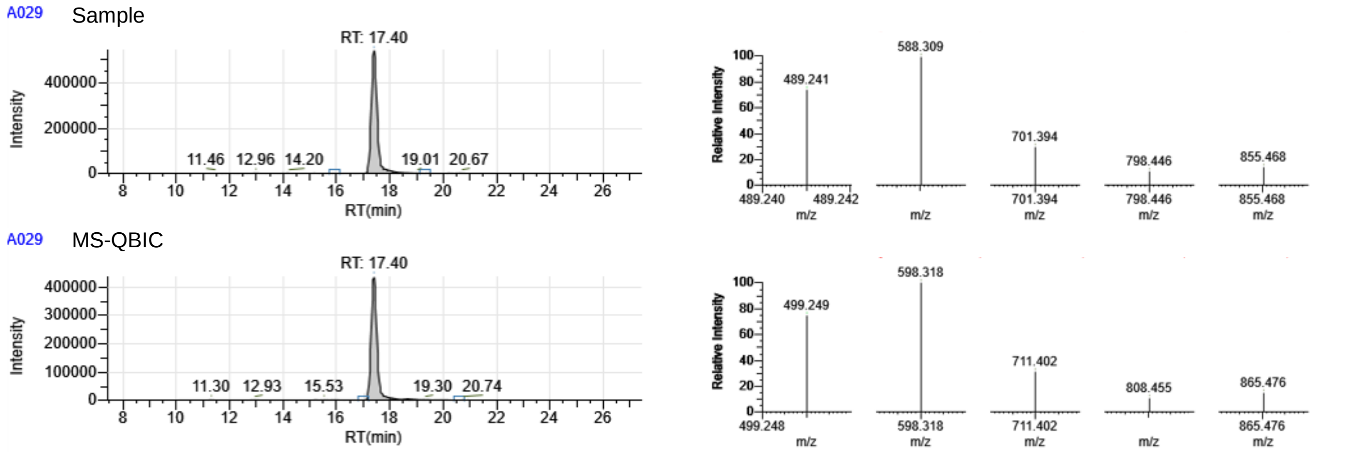

P12

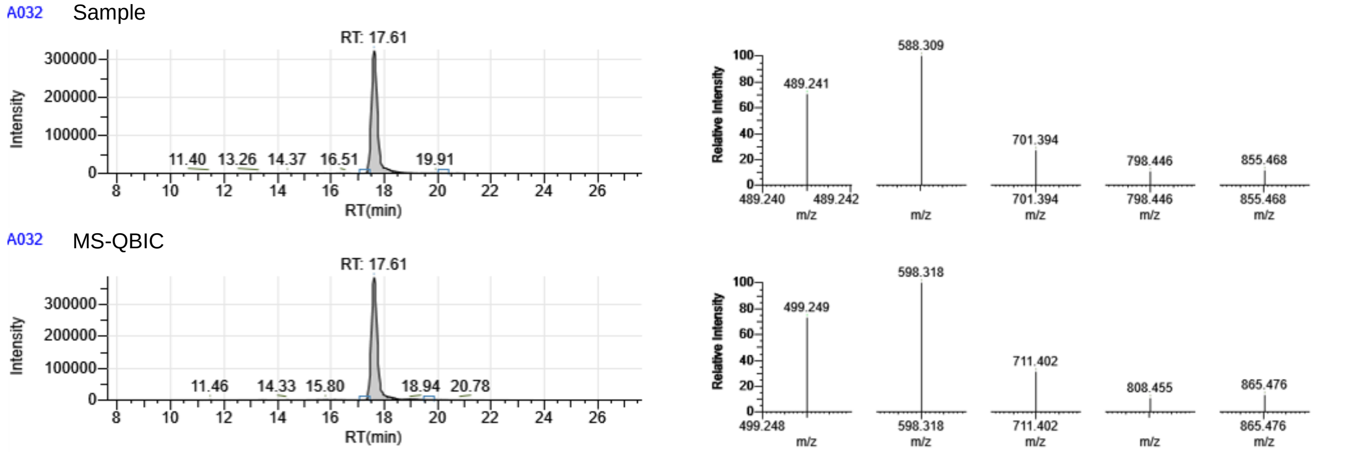

P13

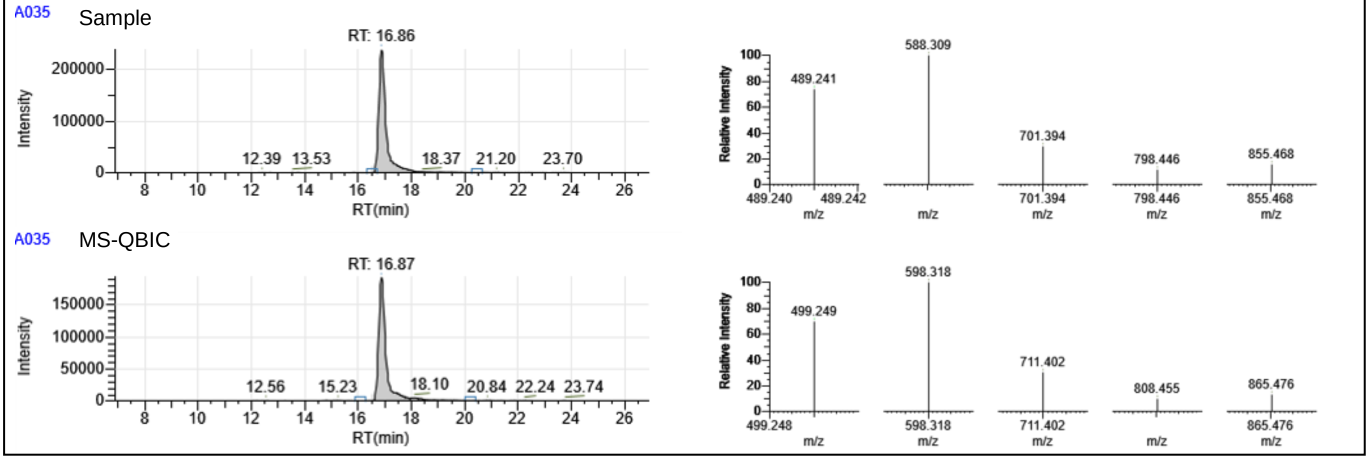

P14

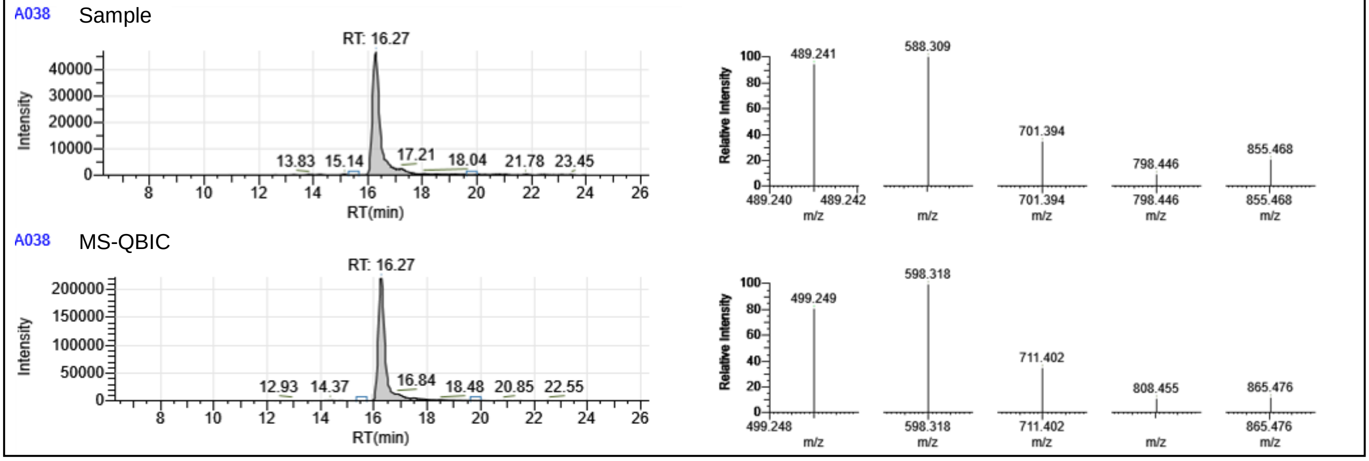

P15

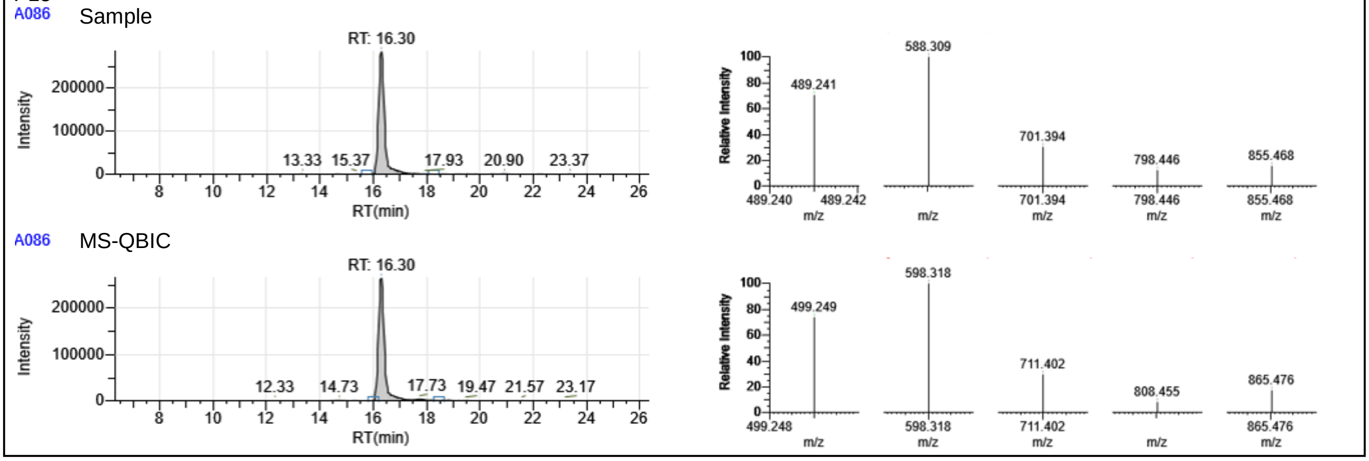

P16

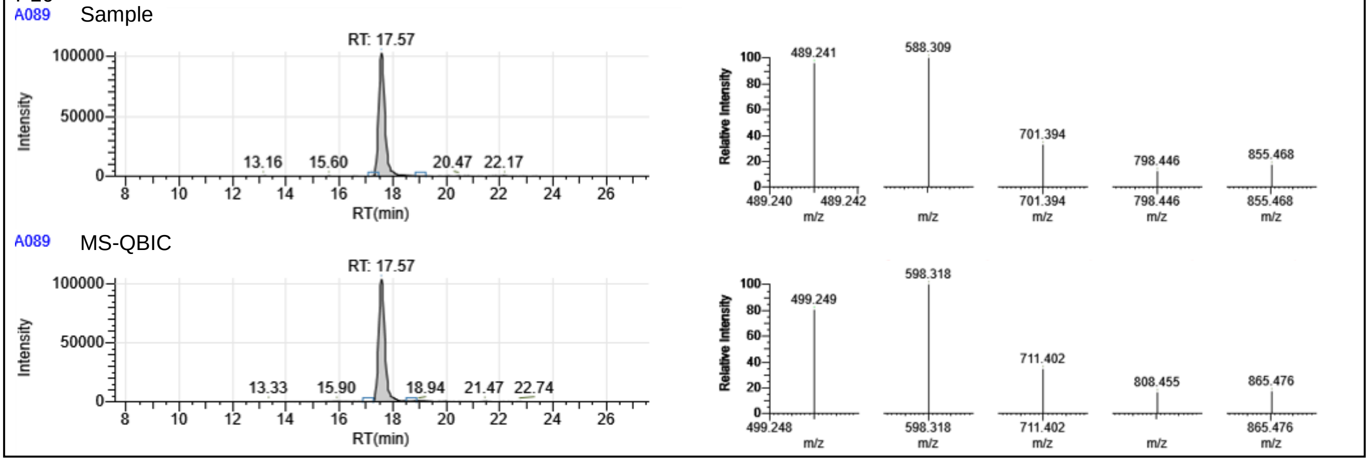

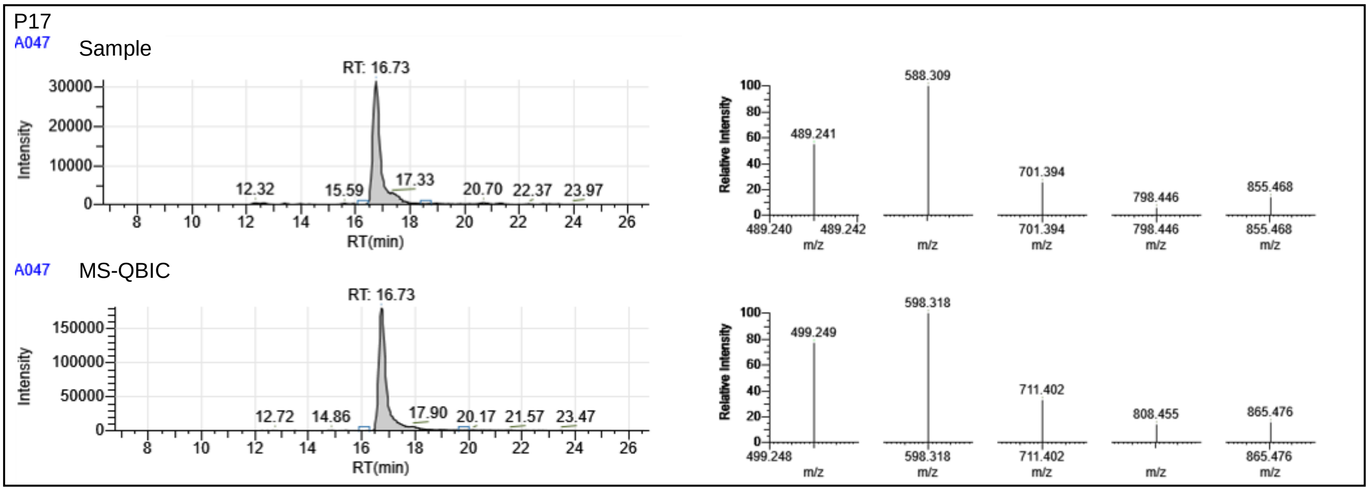
P17
A047 Sample
RT: 16.73
12.32 15.59 17.33 20.70 22.37 23.97
Intensity
RT(min)
489.241 588.309 701.394 798.446 855.468
Relative Intensity
m/z
A047 MS-QBIC
RT: 16.73
12.72 14.86 17.90 20.17 21.57 23.47
499.249 598.318 711.402 808.455 865.476

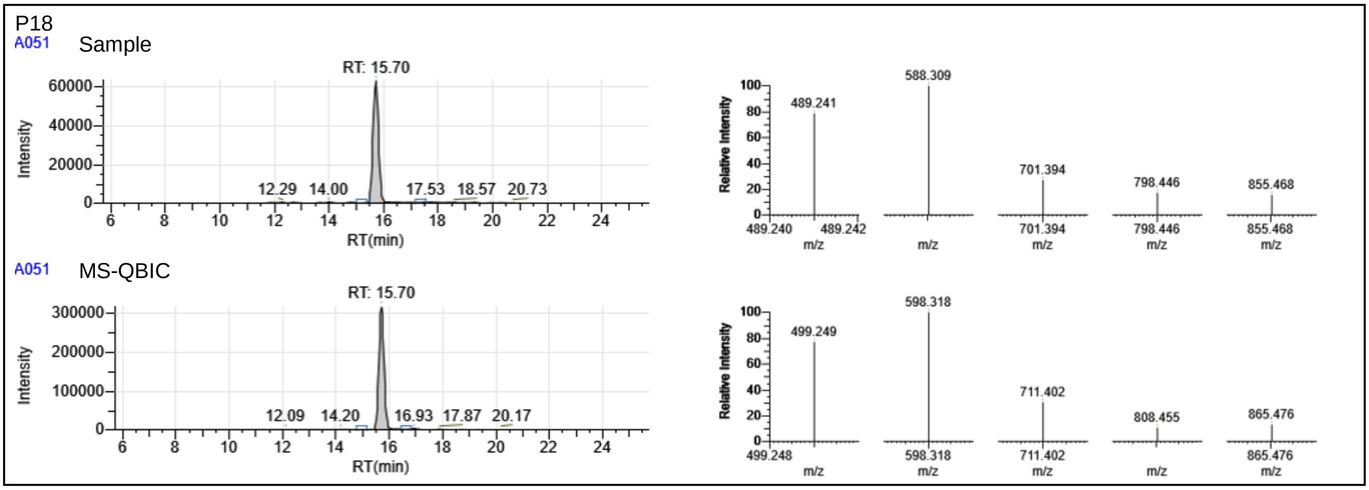
P18
A051 Sample
RT: 15.70
12.29 14.00 17.53 18.57 20.73
Intensity
RT(min)
489.241 588.309 701.394 798.446 855.468
Relative Intensity
m/z
A051 MS-QBIC
RT: 15.70
12.09 14.20 16.93 17.87 20.17
499.249 598.318 711.402 808.455 865.476

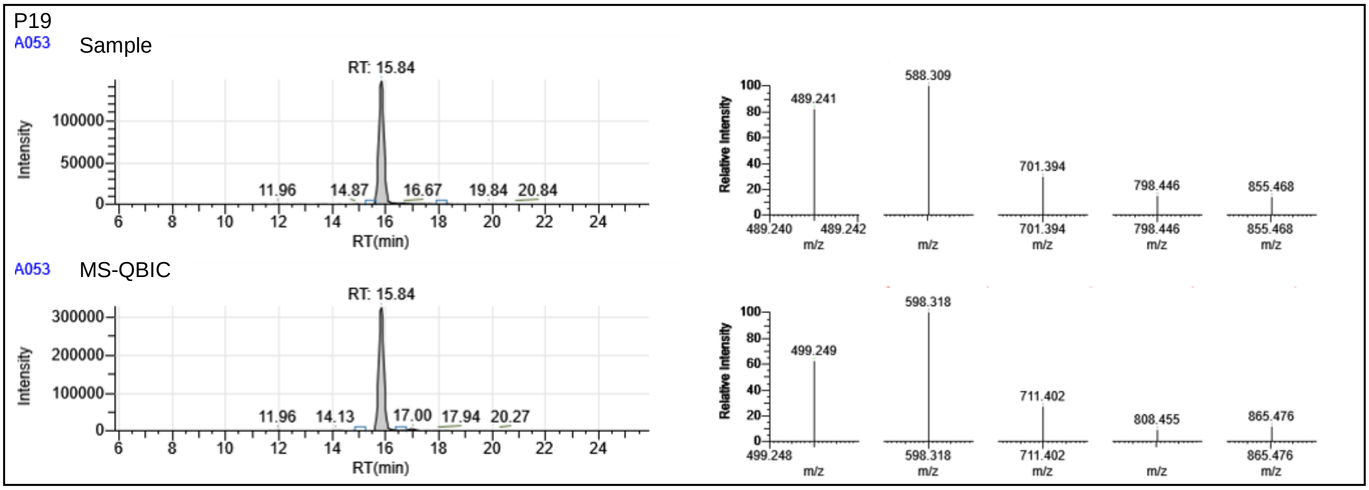
P19
A053 Sample
RT: 15.84
11.96 14.87 16.67 19.84 20.84
Intensity
RT(min)
489.241 588.309 701.394 798.446 855.468
Relative Intensity
m/z
A053 MS-QBIC
RT: 15.84
11.96 14.13 17.00 17.94 20.27
499.249 598.318 711.402 808.455 865.476

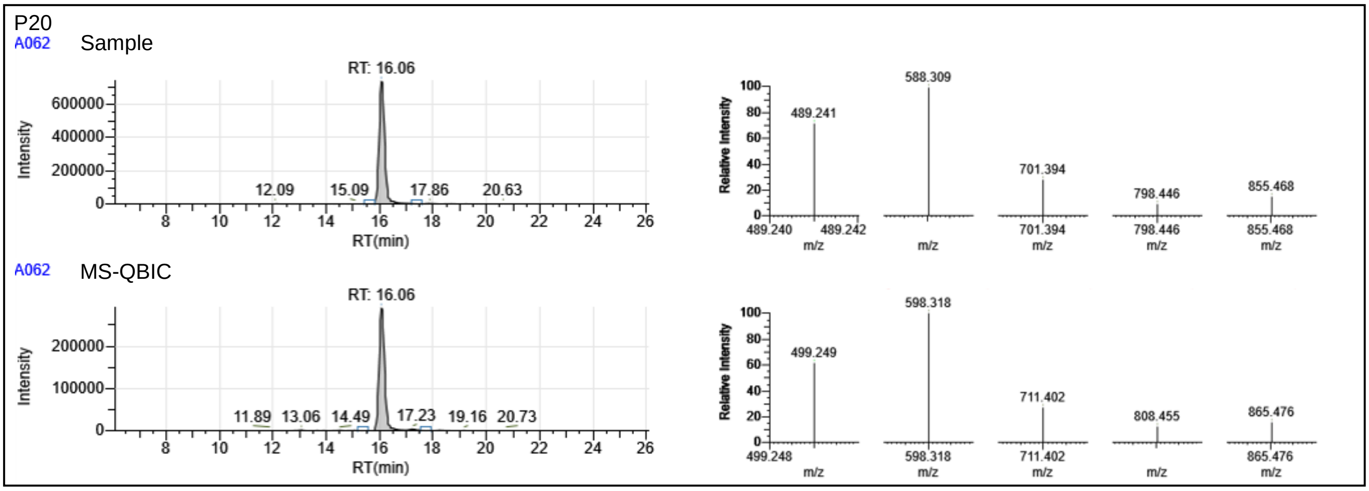
P20
A062 Sample
RT: 16.06
12.09 15.09 17.86 20.63
Intensity
RT(min)
489.241 588.309 701.394 798.446 855.468
Relative Intensity
m/z
A062 MS-QBIC
RT: 16.06
11.89 13.06 14.49 17.23 19.16 20.73
499.249 598.318 711.402 808.455 865.476

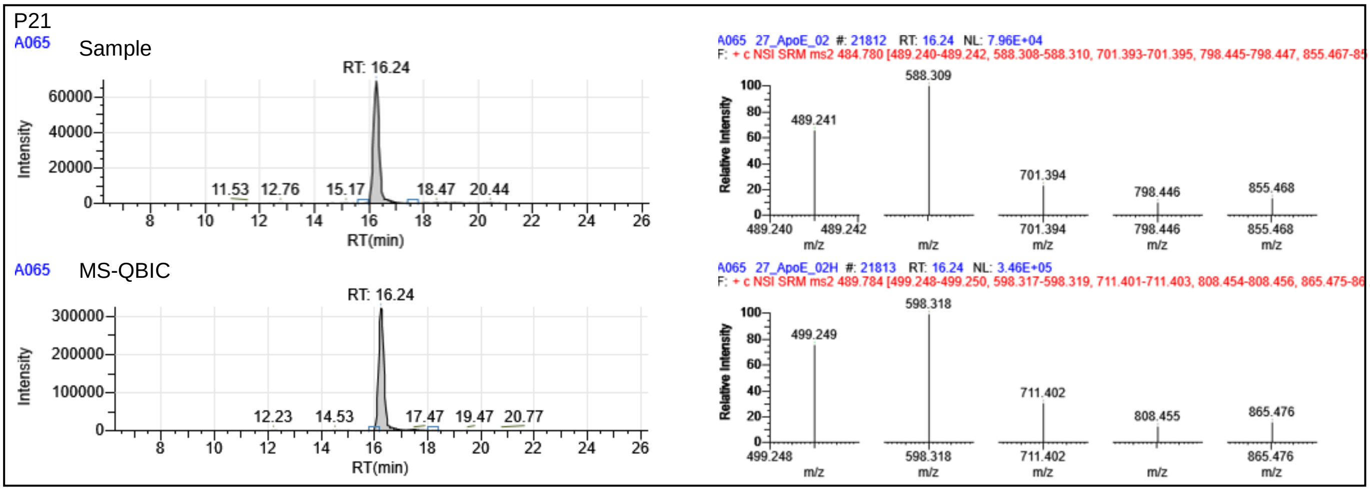
P21
A065 Sample
RT: 16.24
11.53 12.76 15.17 18.47 20.44
Intensity
RT(min)
A065 27_ApoE_02 #: 21812 RT: 16.24 NL: 7.96E+04
F: + c NSI SRM ms2 484.780 [489.240-489.242, 588.308-588.310, 701.393-701.395, 798.445-798.447, 855.467-85
489.241
588.309
701.394
798.446
855.468
Relative Intensity
m/z
A065 MS-QBIC
RT: 16.24
12.23 14.53 17.47 19.47 20.77
Intensity
RT(min)
A065 27_ApoE_02H #: 21813 RT: 16.24 NL: 3.46E+05
F: + c NSI SRM ms2 489.784 [499.248-499.250, 598.317-598.319, 711.401-711.403, 808.454-808.456, 865.475-86
499.249
598.318
711.402
808.455
865.476
Relative Intensity
m/z

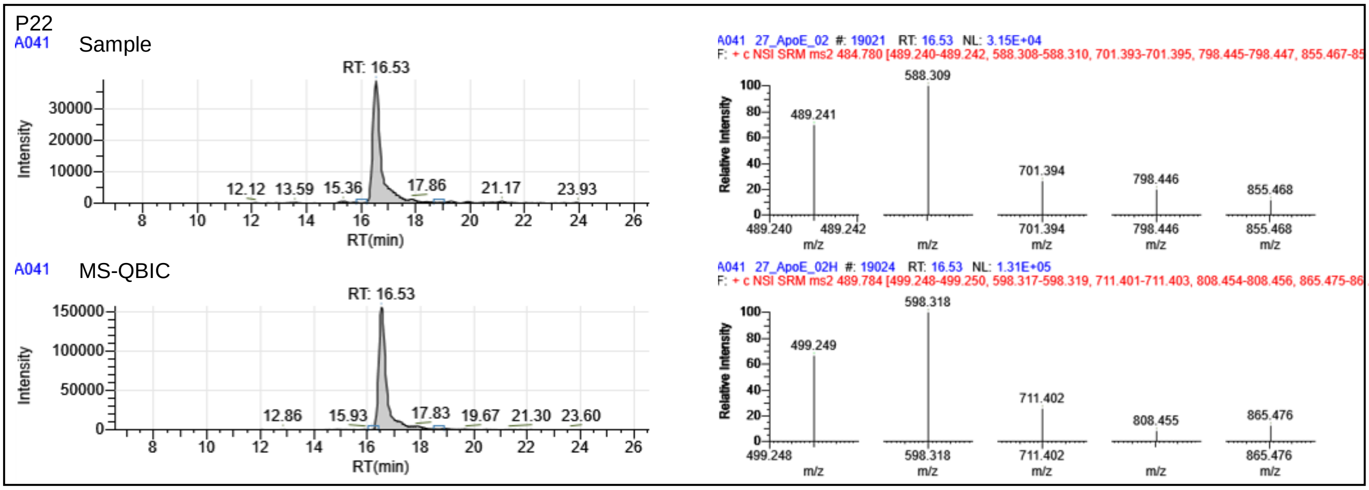
P22
A041 Sample
RT: 16.53
12.12 13.59 15.36 17.86 21.17 23.93
Intensity
RT(min)
A041 27_ApoE_02 #: 19021 RT: 16.53 NL: 3.15E+04
F: + c NSI SRM ms2 484.780 [489.240-489.242, 588.308-588.310, 701.393-701.395, 798.445-798.447, 855.467-85
489.241
588.309
701.394
798.446
855.468
Relative Intensity
m/z
A041 MS-QBIC
RT: 16.53
12.86 15.93 17.83 19.67 21.30 23.60
Intensity
RT(min)
A041 27_ApoE_02H #: 19024 RT: 16.53 NL: 1.31E+05
F: + c NSI SRM ms2 489.784 [499.248-499.250, 598.317-598.319, 711.401-711.403, 808.454-808.456, 865.475-86
499.249
598.318
711.402
808.455
865.476
Relative Intensity
m/z

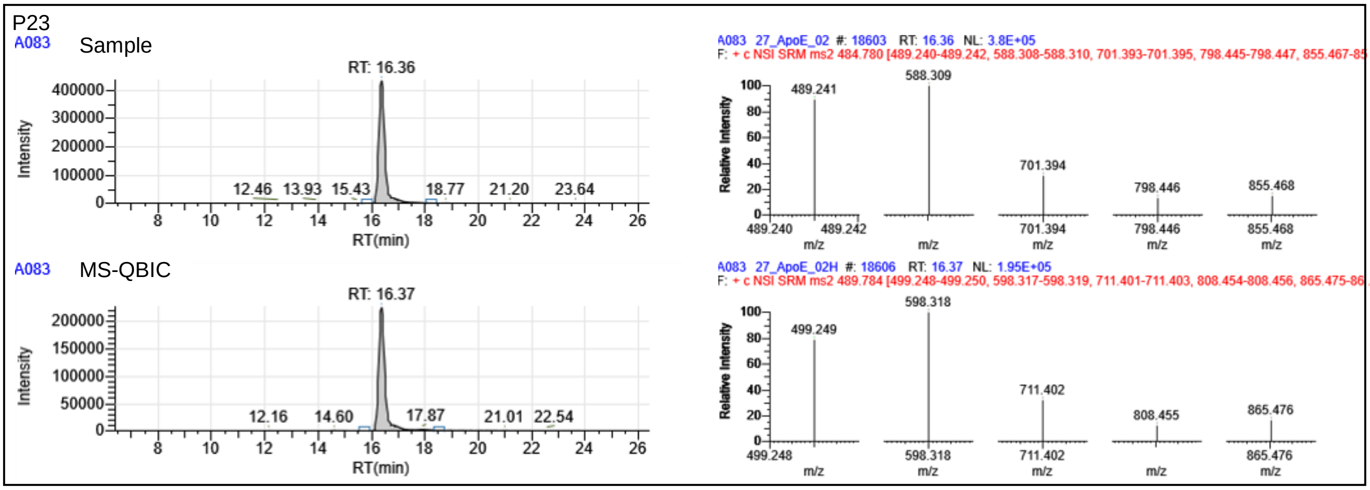
P23
A083 Sample
RT: 16.36
12.46 13.93 15.43 18.77 21.20 23.64
Intensity
RT(min)
A083 27_ApoE_02 #: 18603 RT: 16.36 NL: 3.8E+05
F: + c NSI SRM ms2 484.780 [489.240-489.242, 588.308-588.310, 701.393-701.395, 798.445-798.447, 855.467-85
489.241
588.309
701.394
798.446
855.468
Relative Intensity
m/z
A083 MS-QBIC
RT: 16.37
12.16 14.60 17.87 21.01 22.54
Intensity
RT(min)
A083 27_ApoE_02H #: 18606 RT: 16.37 NL: 1.95E+05
F: + c NSI SRM ms2 489.784 [499.248-499.250, 598.317-598.319, 711.401-711.403, 808.454-808.456, 865.475-86
499.249
598.318
711.402
808.455
865.476
Relative Intensity
m/z

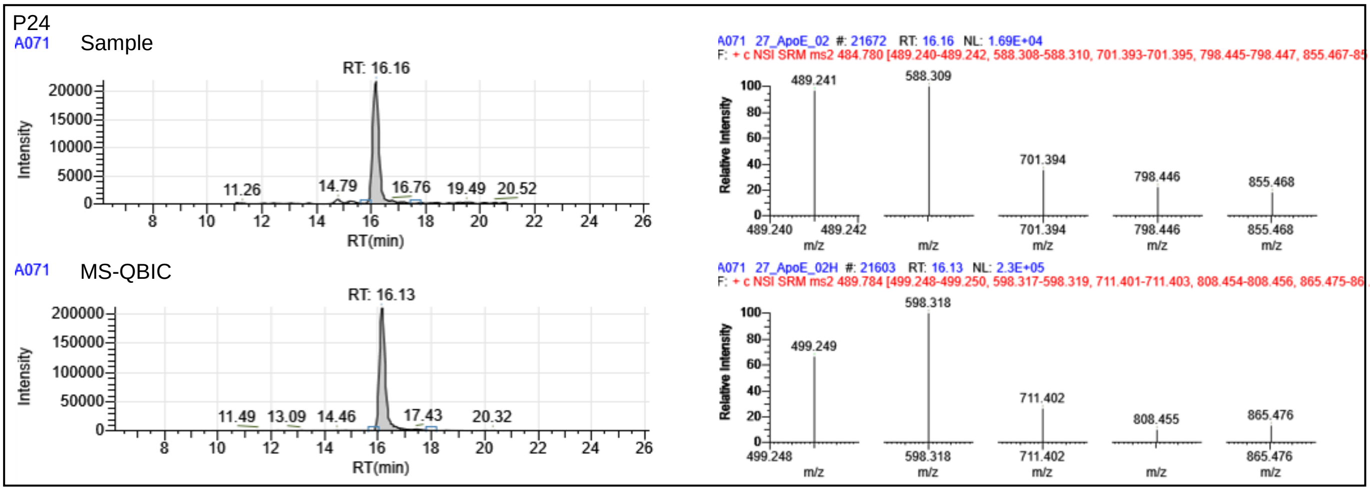
P24
A071 Sample
RT: 16.16
11.26 14.79 16.76 19.49 20.52
Intensity
RT(min)
A071 27_ApoE_02 #: 21672 RT: 16.16 NL: 1.69E+04
F: + c NSI SRM ms2 484.780 [489.240-489.242, 588.308-588.310, 701.393-701.395, 798.445-798.447, 855.467-85
489.241
588.309
701.394
798.446
855.468
Relative Intensity
m/z
A071 MS-QBIC
RT: 16.13
11.49 13.09 14.46 17.43 20.32
Intensity
RT(min)
A071 27_ApoE_02H #: 21603 RT: 16.13 NL: 2.3E+05
F: + c NSI SRM ms2 489.784 [499.248-499.250, 598.317-598.319, 711.401-711.403, 808.454-808.456, 865.475-86
499.249
598.318
711.402
808.455
865.476
Relative Intensity
m/z

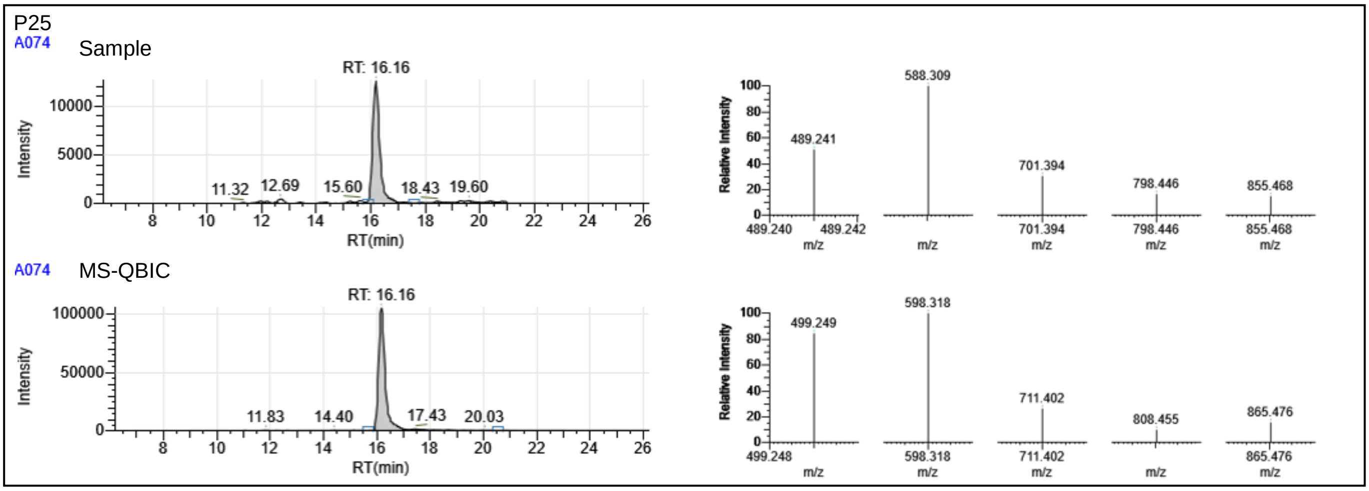
P25
A074 Sample
RT: 16.16
11.32 12.69 15.60 18.43 19.60
Intensity
RT(min)
Relative Intensity
489.241
588.309
701.394
798.446
855.468
m/z
A074 MS-QBIC
RT: 16.16
11.83 14.40 17.43 20.03
499.249
598.318
711.402
808.455
865.476

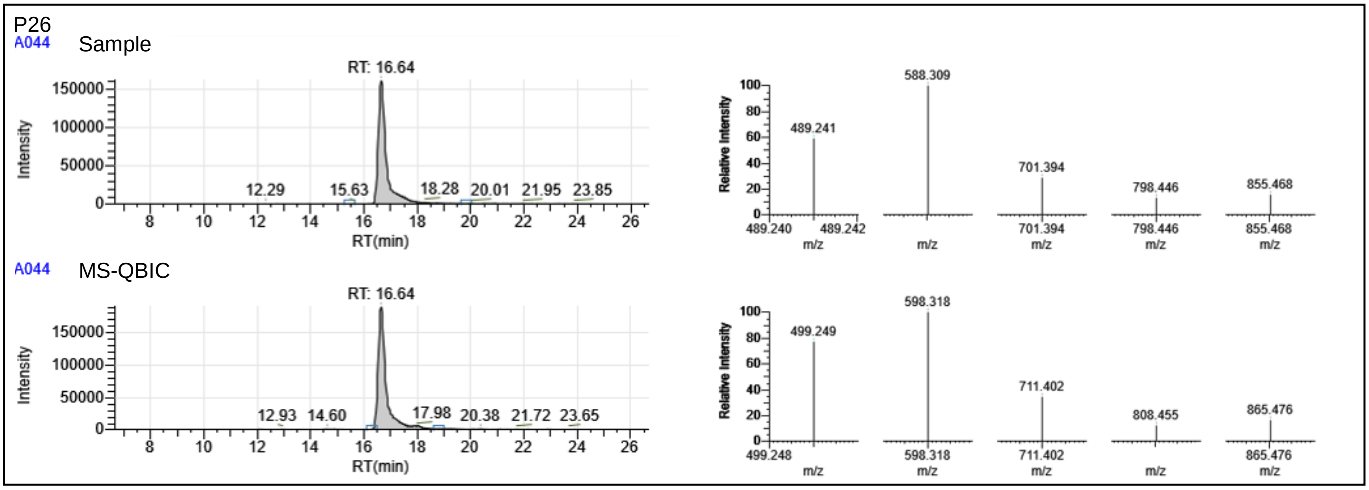
P26
A044 Sample
RT: 16.64
12.29 15.63 18.28 20.01 21.95 23.85
Intensity
RT(min)
Relative Intensity
489.241
588.309
701.394
798.446
855.468
m/z
A044 MS-QBIC
RT: 16.64
12.93 14.60 17.98 20.38 21.72 23.65
499.249
598.318
711.402
808.455
865.476

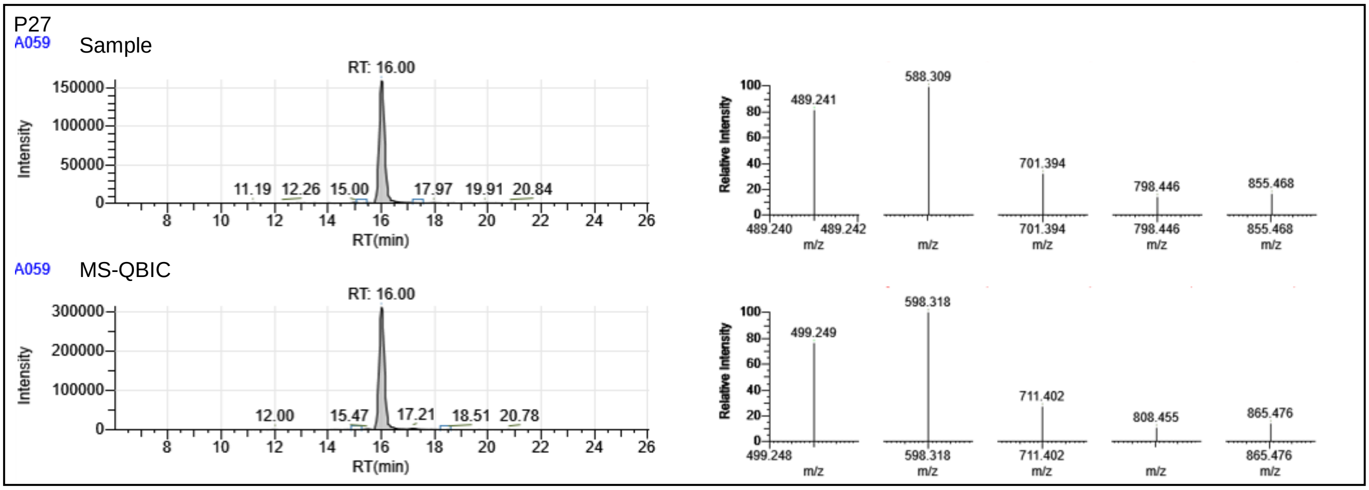
P27
A059 Sample
RT: 16.00
11.19 12.26 15.00 17.97 19.91 20.84
Intensity
RT(min)
Relative Intensity
489.241
588.309
701.394
798.446
855.468
m/z
A059 MS-QBIC
RT: 16.00
12.00 15.47 17.21 18.51 20.78
499.249
598.318
711.402
808.455
865.476

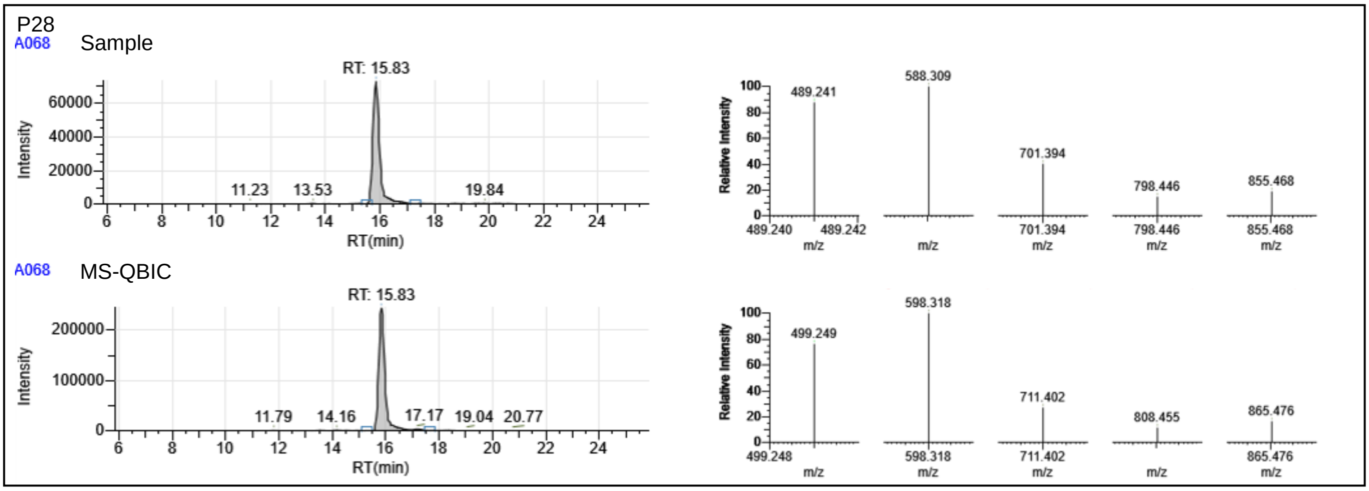
P28
A068 Sample
RT: 15.83
11.23 13.53 19.84
Intensity
RT(min)
Relative Intensity
489.241
588.309
701.394
798.446
855.468
m/z
A068 MS-QBIC
RT: 15.83
11.79 14.16 17.17 19.04 20.77
499.249
598.318
711.402
808.455
865.476

P29

A056 Sample

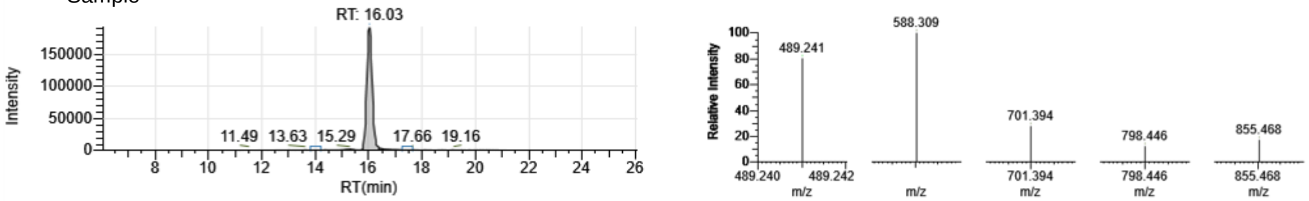

A056 MS-QBIC

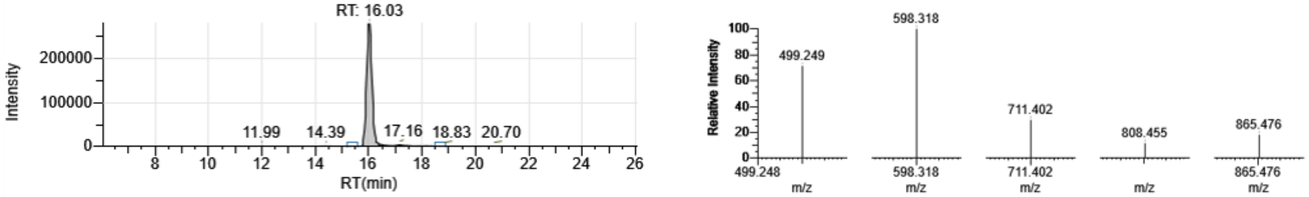

P30

A080 Sample

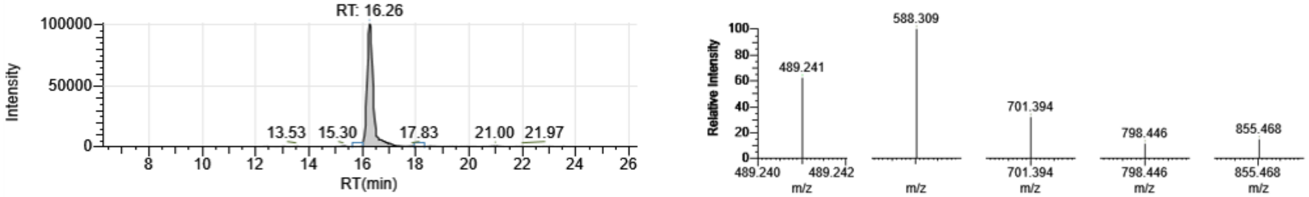

A080 MS-QBIC

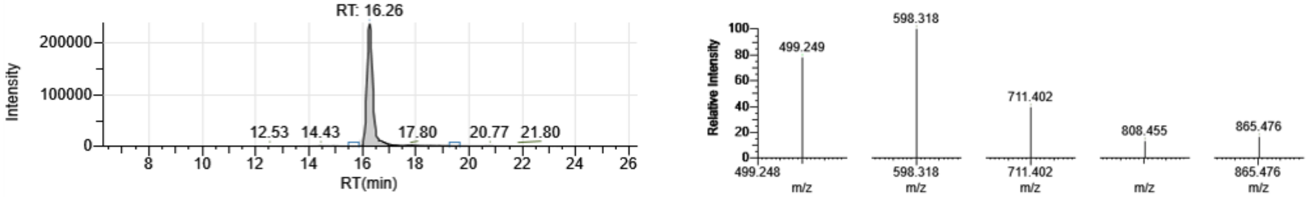

J Gelsolin (EPGLQIWR) Heart

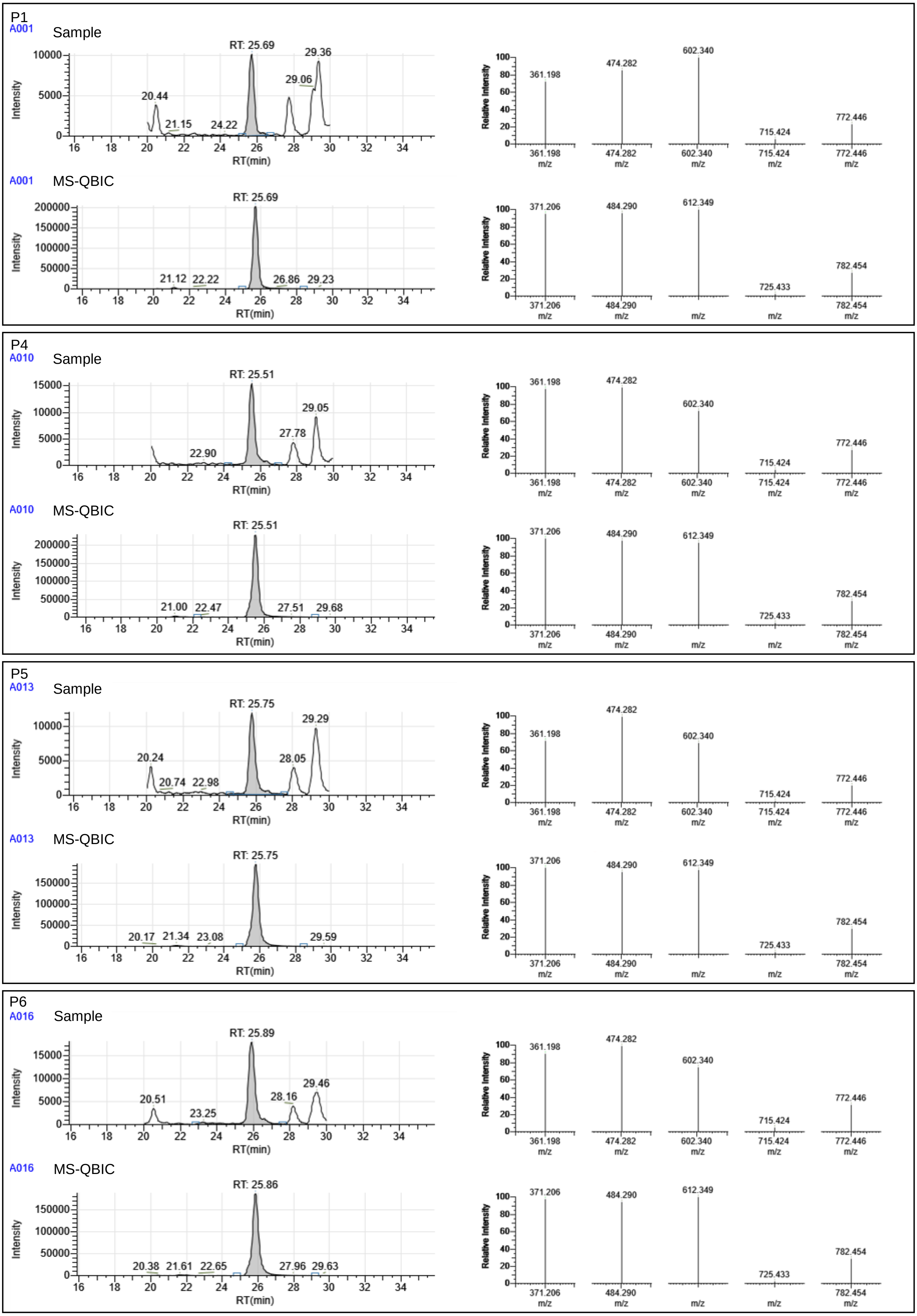

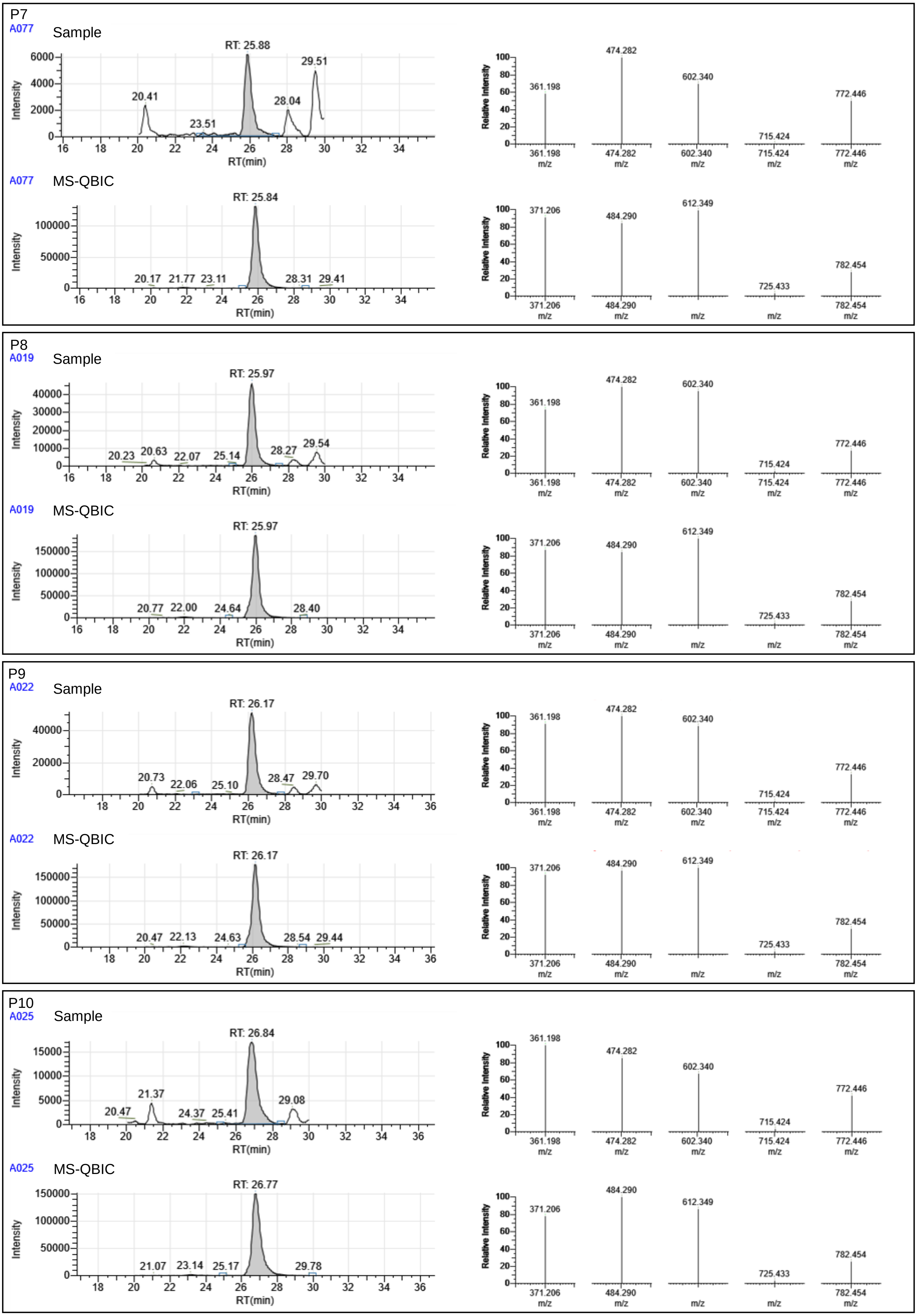
P7
A077 Sample
RT: 25.88
20.41
23.51
28.04
29.51
A077 MS-QBIC
RT: 25.84
20.17 21.77 23.11
28.31 29.41
361.198
474.282
602.340
715.424
772.446
371.206
484.290
612.349
725.433
782.454
P8
A019 Sample
RT: 25.97
20.23 20.63 22.07 25.14
28.27 29.54
A019 MS-QBIC
RT: 25.97
20.77 22.00 24.64
28.40
P9
A022 Sample
RT: 26.17
20.73 22.06 25.10
28.47 29.70
A022 MS-QBIC
RT: 26.17
20.47 22.13 24.63
28.54 29.44
P10
A025 Sample
RT: 26.84
20.47 21.37 24.37 25.41
29.08
A025 MS-QBIC
RT: 26.77
21.07 23.14 25.17
29.78
Intensity
RT(min)
Relative Intensity
m/z

P11

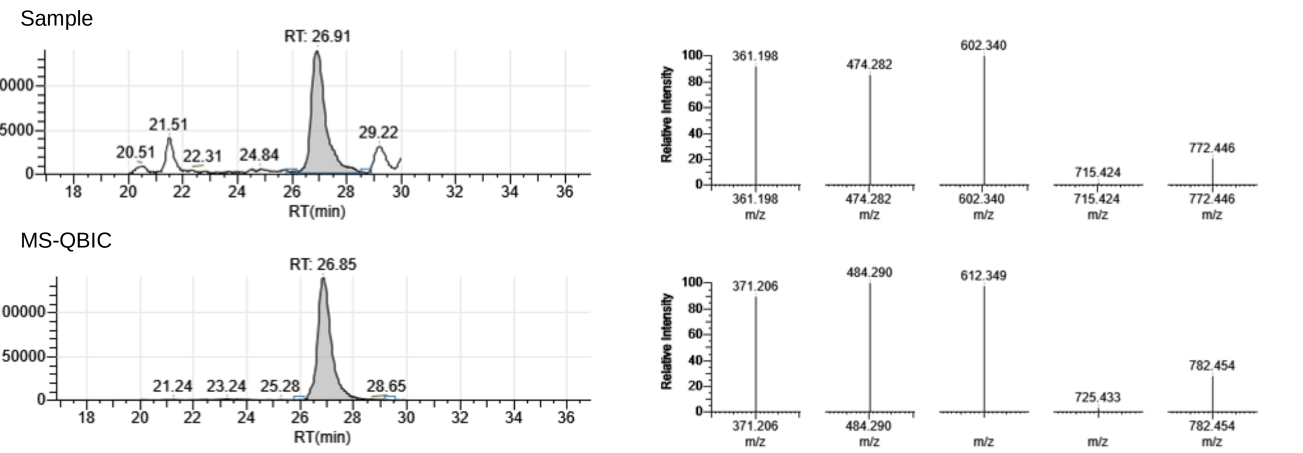

P12

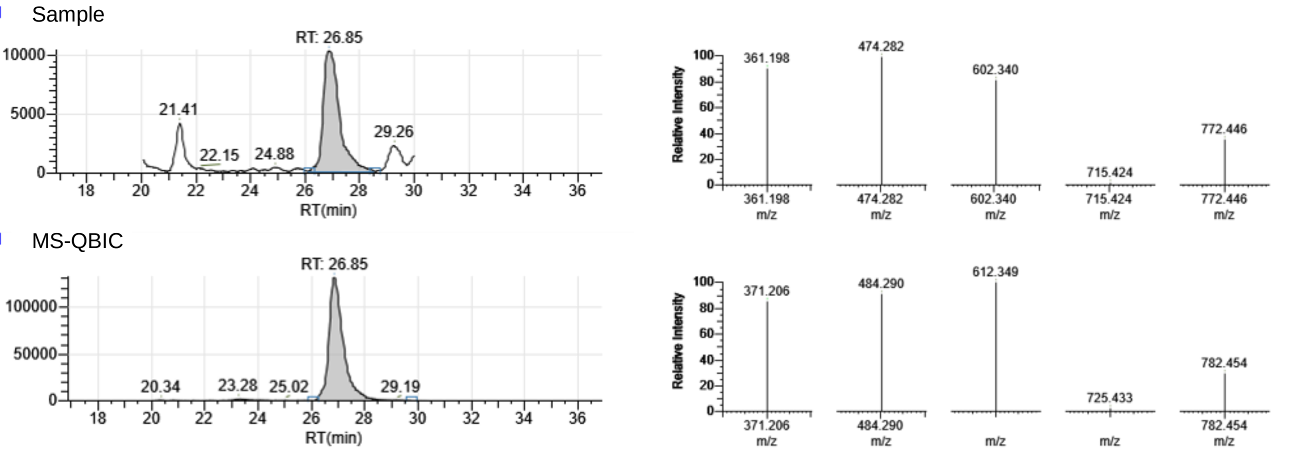

P13

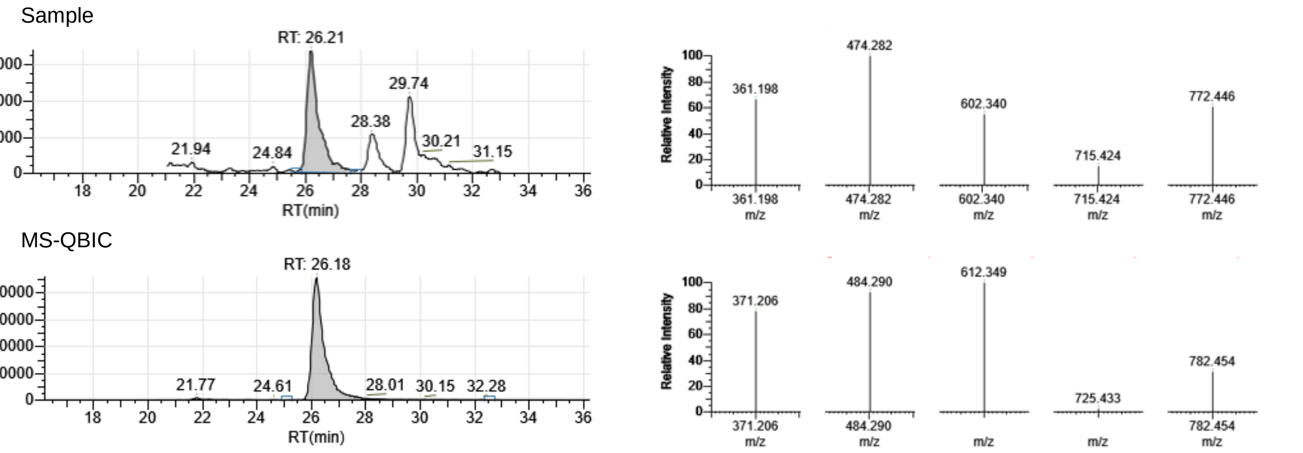

P14

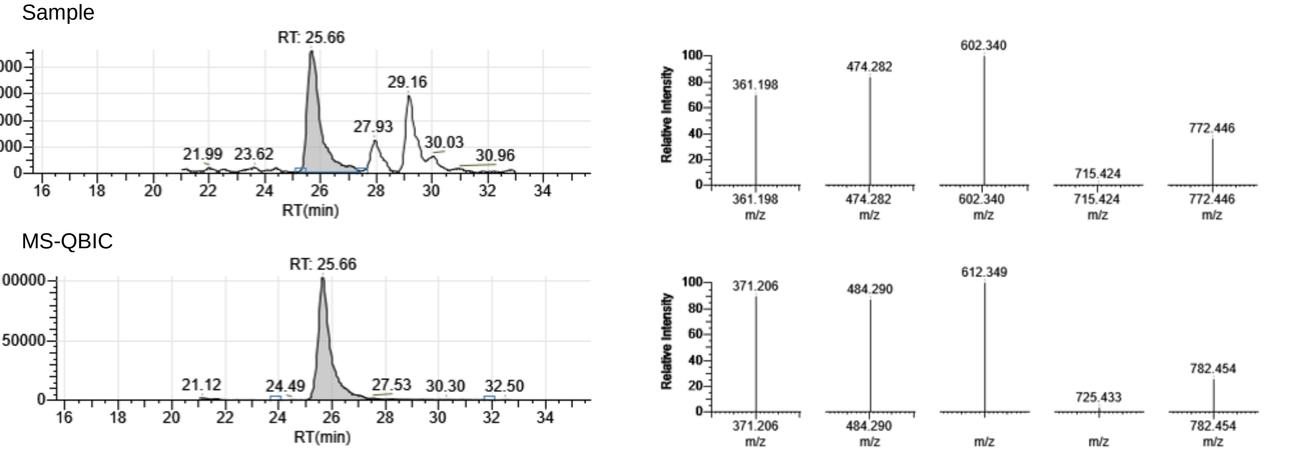

P15

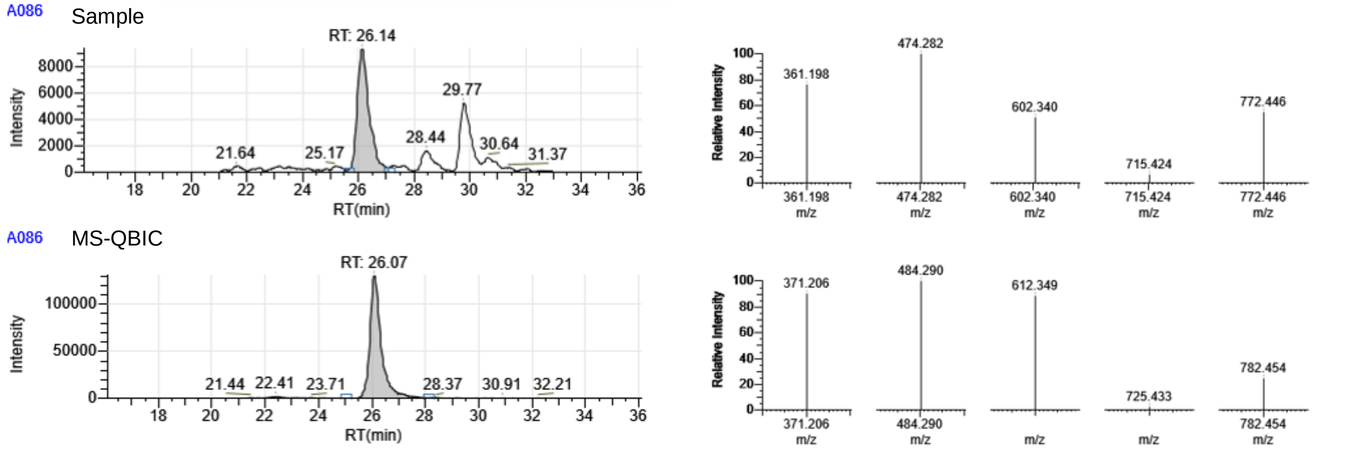

P16

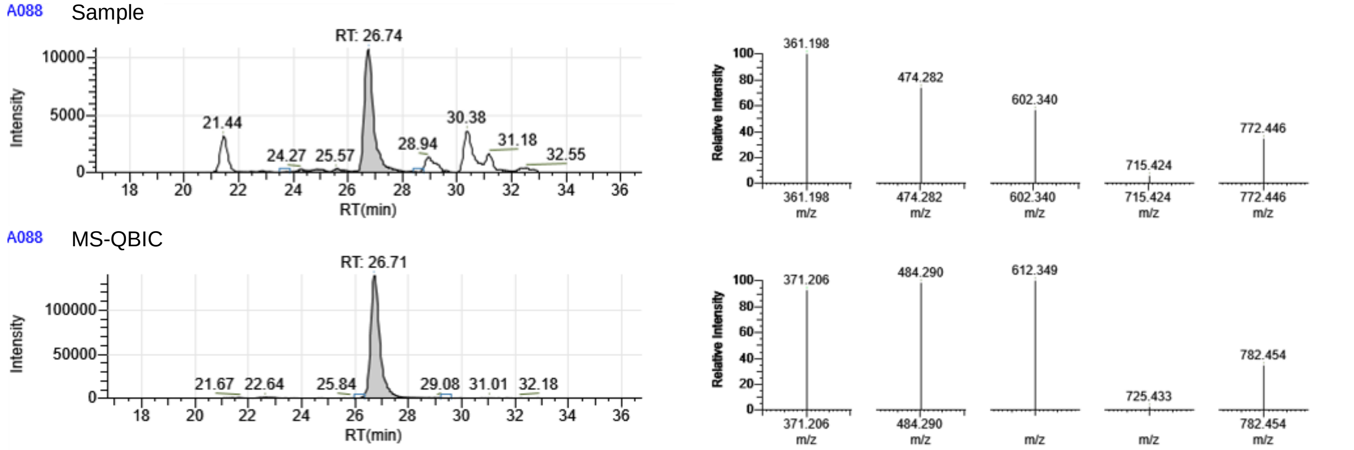

P17

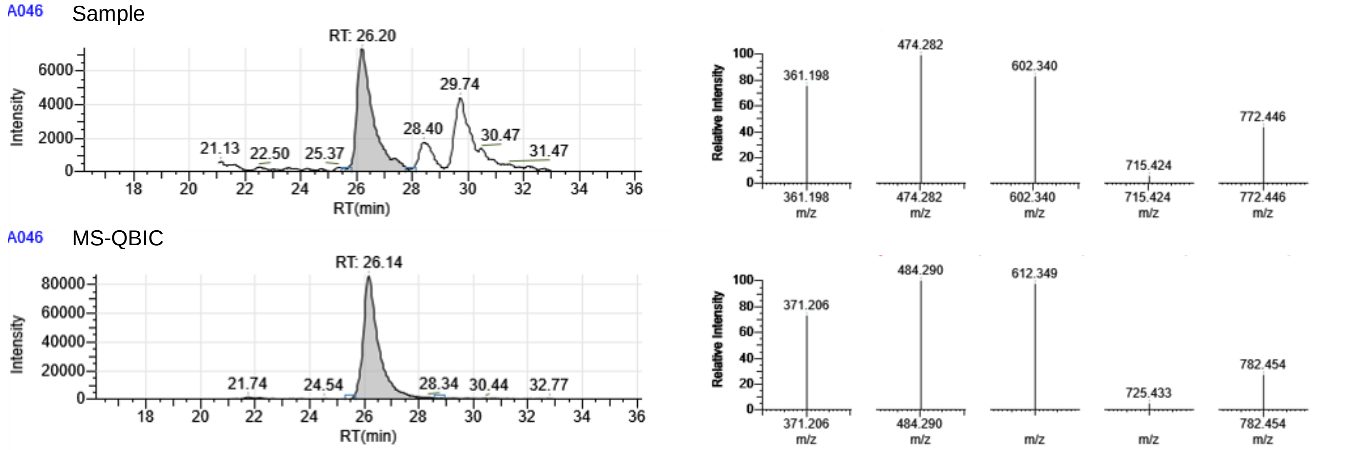

P18

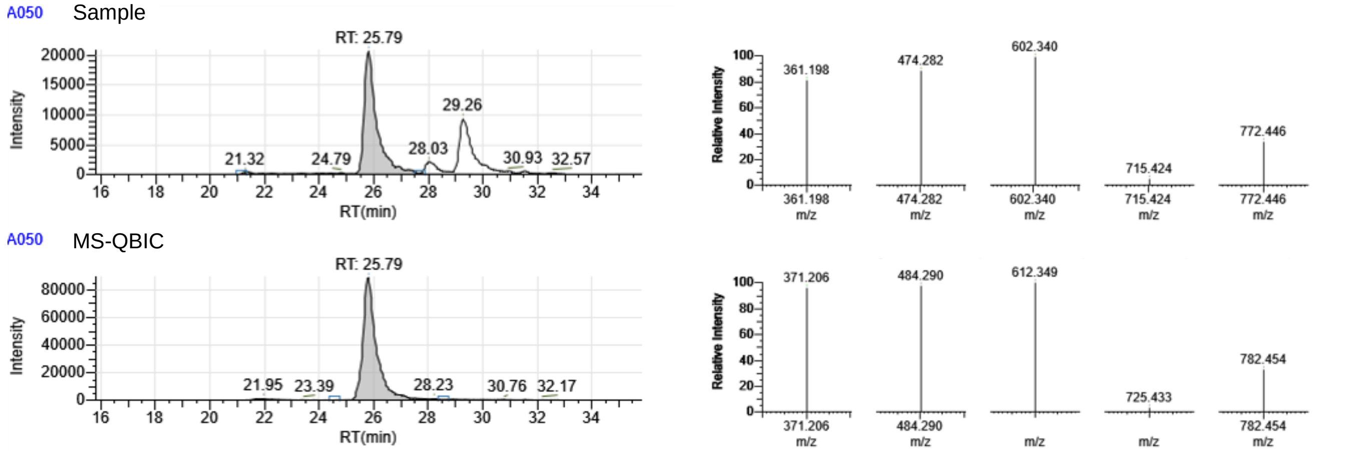

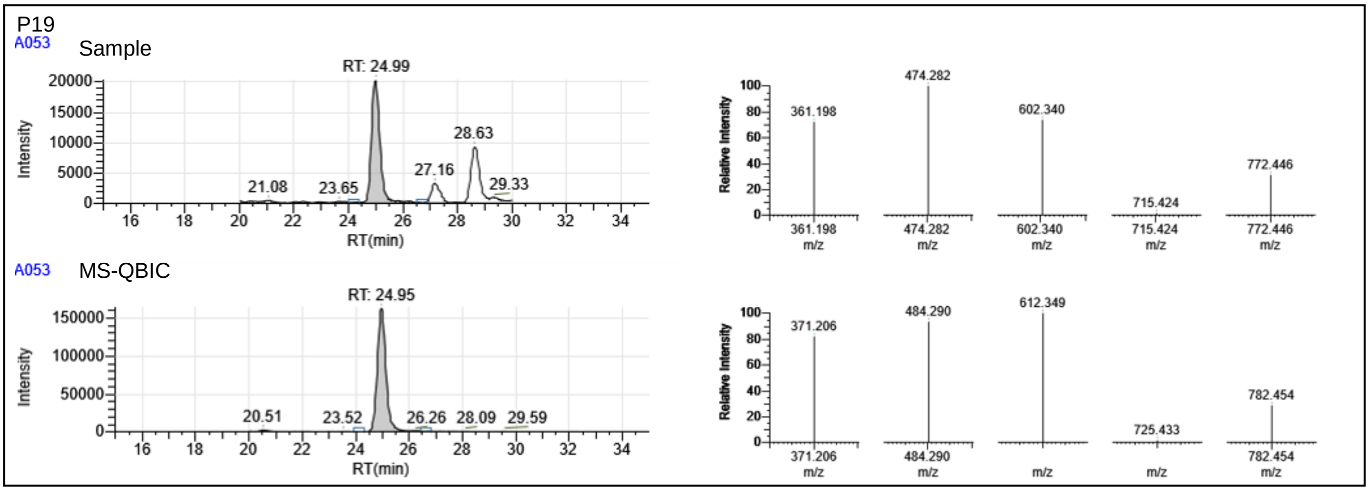
P19
A053 Sample
RT: 24.99
21.08
23.65
27.16
28.63
29.33
Intensity
RT(min)
361.198
474.282
602.340
715.424
772.446
Relative Intensity
m/z
A053 MS-QBIC
RT: 24.95
20.51
23.52
26.26
28.09
29.59
371.206
484.290
612.349
725.433
782.454

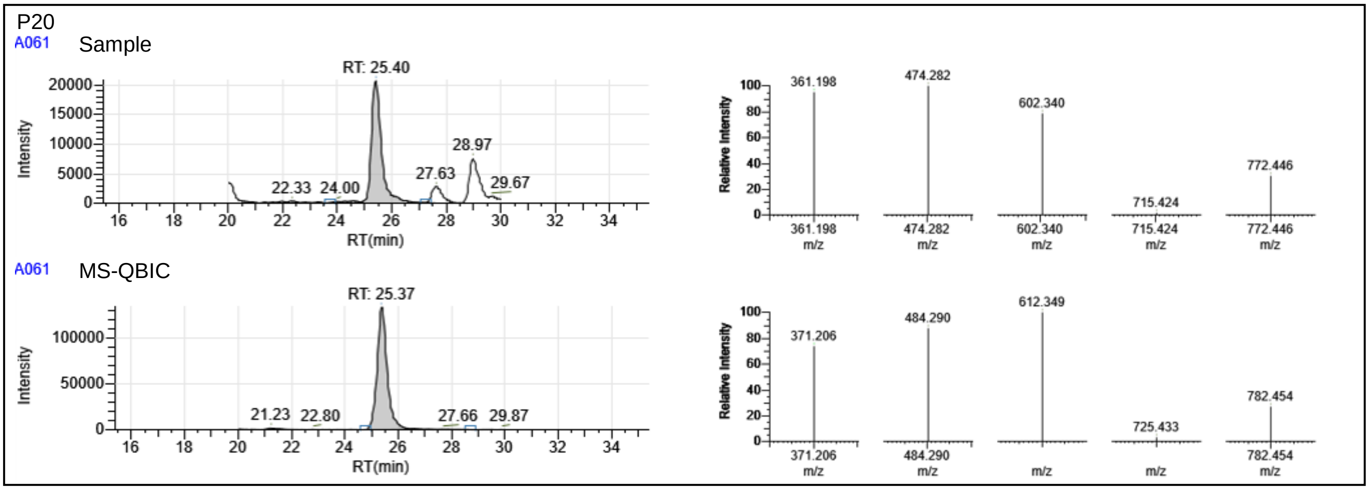
P20
A061 Sample
RT: 25.40
22.33
24.00
27.63
28.97
29.67
Intensity
RT(min)
361.198
474.282
602.340
715.424
772.446
Relative Intensity
m/z
A061 MS-QBIC
RT: 25.37
21.23
22.80
27.66
29.87
371.206
484.290
612.349
725.433
782.454

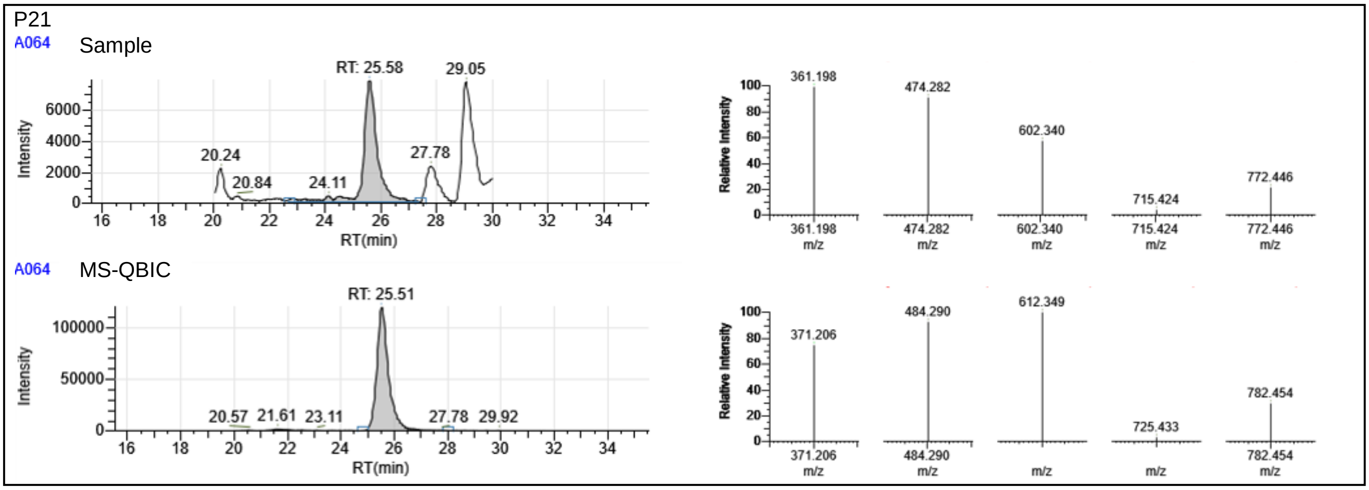
P21
A064 Sample
RT: 25.58
29.05
20.24
20.84
24.11
27.78
Intensity
RT(min)
361.198
474.282
602.340
715.424
772.446
Relative Intensity
m/z
A064 MS-QBIC
RT: 25.51
20.57
21.61
23.11
27.78
29.92
371.206
484.290
612.349
725.433
782.454

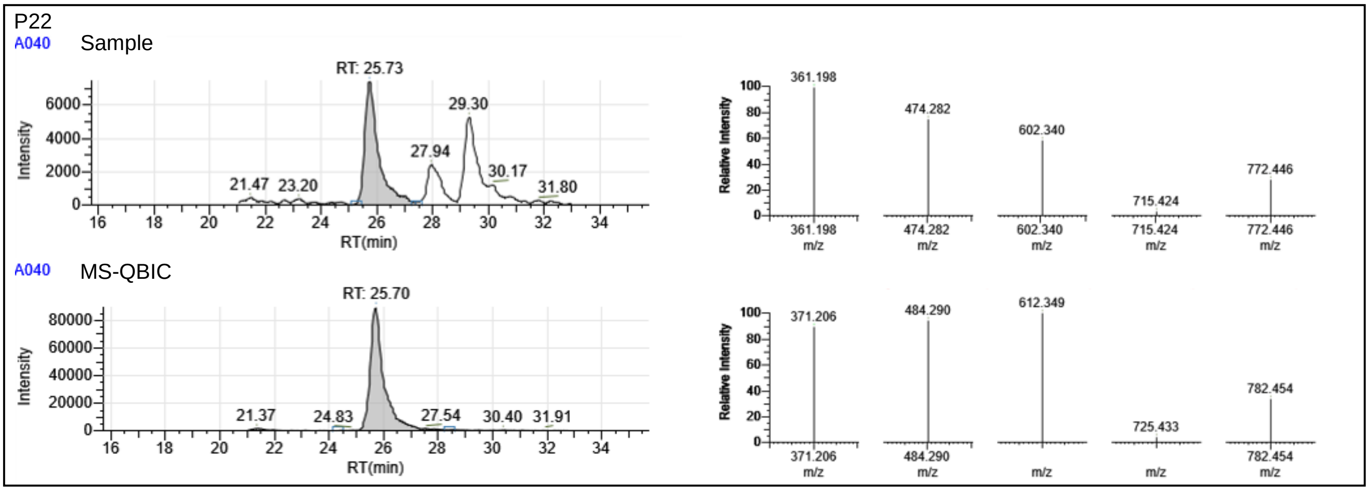
P22
A040 Sample
RT: 25.73
29.30
27.94
21.47
23.20
30.17
31.80
Intensity
RT(min)
361.198
474.282
602.340
715.424
772.446
Relative Intensity
m/z
A040 MS-QBIC
RT: 25.70
21.37
24.83
27.54
30.40
31.91
371.206
484.290
612.349
725.433
782.454

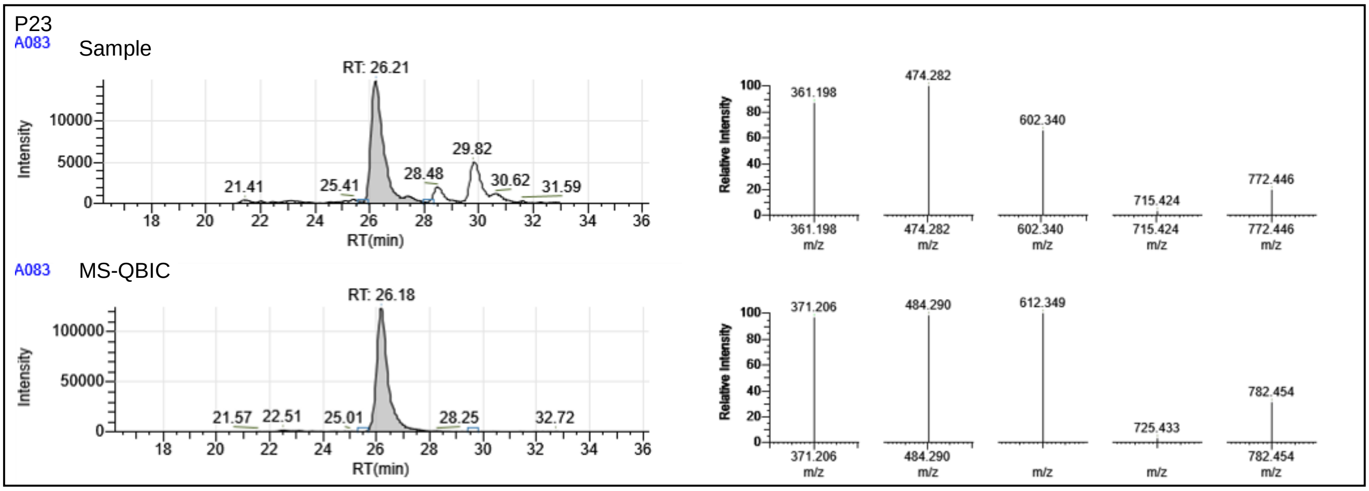

P23
A083
Sample
RT: 26.21
21.41
25.41
28.48
29.82
30.62
31.59
Intensity
RT(min)
361.198
474.282
602.340
715.424
772.446
Relative Intensity
m/z
A083
MS-QBIC
RT: 26.18
21.57
22.51
25.01
28.25
32.72
371.206
484.290
612.349
725.433
782.454

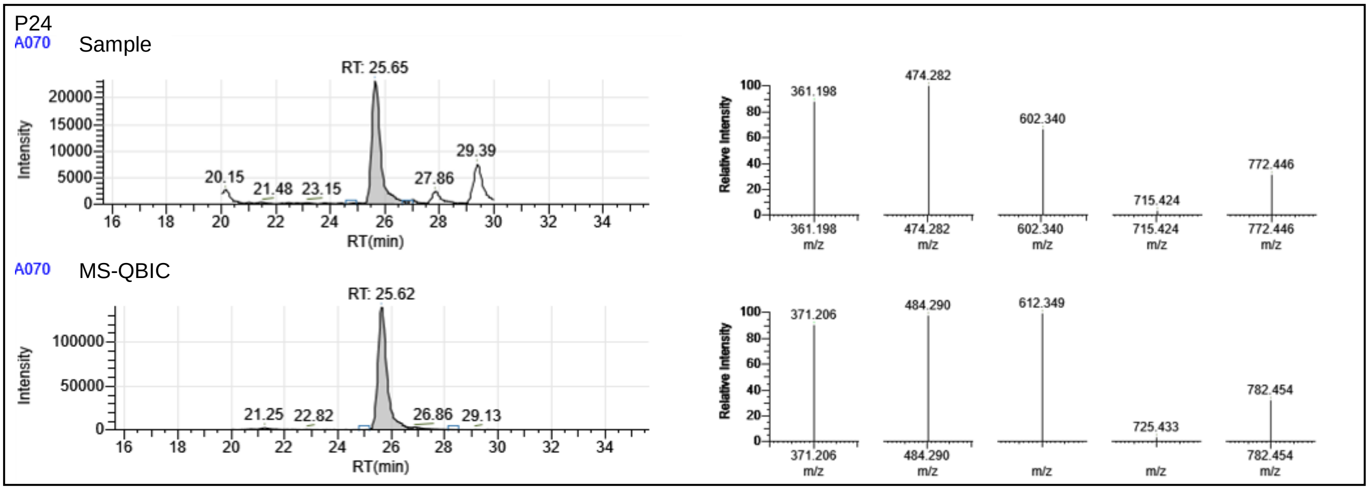

P24
A070
Sample
RT: 25.65
20.15
21.48
23.15
27.86
29.39
Intensity
RT(min)
361.198
474.282
602.340
715.424
772.446
Relative Intensity
m/z
A070
MS-QBIC
RT: 25.62
21.25
22.82
26.86
29.13
371.206
484.290
612.349
725.433
782.454

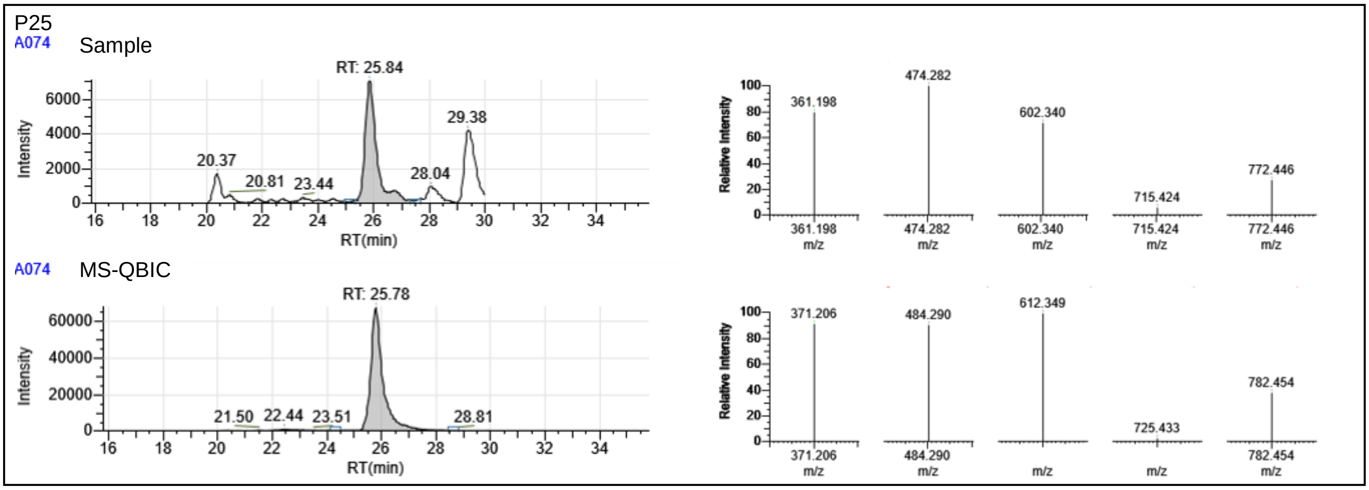

P25
A074
Sample
RT: 25.84
20.37
20.81
23.44
28.04
29.38
Intensity
RT(min)
361.198
474.282
602.340
715.424
772.446
Relative Intensity
m/z
A074
MS-QBIC
RT: 25.78
21.50
22.44
23.51
28.81
371.206
484.290
612.349
725.433
782.454

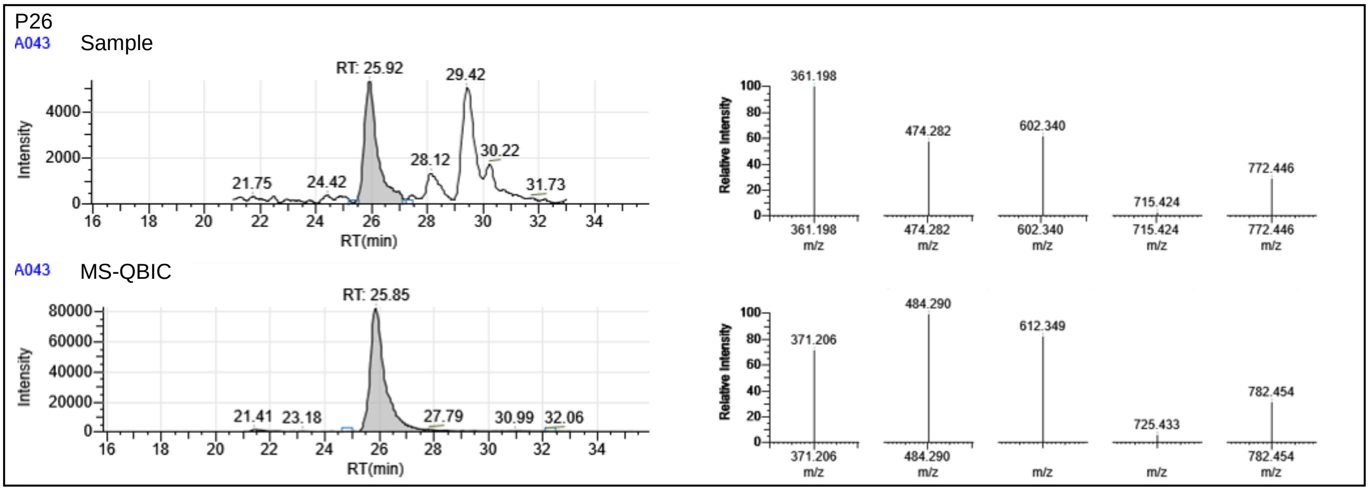

P26
A043
Sample
RT: 25.92
29.42
21.75
24.42
28.12
30.22
31.73
Intensity
RT(min)
361.198
474.282
602.340
715.424
772.446
Relative Intensity
m/z
A043
MS-QBIC
RT: 25.85
21.41
23.18
27.79
30.99
32.06
371.206
484.290
612.349
725.433
782.454

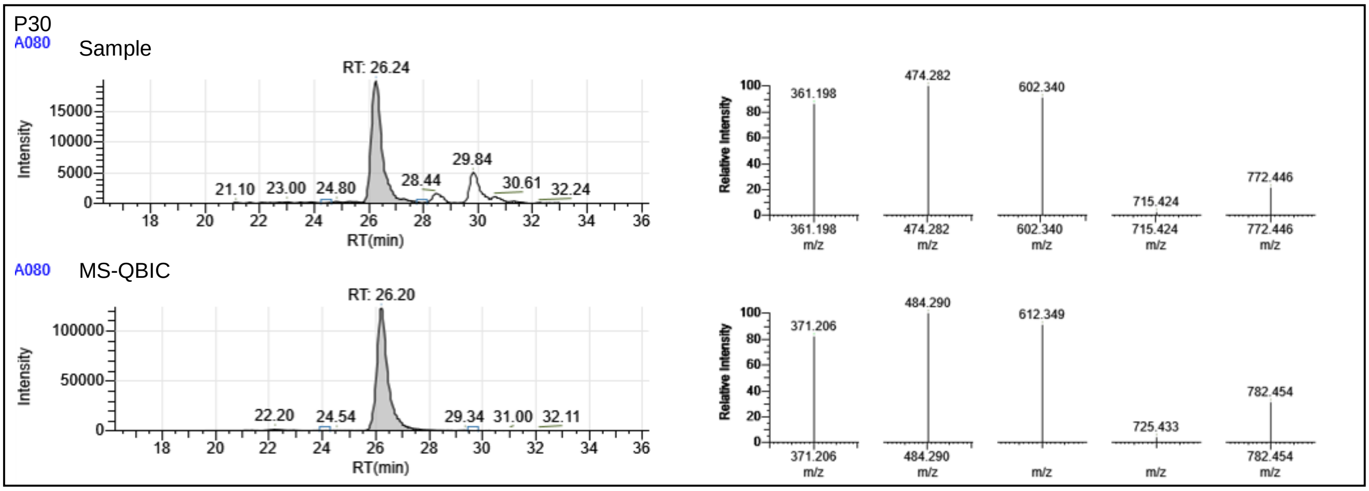

P30
A080
Sample
RT: 26.24
Intensity
RT(min)
21.10
23.00
24.80
28.44
29.84
30.61
32.24
Relative Intensity
361.198
474.282
602.340
715.424
772.446
m/z
A080
MS-QBIC
RT: 26.20
22.20
24.54
29.34
31.00
32.11
371.206
484.290
612.349
725.433
782.454

K. FGA (GGSTSYGTGSETESPR) Heart

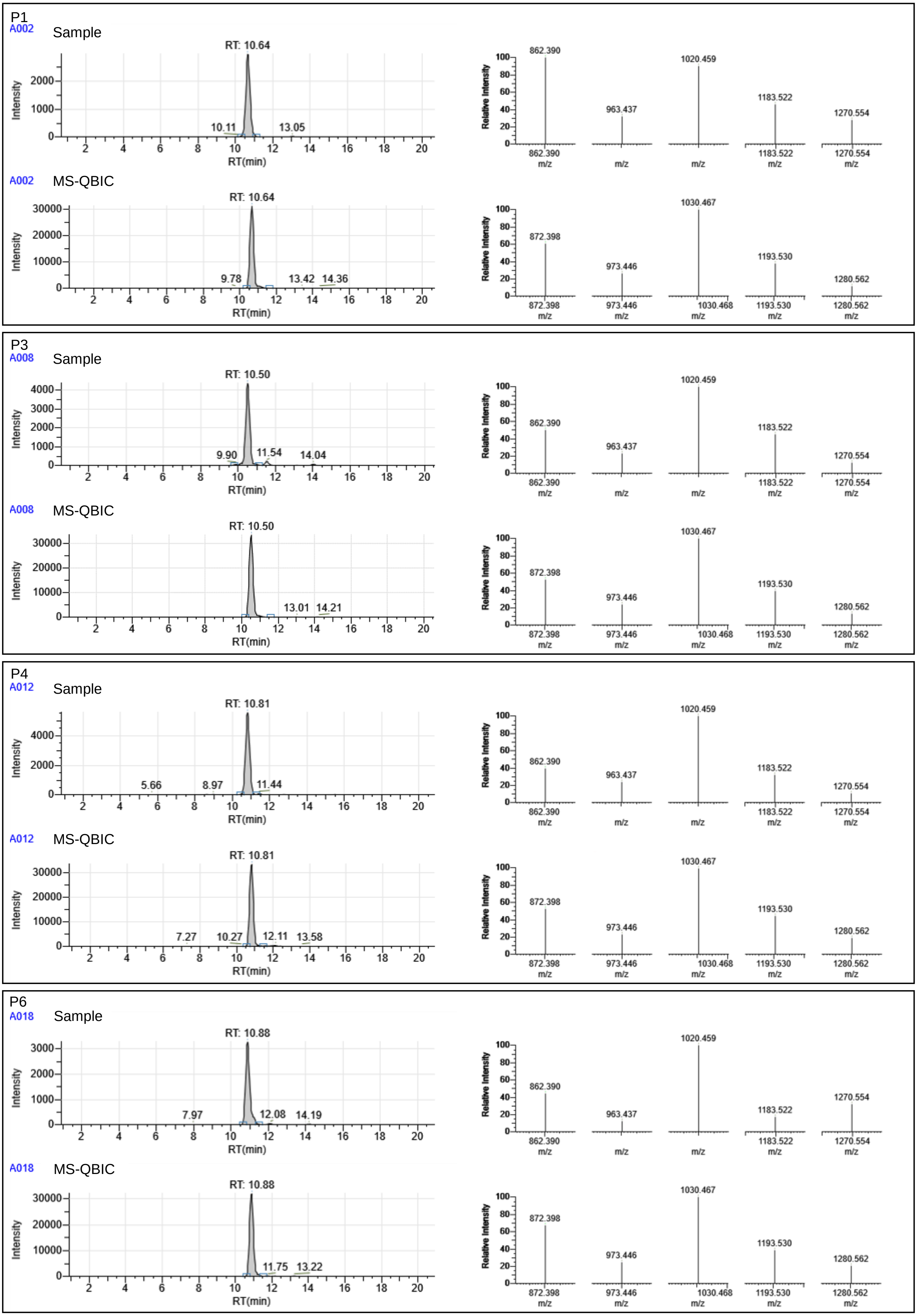

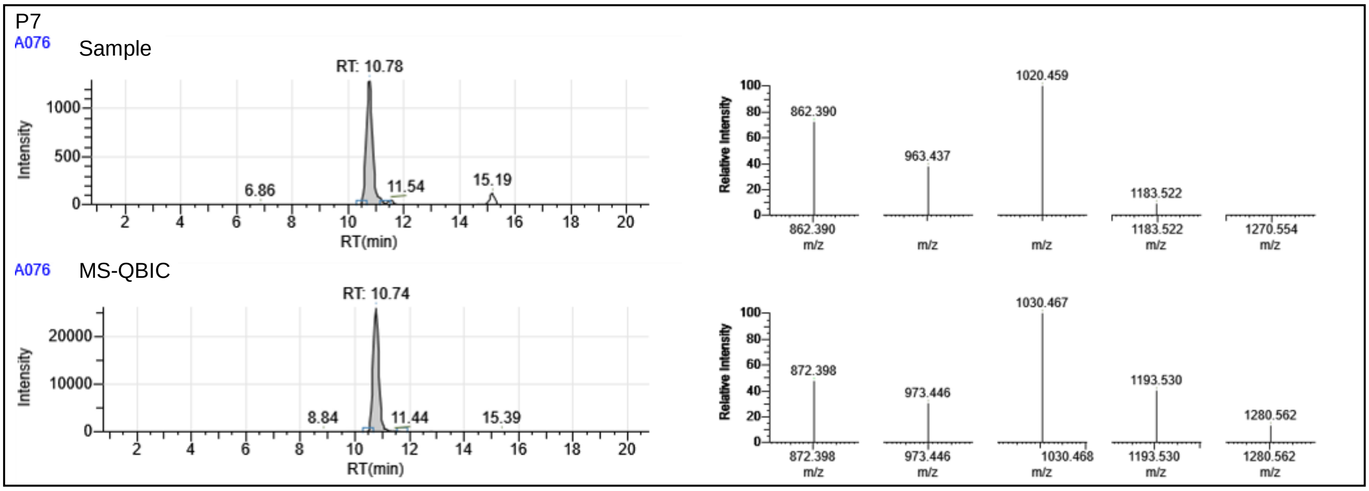
P7
A076 Sample
RT: 10.78
6.86
11.54
15.19
Intensity
RT(min)
Relative Intensity
862.390
963.437
1020.459
1183.522
1270.554
m/z
A076 MS-QBIC
RT: 10.74
8.84
11.44
15.39
872.398
973.446
1030.467
1030.468
1193.530
1280.562

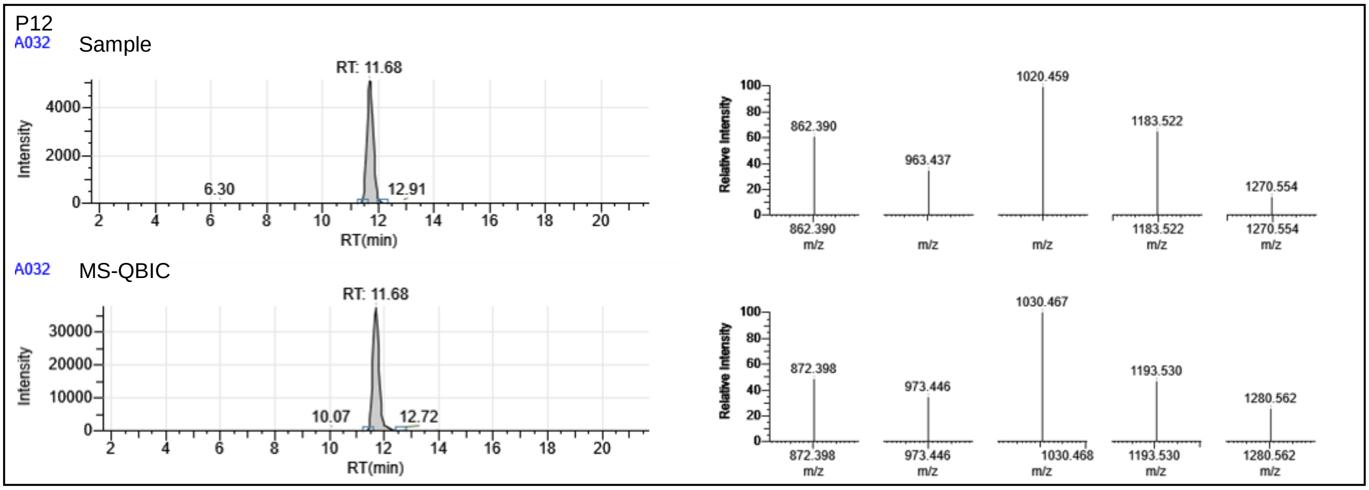
P12
A032 Sample
RT: 11.68
6.30
12.91
Intensity
RT(min)
Relative Intensity
862.390
963.437
1020.459
1183.522
1270.554
m/z
A032 MS-QBIC
RT: 11.68
10.07
12.72
872.398
973.446
1030.467
1030.468
1193.530
1280.562

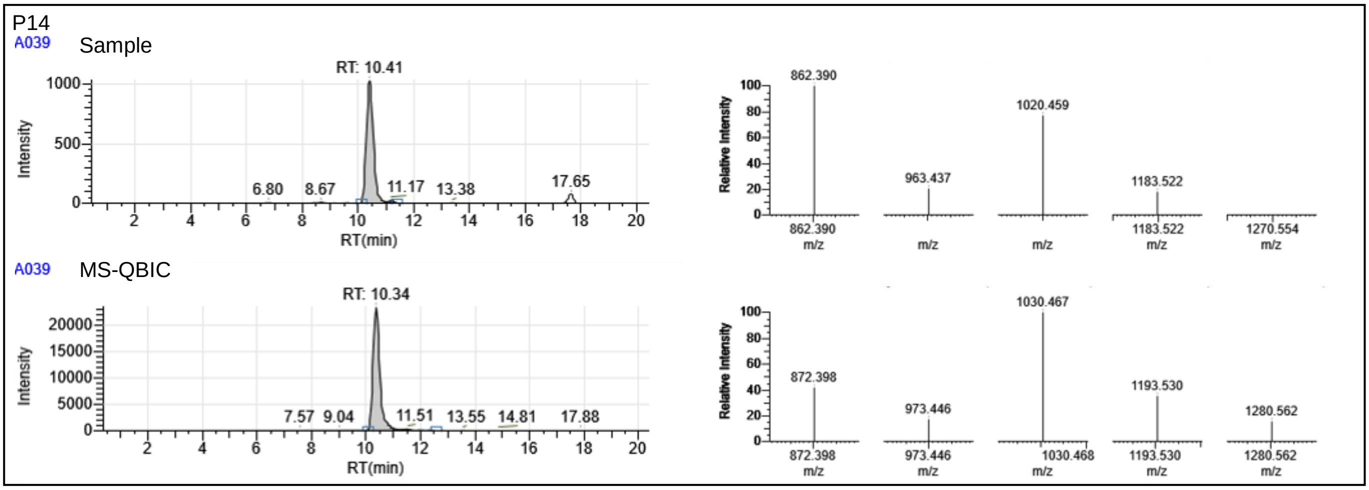
P14
A039 Sample
RT: 10.41
6.80
8.67
11.17
13.38
17.65
Intensity
RT(min)
Relative Intensity
862.390
963.437
1020.459
1183.522
1270.554
m/z
A039 MS-QBIC
RT: 10.34
7.57
9.04
11.51
13.55
14.81
17.88
872.398
973.446
1030.467
1030.468
1193.530
1280.562

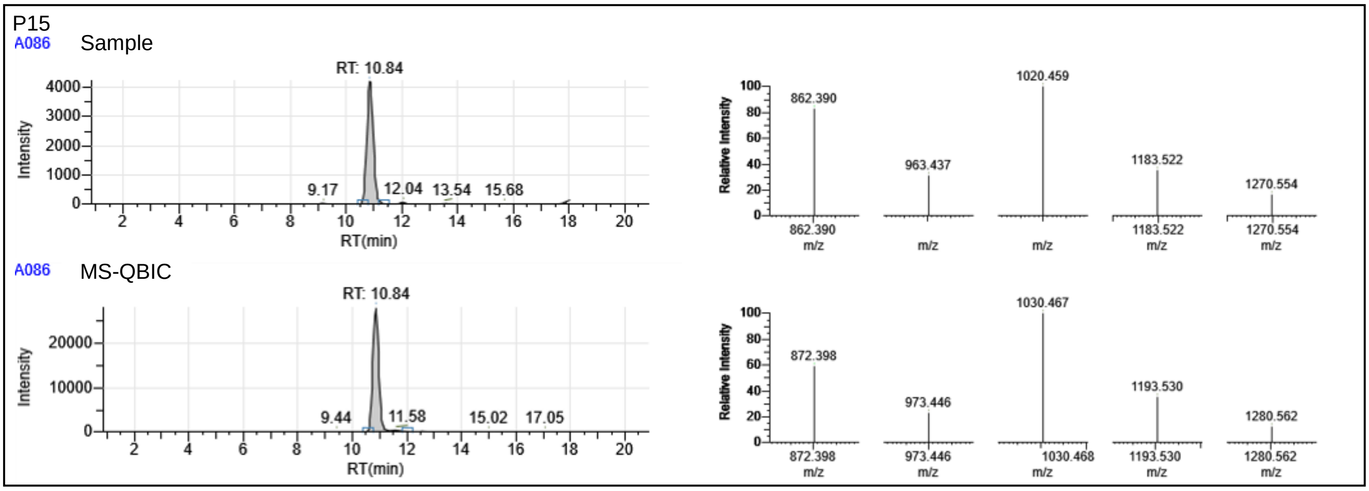
P15
A086 Sample
RT: 10.84
9.17
12.04
13.54
15.68
Intensity
RT(min)
Relative Intensity
862.390
963.437
1020.459
1183.522
1270.554
m/z
A086 MS-QBIC
RT: 10.84
9.44
11.58
15.02
17.05
872.398
973.446
1030.467
1030.468
1193.530
1280.562

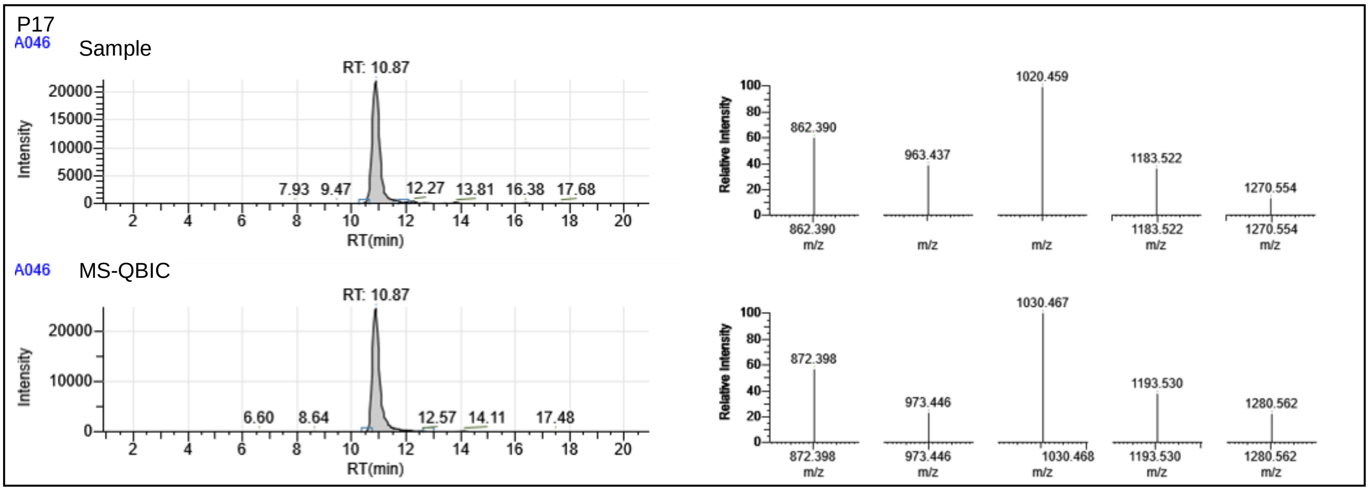
P17
A046 Sample
RT: 10.87
7.93 9.47 12.27 13.81 16.38 17.68
Intensity
RT(min)
862.390
963.437
1020.459
1183.522
1270.554
Relative Intensity
m/z
A046 MS-QBIC
RT: 10.87
6.60 8.64 12.57 14.11 17.48
872.398
973.446
1030.467
1030.468
1193.530
1280.562

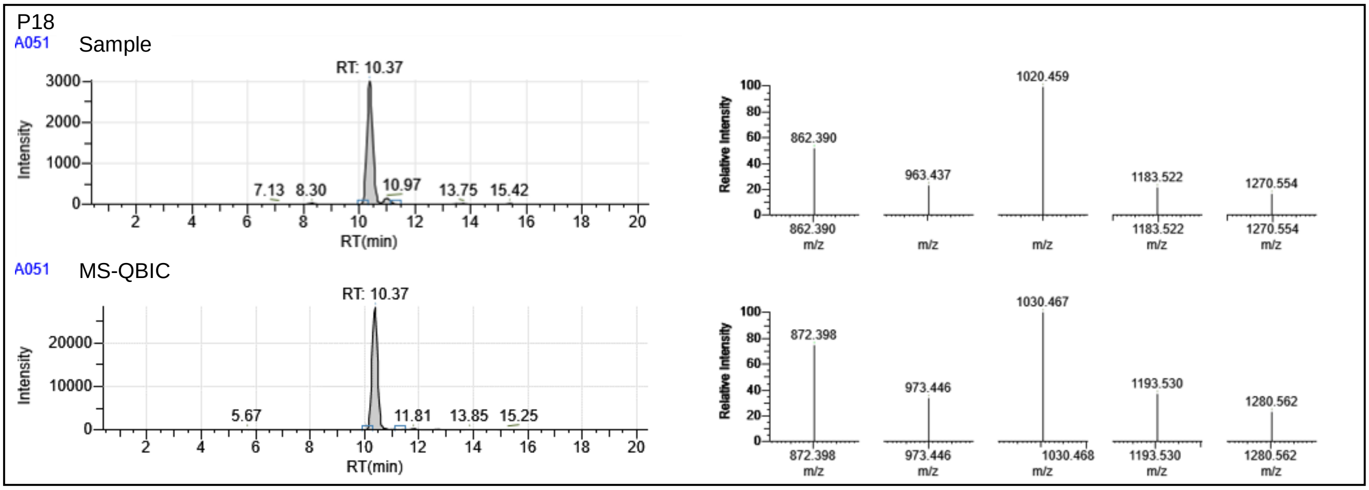
P18
A051 Sample
RT: 10.37
7.13 8.30 10.97 13.75 15.42
Intensity
RT(min)
862.390
963.437
1020.459
1183.522
1270.554
Relative Intensity
m/z
A051 MS-QBIC
RT: 10.37
5.67 11.81 13.85 15.25
872.398
973.446
1030.467
1030.468
1193.530
1280.562

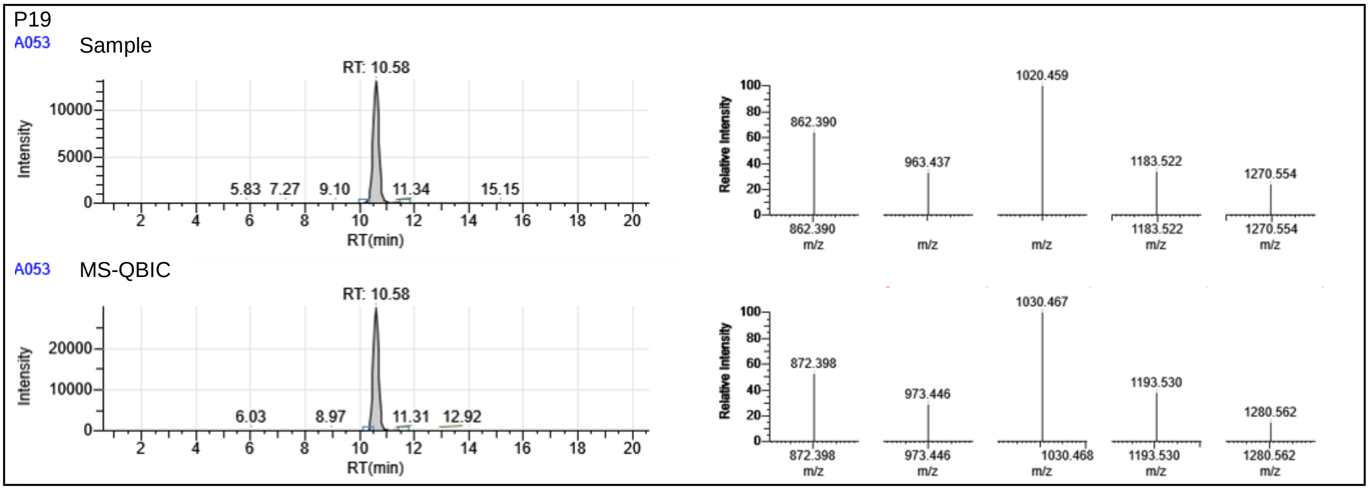
P19
A053 Sample
RT: 10.58
5.83 7.27 9.10 11.34 15.15
Intensity
RT(min)
862.390
963.437
1020.459
1183.522
1270.554
Relative Intensity
m/z
A053 MS-QBIC
RT: 10.58
6.03 8.97 11.31 12.92
872.398
973.446
1030.467
1030.468
1193.530
1280.562

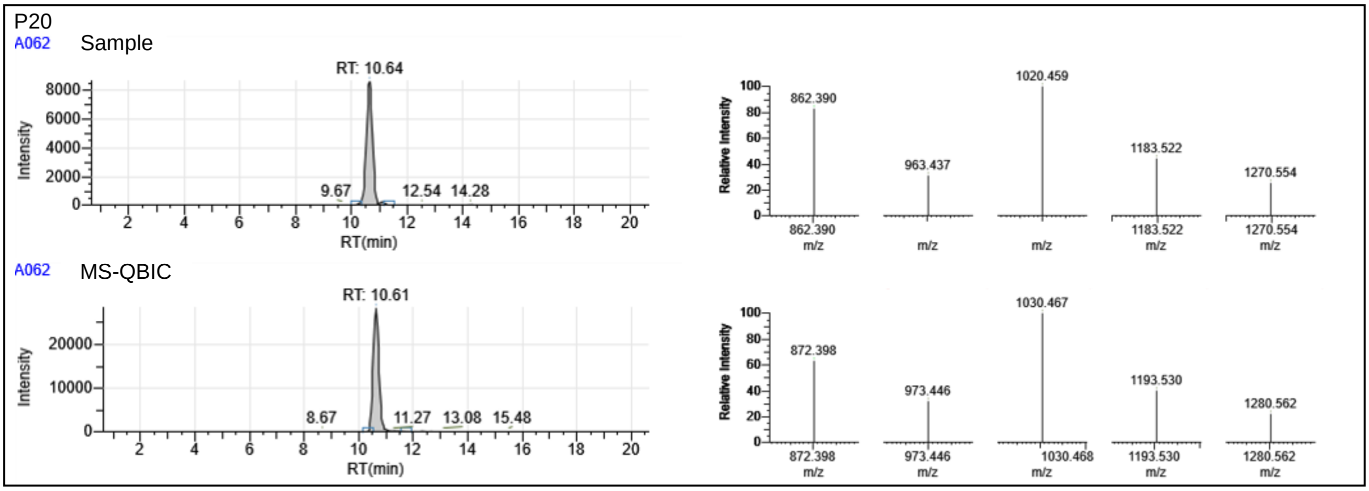
P20
A062 Sample
RT: 10.64
9.67 12.54 14.28
Intensity
RT(min)
862.390
963.437
1020.459
1183.522
1270.554
Relative Intensity
m/z
A062 MS-QBIC
RT: 10.61
8.67 11.27 13.08 15.48
872.398
973.446
1030.467
1030.468
1193.530
1280.562

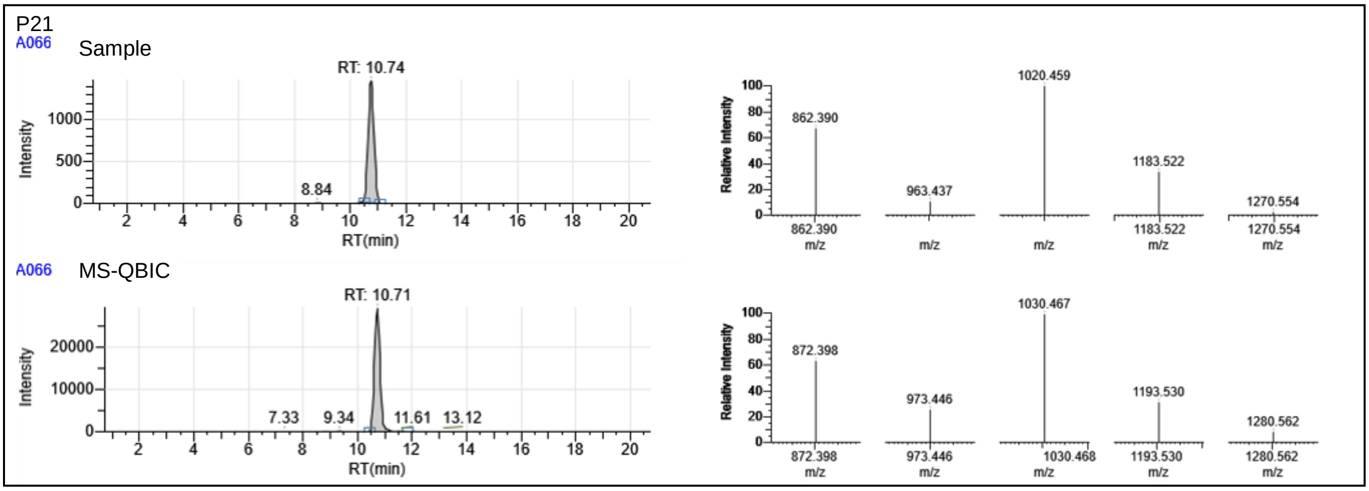
P21
A066 Sample
RT: 10.74
Intensity
RT(min)
8.84
862.390
963.437
1020.459
1183.522
1270.554
Relative Intensity
m/z
A066 MS-QBIC
RT: 10.71
7.33 9.34 11.61 13.12
872.398
973.446
1030.467
1030.468
1193.530
1280.562

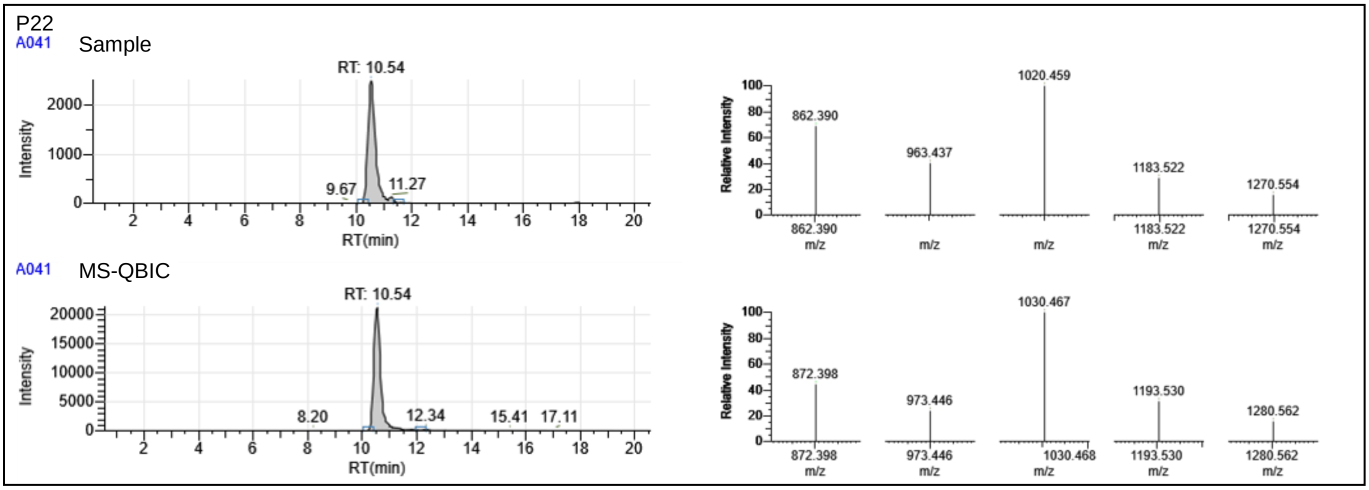
P22
A041 Sample
RT: 10.54
Intensity
RT(min)
9.67 11.27
862.390
963.437
1020.459
1183.522
1270.554
Relative Intensity
m/z
A041 MS-QBIC
RT: 10.54
8.20 12.34 15.41 17.11
872.398
973.446
1030.467
1030.468
1193.530
1280.562

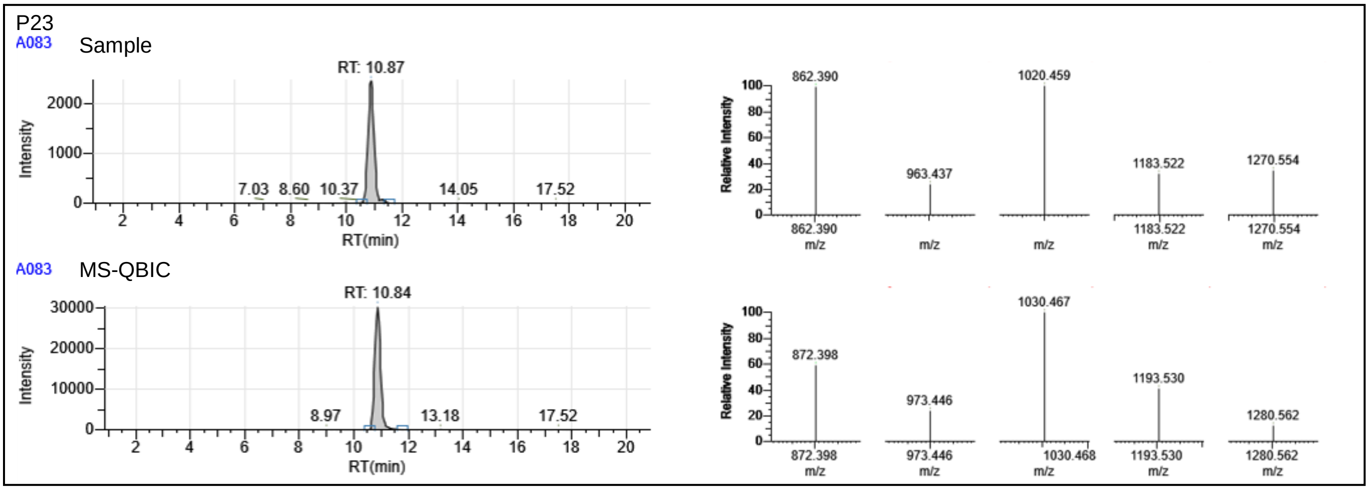
P23
A083 Sample
RT: 10.87
Intensity
RT(min)
7.03 8.60 10.37 14.05 17.52
862.390
963.437
1020.459
1183.522
1270.554
Relative Intensity
m/z
A083 MS-QBIC
RT: 10.84
8.97 13.18 17.52
872.398
973.446
1030.467
1030.468
1193.530
1280.562

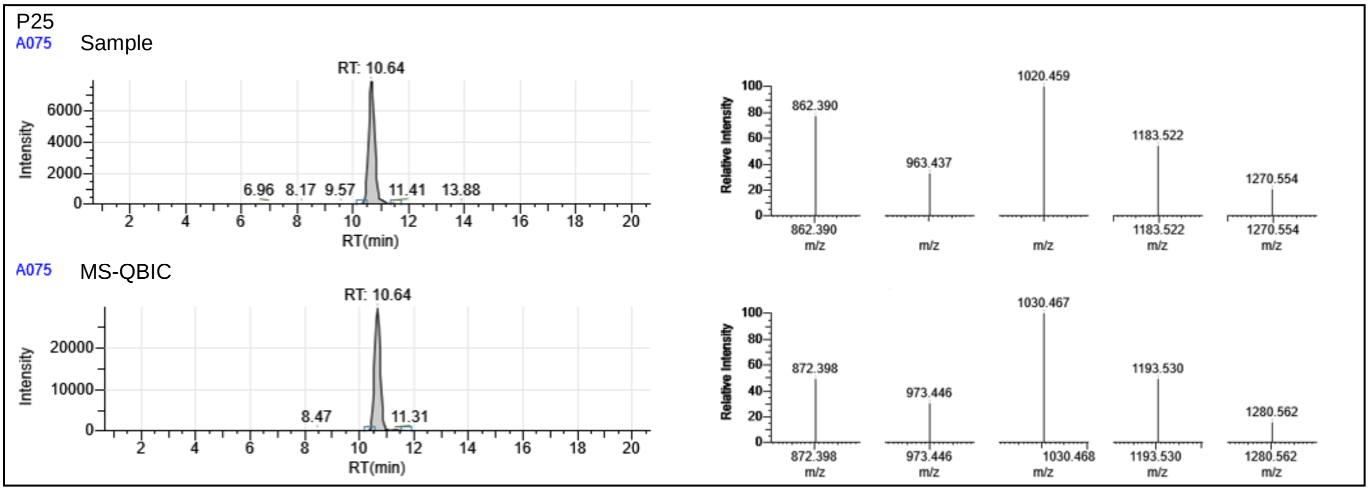
P25
A075 Sample
RT: 10.64
Intensity
RT(min)
6.96 8.17 9.57 11.41 13.88
862.390
963.437
1020.459
1183.522
1270.554
Relative Intensity
m/z
A075 MS-QBIC
RT: 10.64
8.47 11.31
872.398
973.446
1030.467
1030.468
1193.530
1280.562

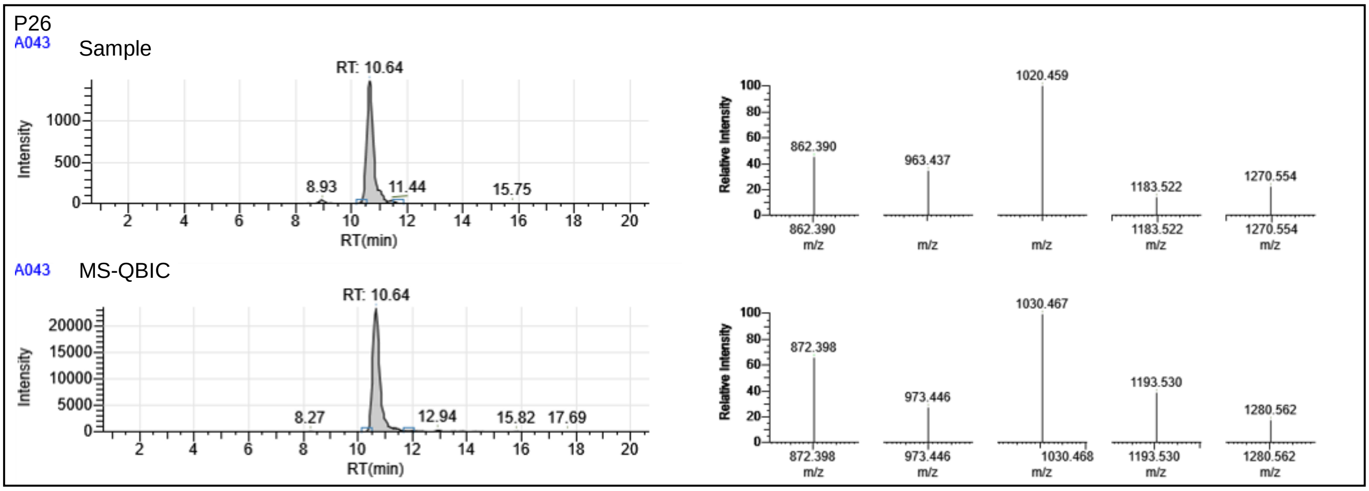
P26
A043 Sample
RT: 10.64
8.93
11.44
15.75
Intensity
RT(min)
862.390
963.437
1020.459
1183.522
1270.554
Relative Intensity
m/z
A043 MS-QBIC
RT: 10.64
8.27
12.94
15.82
17.69
872.398
973.446
1030.467
1030.468
1193.530
1280.562

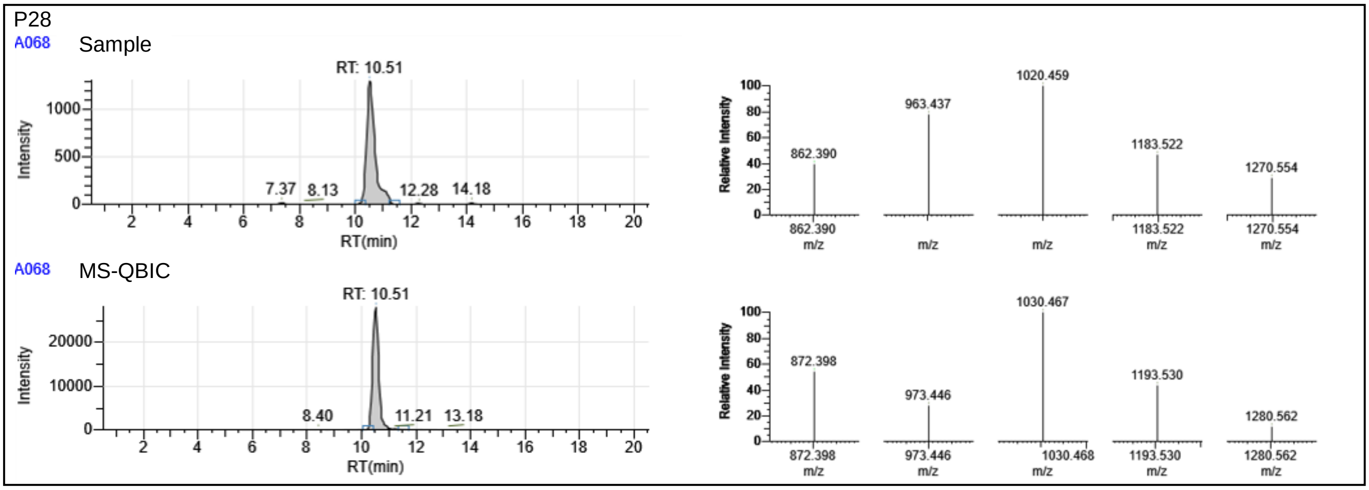
P28
A068 Sample
RT: 10.51
7.37
8.13
12.28
14.18
Intensity
RT(min)
862.390
963.437
1020.459
1183.522
1270.554
Relative Intensity
m/z
A068 MS-QBIC
RT: 10.51
8.40
11.21
13.18
872.398
973.446
1030.467
1030.468
1193.530
1280.562

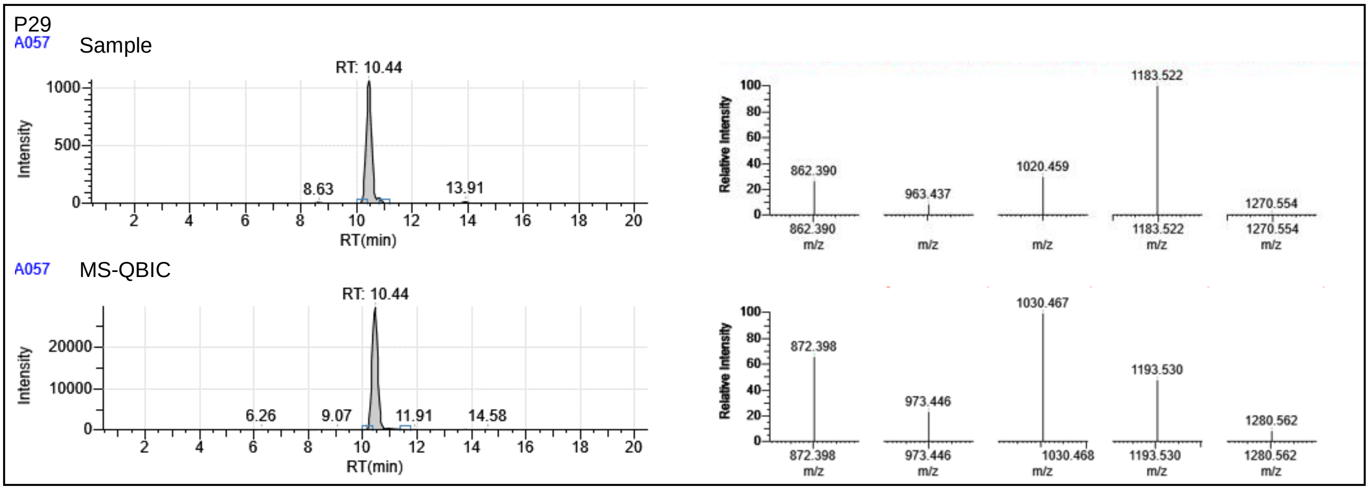
P29
A057 Sample
RT: 10.44
8.63
13.91
Intensity
RT(min)
862.390
963.437
1020.459
1183.522
1270.554
Relative Intensity
m/z
A057 MS-QBIC
RT: 10.44
6.26
9.07
11.91
14.58
872.398
973.446
1030.467
1030.468
1193.530
1280.562

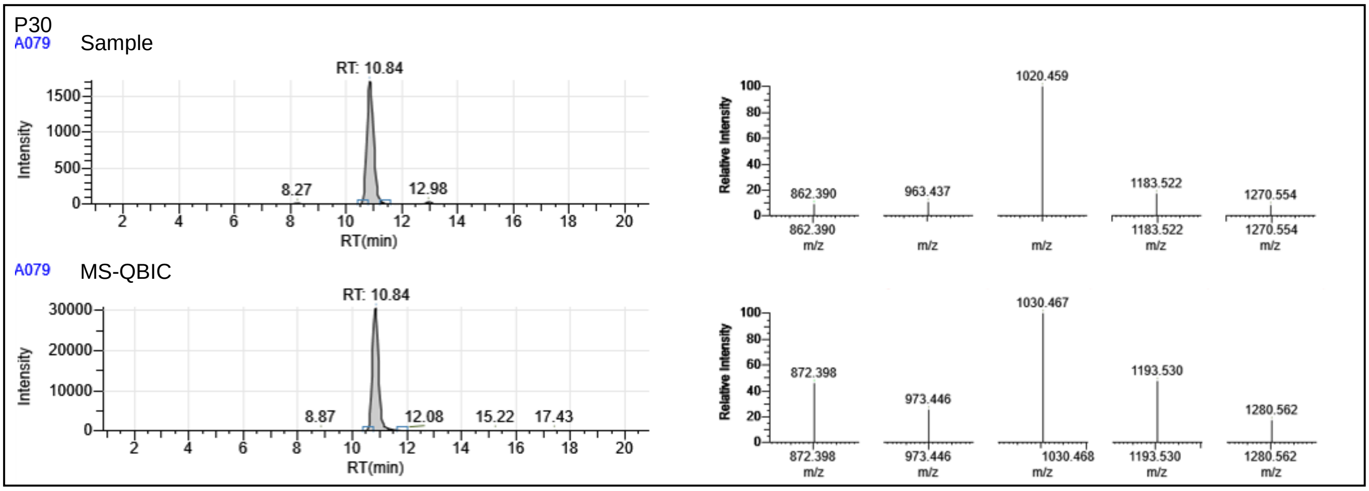
P30
A079 Sample
RT: 10.84
8.27
12.98
Intensity
RT(min)
862.390
963.437
1020.459
1183.522
1270.554
Relative Intensity
m/z
A079 MS-QBIC
RT: 10.84
8.87
12.08
15.22
17.43
872.398
973.446
1030.467
1030.468
1193.530
1280.562

L Lysozyme (STDYGIFQINSR) Heart

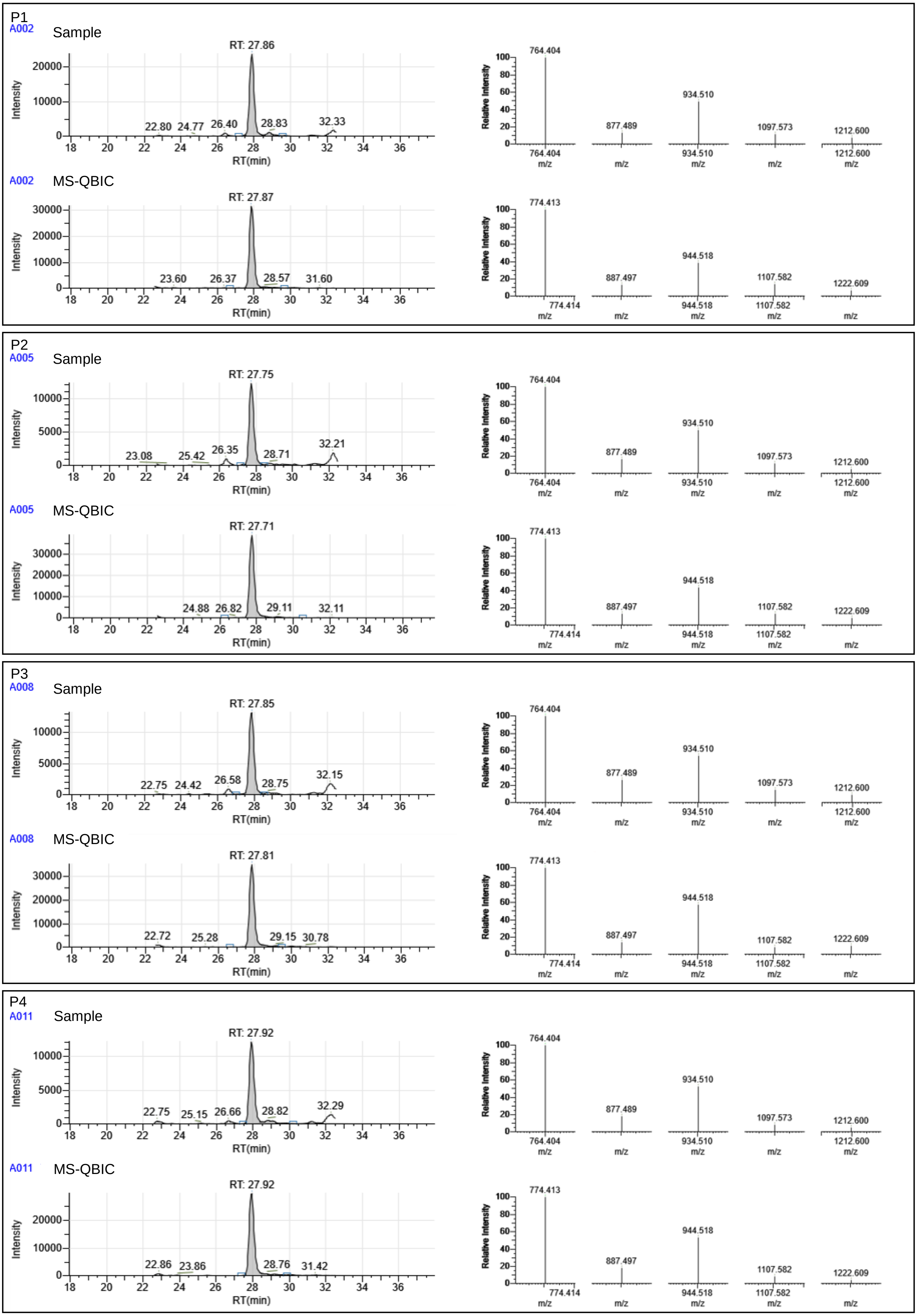

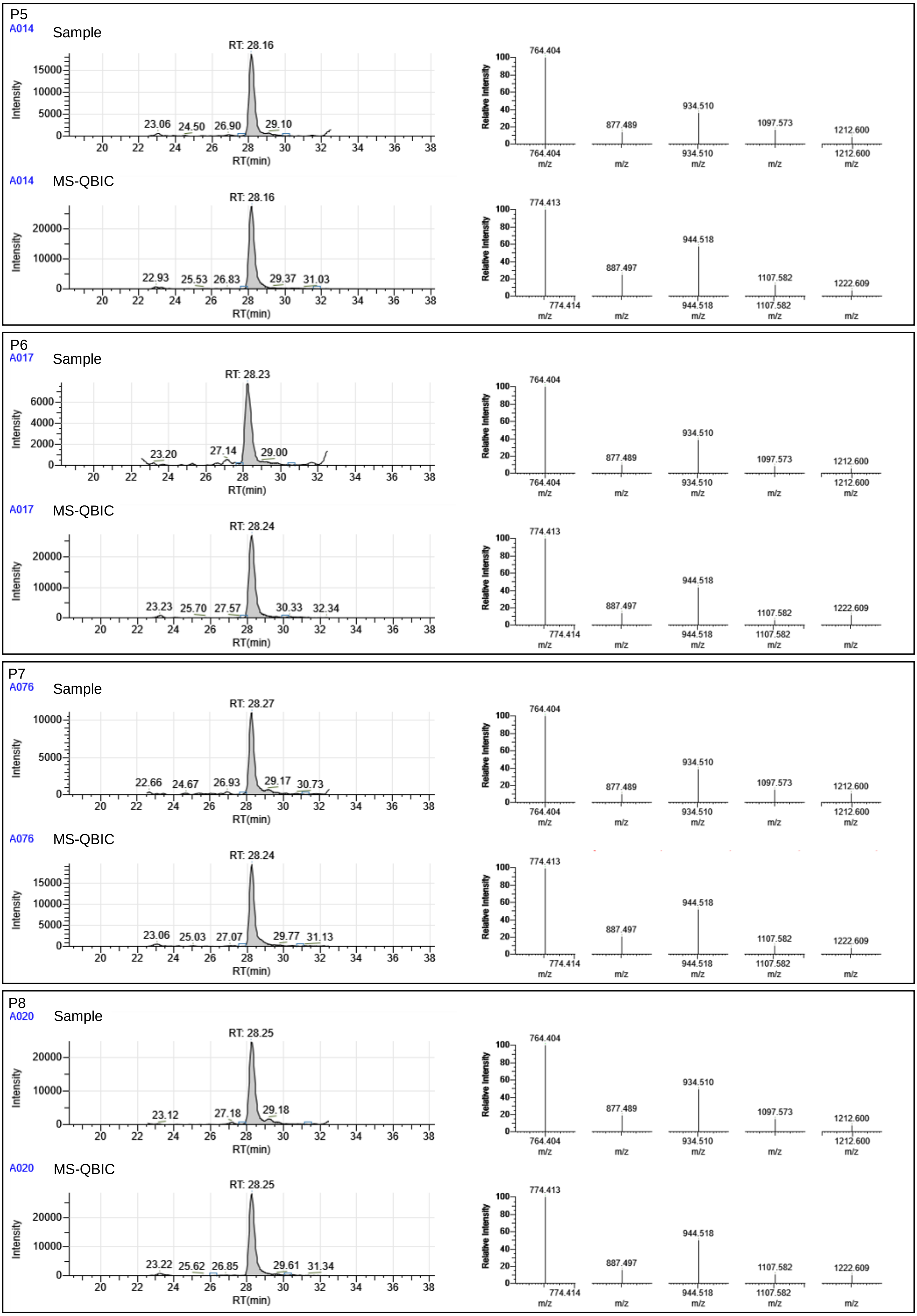

P5
A014 Sample
RT: 28.16
23.06 24.50 26.90 29.10
764.404
877.489
934.510
1097.573
1212.600
A014 MS-QBIC
RT: 28.16
22.93 25.53 26.83 29.37 31.03
774.413
887.497
944.518
1107.582
1222.609
774.414
P6
A017 Sample
RT: 28.23
23.20 27.14 29.00
764.404
877.489
934.510
1097.573
1212.600
A017 MS-QBIC
RT: 28.24
23.23 25.70 27.57 30.33 32.34
774.413
887.497
944.518
1107.582
1222.609
774.414
P7
A076 Sample
RT: 28.27
22.66 24.67 26.93 29.17 30.73
764.404
877.489
934.510
1097.573
1212.600
A076 MS-QBIC
RT: 28.24
23.06 25.03 27.07 29.77 31.13
774.413
887.497
944.518
1107.582
1222.609
774.414
P8
A020 Sample
RT: 28.25
23.12 27.18 29.18
764.404
877.489
934.510
1097.573
1212.600
A020 MS-QBIC
RT: 28.25
23.22 25.62 26.85 29.61 31.34
774.413
887.497
944.518
1107.582
1222.609
774.414
Intensity
RT(min)
Relative Intensity
m/z

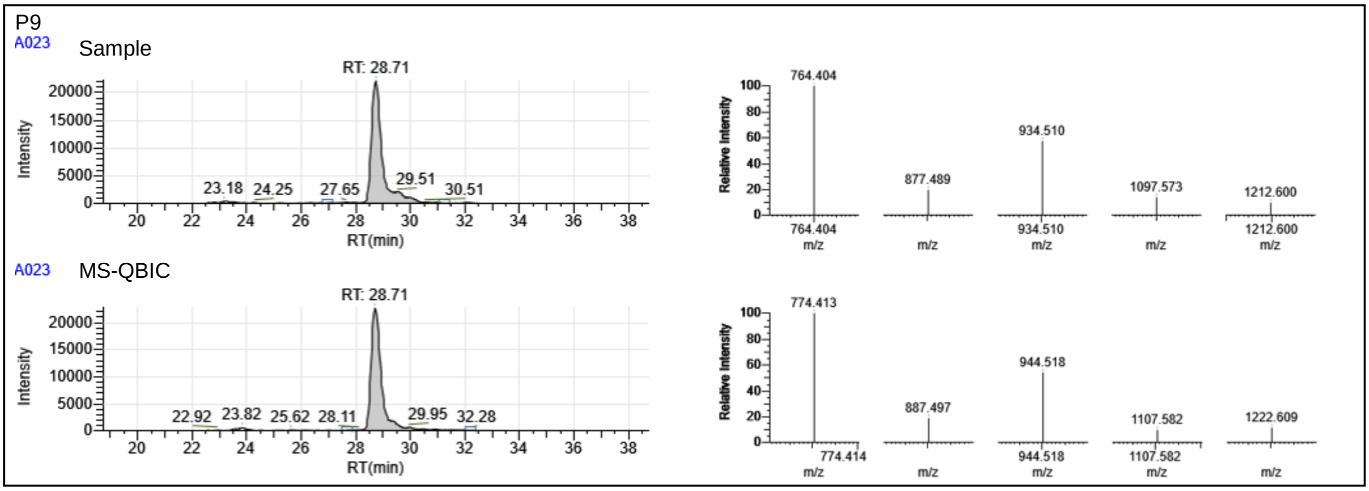
P9
A023 Sample
RT: 28.71
23.18 24.25 27.65 29.51 30.51
Intensity
RT(min)
764.404
934.510
877.489
1097.573
1212.600
Relative Intensity
m/z
A023 MS-QBIC
RT: 28.71
22.92 23.82 25.62 28.11 29.95 32.28
774.413
944.518
887.497
1107.582
1222.609
774.414

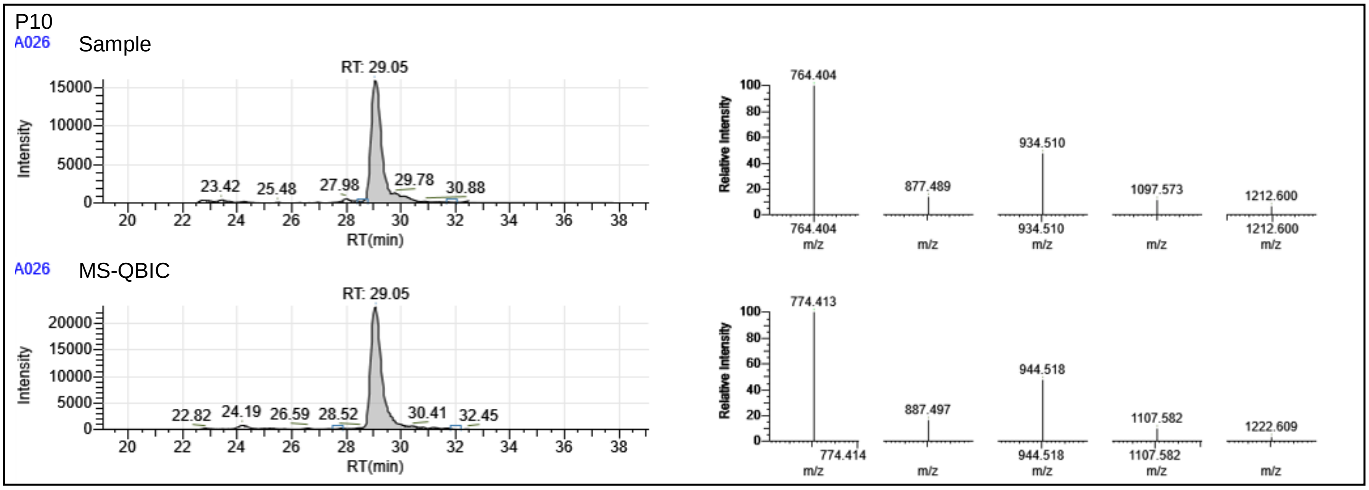
P10
A026 Sample
RT: 29.05
23.42 25.48 27.98 29.78 30.88
Intensity
RT(min)
764.404
934.510
877.489
1097.573
1212.600
Relative Intensity
m/z
A026 MS-QBIC
RT: 29.05
22.82 24.19 26.59 28.52 30.41 32.45
774.413
944.518
887.497
1107.582
1222.609
774.414

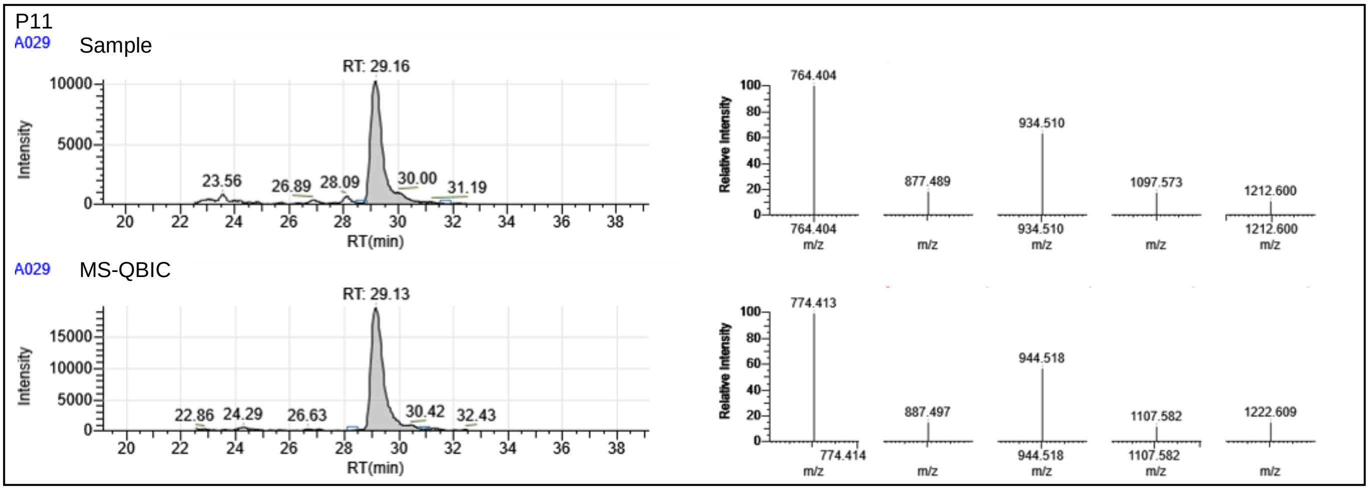
P11
A029 Sample
RT: 29.16
23.56 26.89 28.09 30.00 31.19
Intensity
RT(min)
764.404
934.510
877.489
1097.573
1212.600
Relative Intensity
m/z
A029 MS-QBIC
RT: 29.13
22.86 24.29 26.63 30.42 32.43
774.413
944.518
887.497
1107.582
1222.609
774.414

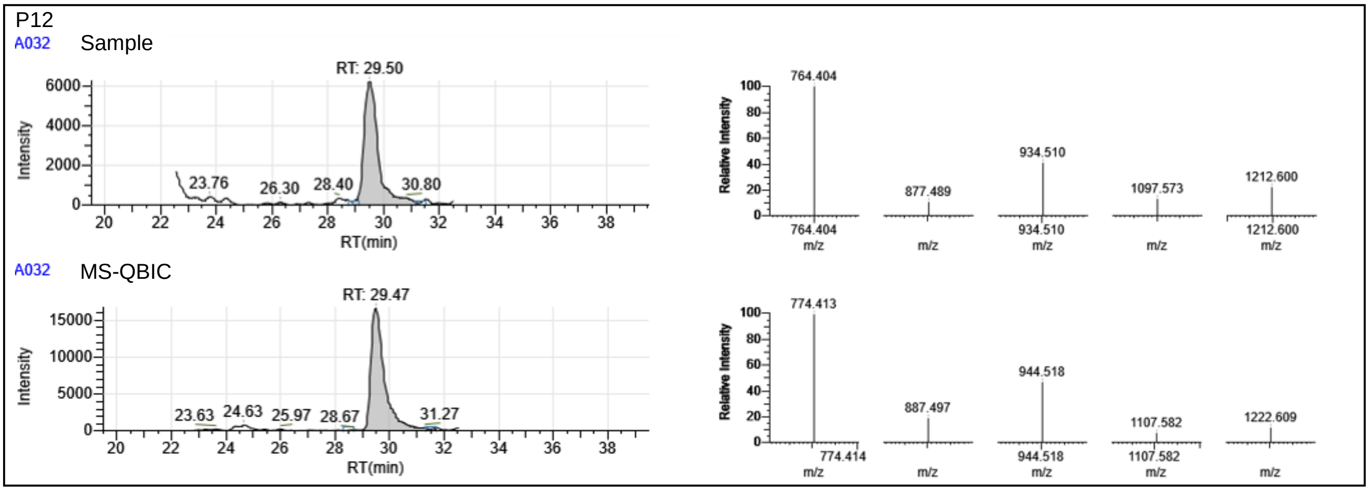
P12
A032 Sample
RT: 29.50
23.76 26.30 28.40 30.80
Intensity
RT(min)
764.404
934.510
877.489
1097.573
1212.600
Relative Intensity
m/z
A032 MS-QBIC
RT: 29.47
23.63 24.63 25.97 28.67 31.27
774.413
944.518
887.497
1107.582
1222.609
774.414

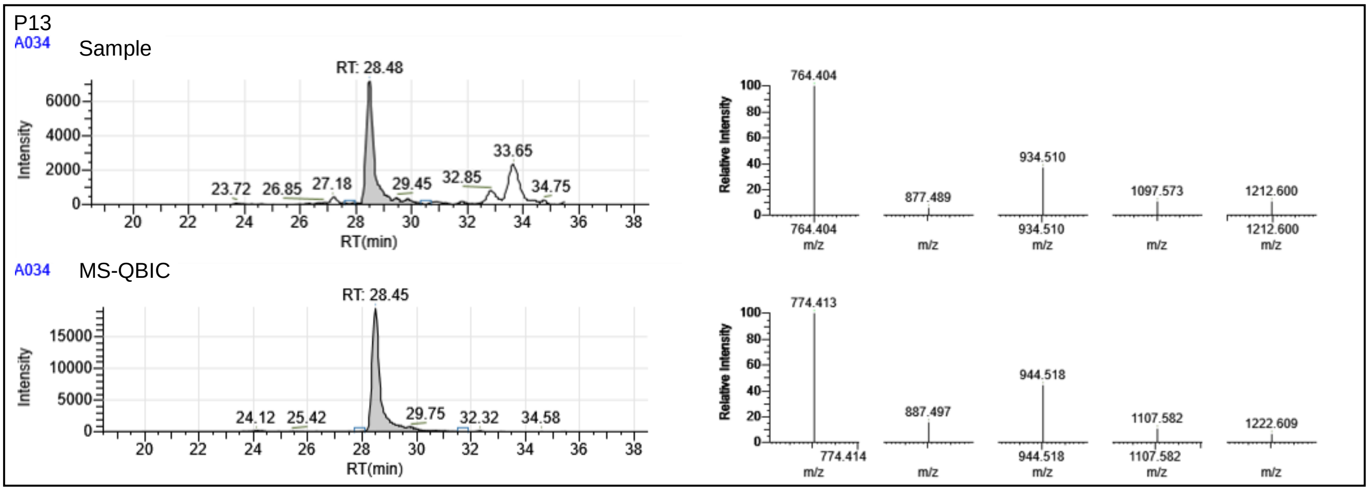
P13
A034 Sample
RT: 28.48
23.72 26.85 27.18 29.45 32.85 33.65 34.75
Intensity
RT(min)
764.404
934.510
877.489
1097.573
1212.600
Relative Intensity
m/z
A034 MS-QBIC
RT: 28.45
24.12 25.42 29.75 32.32 34.58
774.413
774.414
944.518
887.497
1107.582
1222.609

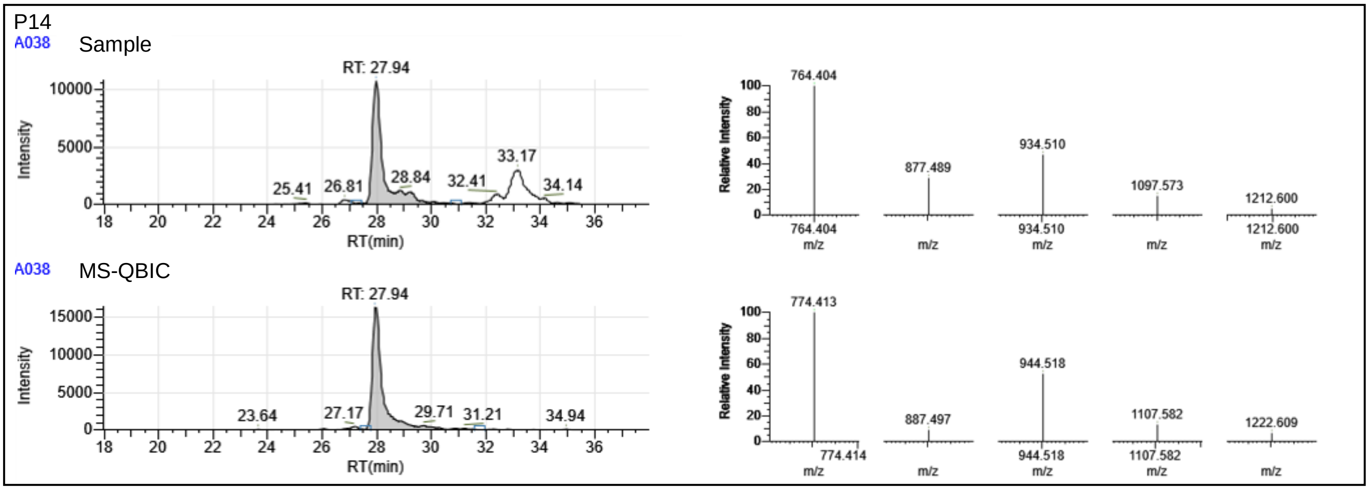
P14
A038 Sample
RT: 27.94
25.41 26.81 28.84 32.41 33.17 34.14
Intensity
RT(min)
764.404
934.510
877.489
1097.573
1212.600
Relative Intensity
m/z
A038 MS-QBIC
RT: 27.94
23.64 27.17 29.71 31.21 34.94
774.413
774.414
944.518
887.497
1107.582
1222.609

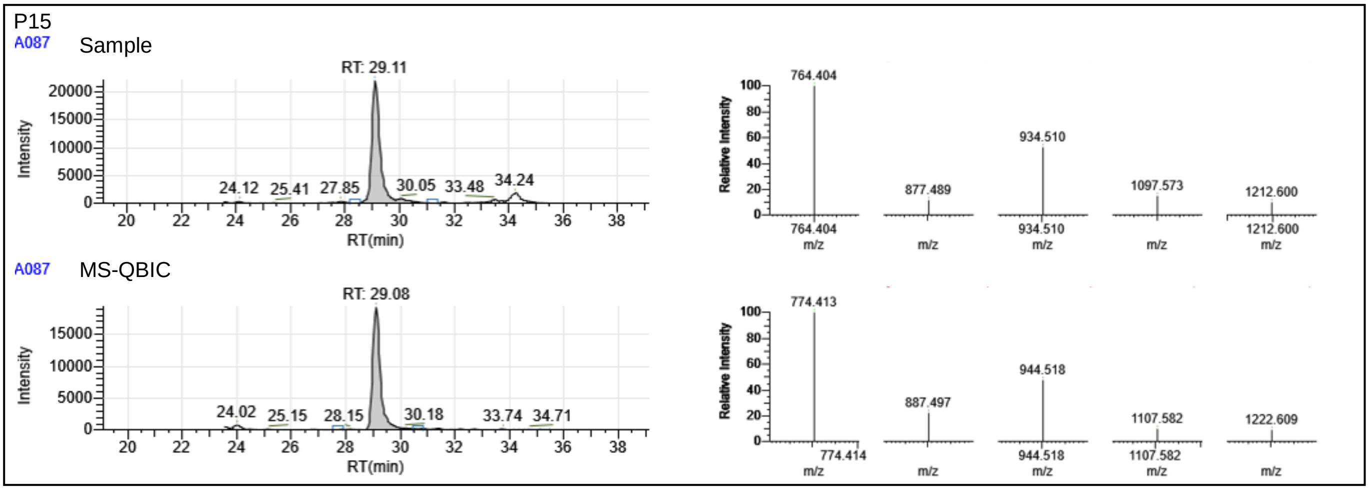
P15
A087 Sample
RT: 29.11
24.12 25.41 27.85 30.05 33.48 34.24
Intensity
RT(min)
764.404
934.510
877.489
1097.573
1212.600
Relative Intensity
m/z
A087 MS-QBIC
RT: 29.08
24.02 25.15 28.15 30.18 33.74 34.71
774.413
774.414
944.518
887.497
1107.582
1222.609

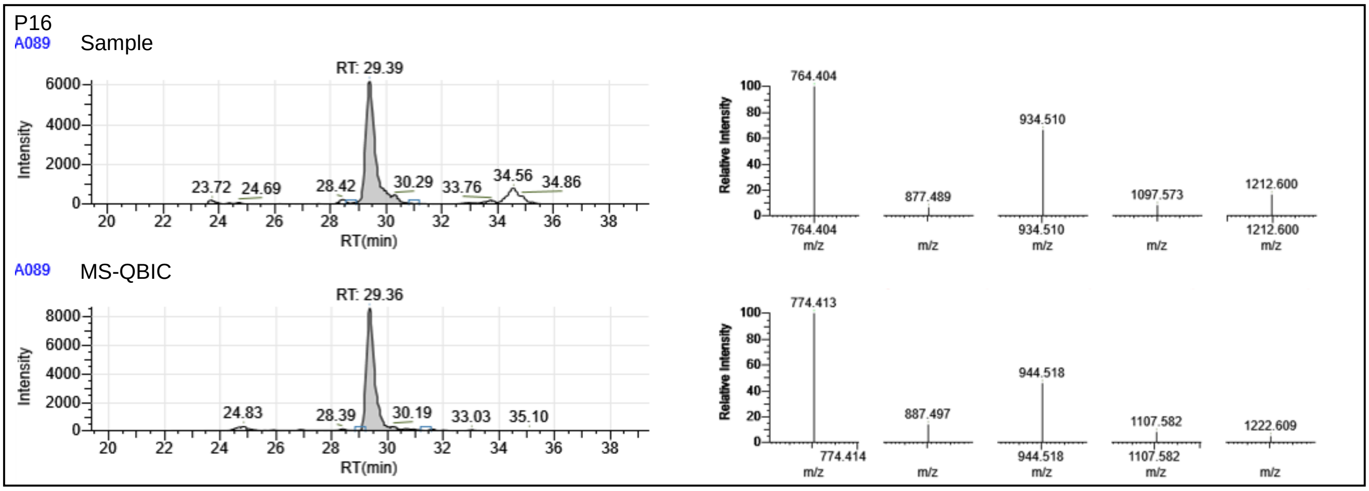
P16
A089 Sample
RT: 29.39
23.72 24.69 28.42 30.29 33.76 34.56 34.86
Intensity
RT(min)
764.404
934.510
877.489
1097.573
1212.600
Relative Intensity
m/z
A089 MS-QBIC
RT: 29.36
24.83 28.39 30.19 33.03 35.10
774.413
774.414
944.518
887.497
1107.582
1222.609

P17

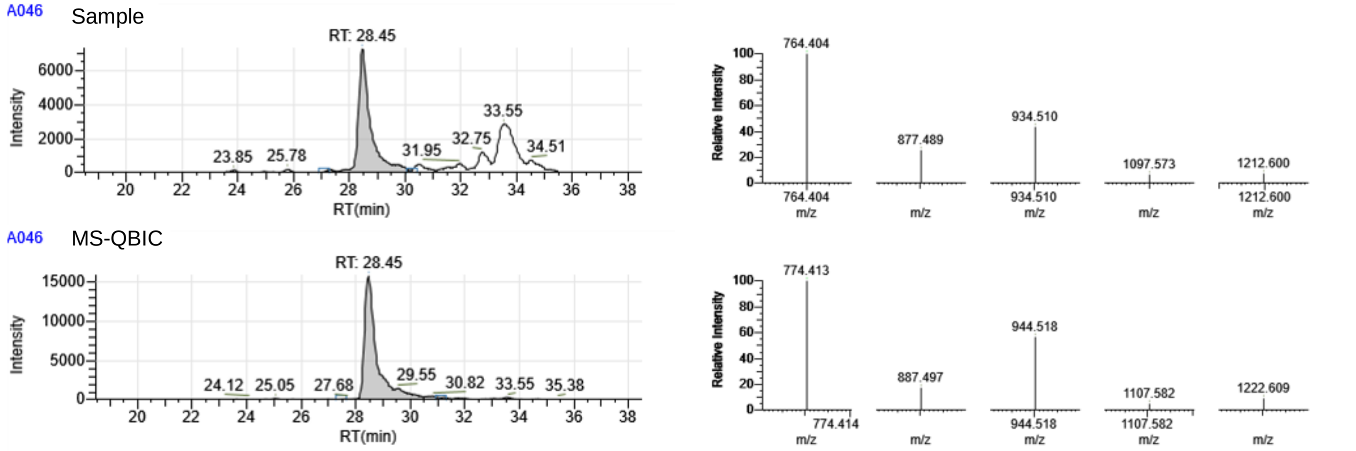

P18

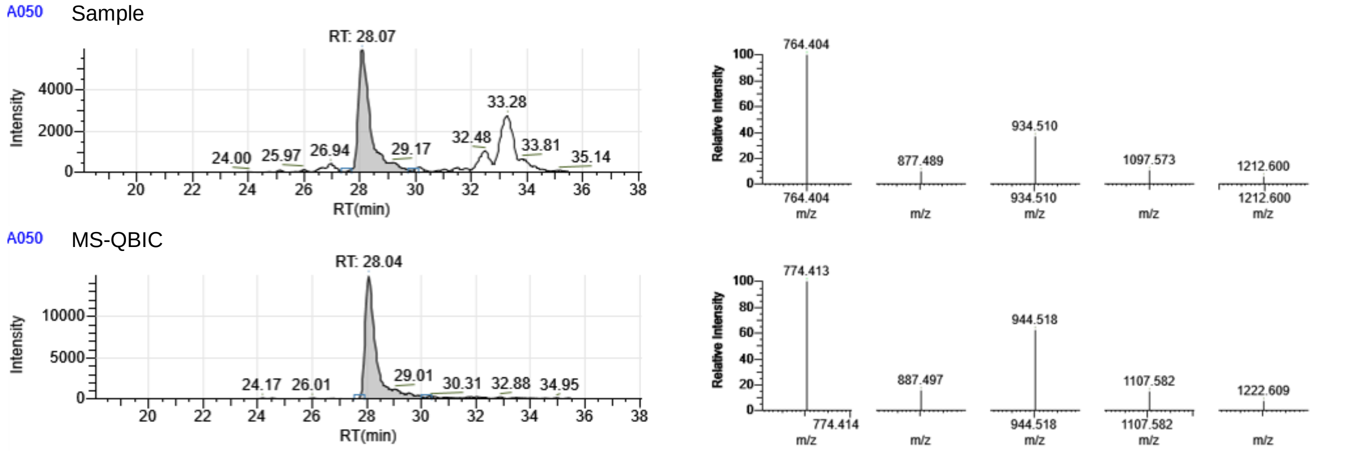

P19

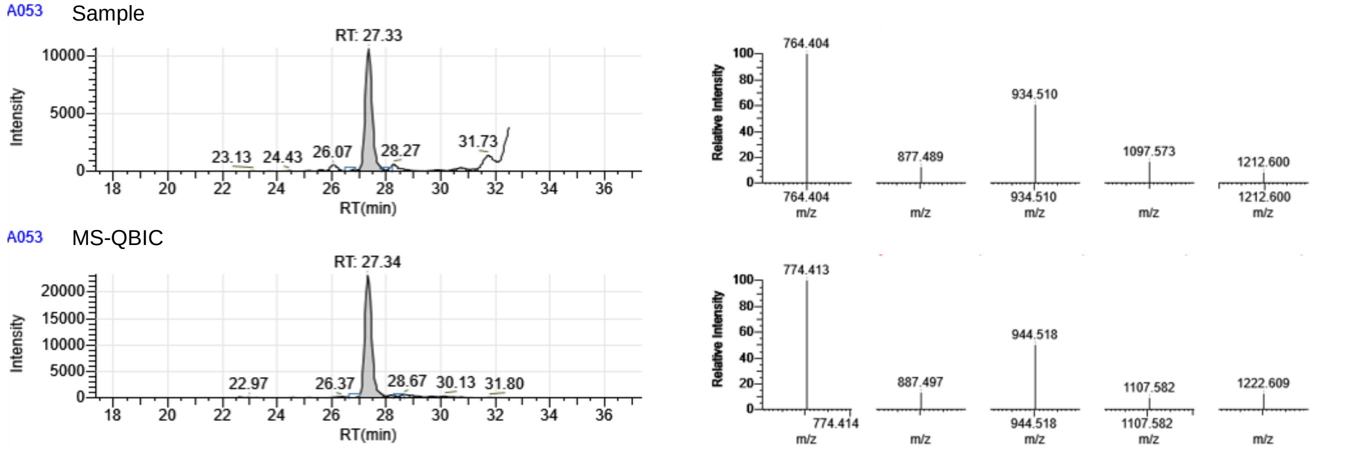

P20

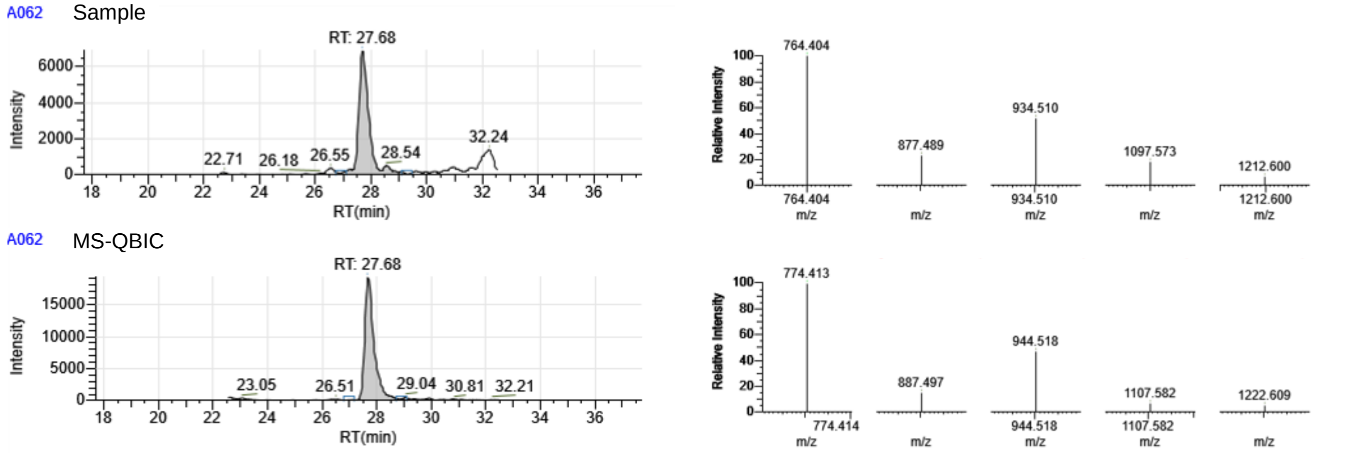

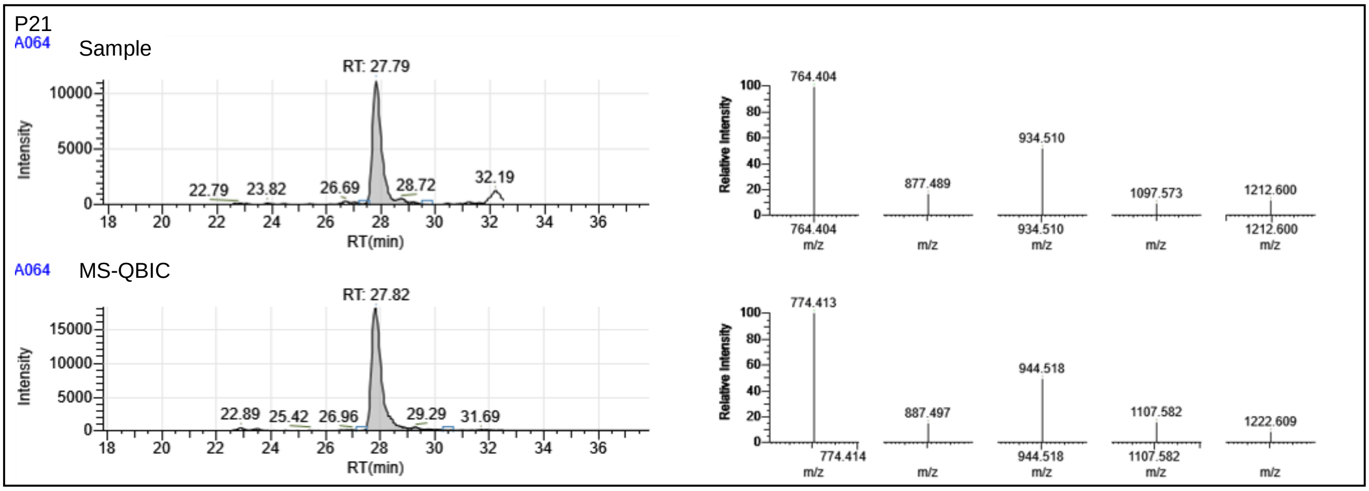
P21
A064 Sample
RT: 27.79
22.79 23.82 26.69 28.72 32.19
Intensity
RT(min)
764.404
934.510
877.489
1097.573
1212.600
Relative Intensity
m/z
A064 MS-QBIC
RT: 27.82
22.89 25.42 26.96 29.29 31.69
774.413
944.518
887.497
1107.582
1222.609
774.414

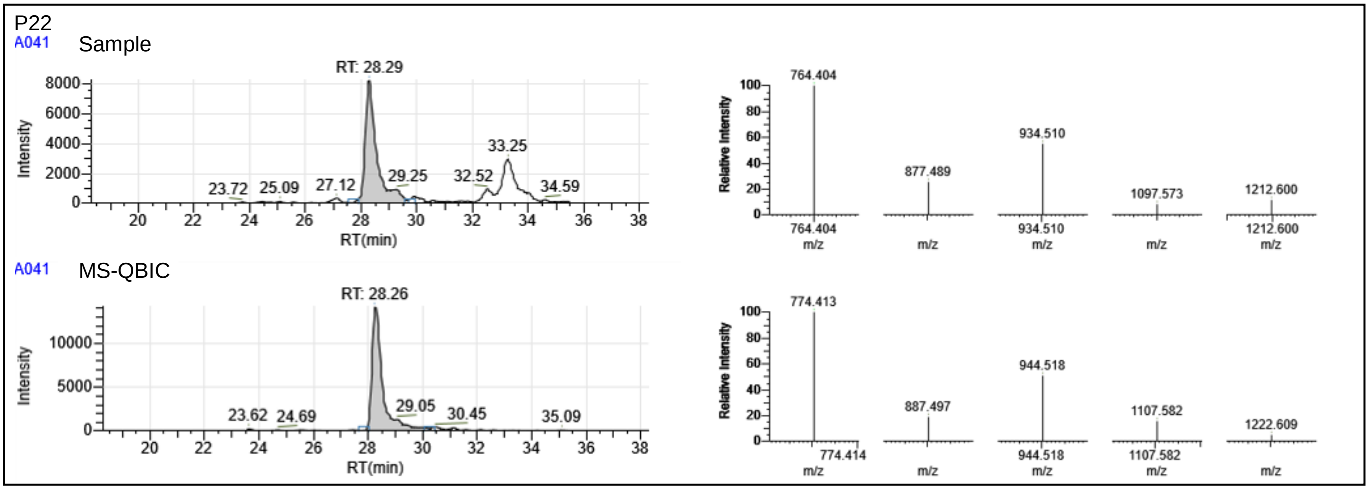
P22
A041 Sample
RT: 28.29
23.72 25.09 27.12 29.25 32.52 33.25 34.59
Intensity
RT(min)
764.404
934.510
877.489
1097.573
1212.600
Relative Intensity
m/z
A041 MS-QBIC
RT: 28.26
23.62 24.69 29.05 30.45 35.09
774.413
944.518
887.497
1107.582
1222.609
774.414

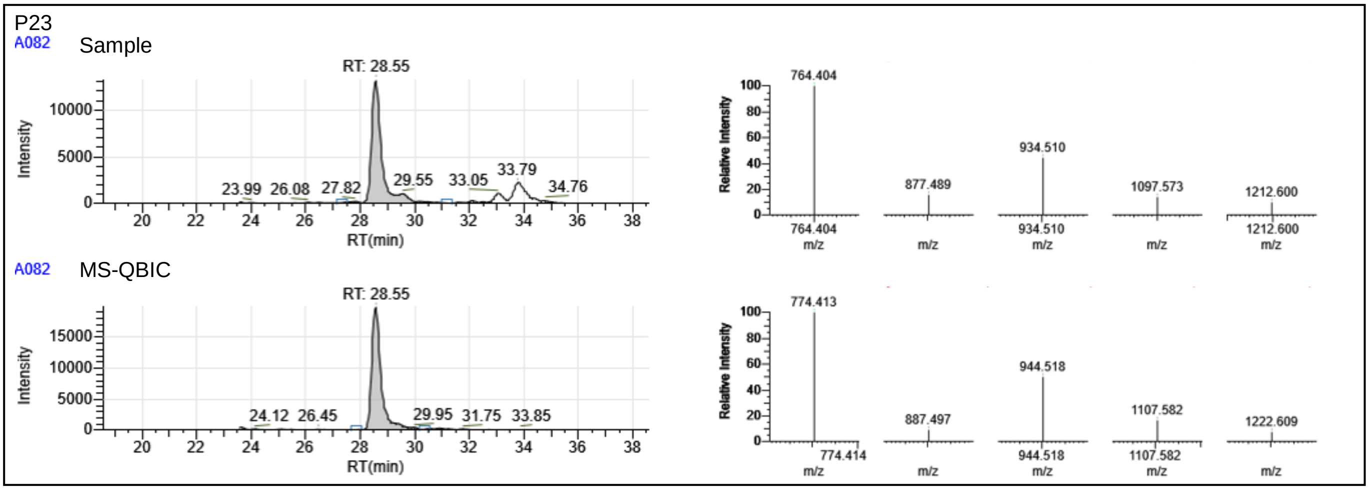
P23
A082 Sample
RT: 28.55
23.99 26.08 27.82 29.55 33.05 33.79 34.76
Intensity
RT(min)
764.404
934.510
877.489
1097.573
1212.600
Relative Intensity
m/z
A082 MS-QBIC
RT: 28.55
24.12 26.45 29.95 31.75 33.85
774.413
944.518
887.497
1107.582
1222.609
774.414

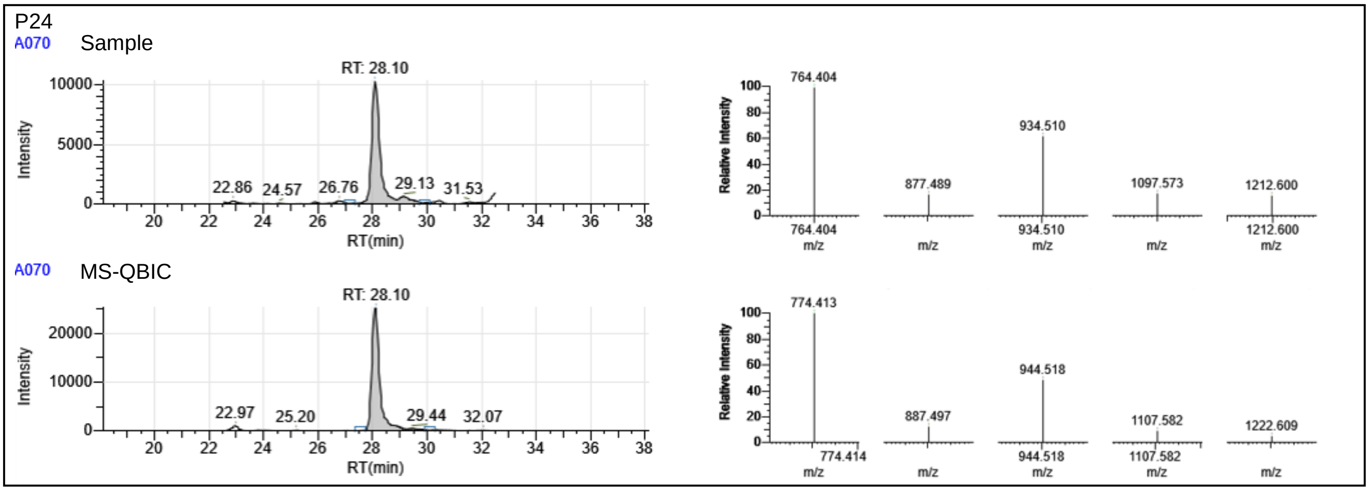
P24
A070 Sample
RT: 28.10
22.86 24.57 26.76 29.13 31.53
Intensity
RT(min)
764.404
934.510
877.489
1097.573
1212.600
Relative Intensity
m/z
A070 MS-QBIC
RT: 28.10
22.97 25.20 29.44 32.07
774.413
944.518
887.497
1107.582
1222.609
774.414

P25

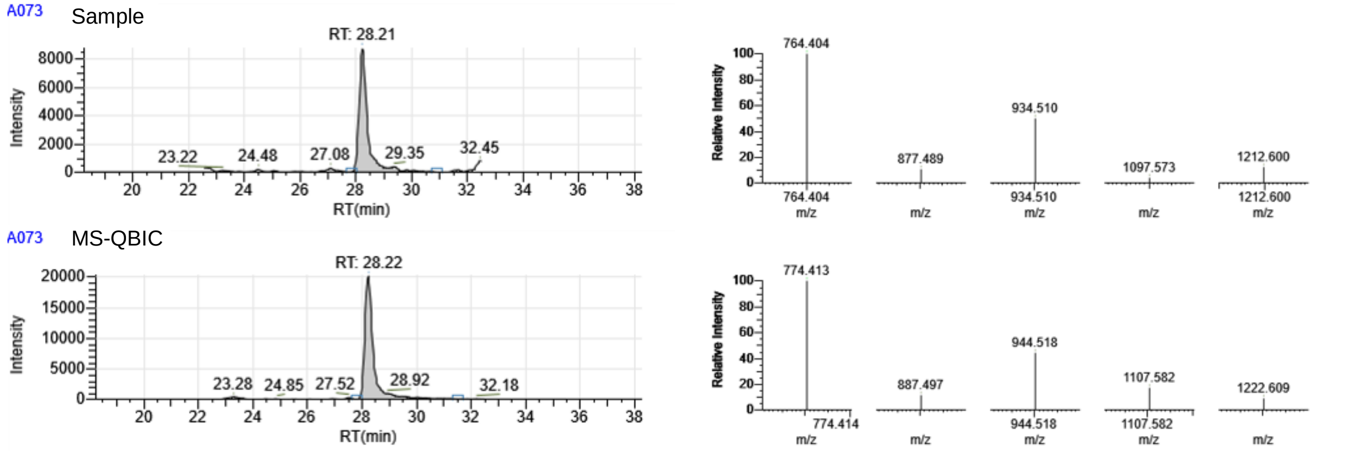

P26

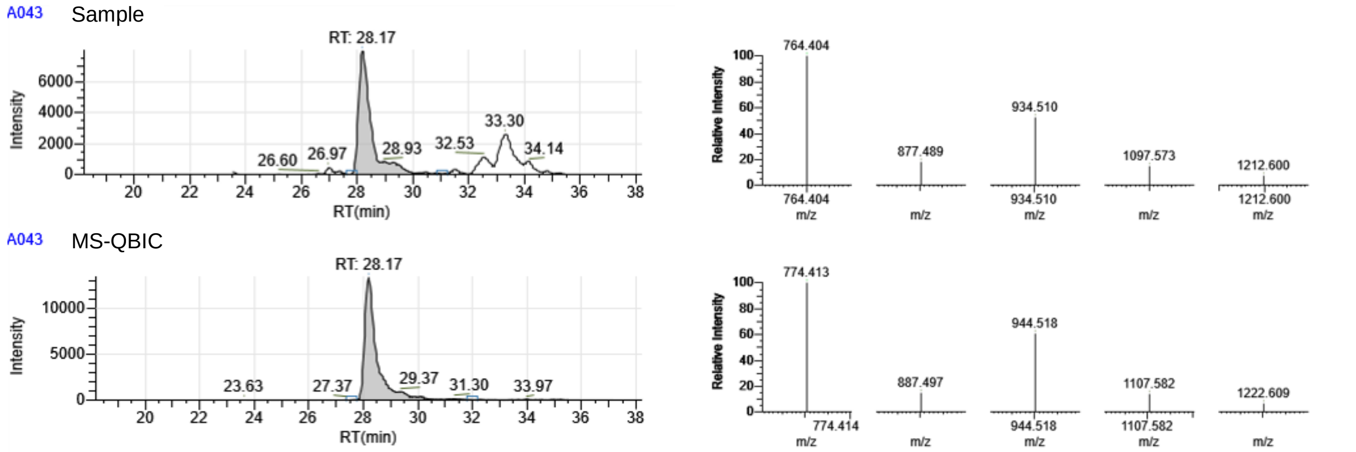

P27

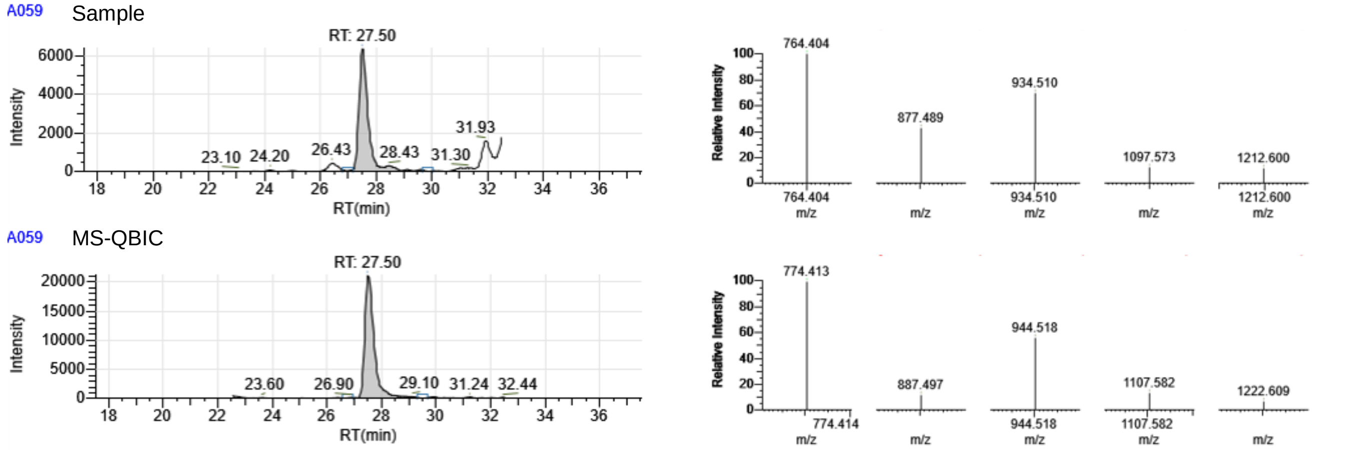

P28

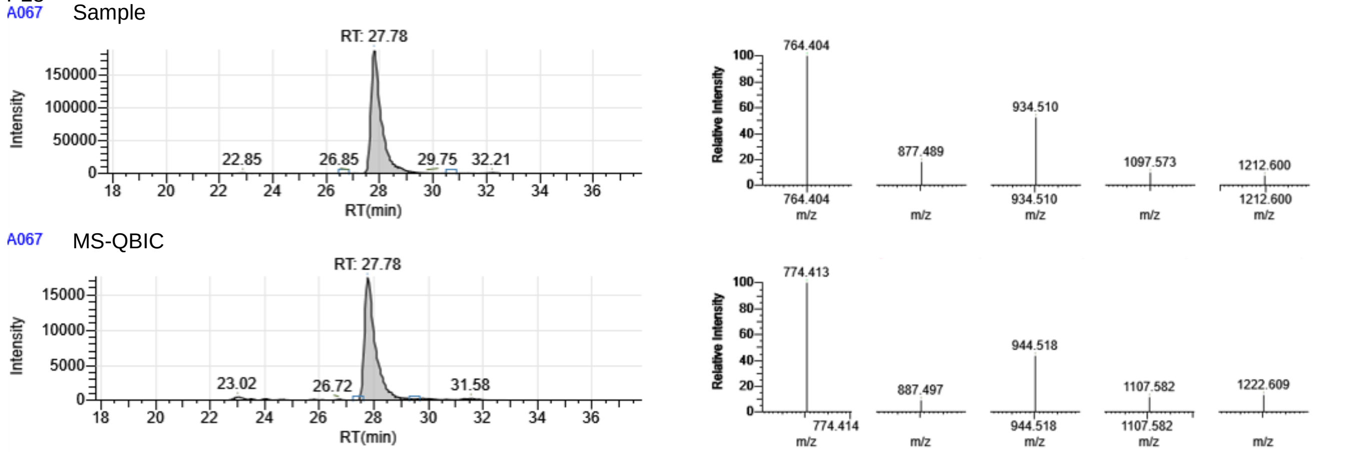

P29

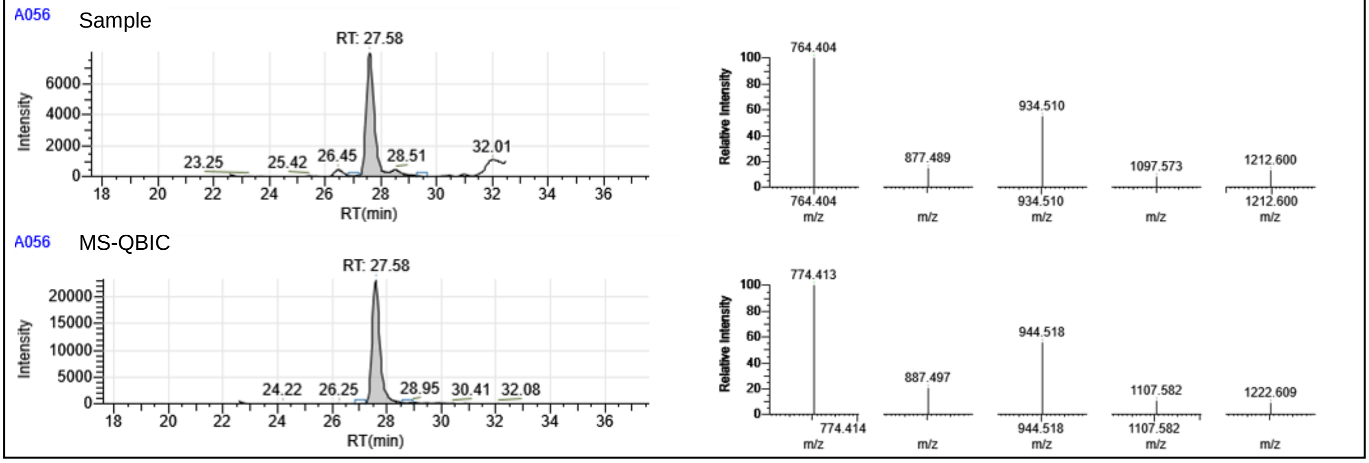
A056 Sample
RT: 27.58
23.25
25.42
26.45
28.51
32.01
Intensity
RT(min)
764.404
877.489
934.510
1097.573
1212.600
Relative Intensity
m/z
A056 MS-QBIC
RT: 27.58
24.22
26.25
28.95
30.41
32.08
774.413
774.414
887.497
944.518
1107.582
1222.609

P30

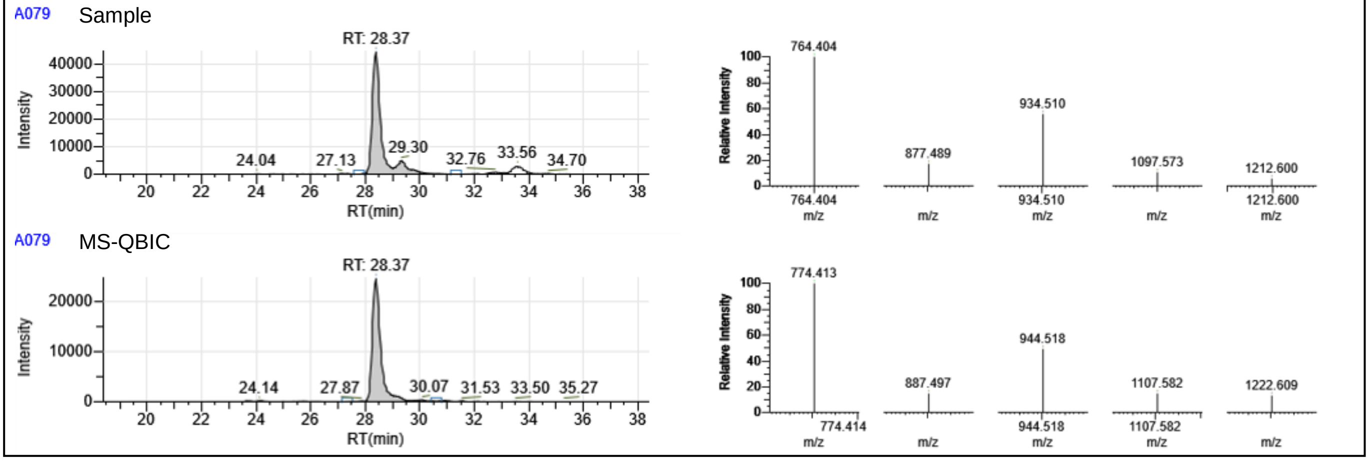
A079 Sample
RT: 28.37
24.04
27.13
29.30
32.76
33.56
34.70
Intensity
RT(min)
764.404
877.489
934.510
1097.573
1212.600
Relative Intensity
m/z
A079 MS-QBIC
RT: 28.37
24.14
27.87
30.07
31.53
33.50
35.27
774.413
774.414
887.497
944.518
1107.582
1222.609

M SAA (EANYIGSDK) Another organ

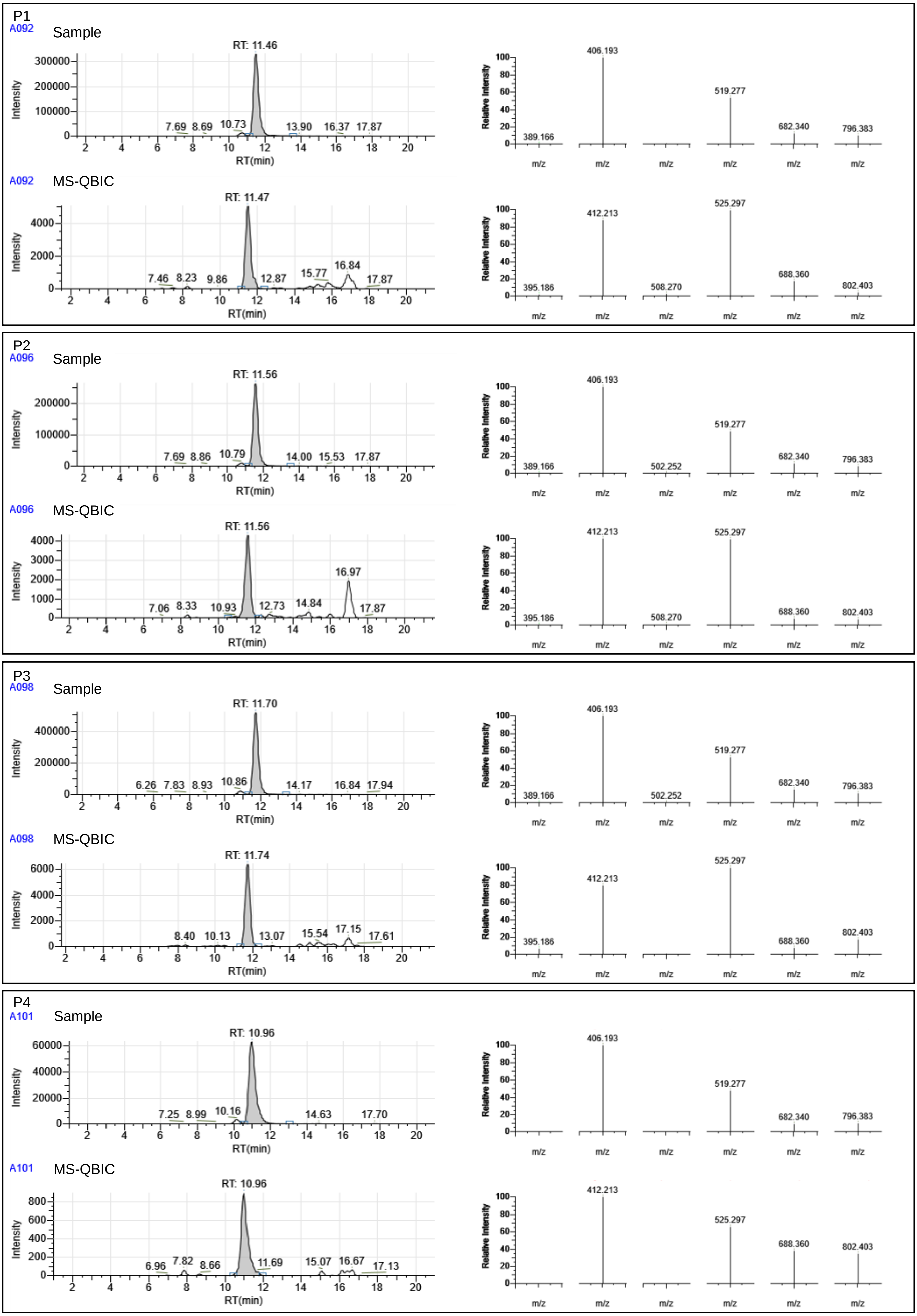

P5

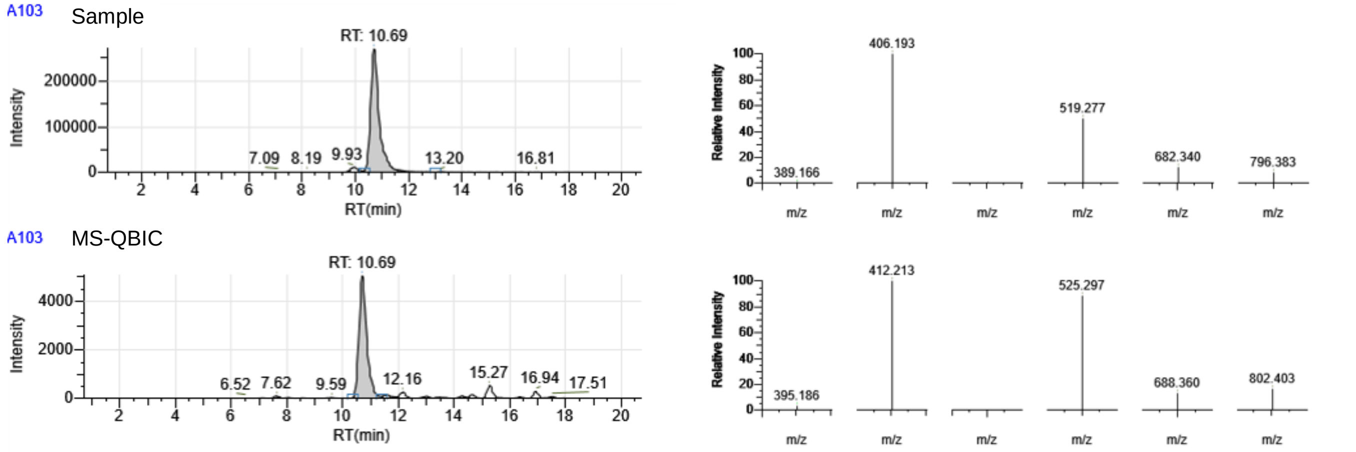
A103 Sample
RT: 10.69
7.09 8.19 9.93 13.20 16.81
Intensity
RT(min)
Relative Intensity
389.166
406.193
519.277
682.340
796.383
m/z
A103 MS-QBIC
RT: 10.69
6.52 7.62 9.59 12.16 15.27 16.94 17.51
395.186
412.213
525.297
688.360
802.403

P6

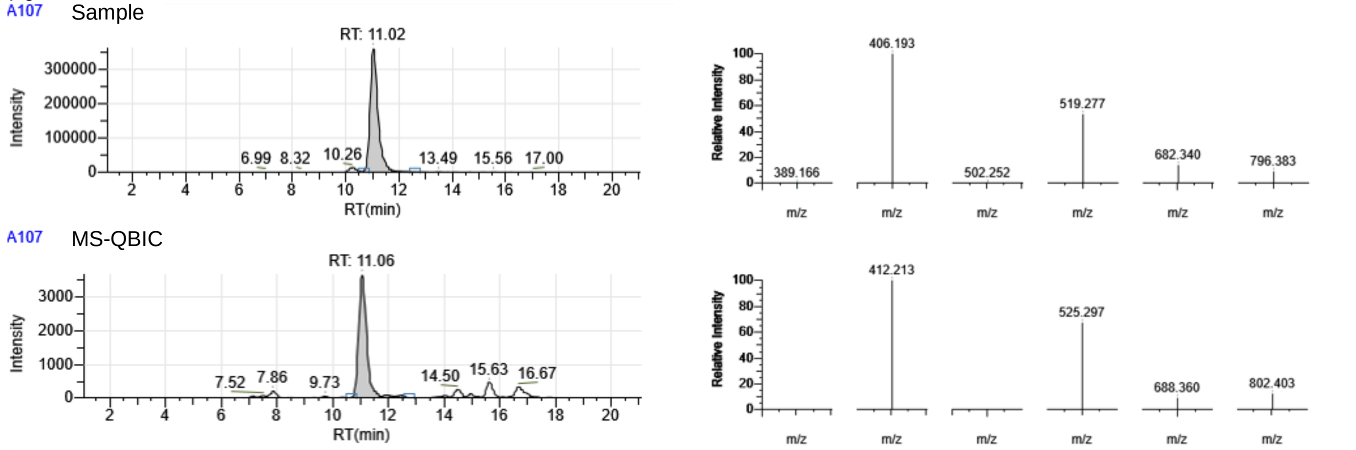
A107 Sample
RT: 11.02
6.99 8.32 10.26 13.49 15.56 17.00
Intensity
RT(min)
Relative Intensity
389.166
406.193
502.252
519.277
682.340
796.383
m/z
A107 MS-QBIC
RT: 11.06
7.52 7.86 9.73 14.50 15.63 16.67
412.213
525.297
688.360
802.403

P7

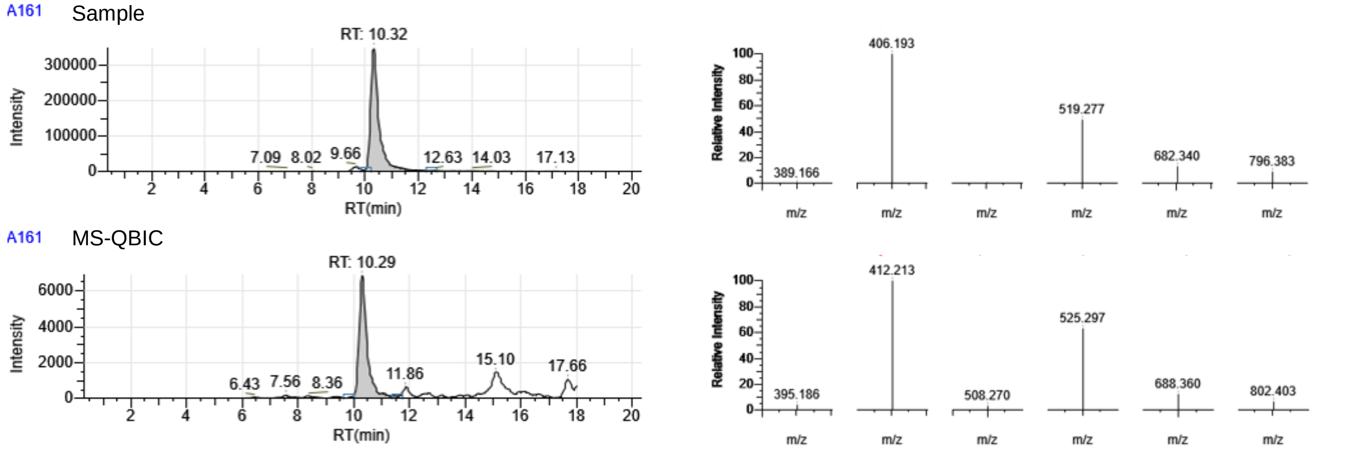
A161 Sample
RT: 10.32
7.09 8.02 9.66 12.63 14.03 17.13
Intensity
RT(min)
Relative Intensity
389.166
406.193
519.277
682.340
796.383
m/z
A161 MS-QBIC
RT: 10.29
6.43 7.56 8.36 11.86 15.10 17.66
395.186
412.213
508.270
525.297
688.360
802.403

P8

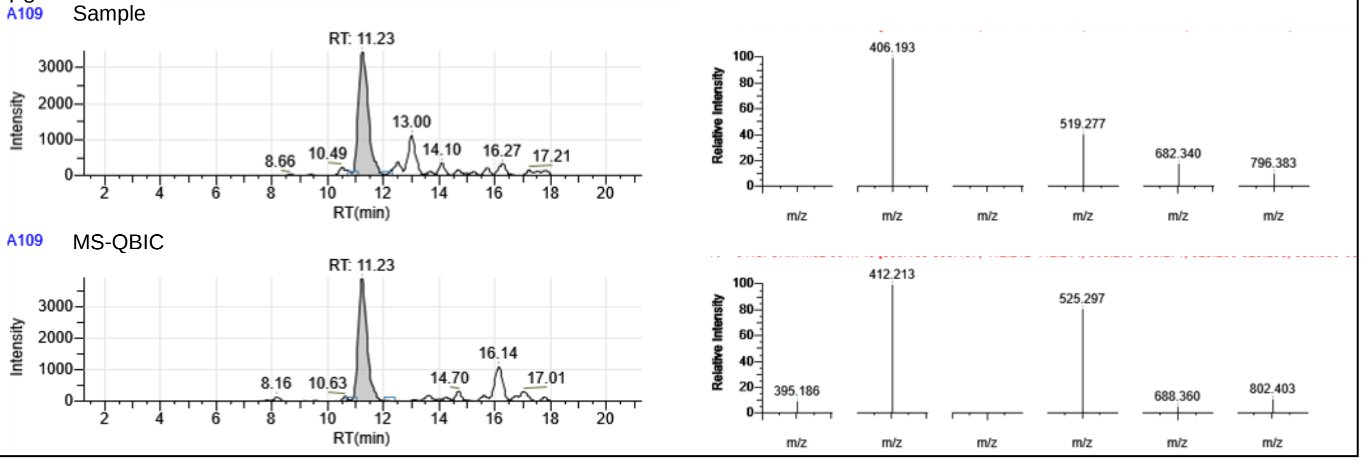
A109 Sample
RT: 11.23
8.66 10.49 13.00 14.10 16.27 17.21
Intensity
RT(min)
Relative Intensity
406.193
519.277
682.340
796.383
m/z
A109 MS-QBIC
RT: 11.23
8.16 10.63 14.70 16.14 17.01
395.186
412.213
525.297
688.360
802.403

P18

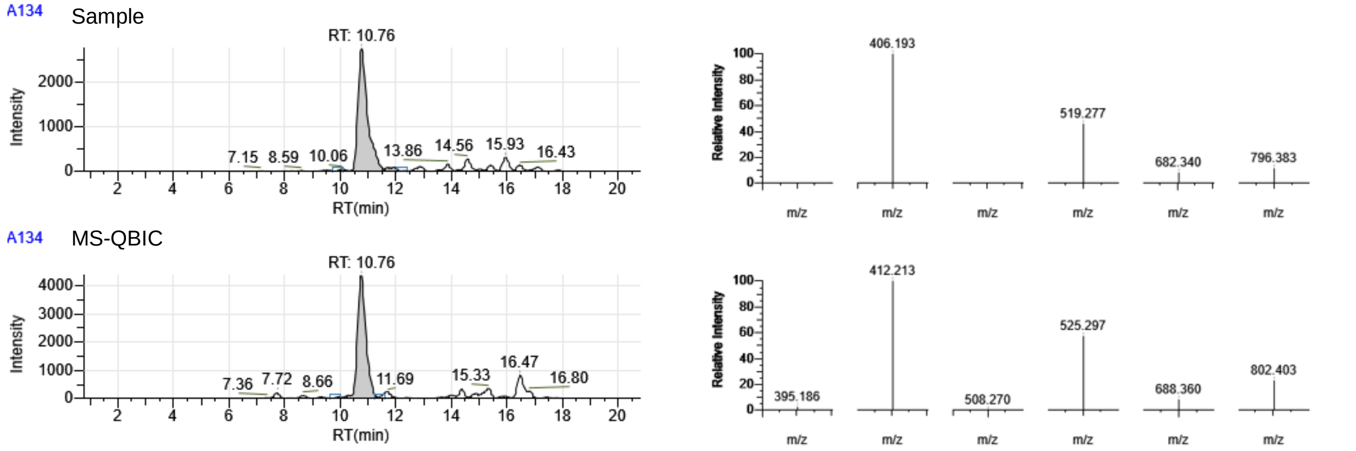

P21

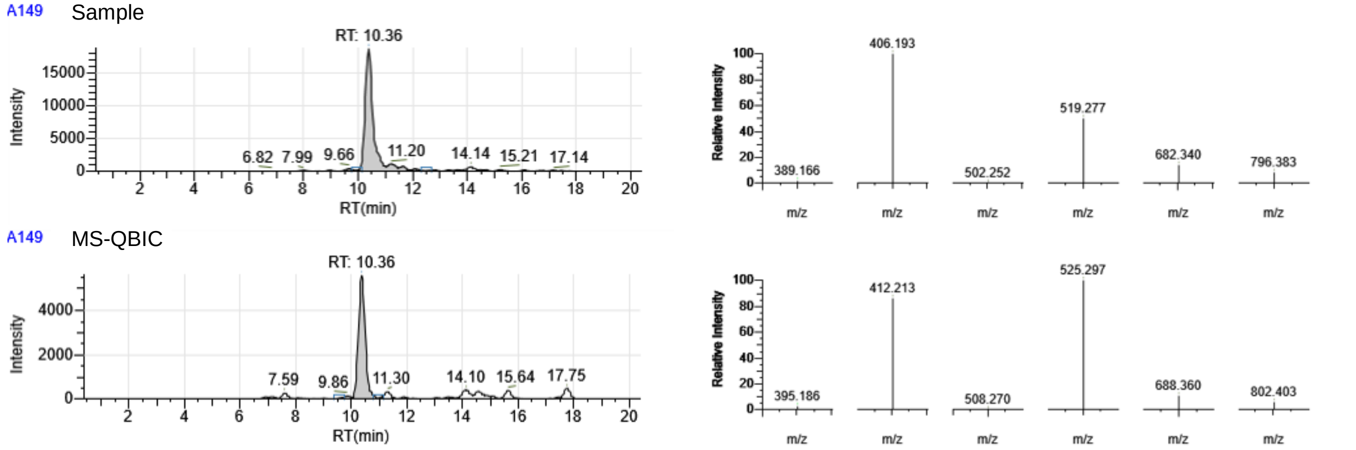

P22

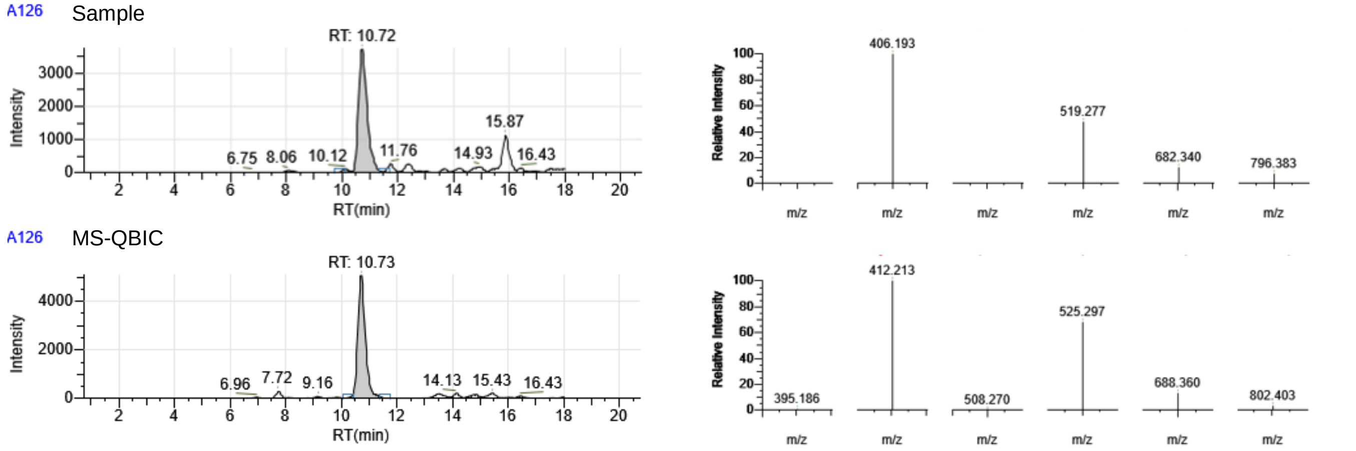

P23

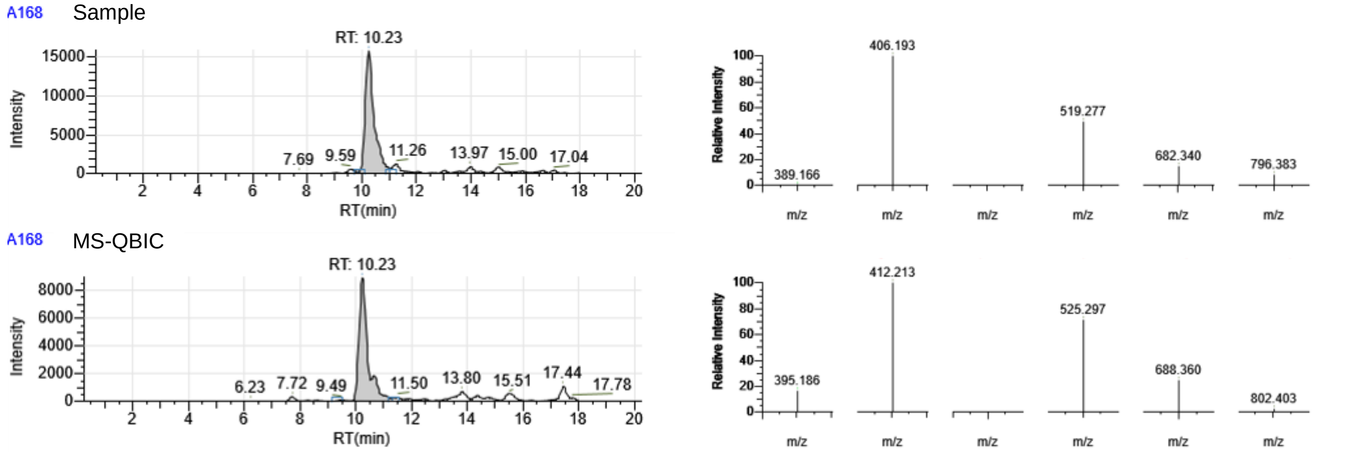

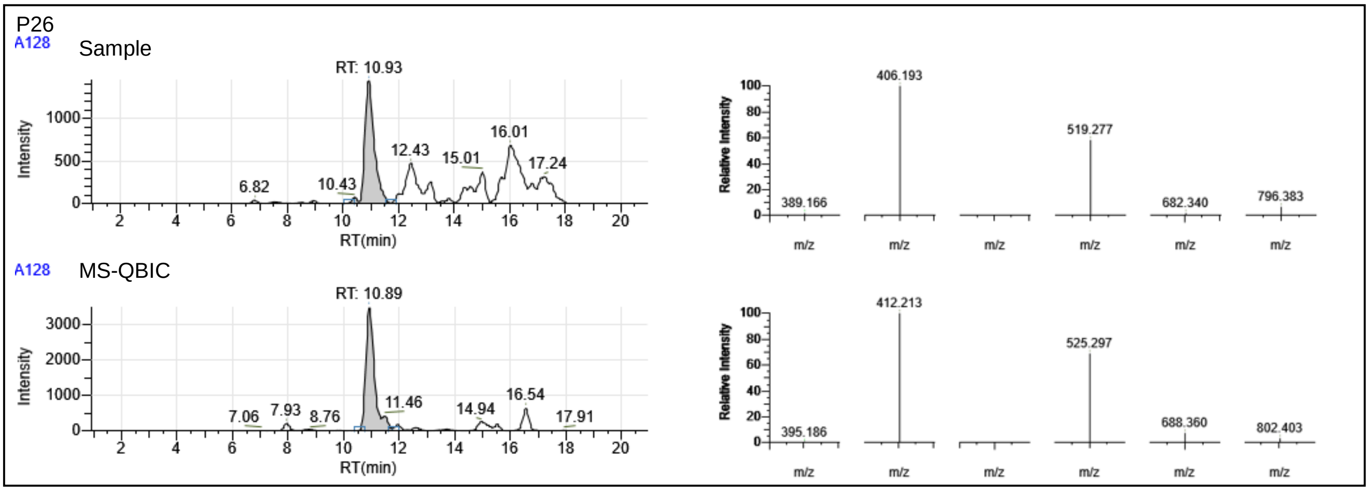
P26
A128 Sample
RT: 10.93
Intensity
RT(min)
6.82
10.43
12.43
15.01
16.01
17.24
Relative Intensity
m/z
389.166
406.193
519.277
682.340
796.383
A128 MS-QBIC
RT: 10.89
7.06
7.93
8.76
11.46
14.94
16.54
17.91
395.186
412.213
525.297
688.360
802.403

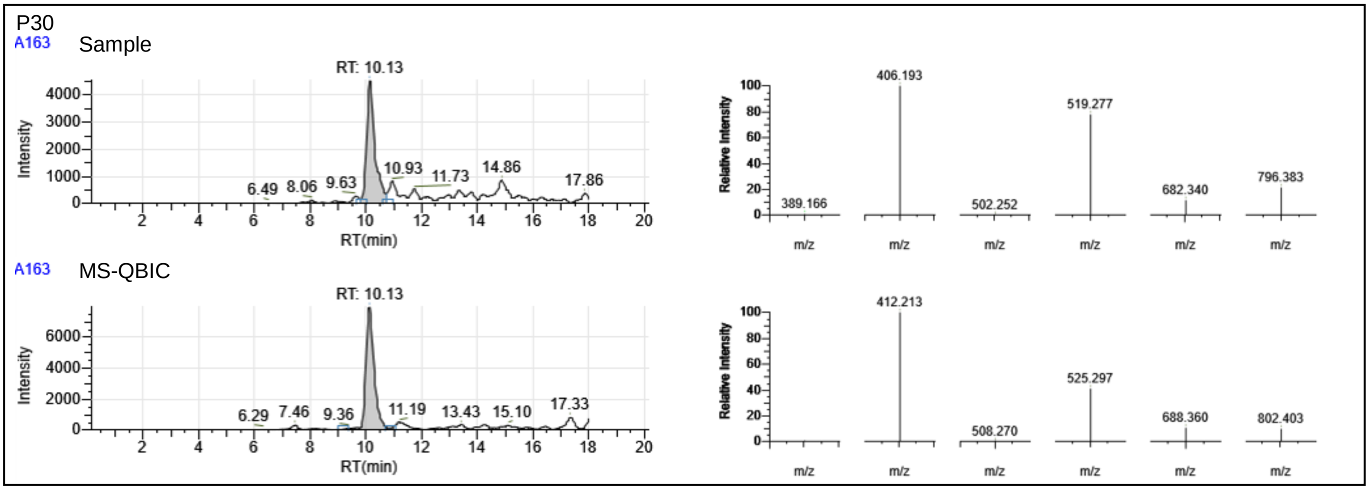
P30
A163 Sample
RT: 10.13
Intensity
RT(min)
6.49
8.06
9.63
10.93
11.73
14.86
17.86
Relative Intensity
m/z
389.166
406.193
502.252
519.277
682.340
796.383
A163 MS-QBIC
RT: 10.13
6.29
7.46
9.36
11.19
13.43
15.10
17.33
412.213
508.270
525.297
688.360
802.403

N. TTR (AADDTWEPFASGK) Another organ

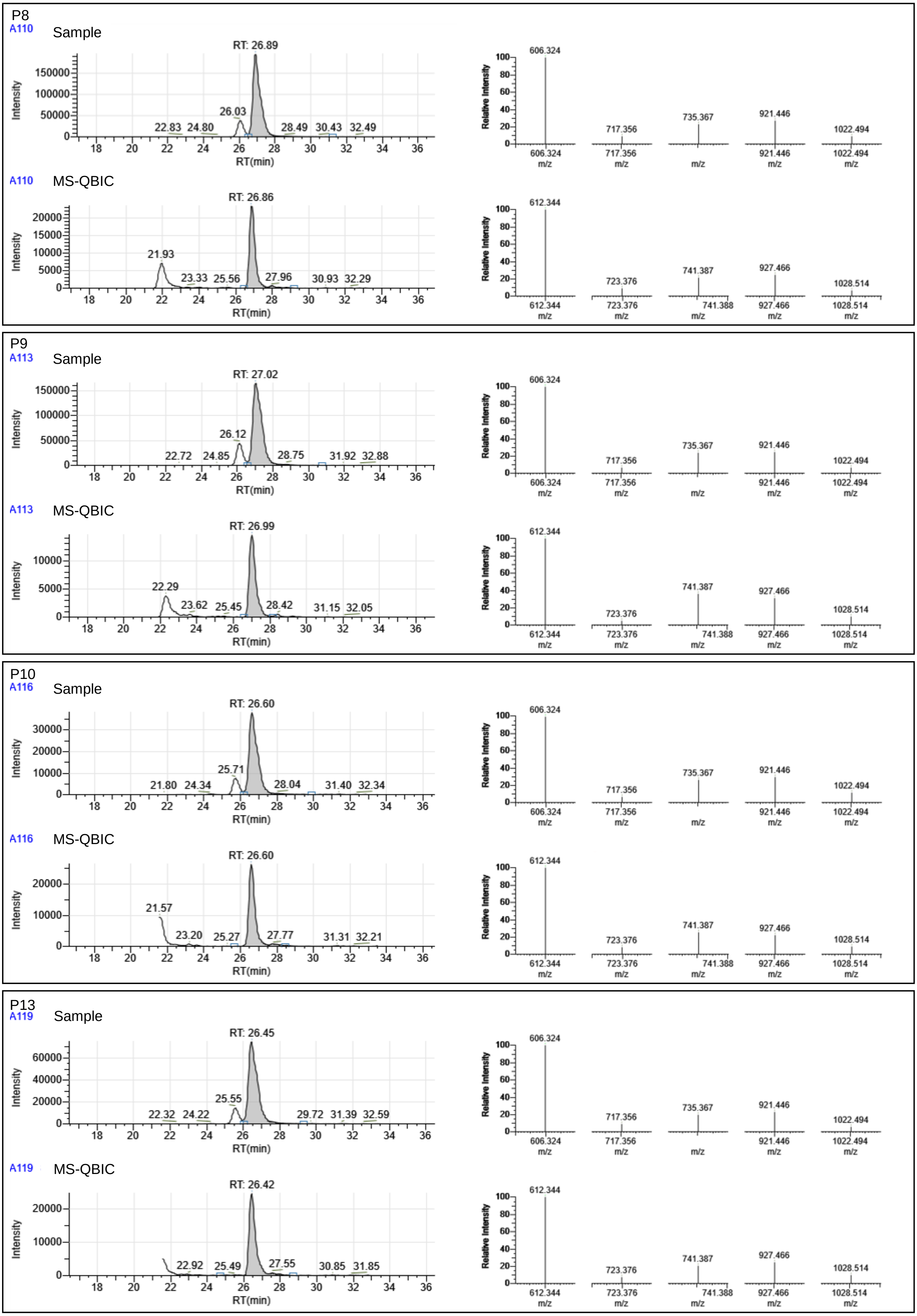

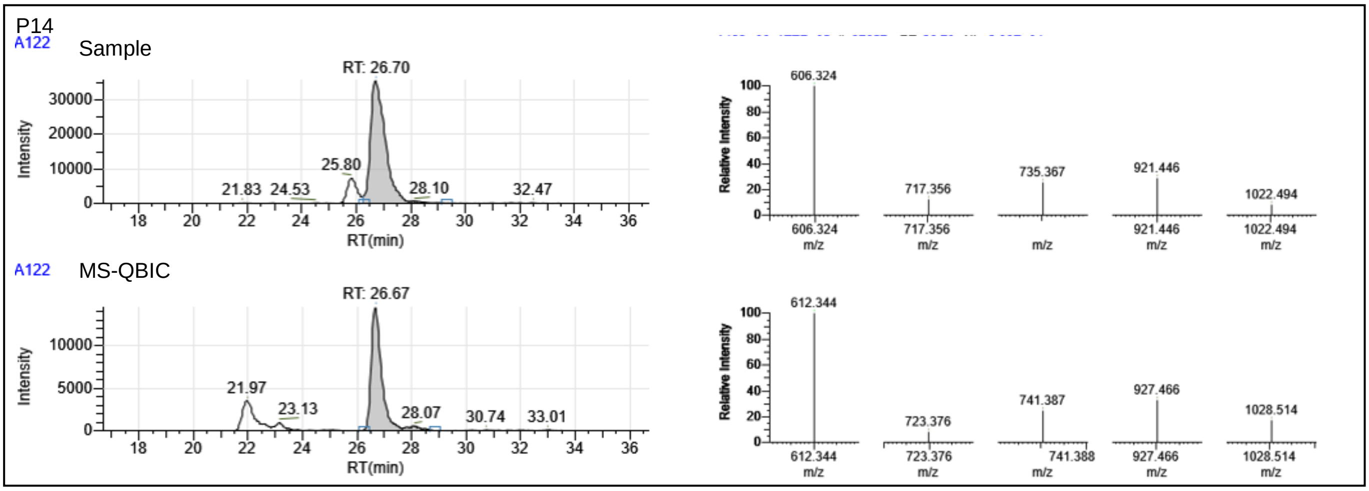
P14
A122 Sample
RT: 26.70
Intensity
30000
20000
10000
0
21.83 24.53 25.80 28.10 32.47
18 20 22 24 26 28 30 32 34 36
RT(min)
A122 MS-QBIC
RT: 26.67
10000
5000
0
21.97 23.13 28.07 30.74 33.01
RT(min)
Relative Intensity
606.324 717.356 735.367 921.446 1022.494
m/z
612.344 723.376 741.387 741.388 927.466 1028.514
m/z

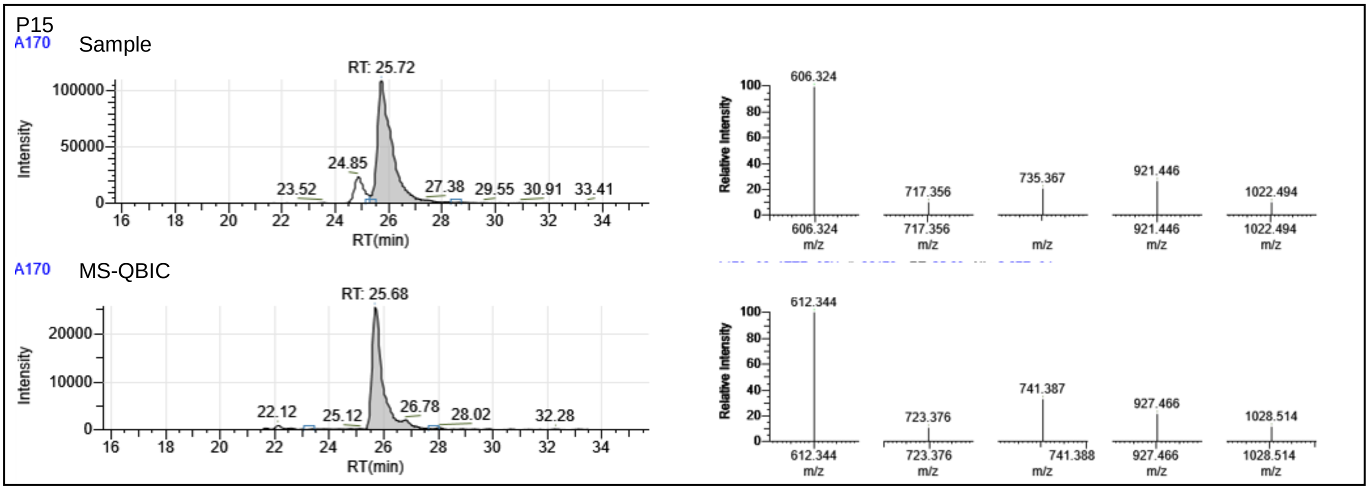
P15
A170 Sample
RT: 25.72
Intensity
100000
50000
0
23.52 24.85 27.38 29.55 30.91 33.41
16 18 20 22 24 26 28 30 32 34
RT(min)
A170 MS-QBIC
RT: 25.68
20000
10000
0
22.12 25.12 26.78 28.02 32.28
RT(min)
Relative Intensity
606.324 717.356 735.367 921.446 1022.494
m/z
612.344 723.376 741.387 741.388 927.466 1028.514
m/z

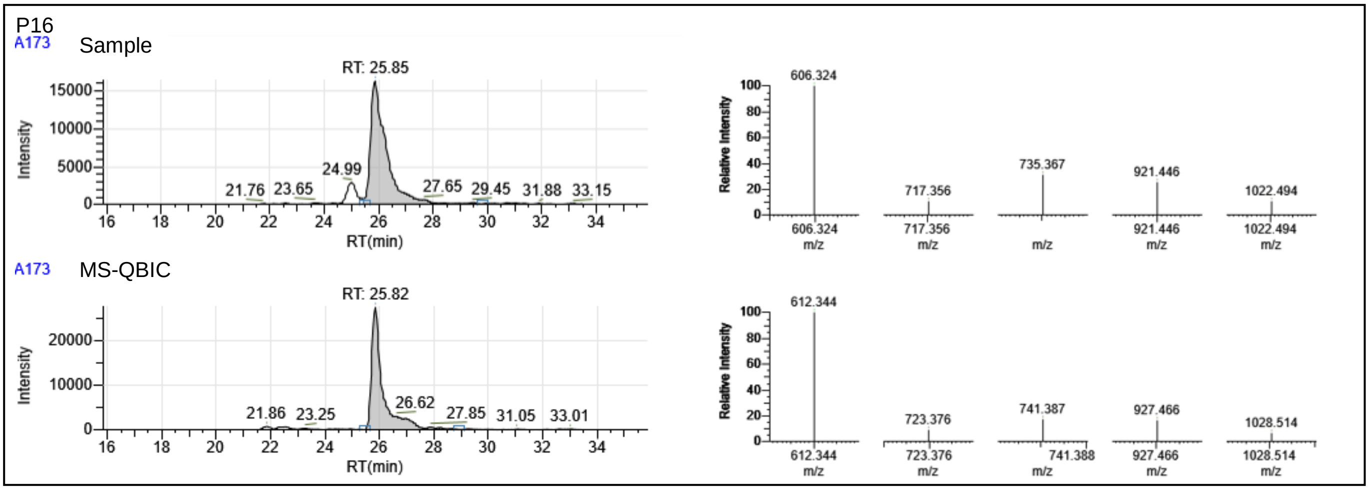
P16
A173 Sample
RT: 25.85
Intensity
15000
10000
5000
0
21.76 23.65 24.99 27.65 29.45 31.88 33.15
16 18 20 22 24 26 28 30 32 34
RT(min)
A173 MS-QBIC
RT: 25.82
20000
10000
0
21.86 23.25 26.62 27.85 31.05 33.01
RT(min)
Relative Intensity
606.324 717.356 735.367 921.446 1022.494
m/z
612.344 723.376 741.387 741.388 927.466 1028.514
m/z

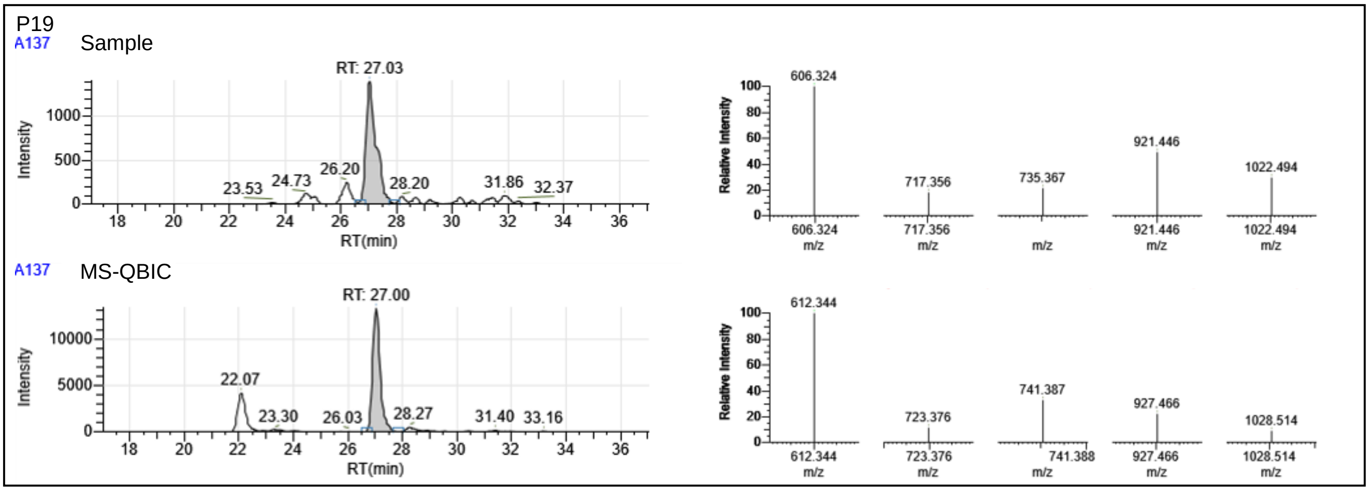
P19
A137 Sample
RT: 27.03
Intensity
1000
500
0
23.53 24.73 26.20 28.20 31.86 32.37
18 20 22 24 26 28 30 32 34 36
RT(min)
A137 MS-QBIC
RT: 27.00
10000
5000
0
22.07 23.30 26.03 28.27 31.40 33.16
RT(min)
Relative Intensity
606.324 717.356 735.367 921.446 1022.494
m/z
612.344 723.376 741.387 741.388 927.466 1028.514
m/z

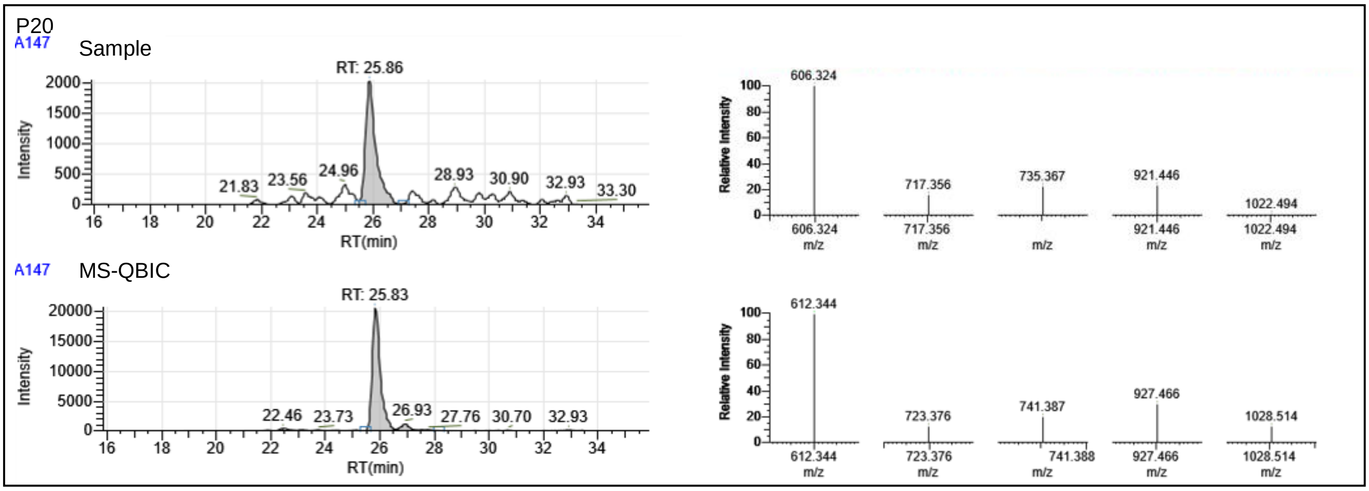
P20
A147 Sample
RT: 25.86
21.83 23.56 24.96 28.93 30.90 32.93 33.30
Intensity
RT(min)
606.324
717.356
735.367
921.446
1022.494
Relative Intensity
m/z
A147 MS-QBIC
RT: 25.83
22.46 23.73 26.93 27.76 30.70 32.93
612.344
723.376
741.387
741.388
927.466
1028.514

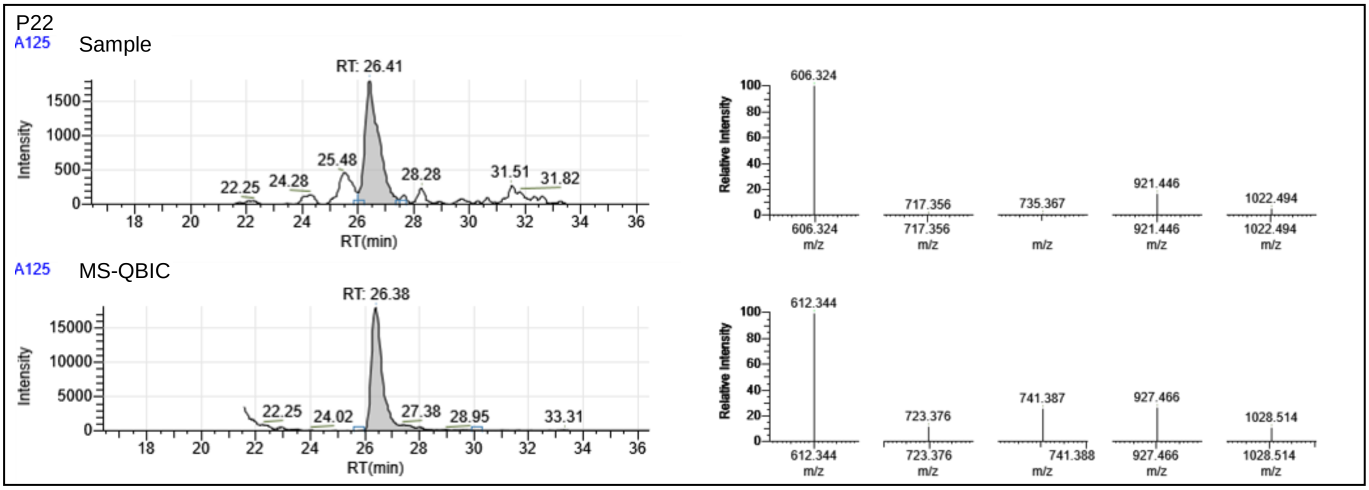
P22
A125 Sample
RT: 26.41
22.25 24.28 25.48 28.28 31.51 31.82
Intensity
RT(min)
606.324
717.356
735.367
921.446
1022.494
Relative Intensity
m/z
A125 MS-QBIC
RT: 26.38
22.25 24.02 27.38 28.95 33.31
612.344
723.376
741.387
741.388
927.466
1028.514

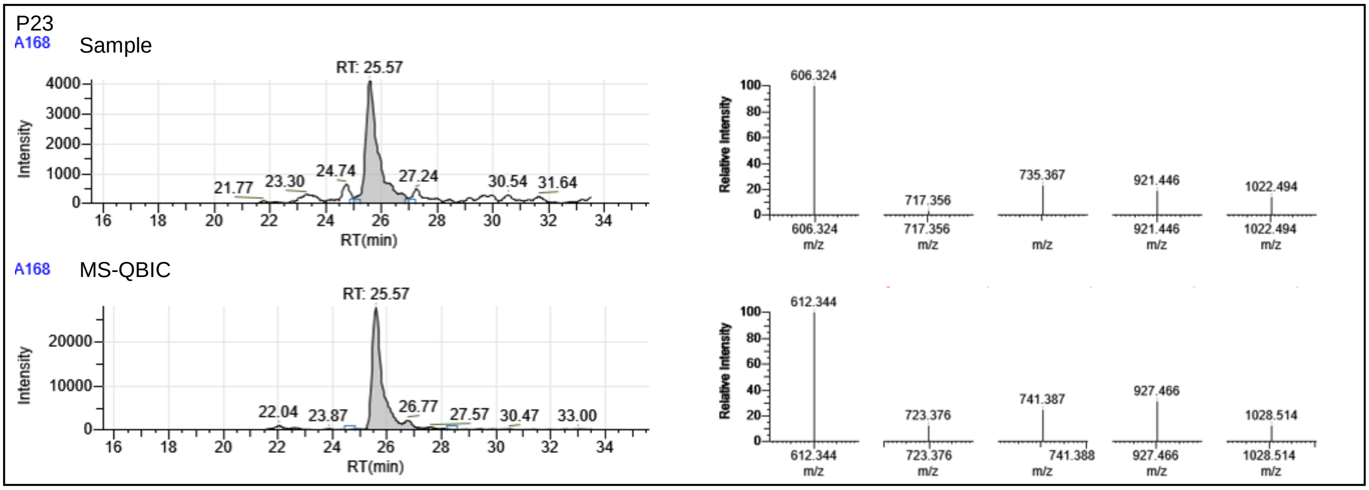
P23
A168 Sample
RT: 25.57
21.77 23.30 24.74 27.24 30.54 31.64
Intensity
RT(min)
606.324
717.356
735.367
921.446
1022.494
Relative Intensity
m/z
A168 MS-QBIC
RT: 25.57
22.04 23.87 26.77 27.57 30.47 33.00
612.344
723.376
741.387
741.388
927.466
1028.514

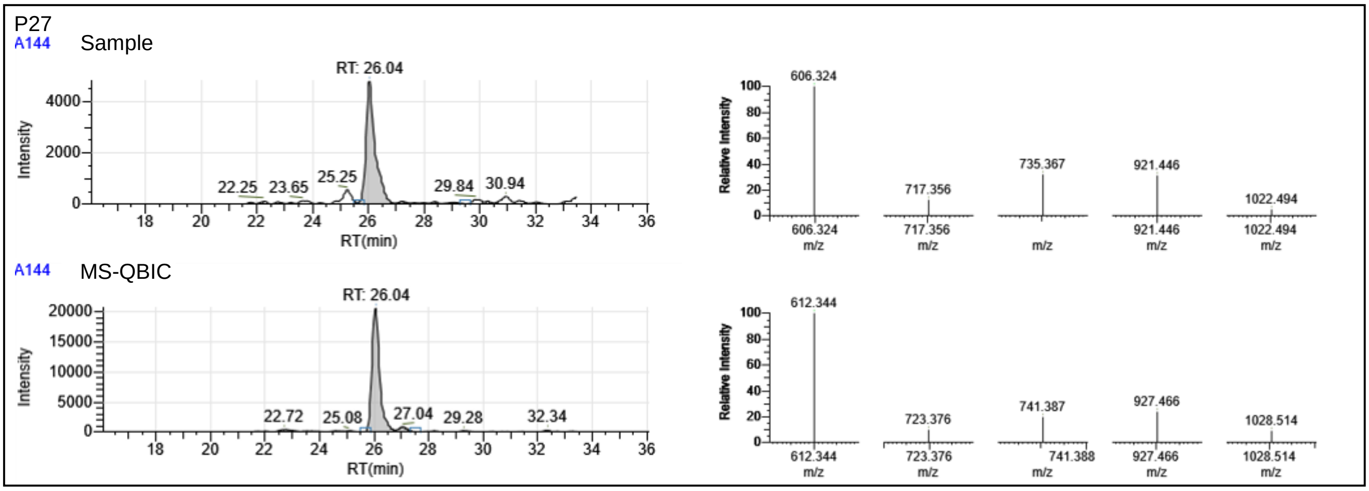
P27
A144 Sample
RT: 26.04
22.25 23.65 25.25 29.84 30.94
Intensity
RT(min)
606.324
717.356
735.367
921.446
1022.494
Relative Intensity
m/z
A144 MS-QBIC
RT: 26.04
22.72 25.08 27.04 29.28 32.34
612.344
723.376
741.387
741.388
927.466
1028.514

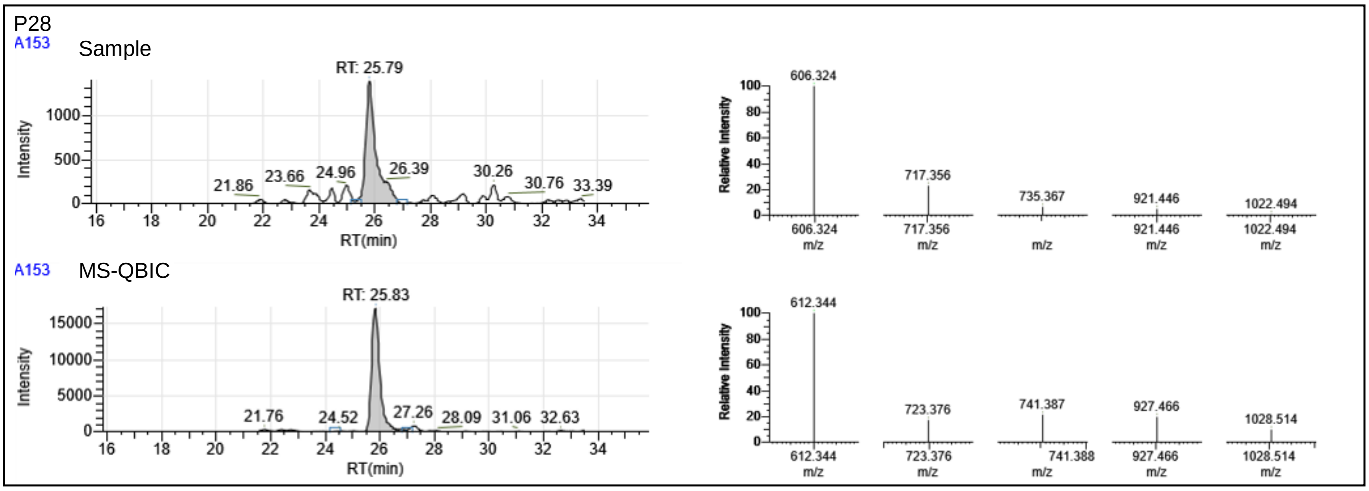
P28
A153 Sample
RT: 25.79
21.86
23.66
24.96
26.39
30.26
30.76
33.39
Intensity
RT(min)
606.324
717.356
735.367
921.446
1022.494
Relative Intensity
m/z
A153 MS-QBIC
RT: 25.83
21.76
24.52
27.26
28.09
31.06
32.63
612.344
723.376
741.387
741.388
927.466
1028.514

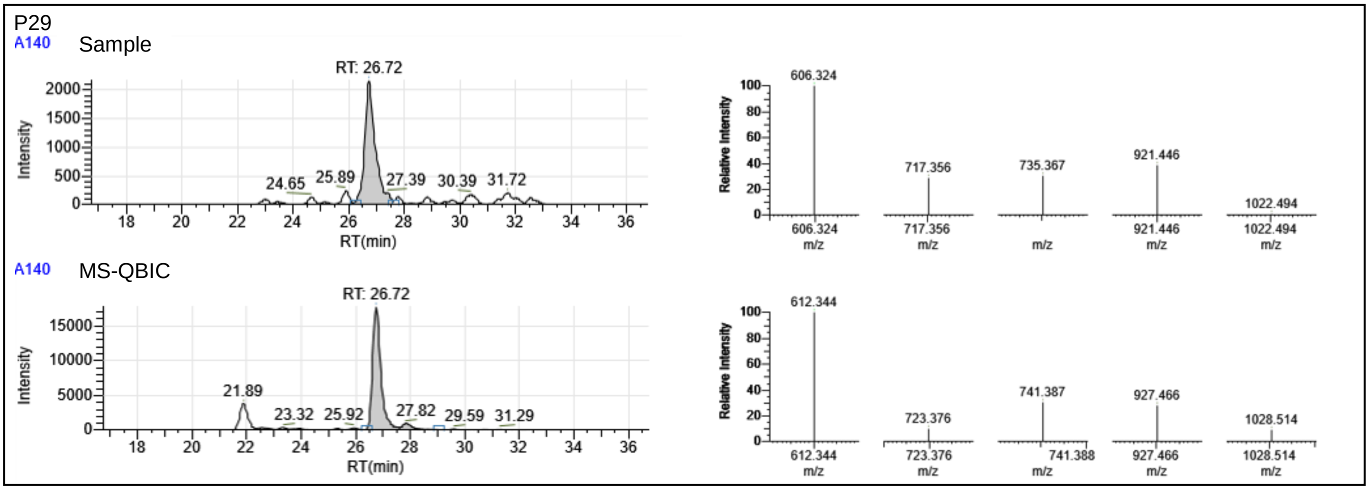
P29
A140 Sample
RT: 26.72
24.65
25.89
27.39
30.39
31.72
Intensity
RT(min)
606.324
717.356
735.367
921.446
1022.494
Relative Intensity
m/z
A140 MS-QBIC
RT: 26.72
21.89
23.32
25.92
27.82
29.59
31.29
612.344
723.376
741.387
741.388
927.466
1028.514

O IGK (TVAAPSVFIFPPSDEQLK) Another organ

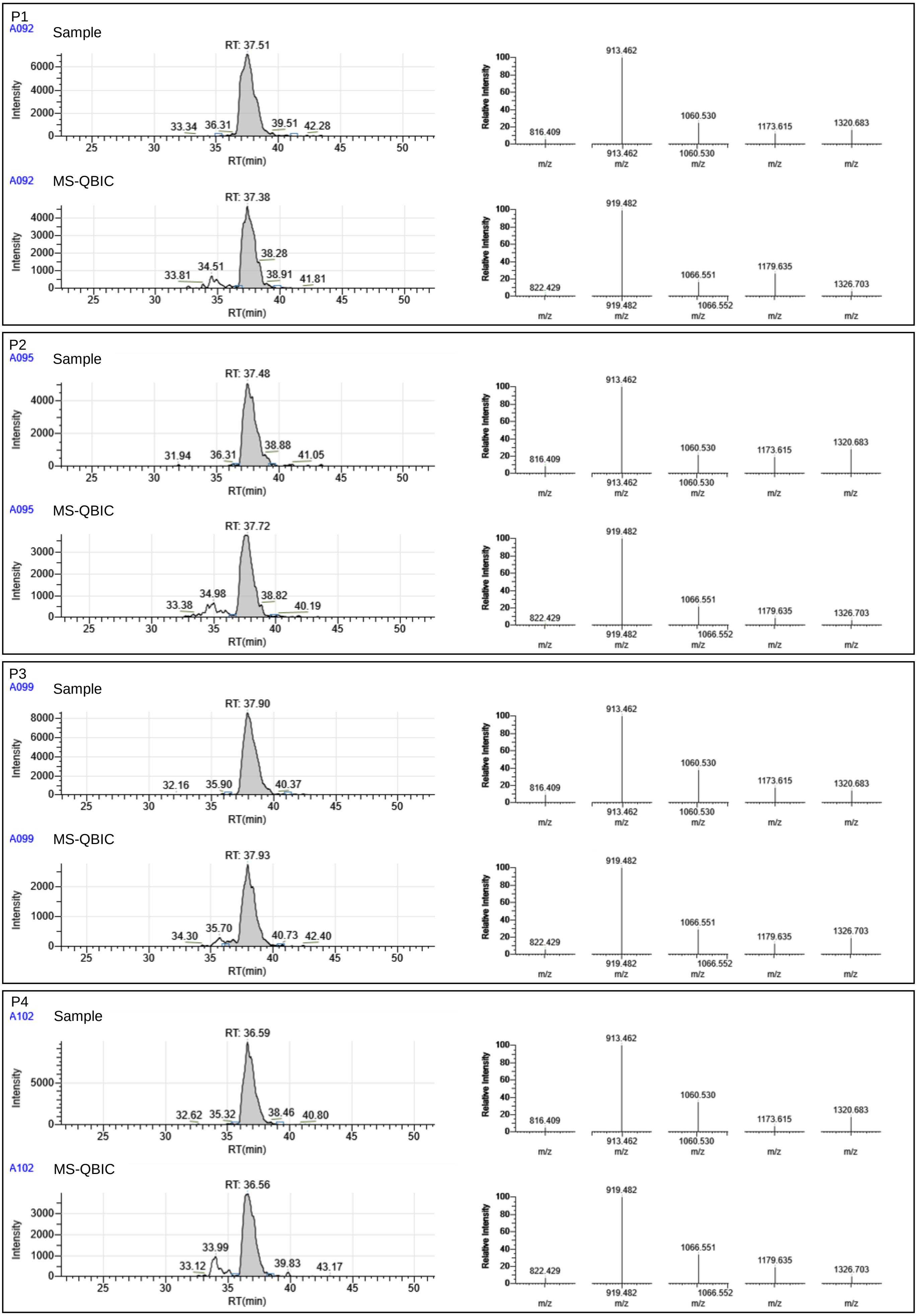

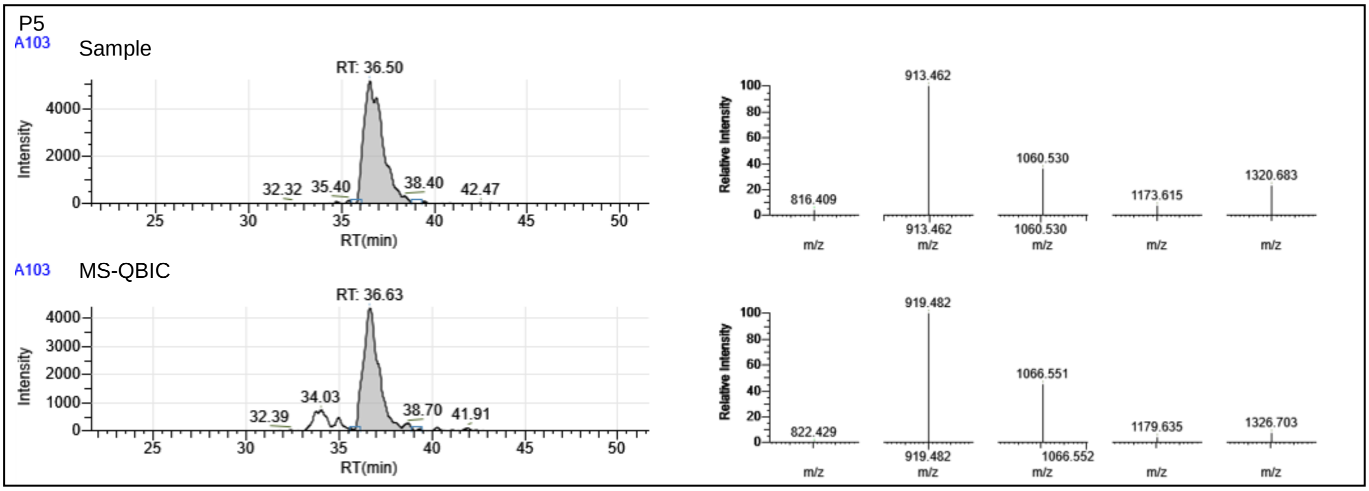
P5
A103 Sample
RT: 36.50
32.32 35.40 38.40 42.47
Intensity
RT(min)
Relative Intensity
913.462
1060.530
816.409
1173.615
1320.683
m/z
A103 MS-QBIC
RT: 36.63
34.03
32.39 38.70 41.91
919.482
1066.551
1066.552
822.429
1179.635
1326.703

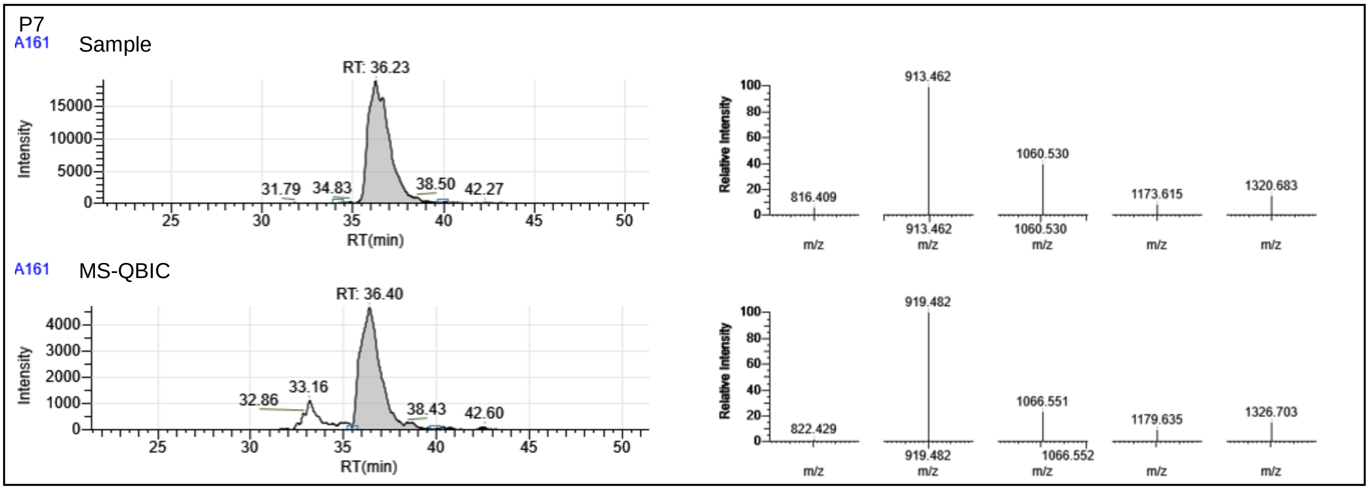
P7
A161 Sample
RT: 36.23
31.79 34.83 38.50 42.27
Intensity
RT(min)
Relative Intensity
913.462
1060.530
816.409
1173.615
1320.683
m/z
A161 MS-QBIC
RT: 36.40
33.16
32.86 38.43 42.60
919.482
1066.551
1066.552
822.429
1179.635
1326.703

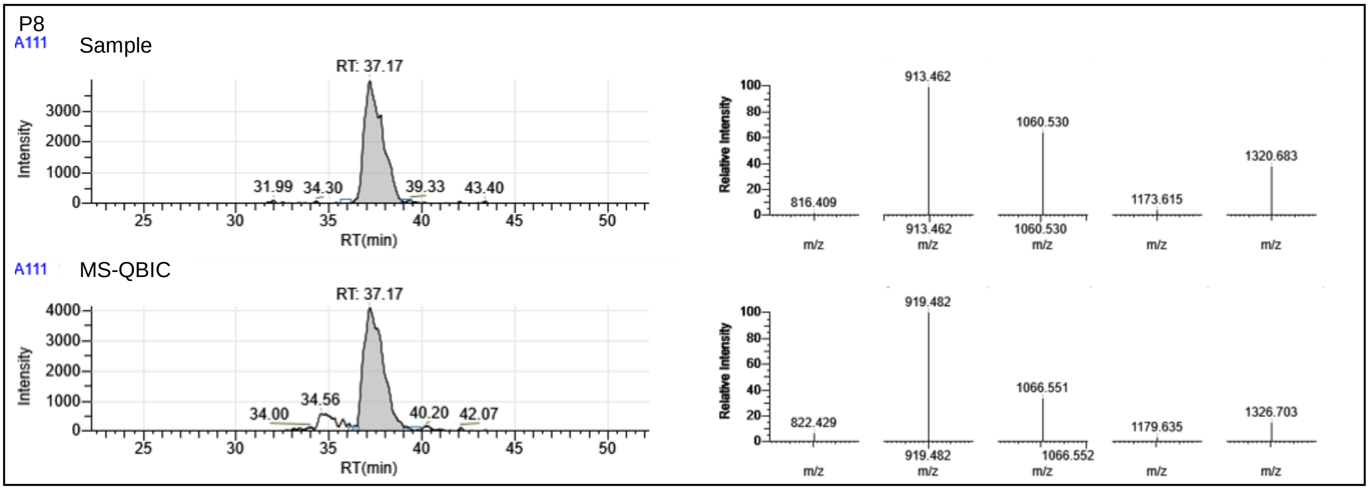
P8
A111 Sample
RT: 37.17
31.99 34.30 39.33 43.40
Intensity
RT(min)
Relative Intensity
913.462
1060.530
816.409
1173.615
1320.683
m/z
A111 MS-QBIC
RT: 37.17
34.56
34.00 40.20 42.07
919.482
1066.551
1066.552
822.429
1179.635
1326.703

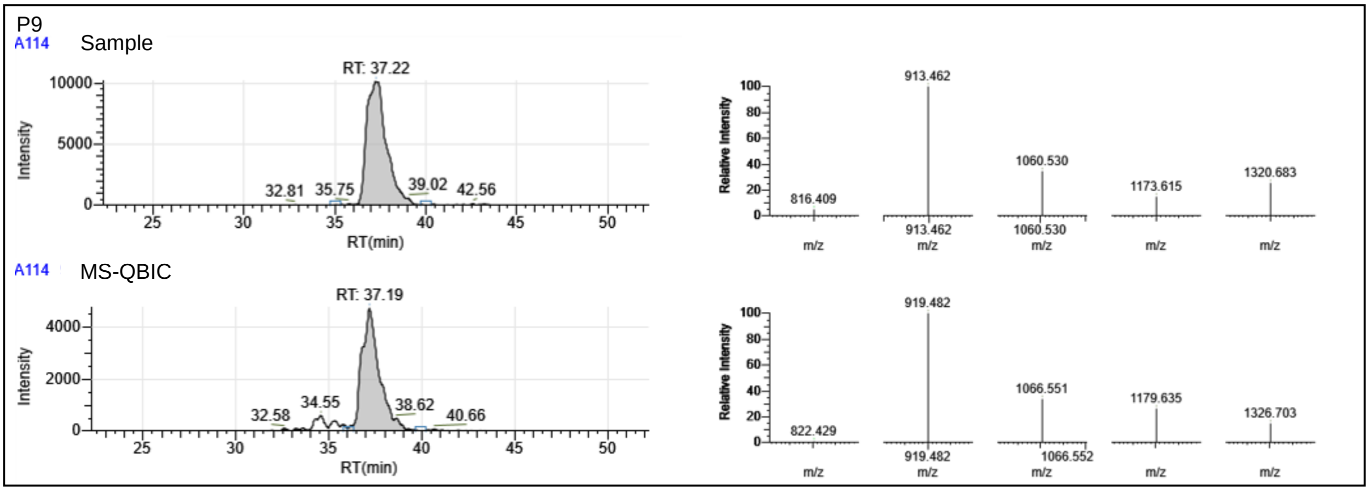
P9
A114 Sample
RT: 37.22
32.81 35.75 39.02 42.56
Intensity
RT(min)
Relative Intensity
913.462
1060.530
816.409
1173.615
1320.683
m/z
A114 MS-QBIC
RT: 37.19
34.55
32.58 38.62 40.66
919.482
1066.551
1066.552
822.429
1179.635
1326.703

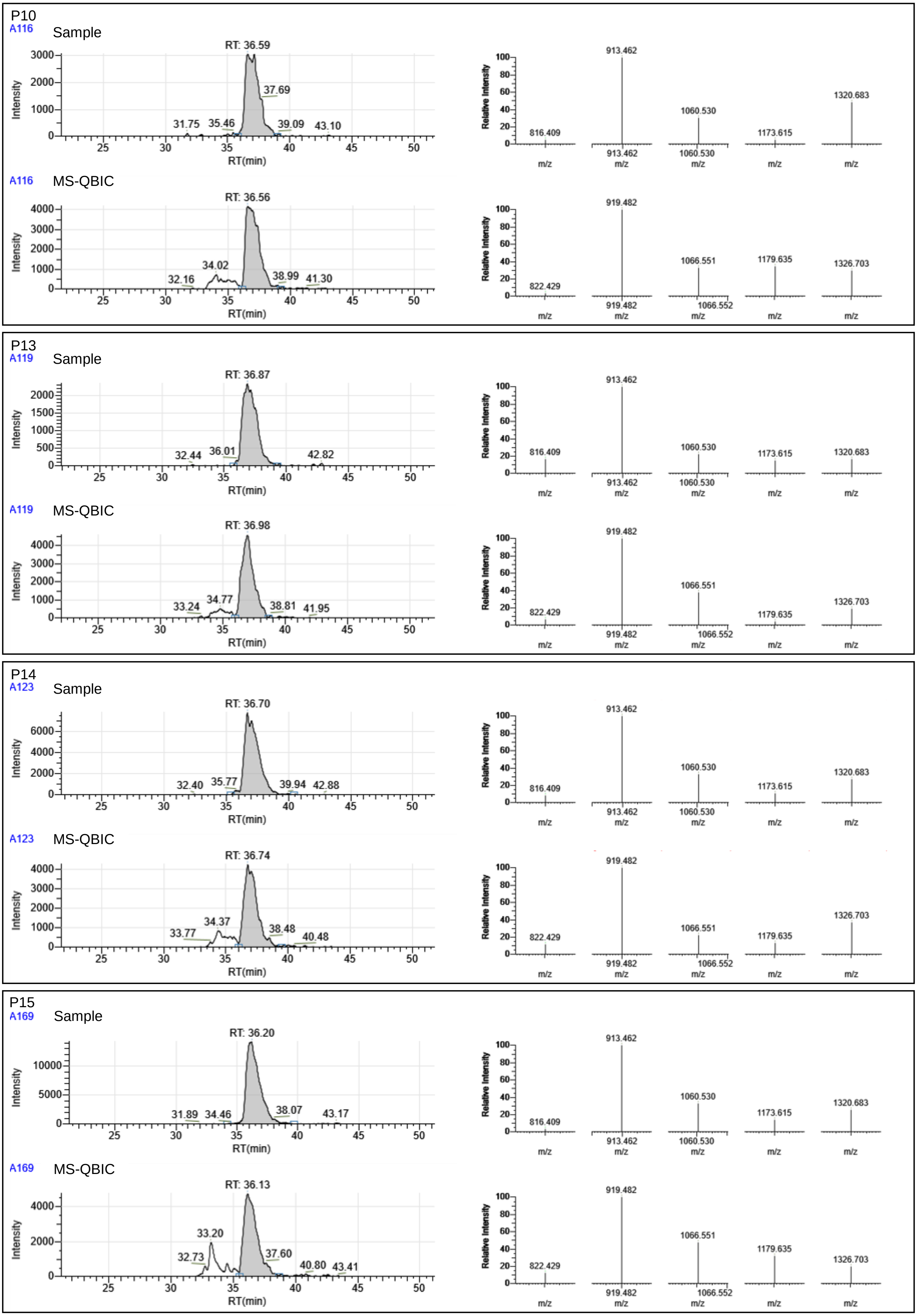
P10
A116 Sample
RT: 36.59
31.75 35.46 37.69 39.09 43.10
Intensity
RT(min)
Relative Intensity
913.462
1060.530
1320.683
816.409
1173.615
m/z
A116 MS-QBIC
RT: 36.56
32.16 34.02 38.99 41.30
919.482
1066.551
1179.635
1326.703
822.429
1066.552
P13
A119 Sample
RT: 36.87
32.44 36.01 42.82
A119 MS-QBIC
RT: 36.98
33.24 34.77 38.81 41.95
P14
A123 Sample
RT: 36.70
32.40 35.77 39.94 42.88
A123 MS-QBIC
RT: 36.74
33.77 34.37 38.48 40.48
P15
A169 Sample
RT: 36.20
31.89 34.46 38.07 43.17
A169 MS-QBIC
RT: 36.13
32.73 33.20 37.60 40.80 43.41

P16

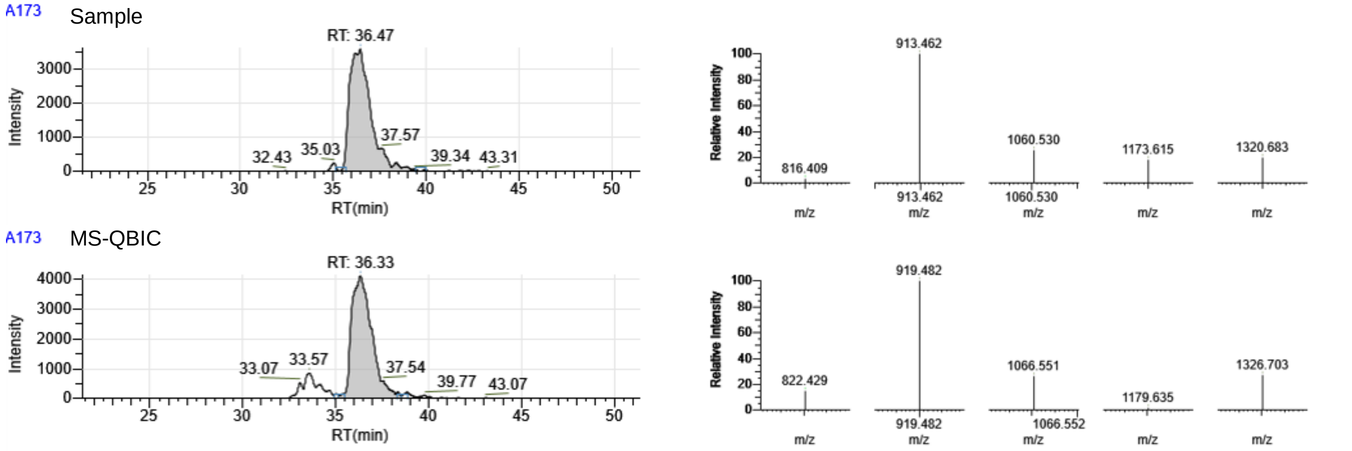

P17

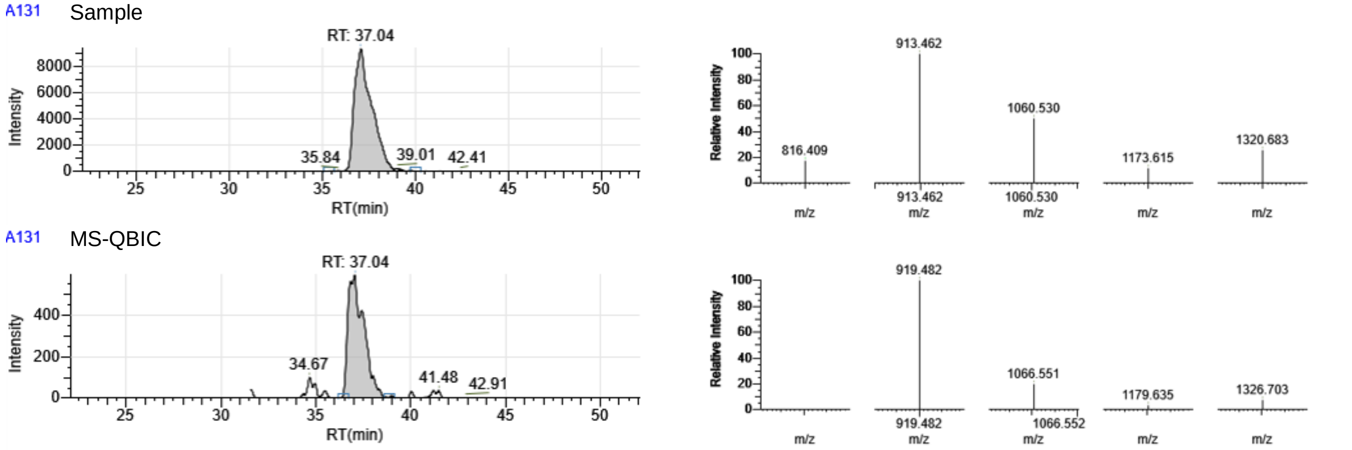

P18

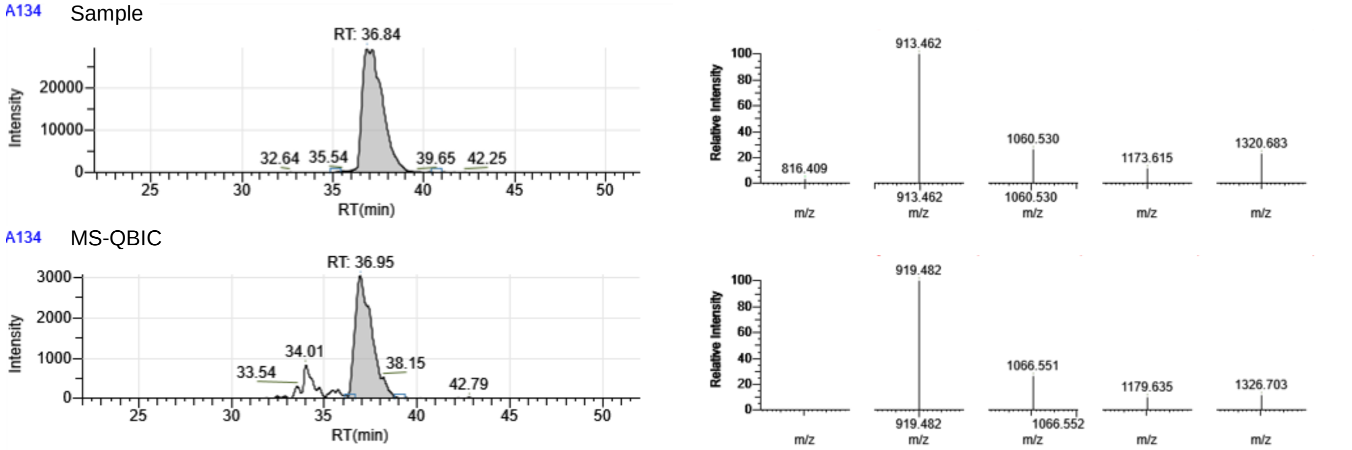

P19

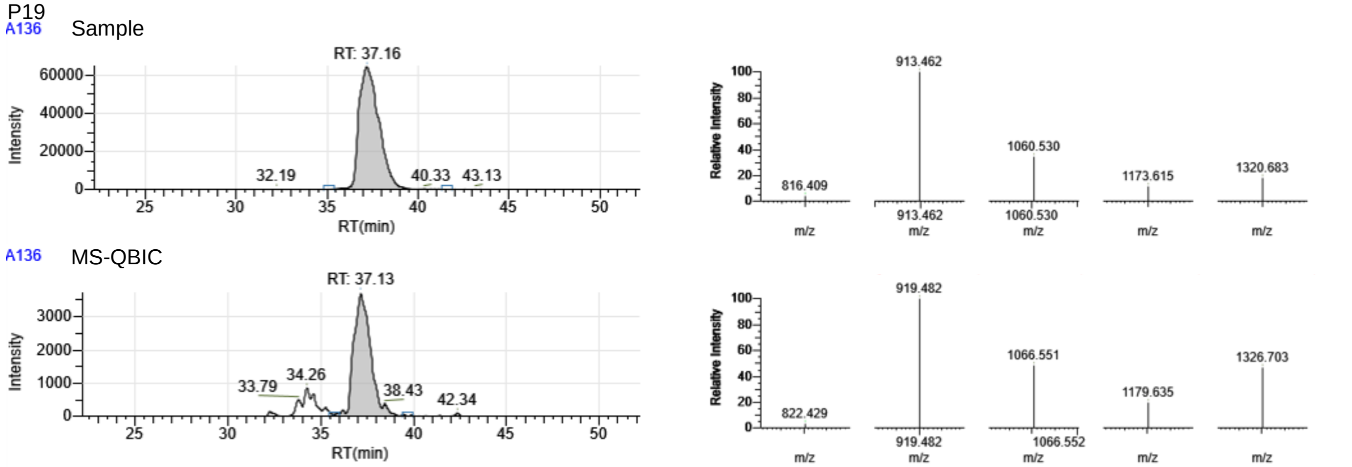

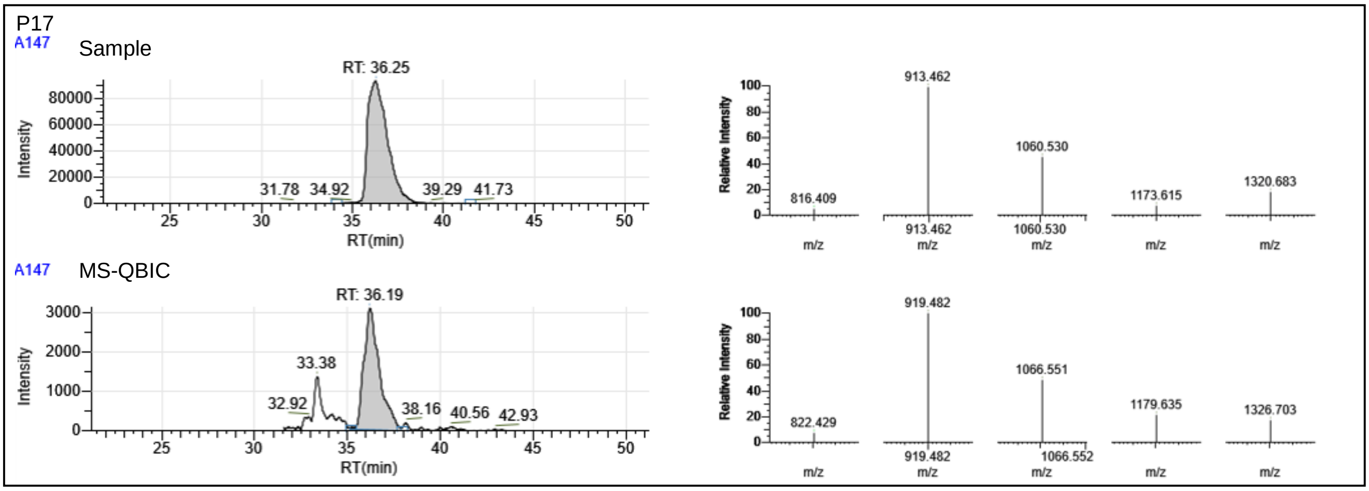
P17
A147 Sample
RT: 36.25
31.78 34.92 39.29 41.73
Intensity
RT(min)
Relative Intensity
816.409 913.462 1060.530 1173.615 1320.683
m/z
A147 MS-QBIC
RT: 36.19
33.38 32.92 38.16 40.56 42.93
822.429 919.482 1066.551 1066.552 1179.635 1326.703

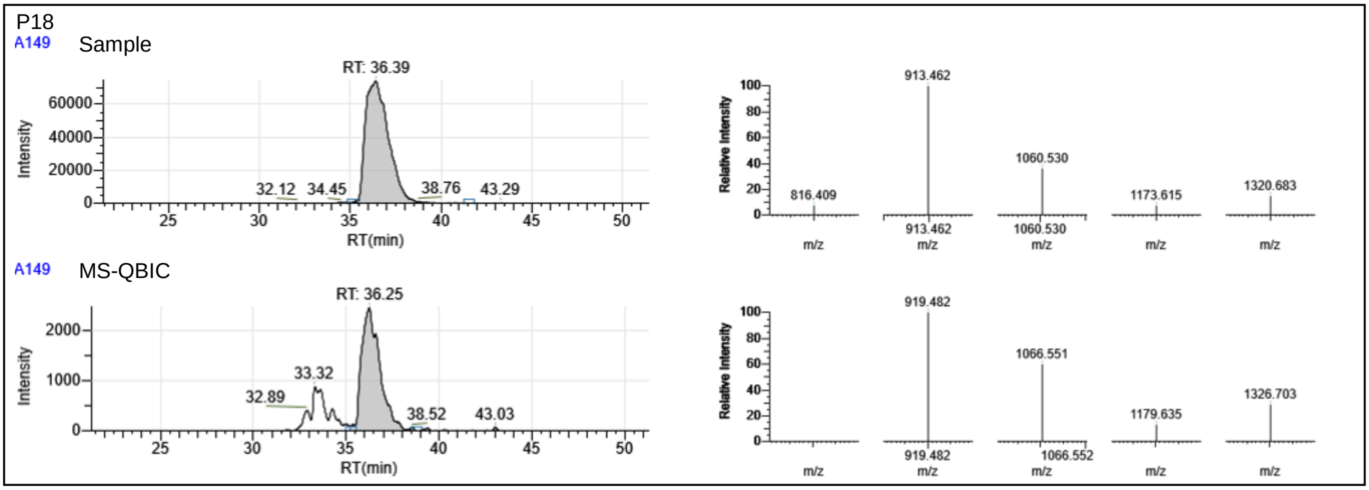
P18
A149 Sample
RT: 36.39
32.12 34.45 38.76 43.29
Intensity
RT(min)
Relative Intensity
816.409 913.462 1060.530 1173.615 1320.683
m/z
A149 MS-QBIC
RT: 36.25
33.32 32.89 38.52 43.03
919.482 1066.551 1066.552 1179.635 1326.703

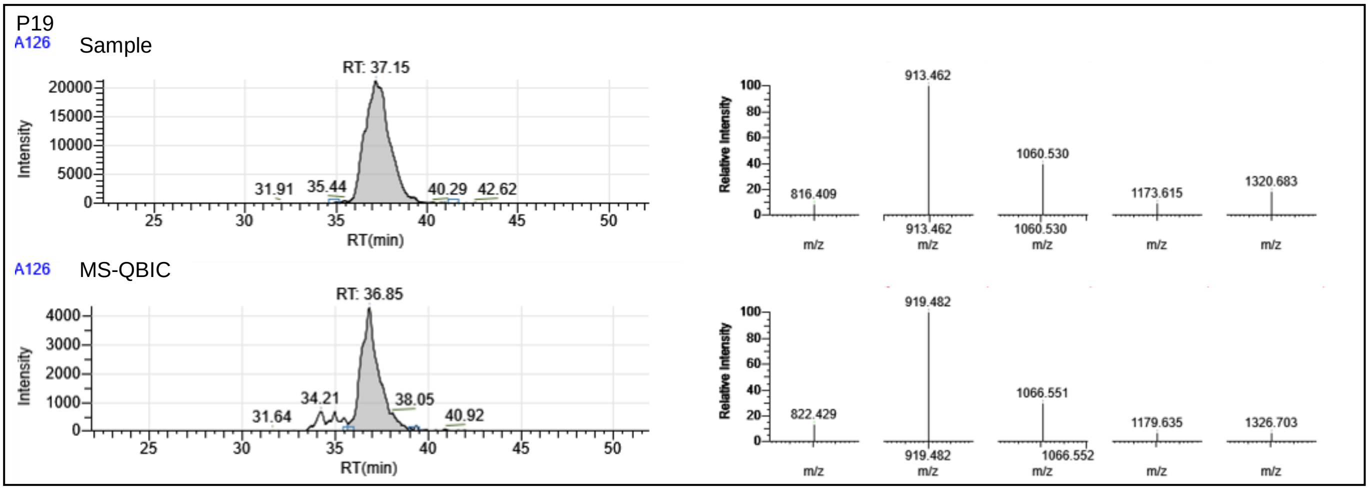
P19
A126 Sample
RT: 37.15
31.91 35.44 40.29 42.62
Intensity
RT(min)
Relative Intensity
816.409 913.462 1060.530 1173.615 1320.683
m/z
A126 MS-QBIC
RT: 36.85
34.21 31.64 38.05 40.92
822.429 919.482 1066.551 1066.552 1179.635 1326.703

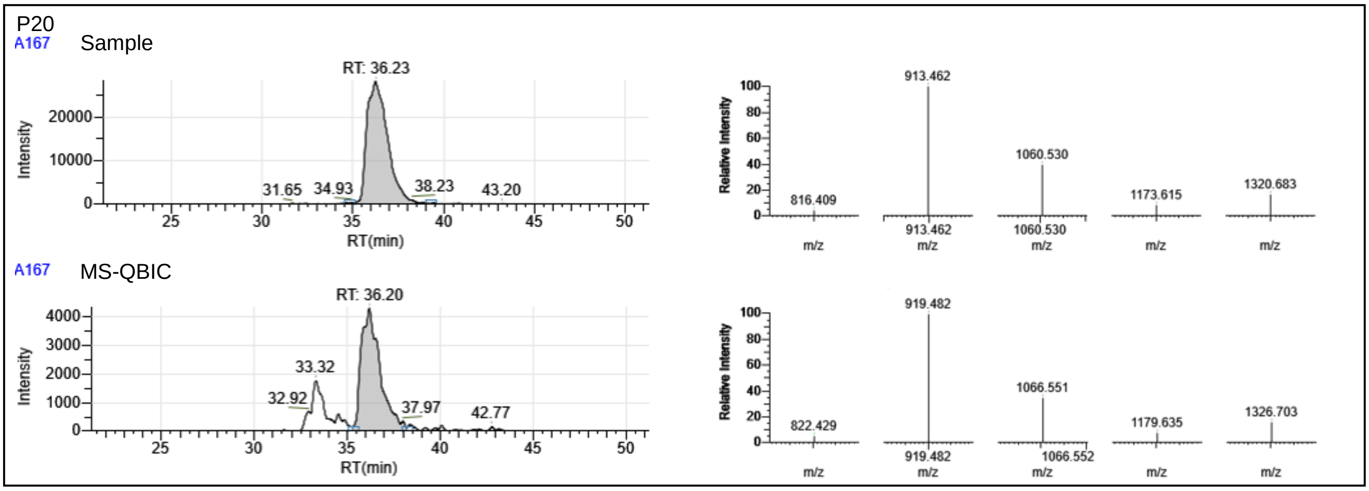
P20
A167 Sample
RT: 36.23
31.65 34.93 38.23 43.20
Intensity
RT(min)
Relative Intensity
816.409 913.462 1060.530 1173.615 1320.683
m/z
A167 MS-QBIC
RT: 36.20
33.32 32.92 37.97 42.77
822.429 919.482 1066.551 1066.552 1179.635 1326.703

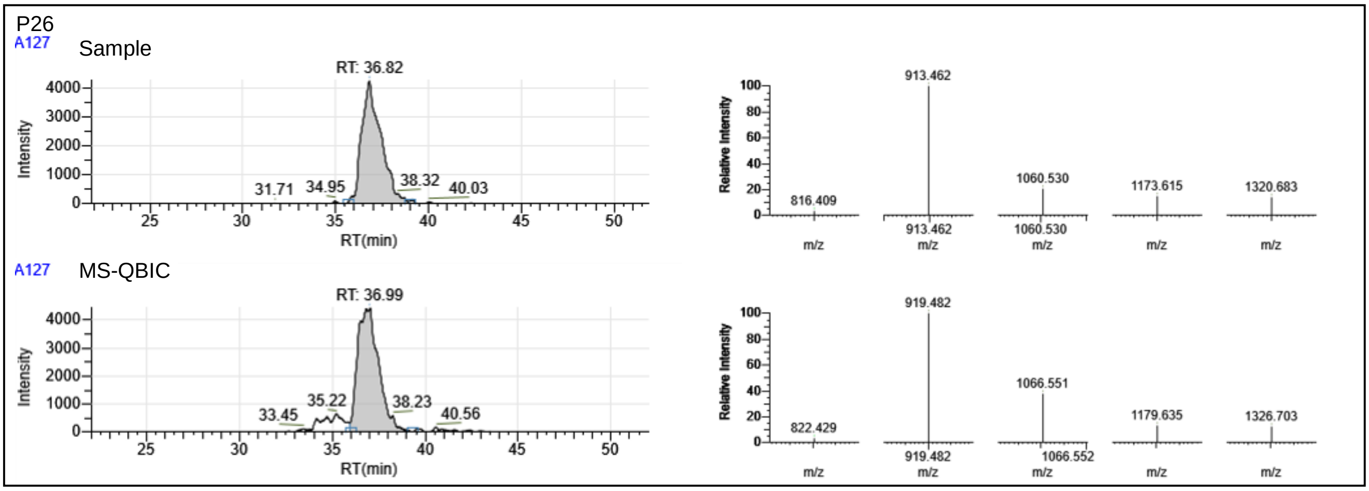
P26
A127 Sample
RT: 36.82
31.71 34.95 38.32 40.03
Intensity
RT(min)
913.462
1060.530
816.409
1173.615
1320.683
Relative Intensity
m/z
A127 MS-QBIC
RT: 36.99
33.45 35.22 38.23 40.56
919.482
1066.551
1066.552
822.429
1179.635
1326.703

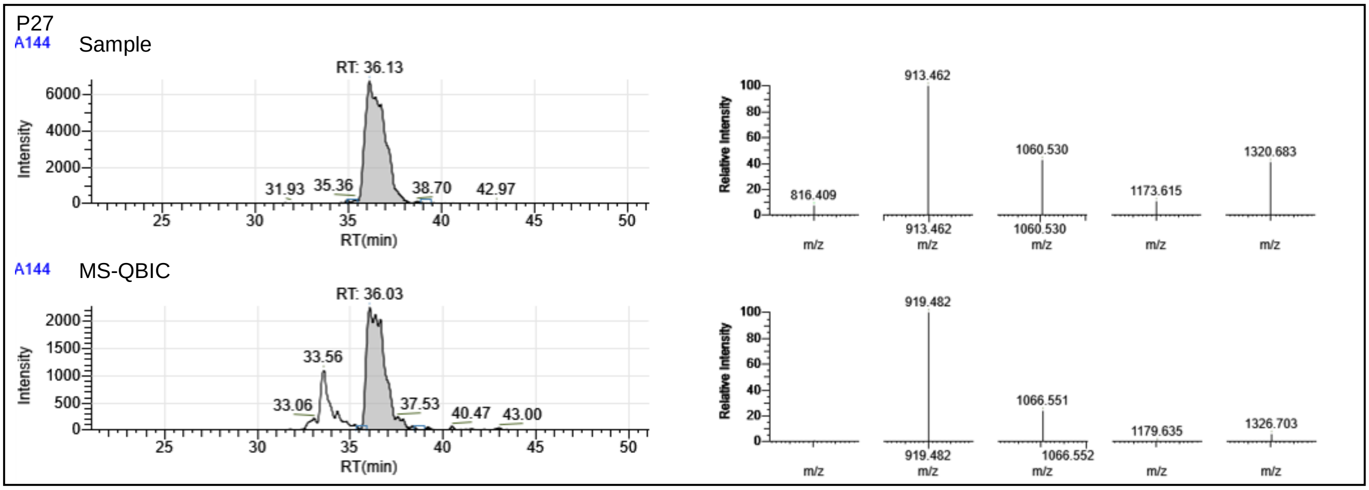
P27
A144 Sample
RT: 36.13
31.93 35.36 38.70 42.97
Intensity
RT(min)
913.462
1060.530
816.409
1173.615
1320.683
Relative Intensity
m/z
A144 MS-QBIC
RT: 36.03
33.56 33.06 37.53 40.47 43.00
919.482
1066.551
1066.552
1179.635
1326.703

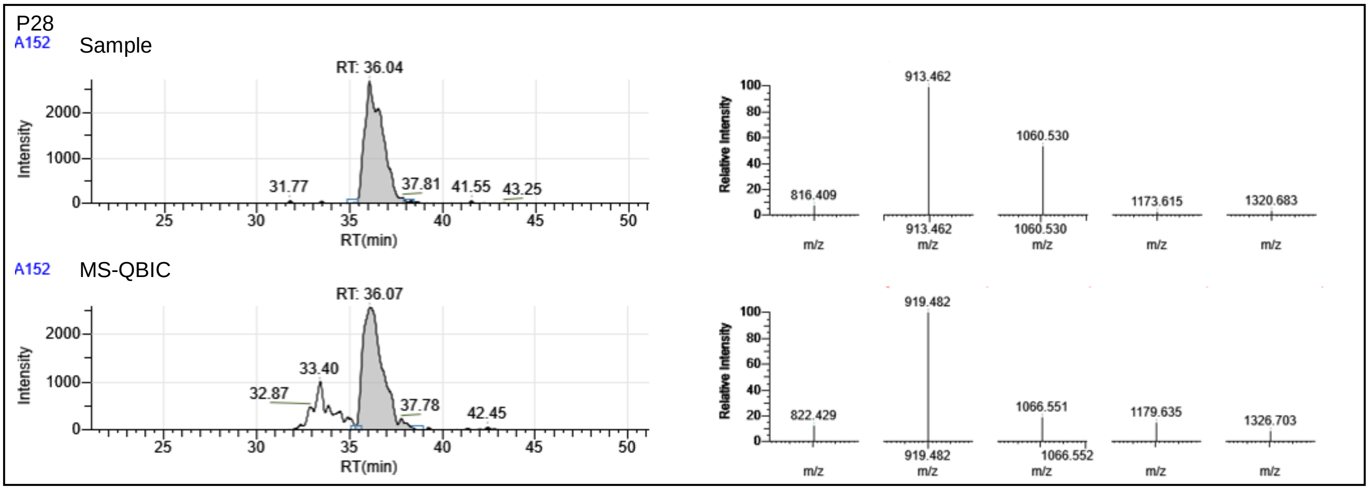
P28
A152 Sample
RT: 36.04
31.77 37.81 41.55 43.25
Intensity
RT(min)
913.462
1060.530
816.409
1173.615
1320.683
Relative Intensity
m/z
A152 MS-QBIC
RT: 36.07
33.40 32.87 37.78 42.45
919.482
1066.551
1066.552
822.429
1179.635
1326.703

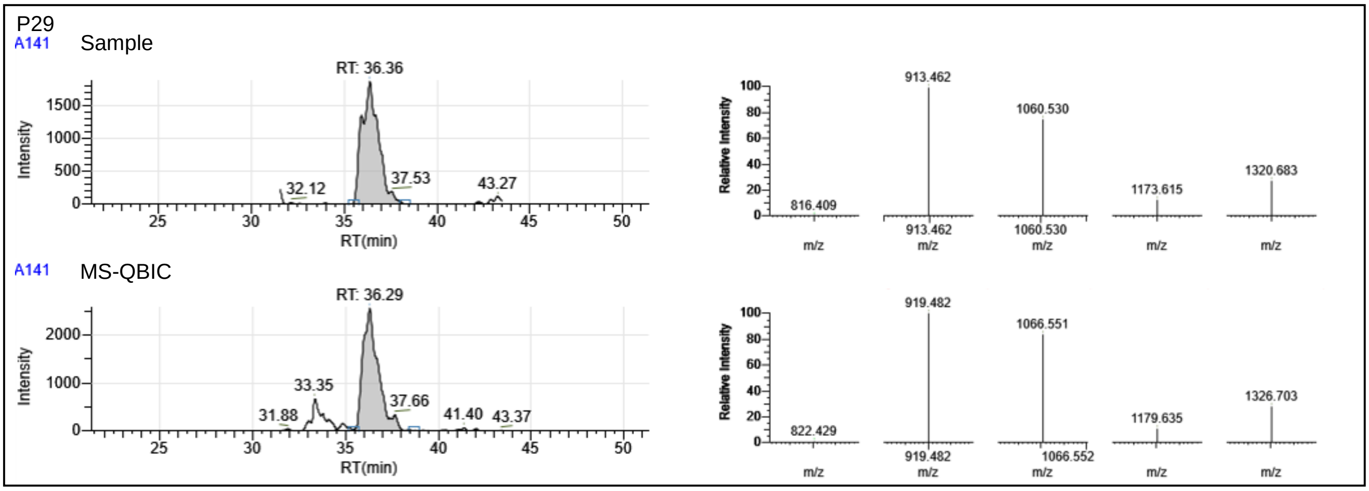
P29
A141 Sample
RT: 36.36
32.12 37.53 43.27
Intensity
RT(min)
913.462
1060.530
816.409
1173.615
1320.683
Relative Intensity
m/z
A141 MS-QBIC
RT: 36.29
33.35 31.88 37.66 41.40 43.37
919.482
1066.551
1066.552
822.429
1179.635
1326.703

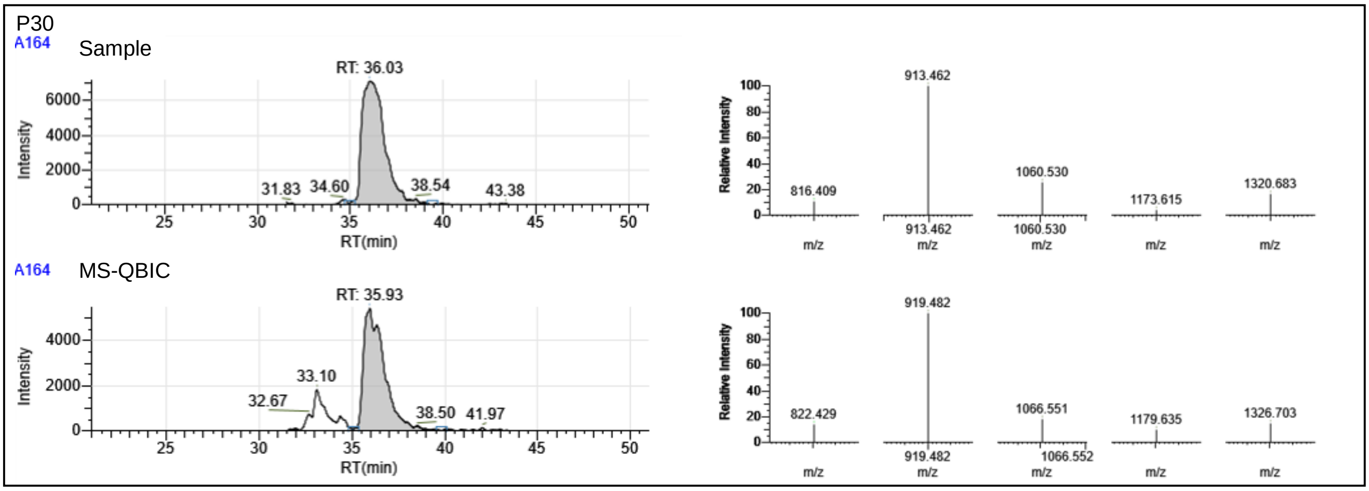

P30
A164
Sample
RT: 36.03
31.83
34.60
38.54
43.38
Intensity
RT(min)
816.409
913.462
1060.530
1173.615
1320.683
Relative Intensity
m/z
A164
MS-QBIC
RT: 35.93
33.10
32.67
38.50
41.97
822.429
919.482
1066.551
1066.552
1179.635
1326.703

P IGL (YAASSYLSLTPEQWK) Another organ

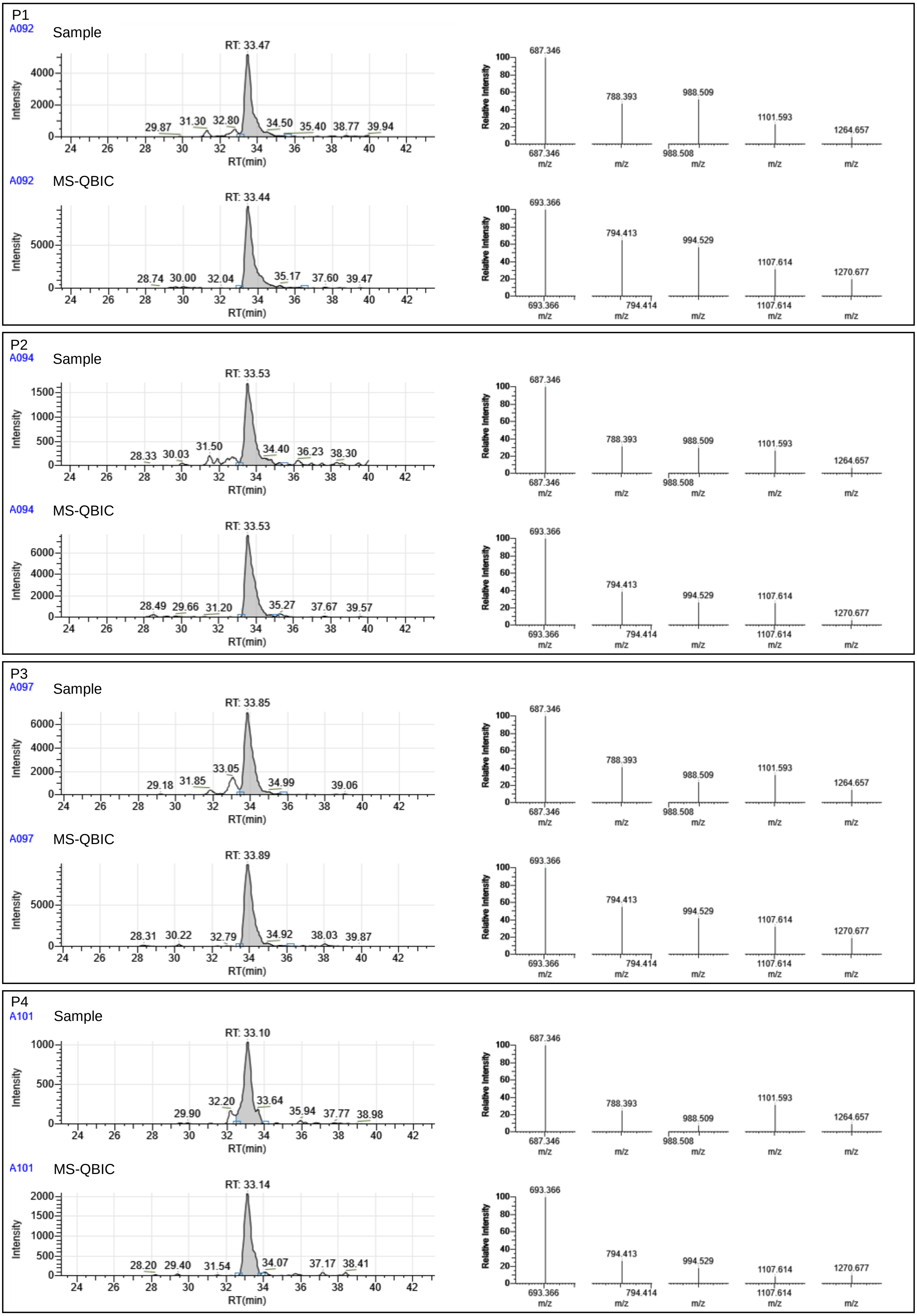

P5

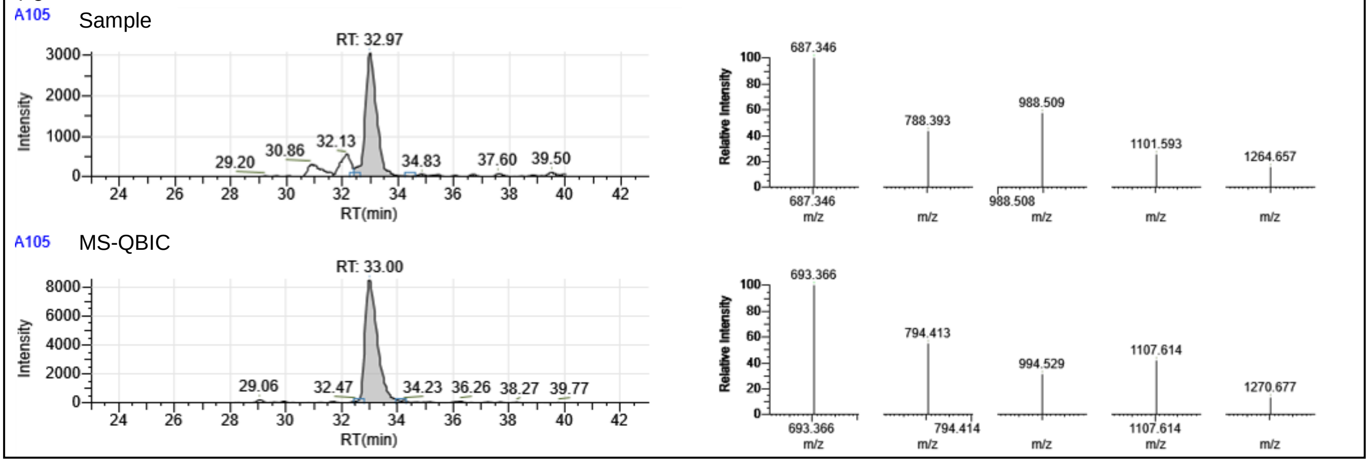

A105 Sample
RT: 32.97
29.20 30.86 32.13 34.83 37.60 39.50
Intensity
RT(min)
Relative Intensity
687.346 788.393 988.509 1101.593 1264.657
A105 MS-QBIC
RT: 33.00
29.06 32.47 34.23 36.26 38.27 39.77
693.366 794.413 994.529 1107.614 1270.677

P6

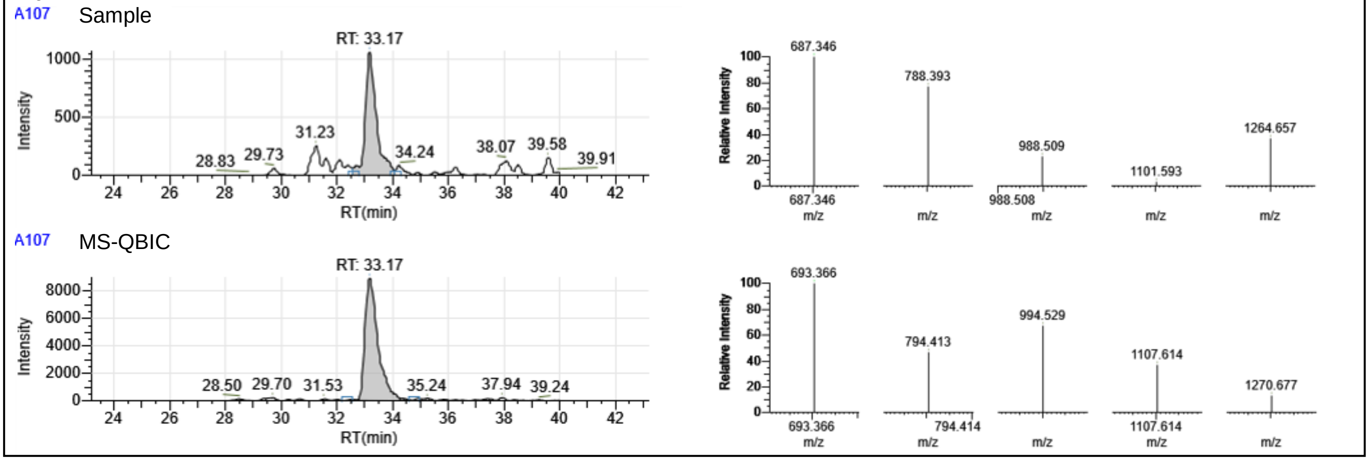

A107 Sample
RT: 33.17
28.83 29.73 31.23 34.24 38.07 39.58 39.91
Intensity
RT(min)
Relative Intensity
687.346 788.393 988.509 1101.593 1264.657
A107 MS-QBIC
RT: 33.17
28.50 29.70 31.53 35.24 37.94 39.24
693.366 794.413 994.529 1107.614 1270.677

P7

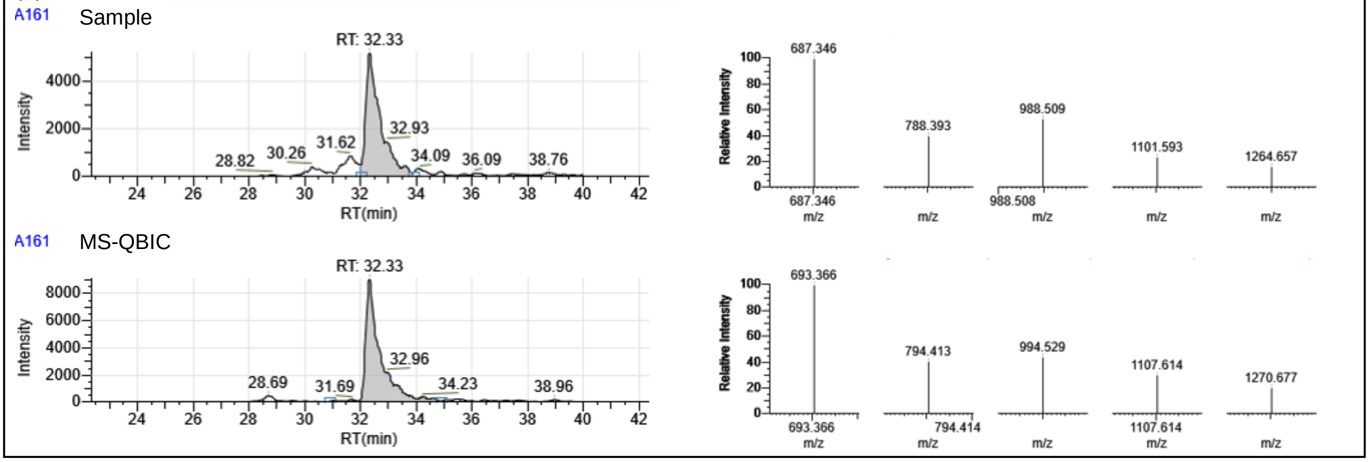

A161 Sample
RT: 32.33
28.82 30.26 31.62 32.93 34.09 36.09 38.76
Intensity
RT(min)
Relative Intensity
687.346 788.393 988.509 1101.593 1264.657
A161 MS-QBIC
RT: 32.33
28.69 31.69 32.96 34.23 38.96
693.366 794.413 994.529 1107.614 1270.677

P8

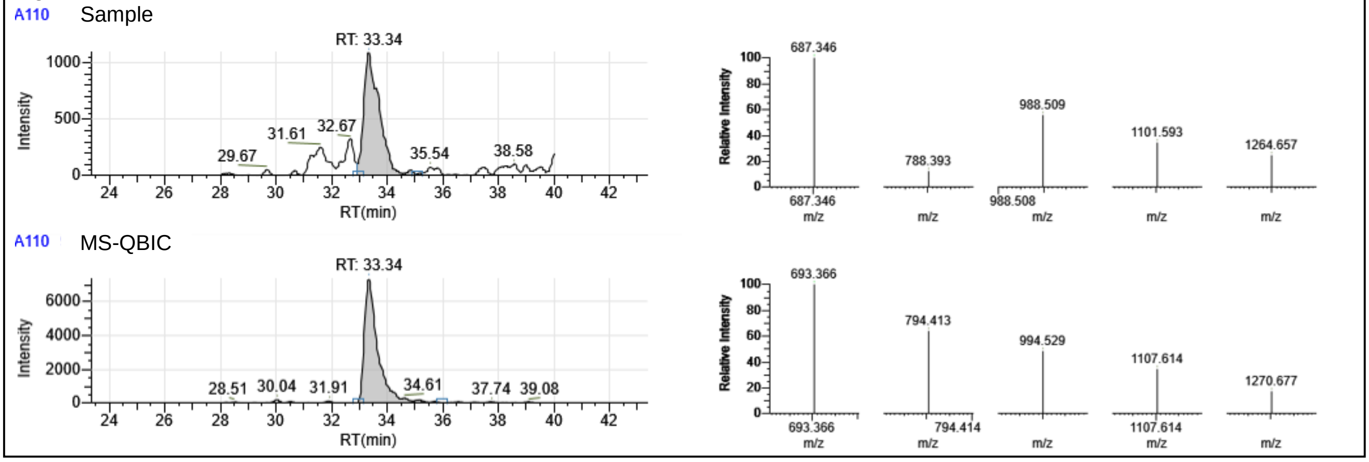

A110 Sample
RT: 33.34
29.67 31.61 32.67 35.54 38.58
Intensity
RT(min)
Relative Intensity
687.346 788.393 988.509 1101.593 1264.657
A110 MS-QBIC
RT: 33.34
28.51 30.04 31.91 34.61 37.74 39.08
693.366 794.413 994.529 1107.614 1270.677

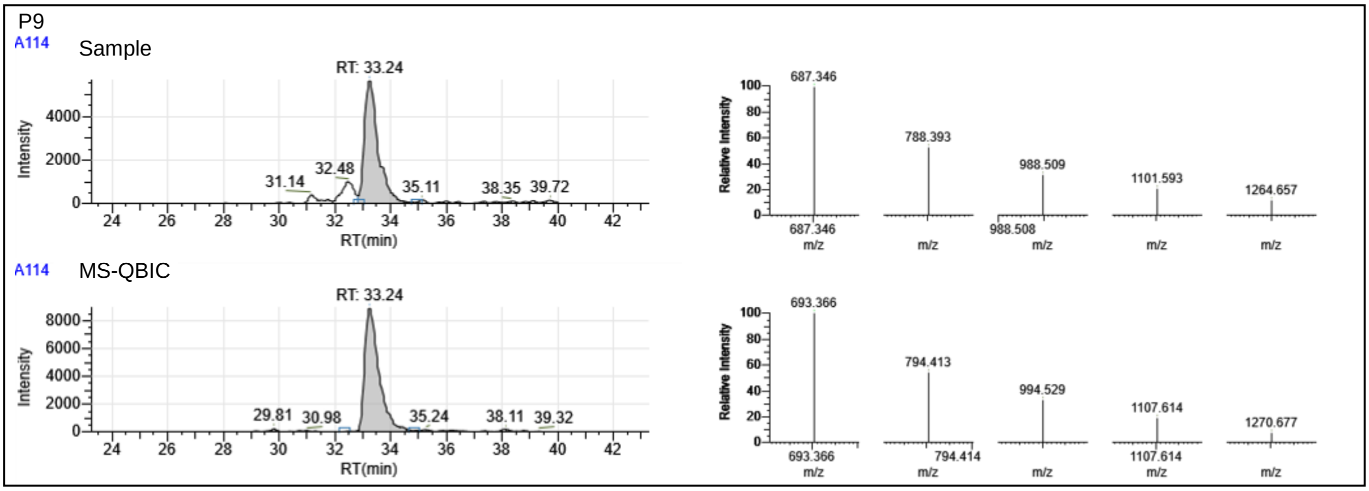
P9
A114 Sample
RT: 33.24
31.14 32.48 35.11 38.35 39.72
687.346 788.393 988.509 1101.593 1264.657
988.508
A114 MS-QBIC
RT: 33.24
29.81 30.98 35.24 38.11 39.32
693.366 794.413 994.529 1107.614 1270.677
794.414
Intensity
RT(min)
Relative Intensity
m/z

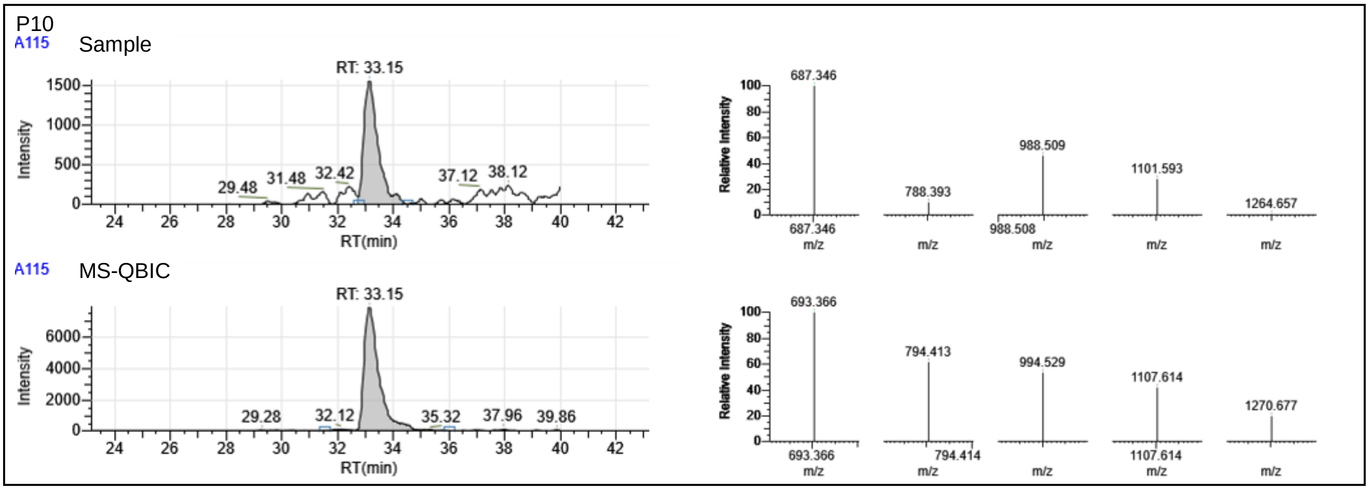
P10
A115 Sample
RT: 33.15
29.48 31.48 32.42 37.12 38.12
687.346 788.393 988.509 1101.593 1264.657
988.508
A115 MS-QBIC
RT: 33.15
29.28 32.12 35.32 37.96 39.86
693.366 794.413 994.529 1107.614 1270.677
794.414
Intensity
RT(min)
Relative Intensity
m/z

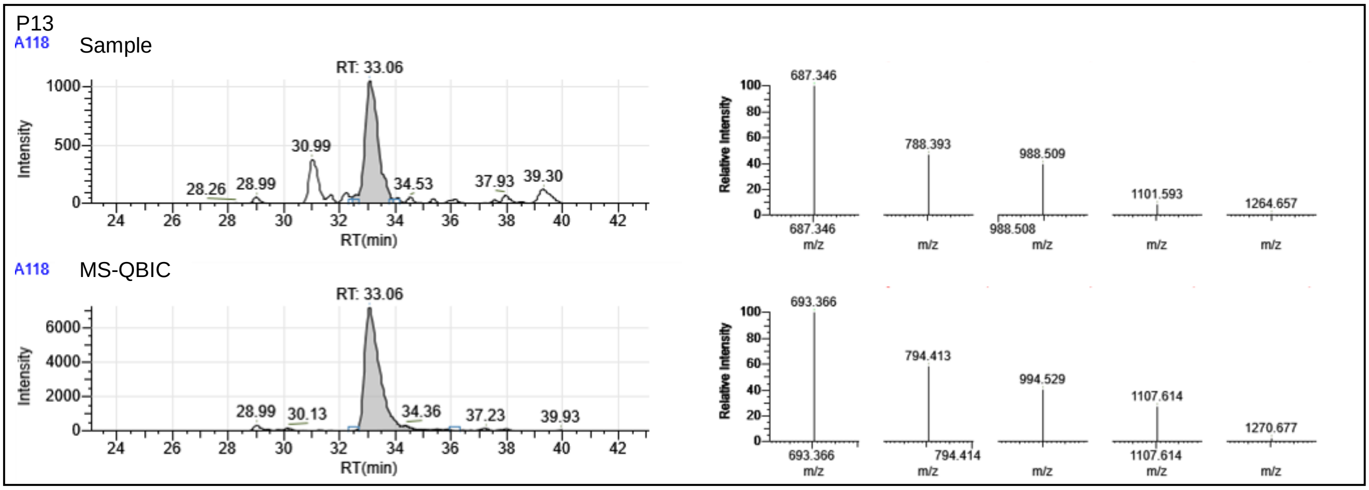
P13
A118 Sample
RT: 33.06
28.26 28.99 30.99 34.53 37.93 39.30
687.346 788.393 988.509 1101.593 1264.657
988.508
A118 MS-QBIC
RT: 33.06
28.99 30.13 34.36 37.23 39.93
693.366 794.413 994.529 1107.614 1270.677
794.414
Intensity
RT(min)
Relative Intensity
m/z

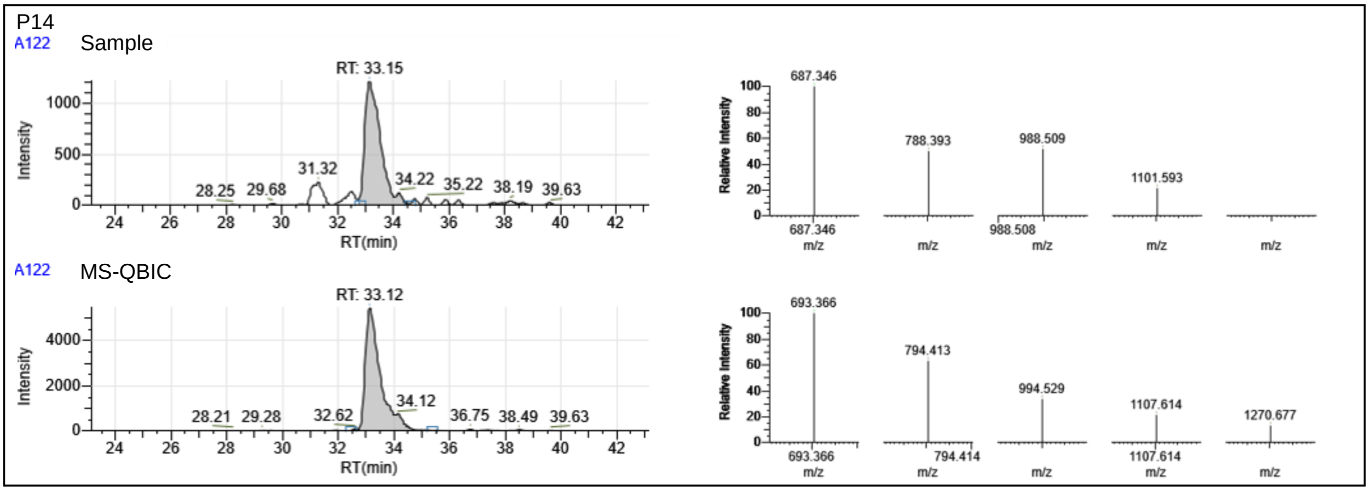
P14
A122 Sample
RT: 33.15
28.25 29.68 31.32 34.22 35.22 38.19 39.63
687.346 788.393 988.509 1101.593
988.508
A122 MS-QBIC
RT: 33.12
28.21 29.28 32.62 34.12 36.75 38.49 39.63
693.366 794.413 994.529 1107.614 1270.677
794.414
Intensity
RT(min)
Relative Intensity
m/z

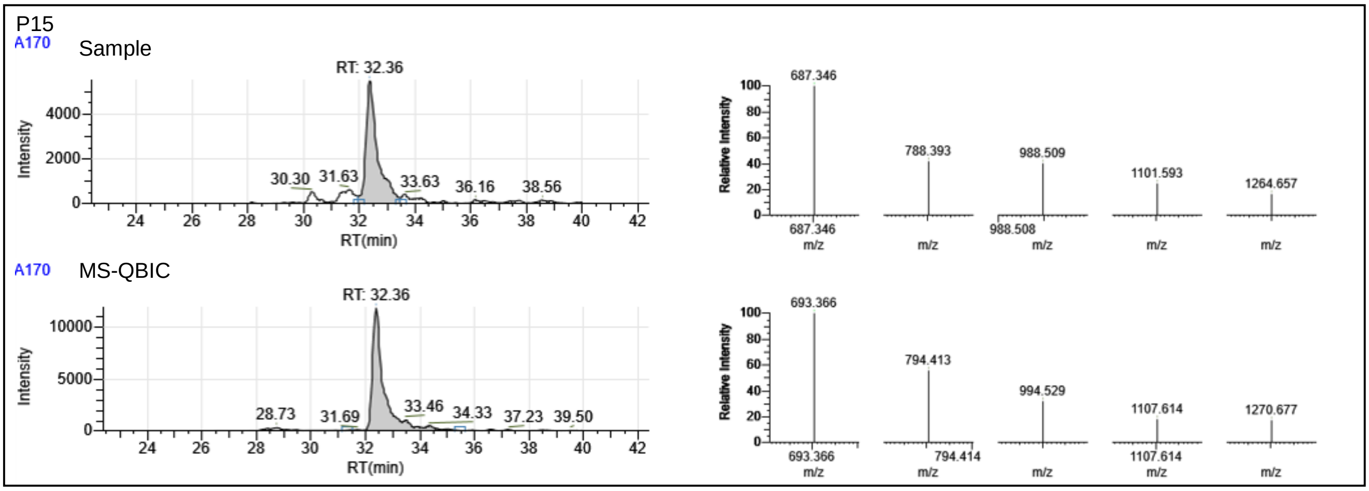
P15
A170 Sample
RT: 32.36
30.30 31.63 33.63 36.16 38.56
Intensity
RT(min)
687.346
788.393
988.509
1101.593
1264.657
988.508
Relative Intensity
m/z
A170 MS-QBIC
RT: 32.36
28.73 31.69 33.46 34.33 37.23 39.50
693.366
794.413
994.529
1107.614
1270.677
794.414

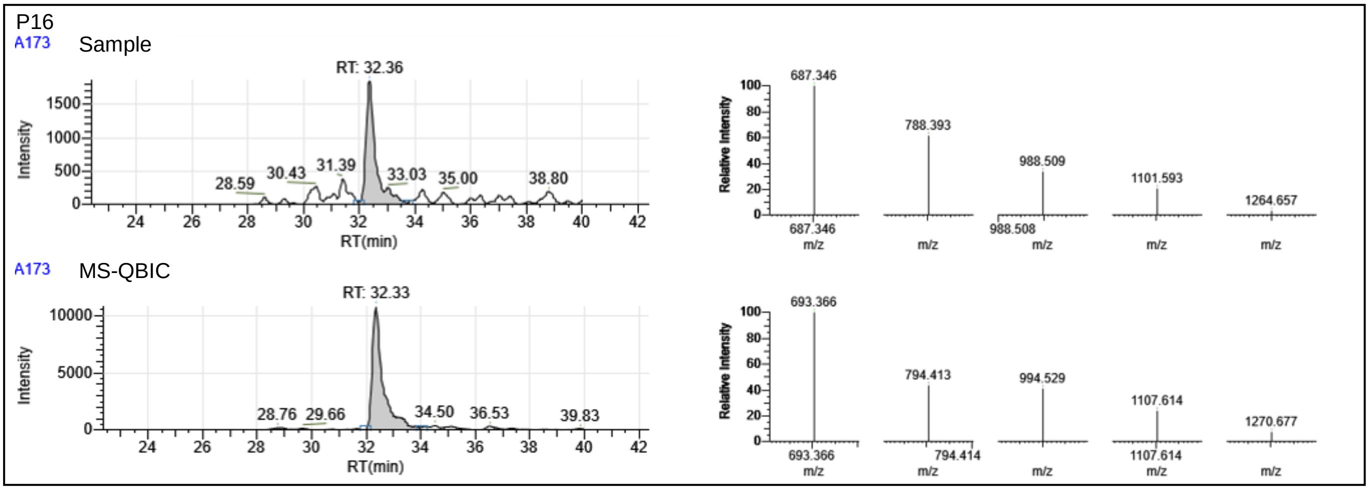
P16
A173 Sample
RT: 32.36
28.59 30.43 31.39 33.03 35.00 38.80
Intensity
RT(min)
687.346
788.393
988.509
1101.593
1264.657
988.508
Relative Intensity
m/z
A173 MS-QBIC
RT: 32.33
28.76 29.66 34.50 36.53 39.83
693.366
794.413
994.529
1107.614
1270.677
794.414

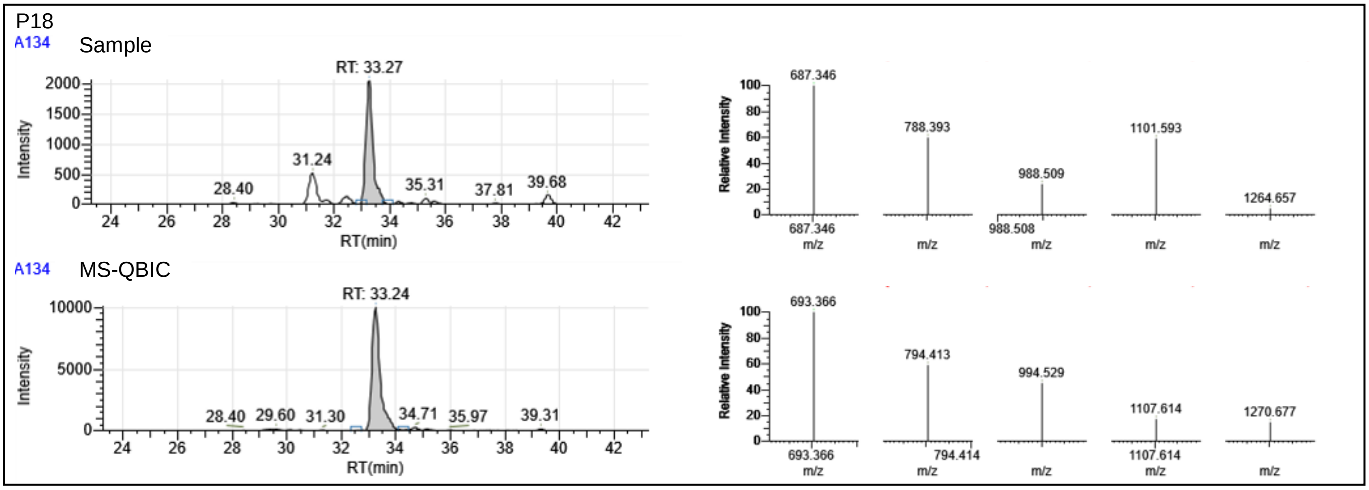
P18
A134 Sample
RT: 33.27
28.40 31.24 35.31 37.81 39.68
Intensity
RT(min)
687.346
788.393
988.509
1101.593
1264.657
988.508
Relative Intensity
m/z
A134 MS-QBIC
RT: 33.24
28.40 29.60 31.30 34.71 35.97 39.31
693.366
794.413
994.529
1107.614
1270.677
794.414

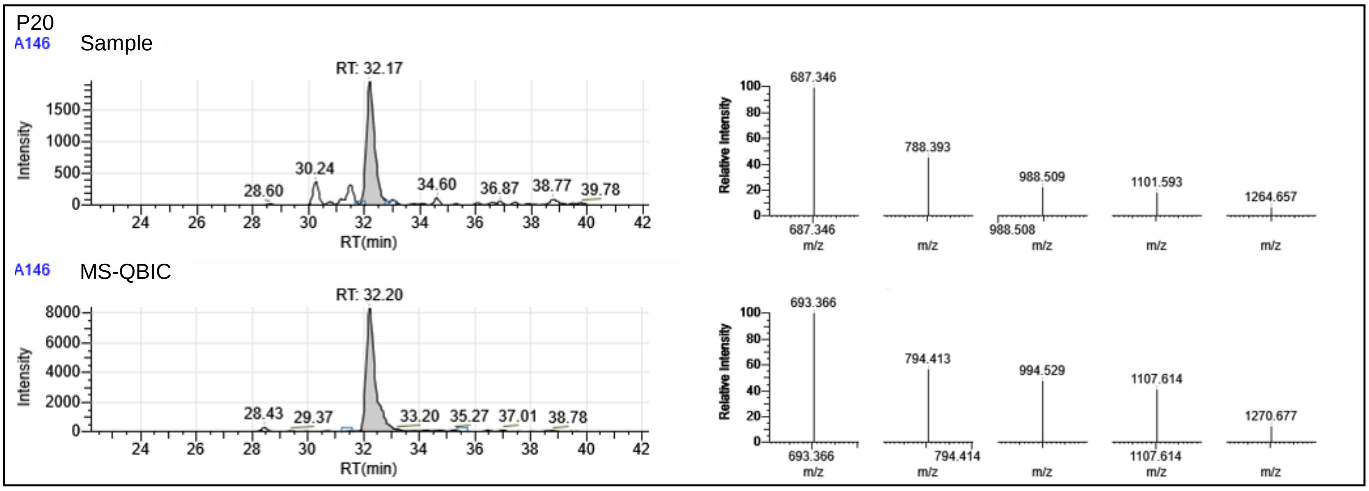
P20
A146 Sample
RT: 32.17
28.60 30.24 34.60 36.87 38.77 39.78
Intensity
RT(min)
687.346
788.393
988.509
1101.593
1264.657
988.508
Relative Intensity
m/z
A146 MS-QBIC
RT: 32.20
28.43 29.37 33.20 35.27 37.01 38.78
693.366
794.413
994.529
1107.614
1270.677
794.414

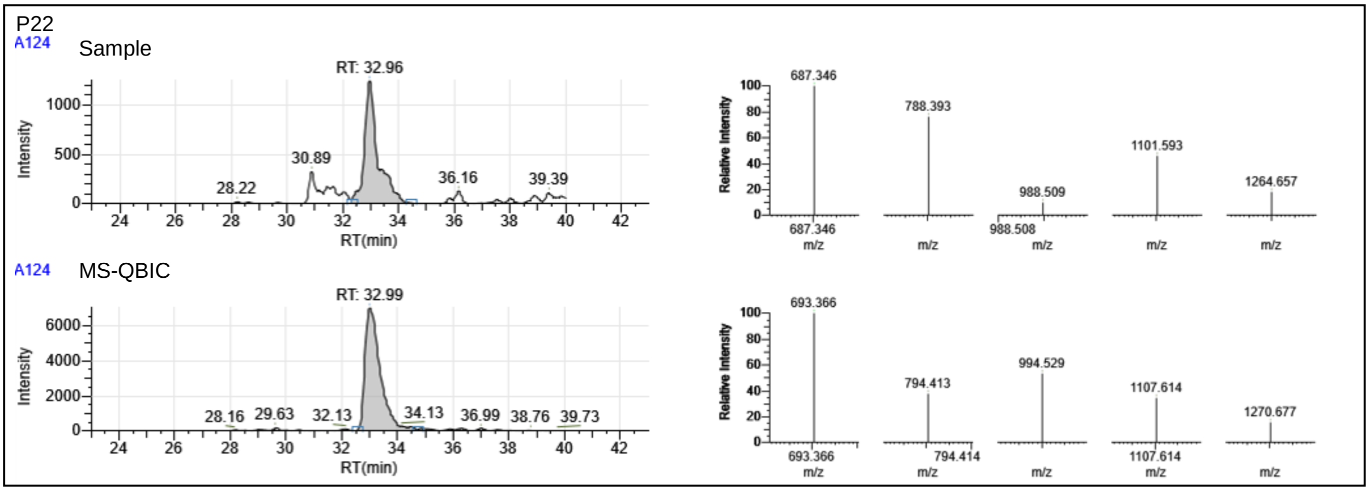
P22
A124 Sample
RT: 32.96
30.89
28.22
36.16
39.39
Intensity
RT(min)
687.346
788.393
988.509
1101.593
1264.657
988.508
Relative Intensity
m/z
A124 MS-QBIC
RT: 32.99
28.16 29.63 32.13 34.13 36.99 38.76 39.73
693.366
794.413
994.529
1107.614
1270.677
794.414

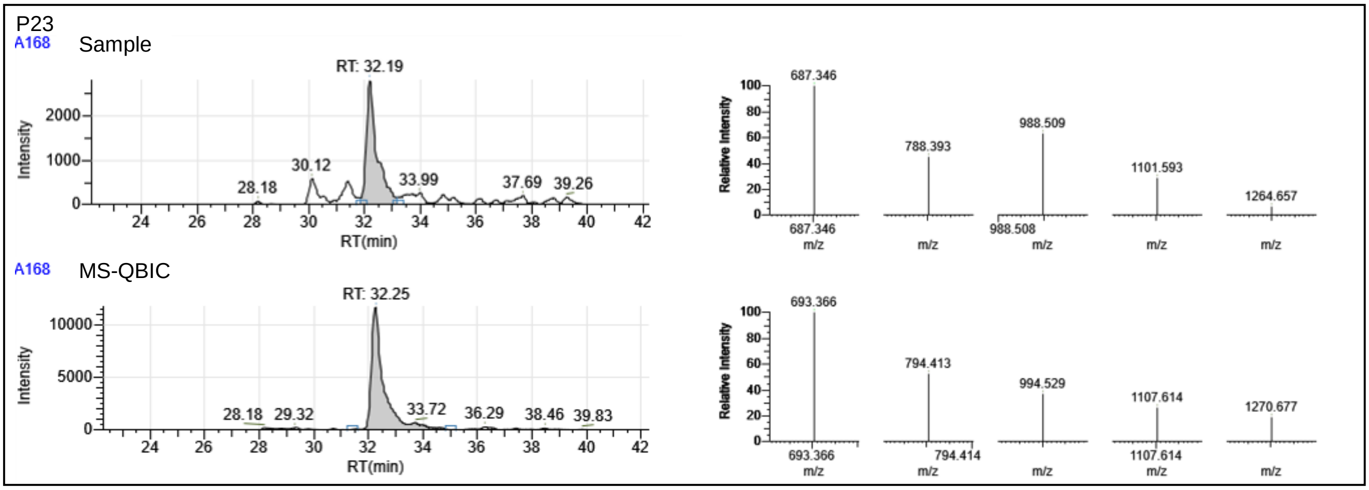
P23
A168 Sample
RT: 32.19
30.12
28.18
33.99
37.69 39.26
Intensity
RT(min)
687.346
788.393
988.509
1101.593
1264.657
988.508
Relative Intensity
m/z
A168 MS-QBIC
RT: 32.25
28.18 29.32 33.72 36.29 38.46 39.83
693.366
794.413
994.529
1107.614
1270.677
794.414

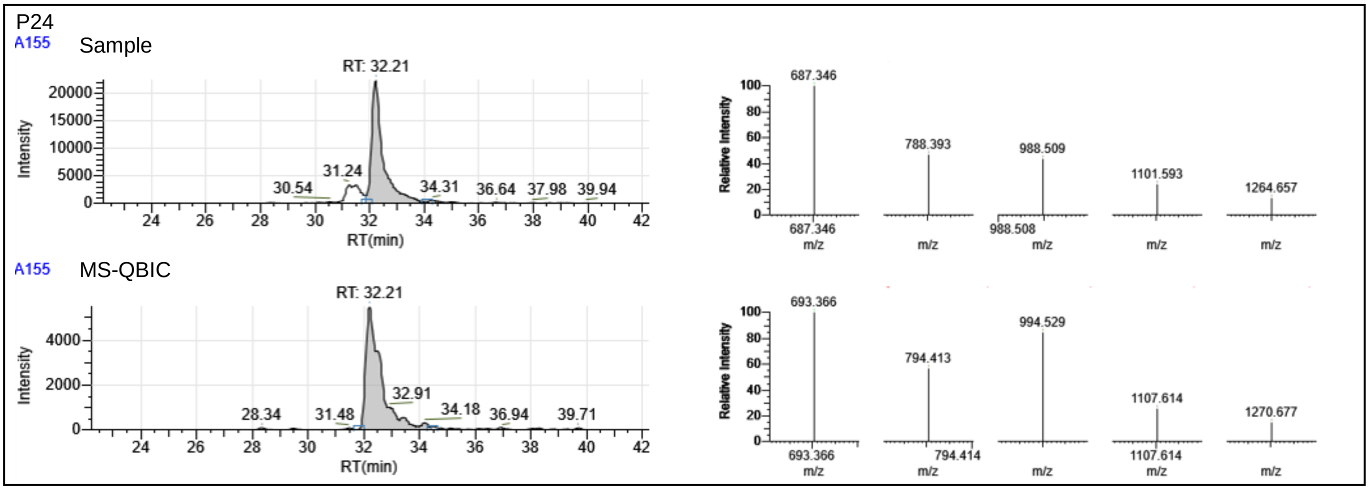
P24
A155 Sample
RT: 32.21
31.24
30.54
34.31 36.64 37.98 39.94
Intensity
RT(min)
687.346
788.393
988.509
1101.593
1264.657
988.508
Relative Intensity
m/z
A155 MS-QBIC
RT: 32.21
32.91
28.34 31.48 34.18 36.94 39.71
693.366
794.413
994.529
1107.614
1270.677
794.414

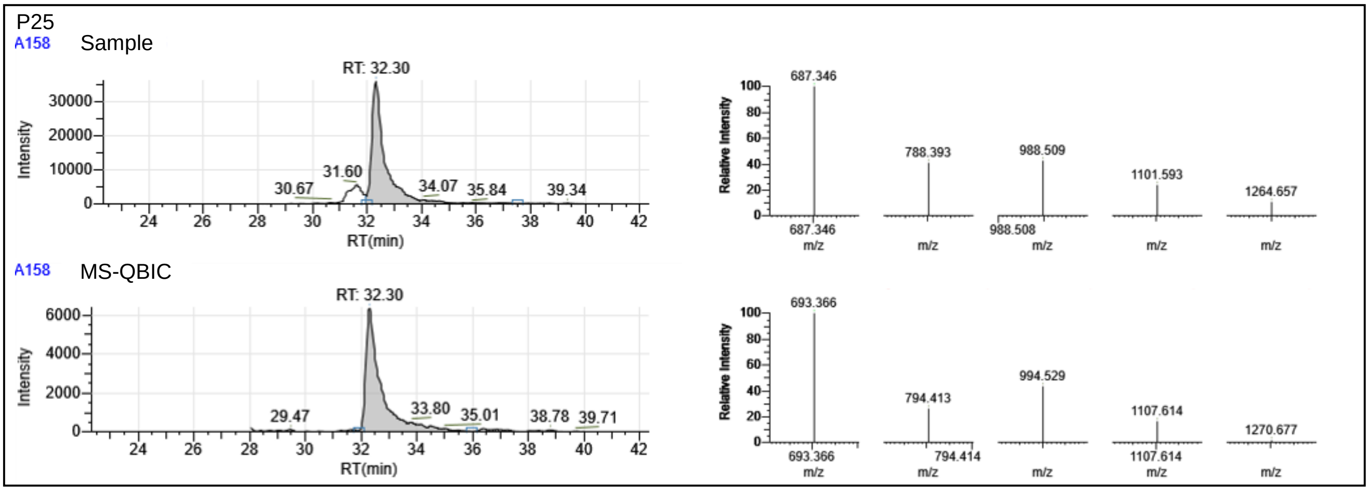
P25
A158 Sample
RT: 32.30
31.60
30.67
34.07 35.84 39.34
Intensity
RT(min)
687.346
788.393
988.509
1101.593
1264.657
988.508
Relative Intensity
m/z
A158 MS-QBIC
RT: 32.30
29.47 33.80 35.01 38.78 39.71
693.366
794.413
994.529
1107.614
1270.677
794.414

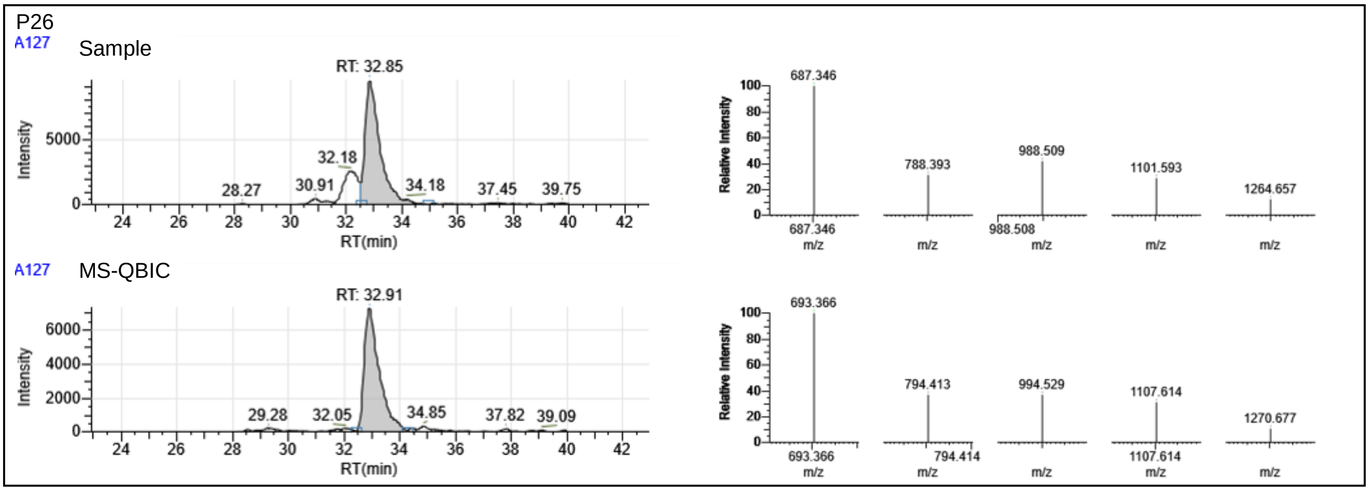
P26
A127 Sample
RT: 32.85
32.18
28.27 30.91 34.18 37.45 39.75
Intensity
RT(min)
687.346
788.393
988.509
1101.593
1264.657
988.508
Relative Intensity
m/z
A127 MS-QBIC
RT: 32.91
29.28 32.05 34.85 37.82 39.09
693.366
794.413
994.529
1107.614
1270.677
794.414

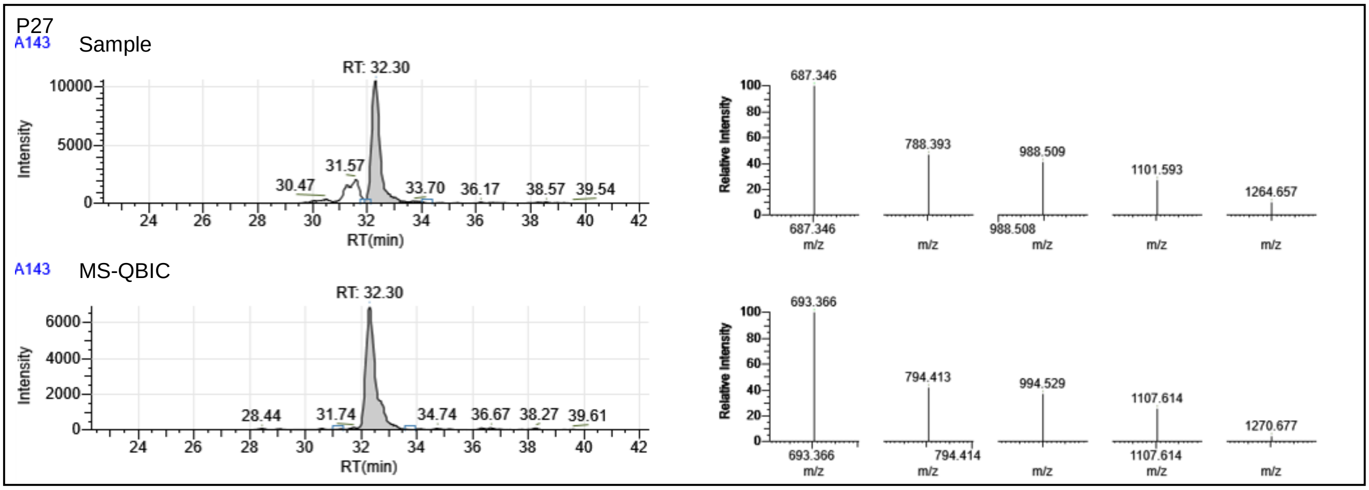
P27
A143 Sample
RT: 32.30
31.57
30.47 33.70 36.17 38.57 39.54
Intensity
RT(min)
687.346
788.393
988.509
1101.593
1264.657
988.508
Relative Intensity
m/z
A143 MS-QBIC
RT: 32.30
28.44 31.74 34.74 36.67 38.27 39.61
693.366
794.413
994.529
1107.614
1270.677
794.414

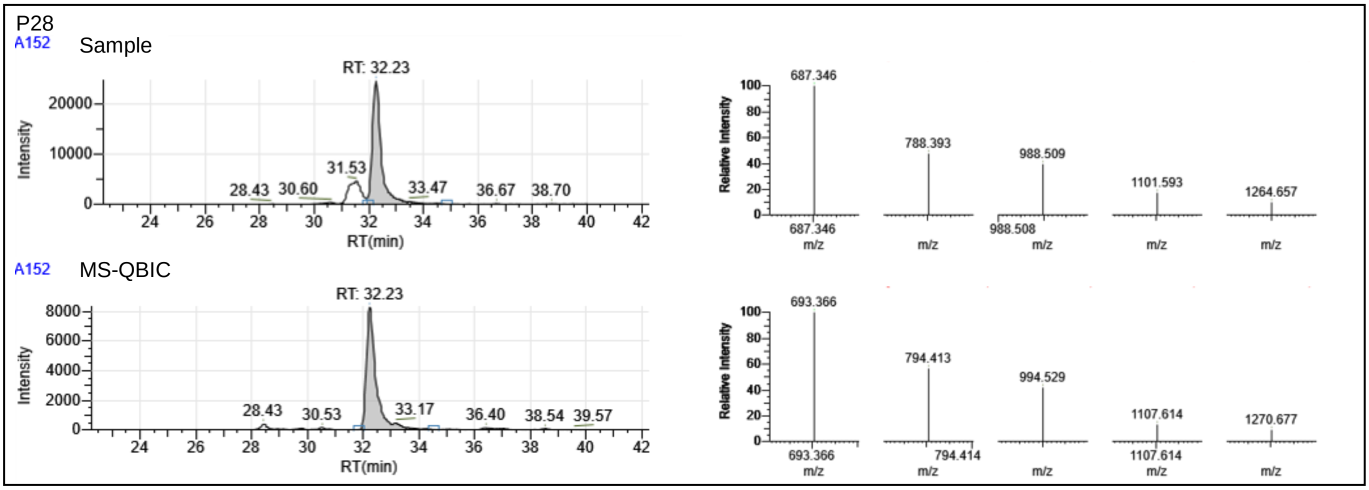
P28
A152 Sample
RT: 32.23
31.53
28.43 30.60 33.47 36.67 38.70
Intensity
RT(min)
687.346
788.393
988.509
1101.593
1264.657
988.508
Relative Intensity
m/z
A152 MS-QBIC
RT: 32.23
28.43 30.53 33.17 36.40 38.54 39.57
693.366
794.413
994.529
1107.614
1270.677
794.414

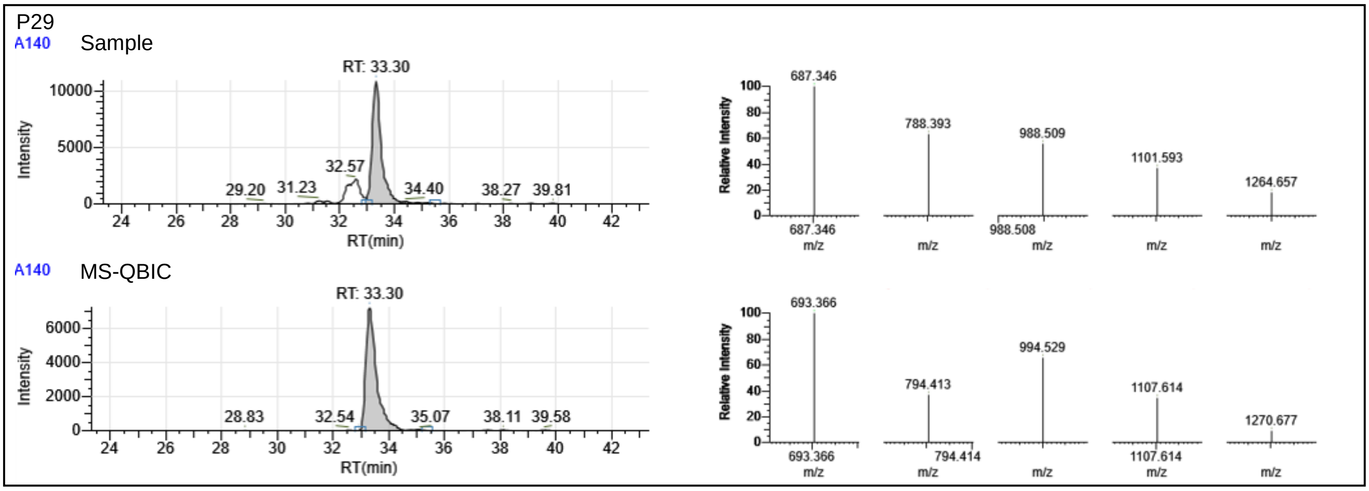
P29
A140 Sample
RT: 33.30
32.57
29.20 31.23 34.40 38.27 39.81
Intensity
RT(min)
687.346
788.393
988.509
1101.593
1264.657
988.508
Relative Intensity
m/z
A140 MS-QBIC
RT: 33.30
28.83 32.54 35.07 38.11 39.58
693.366
794.413
994.529
1107.614
1270.677
794.414

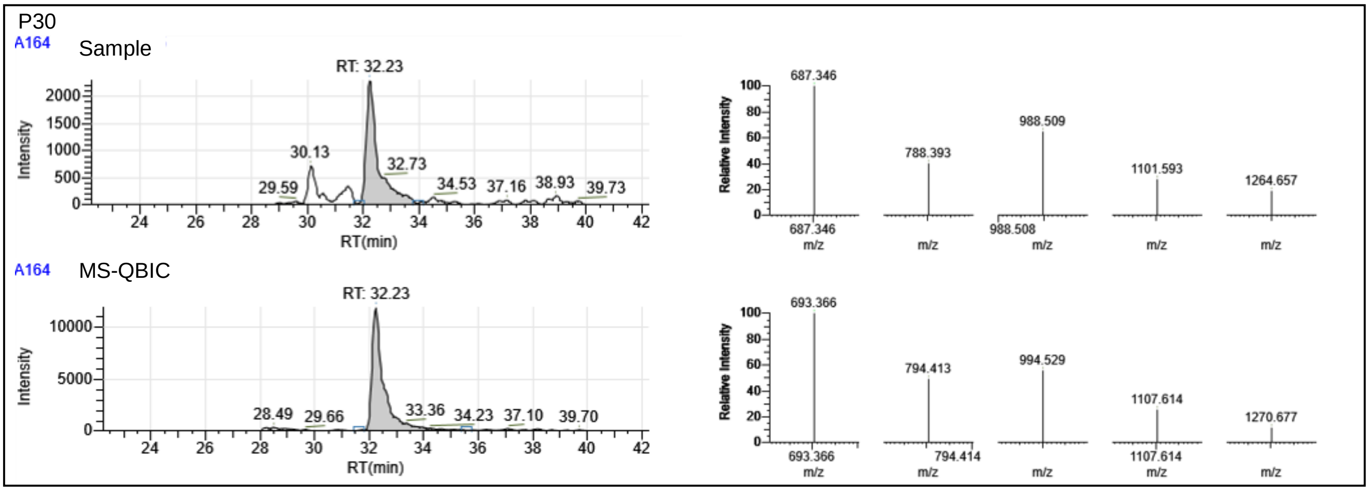

P30
A164
Sample
RT: 32.23
30.13
29.59
32.73
34.53
37.16
38.93
39.73
Intensity
RT(min)
687.346
788.393
988.509
1101.593
1264.657
988.508
Relative Intensity
m/z
A164
MS-QBIC
RT: 32.23
28.49
29.66
33.36
34.23
37.10
39.70
693.366
794.413
994.529
1107.614
1270.677
794.414

Q B2M (IQVYSR) Another organ

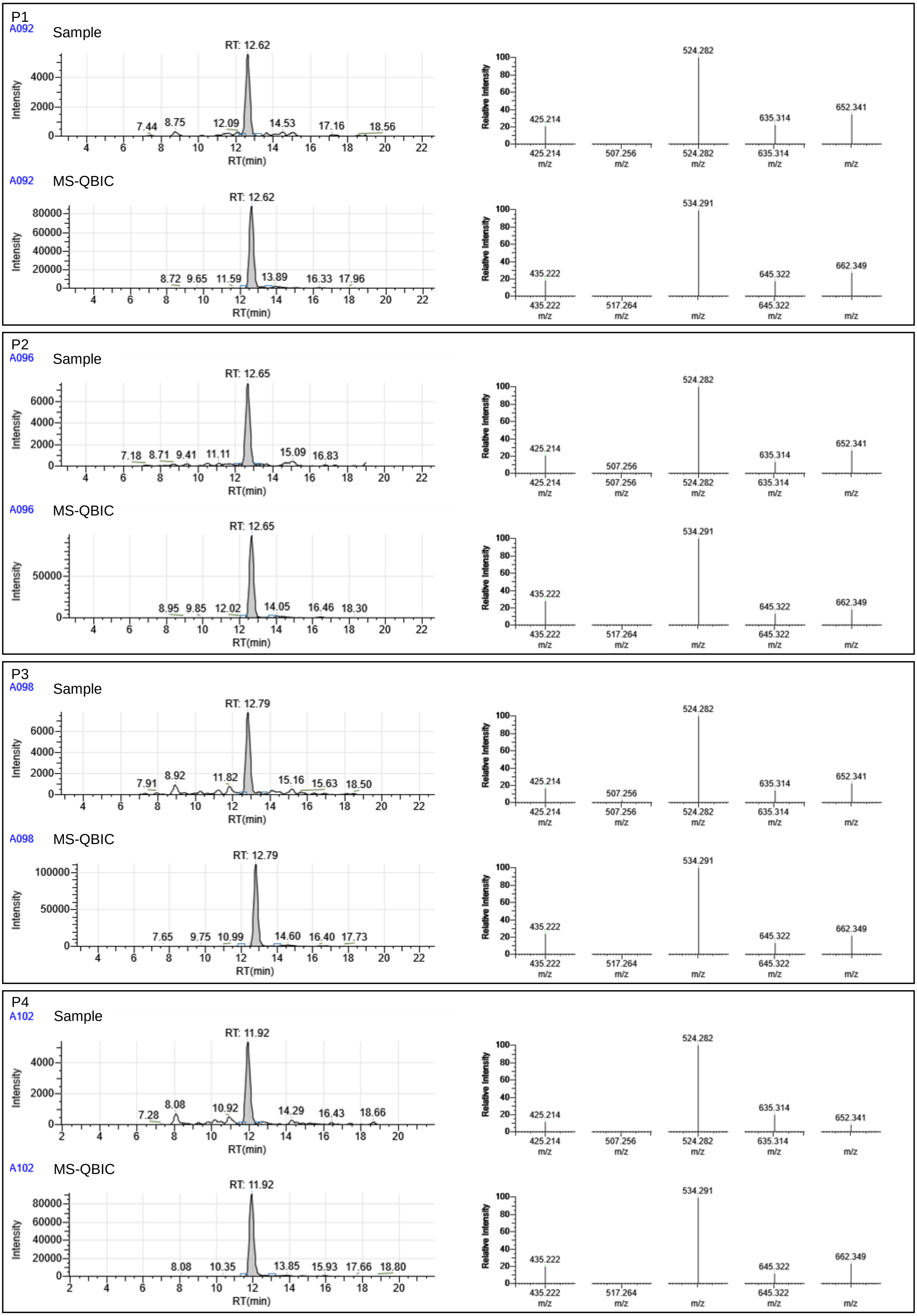

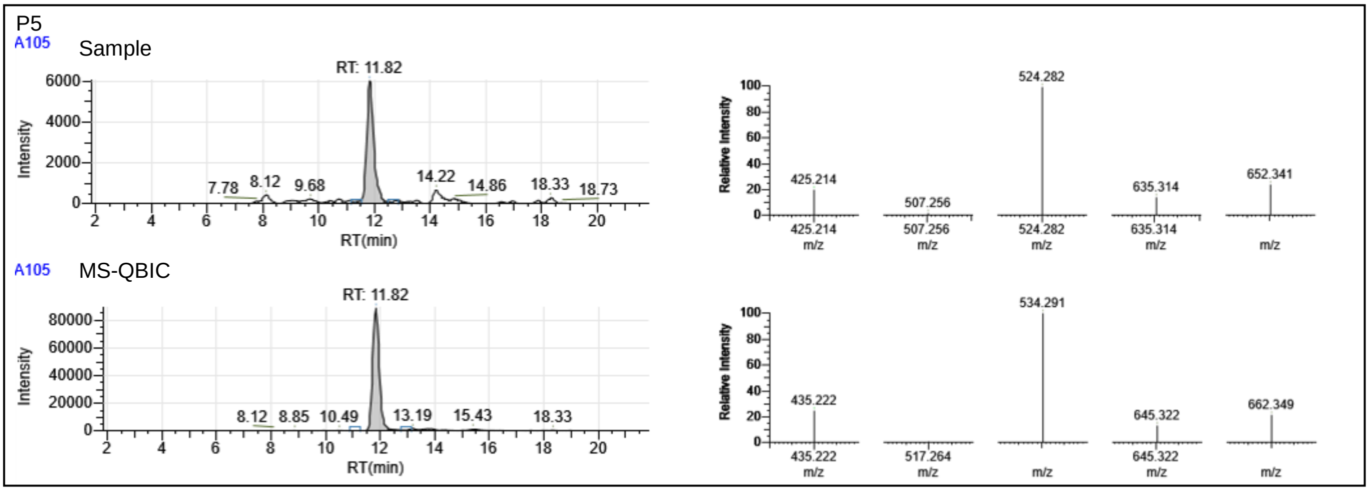
P5
A105 Sample
RT: 11.82
Intensity
7.78 8.12 9.68 14.22 14.86 18.33 18.73
RT(min)
Relative Intensity
425.214 507.256 524.282 635.314 652.341
m/z
A105 MS-QBIC
RT: 11.82
8.12 8.85 10.49 13.19 15.43 18.33
435.222 517.264 534.291 645.322 662.349

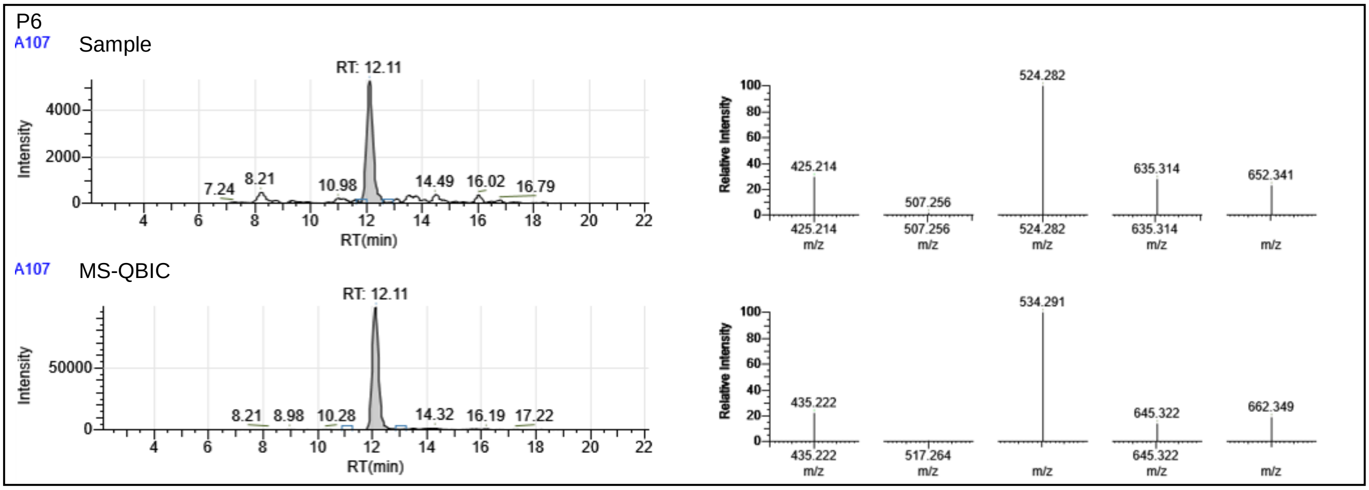
P6
A107 Sample
RT: 12.11
Intensity
7.24 8.21 10.98 14.49 16.02 16.79
RT(min)
Relative Intensity
425.214 507.256 524.282 635.314 652.341
m/z
A107 MS-QBIC
RT: 12.11
8.21 8.98 10.28 14.32 16.19 17.22
435.222 517.264 534.291 645.322 662.349

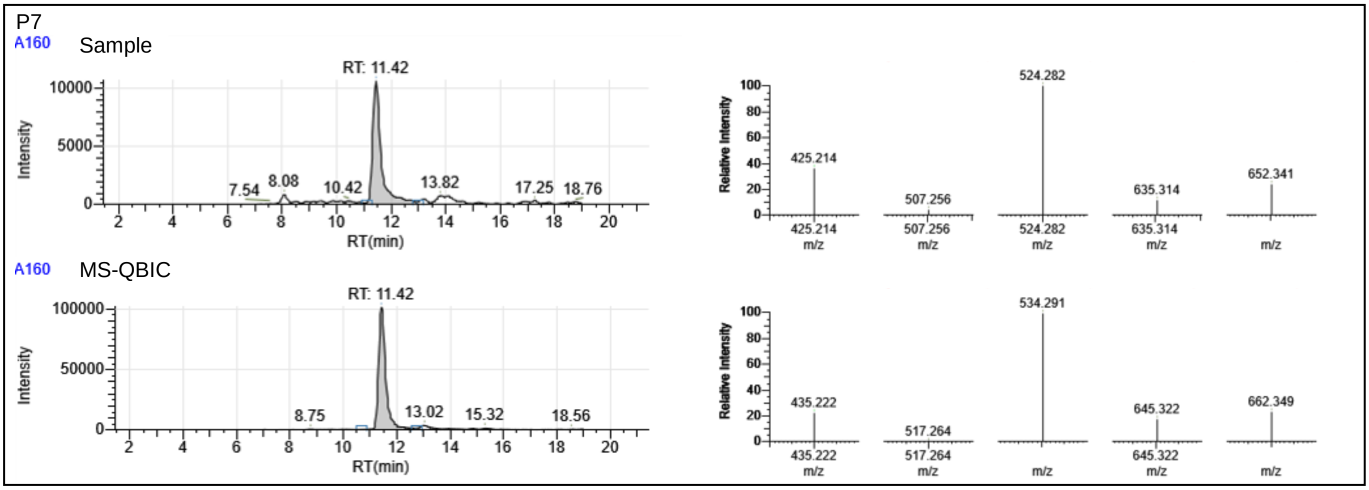
P7
A160 Sample
RT: 11.42
Intensity
7.54 8.08 10.42 13.82 17.25 18.76
RT(min)
Relative Intensity
425.214 507.256 524.282 635.314 652.341
m/z
A160 MS-QBIC
RT: 11.42
8.75 13.02 15.32 18.56
435.222 517.264 534.291 645.322 662.349

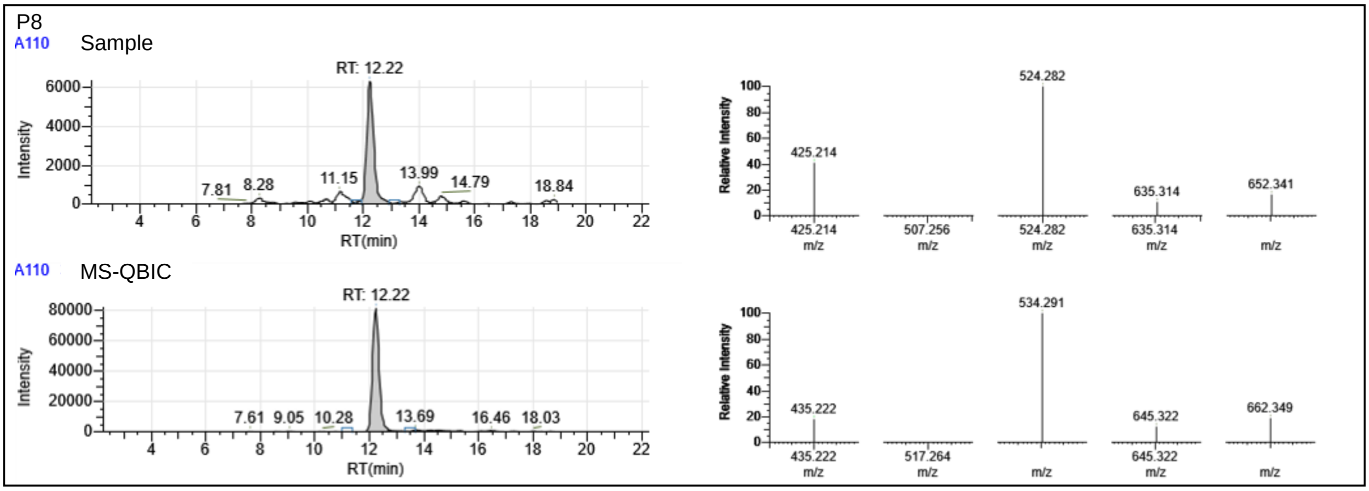
P8
A110 Sample
RT: 12.22
Intensity
7.81 8.28 11.15 13.99 14.79 18.84
RT(min)
Relative Intensity
425.214 507.256 524.282 635.314 652.341
m/z
A110 MS-QBIC
RT: 12.22
7.61 9.05 10.28 13.69 16.46 18.03
435.222 517.264 534.291 645.322 662.349

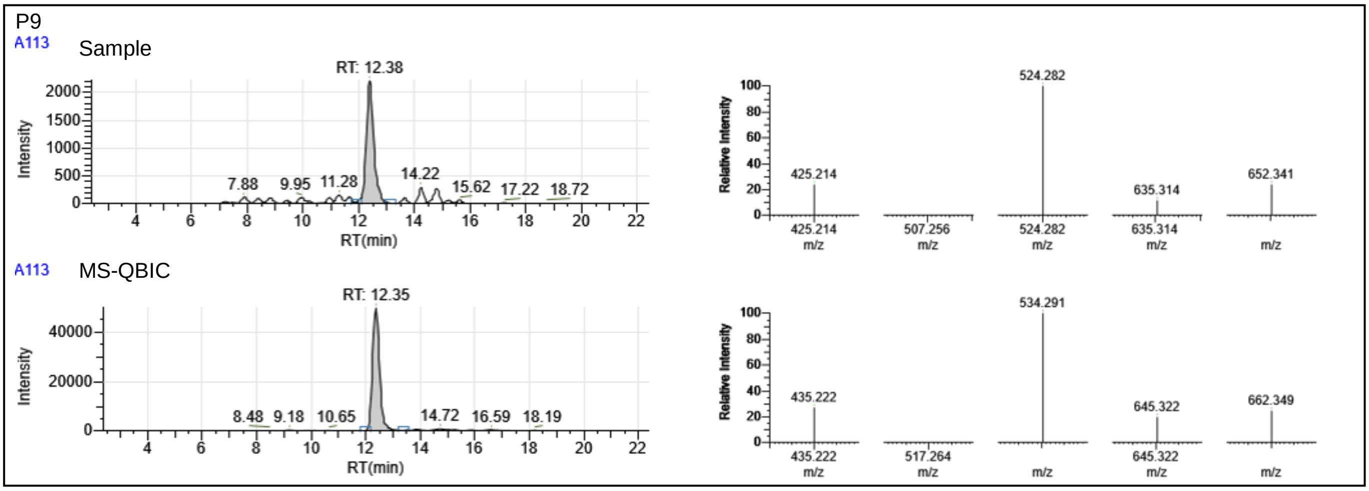
P9
A113 Sample
RT: 12.38
7.88 9.95 11.28 14.22 15.62 17.22 18.72
Intensity
RT(min)
Relative Intensity
425.214
524.282
635.314
652.341
507.256
m/z
A113 MS-QBIC
RT: 12.35
8.48 9.18 10.65 14.72 16.59 18.19
534.291
435.222
645.322
662.349
517.264

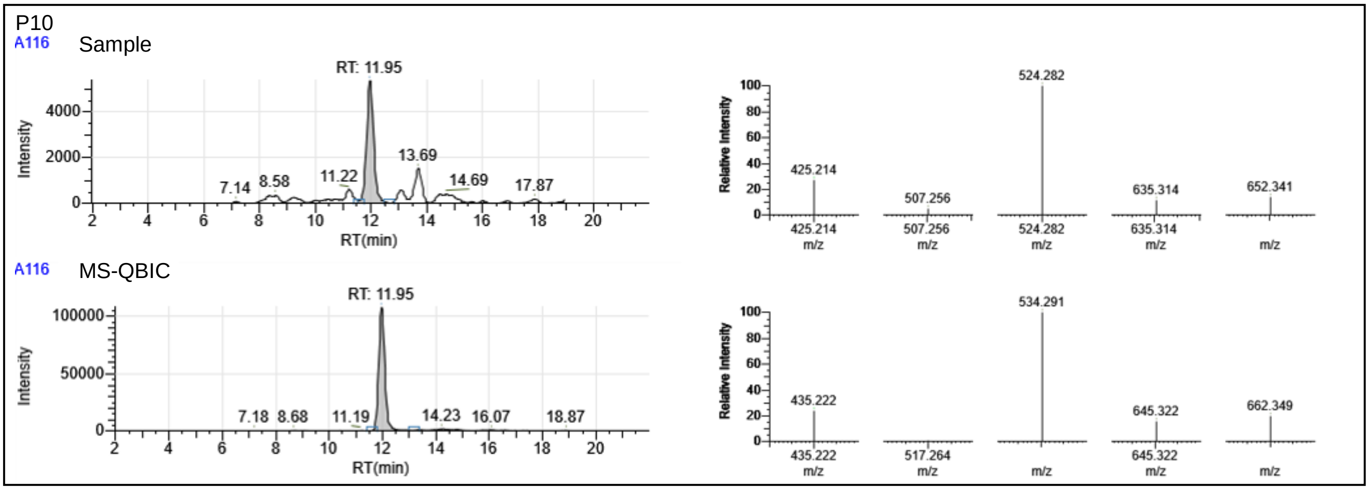
P10
A116 Sample
RT: 11.95
7.14 8.58 11.22 13.69 14.69 17.87
Intensity
RT(min)
Relative Intensity
425.214
507.256
524.282
635.314
652.341
m/z
A116 MS-QBIC
RT: 11.95
7.18 8.68 11.19 14.23 16.07 18.87
534.291
435.222
645.322
662.349
517.264

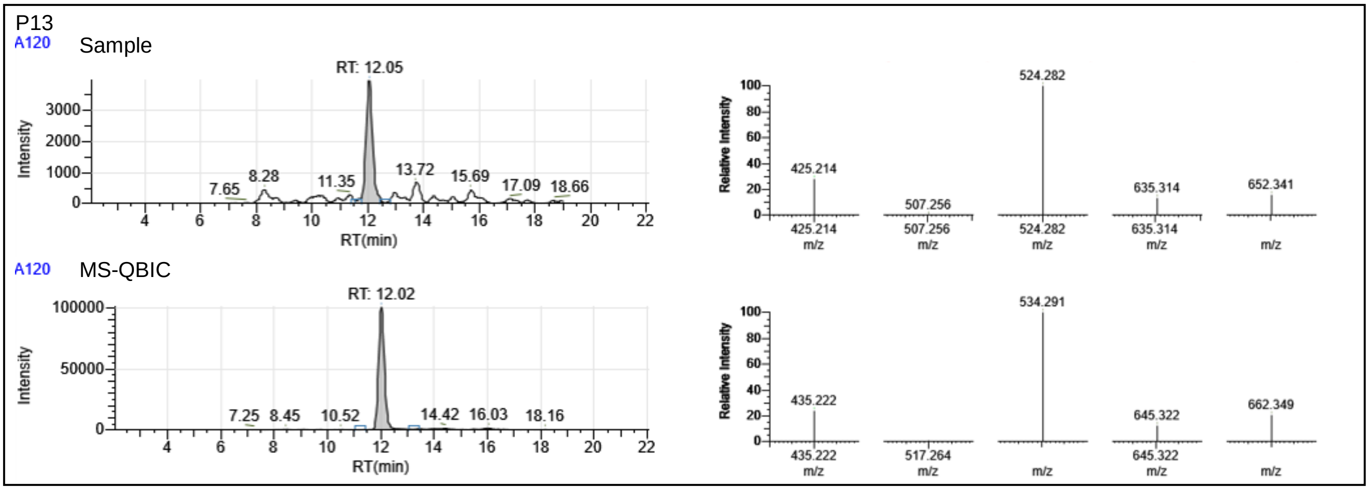
P13
A120 Sample
RT: 12.05
7.65 8.28 11.35 13.72 15.69 17.09 18.66
Intensity
RT(min)
Relative Intensity
425.214
507.256
524.282
635.314
652.341
m/z
A120 MS-QBIC
RT: 12.02
7.25 8.45 10.52 14.42 16.03 18.16
534.291
435.222
645.322
662.349
517.264

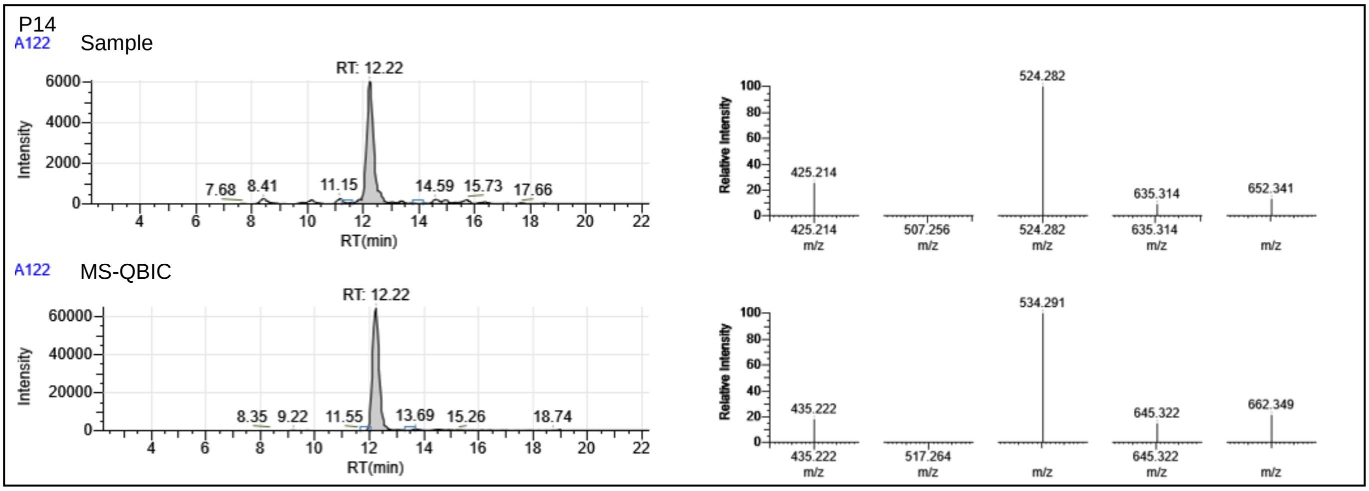
P14
A122 Sample
RT: 12.22
7.68 8.41 11.15 14.59 15.73 17.66
Intensity
RT(min)
Relative Intensity
425.214
524.282
635.314
652.341
507.256
m/z
A122 MS-QBIC
RT: 12.22
8.35 9.22 11.55 13.69 15.26 18.74
534.291
435.222
645.322
662.349
517.264

P15

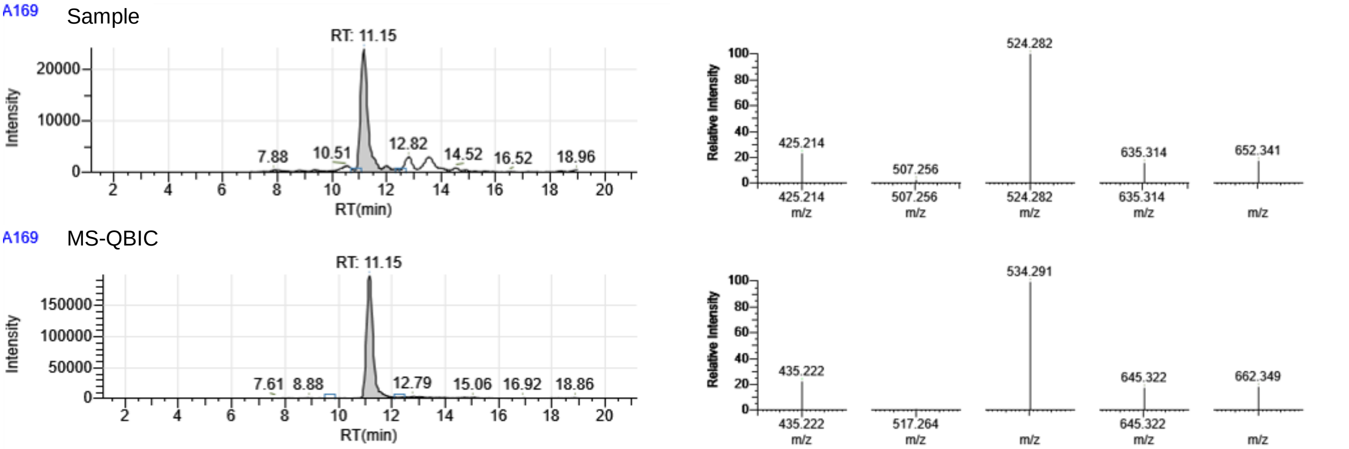

P16

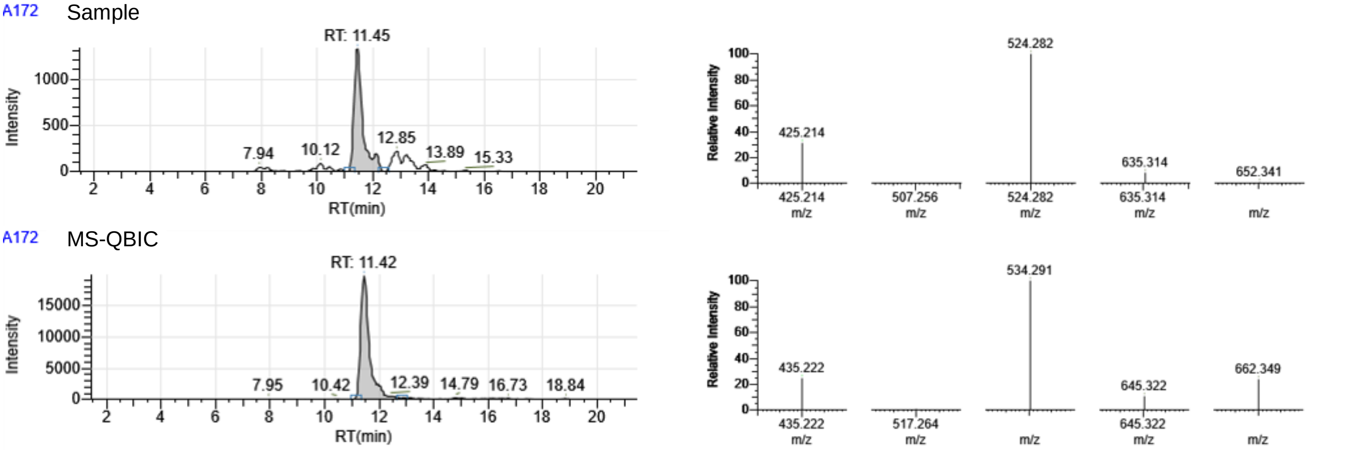

P17

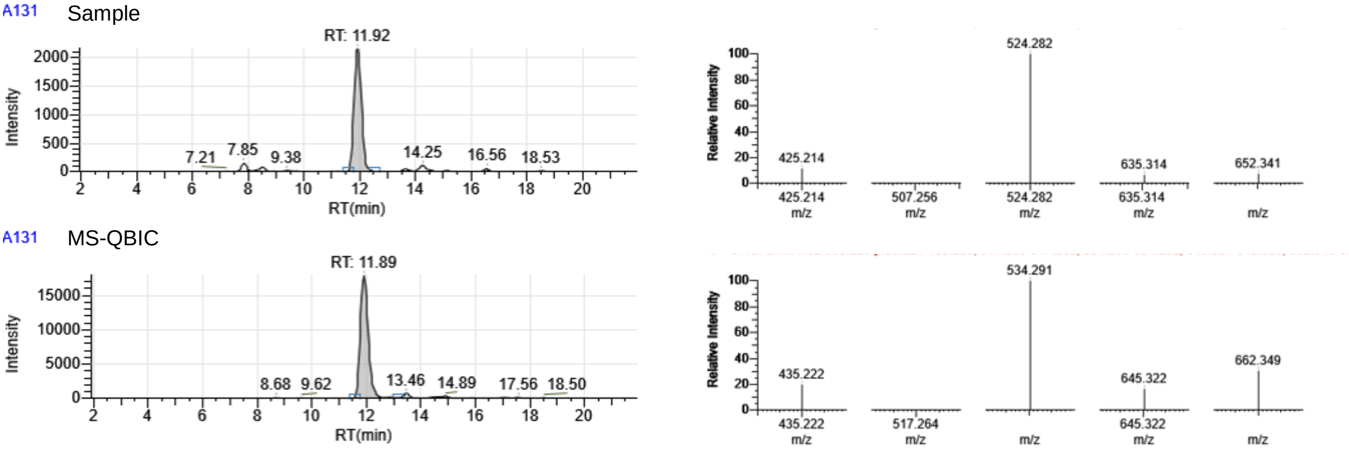

P18

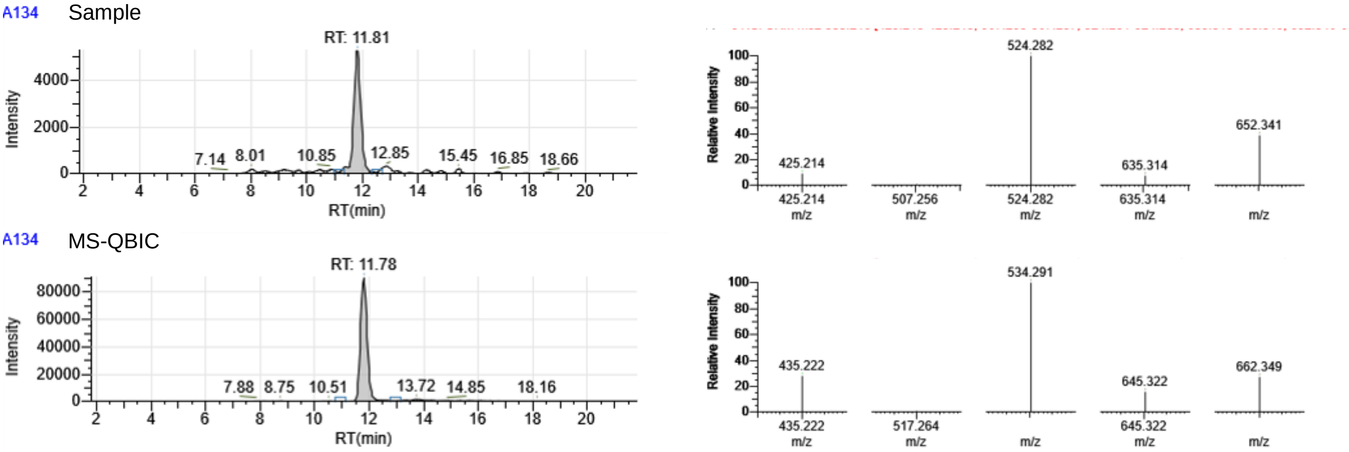

P19

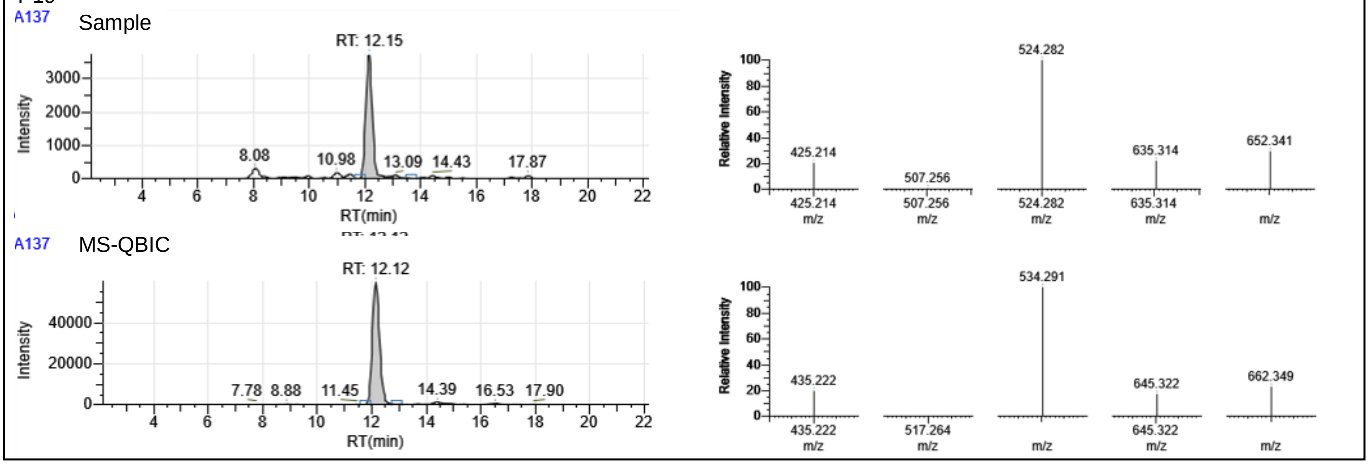

P20

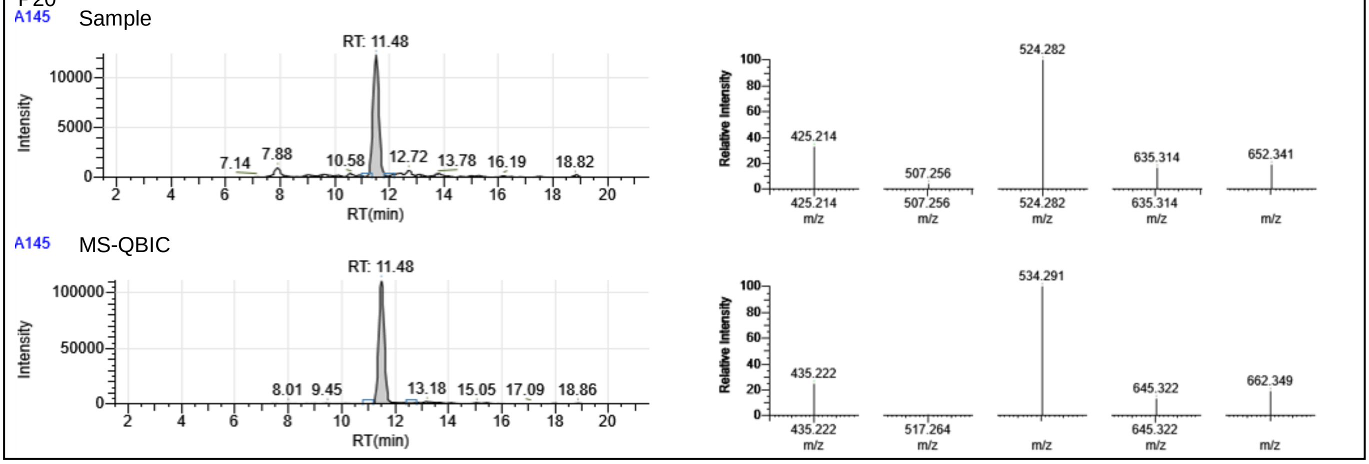

P21

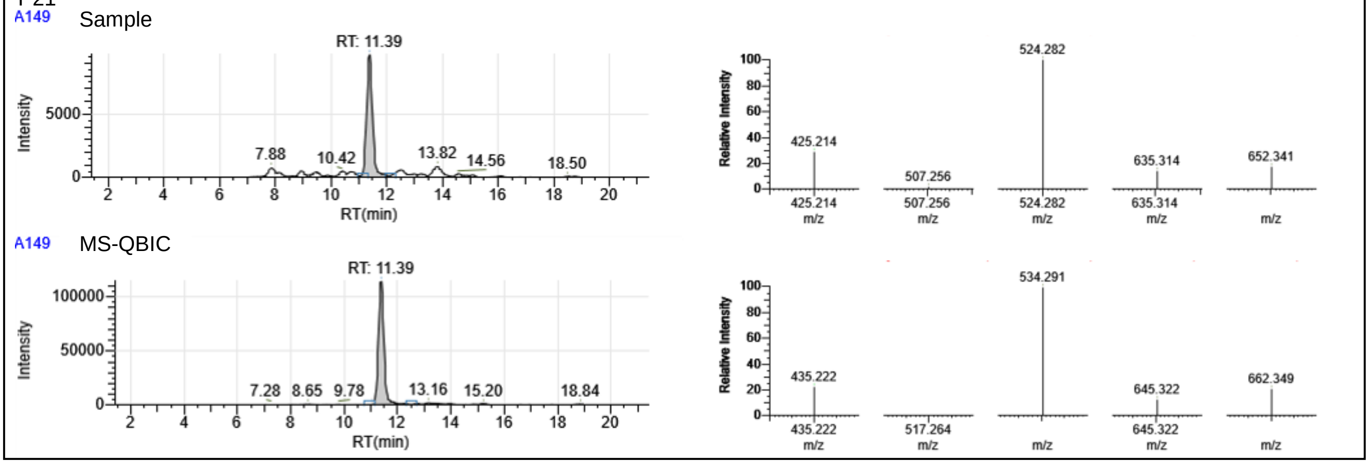

P22

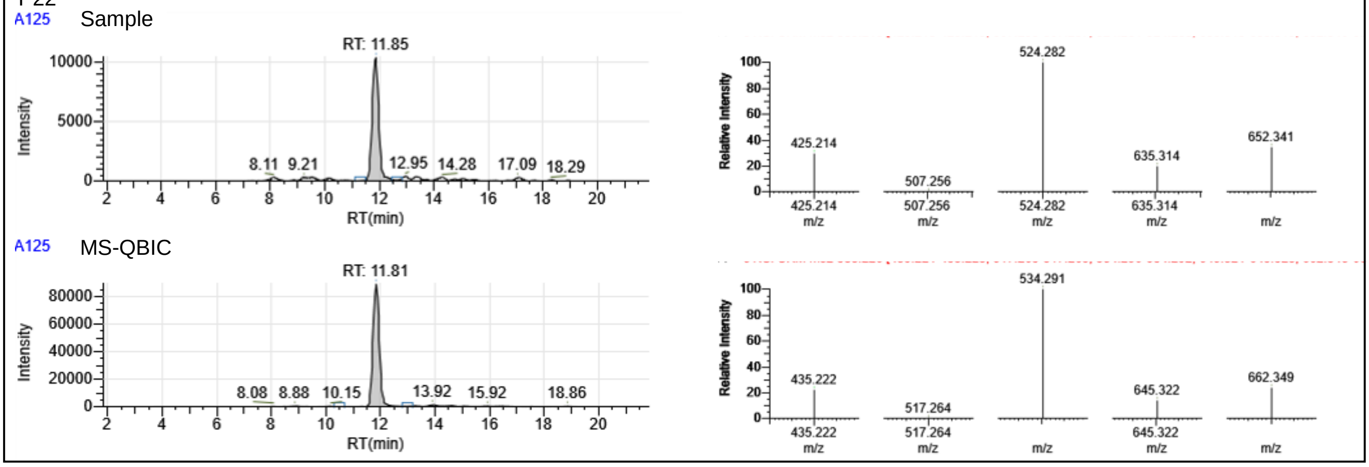

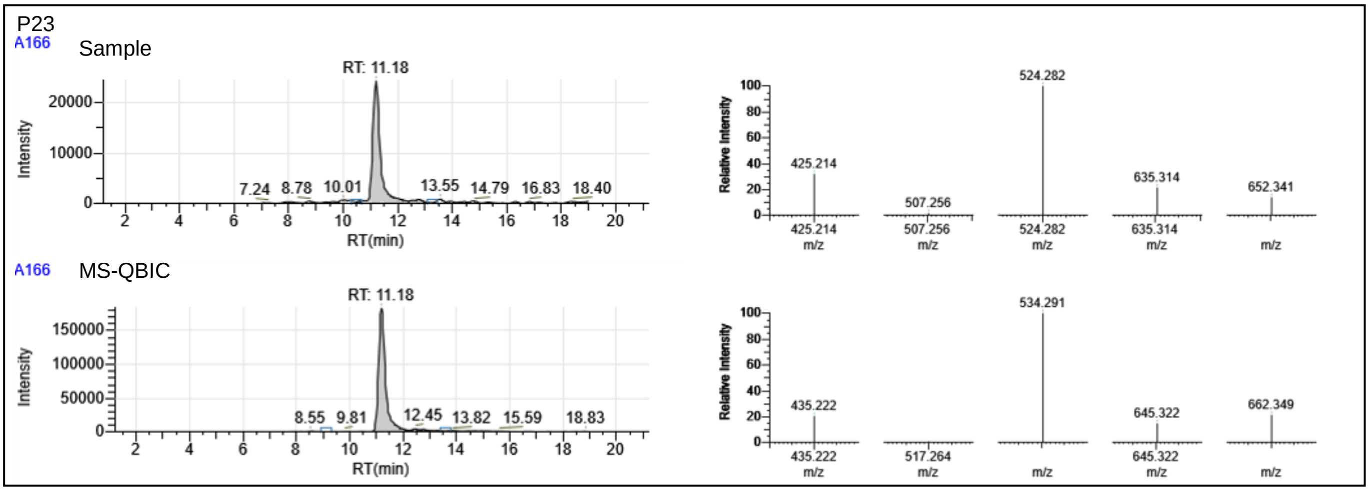
P23
A166 Sample
RT: 11.18
Intensity
RT(min)
7.24 8.78 10.01 13.55 14.79 16.83 18.40
Relative Intensity
425.214 507.256 524.282 635.314 652.341
m/z
A166 MS-QBIC
RT: 11.18
8.55 9.81 12.45 13.82 15.59 18.83
435.222 517.264 534.291 645.322 662.349

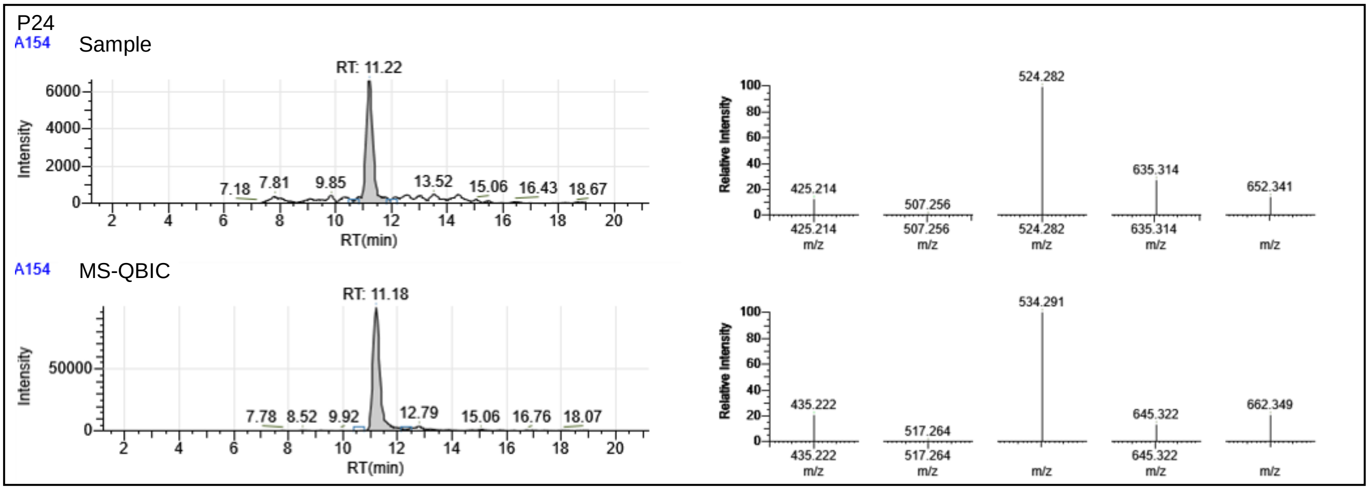
P24
A154 Sample
RT: 11.22
Intensity
RT(min)
7.18 7.81 9.85 13.52 15.06 16.43 18.67
Relative Intensity
425.214 507.256 524.282 635.314 652.341
m/z
A154 MS-QBIC
RT: 11.18
7.78 8.52 9.92 12.79 15.06 16.76 18.07
435.222 517.264 534.291 645.322 662.349

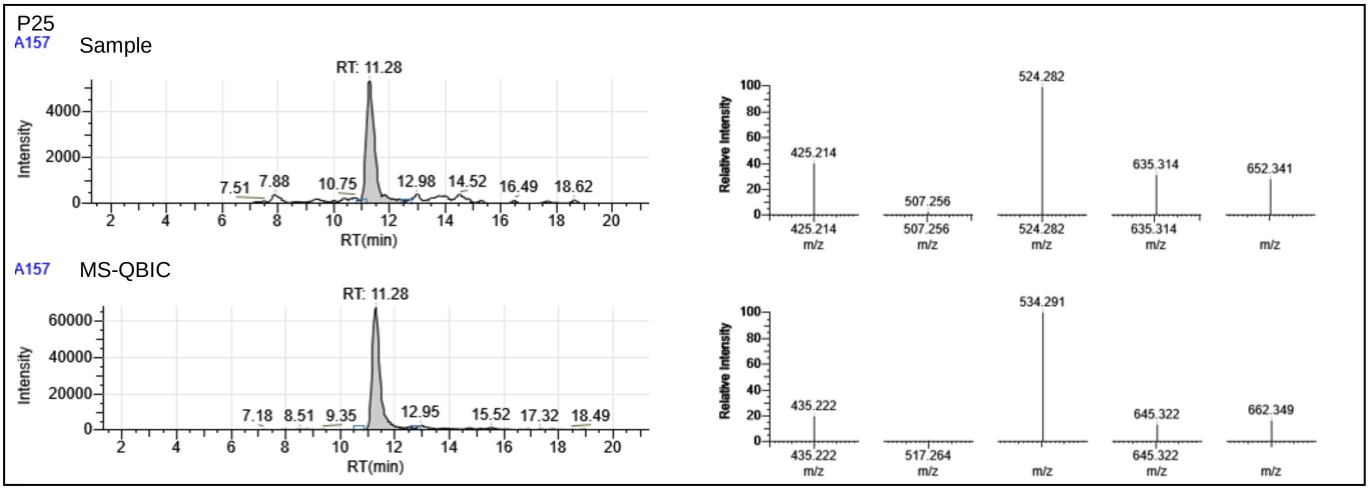
P25
A157 Sample
RT: 11.28
Intensity
RT(min)
7.51 7.88 10.75 12.98 14.52 16.49 18.62
Relative Intensity
425.214 507.256 524.282 635.314 652.341
m/z
A157 MS-QBIC
RT: 11.28
7.18 8.51 9.35 12.95 15.52 17.32 18.49
435.222 517.264 534.291 645.322 662.349

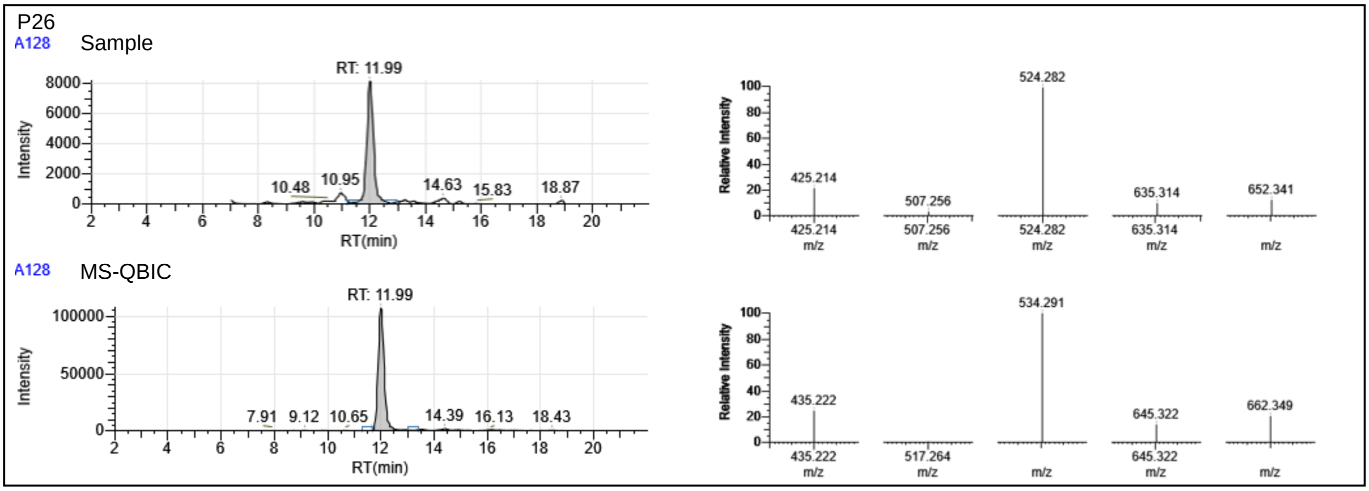
P26
A128 Sample
RT: 11.99
Intensity
RT(min)
10.48 10.95 14.63 15.83 18.87
Relative Intensity
425.214 507.256 524.282 635.314 652.341
m/z
A128 MS-QBIC
RT: 11.99
7.91 9.12 10.65 14.39 16.13 18.43
435.222 517.264 534.291 645.322 662.349

P27

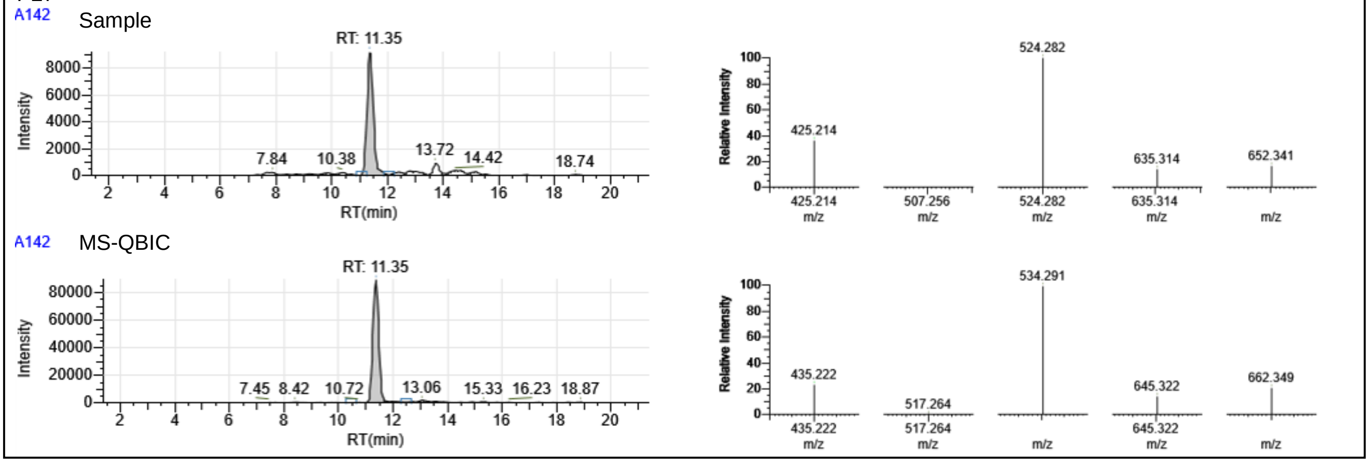

P28

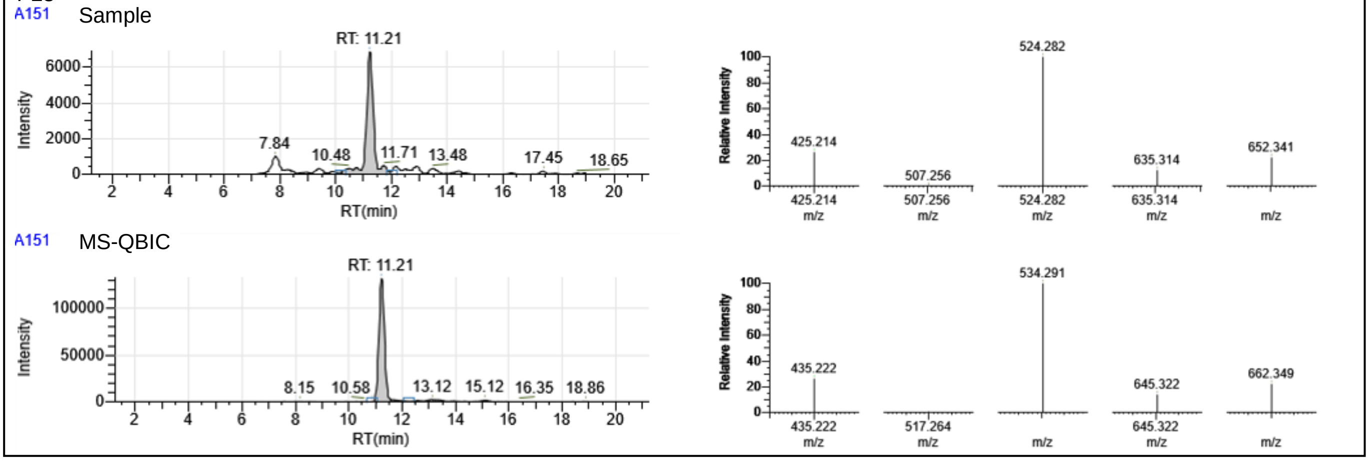

P29

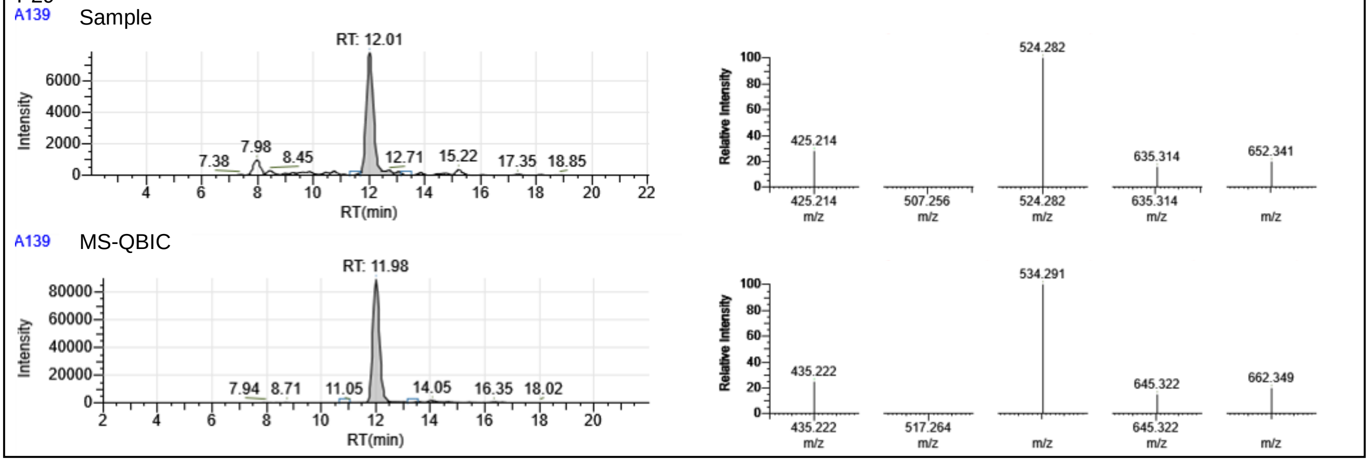

P30

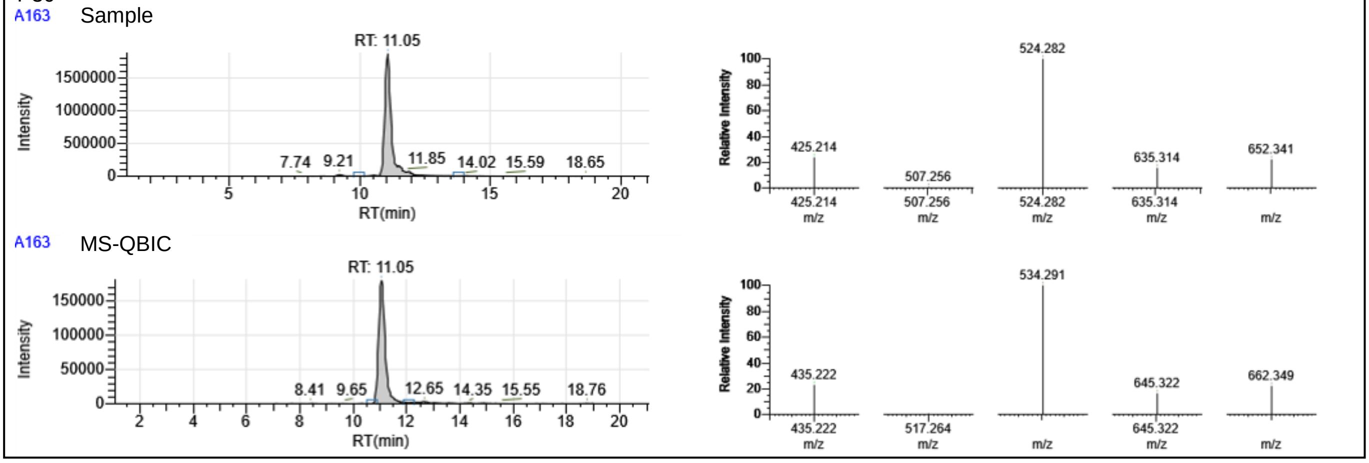

R Apo A1 (DLATVYVDVLK) Another organ

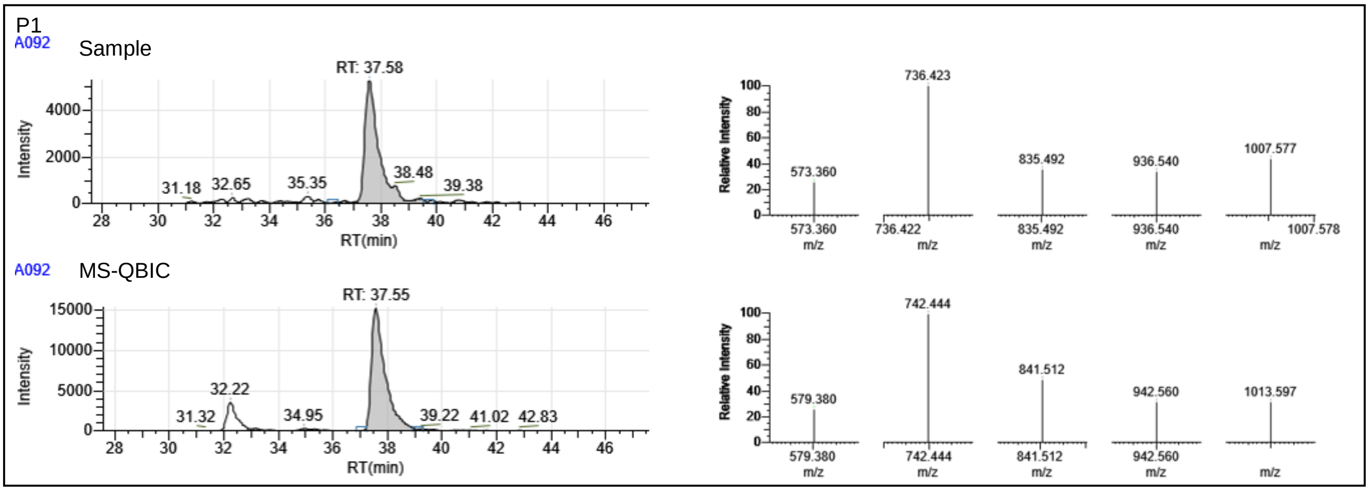

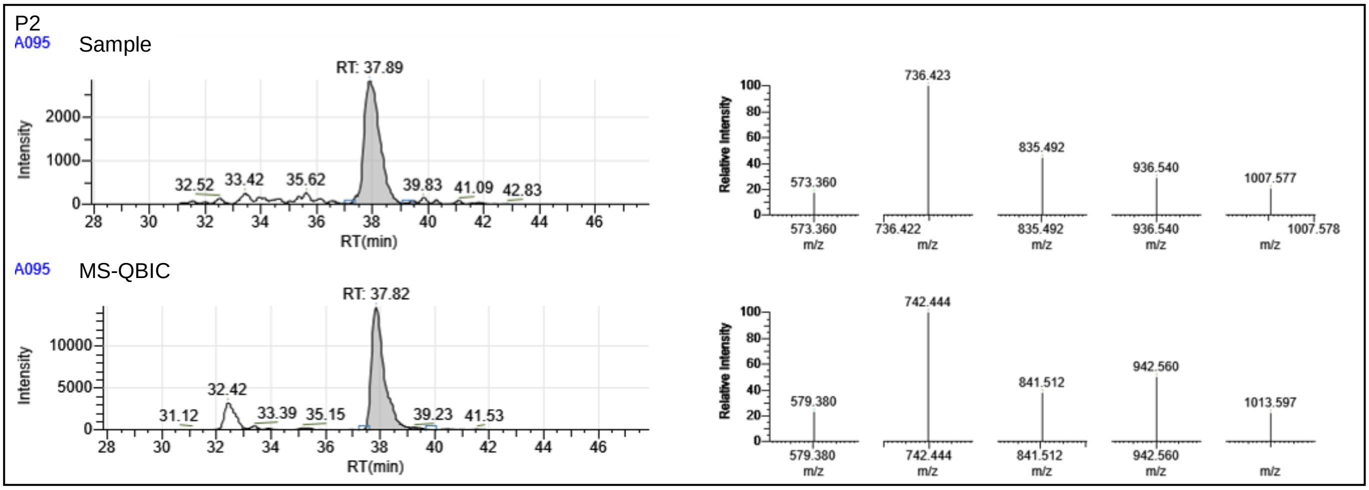

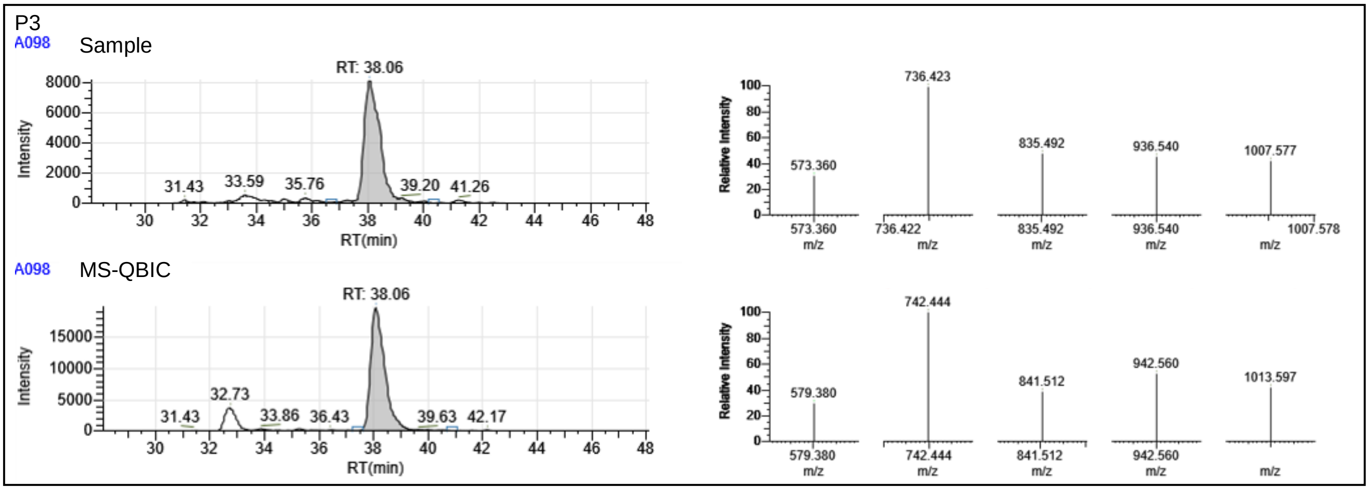

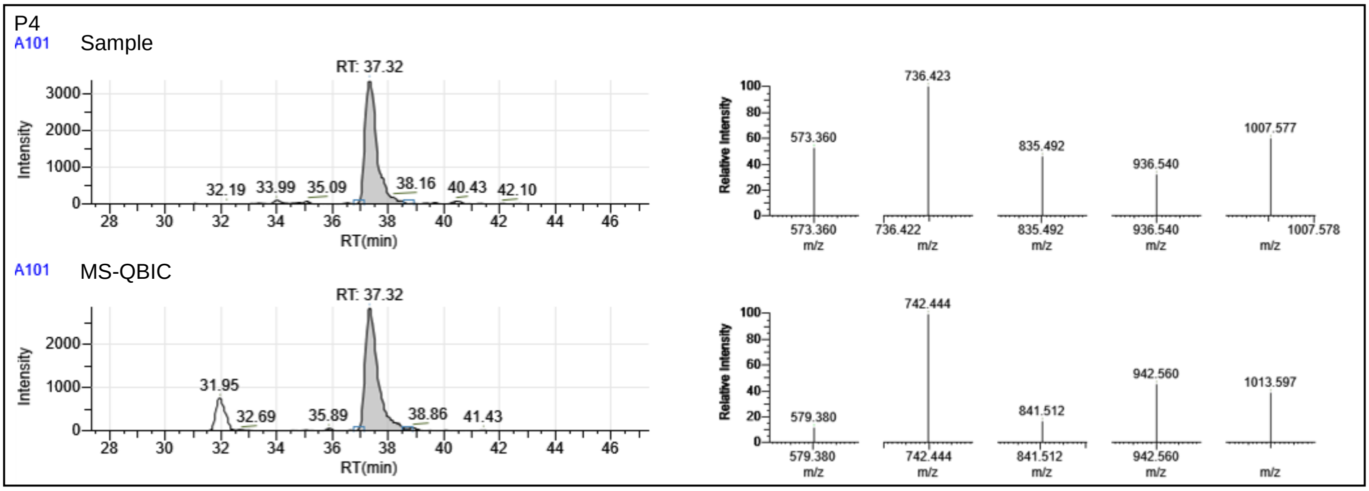

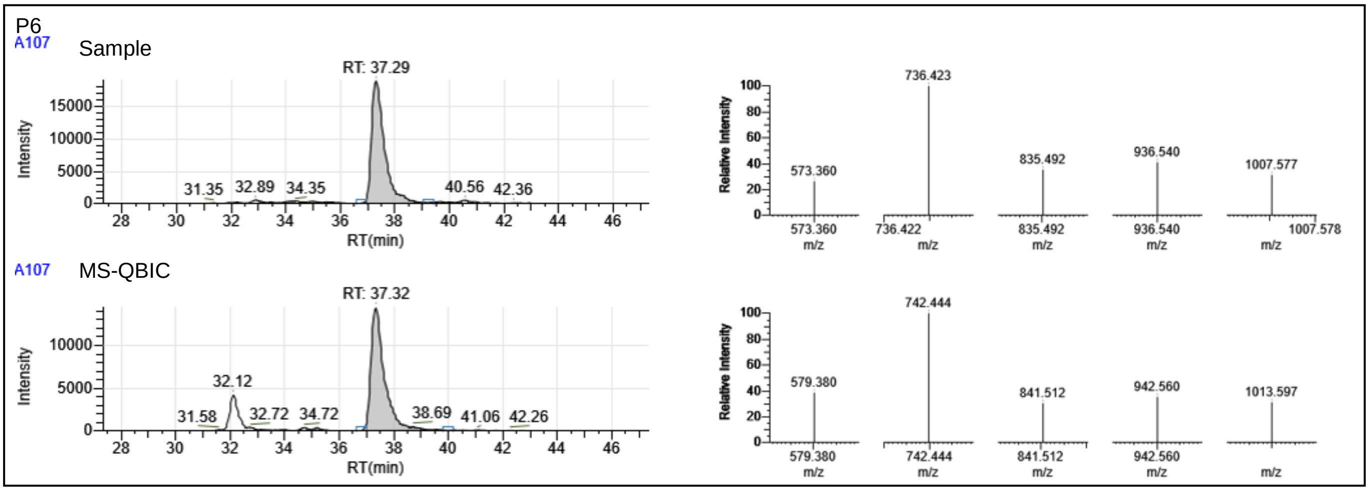
P6
A107 Sample
RT: 37.29
31.35 32.89 34.35 40.56 42.36
Intensity
RT(min)
736.423
573.360 835.492 936.540 1007.577
Relative Intensity
m/z
1007.578
A107 MS-QBIC
RT: 37.32
32.12
31.58 32.72 34.72 38.69 41.06 42.26
742.444
579.380 841.512 942.560 1013.597

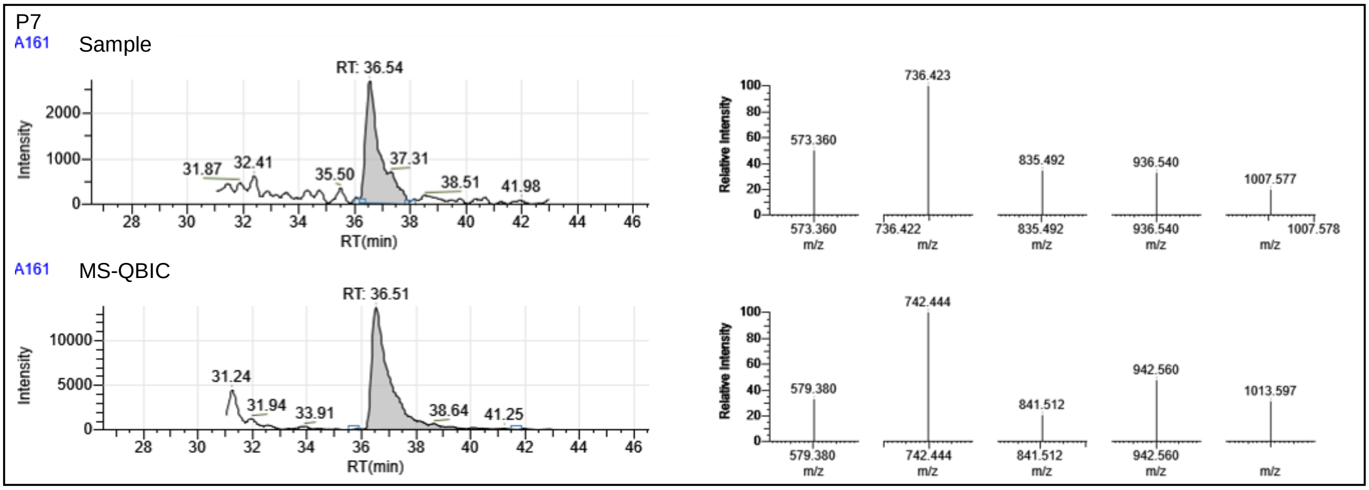
P7
A161 Sample
RT: 36.54
31.87 32.41 35.50 37.31 38.51 41.98
Intensity
RT(min)
736.423
573.360 835.492 936.540 1007.577
Relative Intensity
m/z
1007.578
A161 MS-QBIC
RT: 36.51
31.24 31.94 33.91 38.64 41.25
742.444
579.380 841.512 942.560 1013.597

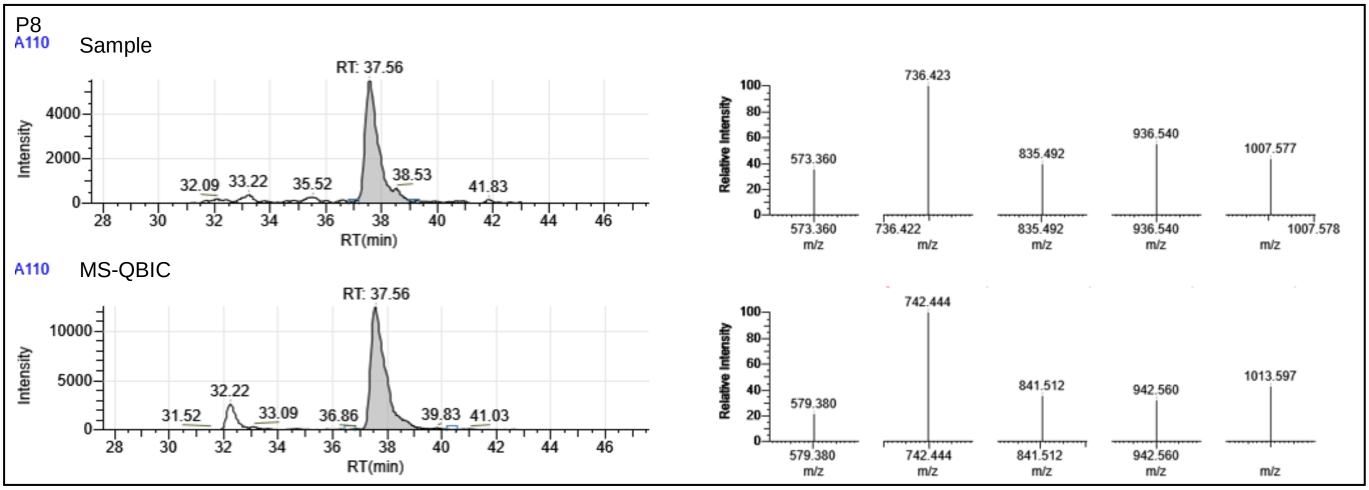
P8
A110 Sample
RT: 37.56
32.09 33.22 35.52 38.53 41.83
Intensity
RT(min)
736.423
573.360 835.492 936.540 1007.577
Relative Intensity
m/z
1007.578
A110 MS-QBIC
RT: 37.56
32.22
31.52 33.09 36.86 39.83 41.03
742.444
579.380 841.512 942.560 1013.597

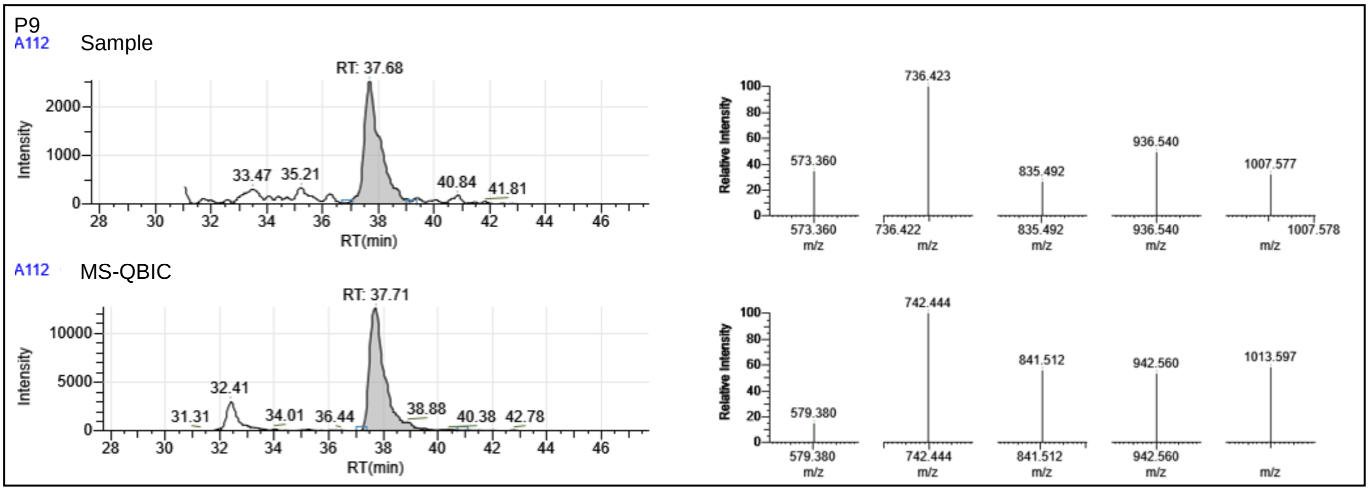
P9
A112 Sample
RT: 37.68
33.47 35.21 40.84 41.81
Intensity
RT(min)
736.423
573.360 835.492 936.540 1007.577
Relative Intensity
m/z
1007.578
A112 MS-QBIC
RT: 37.71
32.41
31.31 34.01 36.44 38.88 40.38 42.78
742.444
579.380 841.512 942.560 1013.597

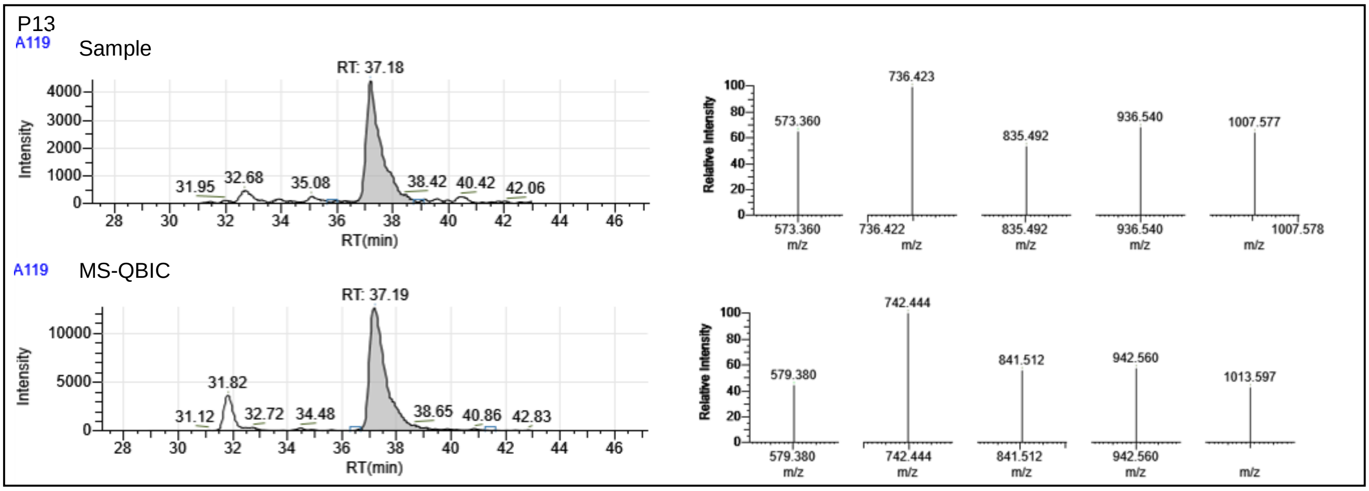
P13
A119 Sample
RT: 37.18
Intensity
31.95
32.68
35.08
38.42
40.42
42.06
RT(min)
Relative Intensity
573.360
736.423
835.492
936.540
1007.577
736.422
1007.578
m/z
A119 MS-QBIC
RT: 37.19
31.82
31.12
32.72
34.48
38.65
40.86
42.83
579.380
742.444
841.512
942.560
1013.597

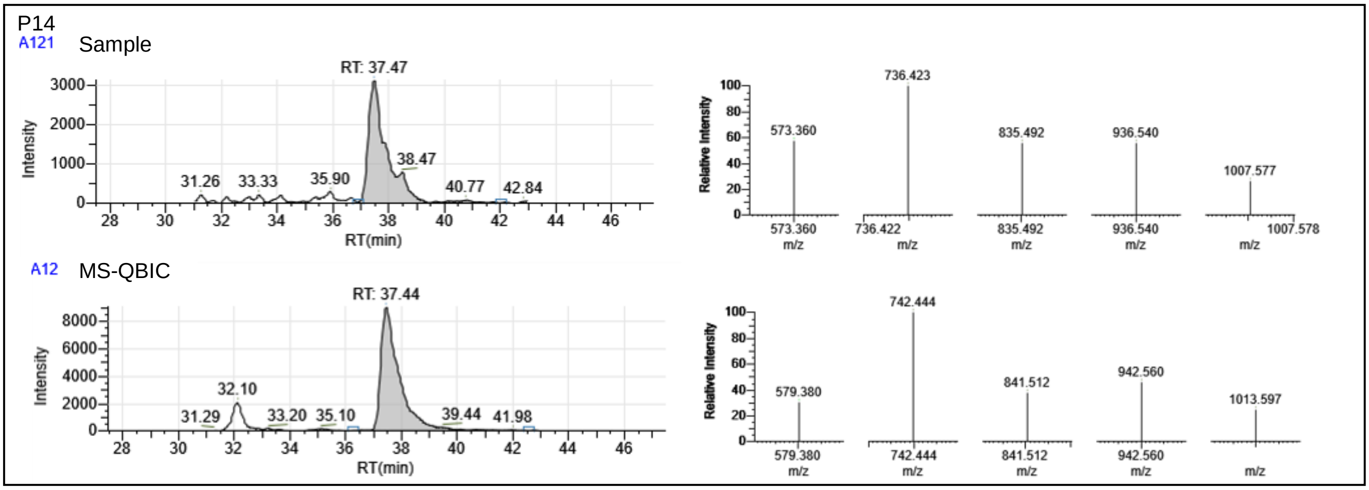
P14
A121 Sample
RT: 37.47
Intensity
31.26
33.33
35.90
38.47
40.77
42.84
RT(min)
Relative Intensity
573.360
736.423
835.492
936.540
1007.577
736.422
1007.578
m/z
A12 MS-QBIC
RT: 37.44
32.10
31.29
33.20
35.10
39.44
41.98
579.380
742.444
841.512
942.560
1013.597

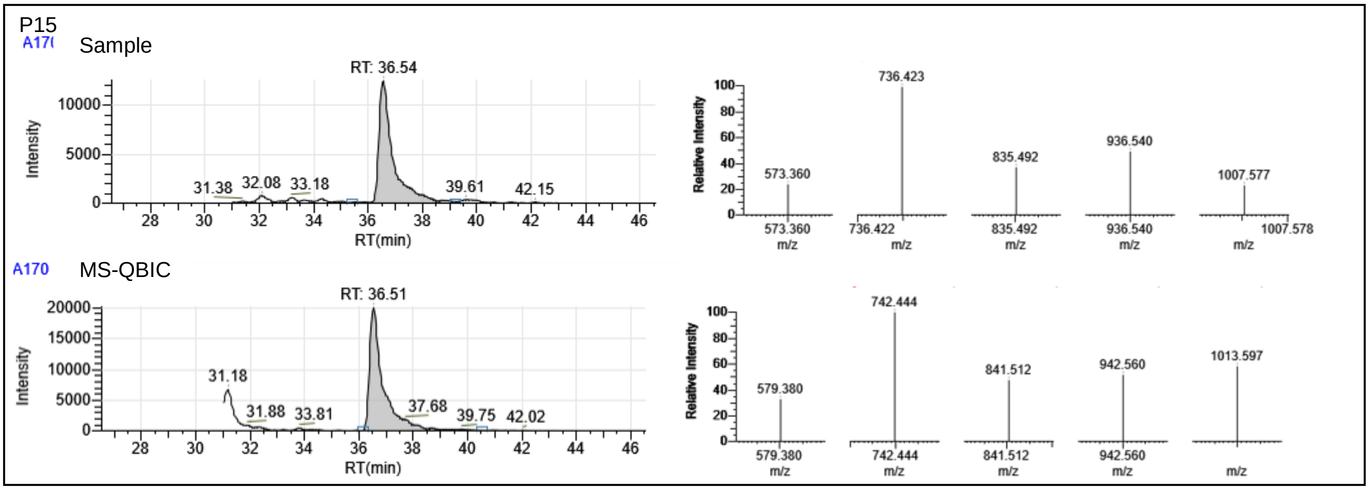
P15
A170 Sample
RT: 36.54
Intensity
31.38
32.08
33.18
39.61
42.15
RT(min)
Relative Intensity
573.360
736.423
835.492
936.540
1007.577
736.422
1007.578
m/z
A170 MS-QBIC
RT: 36.51
31.18
31.88
33.81
37.68
39.75
42.02
579.380
742.444
841.512
942.560
1013.597

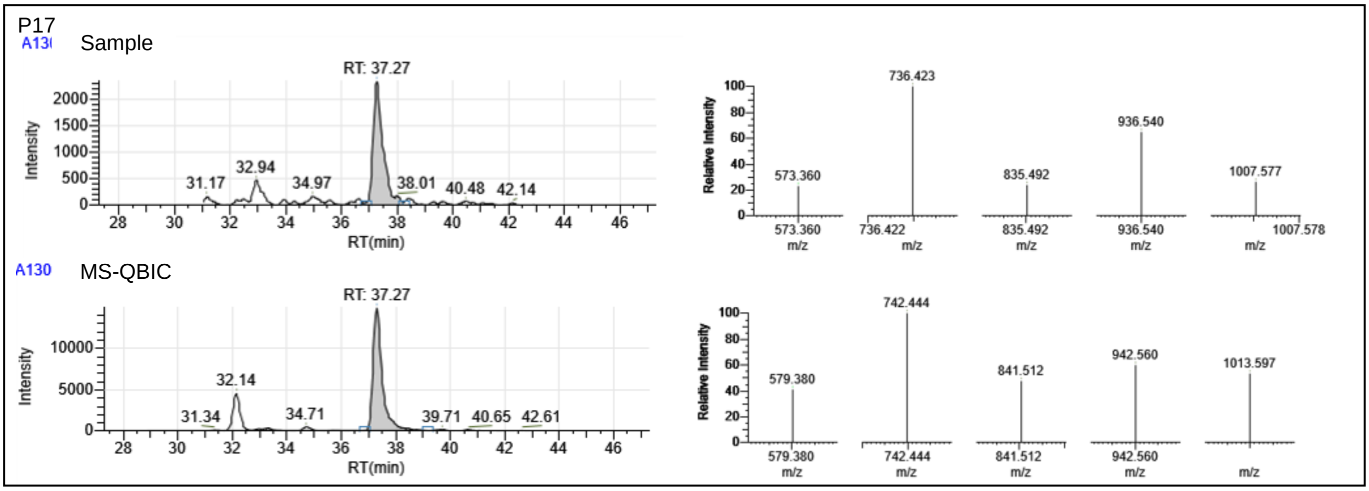
P17
A130 Sample
RT: 37.27
Intensity
31.17
32.94
34.97
38.01
40.48
42.14
RT(min)
Relative Intensity
573.360
736.423
835.492
936.540
1007.577
736.422
1007.578
m/z
A130 MS-QBIC
RT: 37.27
32.14
31.34
34.71
39.71
40.65
42.61
579.380
742.444
841.512
942.560
1013.597

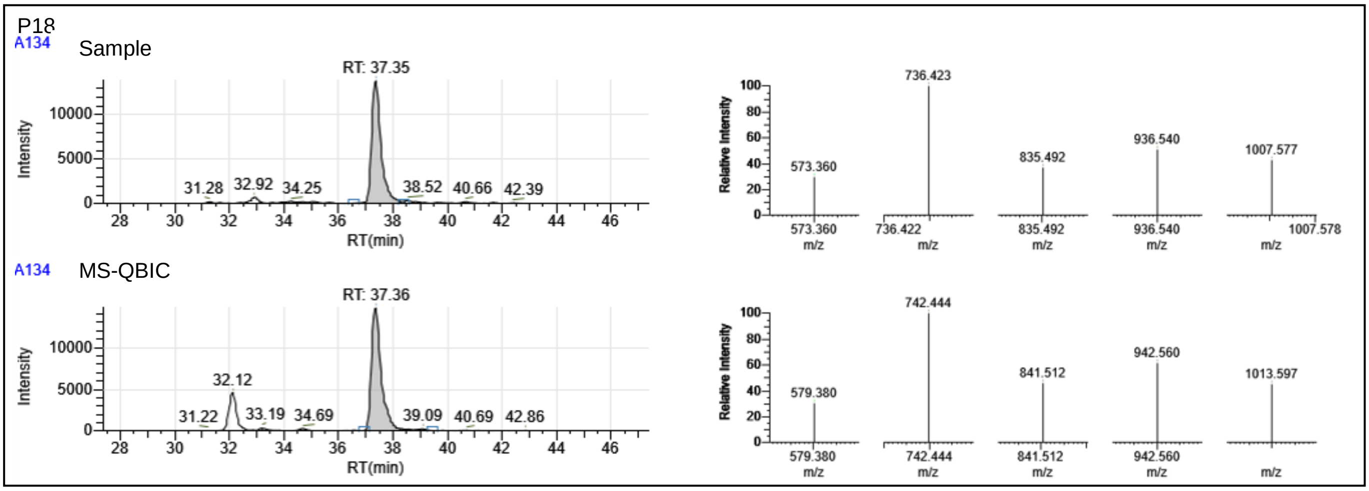
P18
A134 Sample
RT: 37.35
31.28 32.92 34.25 38.52 40.66 42.39
Intensity
RT(min)
Relative Intensity
573.360 736.423 835.492 936.540 1007.577
736.422 1007.578
m/z
A134 MS-QBIC
RT: 37.36
32.12 31.22 33.19 34.69 39.09 40.69 42.86
579.380 742.444 841.512 942.560 1013.597

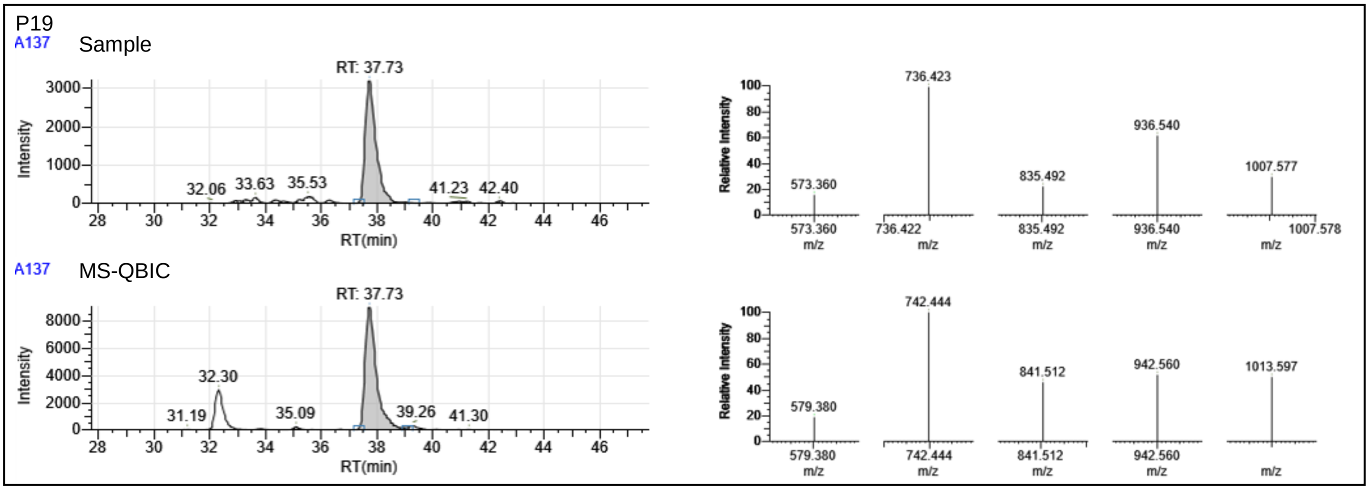
P19
A137 Sample
RT: 37.73
32.06 33.63 35.53 41.23 42.40
Intensity
RT(min)
Relative Intensity
573.360 736.423 835.492 936.540 1007.577
736.422 1007.578
m/z
A137 MS-QBIC
RT: 37.73
32.30 31.19 35.09 39.26 41.30
579.380 742.444 841.512 942.560 1013.597

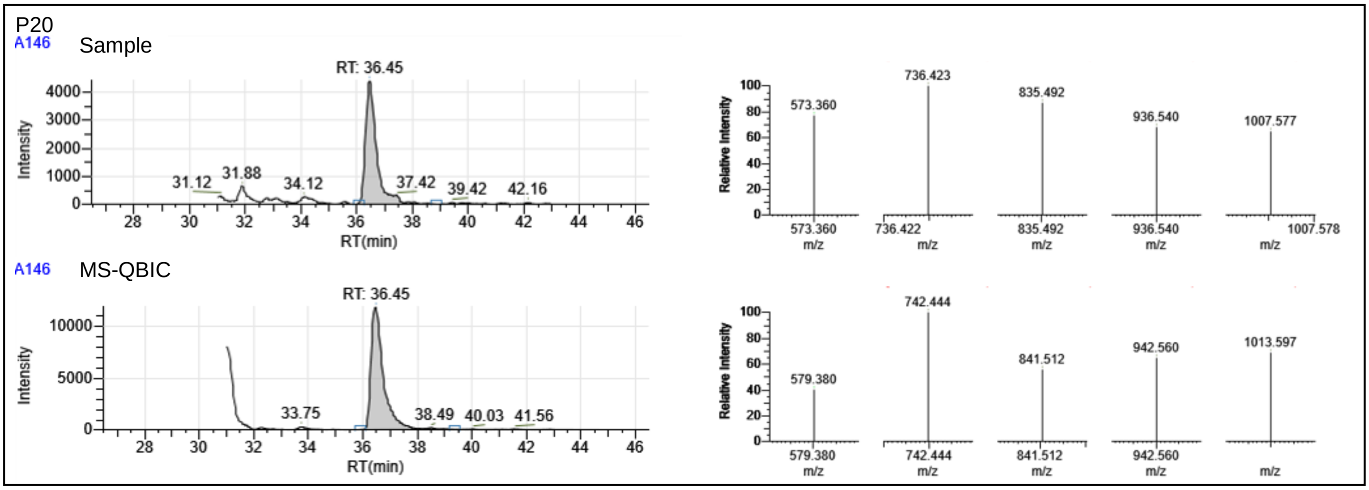
P20
A146 Sample
RT: 36.45
31.12 31.88 34.12 37.42 39.42 42.16
Intensity
RT(min)
Relative Intensity
573.360 736.423 835.492 936.540 1007.577
736.422 1007.578
m/z
A146 MS-QBIC
RT: 36.45
33.75 38.49 40.03 41.56
579.380 742.444 841.512 942.560 1013.597

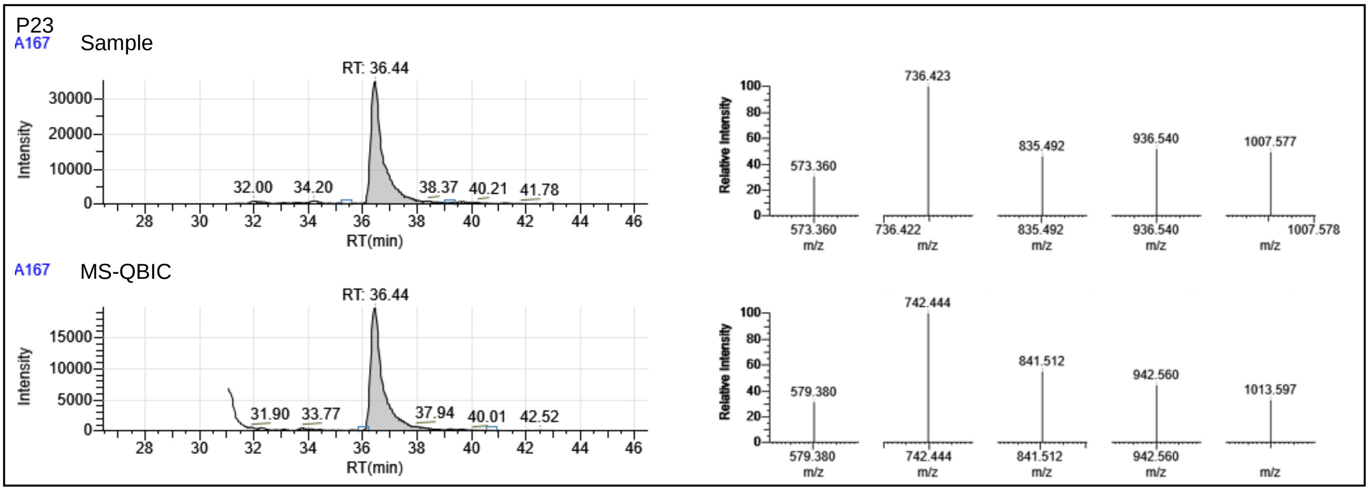
P23
A167 Sample
RT: 36.44
32.00 34.20 38.37 40.21 41.78
Intensity
RT(min)
Relative Intensity
573.360 736.423 835.492 936.540 1007.577
736.422 1007.578
m/z
A167 MS-QBIC
RT: 36.44
31.90 33.77 37.94 40.01 42.52
579.380 742.444 841.512 942.560 1013.597

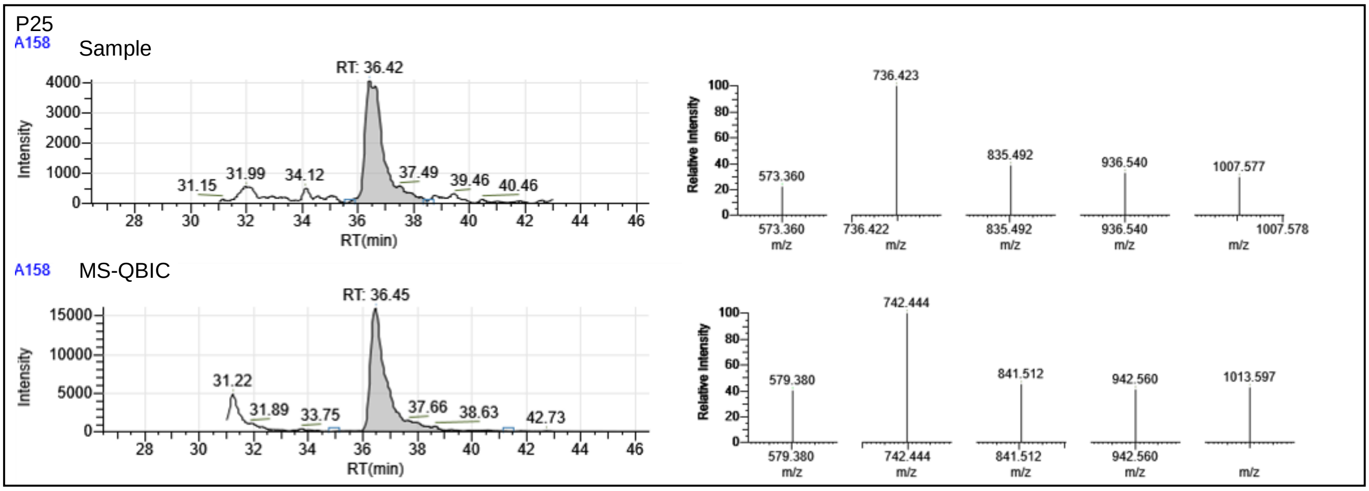
P25
A158 Sample
RT: 36.42
31.15
31.99
34.12
37.49
39.46
40.46
Intensity
RT(min)
Relative Intensity
573.360
736.423
835.492
936.540
1007.577
736.422
1007.578
m/z
A158 MS-QBIC
RT: 36.45
31.22
31.89
33.75
37.66
38.63
42.73
579.380
742.444
841.512
942.560
1013.597

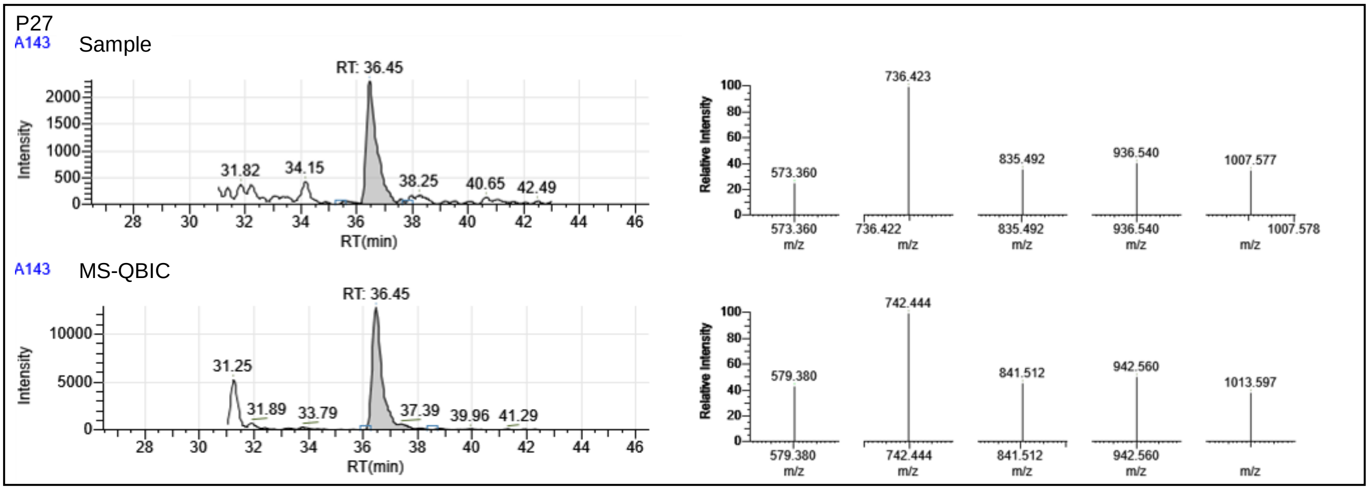
P27
A143 Sample
RT: 36.45
31.82
34.15
38.25
40.65
42.49
Intensity
RT(min)
Relative Intensity
573.360
736.423
835.492
936.540
1007.577
736.422
1007.578
m/z
A143 MS-QBIC
RT: 36.45
31.25
31.89
33.79
37.39
39.96
41.29
579.380
742.444
841.512
942.560
1013.597

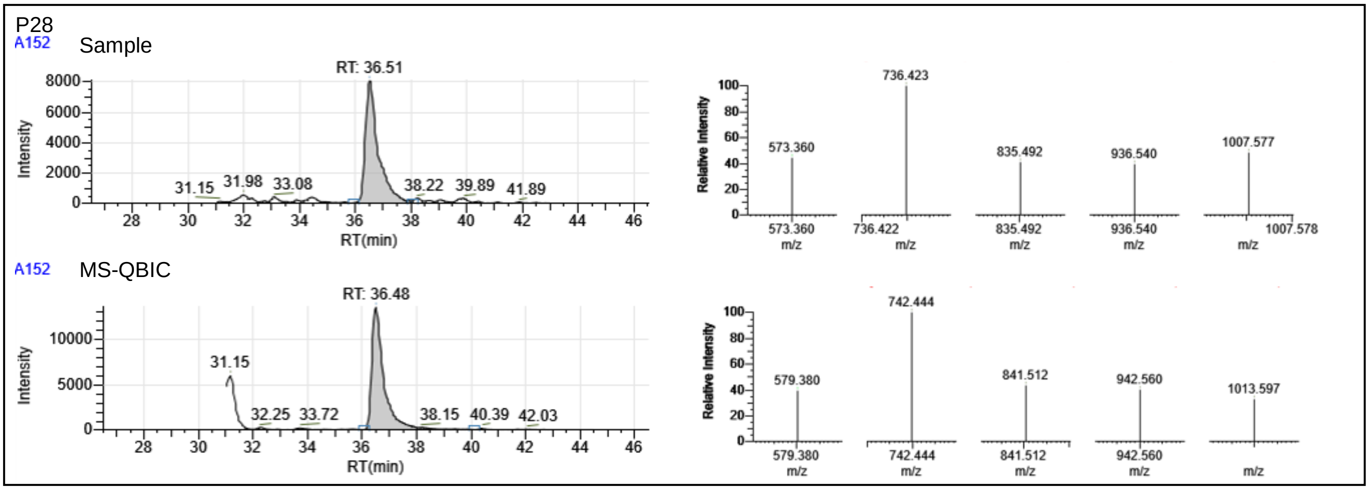
P28
A152 Sample
RT: 36.51
31.15
31.98
33.08
38.22
39.89
41.89
Intensity
RT(min)
Relative Intensity
573.360
736.423
835.492
936.540
1007.577
736.422
1007.578
m/z
A152 MS-QBIC
RT: 36.48
31.15
32.25
33.72
38.15
40.39
42.03
579.380
742.444
841.512
942.560
1013.597

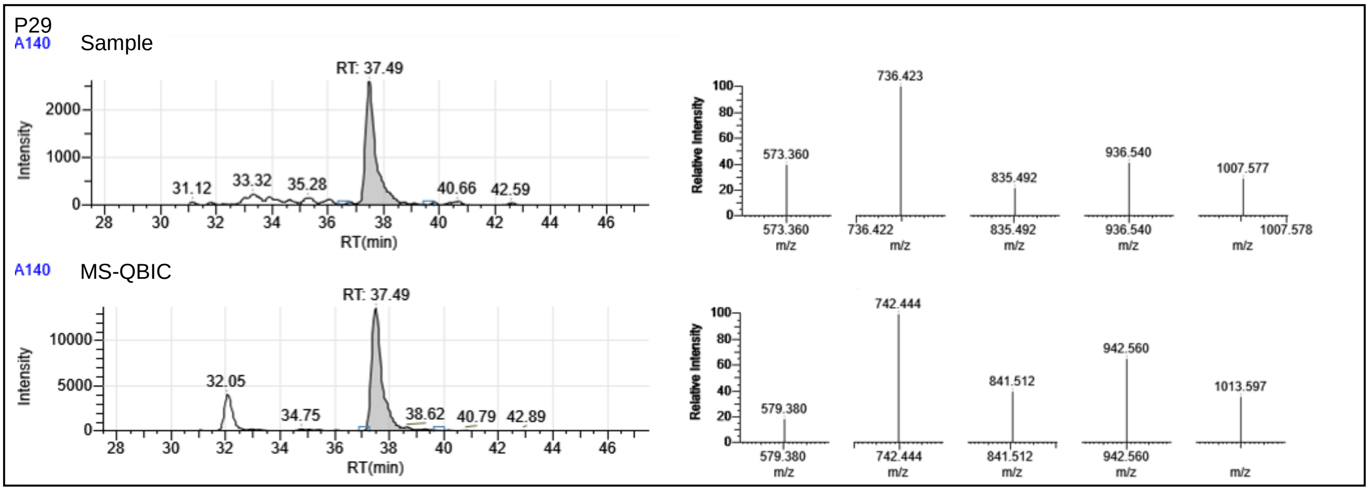
P29
A140 Sample
RT: 37.49
31.12
33.32
35.28
40.66
42.59
Intensity
RT(min)
Relative Intensity
573.360
736.423
835.492
936.540
1007.577
736.422
1007.578
m/z
A140 MS-QBIC
RT: 37.49
32.05
34.75
38.62
40.79
42.89
579.380
742.444
841.512
942.560
1013.597

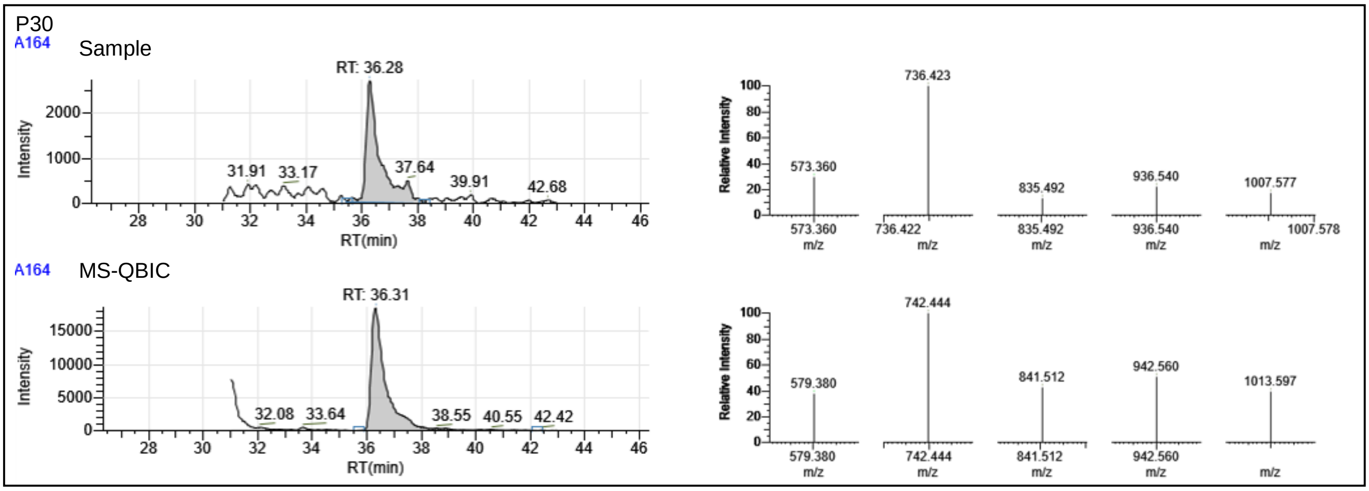
P30
A164
Sample
RT: 36.28
31.91
33.17
37.64
39.91
42.68
Intensity
RT(min)
Relative Intensity
573.360
736.423
835.492
936.540
1007.577
736.422
1007.578
m/z
A164
MS-QBIC
RT: 36.31
32.08
33.64
38.55
40.55
42.42
579.380
742.444
841.512
942.560
1013.597

S Apo A2 (EQLTPLIK) Another organ

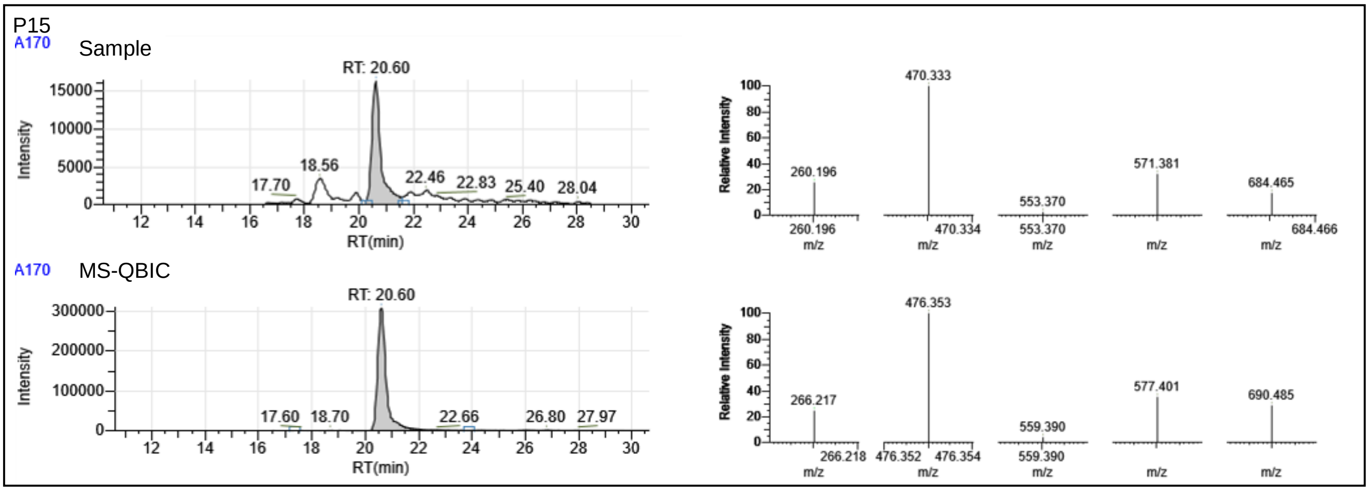

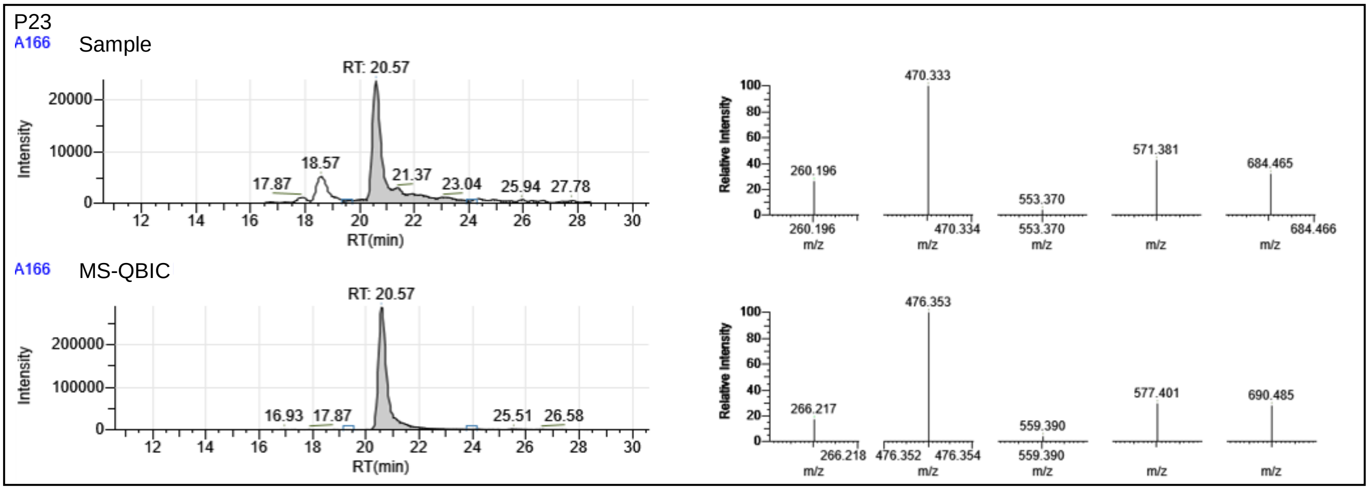

T Apo A4 (LAPLAEDVR) Another organ

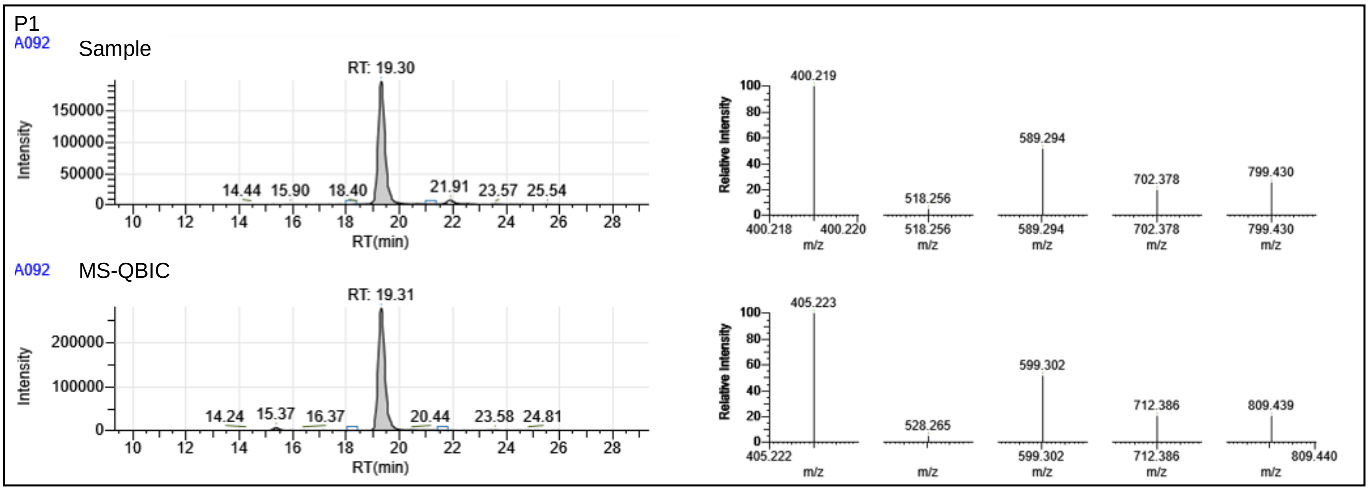

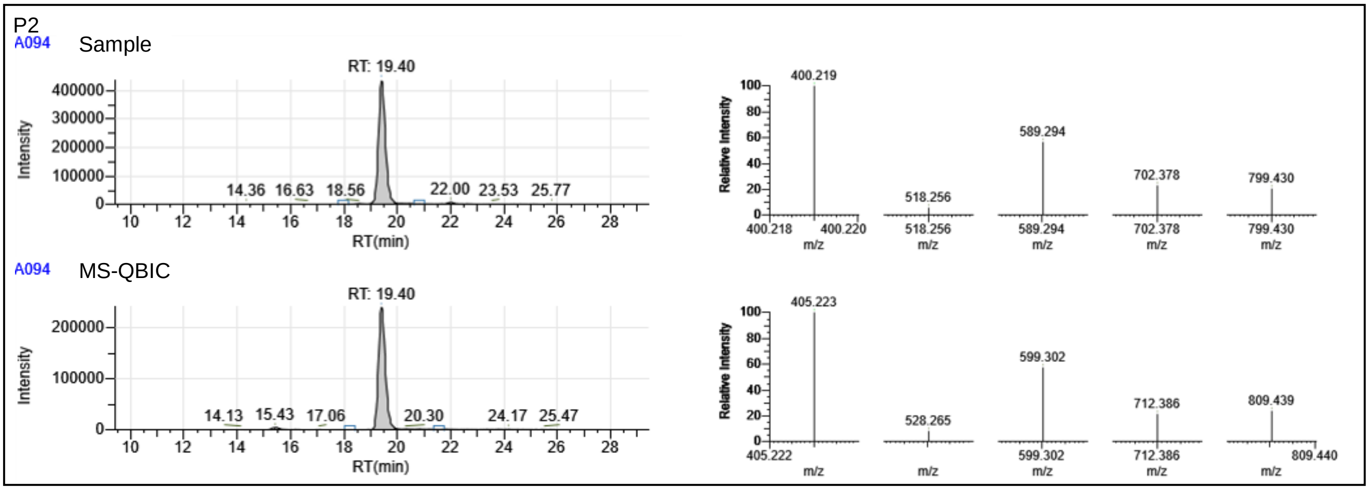

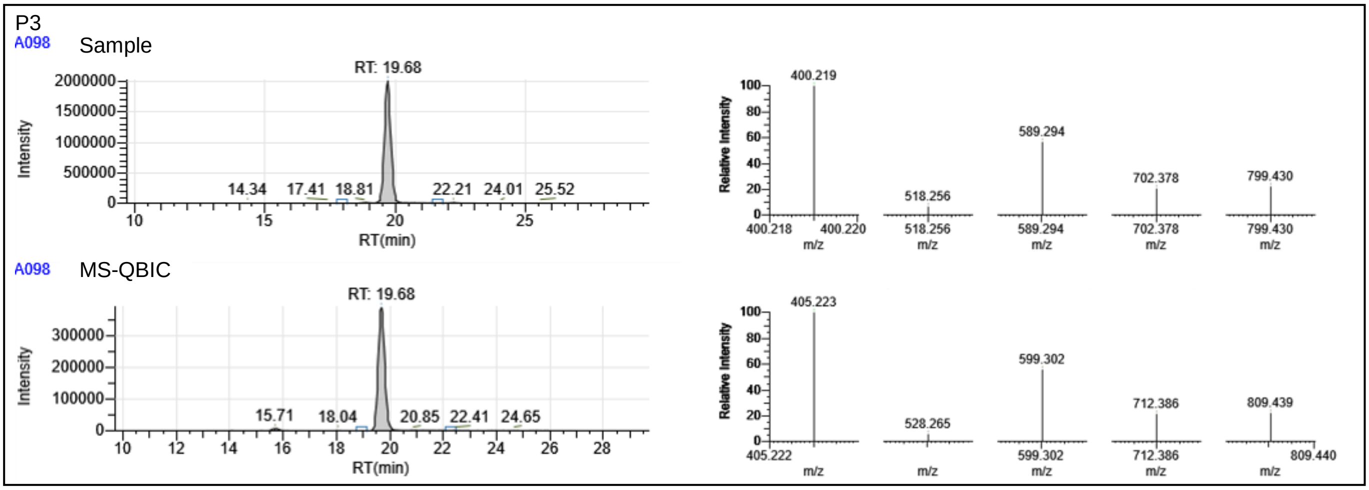

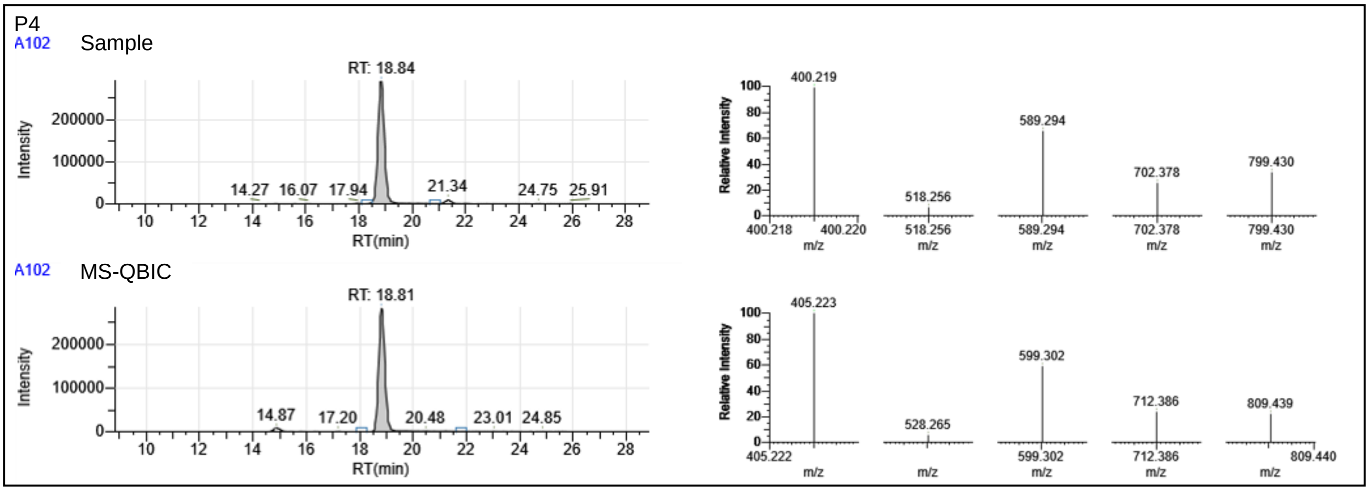

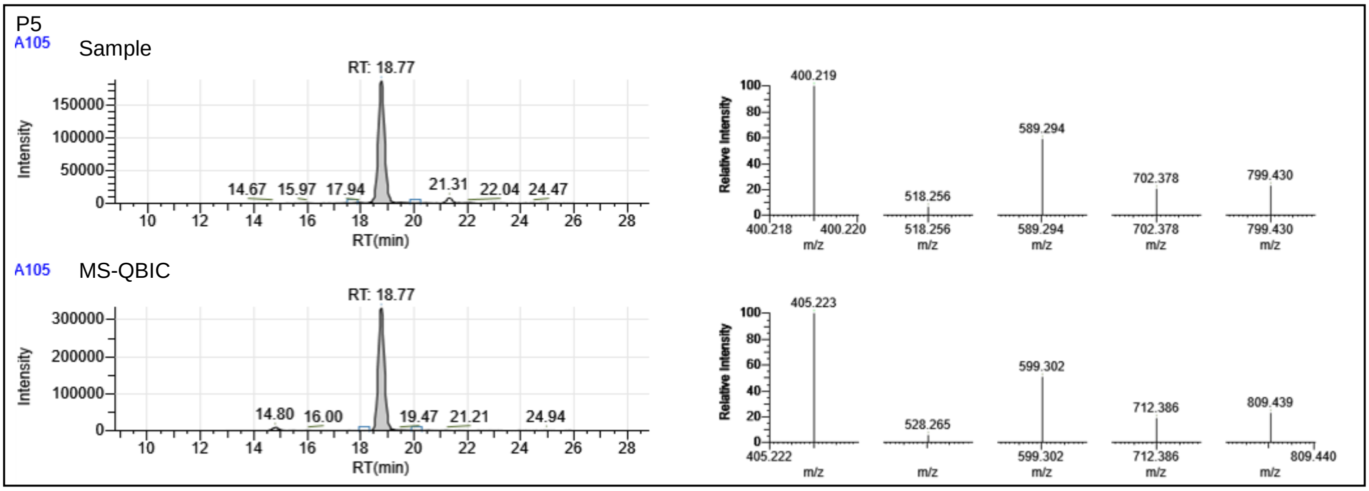
P5
A105 Sample
RT: 18.77
Intensity
RT(min)
14.67 15.97 17.94 21.31 22.04 24.47
Relative Intensity
400.219
518.256
589.294
702.378
799.430
m/z
A105 MS-QBIC
RT: 18.77
14.80 16.00 19.47 21.21 24.94
405.223
528.265
599.302
712.386
809.439

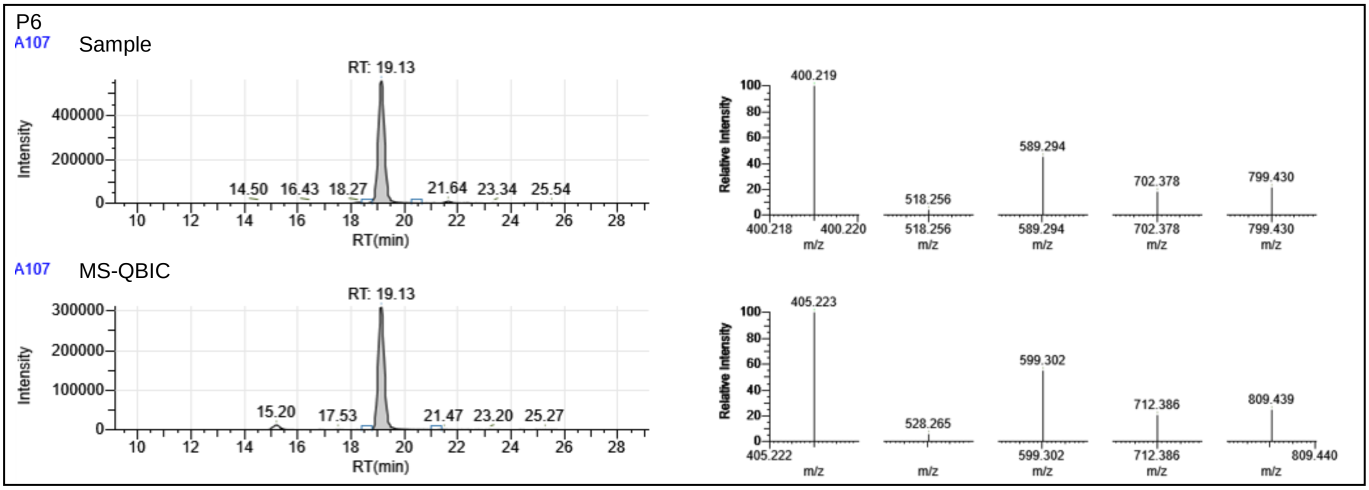
P6
A107 Sample
RT: 19.13
Intensity
RT(min)
14.50 16.43 18.27 21.64 23.34 25.54
Relative Intensity
400.219
518.256
589.294
702.378
799.430
m/z
A107 MS-QBIC
RT: 19.13
15.20 17.53 21.47 23.20 25.27
405.223
528.265
599.302
712.386
809.439

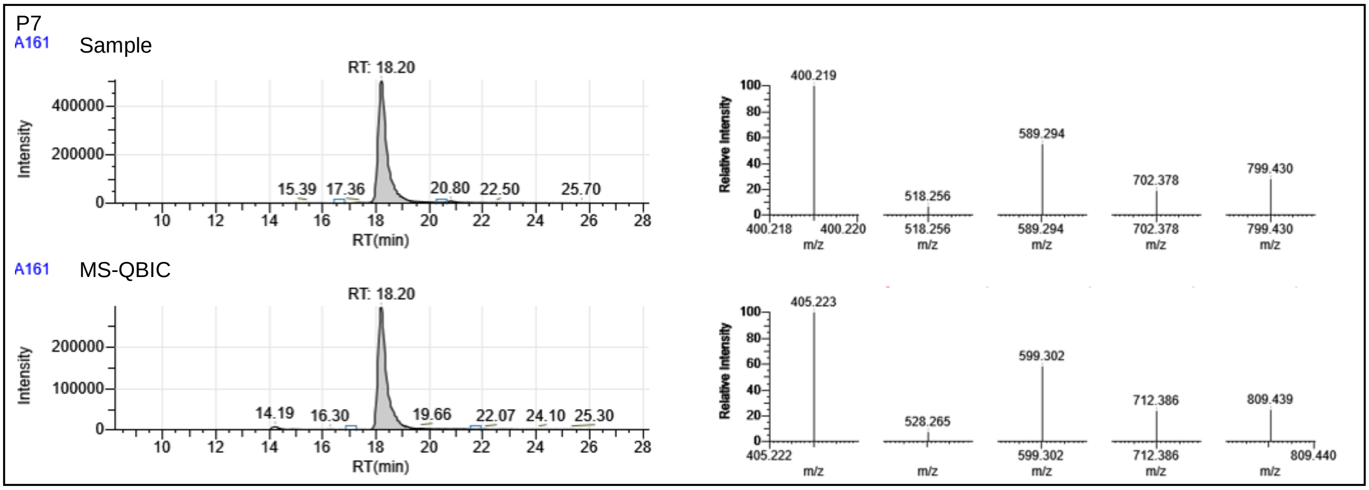
P7
A161 Sample
RT: 18.20
Intensity
RT(min)
15.39 17.36 20.80 22.50 25.70
Relative Intensity
400.219
518.256
589.294
702.378
799.430
m/z
A161 MS-QBIC
RT: 18.20
14.19 16.30 19.66 22.07 24.10 25.30
405.223
528.265
599.302
712.386
809.439

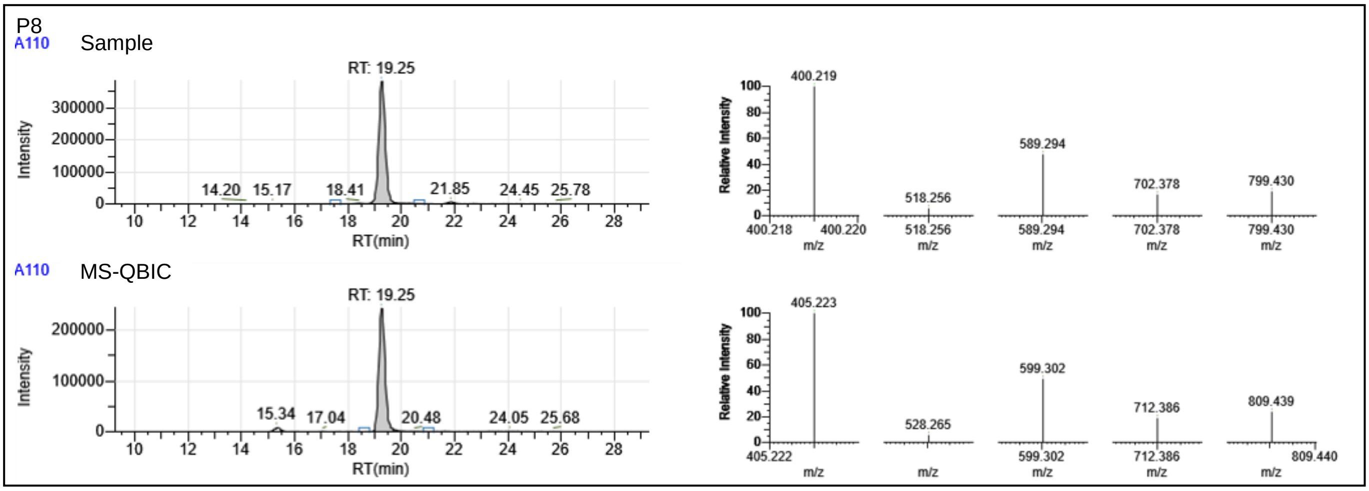
P8
A110 Sample
RT: 19.25
Intensity
RT(min)
14.20 15.17 18.41 21.85 24.45 25.78
Relative Intensity
400.219
518.256
589.294
702.378
799.430
m/z
A110 MS-QBIC
RT: 19.25
15.34 17.04 20.48 24.05 25.68
405.223
528.265
599.302
712.386
809.439

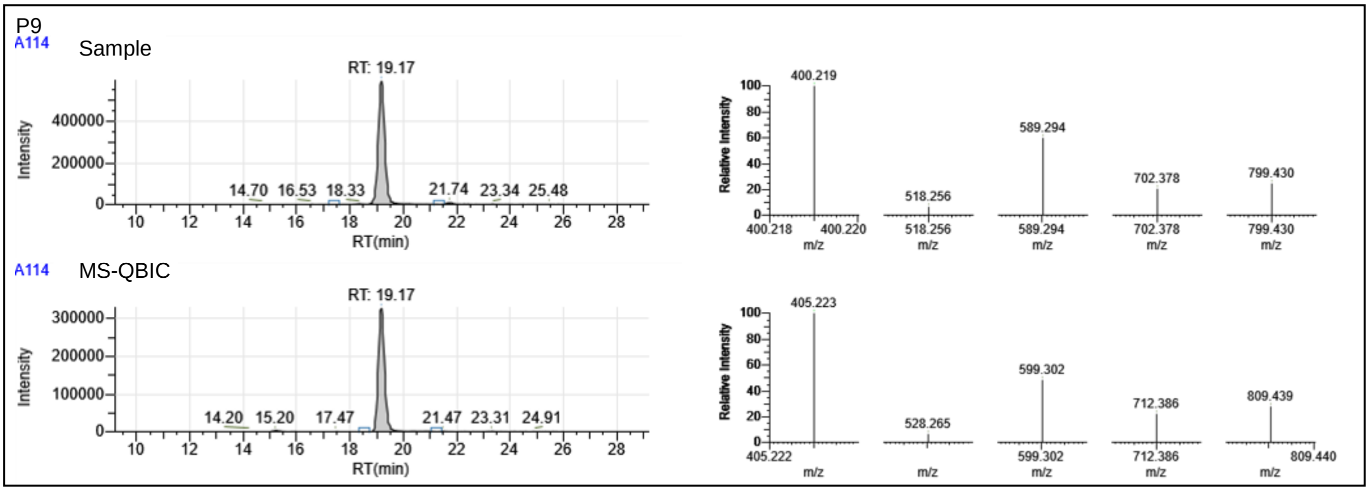
P9
A114 Sample
RT: 19.17
14.70 16.53 18.33 21.74 23.34 25.48
Intensity
RT(min)
400.219
518.256
589.294
702.378
799.430
Relative Intensity
m/z
A114 MS-QBIC
RT: 19.17
14.20 15.20 17.47 21.47 23.31 24.91
405.223
528.265
599.302
712.386
809.439

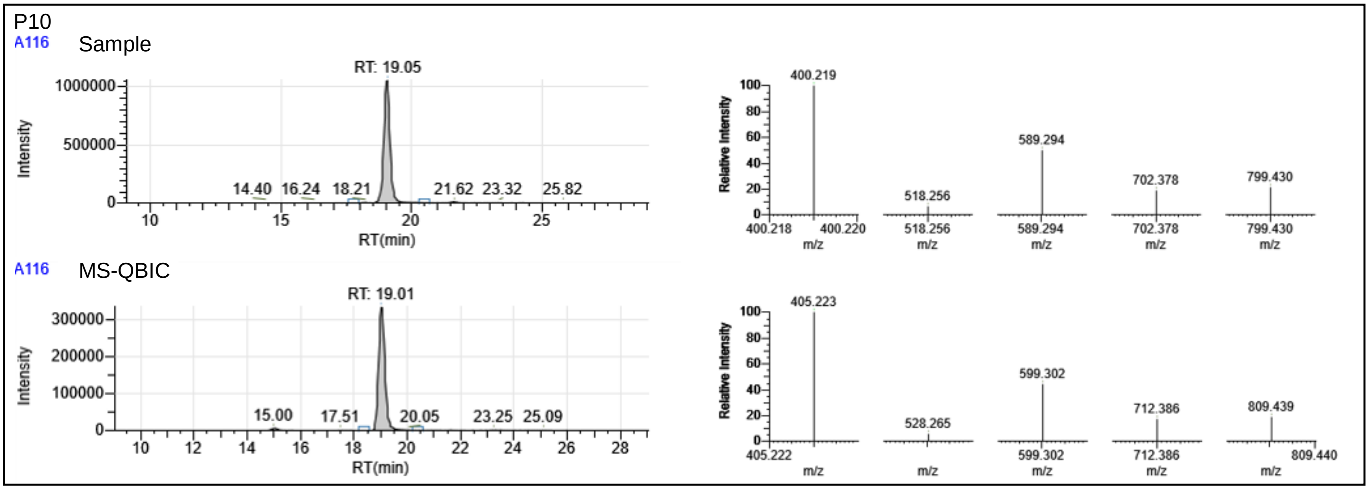
P10
A116 Sample
RT: 19.05
14.40 16.24 18.21 21.62 23.32 25.82
Intensity
RT(min)
400.219
518.256
589.294
702.378
799.430
Relative Intensity
m/z
A116 MS-QBIC
RT: 19.01
15.00 17.51 20.05 23.25 25.09
405.223
528.265
599.302
712.386
809.439

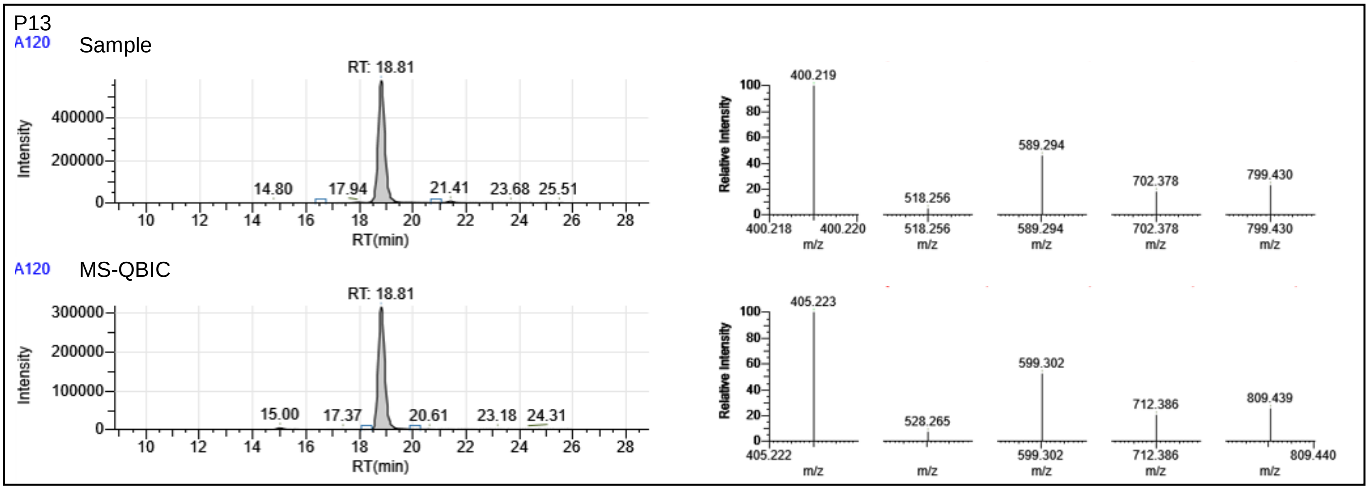
P13
A120 Sample
RT: 18.81
14.80 17.94 21.41 23.68 25.51
Intensity
RT(min)
400.219
518.256
589.294
702.378
799.430
Relative Intensity
m/z
A120 MS-QBIC
RT: 18.81
15.00 17.37 20.61 23.18 24.31
405.223
528.265
599.302
712.386
809.439

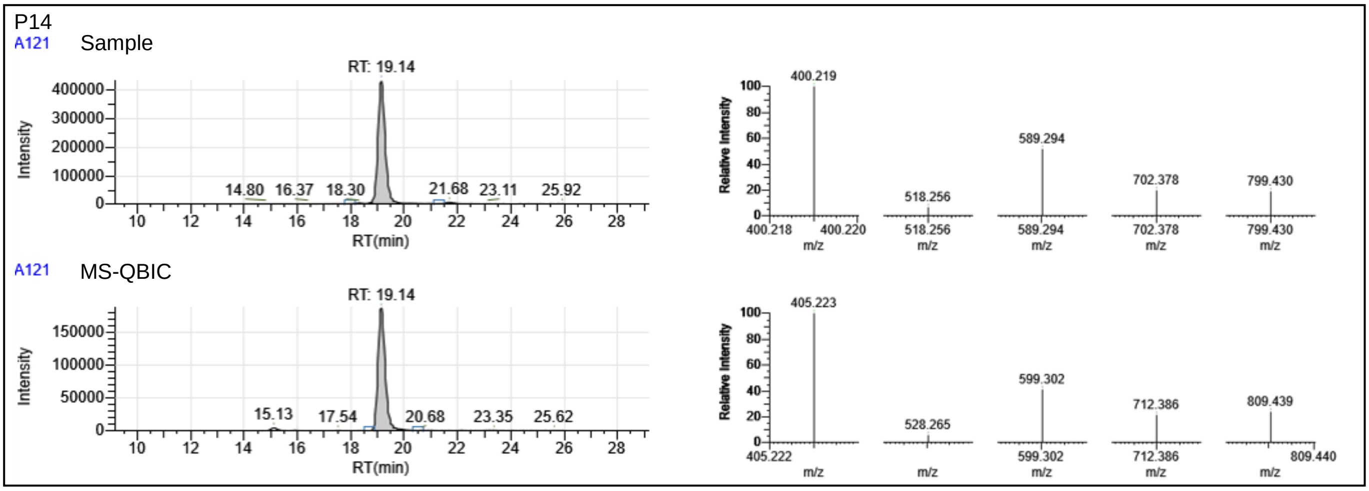
P14
A121 Sample
RT: 19.14
14.80 16.37 18.30 21.68 23.11 25.92
Intensity
RT(min)
400.219
518.256
589.294
702.378
799.430
Relative Intensity
m/z
A121 MS-QBIC
RT: 19.14
15.13 17.54 20.68 23.35 25.62
405.223
528.265
599.302
712.386
809.439

P15

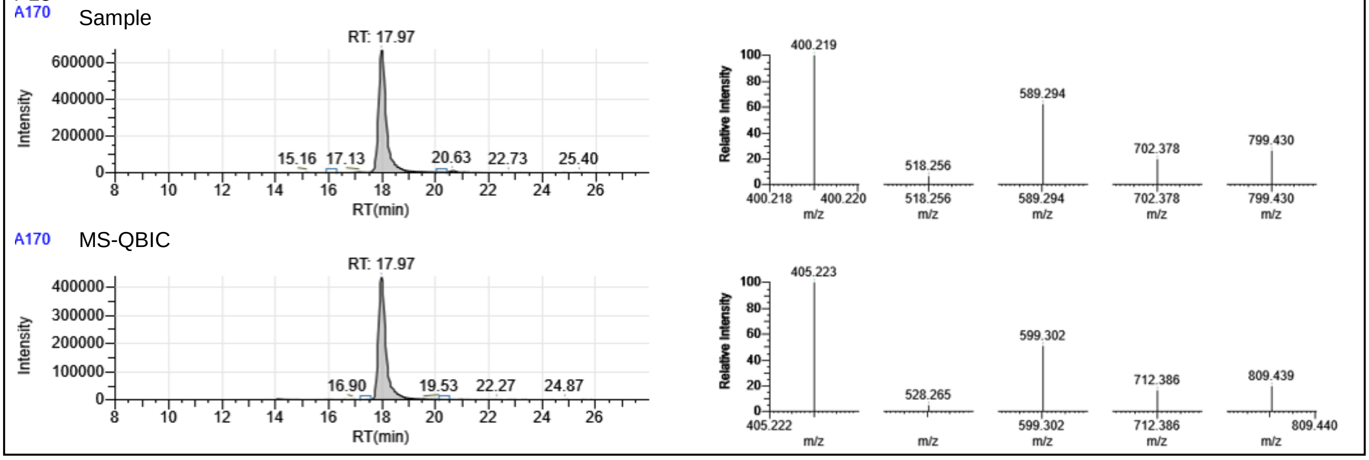

P16

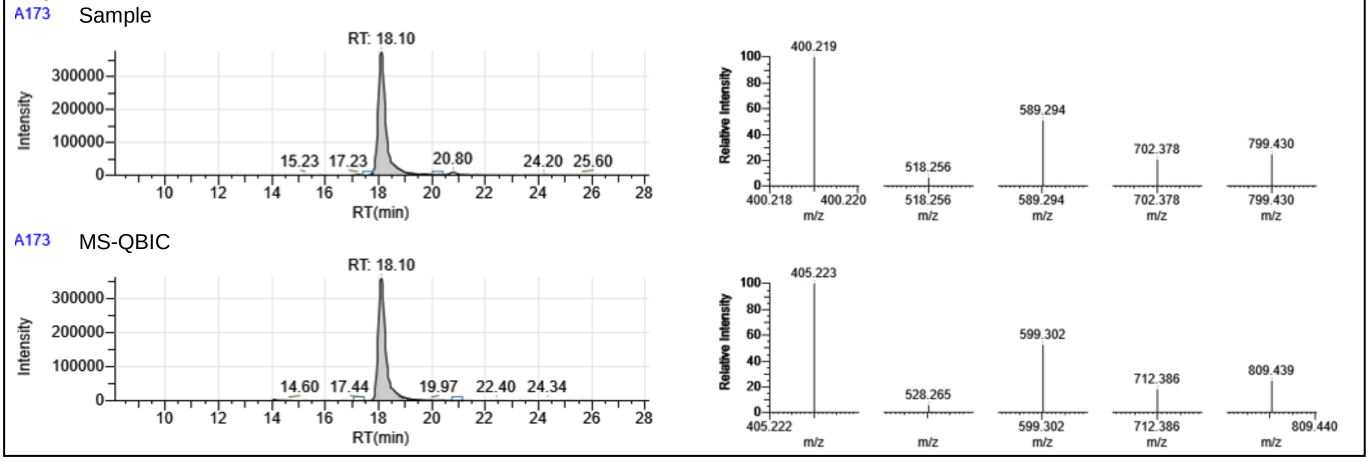

P17

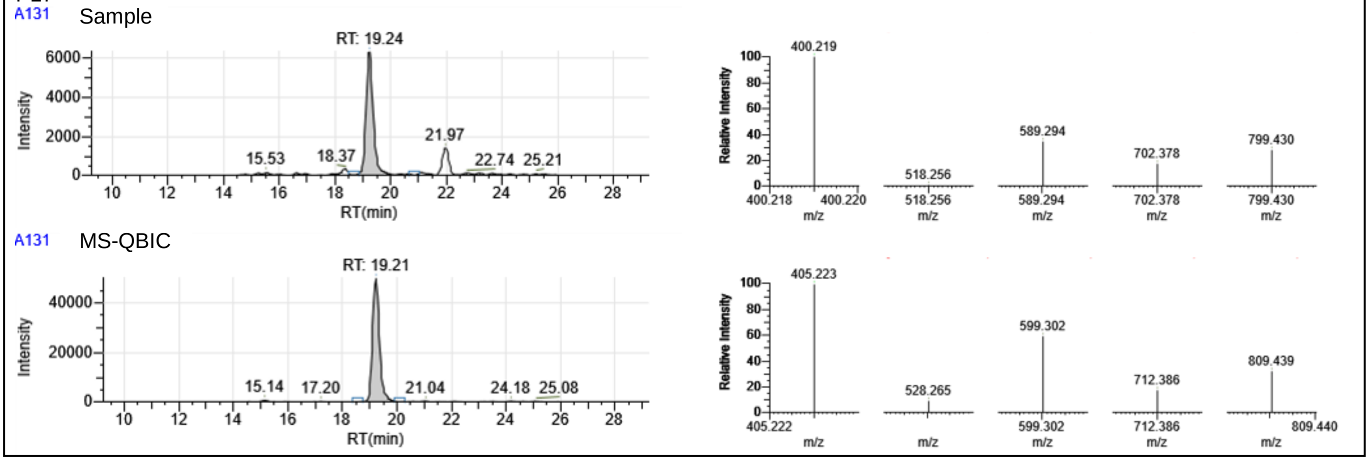

P18

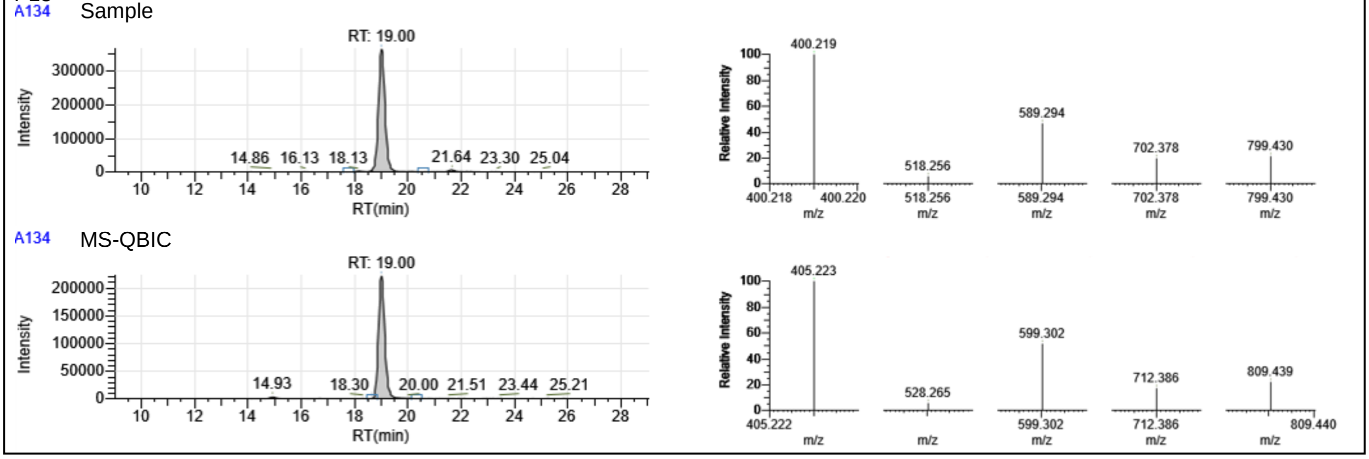

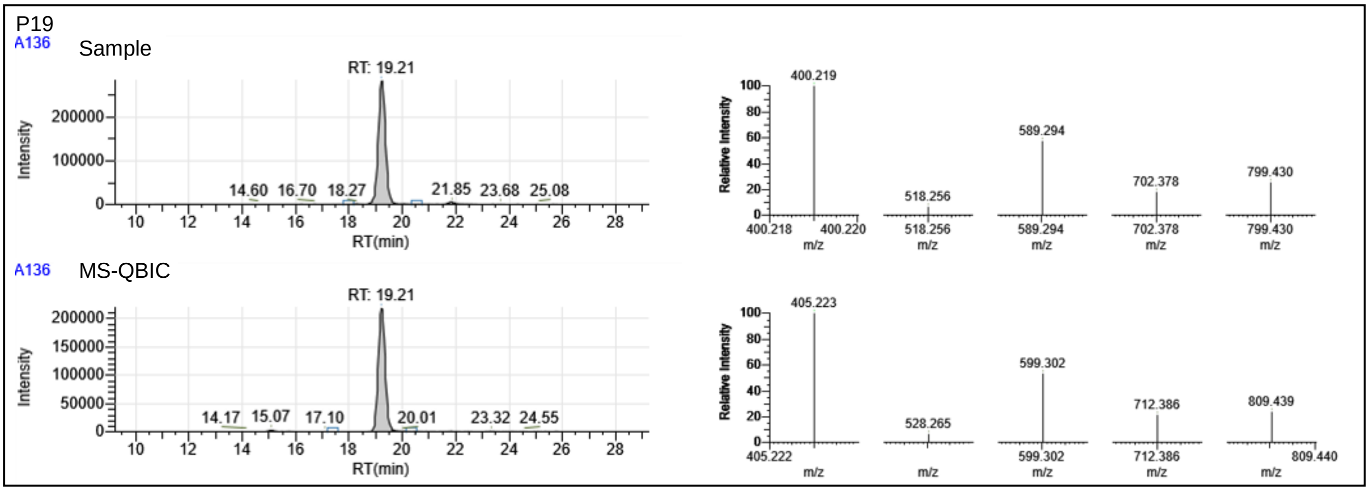
P19
A136 Sample
RT: 19.21
14.60 16.70 18.27 21.85 23.68 25.08
RT(min)
Intensity
400.219
589.294
518.256
702.378
799.430
Relative Intensity
m/z
A136 MS-QBIC
RT: 19.21
14.17 15.07 17.10 20.01 23.32 24.55
405.223
599.302
528.265
712.386
809.439

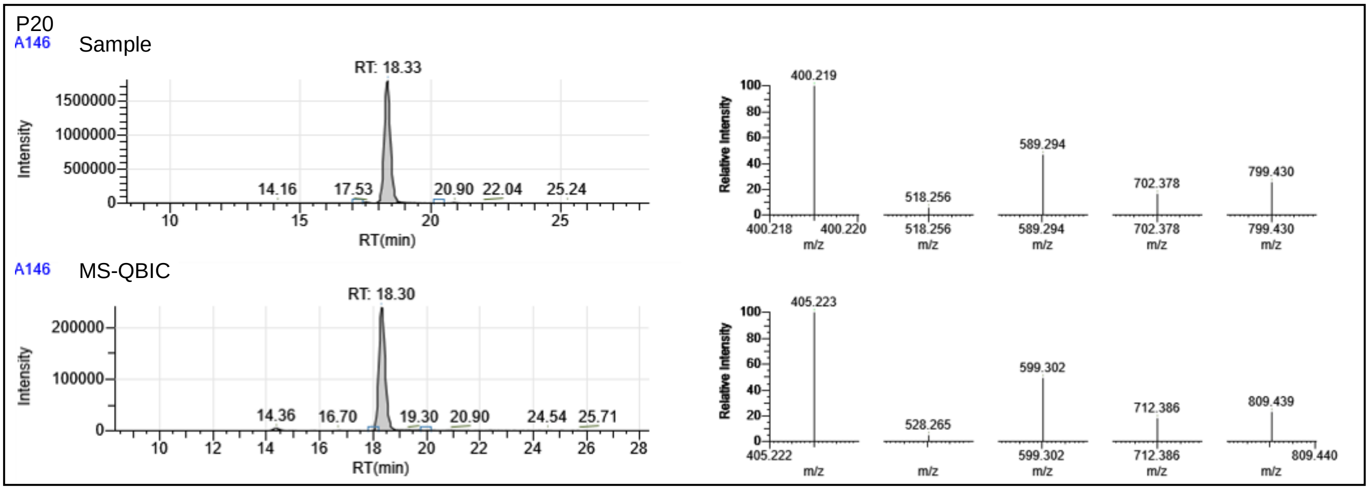
P20
A146 Sample
RT: 18.33
14.16 17.53 20.90 22.04 25.24
RT(min)
Intensity
400.219
589.294
518.256
702.378
799.430
Relative Intensity
m/z
A146 MS-QBIC
RT: 18.30
14.36 16.70 19.30 20.90 24.54 25.71
405.223
599.302
528.265
712.386
809.439

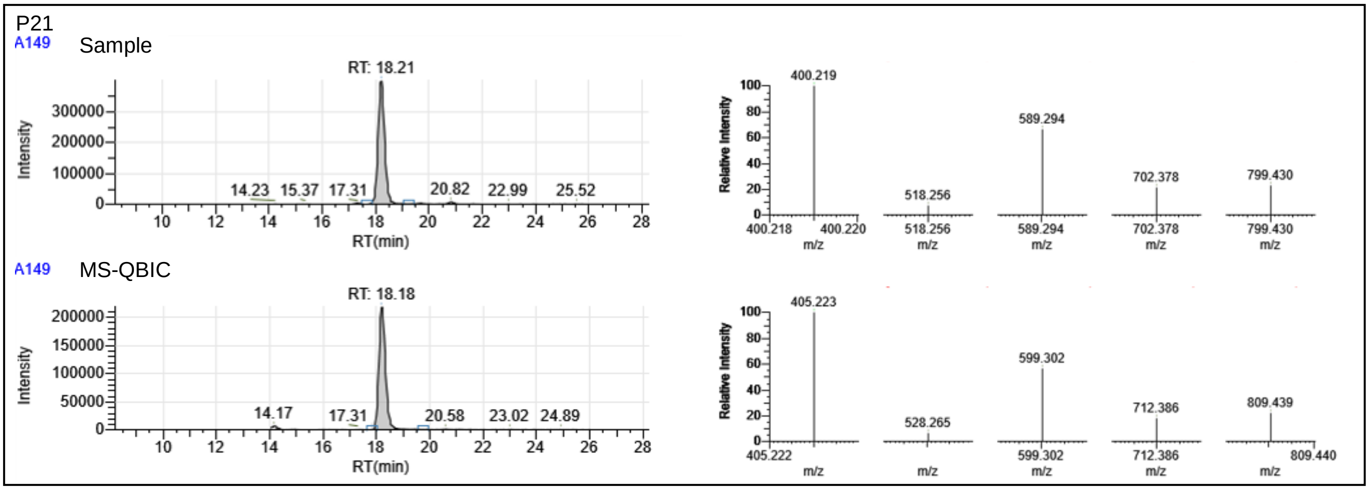
P21
A149 Sample
RT: 18.21
14.23 15.37 17.31 20.82 22.99 25.52
RT(min)
Intensity
400.219
589.294
518.256
702.378
799.430
Relative Intensity
m/z
A149 MS-QBIC
RT: 18.18
14.17 17.31 20.58 23.02 24.89
405.223
599.302
528.265
712.386
809.439

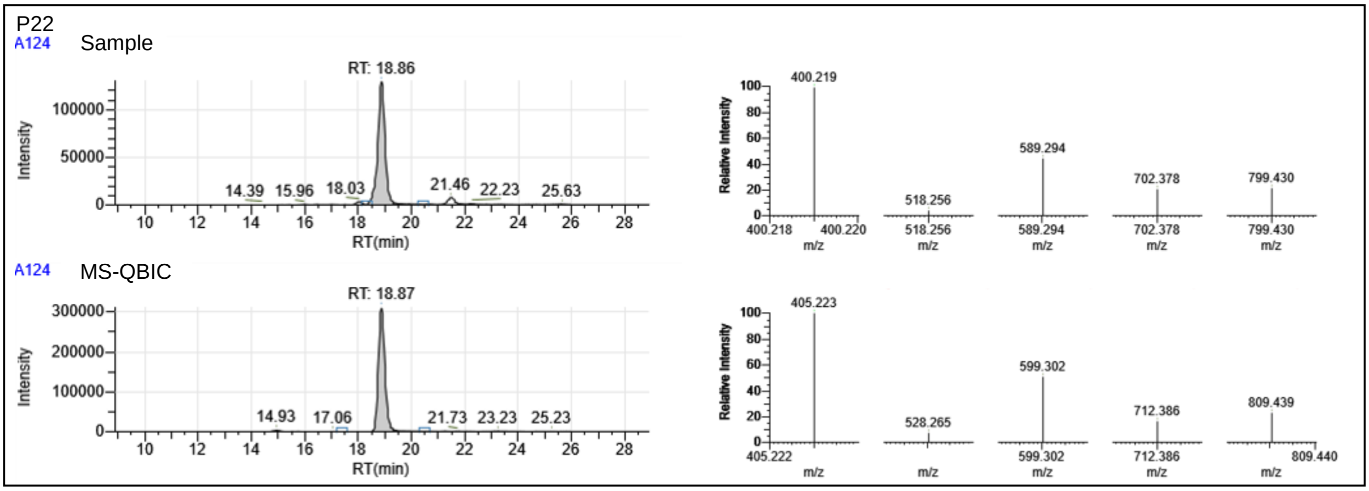
P22
A124 Sample
RT: 18.86
14.39 15.96 18.03 21.46 22.23 25.63
RT(min)
Intensity
400.219
589.294
518.256
702.378
799.430
Relative Intensity
m/z
A124 MS-QBIC
RT: 18.87
14.93 17.06 21.73 23.23 25.23
405.223
599.302
528.265
712.386
809.439

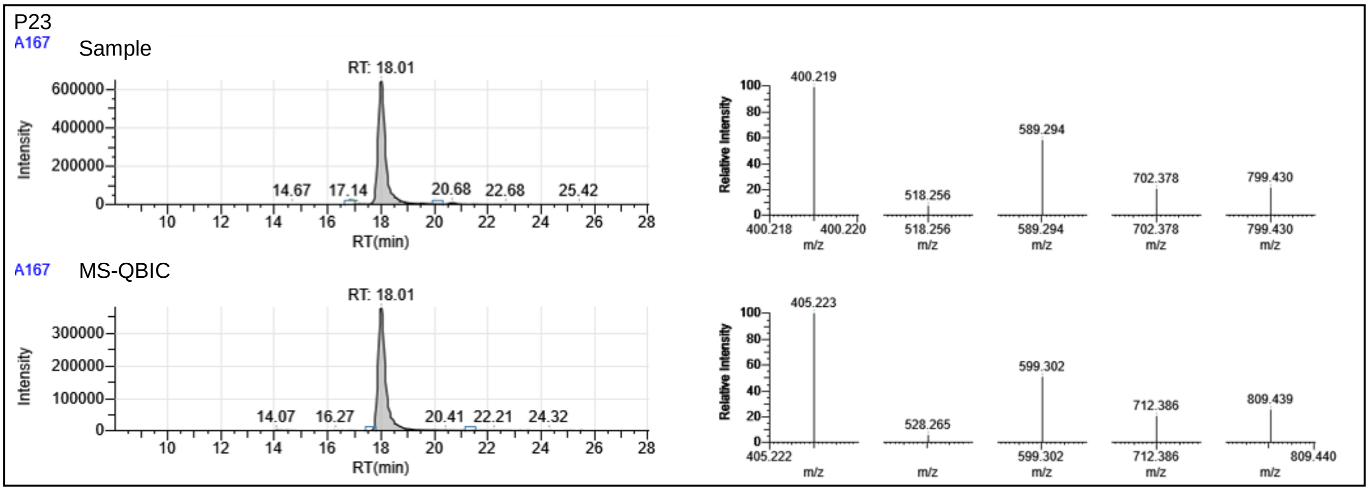
P23
A167 Sample
RT: 18.01
Intensity
600000
400000
200000
0
14.67 17.14 20.68 22.68 25.42
10 12 14 16 18 20 22 24 26 28
RT(min)
Relative Intensity
400.219
518.256
589.294
702.378
799.430
400.218 400.220
m/z
A167 MS-QBIC
RT: 18.01
300000
200000
100000
0
14.07 16.27 20.41 22.21 24.32
405.223
528.265
599.302
712.386
809.439
405.222
809.440

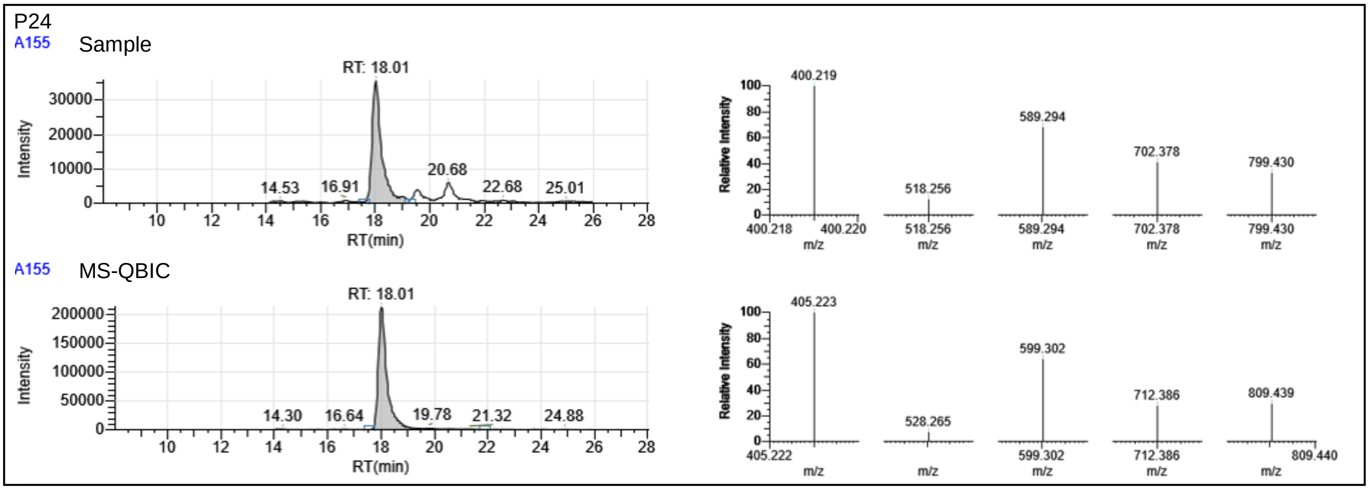
P24
A155 Sample
RT: 18.01
Intensity
30000
20000
10000
0
14.53 16.91 20.68 22.68 25.01
10 12 14 16 18 20 22 24 26 28
RT(min)
Relative Intensity
400.219
518.256
589.294
702.378
799.430
400.218 400.220
m/z
A155 MS-QBIC
RT: 18.01
200000
150000
100000
50000
0
14.30 16.64 19.78 21.32 24.88
405.223
528.265
599.302
712.386
809.439
405.222
809.440

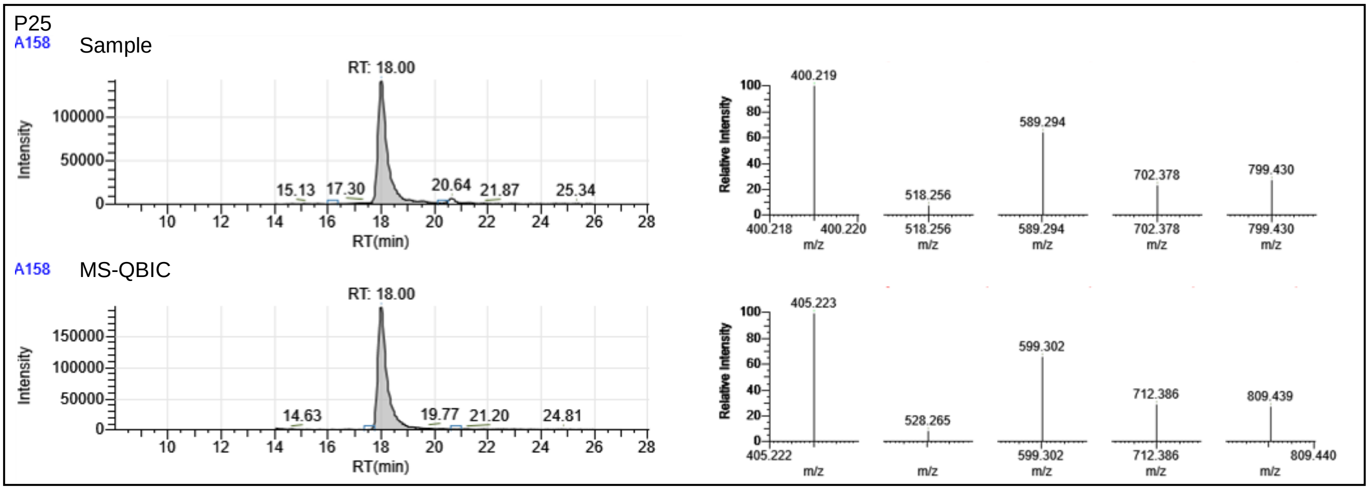
P25
A158 Sample
RT: 18.00
Intensity
100000
50000
0
15.13 17.30 20.64 21.87 25.34
10 12 14 16 18 20 22 24 26 28
RT(min)
Relative Intensity
400.219
518.256
589.294
702.378
799.430
400.218 400.220
m/z
A158 MS-QBIC
RT: 18.00
150000
100000
50000
0
14.63 19.77 21.20 24.81
405.223
528.265
599.302
712.386
809.439
405.222
809.440

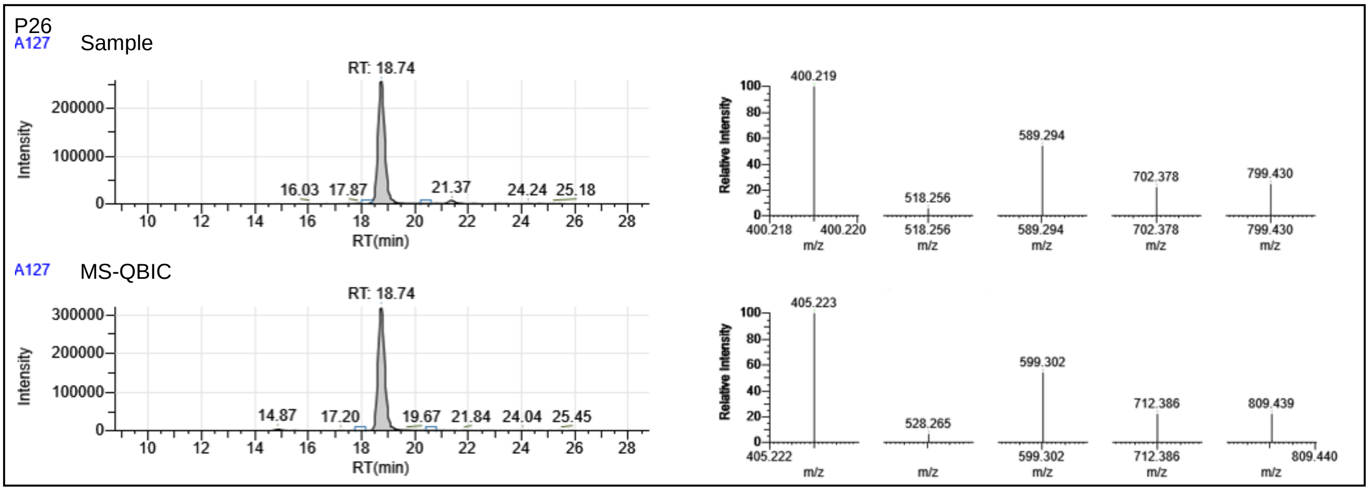
P26
A127 Sample
RT: 18.74
Intensity
200000
100000
0
16.03 17.87 21.37 24.24 25.18
10 12 14 16 18 20 22 24 26 28
RT(min)
Relative Intensity
400.219
518.256
589.294
702.378
799.430
400.218 400.220
m/z
A127 MS-QBIC
RT: 18.74
300000
200000
100000
0
14.87 17.20 19.67 21.84 24.04 25.45
405.223
528.265
599.302
712.386
809.439
405.222
809.440

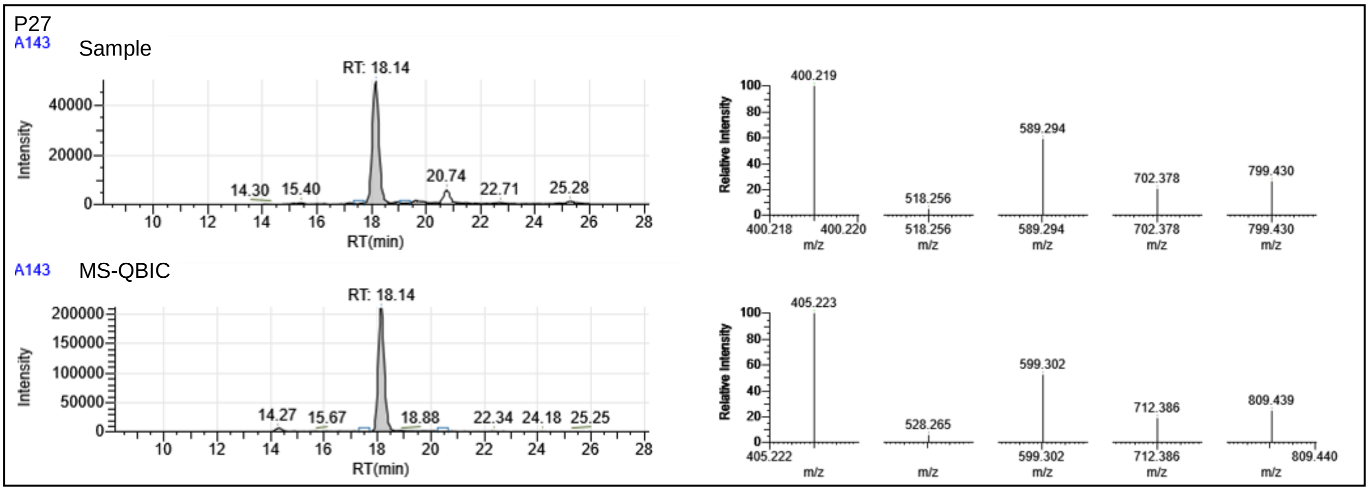

P27
A143 Sample
RT: 18.14
14.30 15.40 20.74 22.71 25.28
Intensity
RT(min)
400.219 518.256 589.294 702.378 799.430
Relative Intensity
m/z
A143 MS-QBIC
RT: 18.14
14.27 15.67 18.88 22.34 24.18 25.25
405.223 528.265 599.302 712.386 809.439

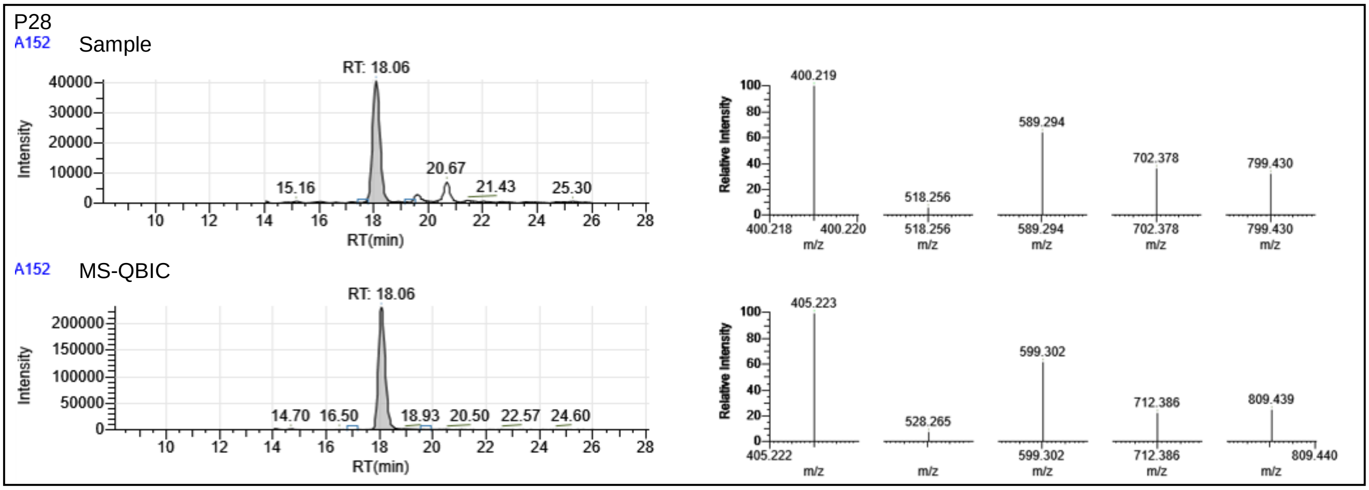

P28
A152 Sample
RT: 18.06
15.16 20.67 21.43 25.30
Intensity
RT(min)
400.219 518.256 589.294 702.378 799.430
Relative Intensity
m/z
A152 MS-QBIC
RT: 18.06
14.70 16.50 18.93 20.50 22.57 24.60
405.223 528.265 599.302 712.386 809.439

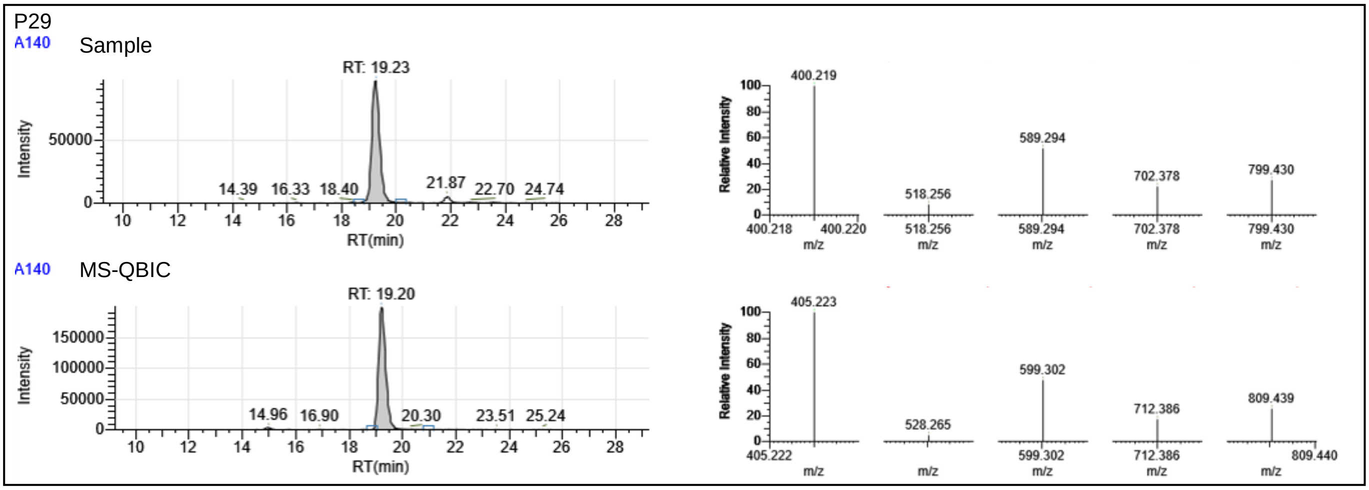

P29
A140 Sample
RT: 19.23
14.39 16.33 18.40 21.87 22.70 24.74
Intensity
RT(min)
400.219 518.256 589.294 702.378 799.430
Relative Intensity
m/z
A140 MS-QBIC
RT: 19.20
14.96 16.90 20.30 23.51 25.24
405.223 528.265 599.302 712.386 809.439

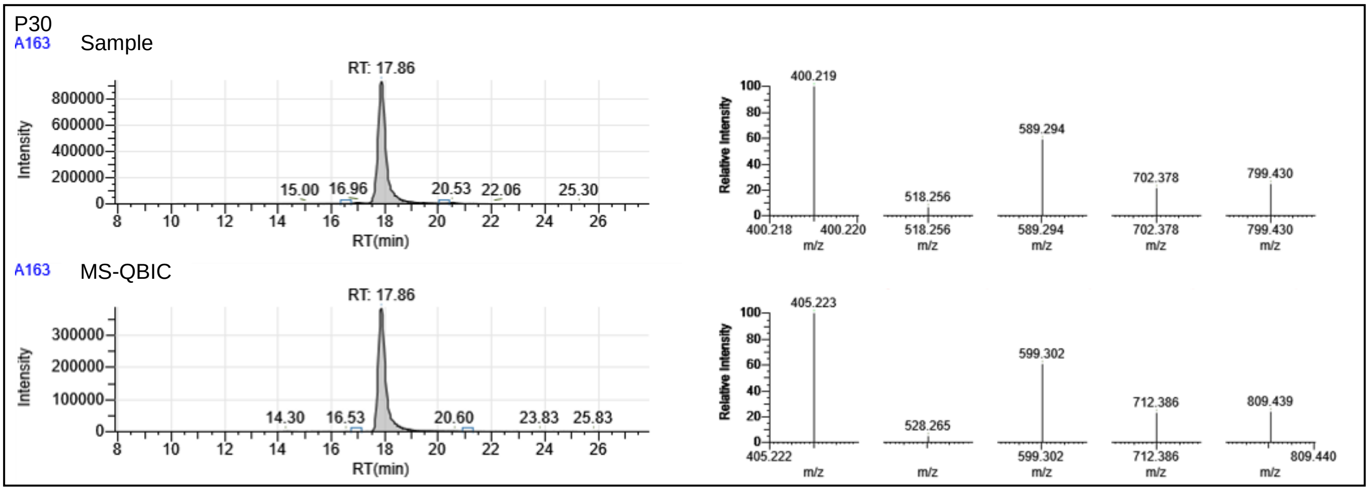

P30
A163 Sample
RT: 17.86
15.00 16.96 20.53 22.06 25.30
Intensity
RT(min)
400.219 518.256 589.294 702.378 799.430
Relative Intensity
m/z
A163 MS-QBIC
RT: 17.86
14.30 16.53 20.60 23.83 25.83
405.223 528.265 599.302 712.386 809.439

U Apo E (LGPLVEQGR) Another organ

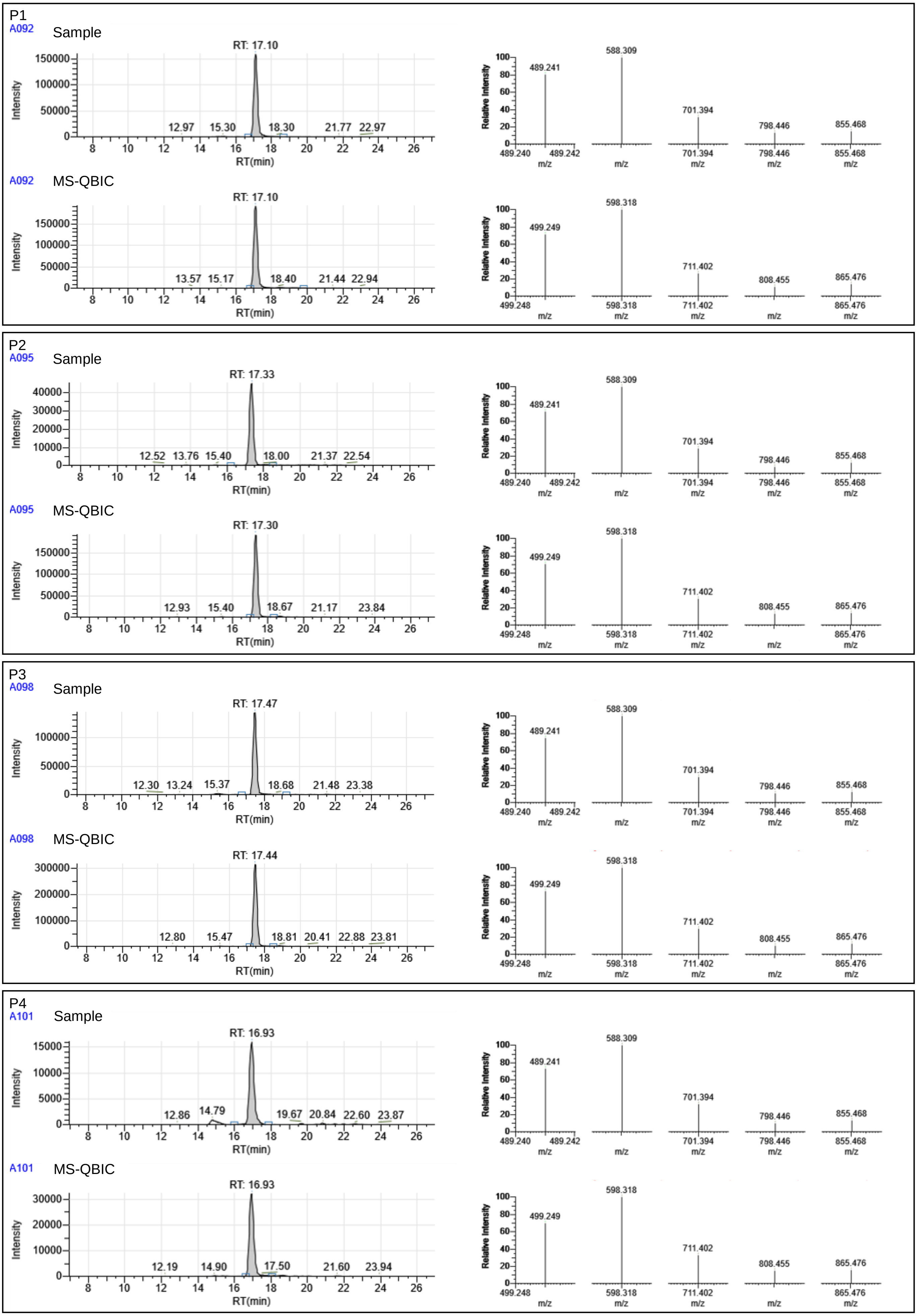

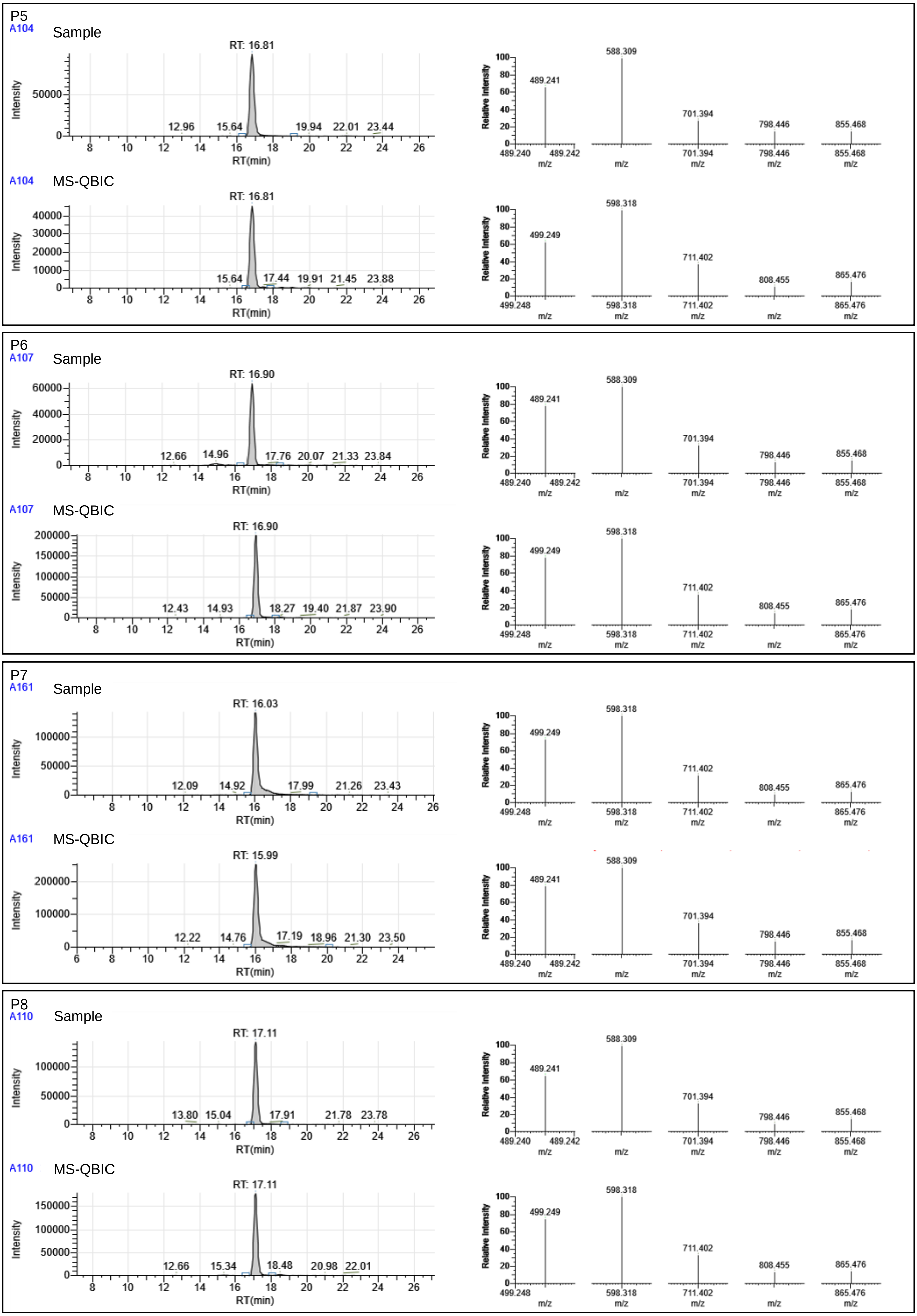
P5
A104 Sample
RT: 16.81
12.96 15.64 19.94 22.01 23.44
Intensity
RT(min)
489.241 588.309 701.394 798.446 855.468
Relative Intensity
m/z
A104 MS-QBIC
RT: 16.81
15.64 17.44 19.91 21.45 23.88
499.249 598.318 711.402 808.455 865.476
P6
A107 Sample
RT: 16.90
12.66 14.96 17.76 20.07 21.33 23.84
489.241 588.309 701.394 798.446 855.468
A107 MS-QBIC
RT: 16.90
12.43 14.93 18.27 19.40 21.87 23.90
499.249 598.318 711.402 808.455 865.476
P7
A161 Sample
RT: 16.03
12.09 14.92 17.99 21.26 23.43
499.249 598.318 711.402 808.455 865.476
A161 MS-QBIC
RT: 15.99
12.22 14.76 17.19 18.96 21.30 23.50
489.241 588.309 701.394 798.446 855.468
P8
A110 Sample
RT: 17.11
13.80 15.04 17.91 21.78 23.78
489.241 588.309 701.394 798.446 855.468
A110 MS-QBIC
RT: 17.11
12.66 15.34 18.48 20.98 22.01
499.249 598.318 711.402 808.455 865.476

P9

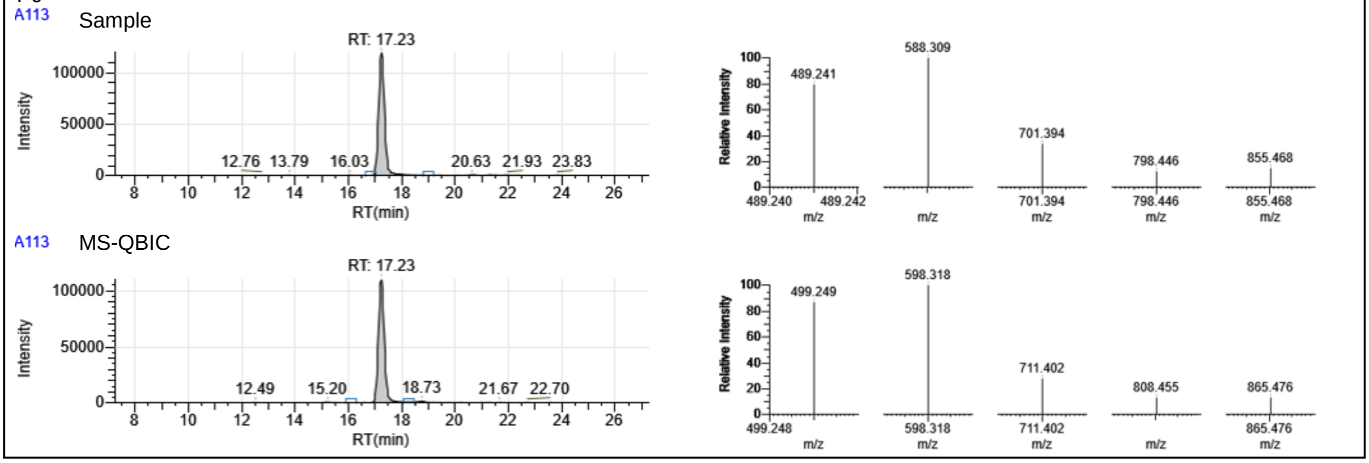

P10

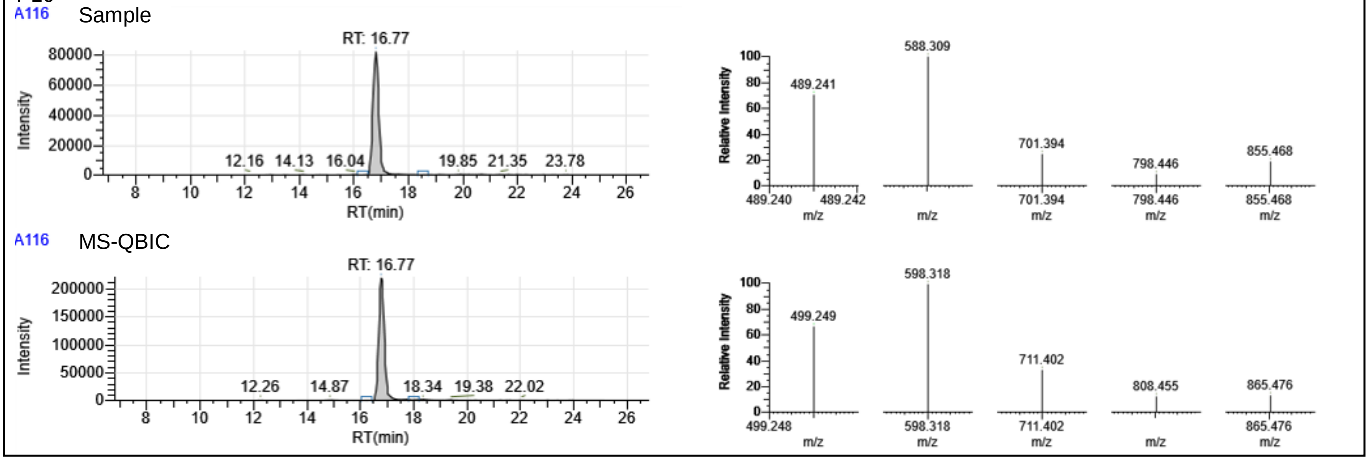

P13

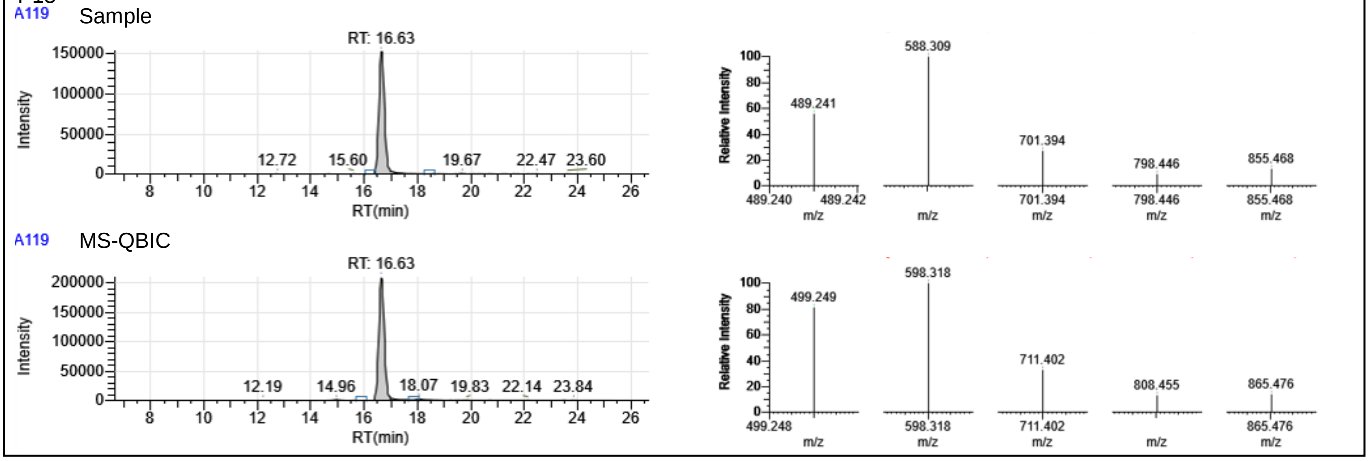

P14

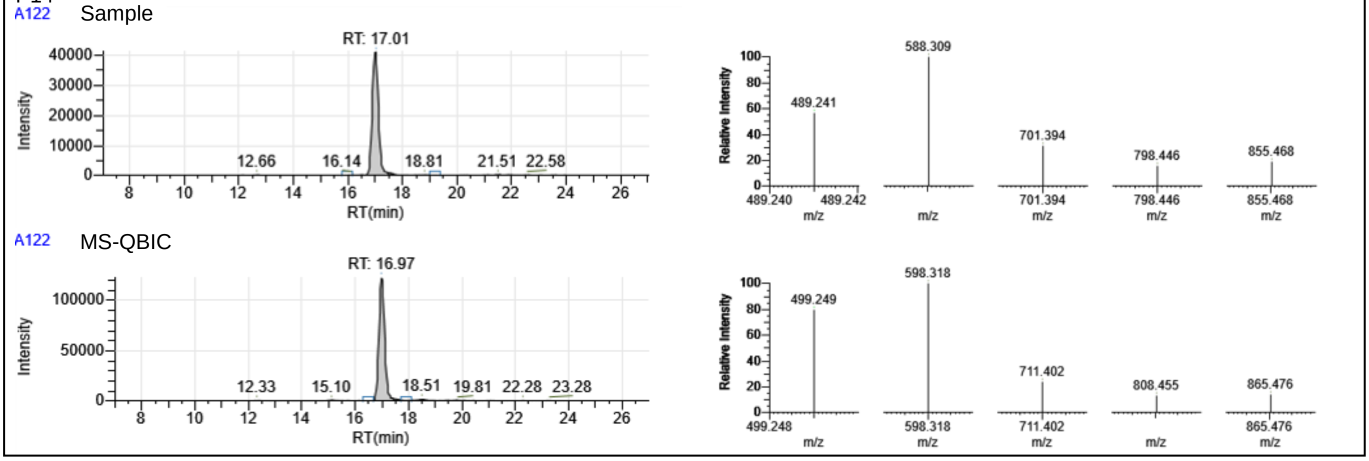

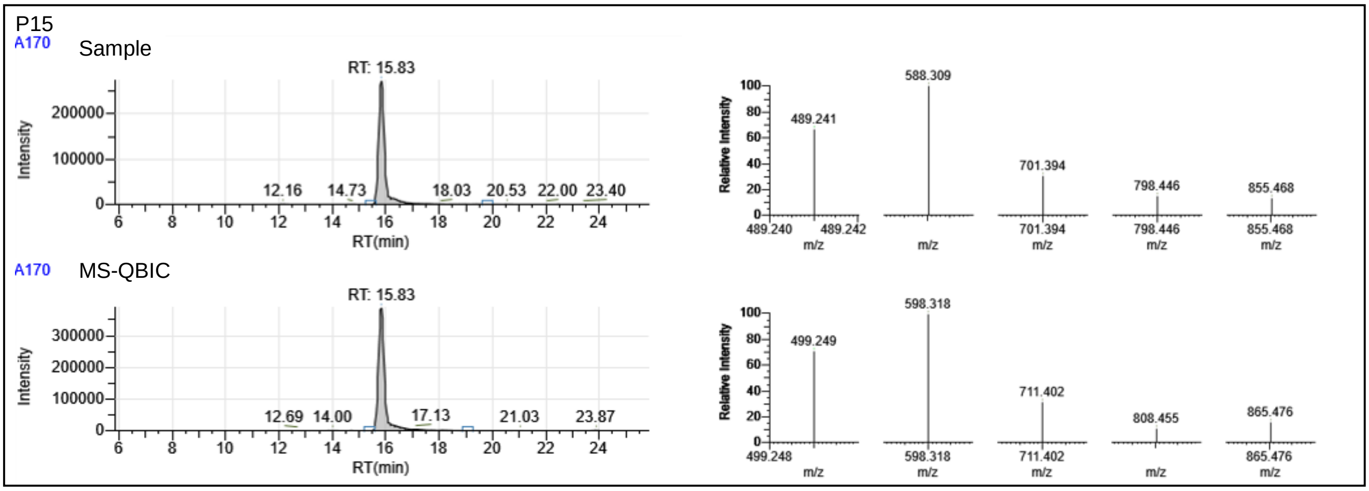
P15
A170 Sample
RT: 15.83
12.16 14.73 18.03 20.53 22.00 23.40
Intensity
RT(min)
Relative Intensity
489.241 588.309 701.394 798.446 855.468
m/z
A170 MS-QBIC
RT: 15.83
12.69 14.00 17.13 21.03 23.87
499.249 598.318 711.402 808.455 865.476

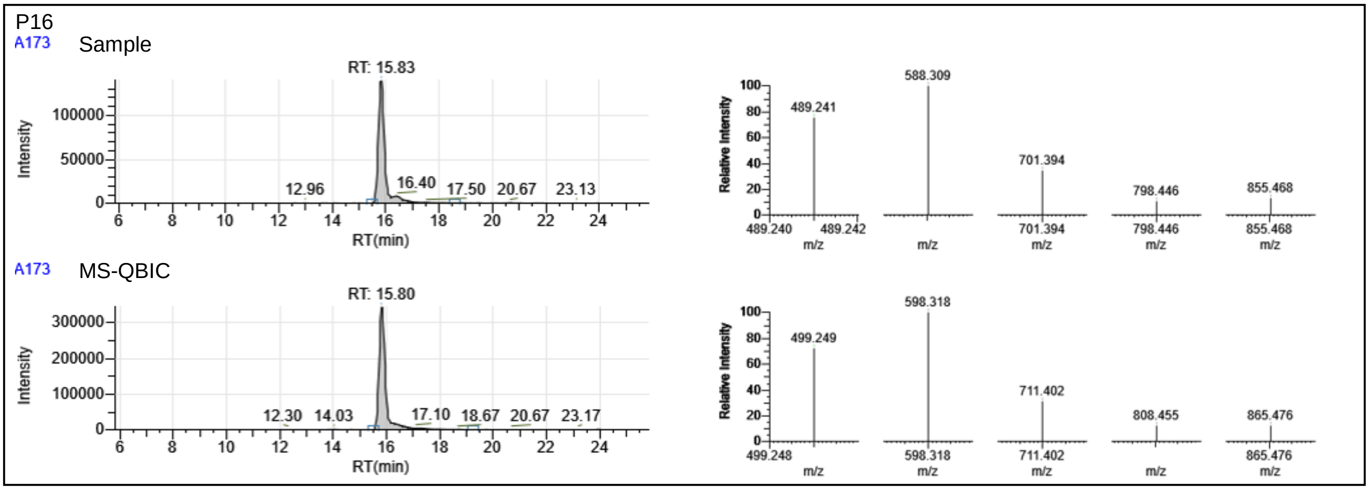
P16
A173 Sample
RT: 15.83
12.96 16.40 17.50 20.67 23.13
Intensity
RT(min)
Relative Intensity
489.241 588.309 701.394 798.446 855.468
m/z
A173 MS-QBIC
RT: 15.80
12.30 14.03 17.10 18.67 20.67 23.17
499.249 598.318 711.402 808.455 865.476

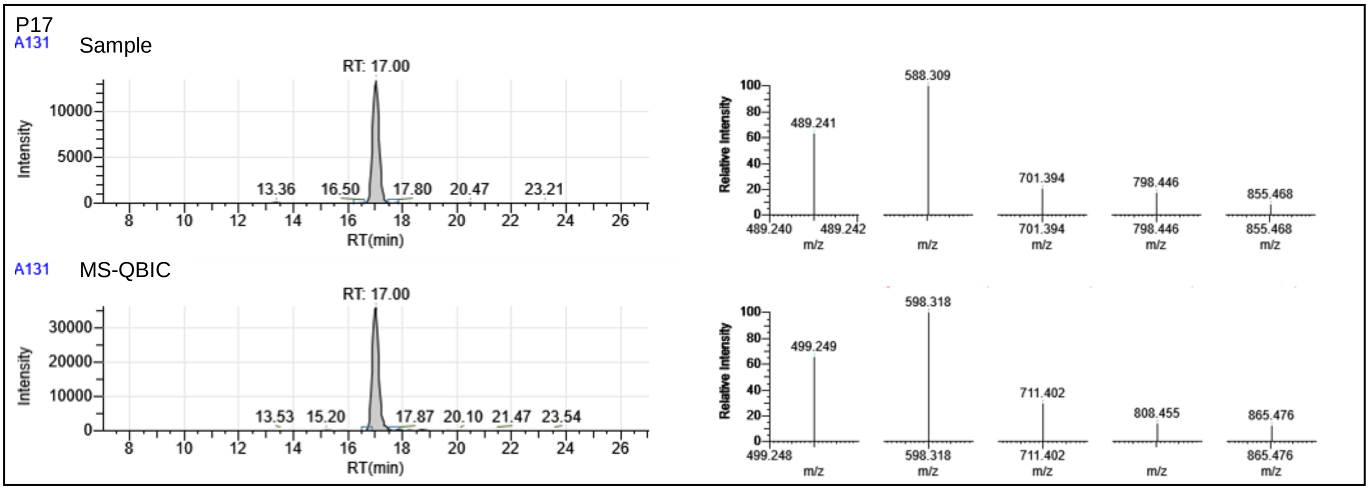
P17
A131 Sample
RT: 17.00
13.36 16.50 17.80 20.47 23.21
Intensity
RT(min)
Relative Intensity
489.241 588.309 701.394 798.446 855.468
m/z
A131 MS-QBIC
RT: 17.00
13.53 15.20 17.87 20.10 21.47 23.54
499.249 598.318 711.402 808.455 865.476

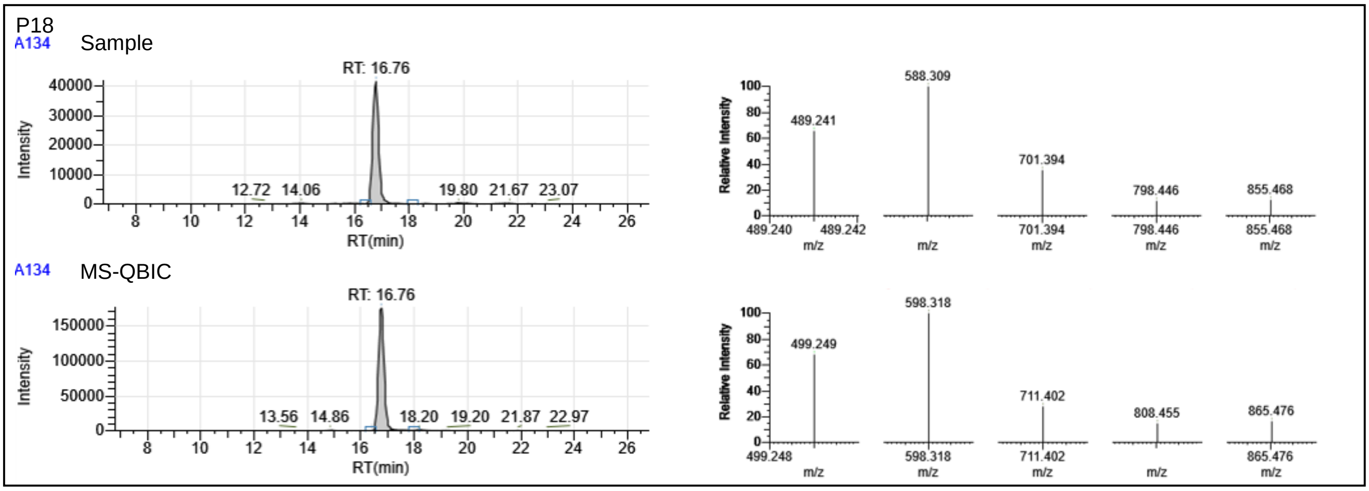
P18
A134 Sample
RT: 16.76
12.72 14.06 19.80 21.67 23.07
Intensity
RT(min)
Relative Intensity
489.241 588.309 701.394 798.446 855.468
m/z
A134 MS-QBIC
RT: 16.76
13.56 14.86 18.20 19.20 21.87 22.97
499.249 598.318 711.402 808.455 865.476

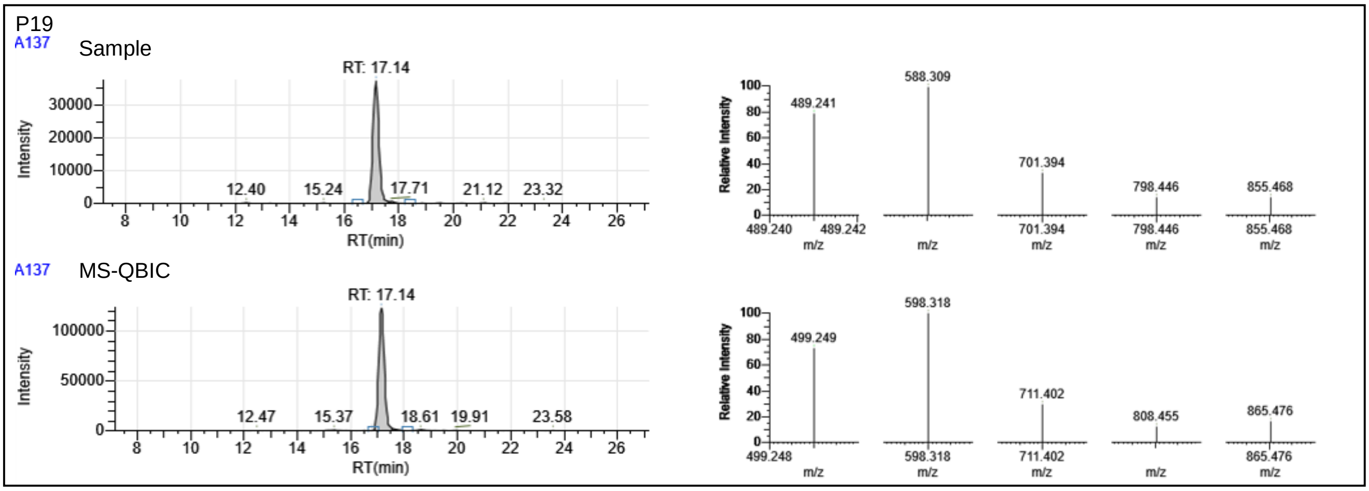
P19
A137 Sample
RT: 17.14
12.40 15.24 17.71 21.12 23.32
Intensity
RT(min)
489.241 588.309 701.394 798.446 855.468
Relative Intensity
m/z
A137 MS-QBIC
RT: 17.14
12.47 15.37 18.61 19.91 23.58
499.249 598.318 711.402 808.455 865.476

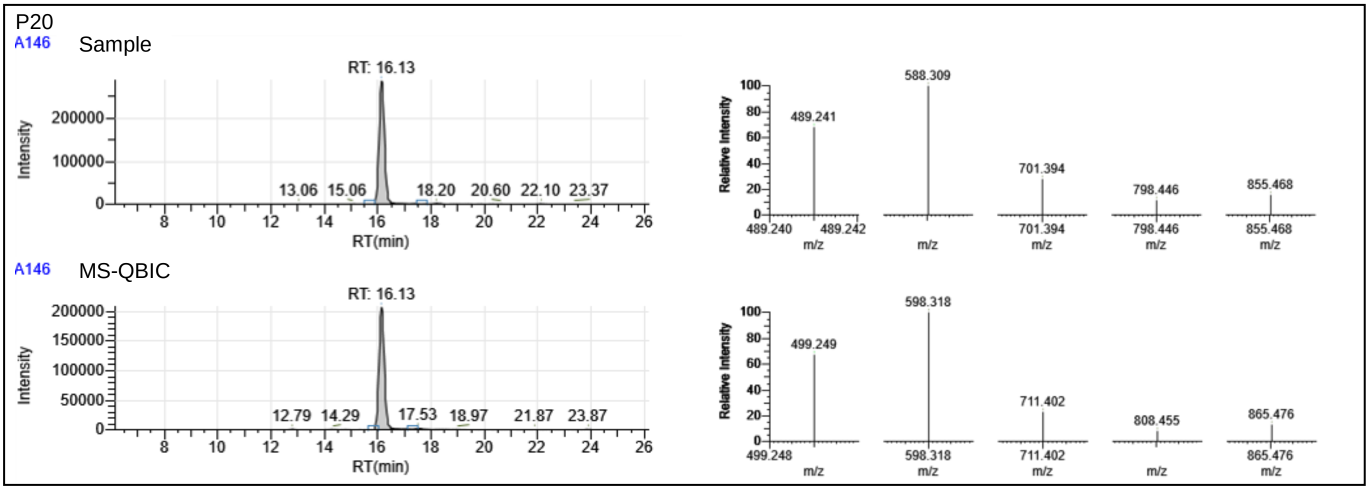
P20
A146 Sample
RT: 16.13
13.06 15.06 18.20 20.60 22.10 23.37
Intensity
RT(min)
489.241 588.309 701.394 798.446 855.468
Relative Intensity
m/z
A146 MS-QBIC
RT: 16.13
12.79 14.29 17.53 18.97 21.87 23.87
499.249 598.318 711.402 808.455 865.476

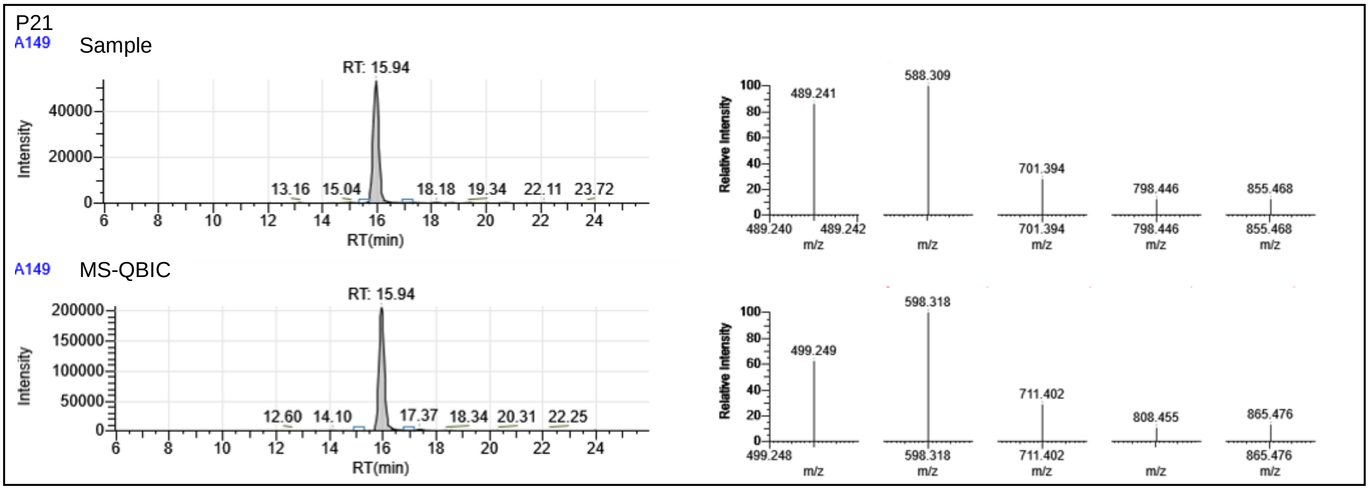
P21
A149 Sample
RT: 15.94
13.16 15.04 18.18 19.34 22.11 23.72
Intensity
RT(min)
489.241 588.309 701.394 798.446 855.468
Relative Intensity
m/z
A149 MS-QBIC
RT: 15.94
12.60 14.10 17.37 18.34 20.31 22.25
499.249 598.318 711.402 808.455 865.476

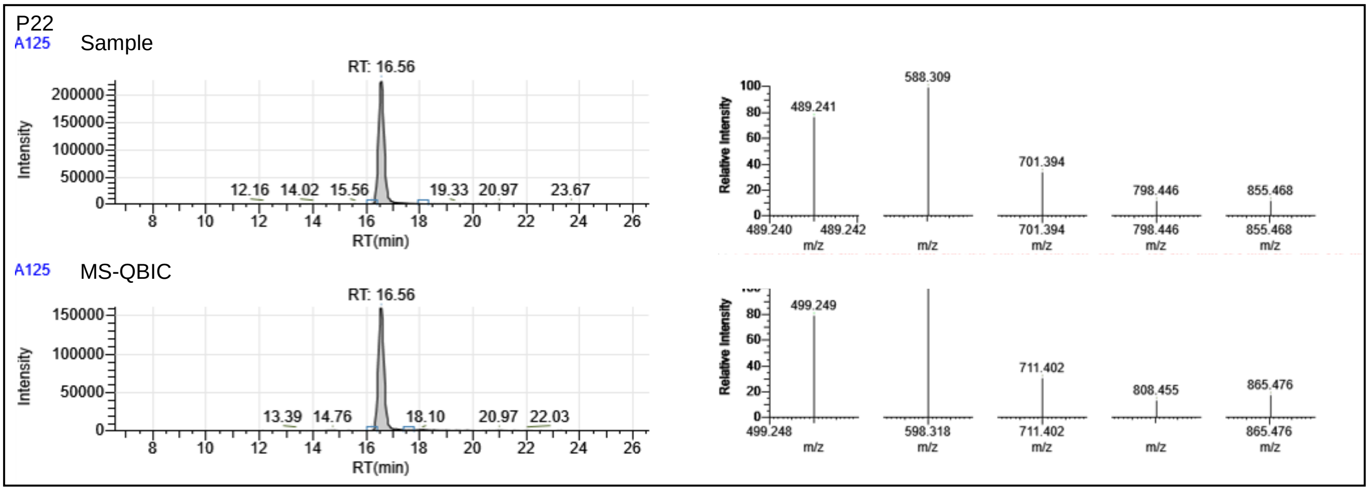
P22
A125 Sample
RT: 16.56
12.16 14.02 15.56 19.33 20.97 23.67
Intensity
RT(min)
489.241 588.309 701.394 798.446 855.468
Relative Intensity
m/z
A125 MS-QBIC
RT: 16.56
13.39 14.76 18.10 20.97 22.03
499.249 711.402 808.455 865.476
598.318

P23

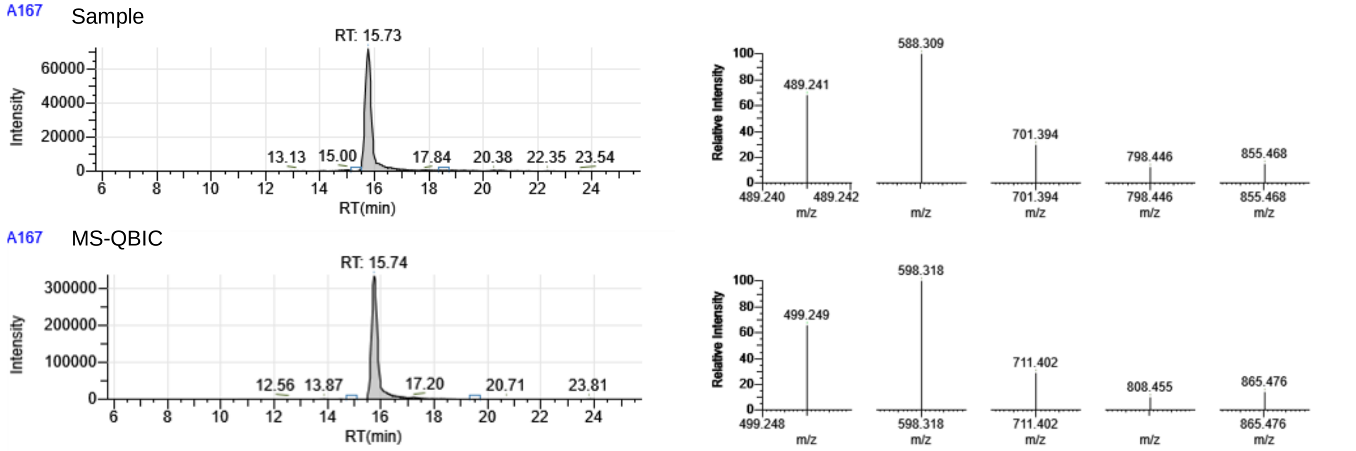

P24

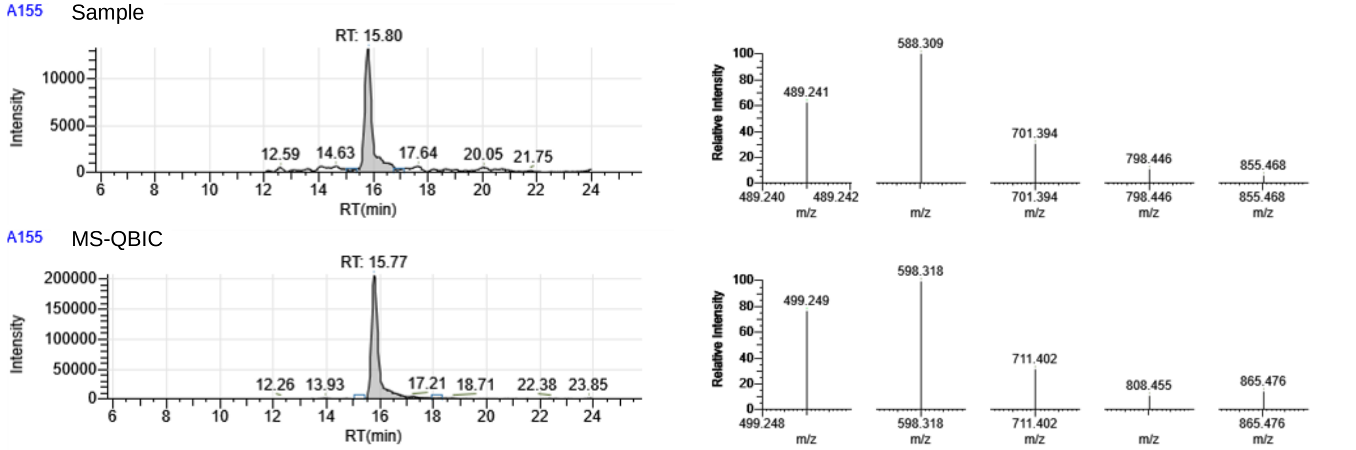

P26

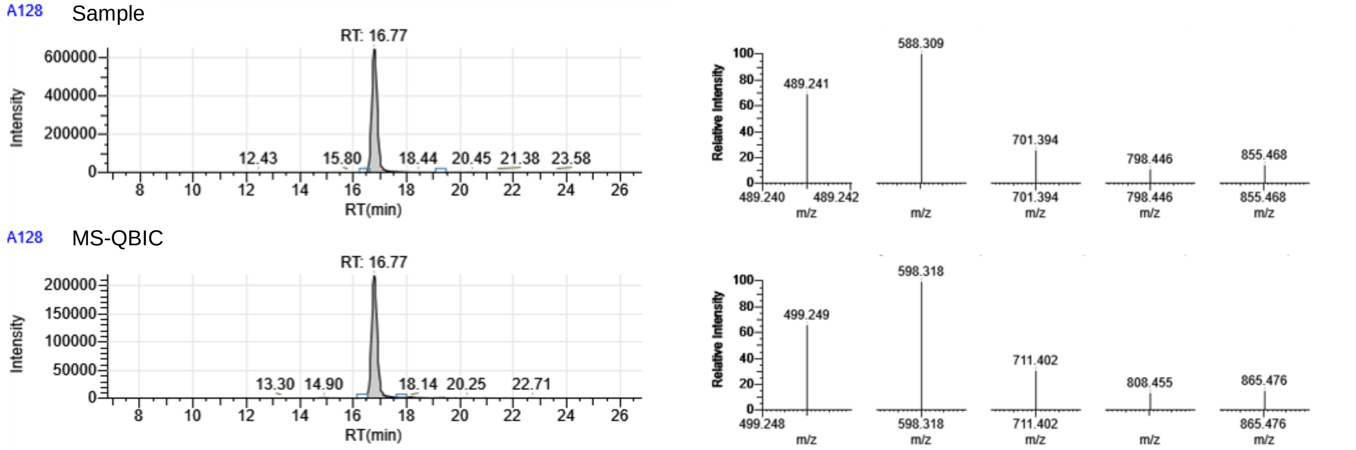

P27

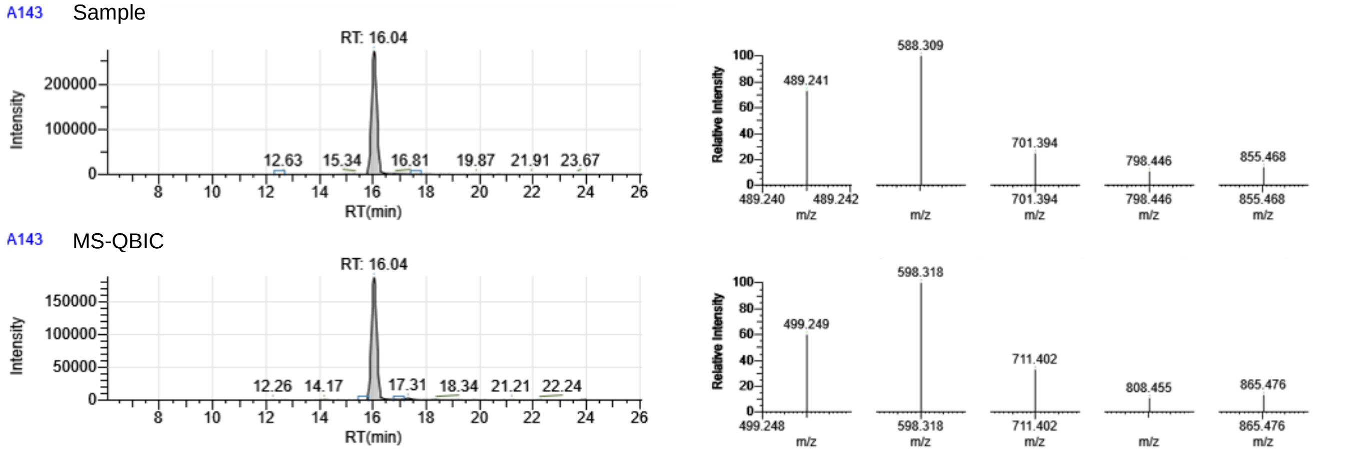

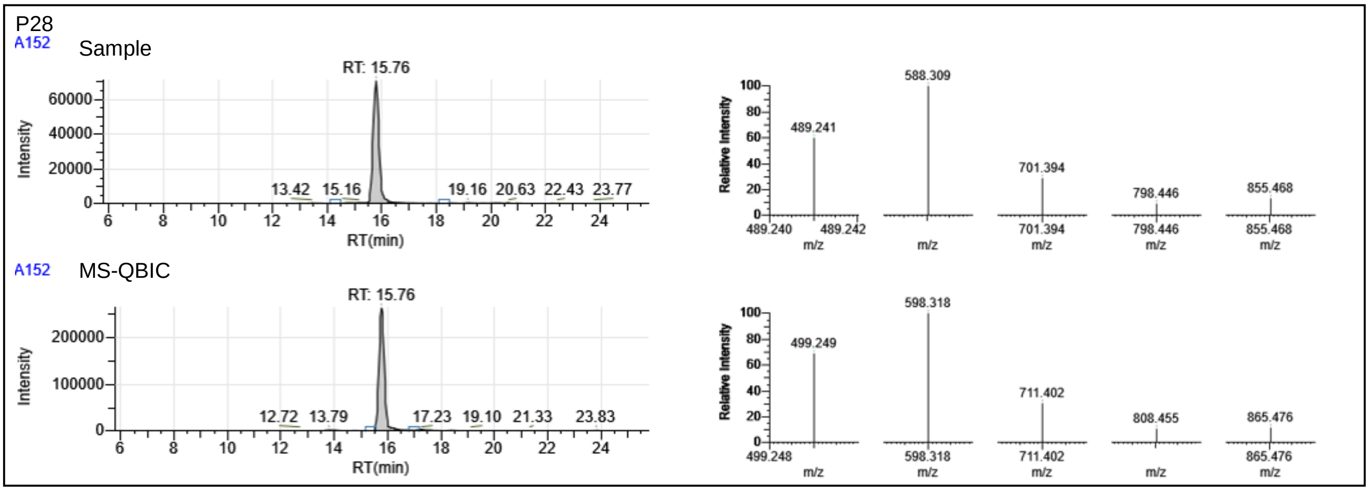
P28
A152 Sample
RT: 15.76
13.42 15.16 19.16 20.63 22.43 23.77
Intensity
RT(min)
Relative Intensity
489.241 588.309 701.394 798.446 855.468
m/z
A152 MS-QBIC
RT: 15.76
12.72 13.79 17.23 19.10 21.33 23.83
499.249 598.318 711.402 808.455 865.476

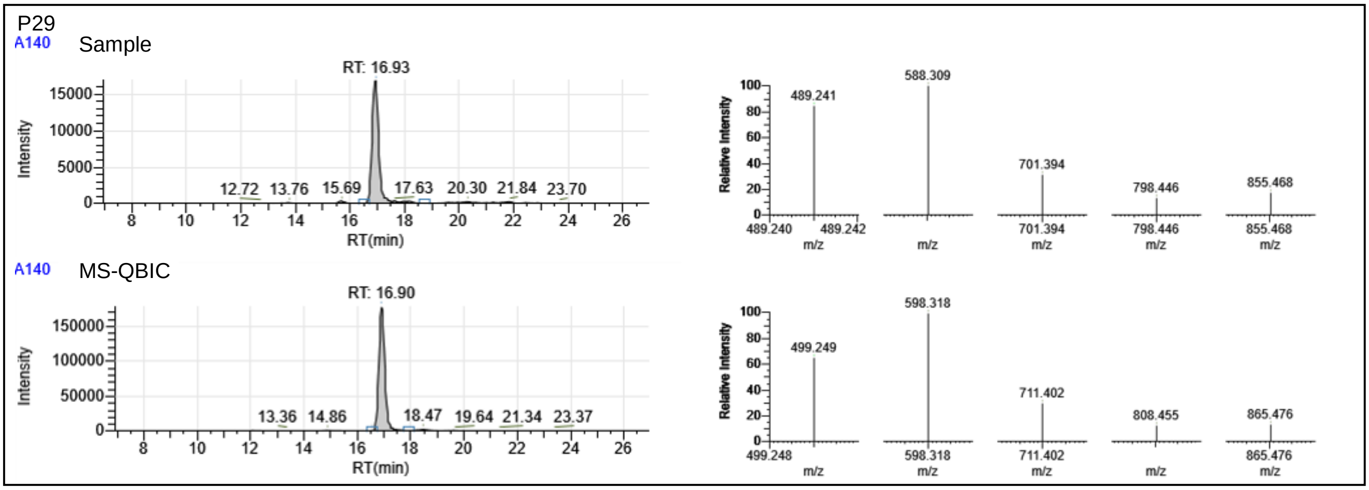
P29
A140 Sample
RT: 16.93
12.72 13.76 15.69 17.63 20.30 21.84 23.70
Intensity
RT(min)
Relative Intensity
489.241 588.309 701.394 798.446 855.468
m/z
A140 MS-QBIC
RT: 16.90
13.36 14.86 18.47 19.64 21.34 23.37
499.249 598.318 711.402 808.455 865.476

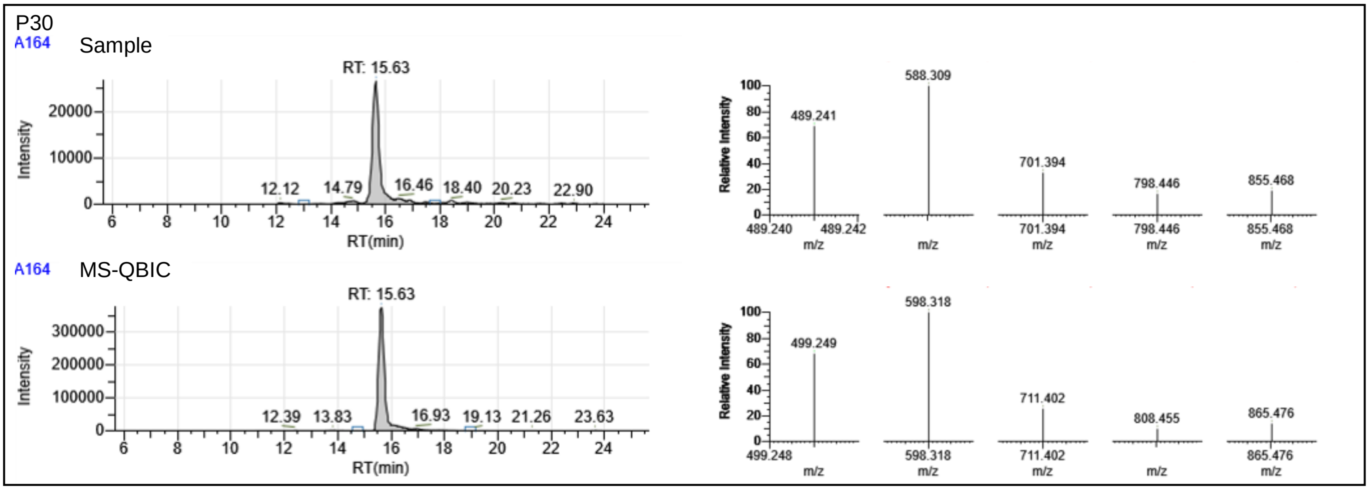
P30
A164 Sample
RT: 15.63
12.12 14.79 16.46 18.40 20.23 22.90
Intensity
RT(min)
Relative Intensity
489.241 588.309 701.394 798.446 855.468
m/z
A164 MS-QBIC
RT: 15.63
12.39 13.83 16.93 19.13 21.26 23.63
499.249 598.318 711.402 808.455 865.476

V Gelsolin (EPGLQIWR) Another organ

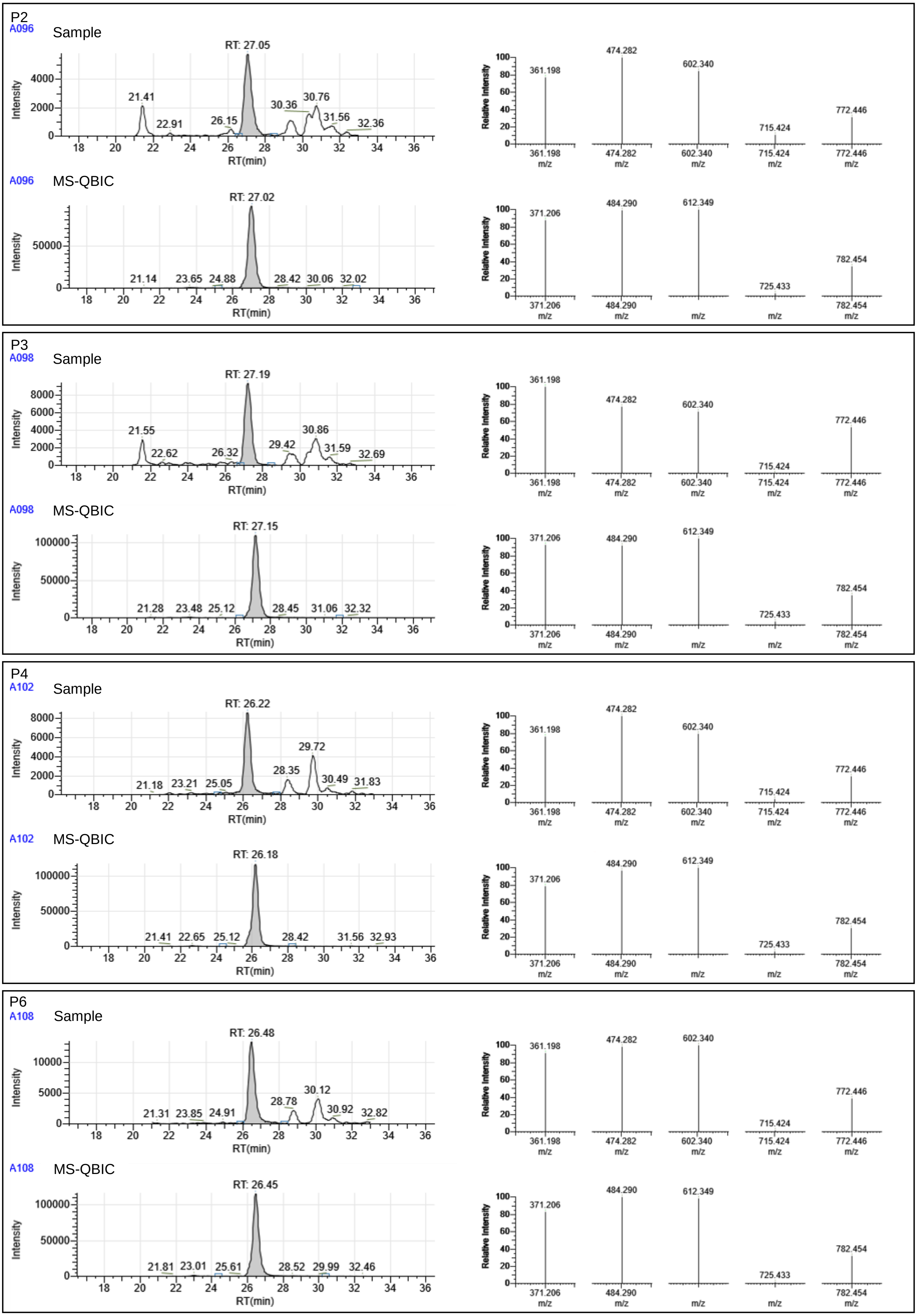

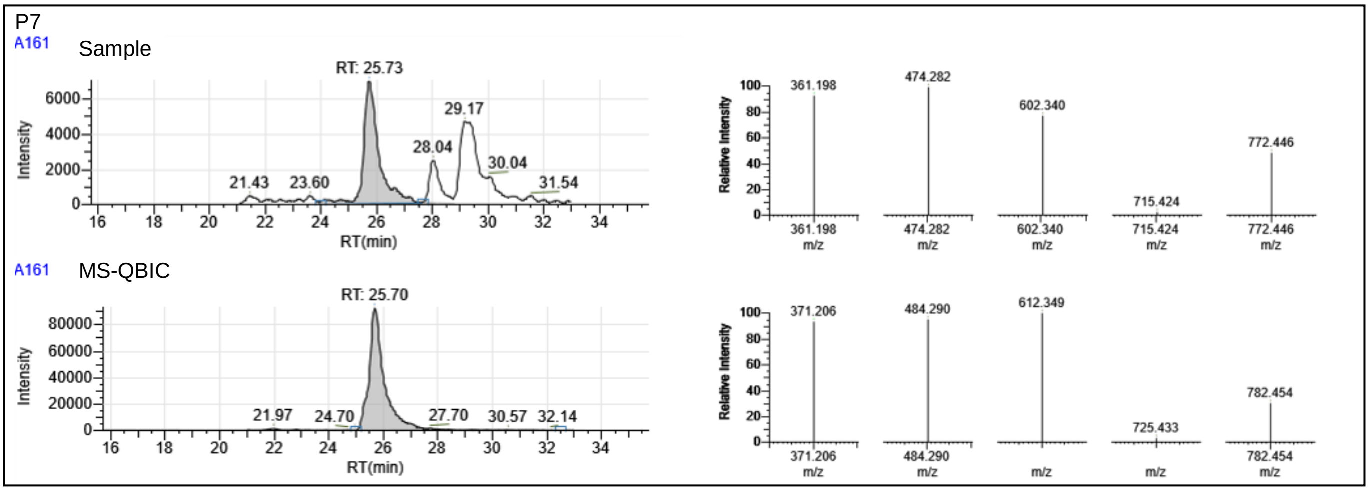
P7
A161 Sample
RT: 25.73
21.43 23.60 28.04 29.17 30.04 31.54
Intensity
RT(min)
361.198
474.282
602.340
715.424
772.446
Relative Intensity
m/z
A161 MS-QBIC
RT: 25.70
21.97 24.70 27.70 30.57 32.14
371.206
484.290
612.349
725.433
782.454

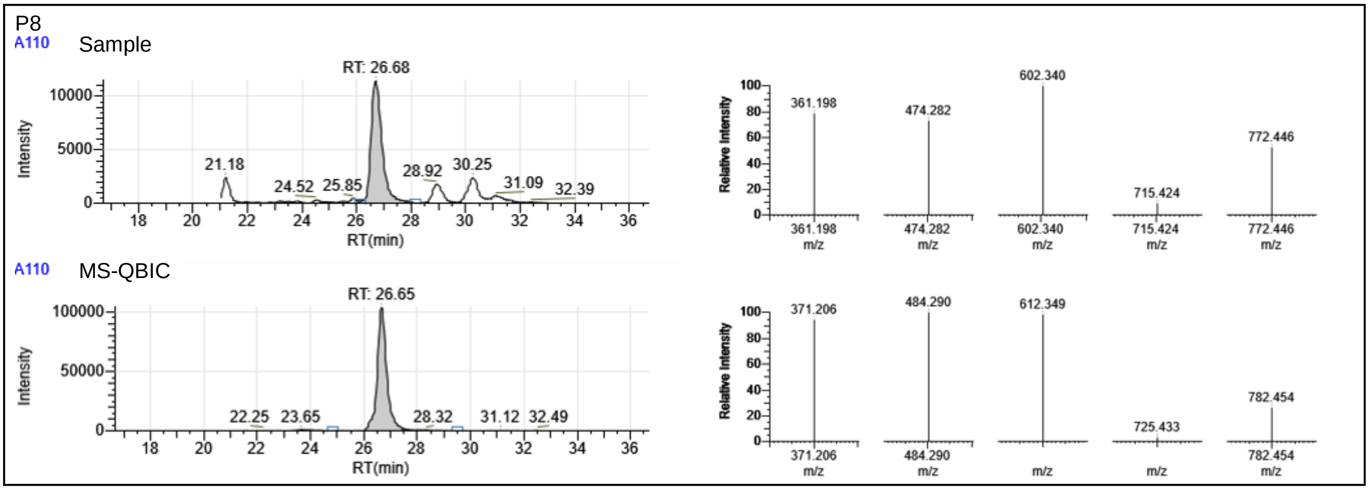
P8
A110 Sample
RT: 26.68
21.18 24.52 25.85 28.92 30.25 31.09 32.39
Intensity
RT(min)
361.198
474.282
602.340
715.424
772.446
Relative Intensity
m/z
A110 MS-QBIC
RT: 26.65
22.25 23.65 28.32 31.12 32.49
371.206
484.290
612.349
725.433
782.454

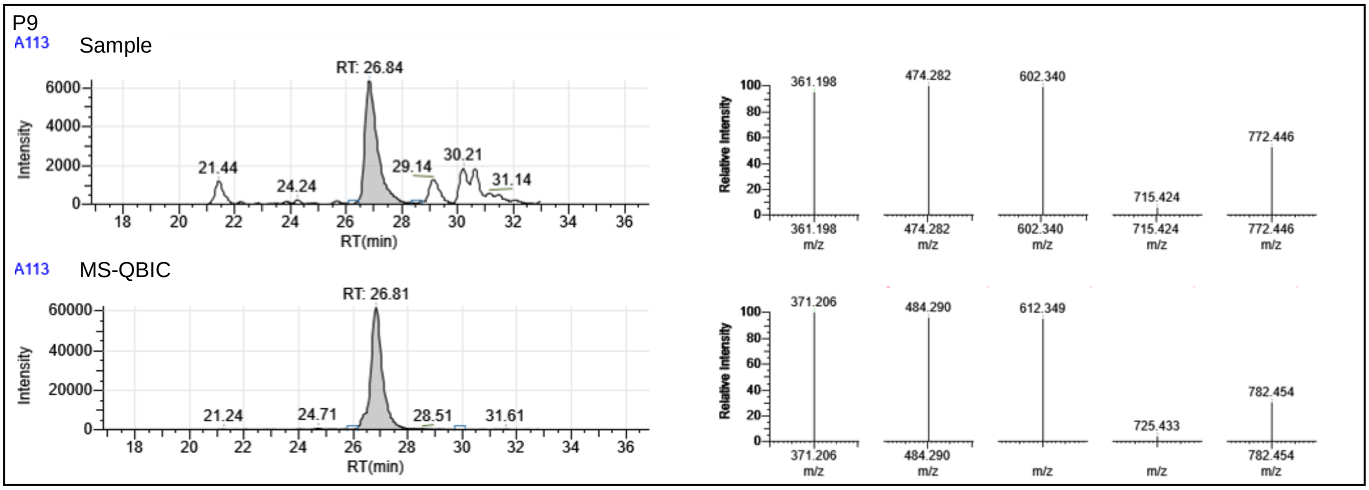
P9
A113 Sample
RT: 26.84
21.44 24.24 29.14 30.21 31.14
Intensity
RT(min)
361.198
474.282
602.340
715.424
772.446
Relative Intensity
m/z
A113 MS-QBIC
RT: 26.81
21.24 24.71 28.51 31.61
371.206
484.290
612.349
725.433
782.454

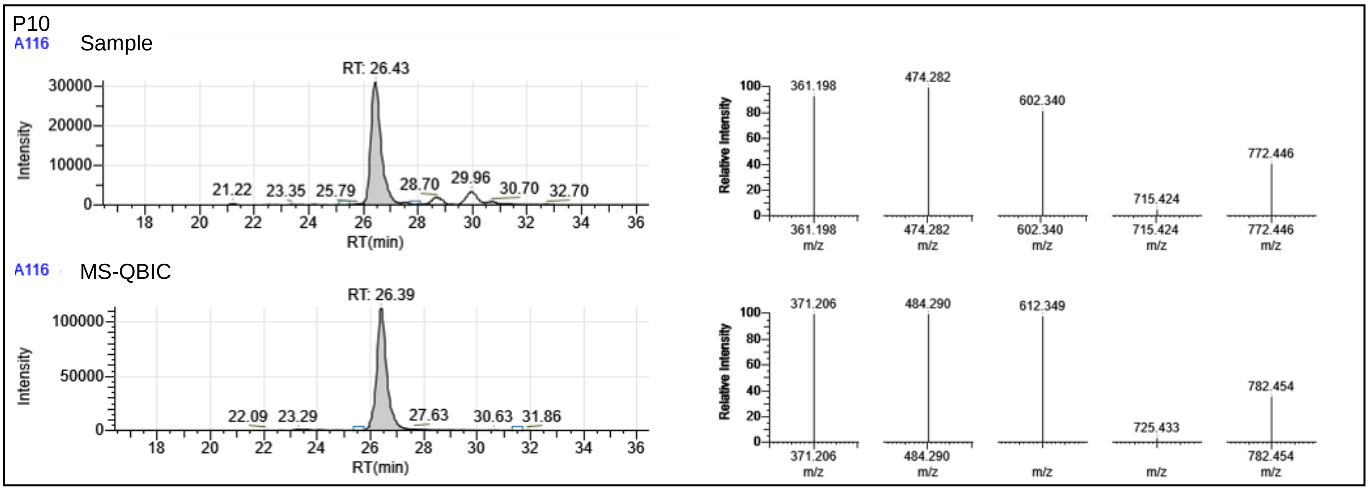
P10
A116 Sample
RT: 26.43
21.22 23.35 25.79 28.70 29.96 30.70 32.70
Intensity
RT(min)
361.198
474.282
602.340
715.424
772.446
Relative Intensity
m/z
A116 MS-QBIC
RT: 26.39
22.09 23.29 27.63 30.63 31.86
371.206
484.290
612.349
725.433
782.454

P13

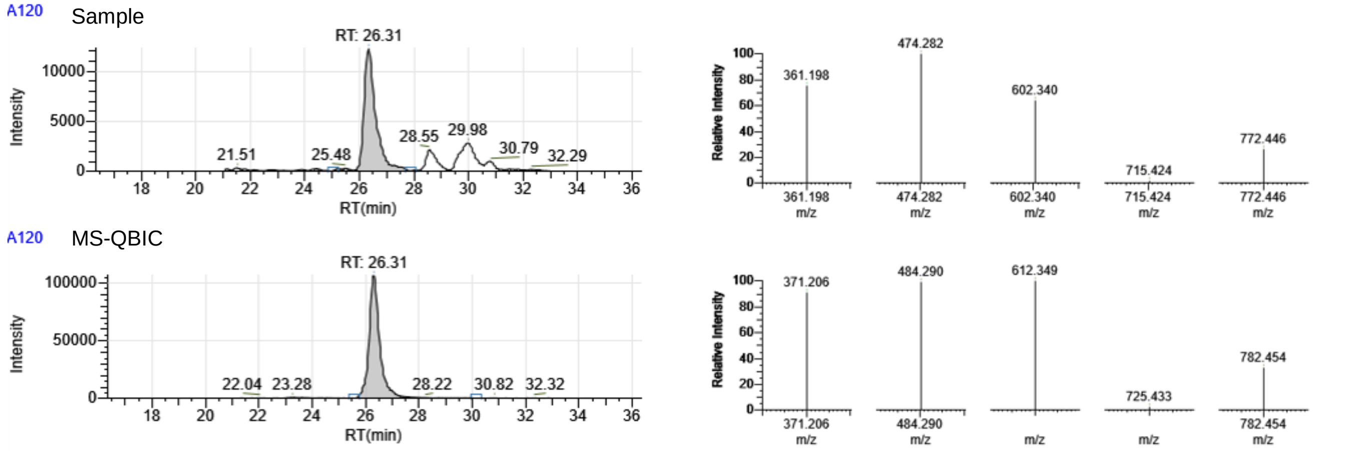

P14

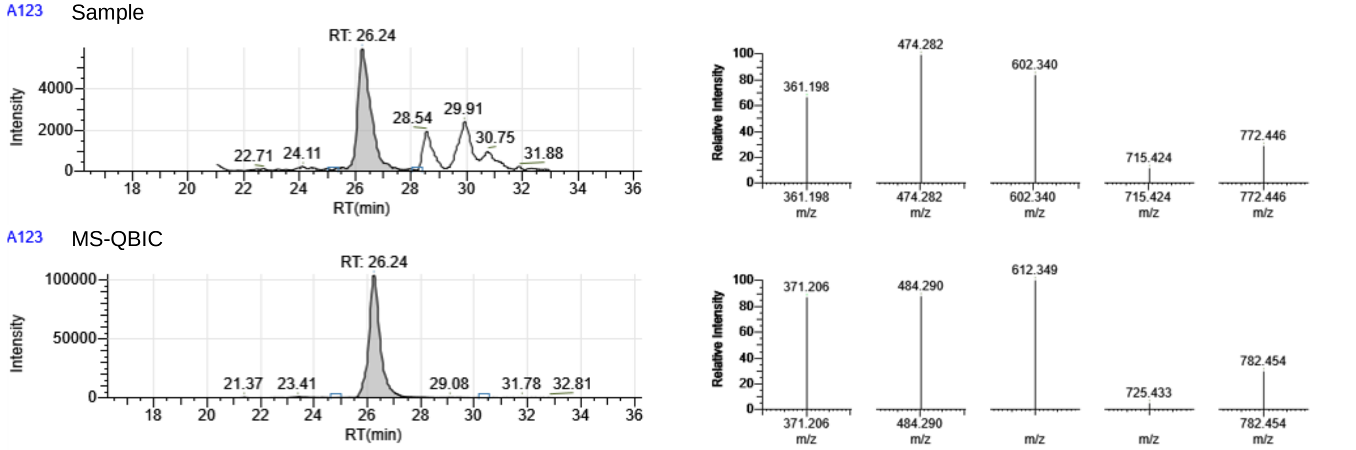

P15

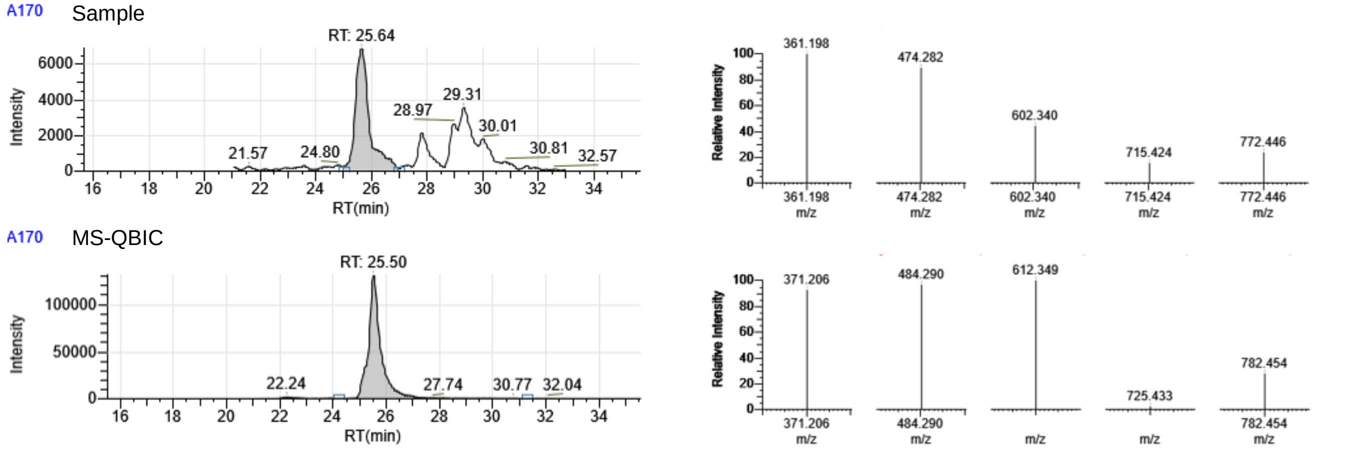

P16

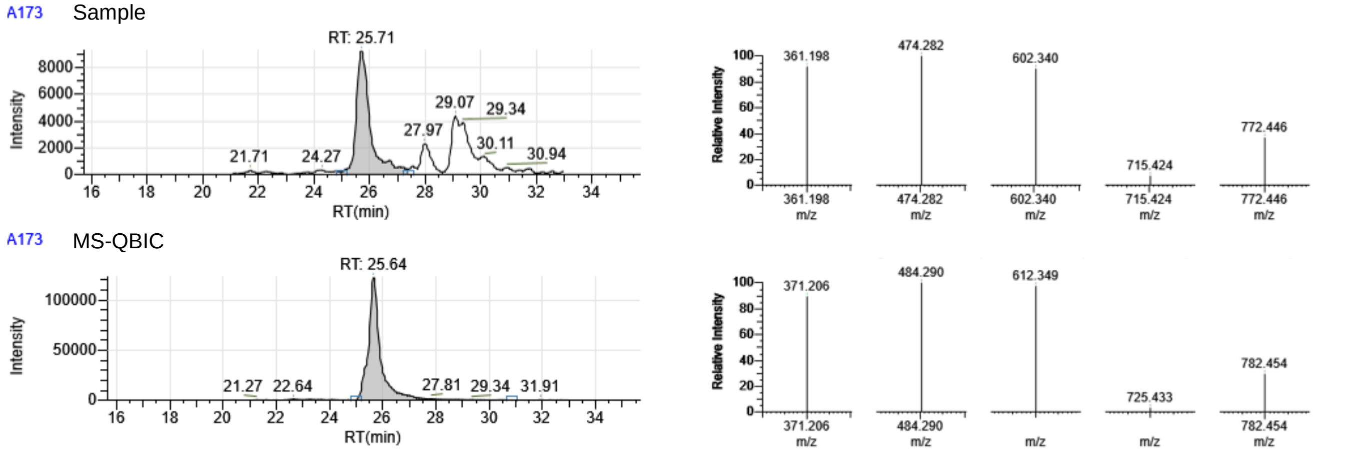

P17

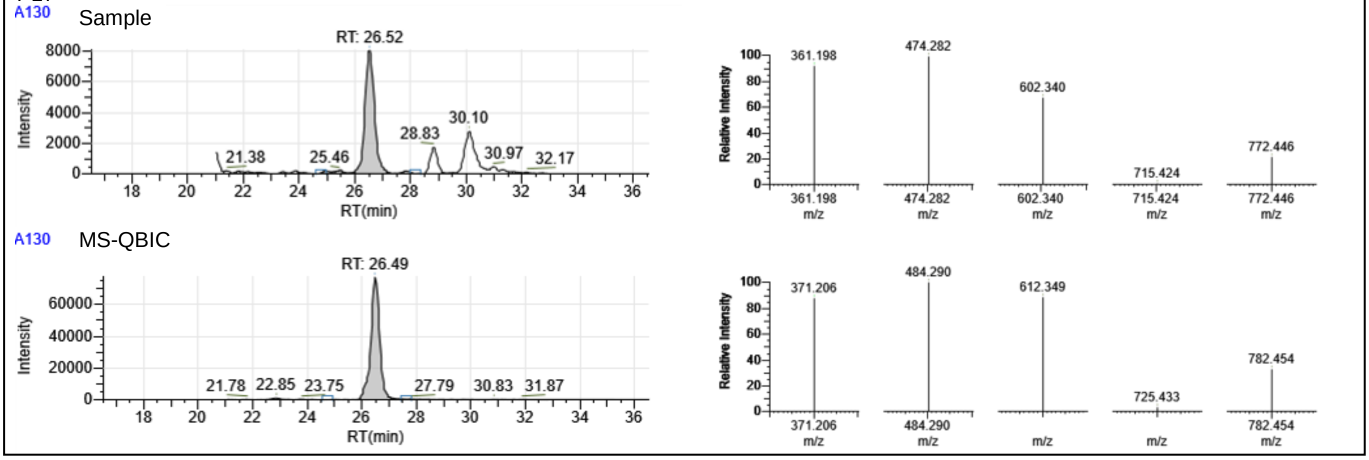

P18

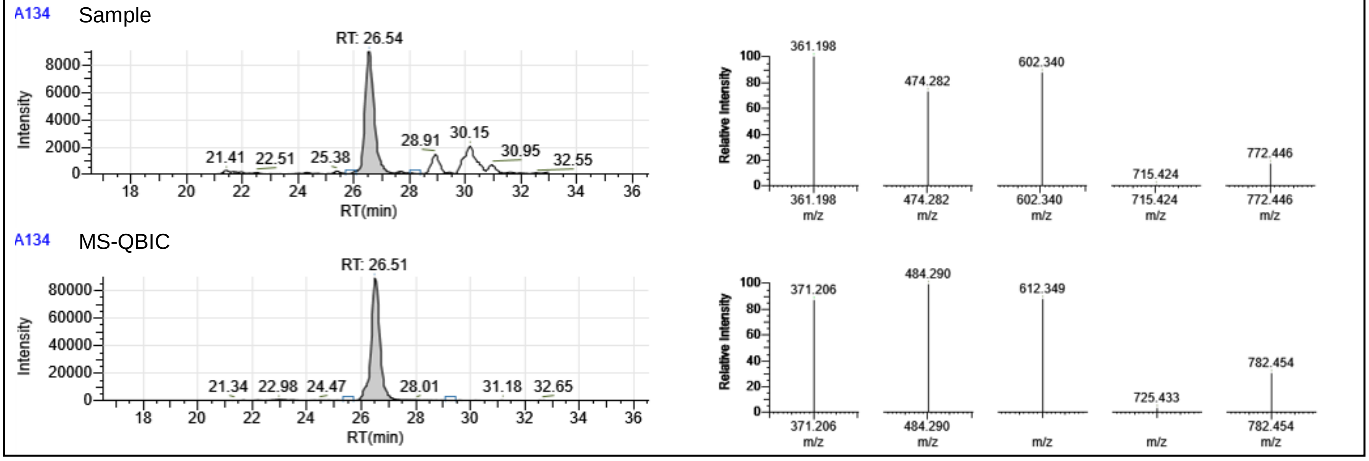

P19

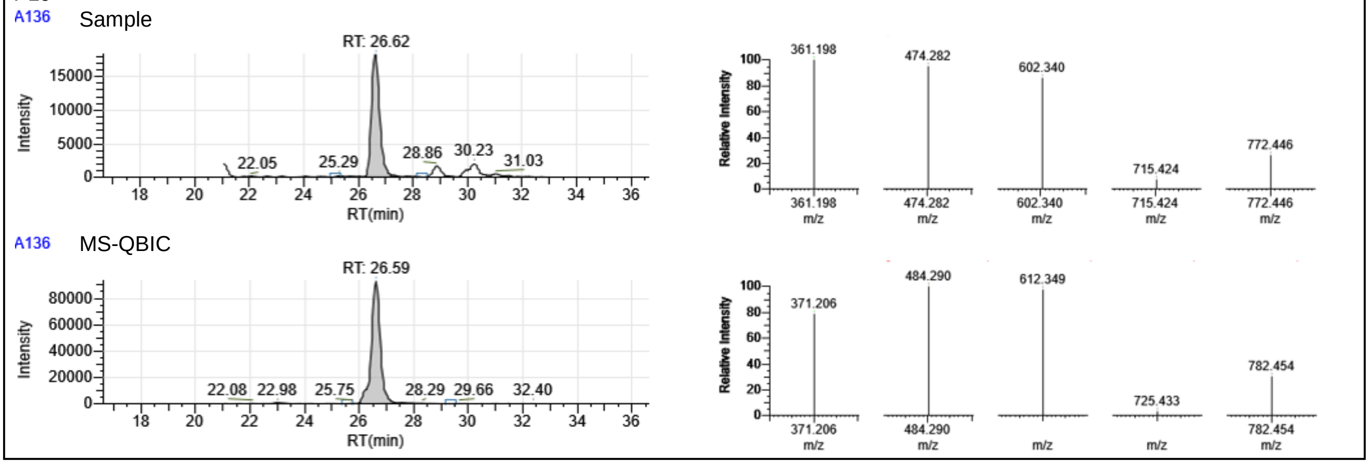

P20

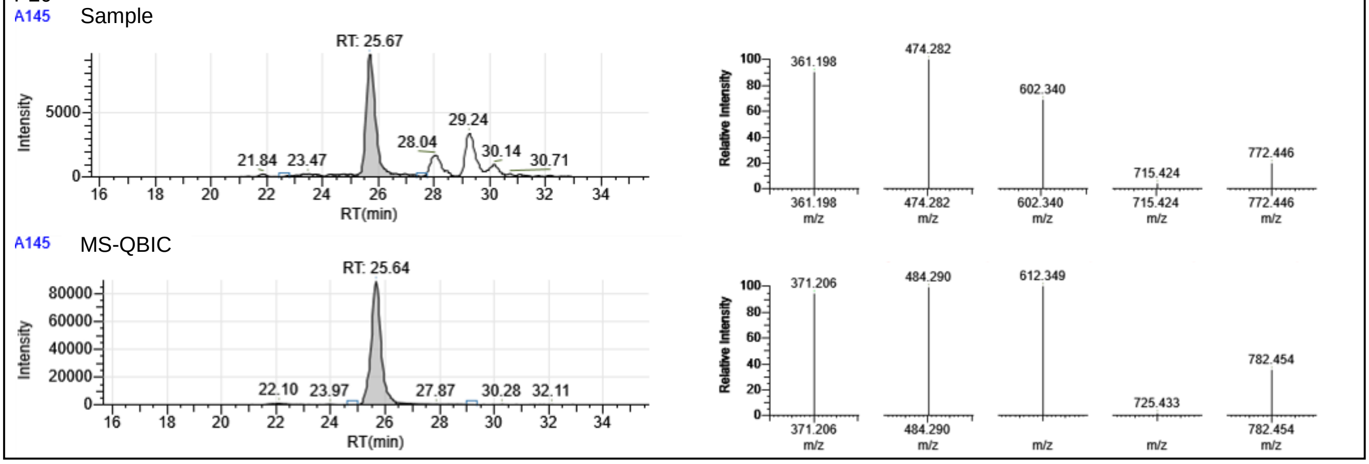

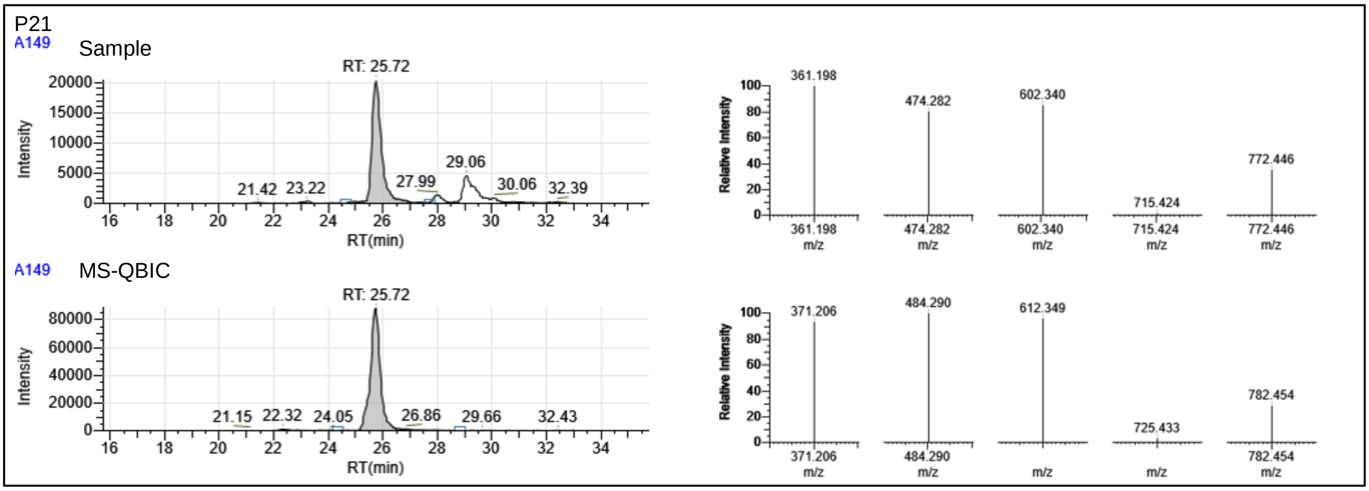
P21
A149 Sample
RT: 25.72
21.42 23.22 27.99 29.06 30.06 32.39
Intensity
RT(min)
361.198 474.282 602.340 715.424 772.446
Relative Intensity
m/z
A149 MS-QBIC
RT: 25.72
21.15 22.32 24.05 26.86 29.66 32.43
371.206 484.290 612.349 725.433 782.454

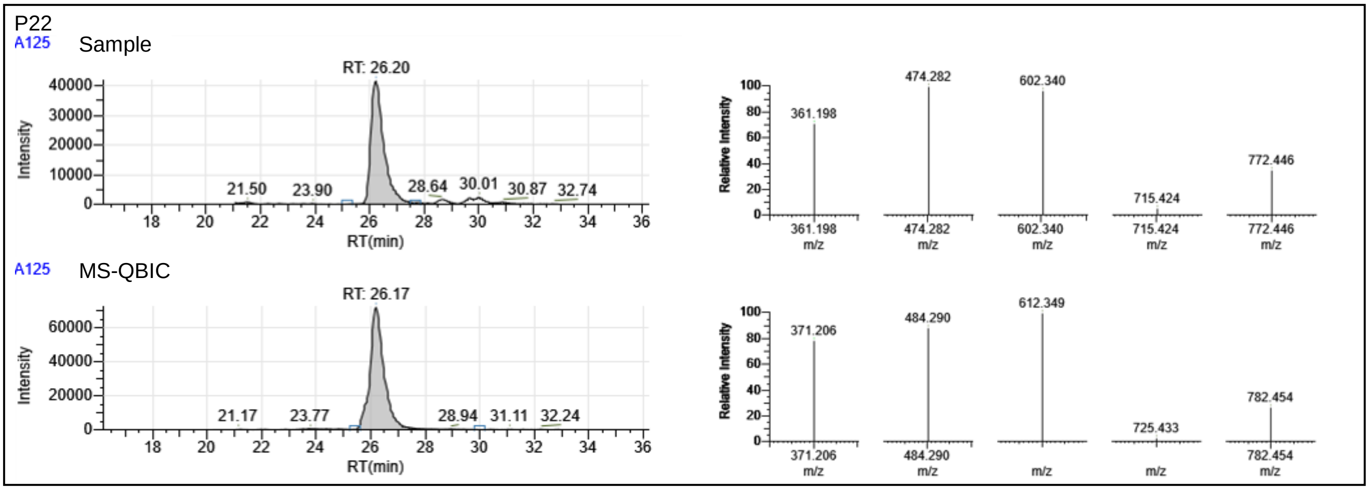
P22
A125 Sample
RT: 26.20
21.50 23.90 28.64 30.01 30.87 32.74
Intensity
RT(min)
361.198 474.282 602.340 715.424 772.446
Relative Intensity
m/z
A125 MS-QBIC
RT: 26.17
21.17 23.77 28.94 31.11 32.24
371.206 484.290 612.349 725.433 782.454

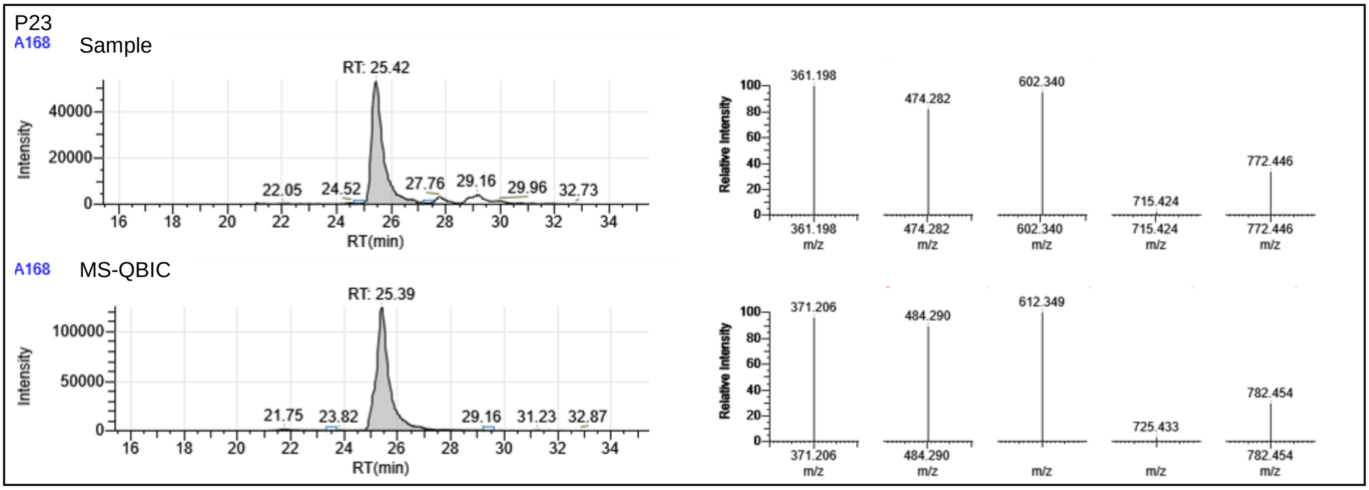
P23
A168 Sample
RT: 25.42
22.05 24.52 27.76 29.16 29.96 32.73
Intensity
RT(min)
361.198 474.282 602.340 715.424 772.446
Relative Intensity
m/z
A168 MS-QBIC
RT: 25.39
21.75 23.82 29.16 31.23 32.87
371.206 484.290 612.349 725.433 782.454

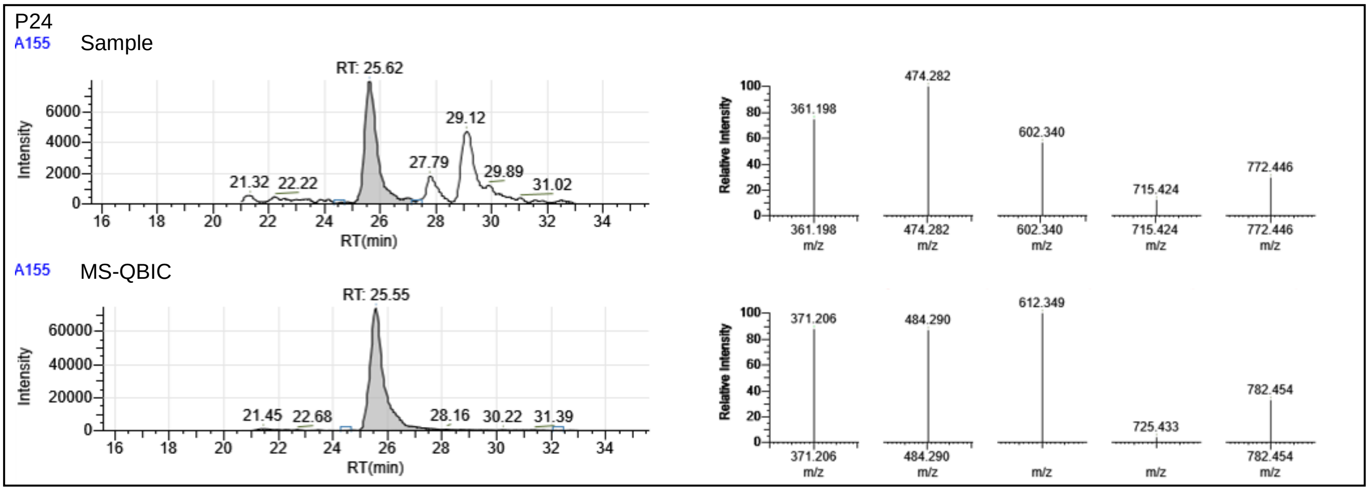
P24
A155 Sample
RT: 25.62
21.32 22.22 27.79 29.12 29.89 31.02
Intensity
RT(min)
361.198 474.282 602.340 715.424 772.446
Relative Intensity
m/z
A155 MS-QBIC
RT: 25.55
21.45 22.68 28.16 30.22 31.39
371.206 484.290 612.349 725.433 782.454

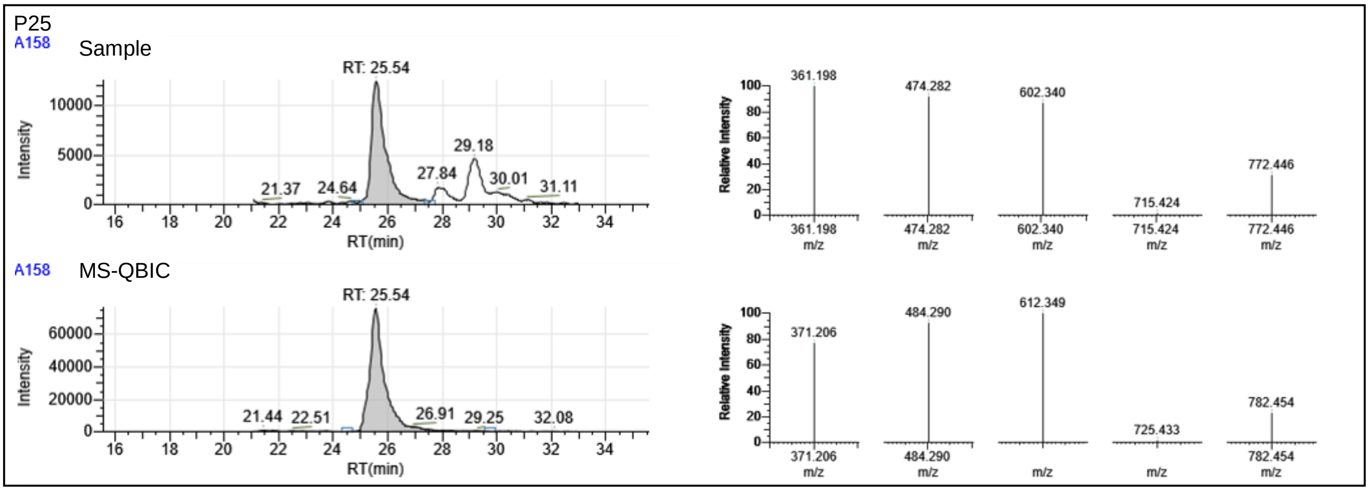
P25
A158 Sample
RT: 25.54
29.18
27.84
30.01
31.11
21.37
24.64
Intensity
RT(min)
Relative Intensity
361.198
474.282
602.340
715.424
772.446
m/z
A158 MS-QBIC
RT: 25.54
21.44
22.51
26.91
29.25
32.08
371.206
484.290
612.349
725.433
782.454

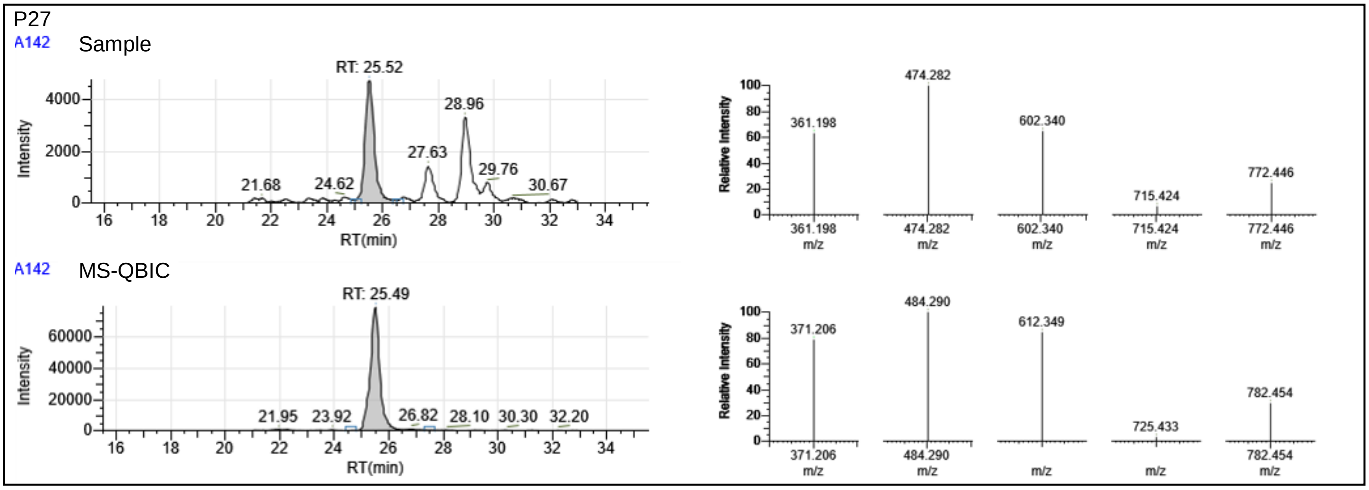
P27
A142 Sample
RT: 25.52
28.96
27.63
21.68
24.62
29.76
30.67
Intensity
RT(min)
Relative Intensity
361.198
474.282
602.340
715.424
772.446
m/z
A142 MS-QBIC
RT: 25.49
21.95
23.92
26.82
28.10
30.30
32.20
371.206
484.290
612.349
725.433
782.454

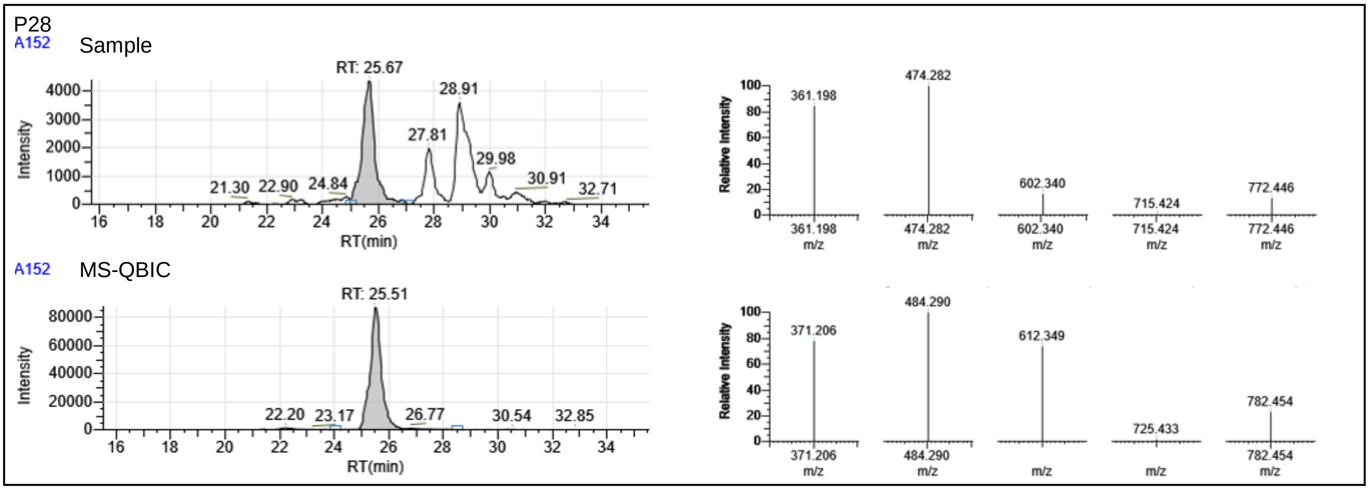
P28
A152 Sample
RT: 25.67
28.91
27.81
29.98
21.30
22.90
24.84
30.91
32.71
Intensity
RT(min)
Relative Intensity
361.198
474.282
602.340
715.424
772.446
m/z
A152 MS-QBIC
RT: 25.51
22.20
23.17
26.77
30.54
32.85
371.206
484.290
612.349
725.433
782.454

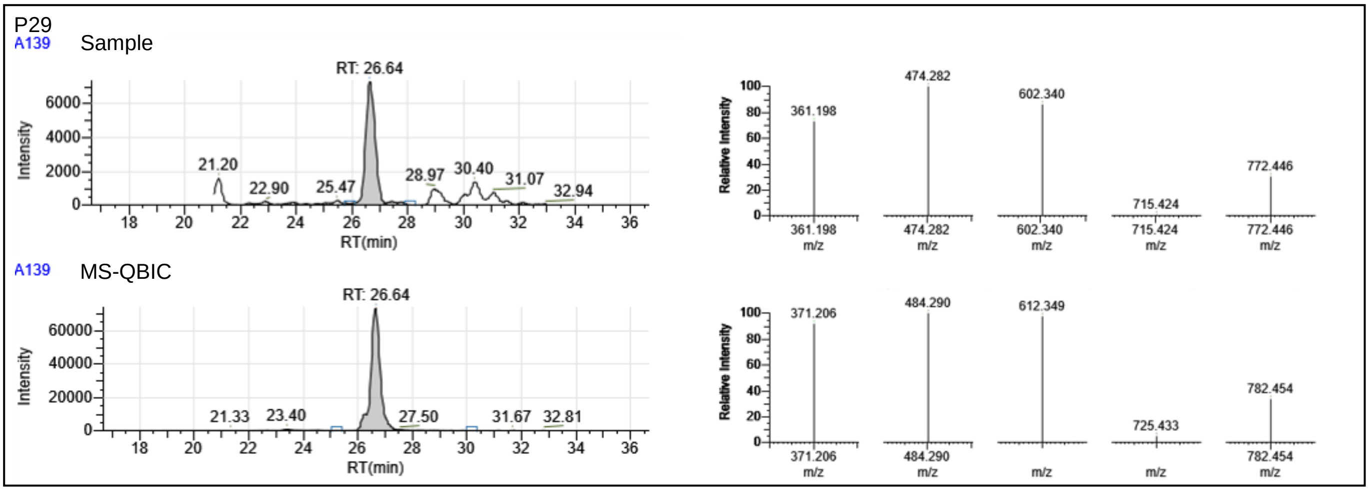
P29
A139 Sample
RT: 26.64
21.20
22.90
25.47
28.97
30.40
31.07
32.94
Intensity
RT(min)
Relative Intensity
361.198
474.282
602.340
715.424
772.446
m/z
A139 MS-QBIC
RT: 26.64
21.33
23.40
27.50
31.67
32.81
371.206
484.290
612.349
725.433
782.454

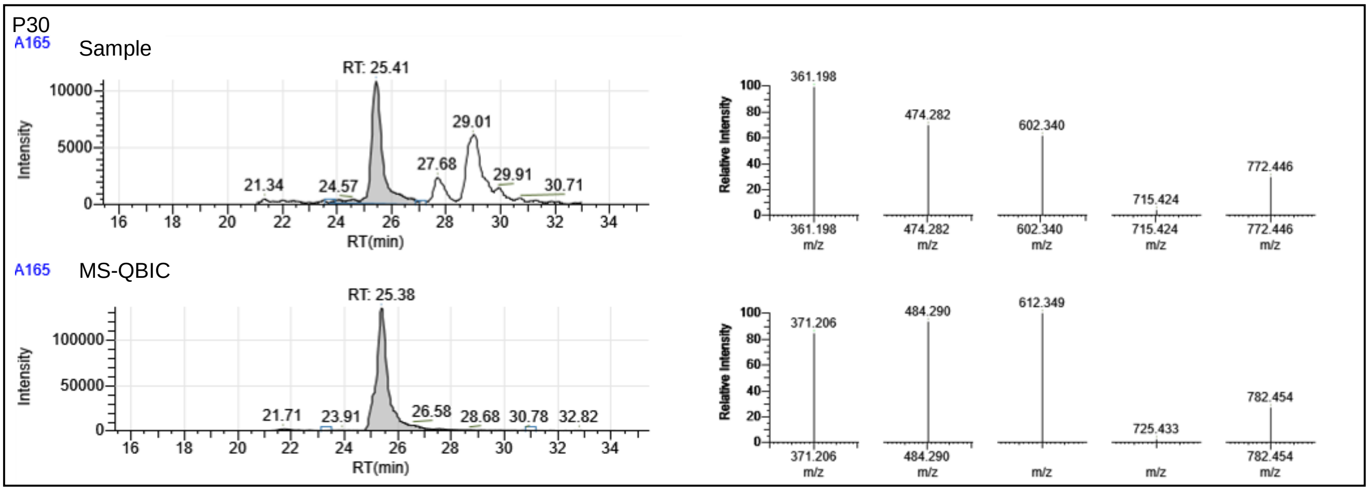
P30
A165
Sample
RT: 25.41
21.34
24.57
27.68
29.01
29.91
30.71
Intensity
RT(min)
Relative Intensity
361.198
474.282
602.340
715.424
772.446
m/z
A165
MS-QBIC
RT: 25.38
21.71
23.91
26.58
28.68
30.78
32.82
371.206
484.290
612.349
725.433
782.454

W FGA (GGSTSYGTGSETESPR) Another organ

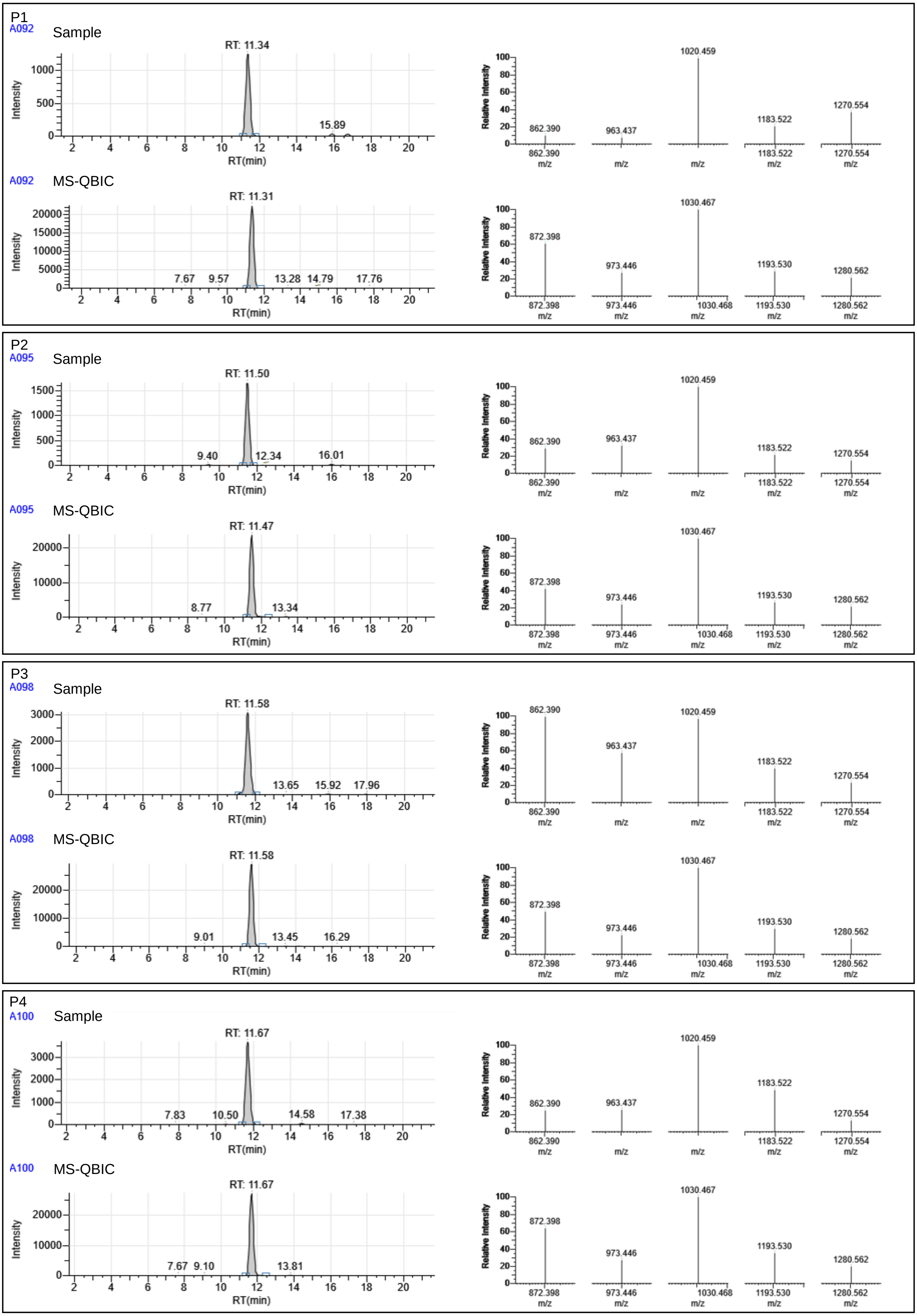

P6

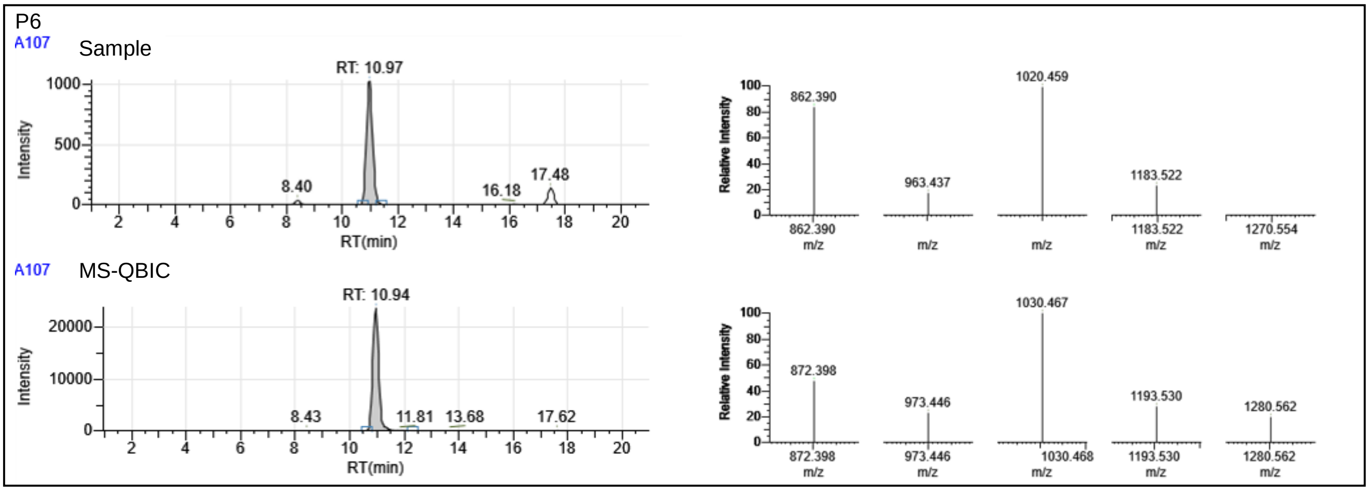
A107 Sample
RT: 10.97
Intensity
RT(min)
8.40
16.18
17.48
Relative Intensity
m/z
862.390
963.437
1020.459
1183.522
1270.554
A107 MS-QBIC
RT: 10.94
8.43
11.81
13.68
17.62
872.398
973.446
1030.467
1030.468
1193.530
1280.562

P7

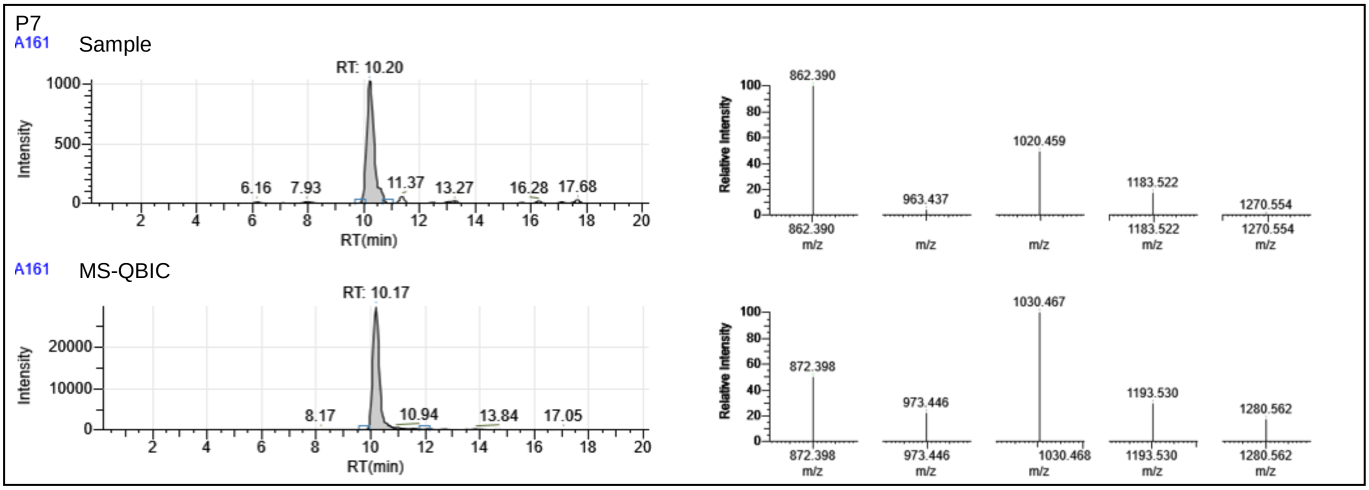
A161 Sample
RT: 10.20
Intensity
RT(min)
6.16
7.93
11.37
13.27
16.28
17.68
Relative Intensity
m/z
862.390
963.437
1020.459
1183.522
1270.554
A161 MS-QBIC
RT: 10.17
8.17
10.94
13.84
17.05
872.398
973.446
1030.467
1030.468
1193.530
1280.562

P8

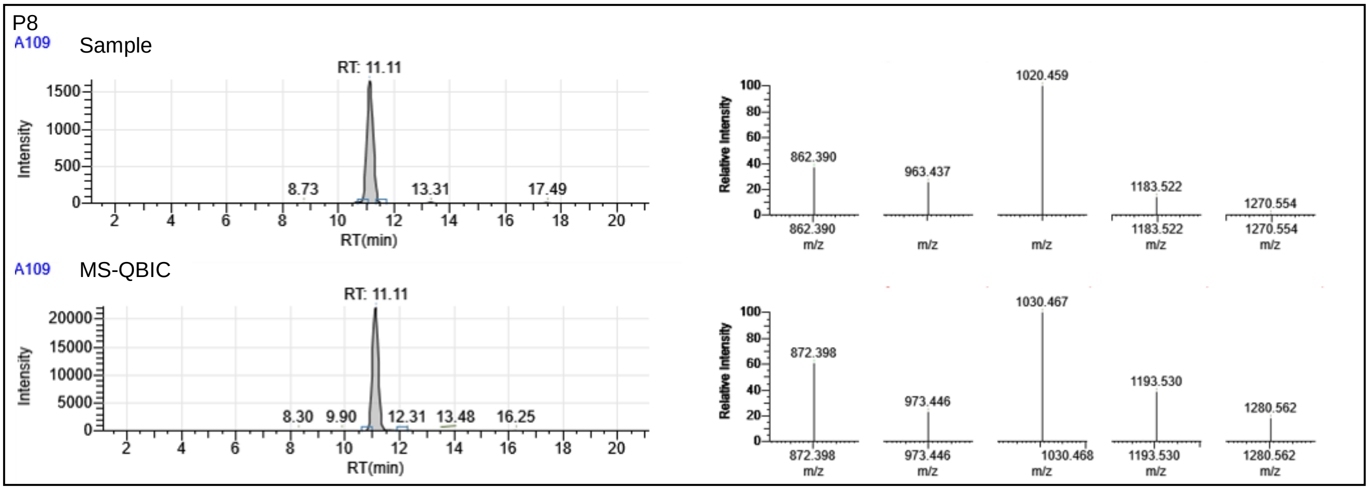
A109 Sample
RT: 11.11
Intensity
RT(min)
8.73
13.31
17.49
Relative Intensity
m/z
862.390
963.437
1020.459
1183.522
1270.554
A109 MS-QBIC
RT: 11.11
8.30
9.90
12.31
13.48
16.25
872.398
973.446
1030.467
1030.468
1193.530
1280.562

P9

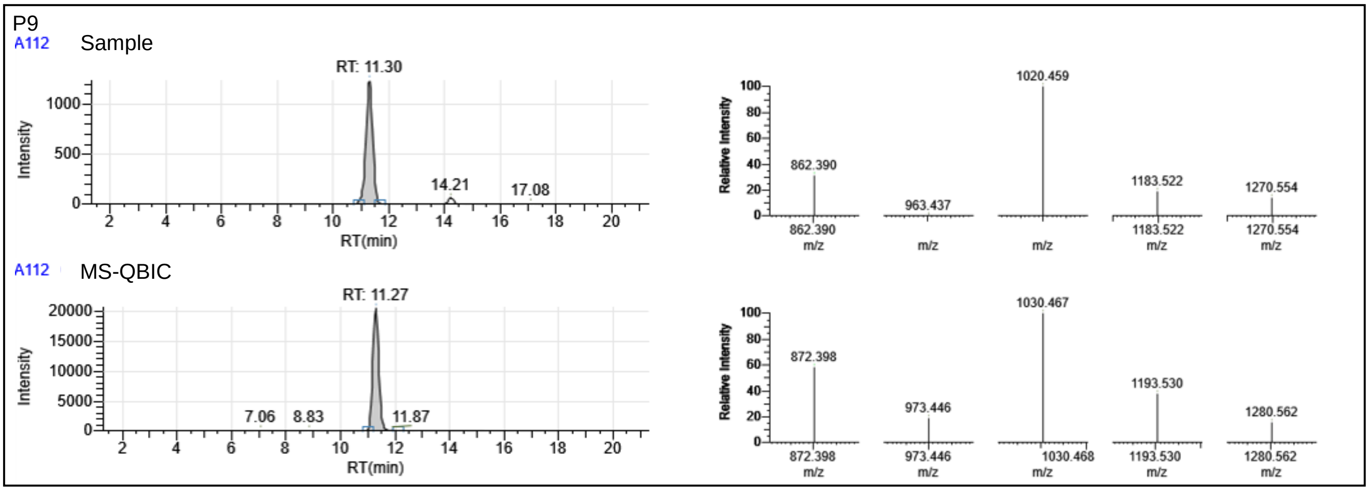
A112 Sample
RT: 11.30
Intensity
RT(min)
14.21
17.08
Relative Intensity
m/z
862.390
963.437
1020.459
1183.522
1270.554
A112 MS-QBIC
RT: 11.27
7.06
8.83
11.87
872.398
973.446
1030.467
1030.468
1193.530
1280.562

P15

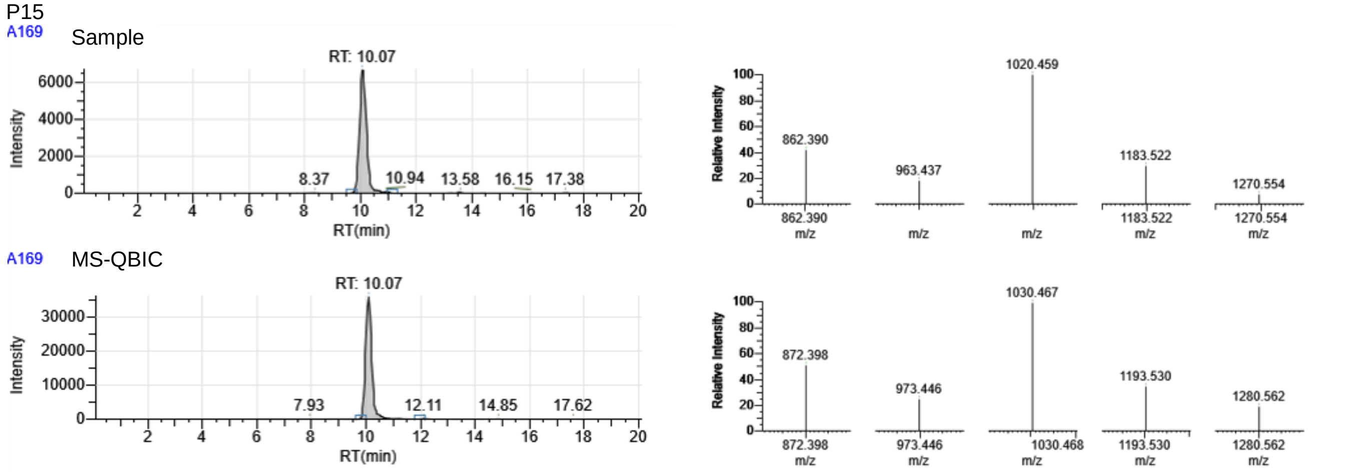

P16

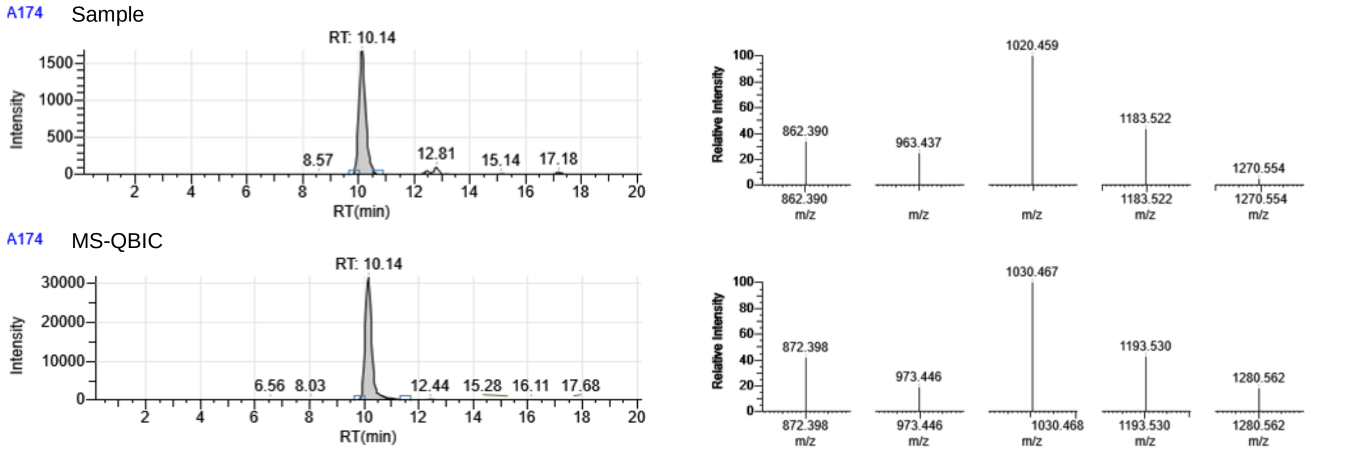

P17

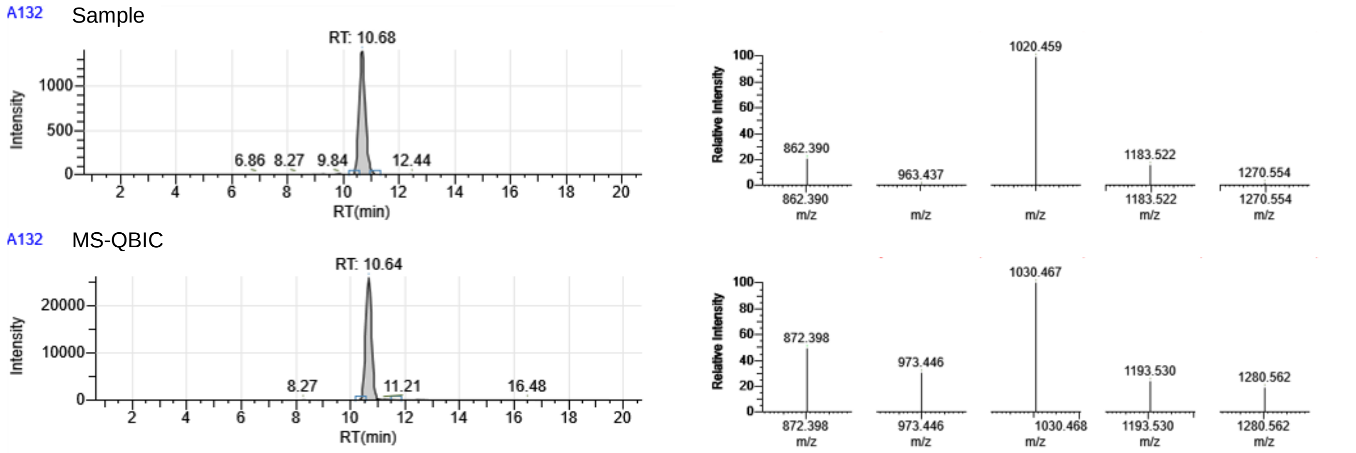

P20

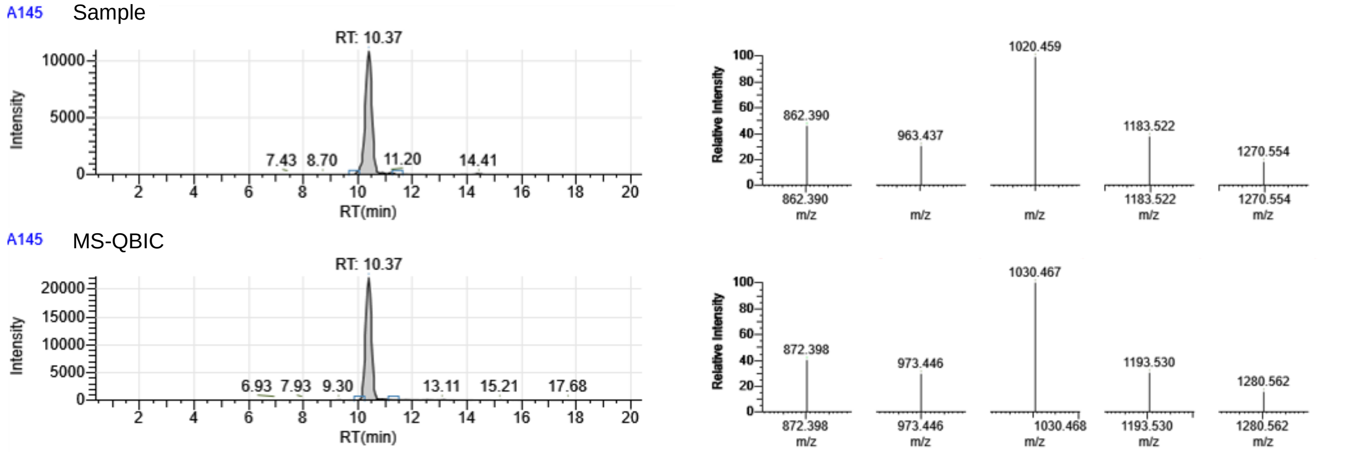

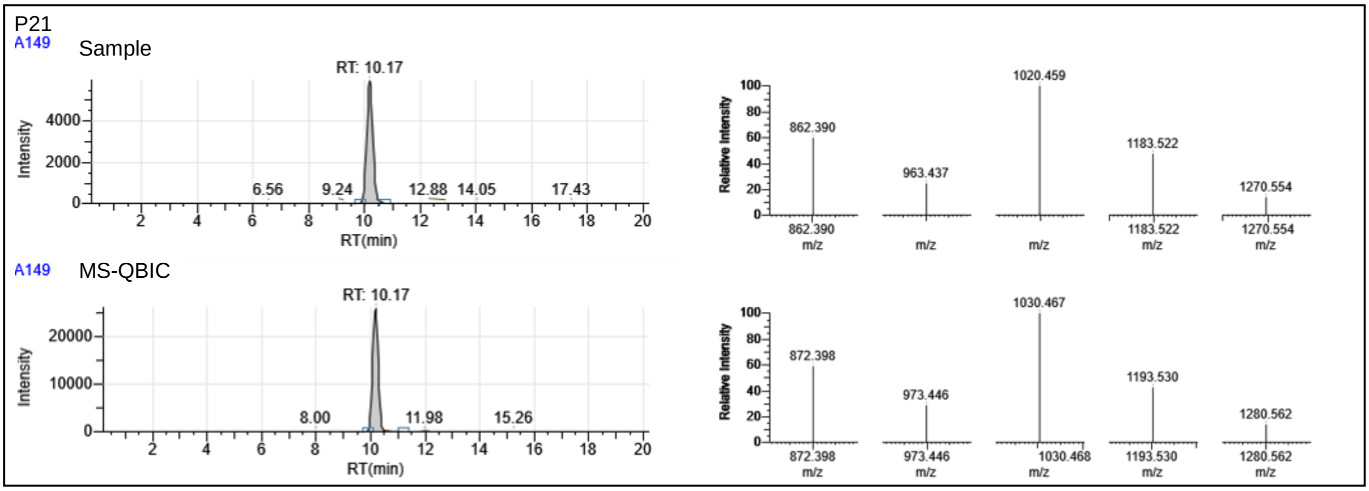
P21
A149 Sample
RT: 10.17
Intensity
RT(min)
6.56 9.24 12.88 14.05 17.43
Relative Intensity
862.390
963.437
1020.459
1183.522
1270.554
m/z
A149 MS-QBIC
RT: 10.17
8.00 11.98 15.26
872.398
973.446
1030.467
1030.468
1193.530
1280.562

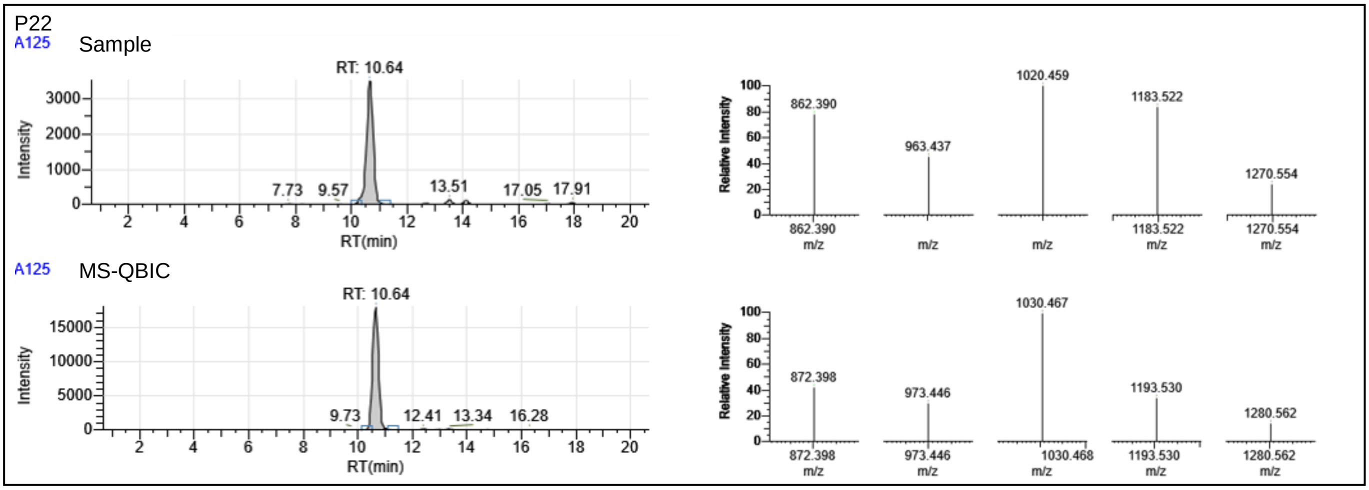
P22
A125 Sample
RT: 10.64
Intensity
RT(min)
7.73 9.57 13.51 17.05 17.91
Relative Intensity
862.390
963.437
1020.459
1183.522
1270.554
m/z
A125 MS-QBIC
RT: 10.64
9.73 12.41 13.34 16.28
872.398
973.446
1030.467
1030.468
1193.530
1280.562

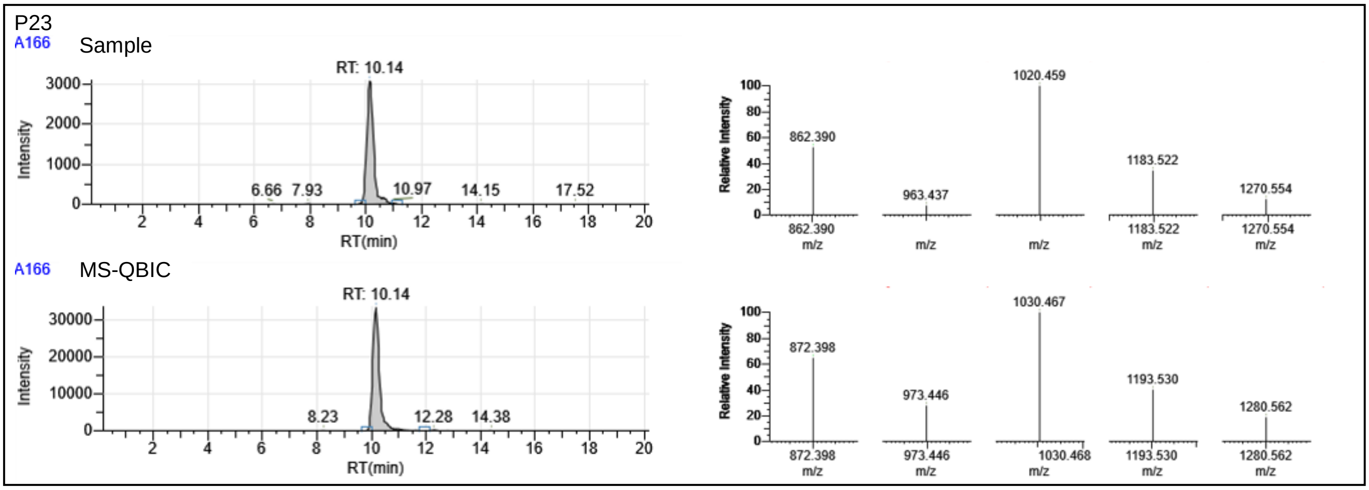
P23
A166 Sample
RT: 10.14
Intensity
RT(min)
6.66 7.93 10.97 14.15 17.52
Relative Intensity
862.390
963.437
1020.459
1183.522
1270.554
m/z
A166 MS-QBIC
RT: 10.14
8.23 12.28 14.38
872.398
973.446
1030.467
1030.468
1193.530
1280.562

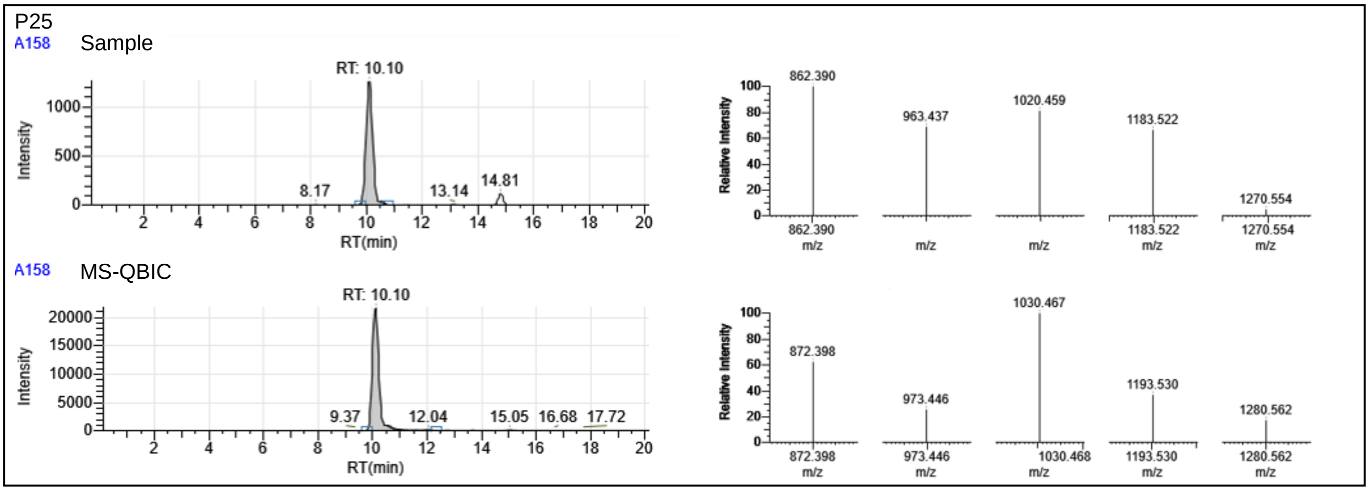
P25
A158 Sample
RT: 10.10
Intensity
RT(min)
8.17 13.14 14.81
Relative Intensity
862.390
963.437
1020.459
1183.522
1270.554
m/z
A158 MS-QBIC
RT: 10.10
9.37 12.04 15.05 16.68 17.72
872.398
973.446
1030.467
1030.468
1193.530
1280.562

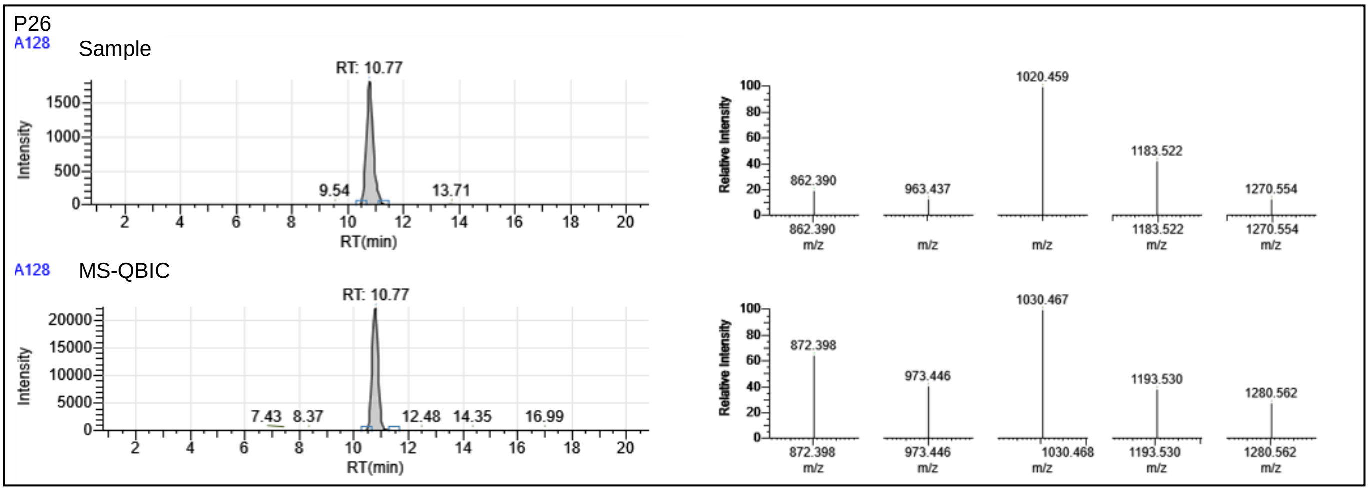
P26
A128 Sample
RT: 10.77
9.54
13.71
Intensity
RT(min)
Relative Intensity
1020.459
862.390
963.437
1183.522
1270.554
m/z
A128 MS-QBIC
RT: 10.77
7.43 8.37
12.48 14.35 16.99
1030.467
872.398
973.446
1193.530
1280.562
1030.468

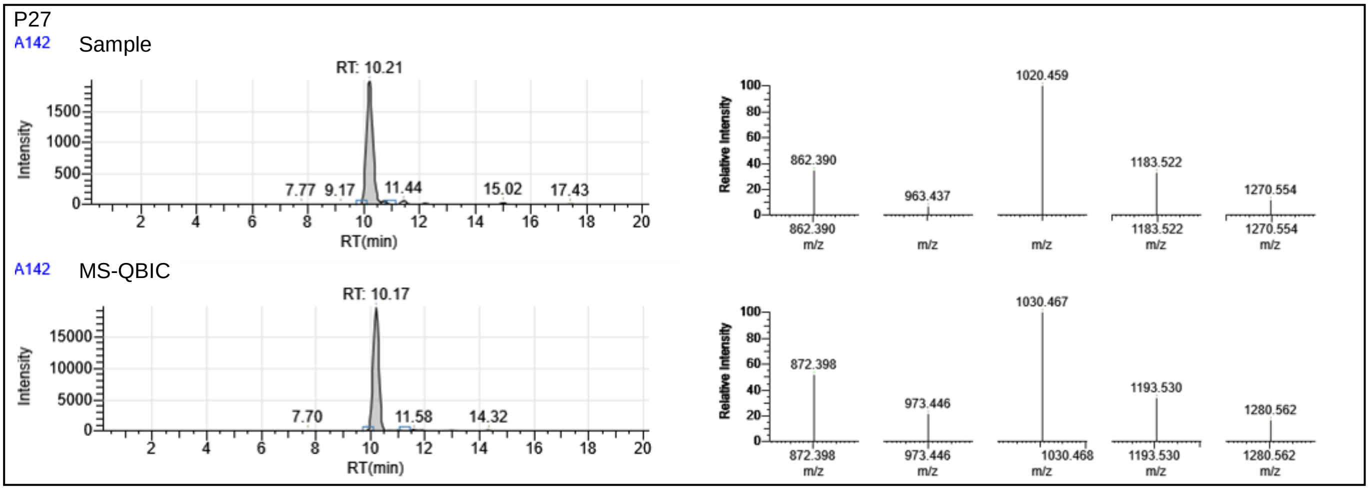
P27
A142 Sample
RT: 10.21
7.77 9.17
11.44
15.02 17.43
Intensity
RT(min)
Relative Intensity
1020.459
862.390
963.437
1183.522
1270.554
m/z
A142 MS-QBIC
RT: 10.17
7.70
11.58 14.32
1030.467
872.398
973.446
1193.530
1280.562
1030.468

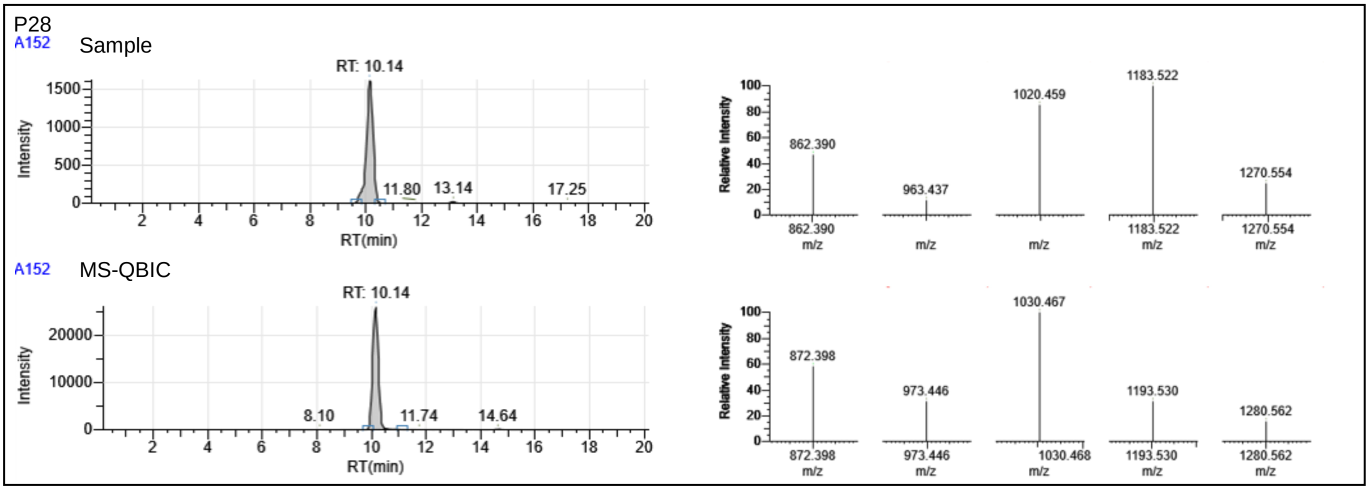
P28
A152 Sample
RT: 10.14
11.80 13.14
17.25
Intensity
RT(min)
Relative Intensity
1183.522
1020.459
862.390
963.437
1270.554
m/z
A152 MS-QBIC
RT: 10.14
8.10
11.74 14.64
1030.467
872.398
973.446
1193.530
1280.562
1030.468

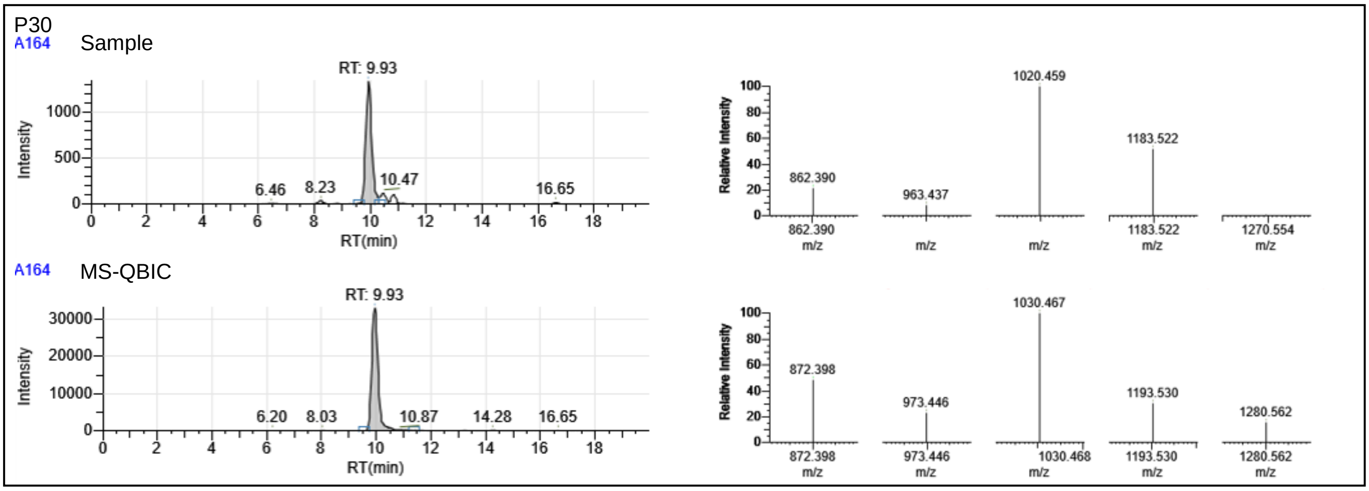
P30
A164 Sample
RT: 9.93
6.46 8.23
10.47
16.65
Intensity
RT(min)
Relative Intensity
1020.459
1183.522
862.390
963.437
1270.554
m/z
A164 MS-QBIC
RT: 9.93
6.20 8.03
10.87 14.28 16.65
1030.467
872.398
973.446
1193.530
1280.562
1030.468

X Lysozyme (STDYGIFQINSR) Another organ

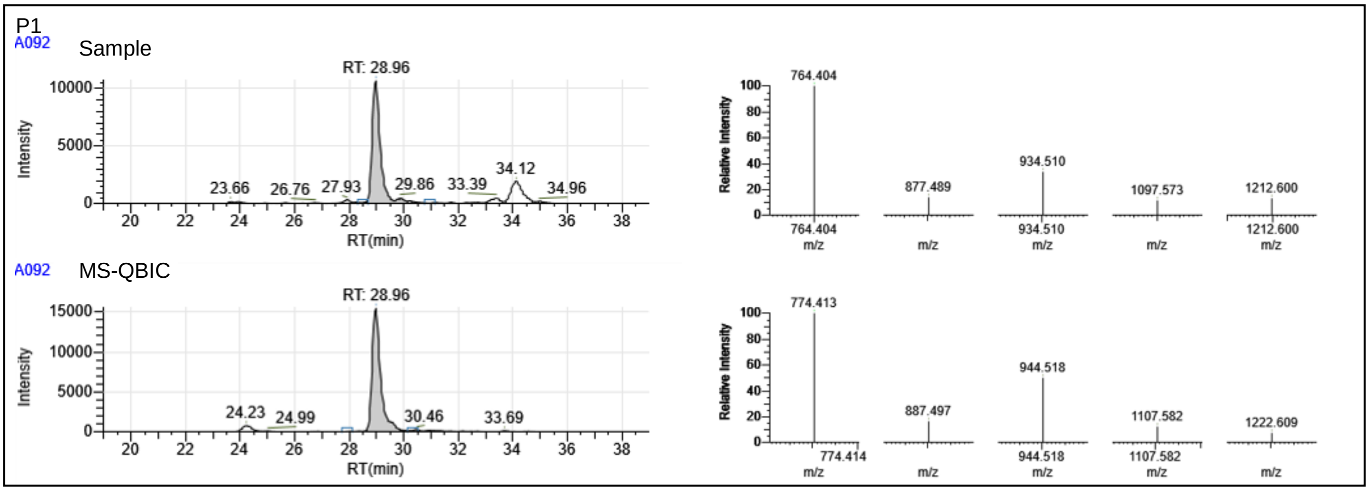

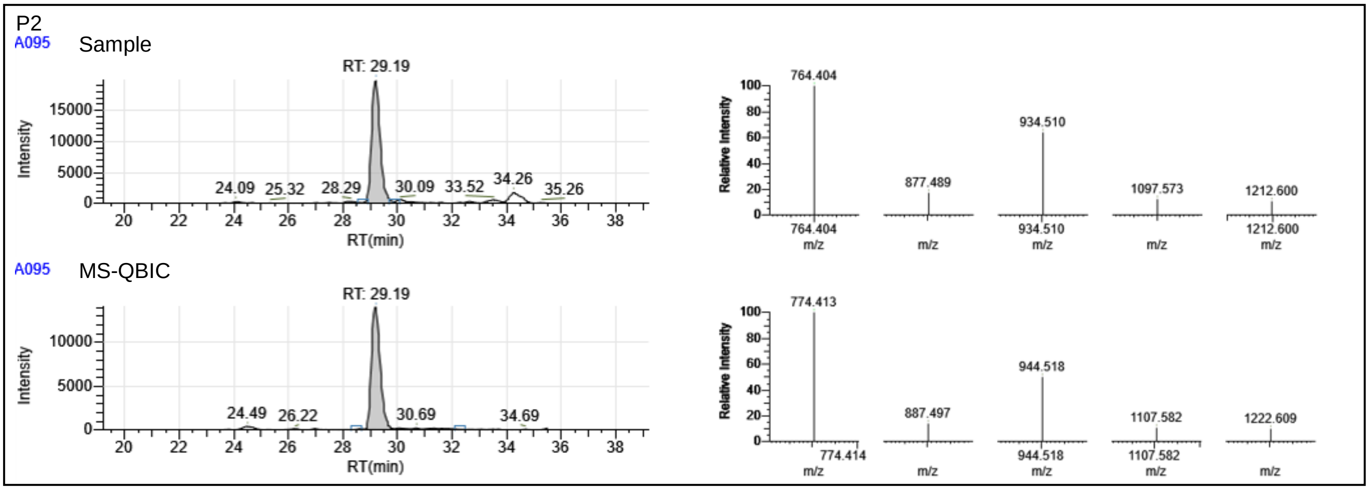

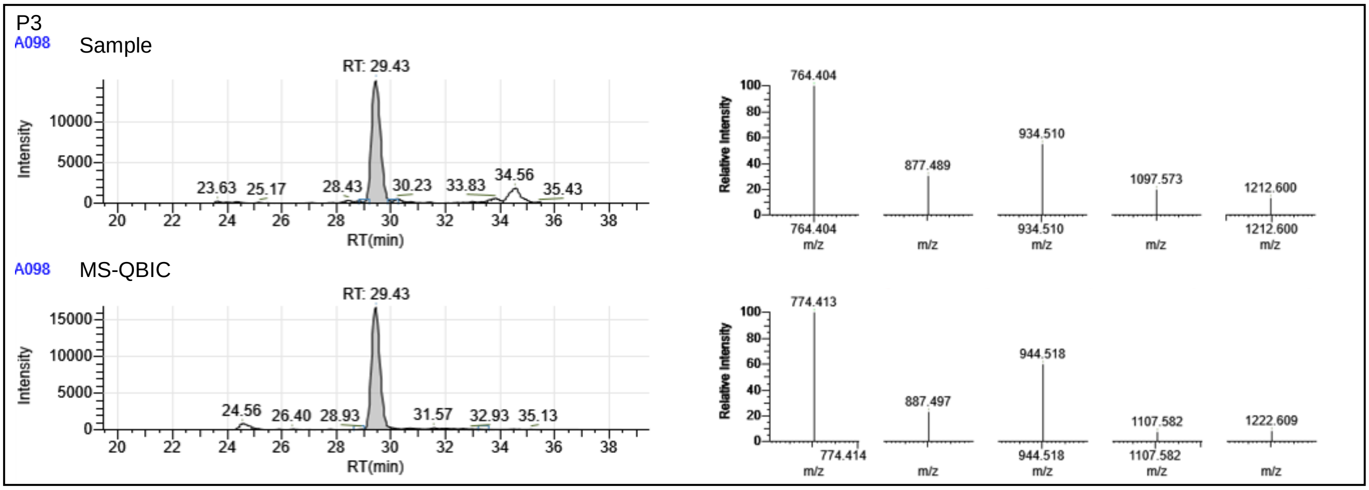

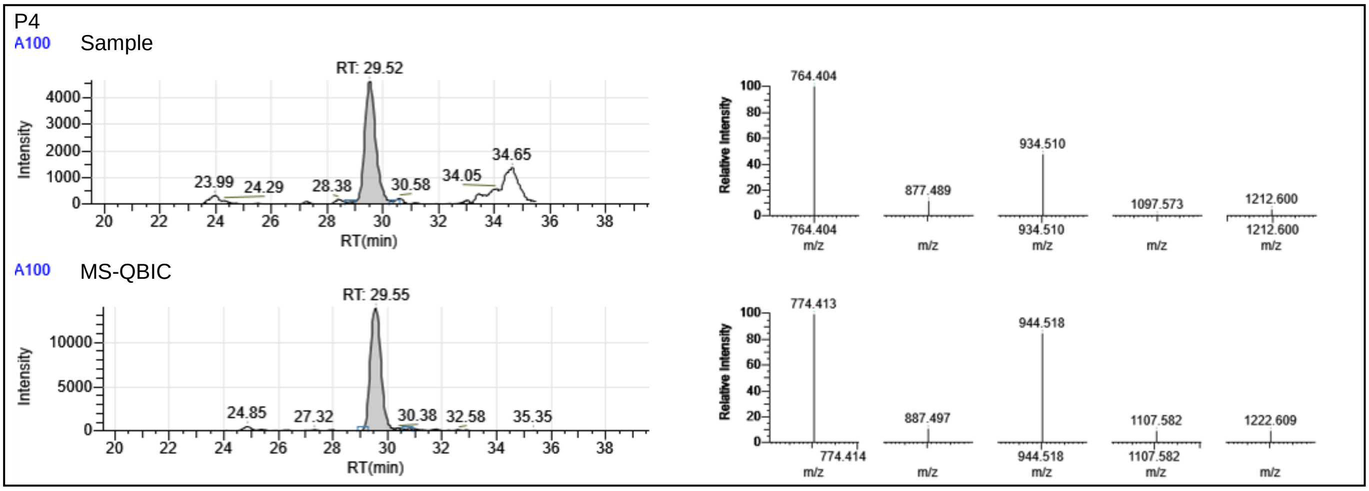

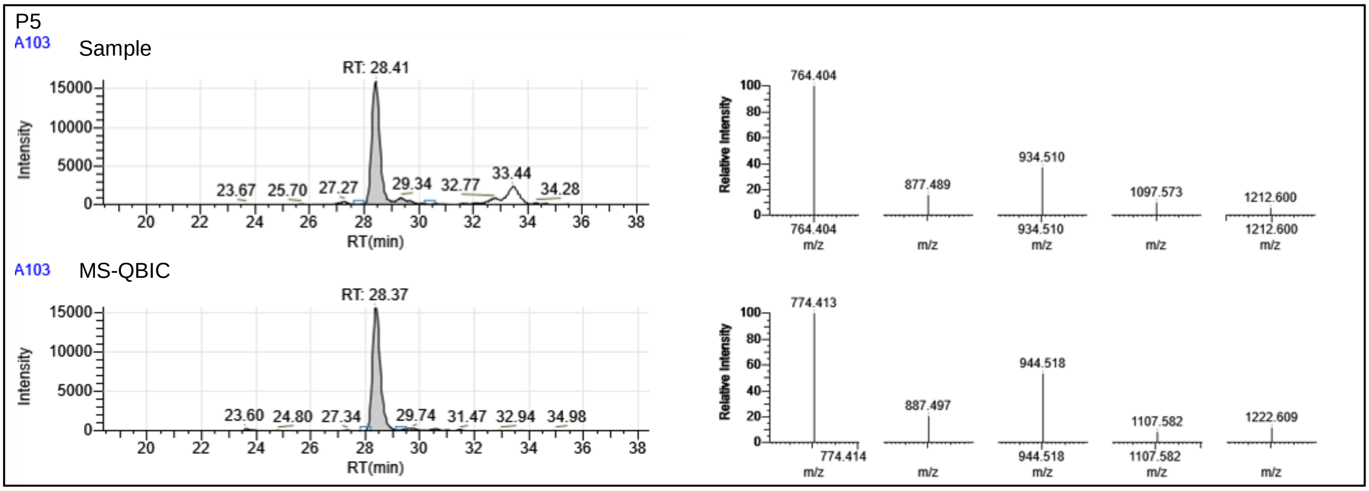
P5
A103 Sample
RT: 28.41
23.67 25.70 27.27 29.34 32.77 33.44 34.28
Intensity
RT(min)
764.404
877.489
934.510
1097.573
1212.600
Relative Intensity
m/z
A103 MS-QBIC
RT: 28.37
23.60 24.80 27.34 29.74 31.47 32.94 34.98
774.413
887.497
944.518
1107.582
1222.609
774.414

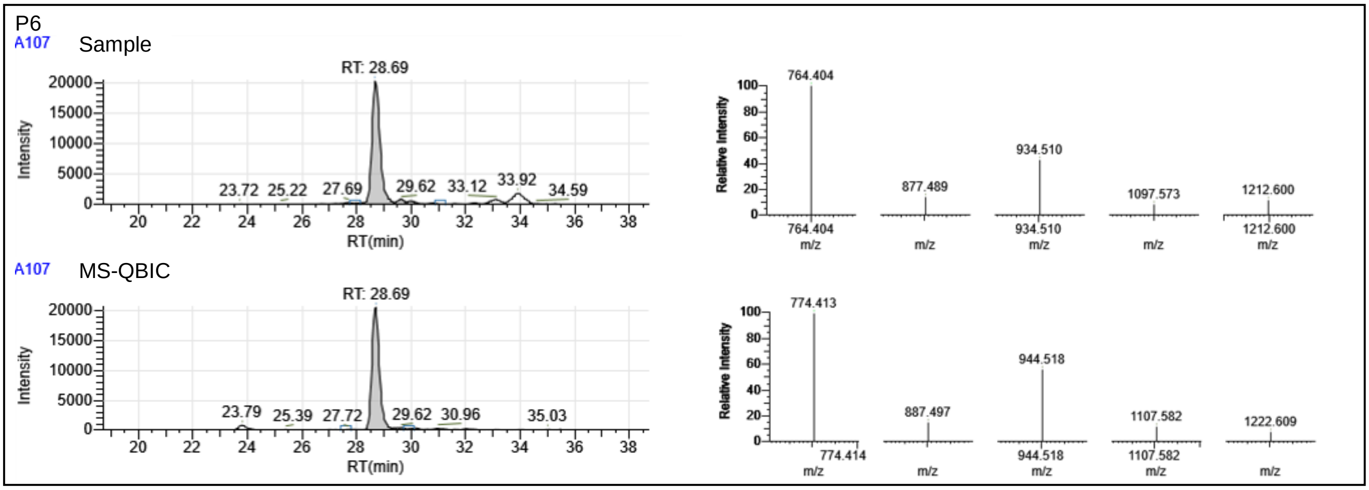
P6
A107 Sample
RT: 28.69
23.72 25.22 27.69 29.62 33.12 33.92 34.59
Intensity
RT(min)
764.404
877.489
934.510
1097.573
1212.600
Relative Intensity
m/z
A107 MS-QBIC
RT: 28.69
23.79 25.39 27.72 29.62 30.96 35.03
774.413
887.497
944.518
1107.582
1222.609
774.414

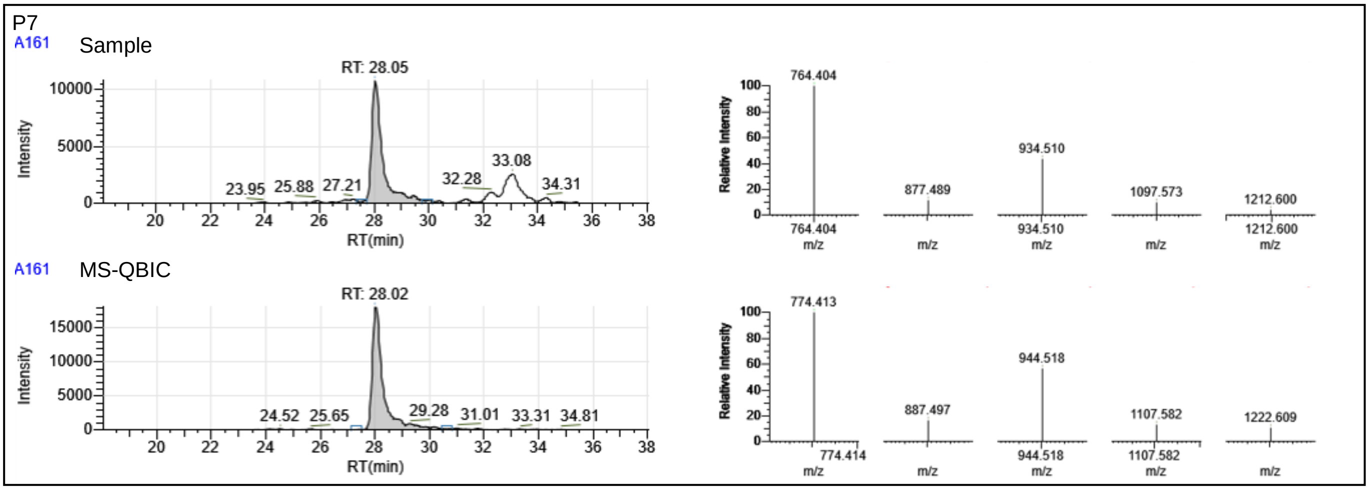
P7
A161 Sample
RT: 28.05
23.95 25.88 27.21 32.28 33.08 34.31
Intensity
RT(min)
764.404
877.489
934.510
1097.573
1212.600
Relative Intensity
m/z
A161 MS-QBIC
RT: 28.02
24.52 25.65 29.28 31.01 33.31 34.81
774.413
887.497
944.518
1107.582
1222.609
774.414

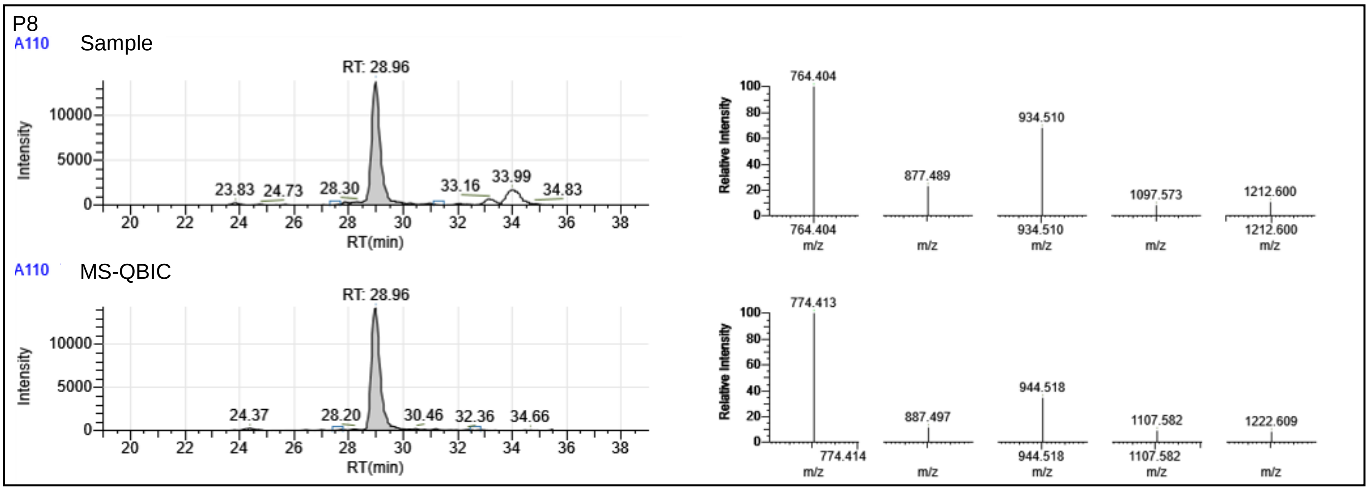
P8
A110 Sample
RT: 28.96
23.83 24.73 28.30 33.16 33.99 34.83
Intensity
RT(min)
764.404
877.489
934.510
1097.573
1212.600
Relative Intensity
m/z
A110 MS-QBIC
RT: 28.96
24.37 28.20 30.46 32.36 34.66
774.413
887.497
944.518
1107.582
1222.609
774.414

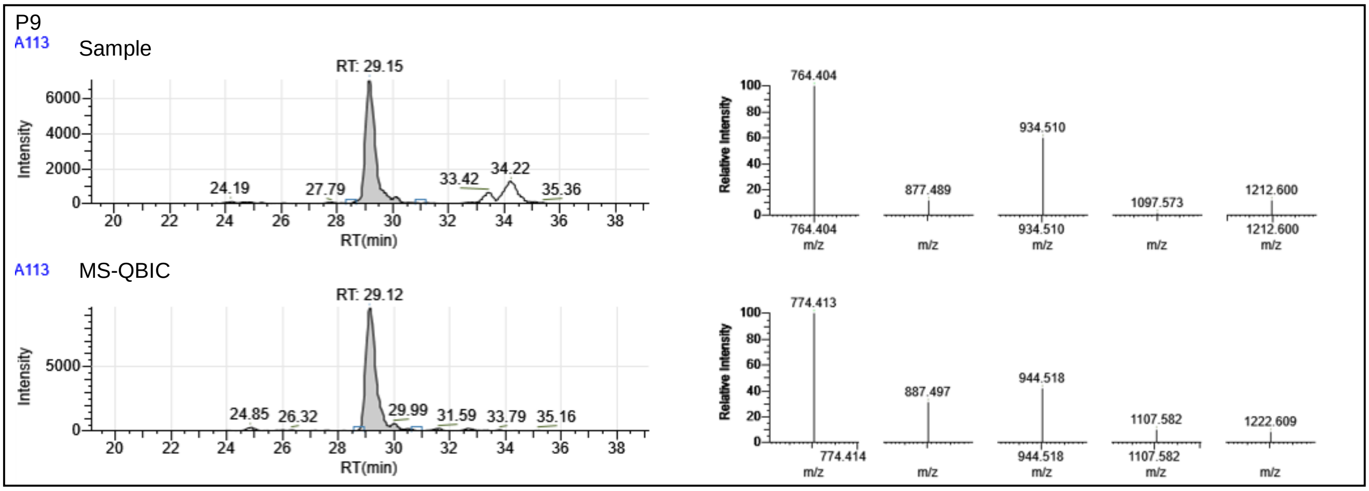
P9
A113 Sample
RT: 29.15
Intensity
6000
4000
2000
0
24.19
27.79
33.42
34.22
35.36
20 22 24 26 28 30 32 34 36 38
RT(min)
764.404
934.510
877.489
1097.573
1212.600
Relative Intensity
100 80 60 40 20 0
m/z
A113 MS-QBIC
RT: 29.12
5000
24.85 26.32
29.99
31.59 33.79 35.16
774.413
887.497
944.518
1107.582
1222.609
774.414

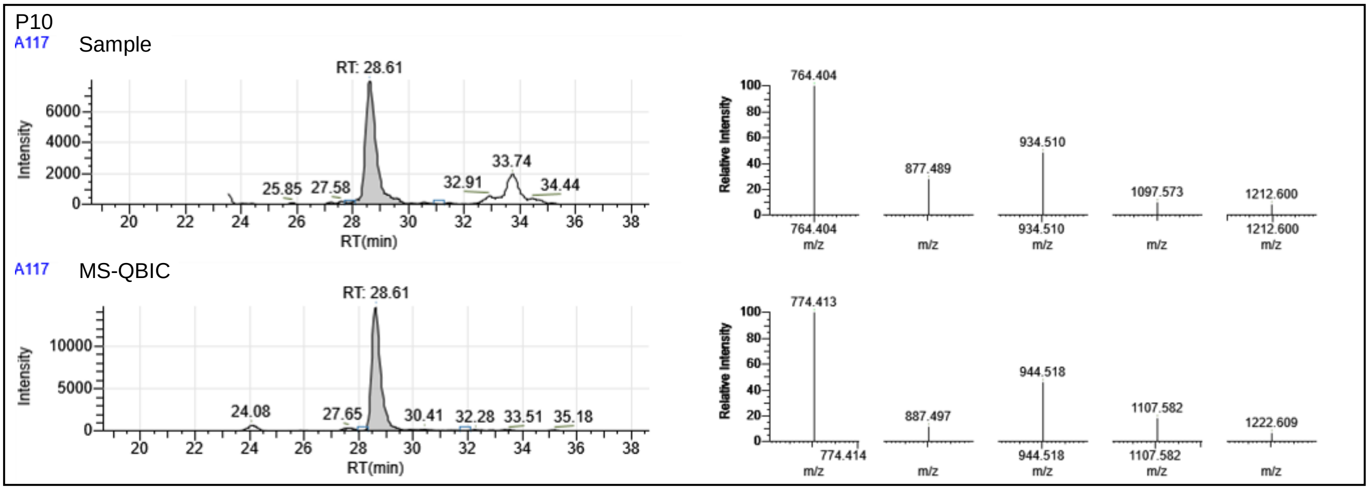
P10
A117 Sample
RT: 28.61
Intensity
6000
4000
2000
0
25.85 27.58
32.91
33.74
34.44
20 22 24 26 28 30 32 34 36 38
RT(min)
764.404
877.489
934.510
1097.573
1212.600
Relative Intensity
m/z
A117 MS-QBIC
RT: 28.61
10000
5000
24.08
27.65
30.41 32.28 33.51 35.18
774.413
887.497
944.518
1107.582
1222.609
774.414

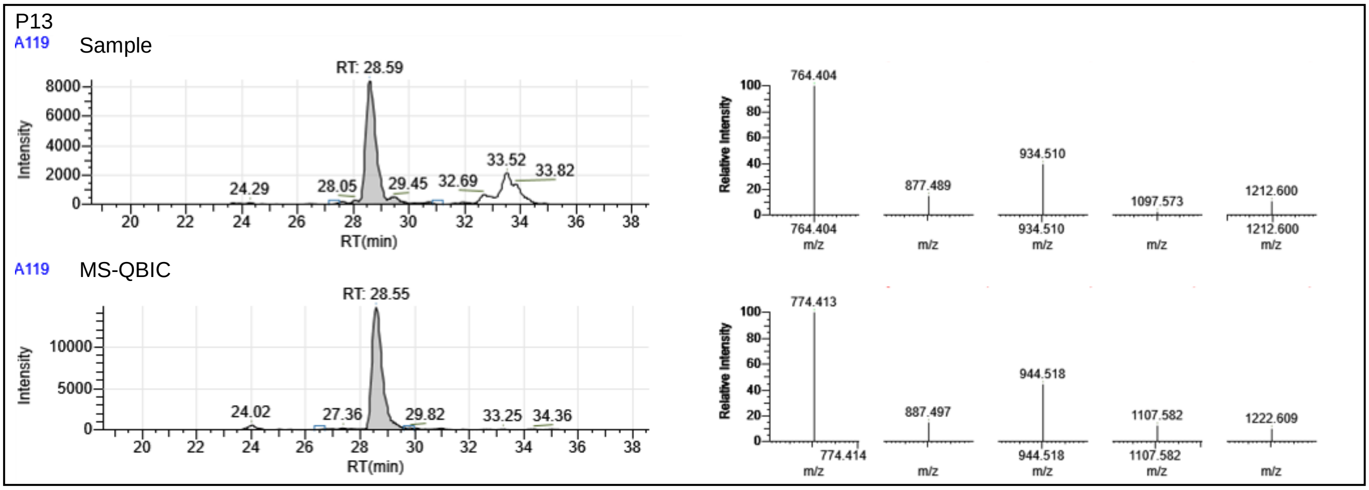
P13
A119 Sample
RT: 28.59
Intensity
8000
6000
4000
2000
0
24.29
28.05
29.45
32.69
33.52
33.82
20 22 24 26 28 30 32 34 36 38
RT(min)
764.404
877.489
934.510
1097.573
1212.600
Relative Intensity
m/z
A119 MS-QBIC
RT: 28.55
10000
5000
24.02
27.36
29.82
33.25 34.36
774.413
887.497
944.518
1107.582
1222.609
774.414

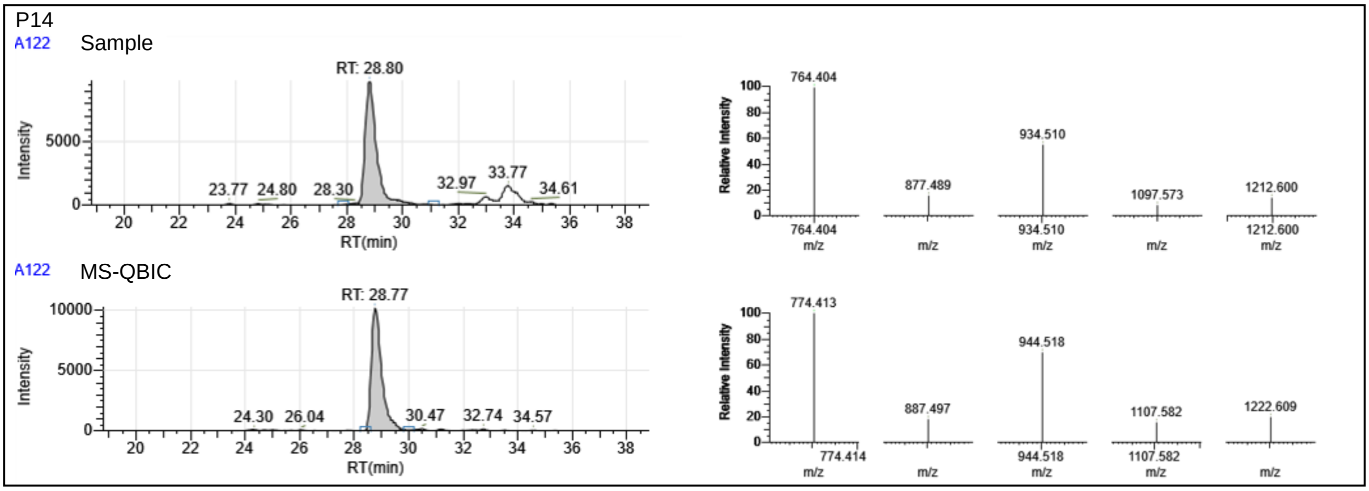
P14
A122 Sample
RT: 28.80
Intensity
5000
0
23.77 24.80 28.30
32.97
33.77
34.61
20 22 24 26 28 30 32 34 36 38
RT(min)
764.404
877.489
934.510
1097.573
1212.600
Relative Intensity
m/z
A122 MS-QBIC
RT: 28.77
10000
5000
24.30 26.04
30.47
32.74 34.57
774.413
887.497
944.518
1107.582
1222.609
774.414

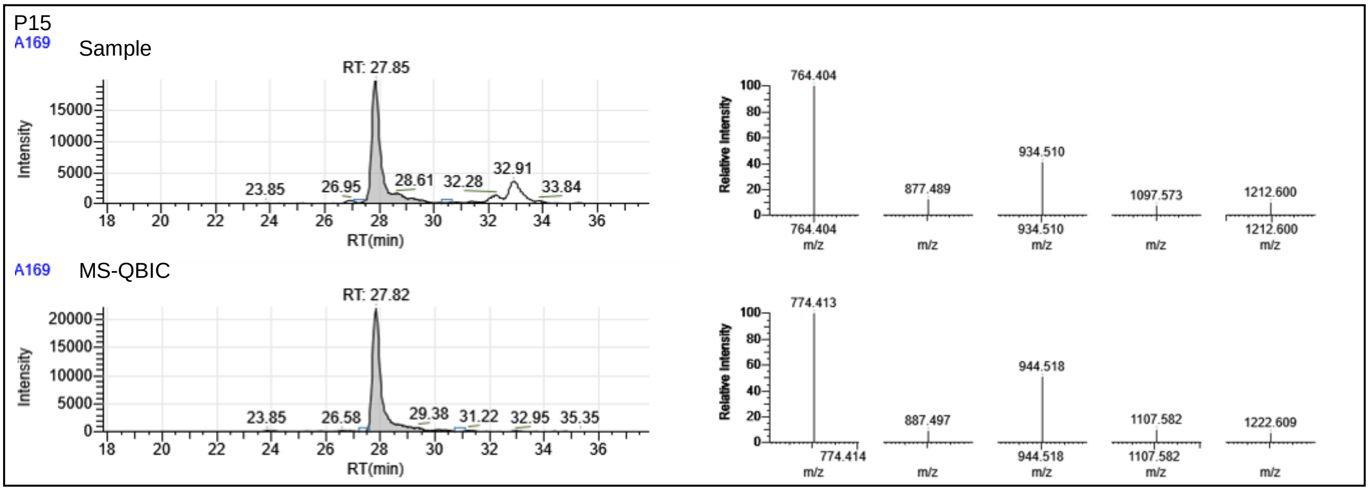
P15
A169 Sample
RT: 27.85
23.85 26.95 28.61 32.28 32.91 33.84
Intensity
RT(min)
764.404
934.510
877.489
1097.573
1212.600
Relative Intensity
m/z
A169 MS-QBIC
RT: 27.82
23.85 26.58 29.38 31.22 32.95 35.35
774.413
944.518
887.497
1107.582
1222.609
774.414

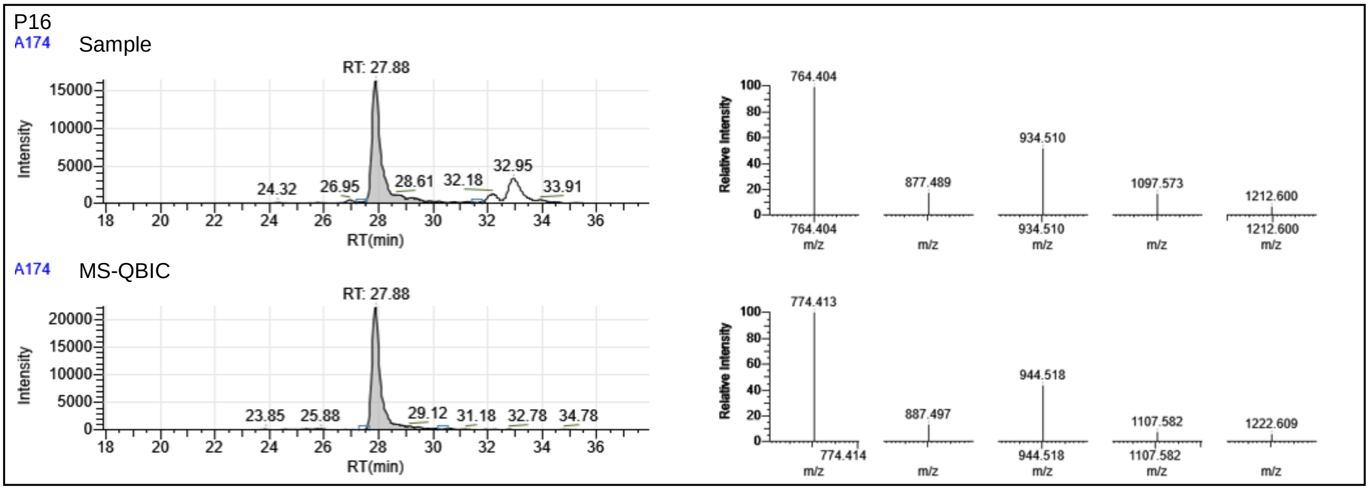
P16
A174 Sample
RT: 27.88
24.32 26.95 28.61 32.18 32.95 33.91
Intensity
RT(min)
764.404
934.510
877.489
1097.573
1212.600
Relative Intensity
m/z
A174 MS-QBIC
RT: 27.88
23.85 25.88 29.12 31.18 32.78 34.78
774.413
944.518
887.497
1107.582
1222.609
774.414

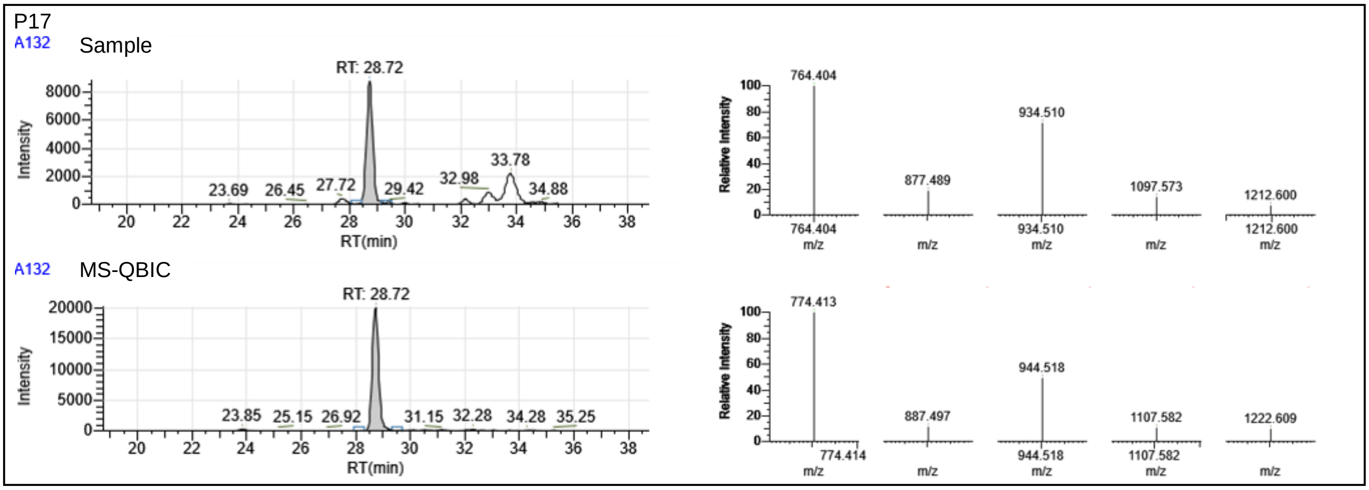
P17
A132 Sample
RT: 28.72
23.69 26.45 27.72 29.42 32.98 33.78 34.88
Intensity
RT(min)
764.404
934.510
877.489
1097.573
1212.600
Relative Intensity
m/z
A132 MS-QBIC
RT: 28.72
23.85 25.15 26.92 31.15 32.28 34.28 35.25
774.413
944.518
887.497
1107.582
1222.609
774.414

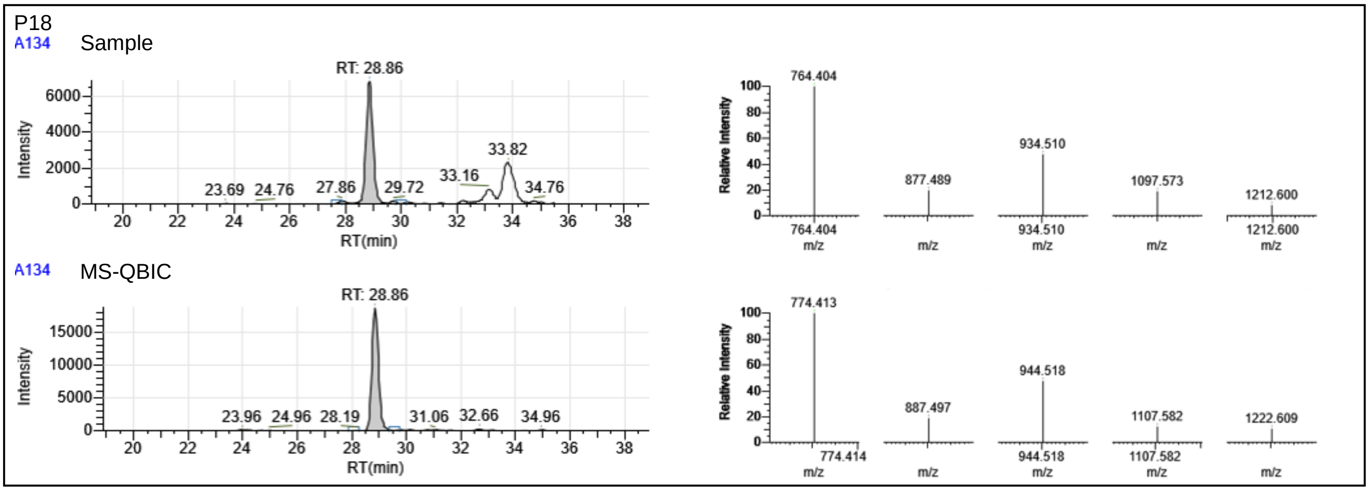
P18
A134 Sample
RT: 28.86
23.69 24.76 27.86 29.72 33.16 33.82 34.76
Intensity
RT(min)
764.404
934.510
877.489
1097.573
1212.600
Relative Intensity
m/z
A134 MS-QBIC
RT: 28.86
23.96 24.96 28.19 31.06 32.66 34.96
774.413
944.518
887.497
1107.582
1222.609
774.414

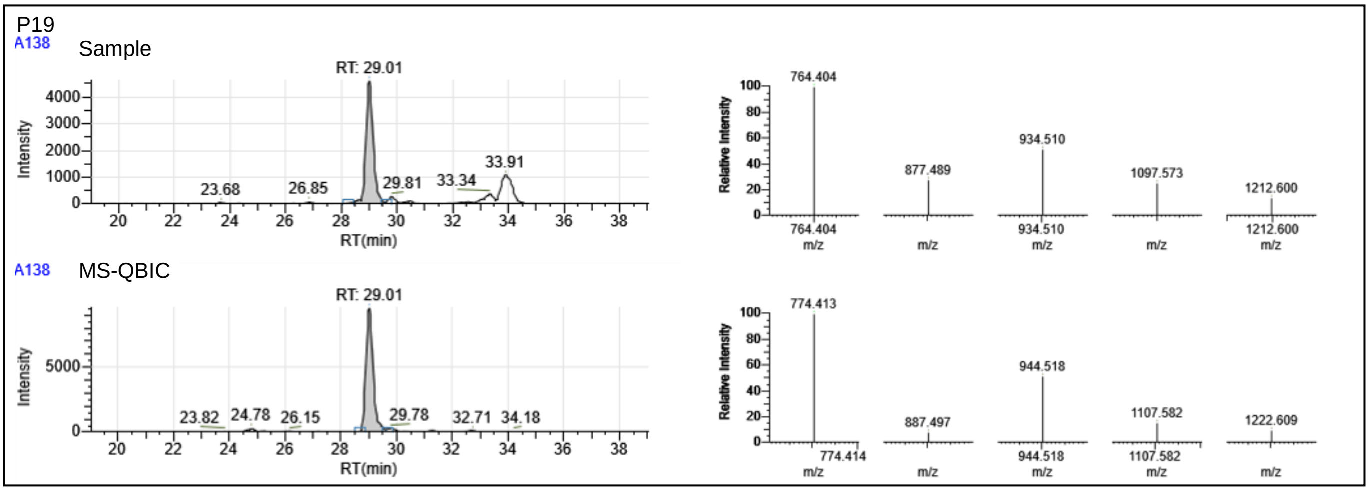
P19
A138 Sample
RT: 29.01
23.68 26.85 29.81 33.34 33.91
764.404
877.489
934.510
1097.573
1212.600
A138 MS-QBIC
RT: 29.01
23.82 24.78 26.15 29.78 32.71 34.18
774.413
774.414
887.497
944.518
1107.582
1222.609
Intensity
RT(min)
Relative Intensity
m/z

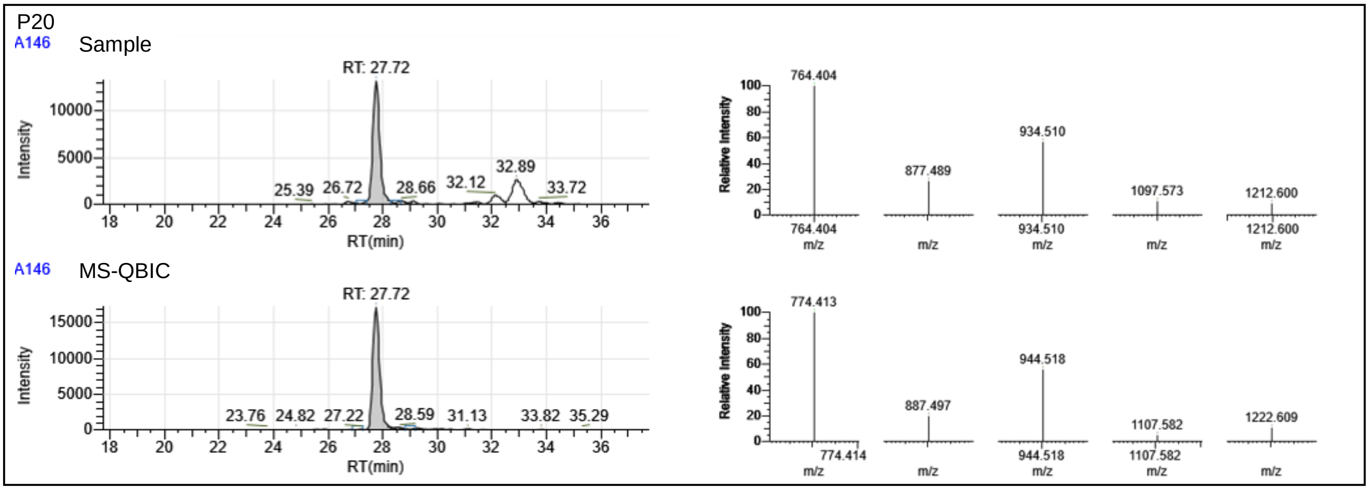
P20
A146 Sample
RT: 27.72
25.39 26.72 28.66 32.12 32.89 33.72
764.404
877.489
934.510
1097.573
1212.600
A146 MS-QBIC
RT: 27.72
23.76 24.82 27.22 28.59 31.13 33.82 35.29
774.413
774.414
887.497
944.518
1107.582
1222.609
Intensity
RT(min)
Relative Intensity
m/z

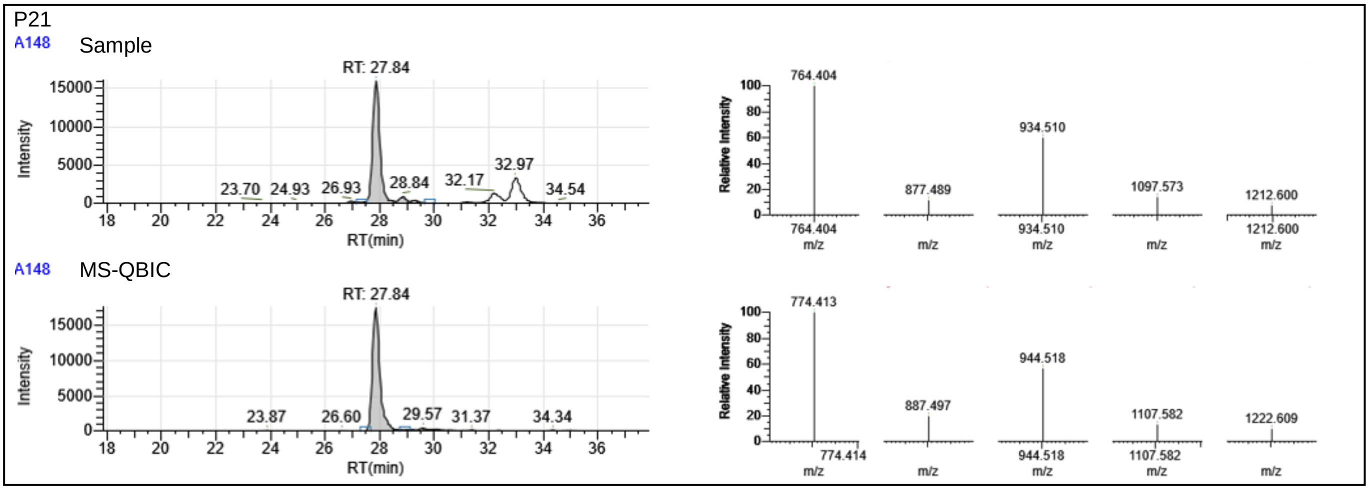
P21
A148 Sample
RT: 27.84
23.70 24.93 26.93 28.84 32.17 32.97 34.54
764.404
877.489
934.510
1097.573
1212.600
A148 MS-QBIC
RT: 27.84
23.87 26.60 29.57 31.37 34.34
774.413
774.414
887.497
944.518
1107.582
1222.609
Intensity
RT(min)
Relative Intensity
m/z

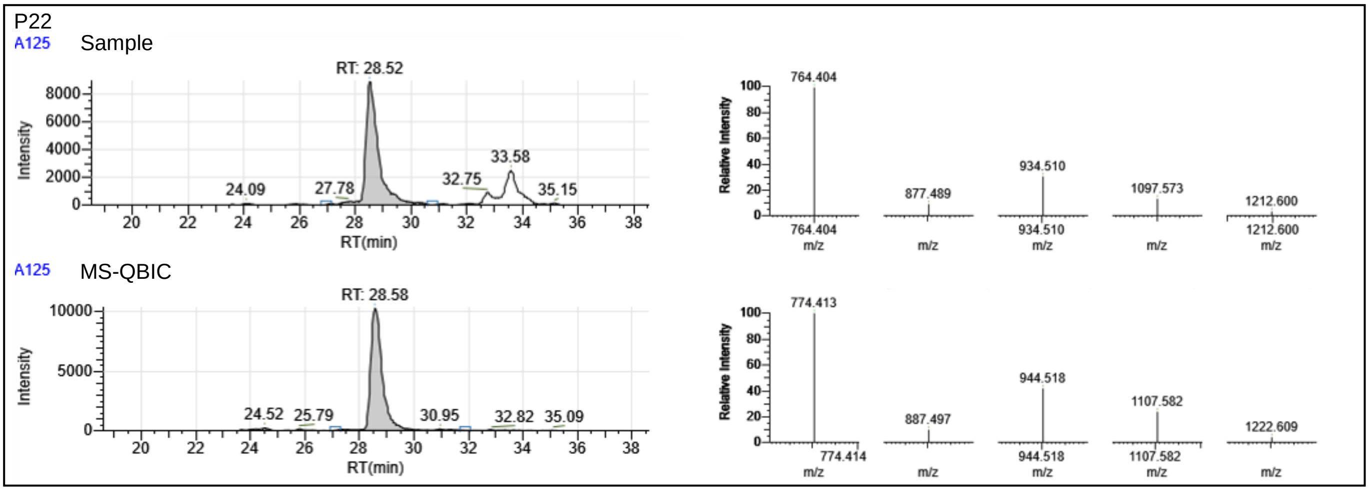
P22
A125 Sample
RT: 28.52
24.09 27.78 32.75 33.58 35.15
764.404
877.489
934.510
1097.573
1212.600
A125 MS-QBIC
RT: 28.58
24.52 25.79 30.95 32.82 35.09
774.413
774.414
887.497
944.518
1107.582
1222.609
Intensity
RT(min)
Relative Intensity
m/z

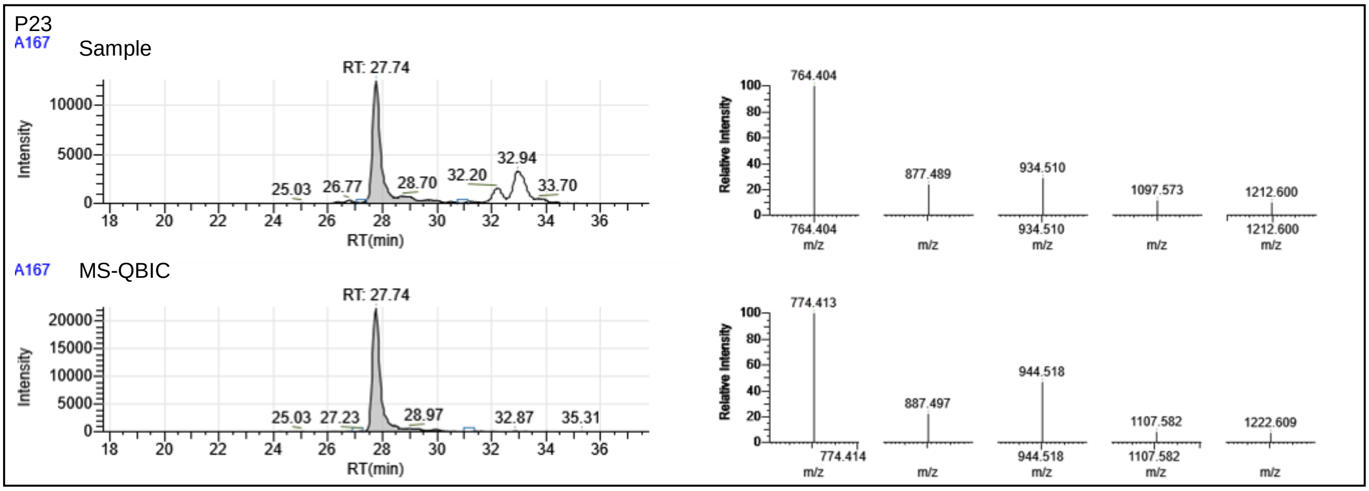
P23
A167 Sample
RT: 27.74
25.03 26.77 28.70 32.20 32.94 33.70
Intensity
RT(min)
764.404 877.489 934.510 1097.573 1212.600
Relative Intensity
m/z
A167 MS-QBIC
RT: 27.74
25.03 27.23 28.97 32.87 35.31
774.413 887.497 944.518 1107.582 1222.609
774.414

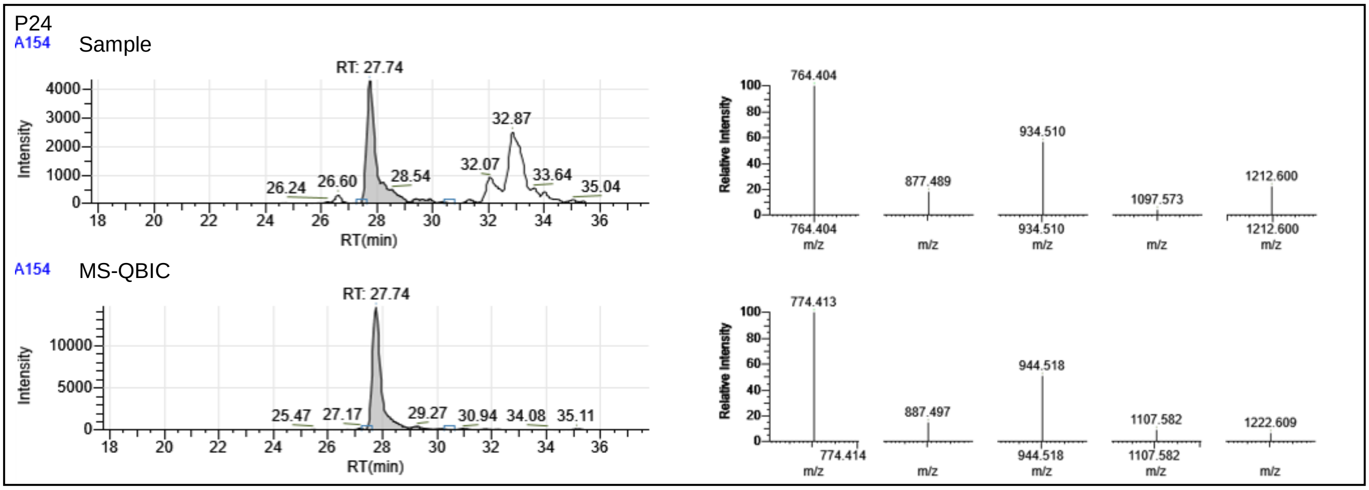
P24
A154 Sample
RT: 27.74
26.24 26.60 28.54 32.07 32.87 33.64 35.04
Intensity
RT(min)
764.404 877.489 934.510 1097.573 1212.600
Relative Intensity
m/z
A154 MS-QBIC
RT: 27.74
25.47 27.17 29.27 30.94 34.08 35.11
774.413 887.497 944.518 1107.582 1222.609
774.414

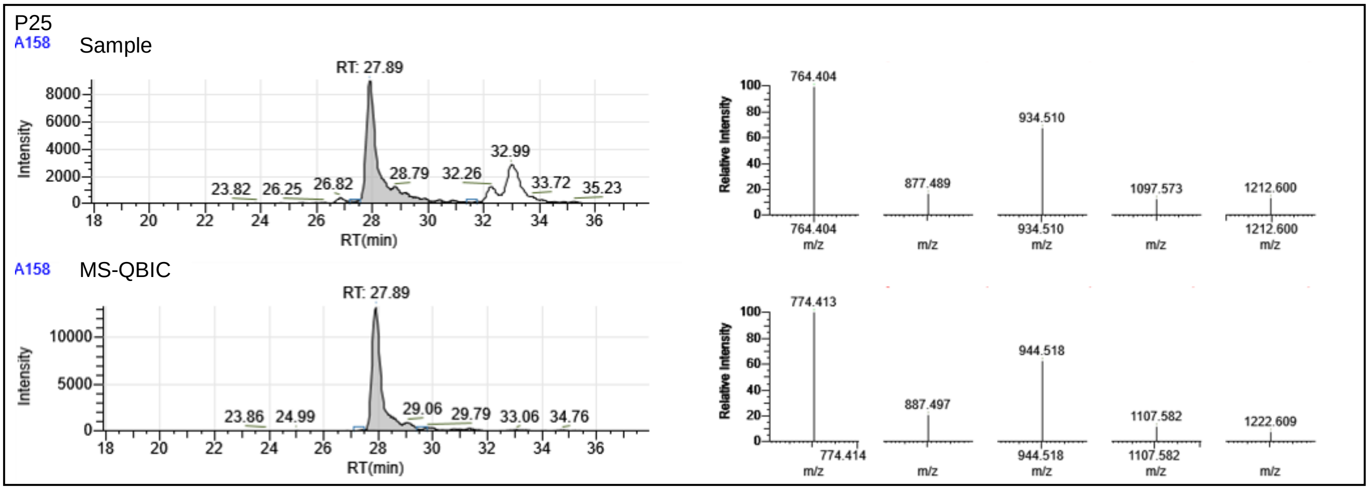
P25
A158 Sample
RT: 27.89
23.82 26.25 26.82 28.79 32.26 32.99 33.72 35.23
Intensity
RT(min)
764.404 877.489 934.510 1097.573 1212.600
Relative Intensity
m/z
A158 MS-QBIC
RT: 27.89
23.86 24.99 29.06 29.79 33.06 34.76
774.413 887.497 944.518 1107.582 1222.609
774.414

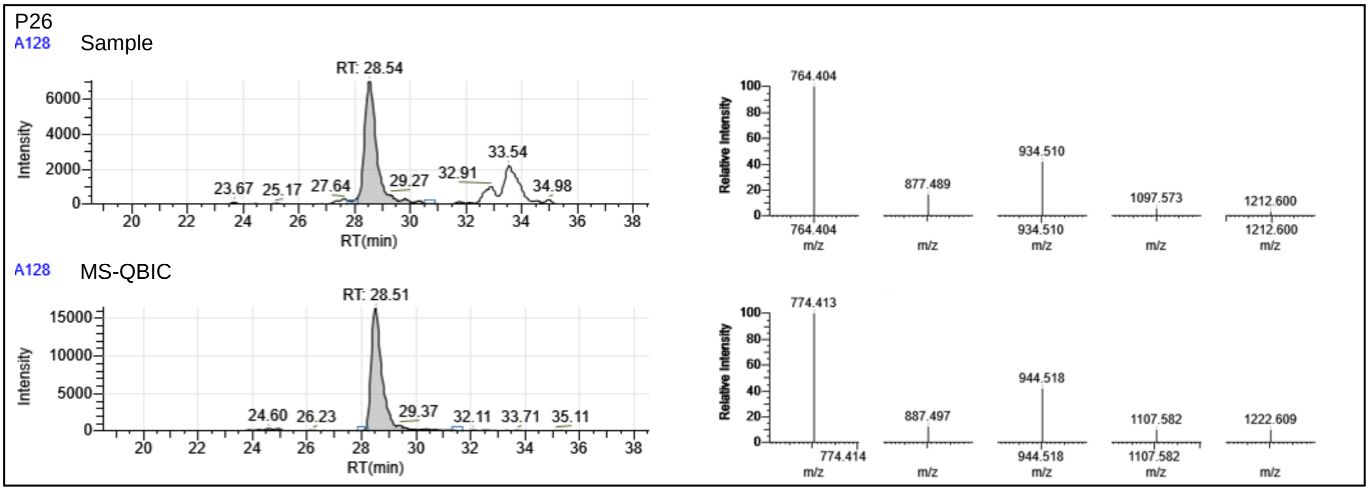
P26
A128 Sample
RT: 28.54
23.67 25.17 27.64 29.27 32.91 33.54 34.98
Intensity
RT(min)
764.404 877.489 934.510 1097.573 1212.600
Relative Intensity
m/z
A128 MS-QBIC
RT: 28.51
24.60 26.23 29.37 32.11 33.71 35.11
774.413 887.497 944.518 1107.582 1222.609
774.414

P27

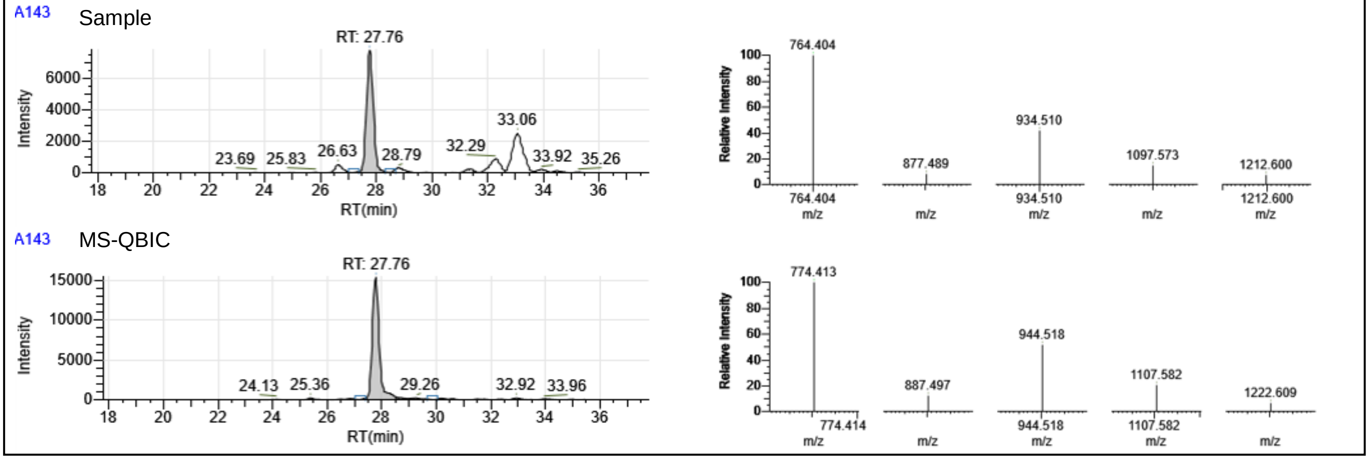

P28

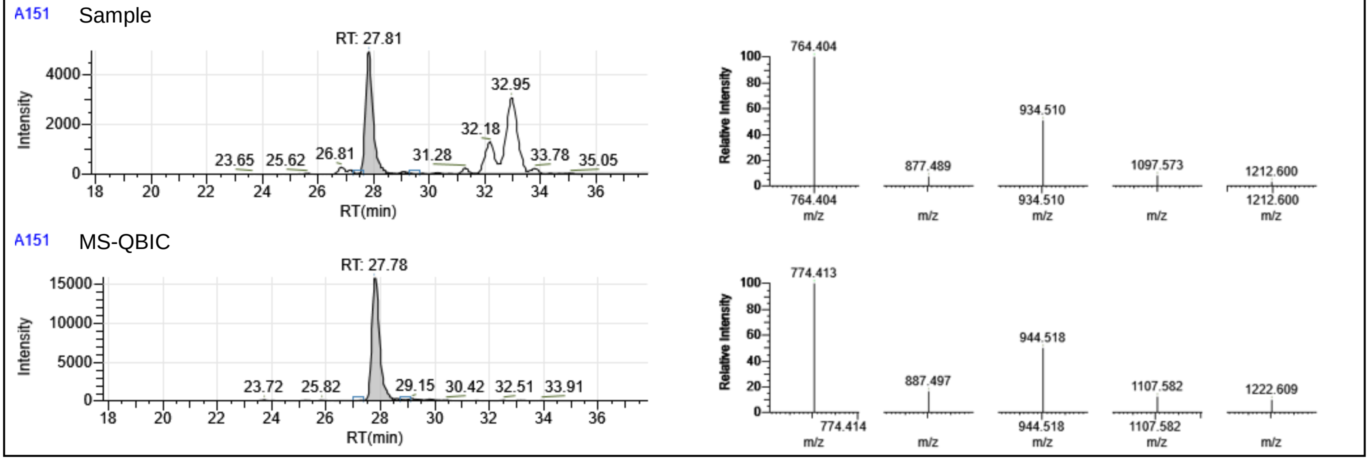

P29

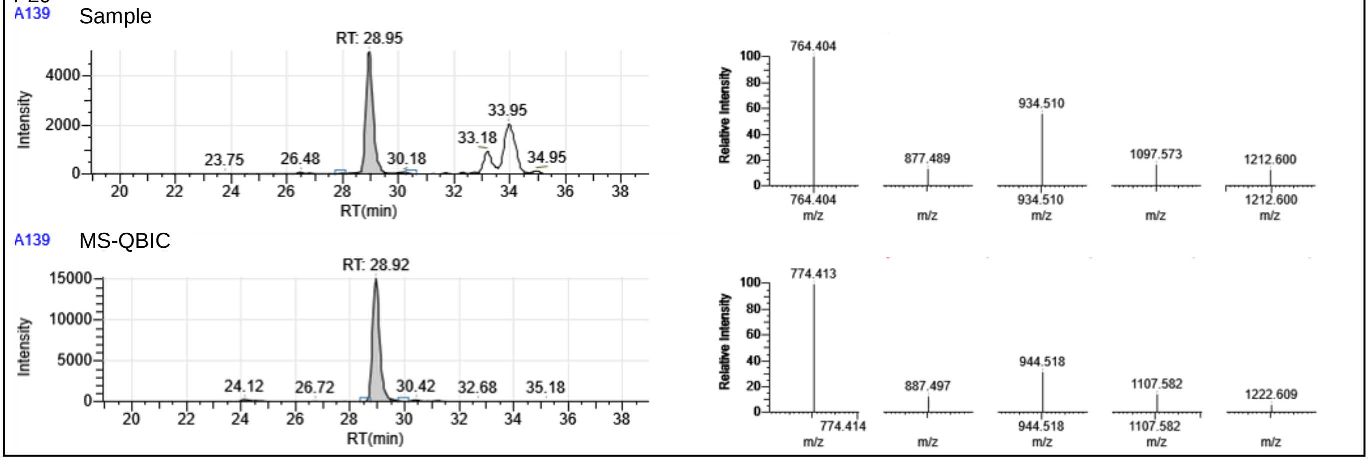

P30

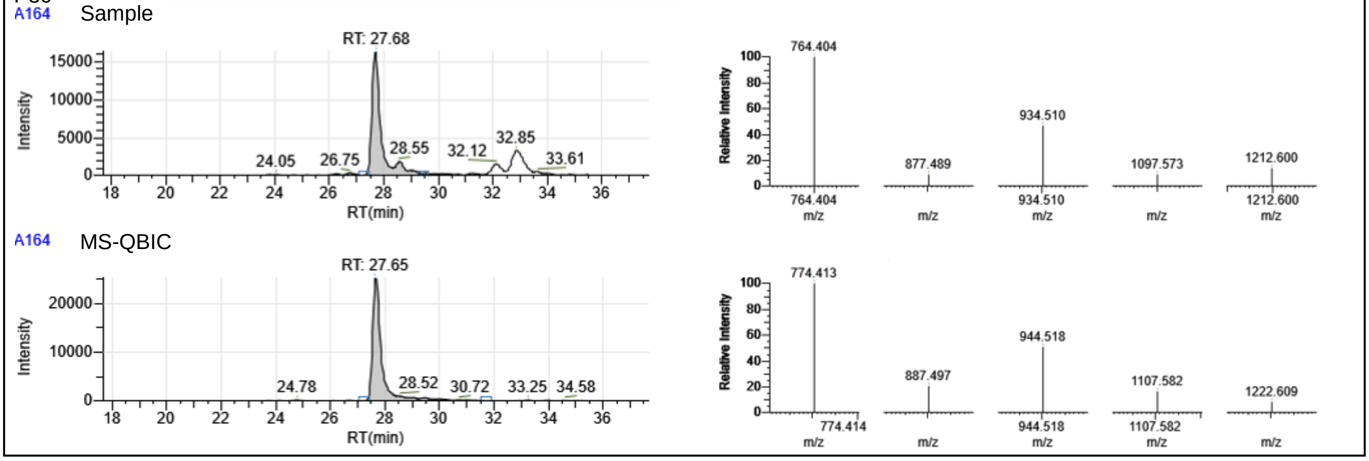

Supplement: S3 Appendix — Case ID (e.g., P1) and raw file ID (e.g., A003) correspond to the ID numbers shown in S3 Table. (A) and (M) serum amyloid A (SAA). (B) and (N) transthyretin (TTR), (C) and (O) immunoglobulin kappa light chain (IGK), (D) and (P) immunoglobulin lambda light chain (IGL), (E) and (Q) beta-2-microglobulin (B2M), (F) and (R) apolipoprotein (Apo) A1, (G) and (S) Apo A2, (H) and (T) Apo A4, (I) and (U) Apo E, (J) and (V) gelsolin, (K) and (W) fibrinogen alpha chain (FGA), (L) and (X) lysozyme, (A-L) specimens are derived from the heart, and (M-X) from other organs. (PDF) [file pone.0235143.s003.pdf]
