# Supplementary material for: Mass spectrometry-based absolute quantification of amyloid proteins in pathology tissue specimens: Merits and limitations
Source: PLoS One. 2020 Jul 1;15(7):e0235143. doi: 10.1371/journal.pone.0235143 (PMC7329117; doi:10.1371/journal.pone.0235143)
Supplement: S1 Fig — The amount of Congo red-positive deposition was scored in five grades 1–5. from minimal to severe (A, C, E). The staining is well visualized under the FITC filter of a BZ-X710 all-in-one fluorescent microscope (B, D, F). Grade 2 (A and B), Grade 3 (C and D), and Grade 5 (E and F). Scale bars, 200μm. (PDF) [file pone.0235143.s004.pdf]

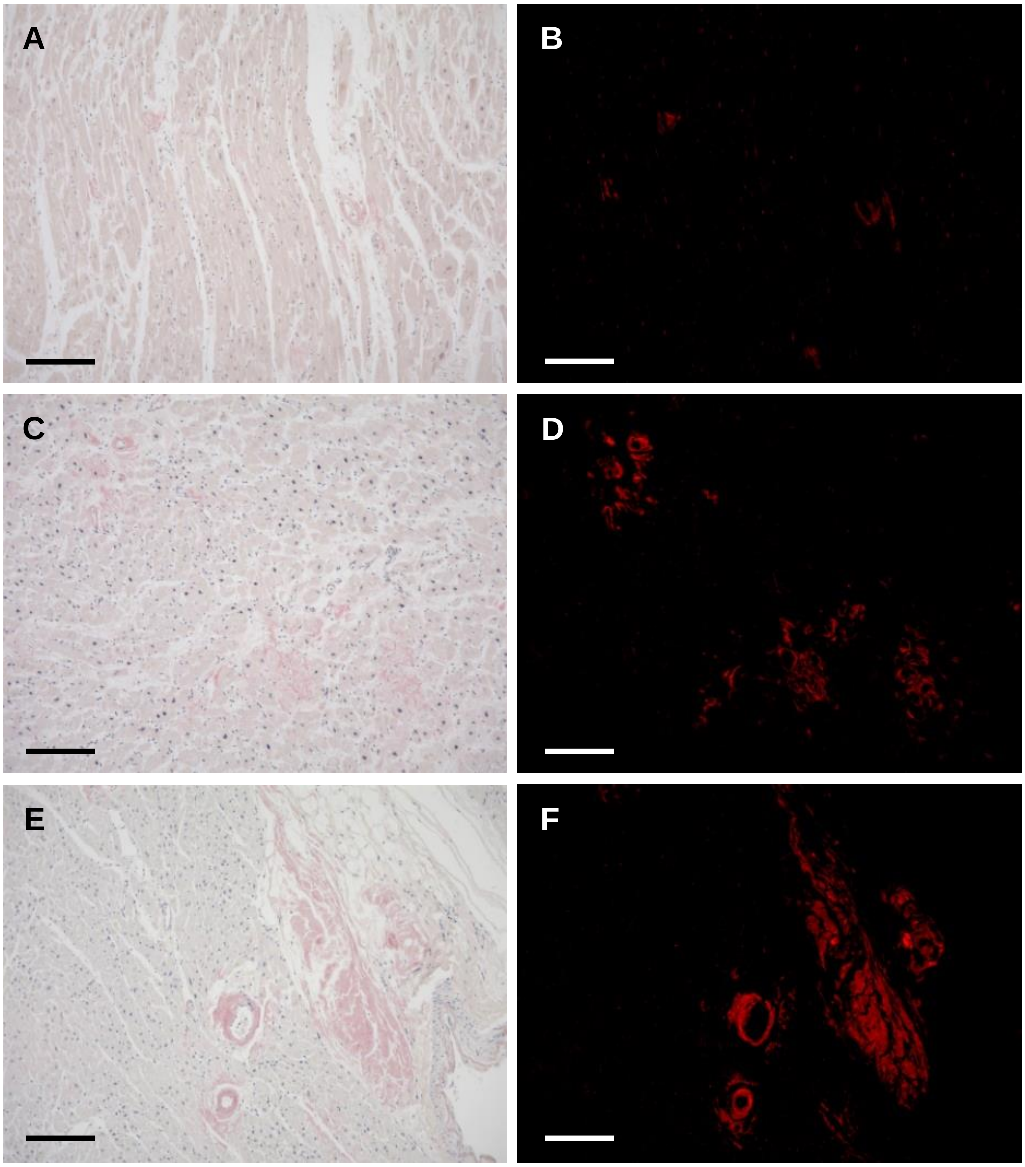

S1 Fig. Amyloid deposition by Congo red staining.

The amount of Congo red-positive deposition was scored in five grades 1 - 5. from minimal to severe (A, C, E). The staining was well visualized under the FITC filter of a BZ-X710 all-in-one fluorescent microscope (B, D, F). Grade 2 (A and B), Grade 3 (C and D), and Grade 5 (E and F). Scale bars, 200μm
