# Supplementary material for: Mass spectrometry-based absolute quantification of amyloid proteins in pathology tissue specimens: Merits and limitations
Source: PLoS One. 2020 Jul 1;15(7):e0235143. doi: 10.1371/journal.pone.0235143 (PMC7329117; doi:10.1371/journal.pone.0235143)
Supplement: S2 Fig — The workflow has two parts: the synthesis and quantification of the MS-QBIC peptides (left side) and the quantification of target peptides using the MS-QBIC peptide as reference (right side). (PDF) [file pone.0235143.s005.pdf]

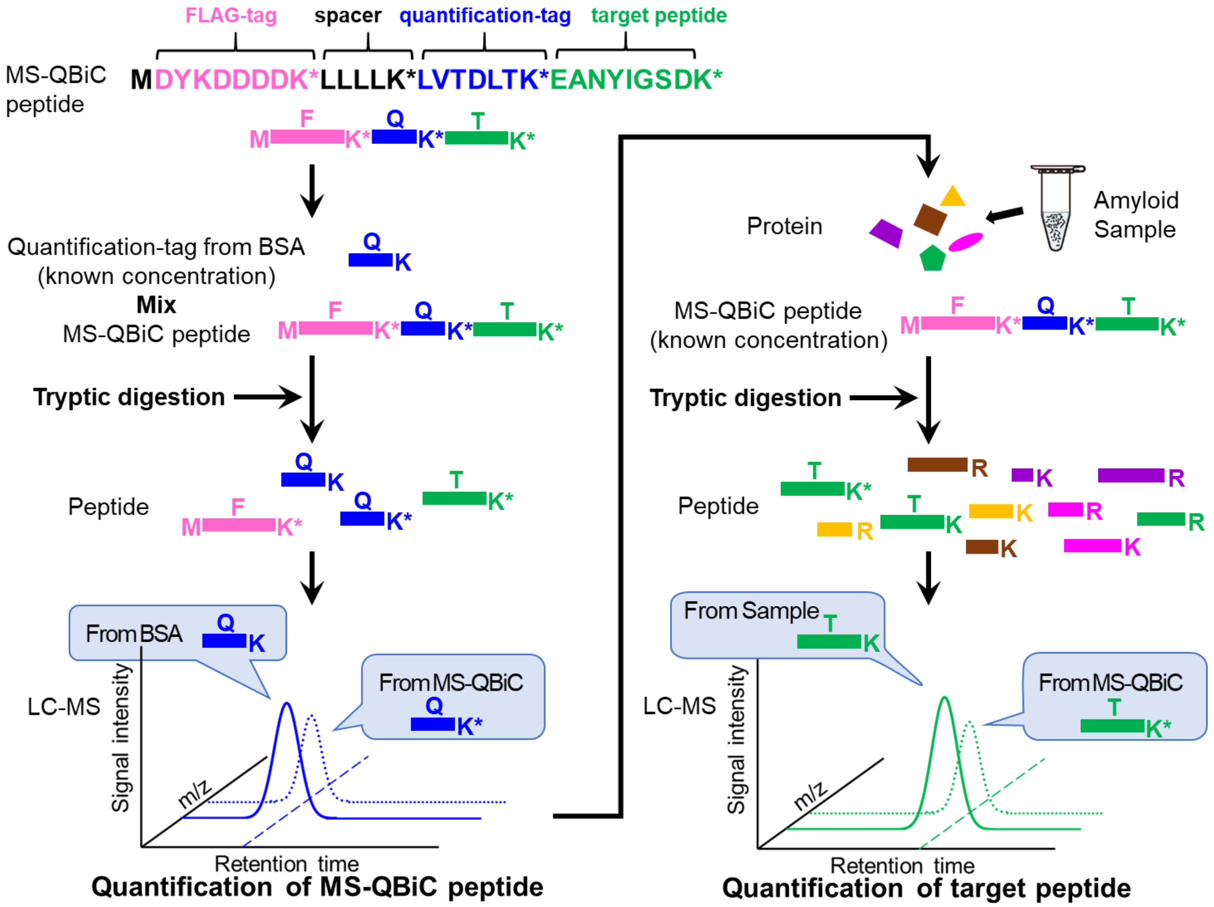

S2 Fig. Workflow of quantification of amyloid proteins by mass spectrometry–based quantification by isotope-labeled cell-free products (MS-QBIC).
The workflow has two parts: the synthesis and quantification of the MS-QBIC peptides (left side) and the quantification of target peptides using the MS-QBIC peptide as reference (right side).
