# Supplementary material for: Mass spectrometry-based absolute quantification of amyloid proteins in pathology tissue specimens: Merits and limitations
Source: PLoS One. 2020 Jul 1;15(7):e0235143. doi: 10.1371/journal.pone.0235143 (PMC7329117; doi:10.1371/journal.pone.0235143)
Supplement: S3 Fig — (A) Signal linearity of the quantification tag measured by mass spectrometry. (B) Heavy to light ratios of quantification tag signals used to estimate the concentrations of MS-QBIC peptides. (PDF) [file pone.0235143.s006.pdf]

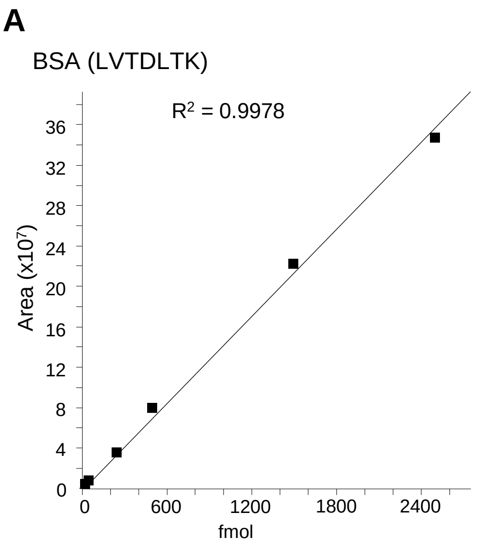

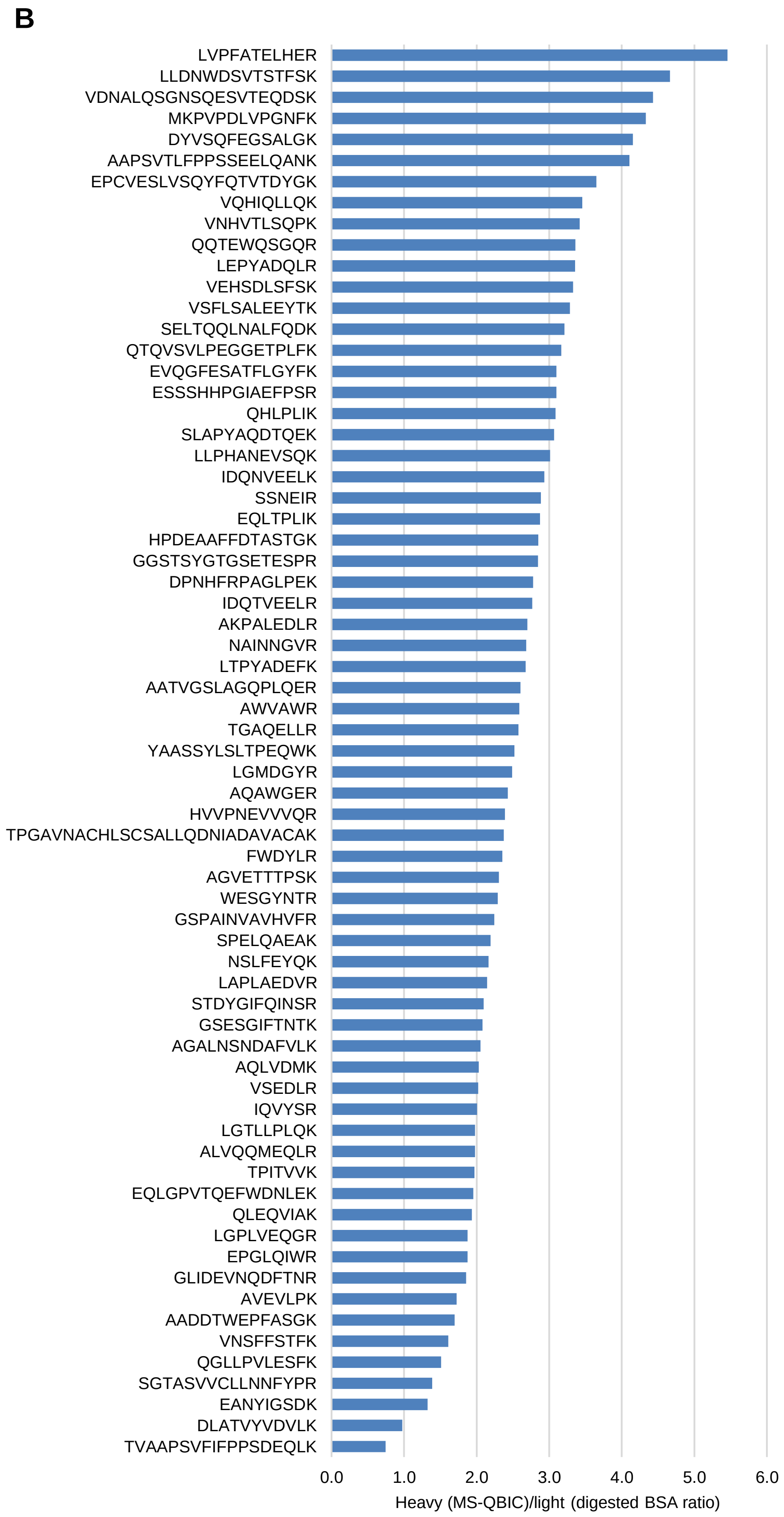

S3 Fig. Supporting information for the estimation of the concentration of MS-QBIC peptides

A) Signal linearity of the quantification tag measured by mass spectrometry.

B) Heavy to light ratios of quantification tag signals used to estimate the concentration of MS-QBIC peptides.
