# Supplementary material for: Mass spectrometry-based absolute quantification of amyloid proteins in pathology tissue specimens: Merits and limitations
Source: PLoS One. 2020 Jul 1;15(7):e0235143. doi: 10.1371/journal.pone.0235143 (PMC7329117; doi:10.1371/journal.pone.0235143)
Supplement: S1 Table — (DOCX) [file pone.0235143.s007.docx]

**S1 Table. Specimens used for data-dependent MS/MS analysis**

| **Disease** | **Specimens** |
| --- | --- |
| Systemic amyloidosis |  |
| AA amyloidosis | Thyroid, Heart |
| ATTR amyloidosis | Heart (n=4) *^1, 2^, Tongue*^1^, Esophagus*^2^ |
| Amyloid light chain (kappa) amyloidosis | Pancreas, Kidney, Heart*^3^, Peritoneum*^3^ |
| Amyloid light chain (lambda) amyloidosis | Liver*^4^, Kidney*^4^ |
| Beta-2-microglobulin amyloidosis | Synovium*5, Skeletal muscle*5 |
| Gamma heavy chain disease | Lymph node |
|  |  |
| Local amyloidosis |  |
| Diabetes mellitus | Pancreas (n=4) *^6^ |
| Alzheimer disease | Brain (n=3) |
|  |  |
| Other disease |  |
| Multiple myeloma | Bone marrow (n=2) *^6^ |

The number of specimens is one, unless otherwise described.

*^1-6^, the organs of each number were derived from the same patient, respectively.
