# Supplementary material for: Mass spectrometry-based absolute quantification of amyloid proteins in pathology tissue specimens: Merits and limitations
Source: PLoS One. 2020 Jul 1;15(7):e0235143. doi: 10.1371/journal.pone.0235143 (PMC7329117; doi:10.1371/journal.pone.0235143)
Supplement: Attachment — Submitted filename: PONE_revise2.docx [file pone.0235143.s011.docx]

**PONE-D-20-02311**

**Mass spectrometry–based absolute quantification of amyloid proteins in pathology tissue specimens: merits and limitations**

**Responses to Reviewers’ Comments**

We greatly appreciate the critical comments and useful suggestions for our submission from reviewer #1.

**Point-by-point responses**

The reviewer’s comments are italicized and enclosed in square brackets. Modifications in the revised manuscript are highlighted in red.

*Line 203, 429: The authors refer to the Q-Exactive mass spectrometer as “equipment”. The term instrument is more commonly used.]*

As the reviewer pointed out, we have corrected “equipment” to “instrument” in

Revised manuscript, Line 197, and Revised manuscript, Line 386.

*[Line 410: “CD2O and heavy 13CD2O isotopes, respectively, through dimethyl labeling with” Should it be “CH2O and heavy 13CD2O isotopes, respectively, through dimethyl labeling with” Are the authors using CH2O (+0 Da), CD2O (+2 Da), and 13CD2O (+3 Da) or CH2O (+0 Da) and 13CD2O (+ 3 Da) in their analysis? Lines 424-425 suggest the latter is the Authors’ intent.*

We are very sorry for our mistake, and we have corrected “CD2O” to “CH2O”:

Revised manuscript, Line 367

Nonamyloid and amyloid samples were differently labeled with light CH2O and heavy CD2O isotopes, respectively

*[Line 412: “The interstitial tissues of 11 specimens without amyloidosis” “interstitial” seem to be the incorrect word for the sentence.]*

As the reviewer pointed out, we changed it as follows;

Revised manuscript, Line 369

The stromal components of 11 specimens devoid of amyloid (derived from seven patients with conditions unrelated to amyloidosis) were micro-dissected using LMD.

*[Lines 450-456: 6+7+2+5+6 =26 Should it be 26 of 30 or 24 of 30, but some patients had two forms? Compare with lines 502-507: 7+7+4+5+1=24.]*
We are very sorry for the confusing description.

Revised manuscript, Lines 406-412:

In 24 of the 30 patients, amyloidosis was subclassified by clinical and pathological examination at autopsy, based on immunohistochemical criteria. Seven patients were AA amyloidosis. Of the seven patients with ATTR/TTR amyloidosis, two patients had hereditary disease, and five patients had acquired disease. Six patients had associated multiple myeloma, and three patients were diagnosed as primary or systemic amyloidosis. One patient had dialysis related amyloidosis.

*[Lines 606-608: An awkward sentence that should be revised: “For setting the cut-off value for MS-quantification in the present study, we used a simulation to obtain the cut-off value as shown in Table 4.”]*

We are very sorry for the awkward sentence. We change the sentence as follows.

Revised manuscript, Lines 408-415:

For setting the cut-off value for MS-quantification in the present study, we used a simulation as shown in Table 4.

*[Lines 414, 592, 724: It is Sjögren or Sjögren’s syndrome]*

We have corrected “Sjogren” and “Sjögren’s” to “Sjögren” (Revised manuscript, Table 1 and line 540).
